# TreeSnatcher plus: capturing phylogenetic trees from images

**DOI:** 10.1186/1471-2105-13-110

**Published:** 2012-05-24

**Authors:** Thomas Laubach, Arndt von Haeseler, Martin J Lercher

**Affiliations:** 1 Department of Bioinformatics, Heinrich-Heine-University Duesseldorf, Universitaetsstrasse 1, Duesseldorf 40225, Germany; 2 Center for Integrative Bioinformatics Vienna, Max F Perutz Laboratories, Dr-Bohr-Gasse 9, Vienna, Austria; 3 University of Vienna, Vienna, Austria; 4 Medical University Vienna, Vienna, Austria

**Keywords:** Newick format, Phylogenetic tree recognition, Image digitization, Phylogeny preservation

## Abstract

**Background:**

Figures of phylogenetic trees are widely used to illustrate the result of evolutionary analyses. However, one cannot easily extract a machine-readable representation from such images. Therefore, new software emerges that helps to preserve phylogenies digitally for future research.

**Results:**

TreeSnatcher Plus is a GUI-driven JAVA application that semi-automatically generates a Newick format for multifurcating, arbitrarily shaped, phylogenetic trees contained in pixel images. It offers a range of image pre-processing methods and detects the topology of a depicted tree with adequate user assistance. The user supervises the recognition process, makes corrections to the image and to the topology and repeats steps if necessary. At the end TreeSnatcher Plus produces a Newick tree code optionally including branch lengths for rectangular and freeform trees.

**Conclusions:**

Although illustrations of phylogenies exist in a vast number of styles, TreeSnatcher Plus imposes no limitations on the images it can process with adequate user assistance. Given that a fully automated digitization of all figures of phylogenetic trees is desirable but currently unrealistic, TreeSnatcher Plus is the only program that reliably facilitates at least a semi-automatic conversion from such figures into a machine-readable format.

## Background

Every scientific field that processes information with the aid of computers needs to maintain and preserve its technical illustrations in a machine-readable fashion for later reuse. For this, the structure of an illustration has to be decomposed into its geometric primitives. The more previous knowledge of the image content is available to the computer, the less errors will occur during the decomposition process. Computer programs today digitally capture and archive decades-old architectural drawings, for which the usage of symbols, icons and font types is standardized. Conversely, no computer program can automatically convert arbitrarily shaped phylogenetic trees from an illustration into a machine-readable expression, e.g., the Newick format [1]. The styles in which phylogenetic trees have been published are as manifold as are the software packages used for the creation of the trees and the pictures. A comprehensive list of such programs is published in [2]. Because there are no strict design rules, a program intended for the recognition of arbitrary trees must not assume any previous knowledge beyond the existence of a depicted phylogeny.

**Figure 1:**
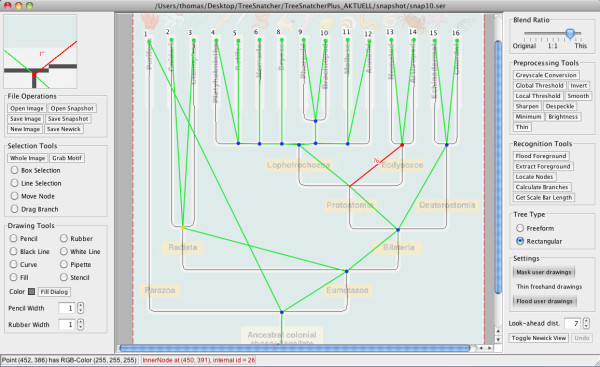
**TreeSnatcher Plus main screen.** The default view is a blend between the current processing state and the original image. Here, after gray-scale conversion, binarization and skeletonization, the thinned branches were manually complemented where text overlaps the tree. The program then recognized all tree node locations and measured the branch lengths. At this moment, the Newick expression can be calculated.

TreeSnatcher Plus (Figure 1) is not the first program aimed at the digitization of phylogenies. Indeed, TreeThief [3] was the first application that converts a tree image into a computer-readable representation of the tree. It allowed the user to digitize a tree by clicking on each of its nodes in turn. It is restricted to Apple Macintosh computers running Mac OS 9. In 2007, we presented TreeSnatcher [4], an application that identified the topology of an arbitrarily shaped tree (e.g., a figure from a publication) semi-automatically with user interaction. However, it required the user to pre-process an image using an external drawing package, to follow a strict succession of program stages and lacked any Undo functionality. Finally, the program TreeRipper by Joseph Hughes [5] automatically converts images of rectangular trees that fulfil a strict set of criteria into the Newick format. However, TreeRipper’s success rate is relatively low, with only about one third of sample images converted correctly [5].

Using already published trees in research projects would be trivial if all phylogeny related data were also published in open-access online repositories, e.g., TreeBASE [6], MorphoBank [7], or Dryad [8]. Leebens-Mack et al. [9] propose a roadmap for the development of minimal reporting standards for phylogenetic analyses, MIAPA (Minimal Information about a Phylogenetic Analysis). They maintain a website on which they discuss potential barriers to re-use data from scientific analyses [10]. In principle, electronic data could also be obtained from the authors. This obvious approach appears not to be practical. In an example from another field of study, 73% of the authors refused to share their data when approached [11].

Thus, to reuse most published phylogenetic results, it appears that reliable digitization of tree images is currently the only realistic option.

### Implementation

TreeSnatcher Plus is an extended and fully re-conceptualized version of TreeSnatcher; it is both easier to use and more accurate than its predecessor. The new program features a graphical user interface that is based on the JAVA Swing API. A more flexible workflow is complemented with multiple Undo functionality and the possibility to restore the program state ('snapshot'). The user can now pre-process any image within TreeSnatcher Plus, selecting from a full range of pre-processing tools. The current state of processing can be saved as an image that may contain different layers of visual information. The program calculates the branch lengths in freeform and skewed rectangular trees and can mix calculated and user defined branch lengths. Additionally, the user can modify an existing tree or to construct a new tree.

The application opens image files in the formats PNG, JPG/JPEG or GIF. The PDF format is currently not supported, but tools for the extraction of images from PDF documents are readily available (e.g., the Xpdf suite for Linux operating systems [12]). The program offers the following pre-processing tools, most of which were modified from standard algorithms [13]: pencil, rubber, line, fill, stencil, histogram stretch, colour reduction, gray-scale conversion, local and global thresholding, colour manipulation, inversion, median and minimum filter, blurring and sharpening, lightening and darkening, and thinning [14].

Prior to automated node placement, the user has to prepare the image within TreeSnatcher Plus, analogous to the requirements of its predecessor [4]. In particular, the tree has to be converted into a line drawing without intersections with text or graphics unrelated to the tree topology. If the image does not meet those requirements when the automatic node placement is issued, the tree topology is unlikely to be identified correctly.

Working with TreeSnatcher Plus takes place along a general succession of global tasks, which are executed at least once, either on the whole image or on parts. The user supervises all image manipulations and recognition tasks performed by the program, makes corrections and repeats steps if necessary. This process is explained in detail in the tutorials accompanying the program.

The workflow is thus as follows:

1. The program reads the specified image file. The user trims and cuts the image at will. In this way, one can select sub-trees or a subset of taxa from the image.

2. Image pre-processing: The user prepares the image with the pre-processing tools.

3. Binarization: The user thresholds the image to ensure that the foreground is black and the background is white and both are clearly separated.

4. Skeletonization: The user semi-automatically thins the foreground of the image portion that contains the tree. This is necessary to enable the program to find the paths between the line intersections (step 8).

5. Foreground flooding: The user marks a position in the tree. The program colours ('floods') the foreground reachable from there. In subsequent steps, the flooded area will be treated as the tree. Everything else is ignored.

6. Inner nodes and outer nodes placement: The program suggests locations for line intersections and end of lines. These represent branching locations and tips. A logical node is assigned to each location. The user can move, remove and add nodes. In the thinned image, black pixels adjacent to exactly one other black pixel become a tip location. Black pixels adjacent to at least three black pixels are candidates for a branching location. If several candidate pixels are adjacent to each other, the branching location is averaged from their positions.

7. Choice of tree type: The program can distinguish and calibrate freeform and rectangular trees. The choice of the tree type influences how the program treats branch lengths. The tree type must be chosen prior to step 8.

8. Recognition of branches: The program traces gapless foreground paths between each pair of nodes in order to find the branches of the tree. If there are several candidate paths, the shortest is selected. If a branch is missing or wrong, the user either modifies the image with the drawing tools and repeats step 8, or he/she drags a new branch and manually specifies its length.

9. Determination of branch lengths: The accuracy depends on the congruence of thinned tree structure, node placement, and original tree. For freeform trees, the branch length in pixels is based on the entire foreground path between the two defining nodes. For rectangular trees, the branch length is the sum of the lengths of the horizontal path segments. The user may type in self-defined branch lengths and mix them with the calculated lengths. The tree can be scaled using a line of known length in the image, e.g., a scale bar.

10. Assignment of species names: The user right-clicks on each leaf node in turn in order to type in the corresponding species name.

11. Choice of the tree root: The program, assisted by the user if necessary, chooses the inner node based on which the rooted Newick expression is calculated.

12. Construction of the Newick string: The program calculates and displays the Newick tree code for the tree depicted. The user may save it to the clipboard or export it into a text file.

## Results

An image that shows a uniformly dark, rectangular phylogenetic tree on a uniformly light background in sufficient resolution, without foreground elements overlapping with the tree, will need almost no pre-processing. If the user then settles for consecutively numbered tip labels, the whole recognition process can be finished within minutes. However, in general there will be image portions which require manual correction, e.g., a branch of the tree is not clearly separated from other foreground elements such as lettering. TreeSnatcher Plus offers a special tool that surrounds black drawings with a white border.

For the determination of branch lengths, the program needs to assess the path length in pixels between branching positions on the skeletonized foreground. Additionally, it must reliably detect bends in a branch and horizontal branch portions. These tasks work better if the structures come with a sufficiently high number of pixels. The better the branches in the original image and those in the skeletonized image align, the more accurate are the branch lengths.

The time needed for the complete tree digitization depends on several factors, among them image size and quality, tree size and complexity, whether or not branch lengths are desired and species names are typed in, how much pre-processing is necessary, and how experienced the user is in the usage of TreeSnatcher Plus.

We benchmarked TreeSnatcher Plus using the set of 100 rectangular trees published by Hughes [5], nine additional images with non-standard tree styles, and another image modified from the benchmark set of Hughes. The topologies of all trees and their branch lengths were recognized correctly, with necessary user interaction ranging from minor to extensive (Additional file 1, table 'BenchmarkTreeSet'). All tip labels were typed manually. As TreeSnatcher Plus is not meant to work autonomously, the user is required to initiate all pre-processing and analysis steps. For different users, the time needed for a particular task may vary. For the benchmark set [5], the time needed for topology reconstruction was on average 165 s, ranging from 30 to 1,800 s on a PC equipped with a Core i7-960 processor. Typing the tip labels required on average 4.4 min, the minimum time was 0 min for an image without labels and the maximum time 35 min. The shortest tree digitisation job finished within one minute, the longest job required 45 min. Recognizing the topology of a tree with 100+ tips (image 1471-2148-6-93 in the tree set by Hughes) and entering the tip labels required 17 min. Using the TreeRipper web frontend [5], we obtained the topology and the tip labels for the same tree within five minutes, the processing time was 272 s. However, all tip labels needed manual correction.

Additionally, we processed nine images of non-rectangular trees (Additional file 1, table 'InternetTrees'). Again, we obtained the topologies and the branch lengths for all trees. TreeSnatcher Plus can measure the branch lengths in freeform trees but not in circular and polar trees. For three circular trees ('bustard', 'TreeofLife' and 'Phylogenetic_Tree_of_Life'), we therefore approximated the lengths of the horizontal branch portions with the 'Line Selection' tool. In one case ('vert_tree'), we needed to retrace the tree manually.

We rotated image 1471-2148-6-99-1 from the benchmark set by 6° in order to test how well the program can recognize the rectangular branch portions in the new image ('RotatedTree'). While the editing steps were similar for the original and the modified image, TreeSnatcher Plus failed to recognize 10 out of a total 81 horizontal branch portions, compared to 3 in the original image.

In general, the correction of overlapping structures in a tree and typing the branch lengths are the most demanding tasks. For some images, we needed several attempts until we found a suitable order of processing steps.

For all jobs, we provide TreeSnatcher Plus snapshot data that can be reloaded into the program in order to reproduce the recognition results (Additional file 2, Additional file 3, Additional file 4, Additional file 5, Additional file 6.

On the TreeSnatcher Plus project website, we offer tutorials and sample recognition projects for the application. They provide a comprehensive overview about the performance of the program and the expected effort with different illustration styles and topologies.

## Discussion

Given the performance of today's image analysis and OCR methods, two distinct strategies are feasible: either a fully-automated approach requiring one specific tree type and a fixed illustration style, or a semi-automated approach for arbitrary trees.

TreeRipper, which to our knowledge is the only currently available program with comparable functionality, aims at a fully automatic recognition. To achieve this, it restricts itself to rectangular trees fulfilling a set of stringent criteria. For the trees fulfilling these criteria in the same benchmark set analysed here, TreeRipper was able to recognise the topology correctly in 32% of cases without any manual pre-processing.

TreeSnatcher Plus, on the other hand, accepts the necessity for manual image pre-processing and thus achieves a 100% success rate. It allows the user to process virtually any phylogenetic tree, albeit sometimes with extensive user interaction. On average, extraction of one phylogenetic tree topology required less than three minutes. Currently, tip names have to be entered by hand - using OCR here is very difficult to implement, as the tip labels can be anywhere on the image and in any orientation. TreeSnatcher Plus is easy to install, the single prerequisite being a working Java 1.6 Runtime Environment.

Further improvements are planned. The next version of TreeSnatcher Plus will be able to compute branch lengths for circular tree topologies. Moreover, it is planned to include an experimental OCR option for the recognition of tip label names in rectangular trees. The thinning technique could be improved as it is not tailored specifically to TreeSnatcher Plus.

## Conclusions

Although TreeSnatcher Plus does not work fully automatically, it can be used to preserve virtually any phylogenetic tree for future research. Today, automatic digitization, let alone batch processing, of even a subclass of phylogenetic tree images including labels seems hardly realizable. We are nevertheless convinced that novel programs will recognize a large number of different tree topologies in diverse styles. They will combine classical methods from the field of pictorial pattern recognition and image segmentation with new approaches. Until then, TreeSnatcher Plus can be used, with an acceptable effort for the user, for the semi-automated recognition of arbitrarily shaped trees in images. If the images to be digitised fulfil its requirements, TreeRipper may instead be used for the automatic recognition of topologies.

A greater acceptance of increased minimum data reporting standards in phylogenetic research would guarantee that phylogenetic data communicated in future research papers is machine-readable and available to other scientists. It has been mandatory for years to publish DNA sequences electronically in one of the universally accepted formats, and one may hope that a similar requirement will be enforced by scientific journals for phylogenies.

## Availability and requirements

Project name: TreeSnatcher Plus: capturing phylogenetic trees from images

Project home page: http://www.cs.uni-duesseldorf.de/AG/BI/Software/treesnatcher/

Operating Systems: Windows, Mac OS X, Linux

Programming language: Sun/Oracle Java 1.6

Other requirements: Sun/Oracle Java Runtime Environment Version 1.6 or higher

License: GNU General Public License

## Competing interests

The authors declare that they have no competing interests.

## Authors’ contributions

TL developed the idea, realized the program, tested the software and drafted the manuscript. MJL and AVH helped to shape the features of the application and revised and approved the manuscript.

## Supplementary Material

Additional file 1 MS Excel XLS table with Benchmark tree set results and results on non-rectangular trees. Click here for file

Additional file 2 ZIP files containing several folders, each of which with TreeSnatcher Plus snapshot files, the original image and a text file. Click here for file

Additional file 3 ZIP files containing several folders, each of which with TreeSnatcher Plus snapshot files, the original image and a text file. Click here for file

Additional file 4 ZIP files containing several folders, each of which with TreeSnatcher Plus snapshot files, the original image and a text file. Click here for file

Additional file 5 ZIP files containing several folders, each of which with TreeSnatcher Plus snapshot files, the original image and a text file. Click here for file

Additional file 6 ZIP files containing several folders, each of which with TreeSnatcher Plus snapshot files, the original image and a text file. Click here for file
