# Supplementary figures and images for: TreeSnatcher plus: capturing phylogenetic trees from images (part 1 of 5)
Source: BMC Bioinformatics. 2012 May 24;13:110. doi: 10.1186/1471-2105-13-110 (PMC3411374; doi:10.1186/1471-2105-13-110)

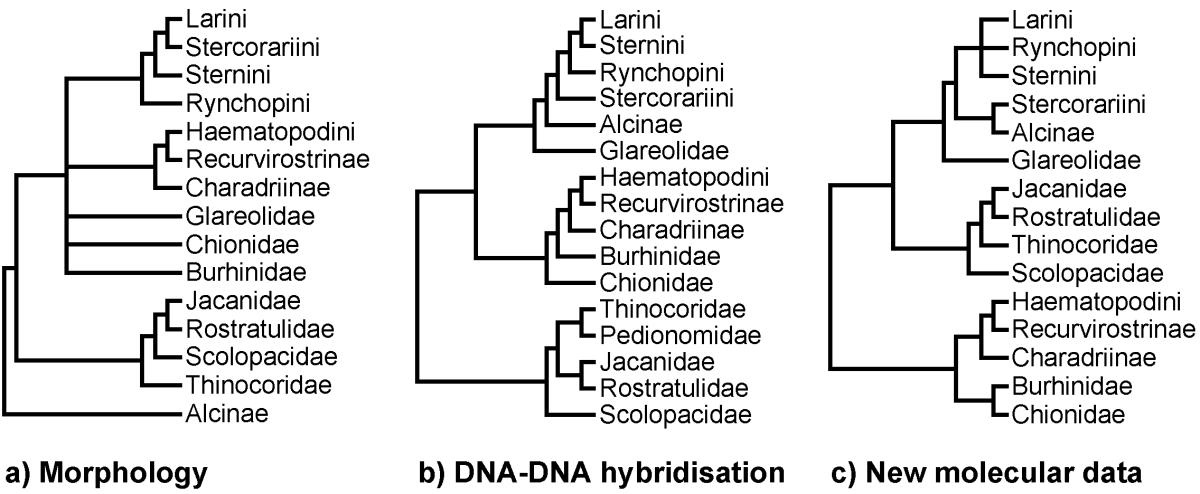

Supplement: Additional file 2 — ZIP files containing several folders, each of which with TreeSnatcher Plus snapshot files, the original image and a text file. [file 1471-2105-13-110-S2.zip › 1471-2148-4-28-1/1471-2148-4-28-1-l.jpg]

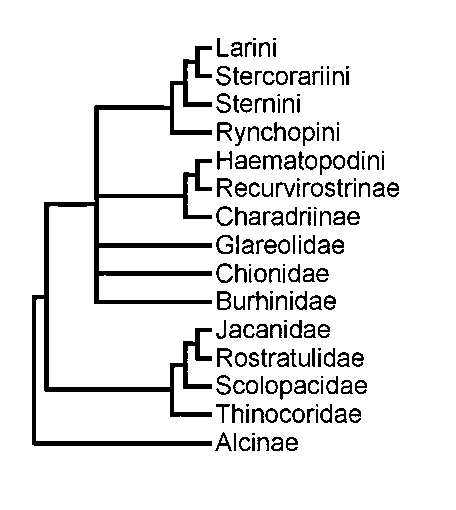

Supplement: Additional file 2 — ZIP files containing several folders, each of which with TreeSnatcher Plus snapshot files, the original image and a text file. [file 1471-2105-13-110-S2.zip › 1471-2148-4-28-1/1471-2148-4-28-1-l_b.PNG]

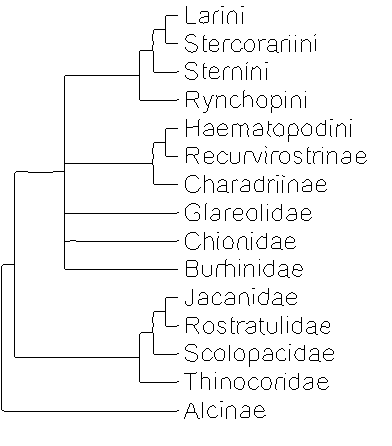

Supplement: Additional file 2 — ZIP files containing several folders, each of which with TreeSnatcher Plus snapshot files, the original image and a text file. [file 1471-2105-13-110-S2.zip › 1471-2148-4-28-1/1471-2148-4-28-1-l_c.PNG]

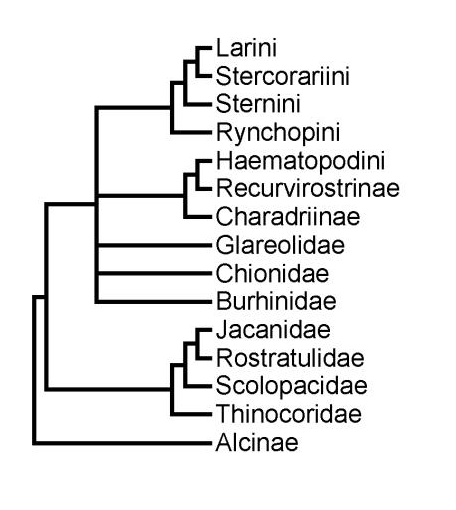

Supplement: Additional file 2 — ZIP files containing several folders, each of which with TreeSnatcher Plus snapshot files, the original image and a text file. [file 1471-2105-13-110-S2.zip › 1471-2148-4-28-1/1471-2148-4-28-1-l_o.PNG]

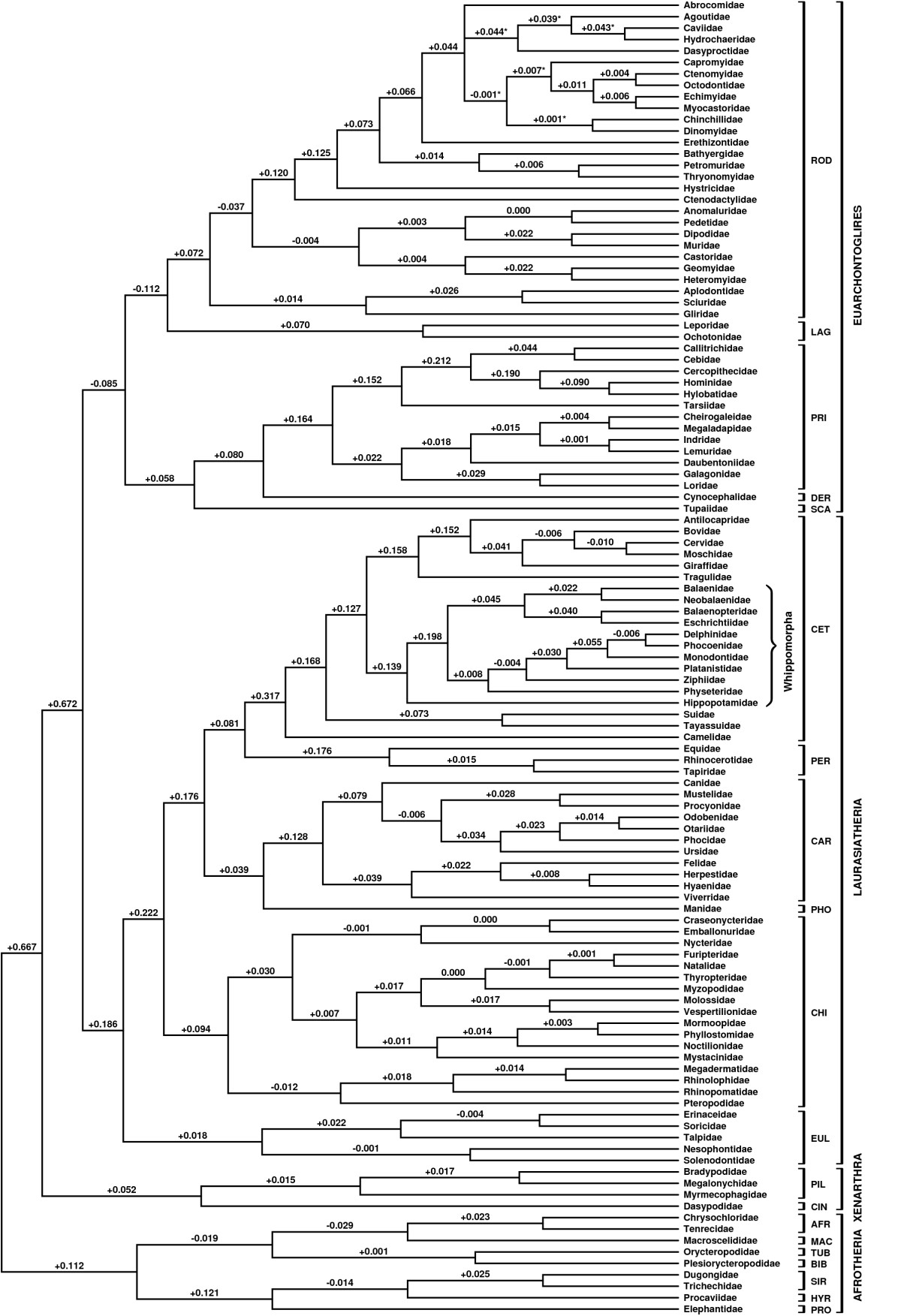

Supplement: Additional file 2 — ZIP files containing several folders, each of which with TreeSnatcher Plus snapshot files, the original image and a text file. [file 1471-2105-13-110-S2.zip › 1471-2148-6-93-1/1471-2148-6-93-1-l.jpg]

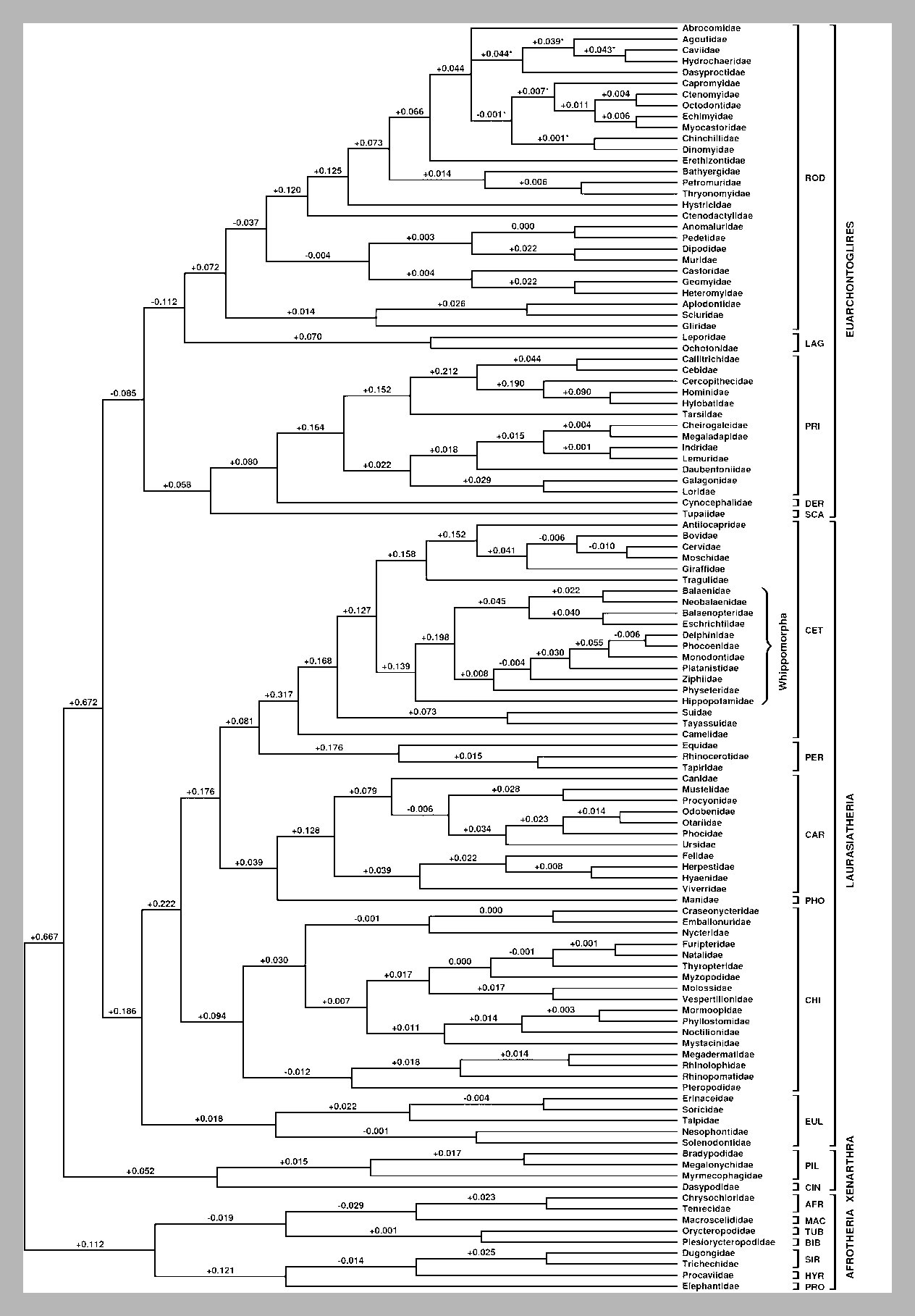

Supplement: Additional file 2 — ZIP files containing several folders, each of which with TreeSnatcher Plus snapshot files, the original image and a text file. [file 1471-2105-13-110-S2.zip › 1471-2148-6-93-1/1471-2148-6-93-1-l_b.PNG]

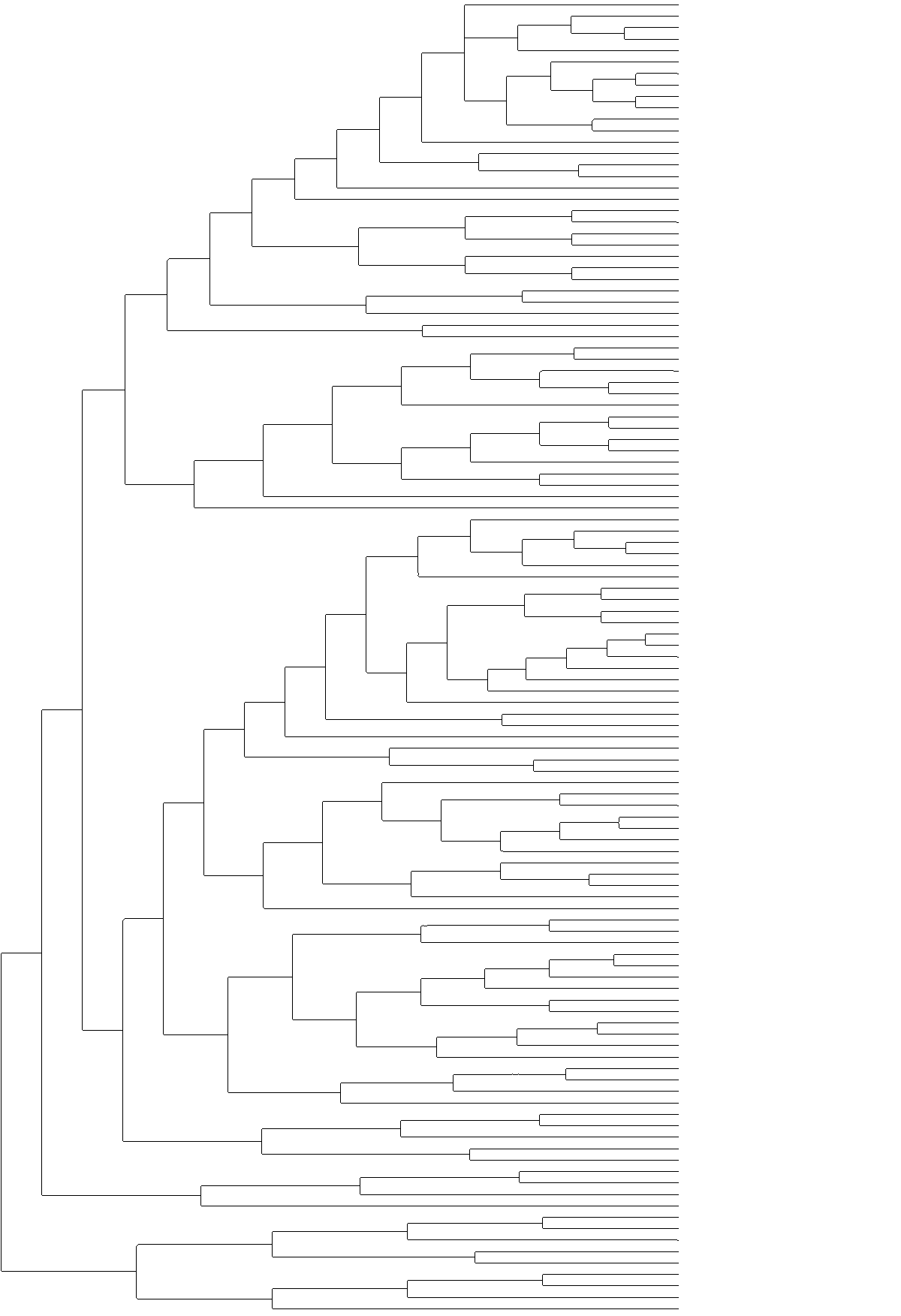

Supplement: Additional file 2 — ZIP files containing several folders, each of which with TreeSnatcher Plus snapshot files, the original image and a text file. [file 1471-2105-13-110-S2.zip › 1471-2148-6-93-1/1471-2148-6-93-1-l_c.PNG]

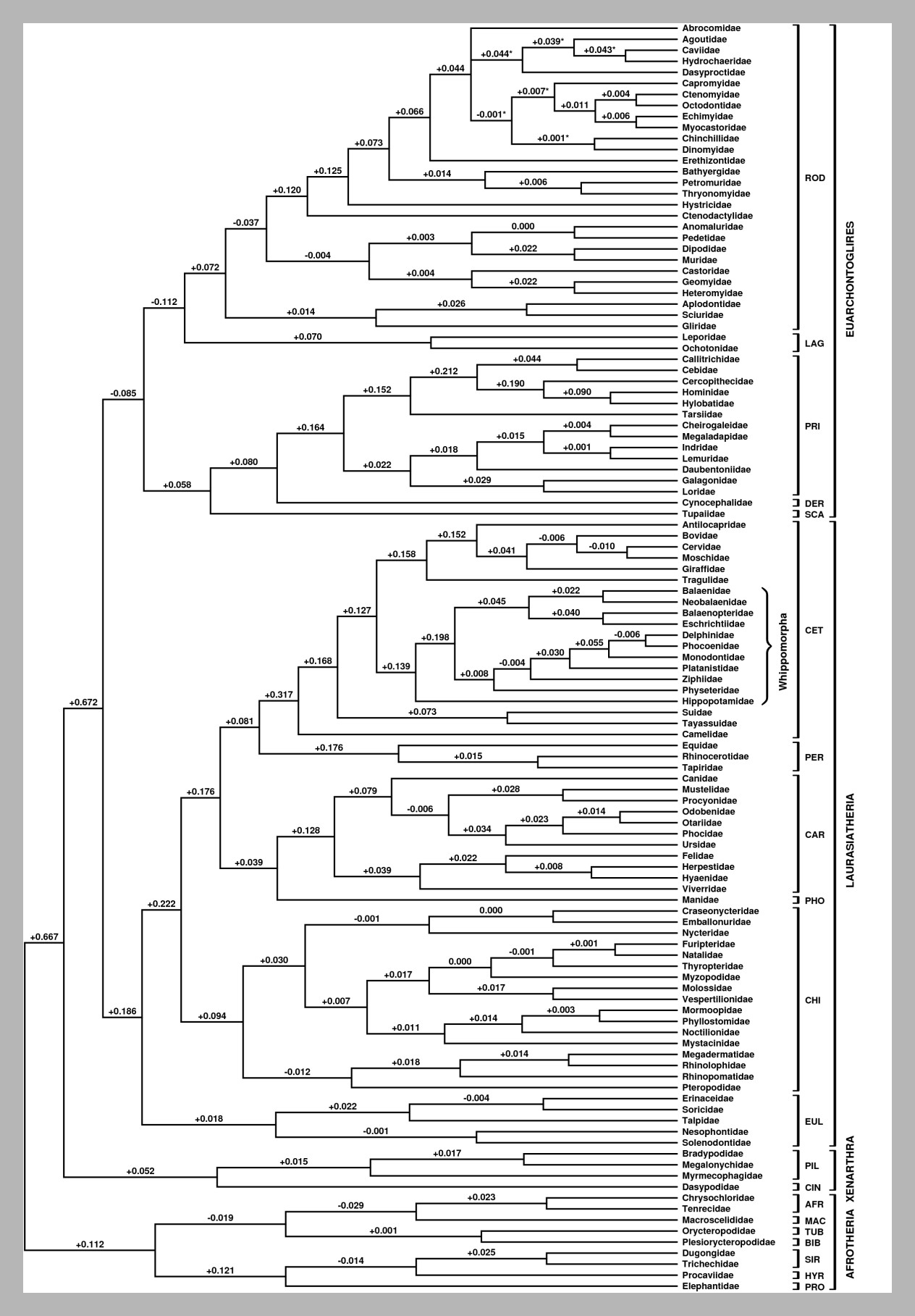

Supplement: Additional file 2 — ZIP files containing several folders, each of which with TreeSnatcher Plus snapshot files, the original image and a text file. [file 1471-2105-13-110-S2.zip › 1471-2148-6-93-1/1471-2148-6-93-1-l_o.PNG]

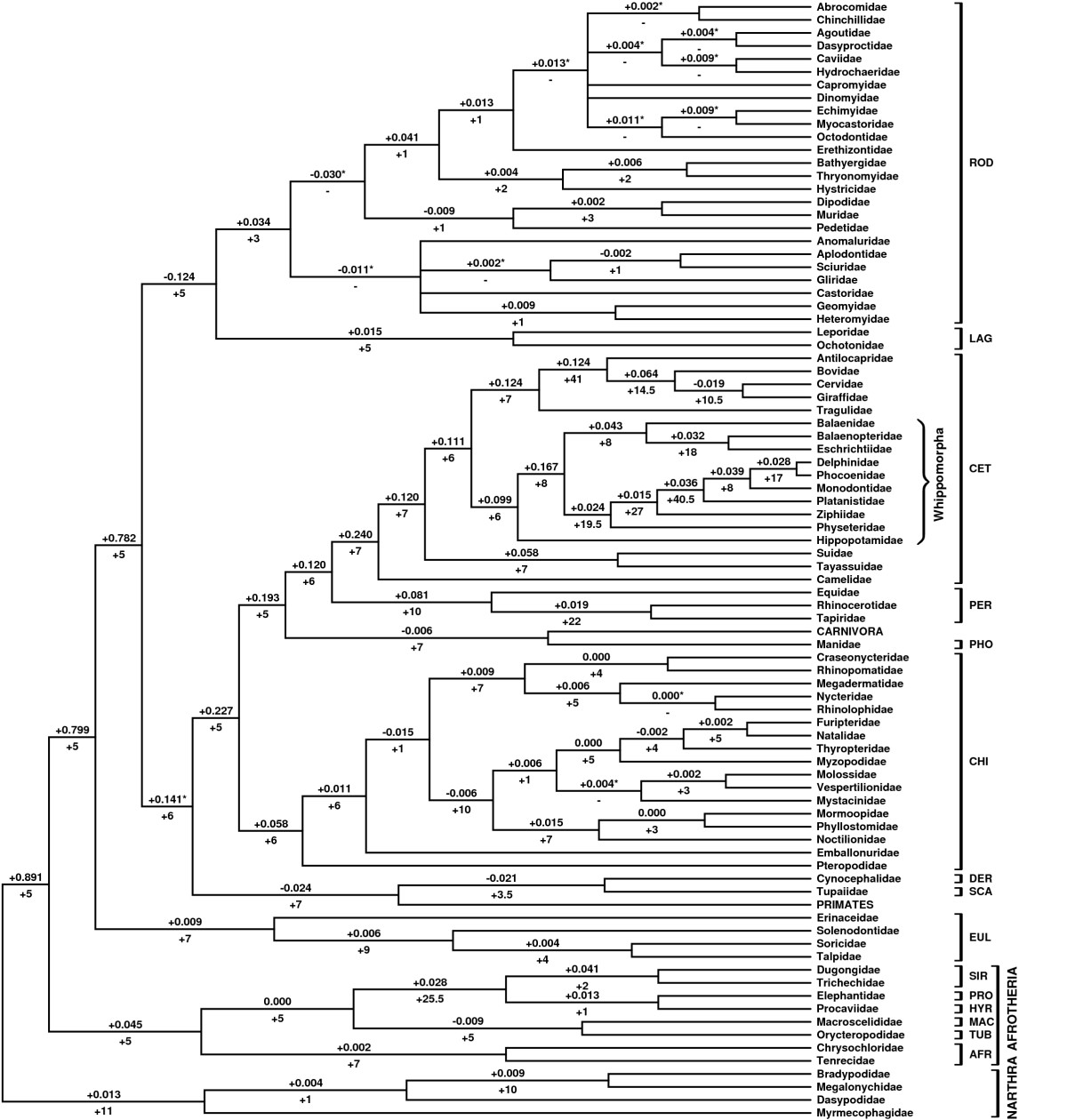

Supplement: Additional file 2 — ZIP files containing several folders, each of which with TreeSnatcher Plus snapshot files, the original image and a text file. [file 1471-2105-13-110-S2.zip › 1471-2148-6-93-2/1471-2148-6-93-2-l.jpg]

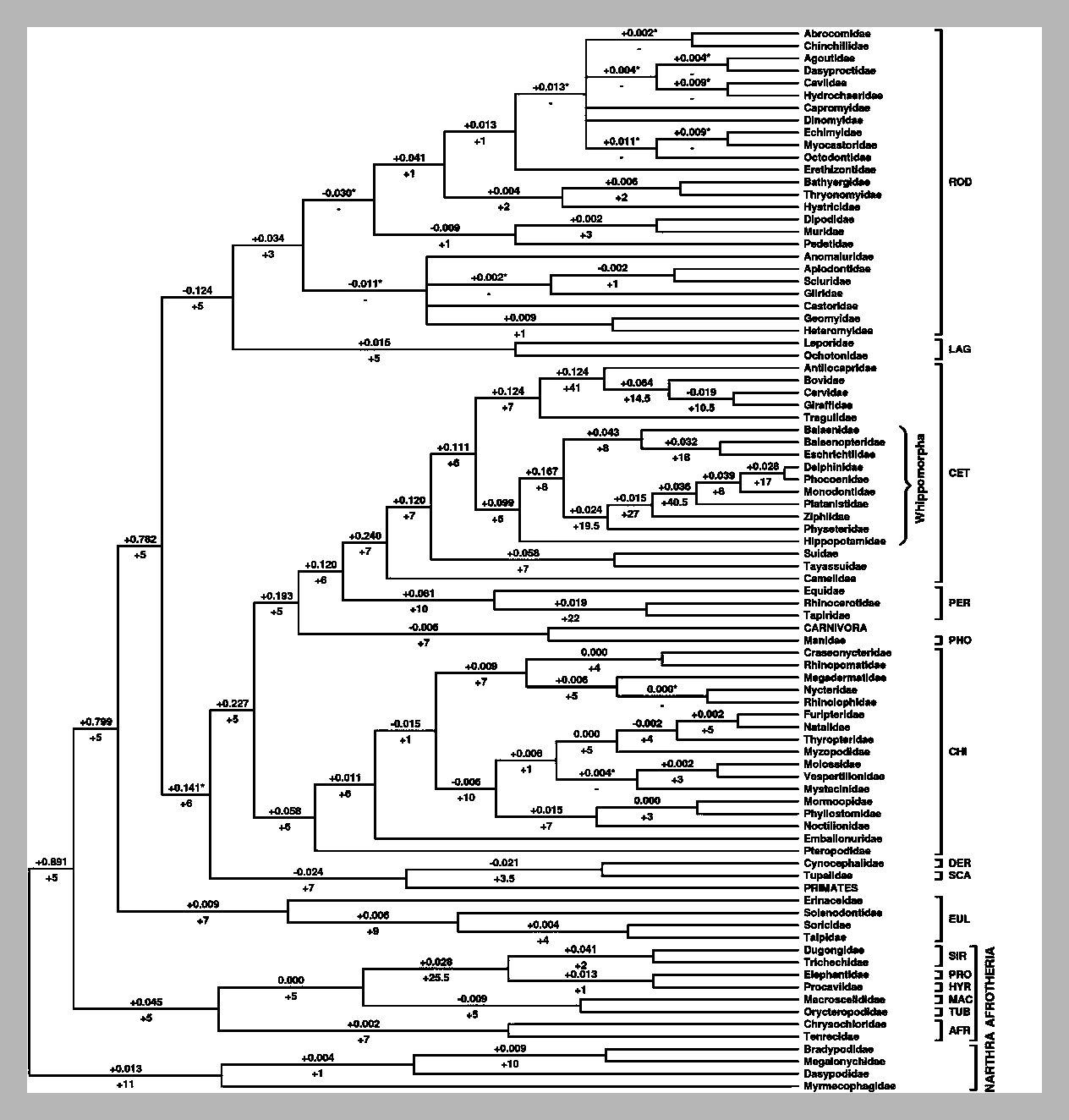

Supplement: Additional file 2 — ZIP files containing several folders, each of which with TreeSnatcher Plus snapshot files, the original image and a text file. [file 1471-2105-13-110-S2.zip › 1471-2148-6-93-2/1471-2148-6-93-2-l_b.PNG]

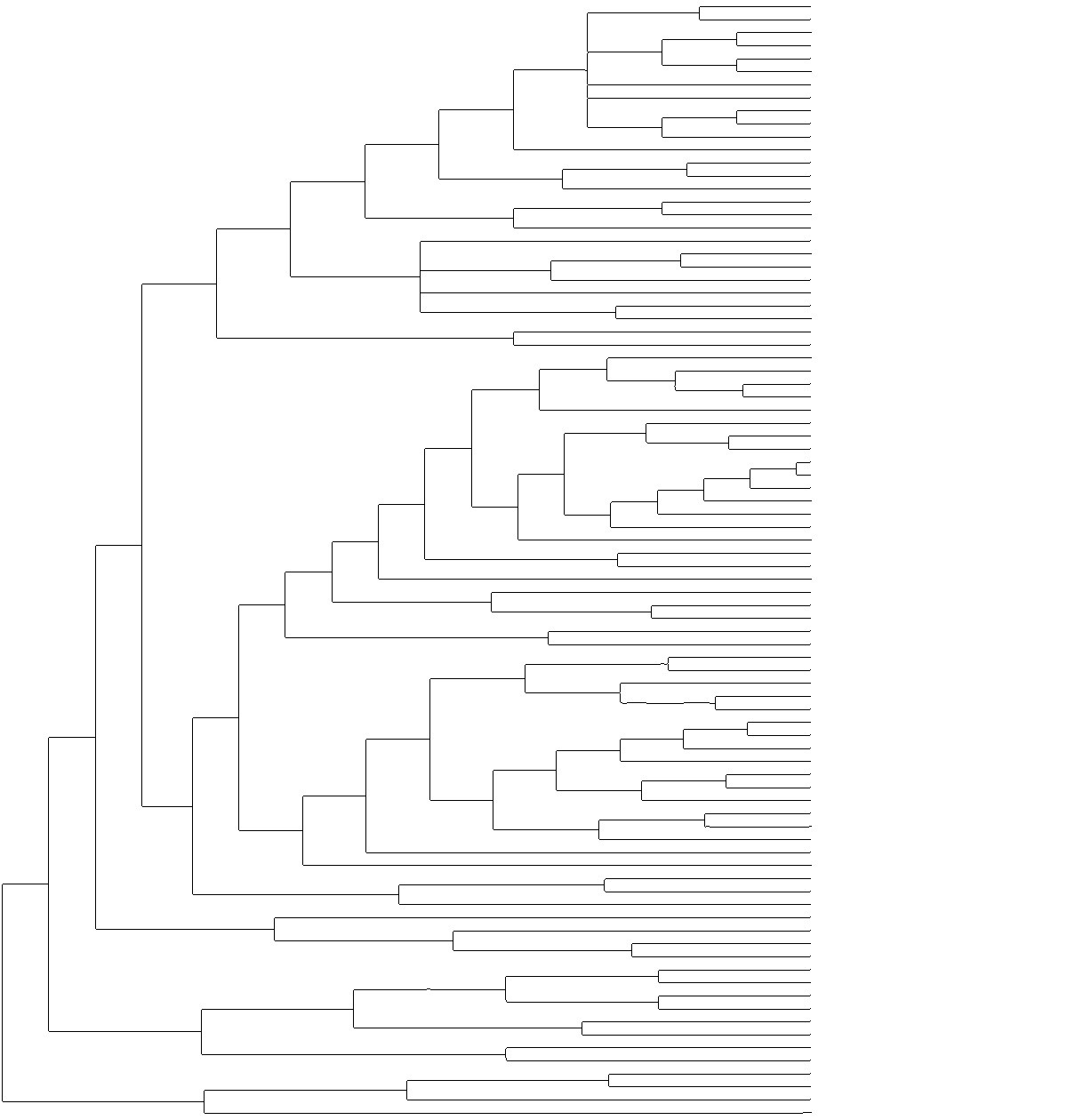

Supplement: Additional file 2 — ZIP files containing several folders, each of which with TreeSnatcher Plus snapshot files, the original image and a text file. [file 1471-2105-13-110-S2.zip › 1471-2148-6-93-2/1471-2148-6-93-2-l_c.PNG]

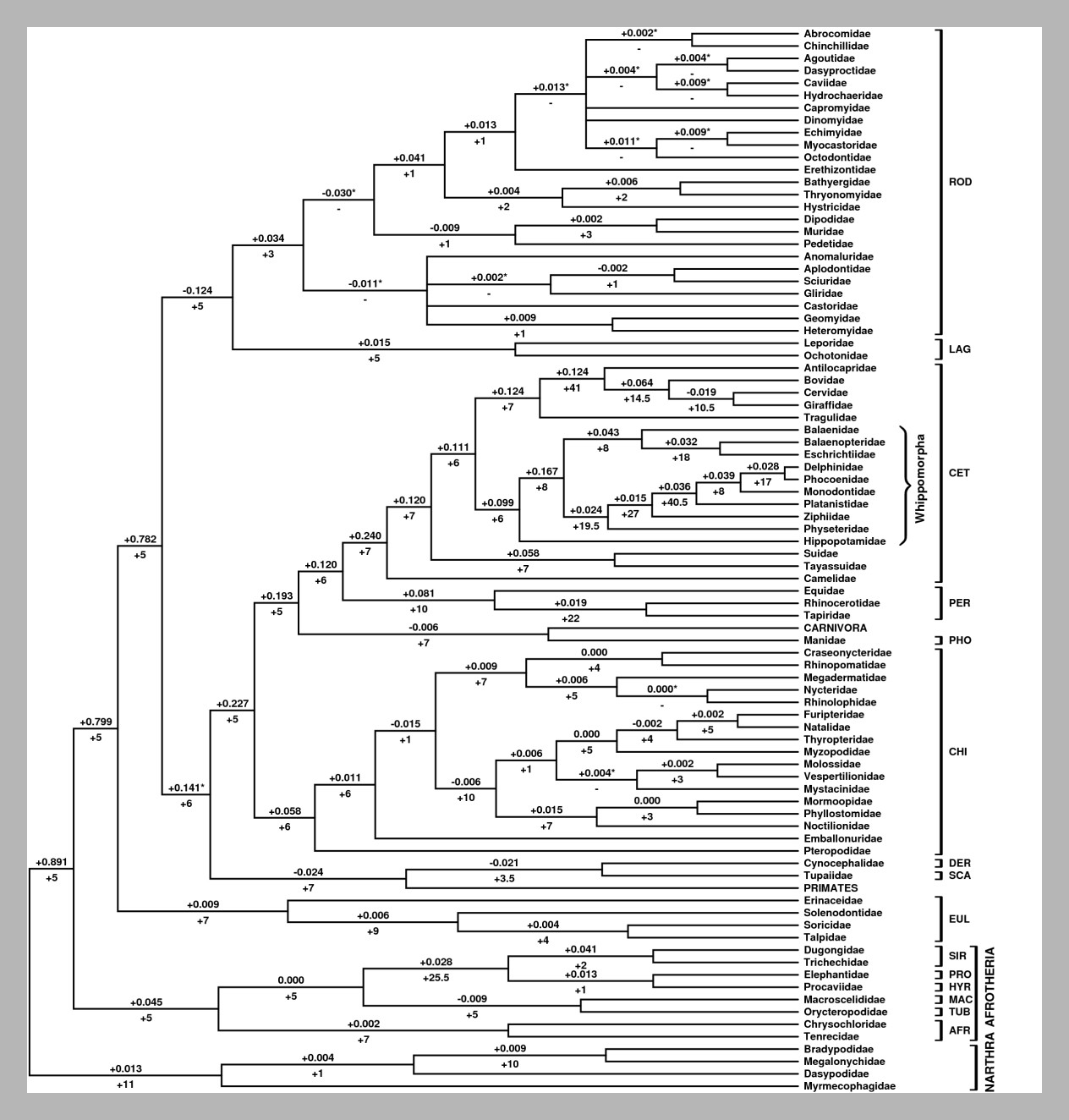

Supplement: Additional file 2 — ZIP files containing several folders, each of which with TreeSnatcher Plus snapshot files, the original image and a text file. [file 1471-2105-13-110-S2.zip › 1471-2148-6-93-2/1471-2148-6-93-2-l_o.PNG]

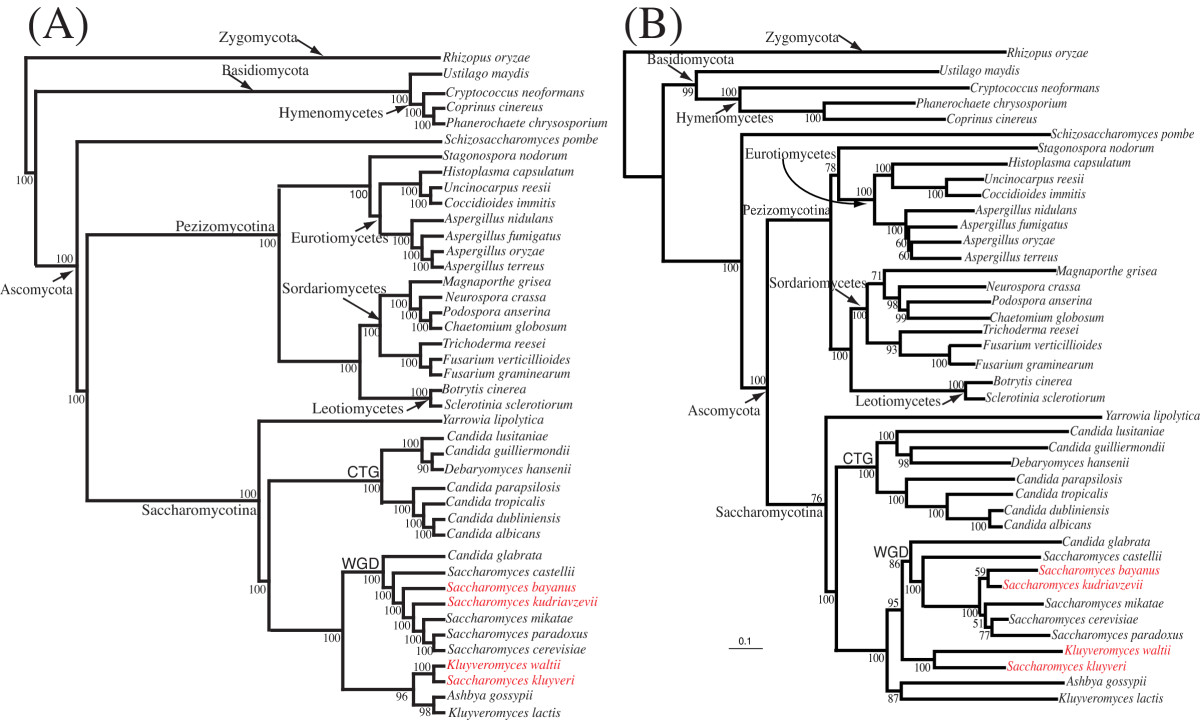

Supplement: Additional file 2 — ZIP files containing several folders, each of which with TreeSnatcher Plus snapshot files, the original image and a text file. [file 1471-2105-13-110-S2.zip › 1471-2148-6-99-1/1471-2148-6-99-1-l.jpg]

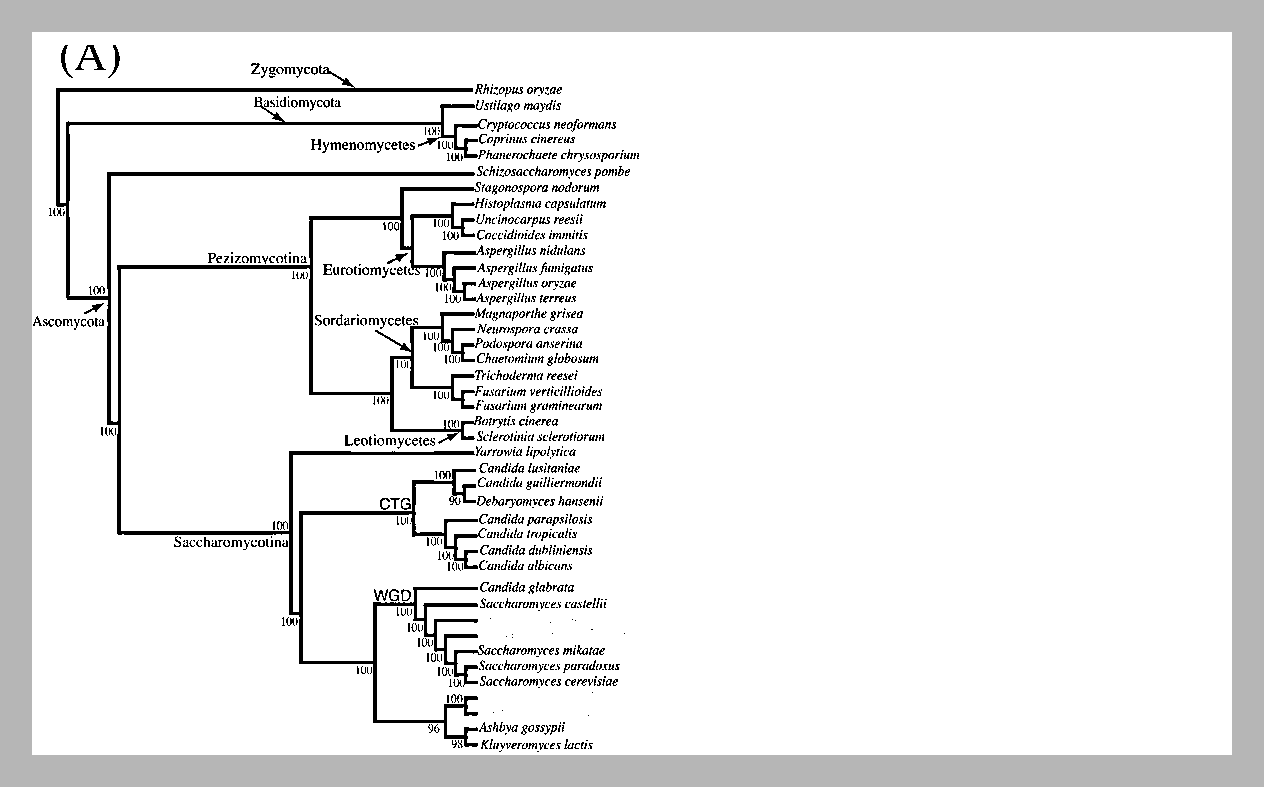

Supplement: Additional file 2 — ZIP files containing several folders, each of which with TreeSnatcher Plus snapshot files, the original image and a text file. [file 1471-2105-13-110-S2.zip › 1471-2148-6-99-1/1471-2148-6-99-1-l_b.PNG]

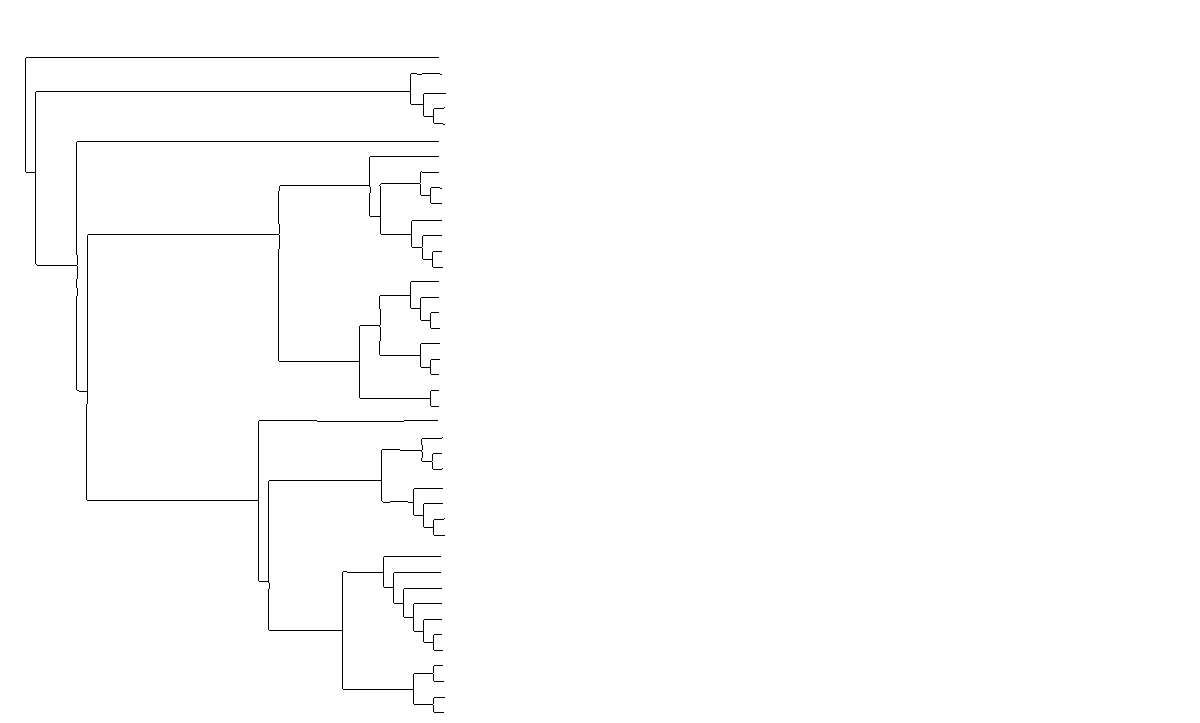

Supplement: Additional file 2 — ZIP files containing several folders, each of which with TreeSnatcher Plus snapshot files, the original image and a text file. [file 1471-2105-13-110-S2.zip › 1471-2148-6-99-1/1471-2148-6-99-1-l_c.PNG]

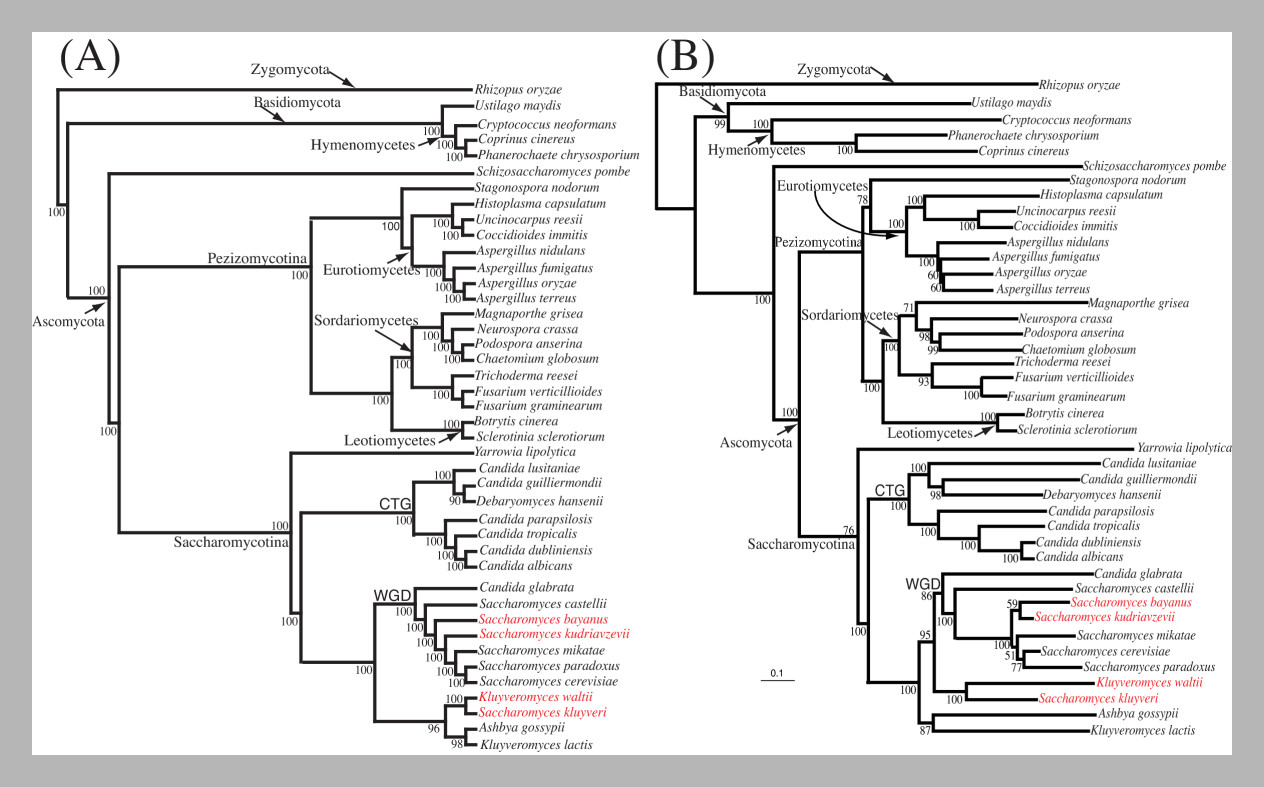

Supplement: Additional file 2 — ZIP files containing several folders, each of which with TreeSnatcher Plus snapshot files, the original image and a text file. [file 1471-2105-13-110-S2.zip › 1471-2148-6-99-1/1471-2148-6-99-1-l_o.PNG]

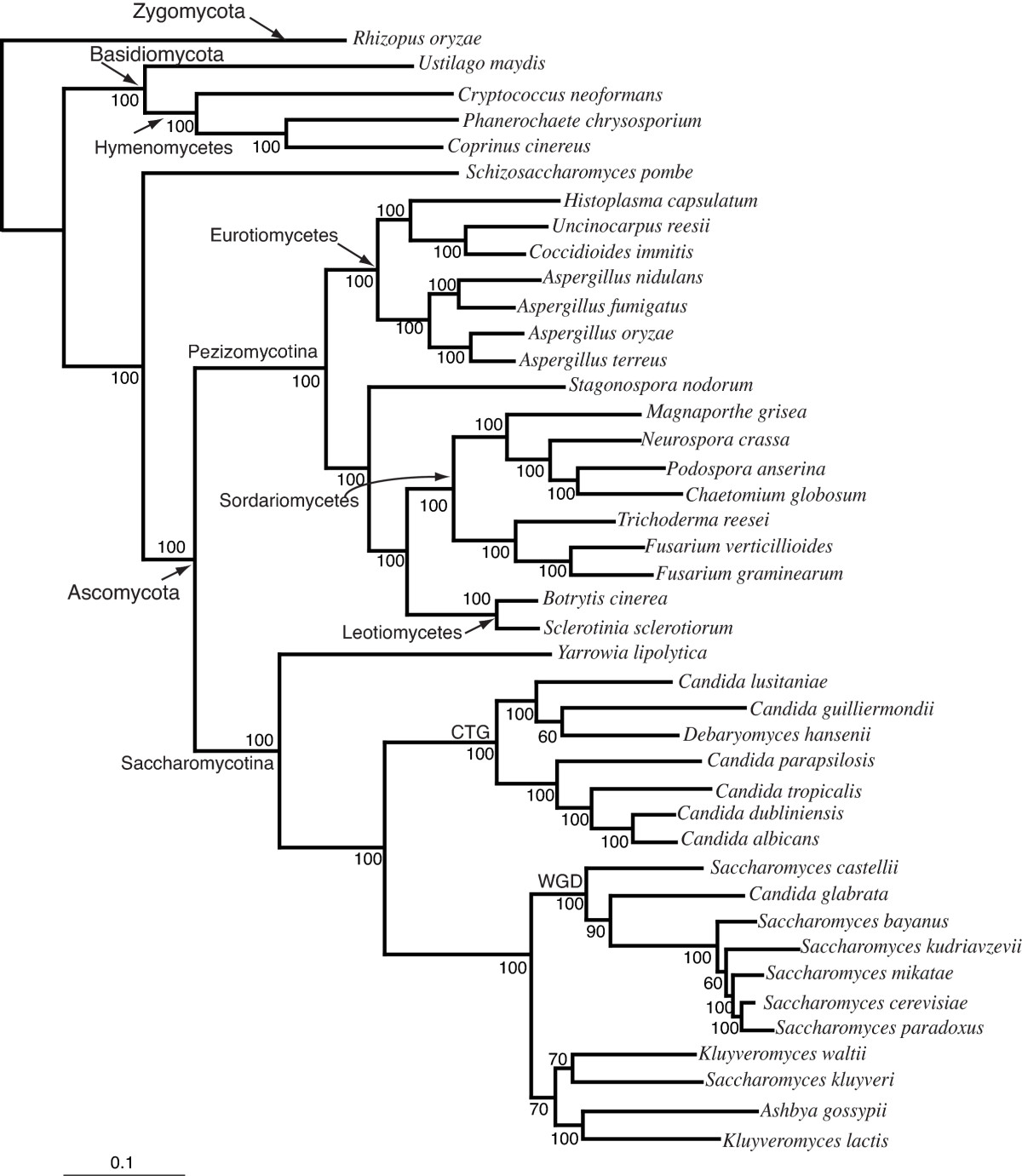

Supplement: Additional file 2 — ZIP files containing several folders, each of which with TreeSnatcher Plus snapshot files, the original image and a text file. [file 1471-2105-13-110-S2.zip › 1471-2148-6-99-2/1471-2148-6-99-2-l.jpg]

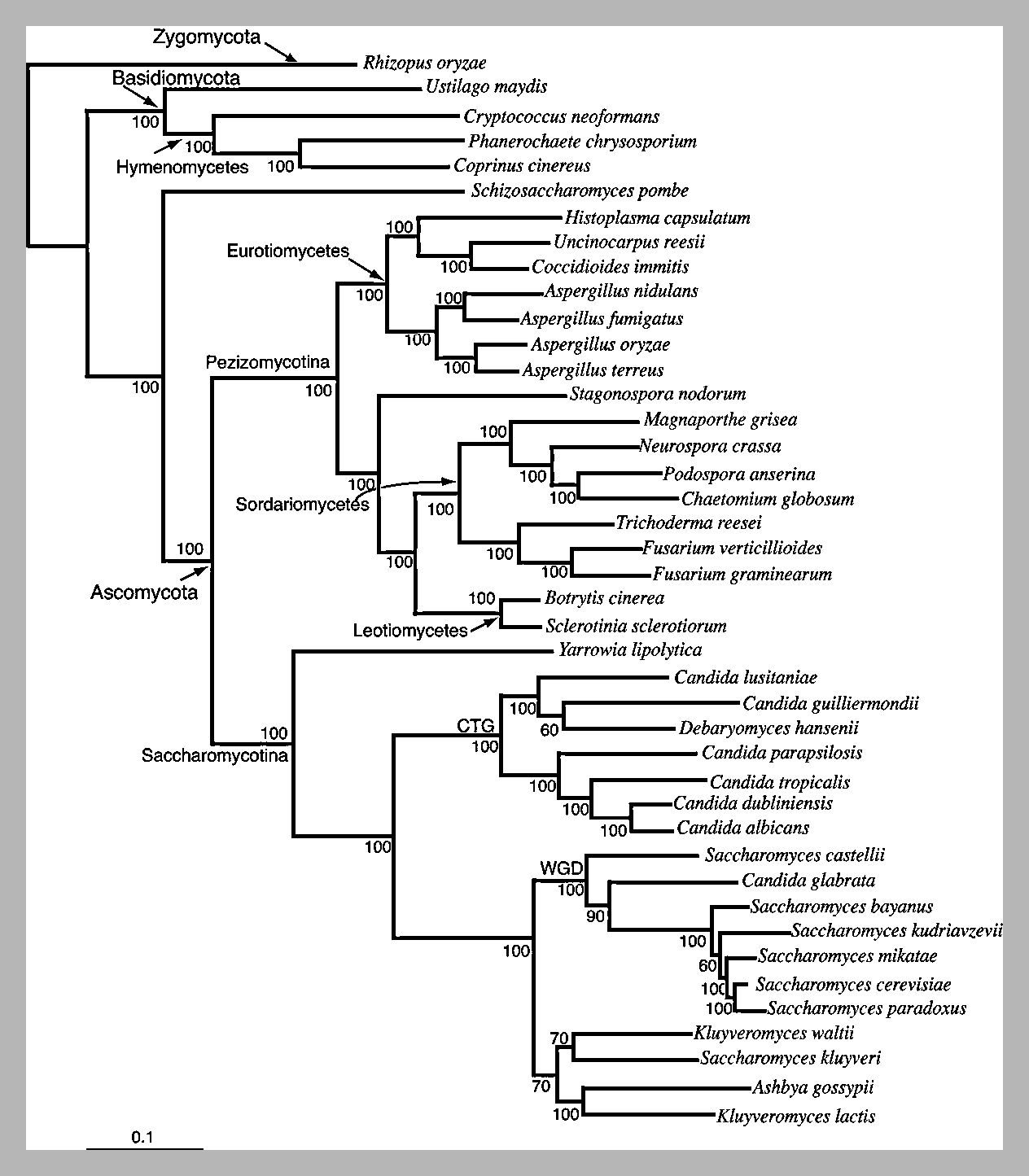

Supplement: Additional file 2 — ZIP files containing several folders, each of which with TreeSnatcher Plus snapshot files, the original image and a text file. [file 1471-2105-13-110-S2.zip › 1471-2148-6-99-2/1471-2148-6-99-2-l_b.PNG]

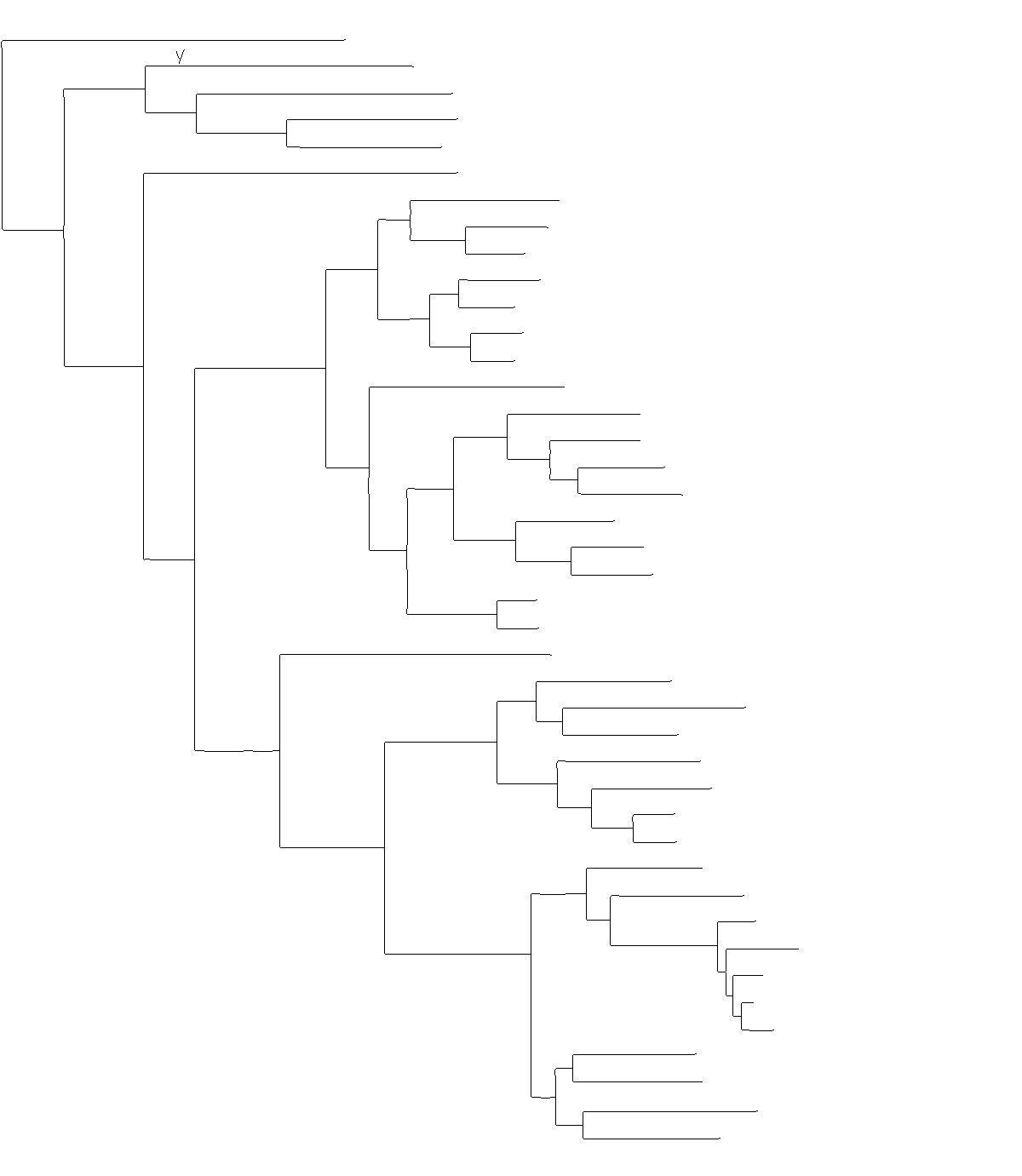

Supplement: Additional file 2 — ZIP files containing several folders, each of which with TreeSnatcher Plus snapshot files, the original image and a text file. [file 1471-2105-13-110-S2.zip › 1471-2148-6-99-2/1471-2148-6-99-2-l_c.PNG]

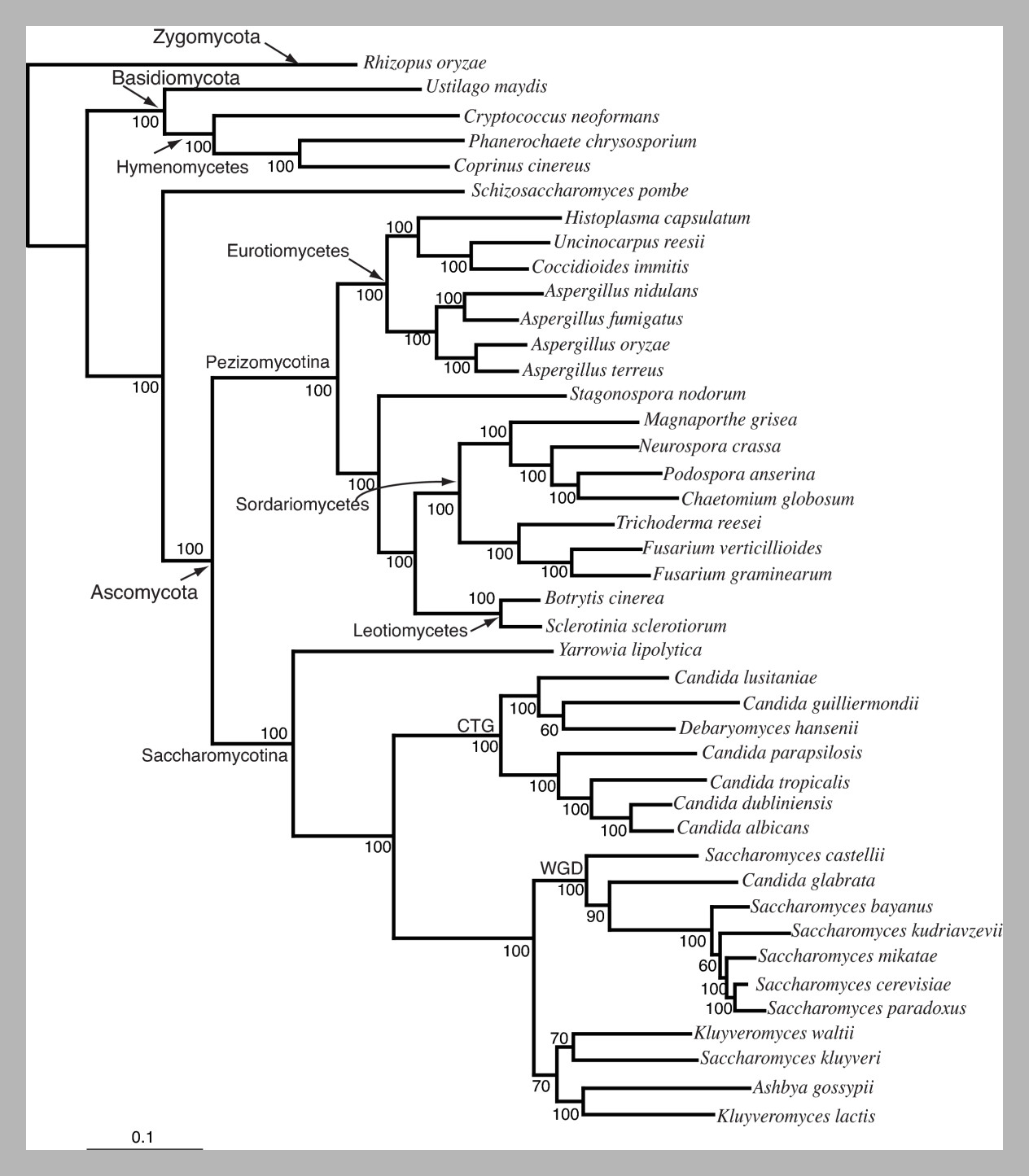

Supplement: Additional file 2 — ZIP files containing several folders, each of which with TreeSnatcher Plus snapshot files, the original image and a text file. [file 1471-2105-13-110-S2.zip › 1471-2148-6-99-2/1471-2148-6-99-2-l_o.PNG]

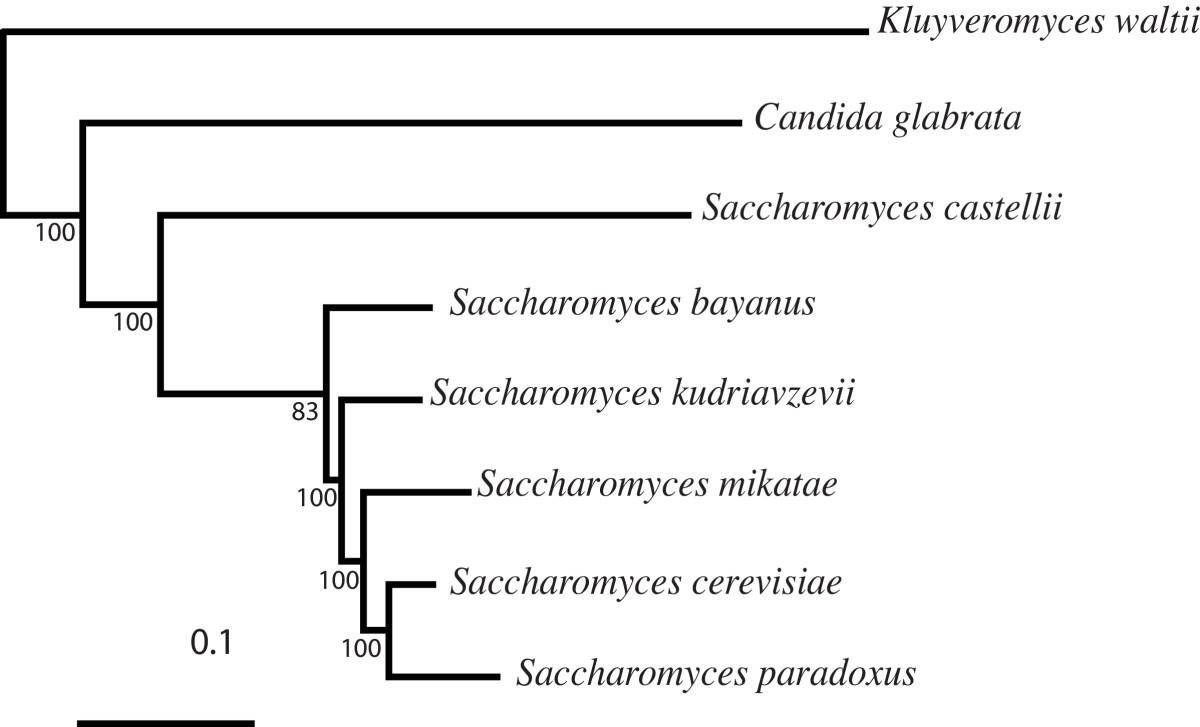

Supplement: Additional file 2 — ZIP files containing several folders, each of which with TreeSnatcher Plus snapshot files, the original image and a text file. [file 1471-2105-13-110-S2.zip › 1471-2148-6-99-3/1471-2148-6-99-3-l.jpg]

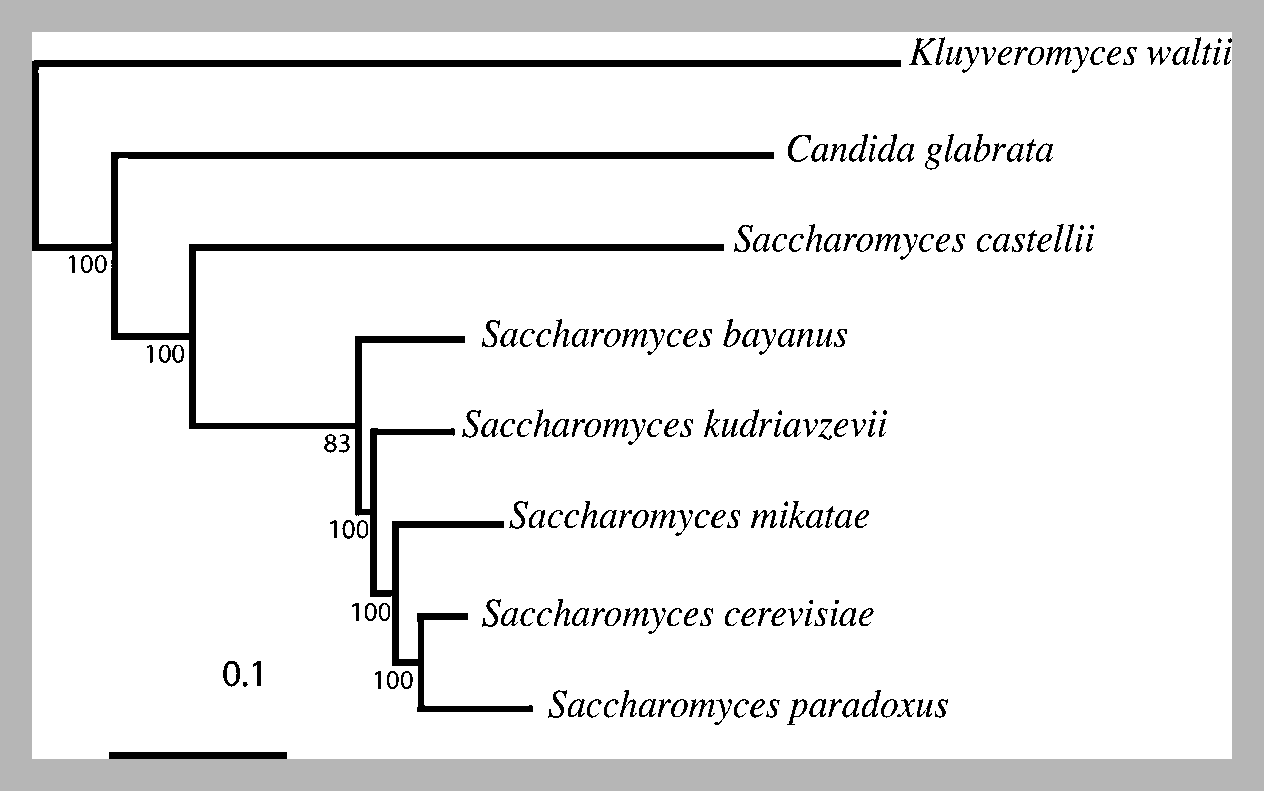

Supplement: Additional file 2 — ZIP files containing several folders, each of which with TreeSnatcher Plus snapshot files, the original image and a text file. [file 1471-2105-13-110-S2.zip › 1471-2148-6-99-3/1471-2148-6-99-3-l_b.PNG]

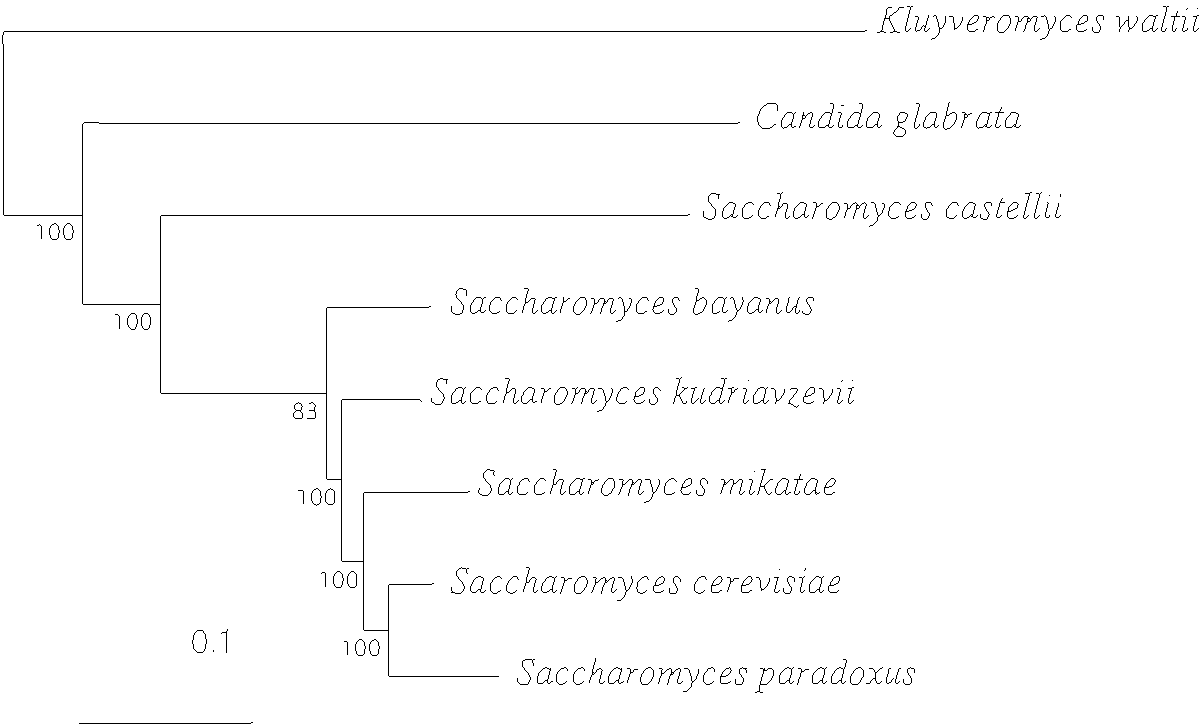

Supplement: Additional file 2 — ZIP files containing several folders, each of which with TreeSnatcher Plus snapshot files, the original image and a text file. [file 1471-2105-13-110-S2.zip › 1471-2148-6-99-3/1471-2148-6-99-3-l_c.PNG]

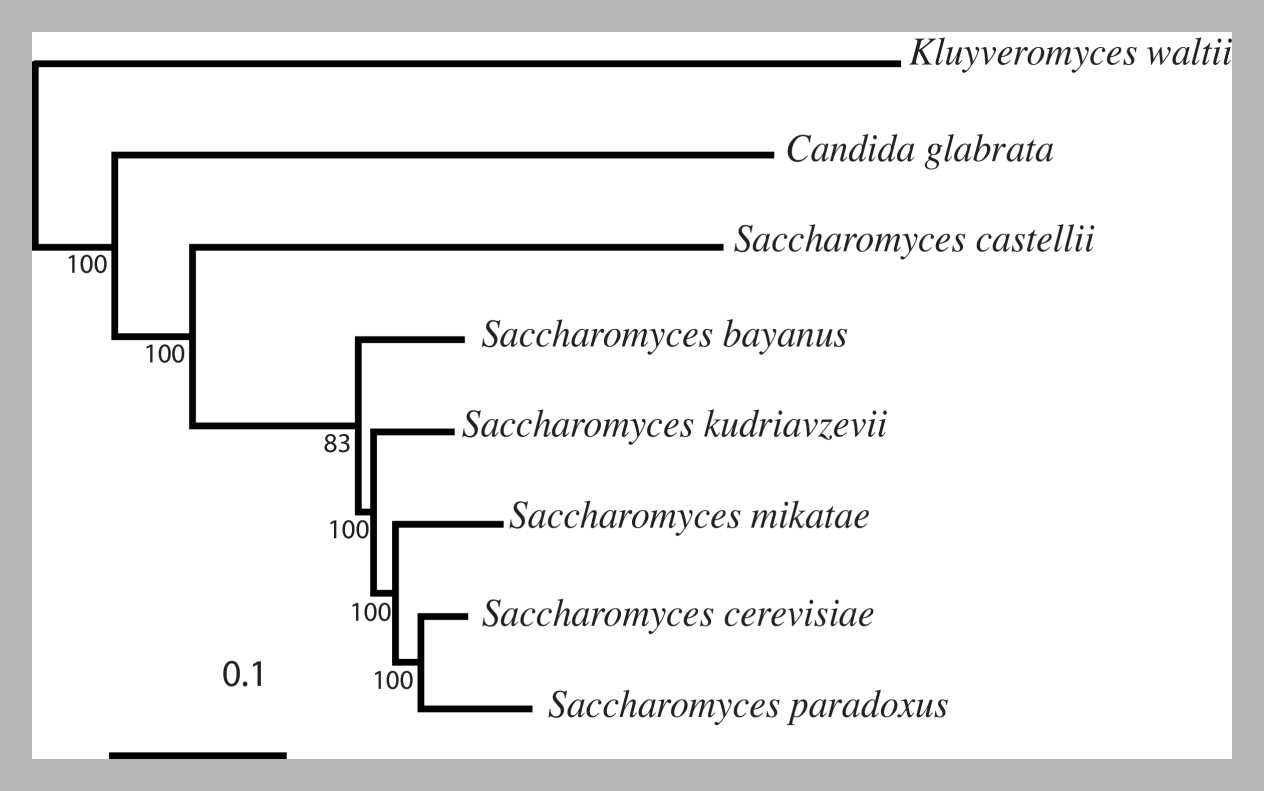

Supplement: Additional file 2 — ZIP files containing several folders, each of which with TreeSnatcher Plus snapshot files, the original image and a text file. [file 1471-2105-13-110-S2.zip › 1471-2148-6-99-3/1471-2148-6-99-3-l_o.PNG]

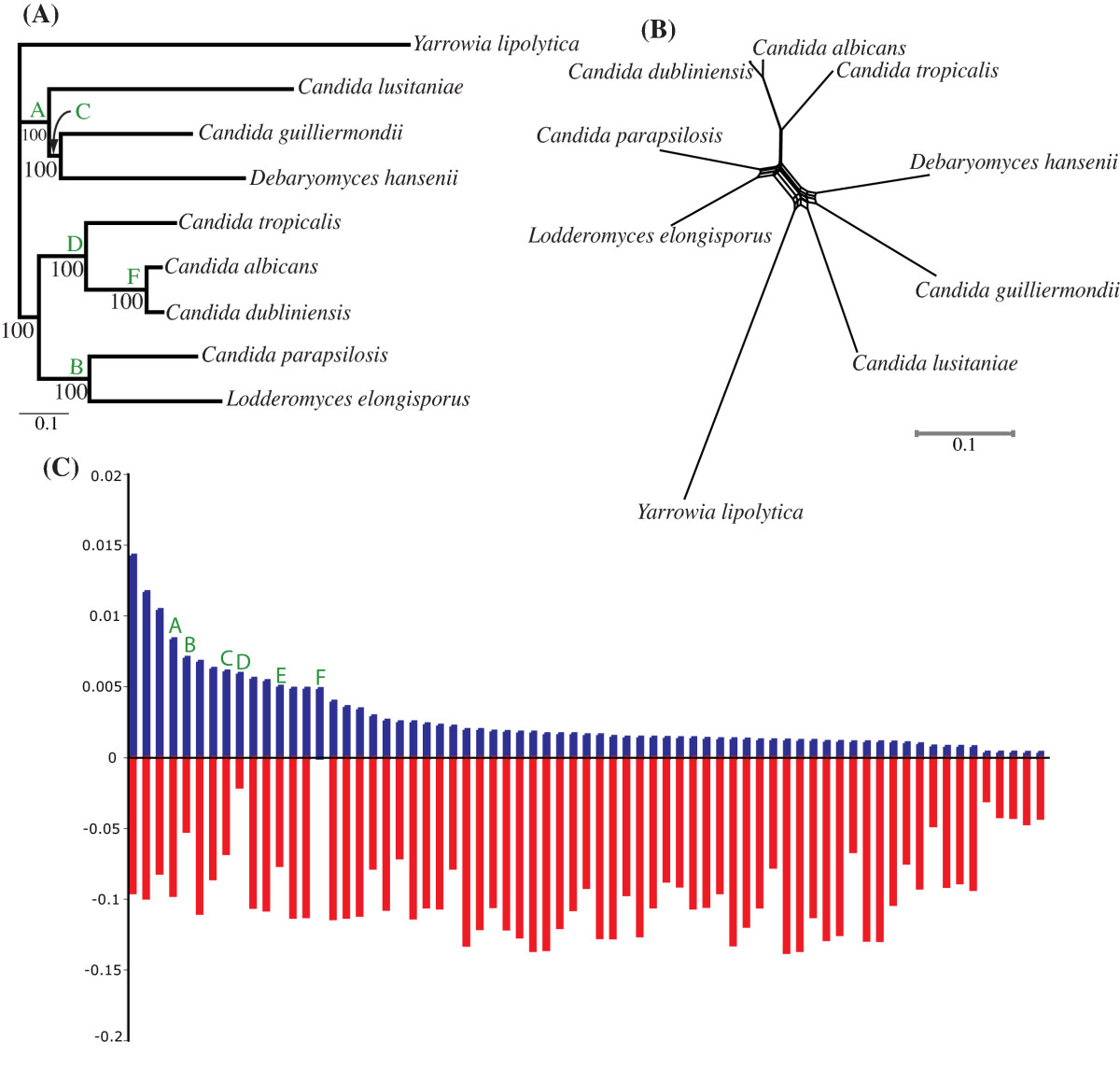

Supplement: Additional file 2 — ZIP files containing several folders, each of which with TreeSnatcher Plus snapshot files, the original image and a text file. [file 1471-2105-13-110-S2.zip › 1471-2148-6-99-5/1471-2148-6-99-5-l.jpg]

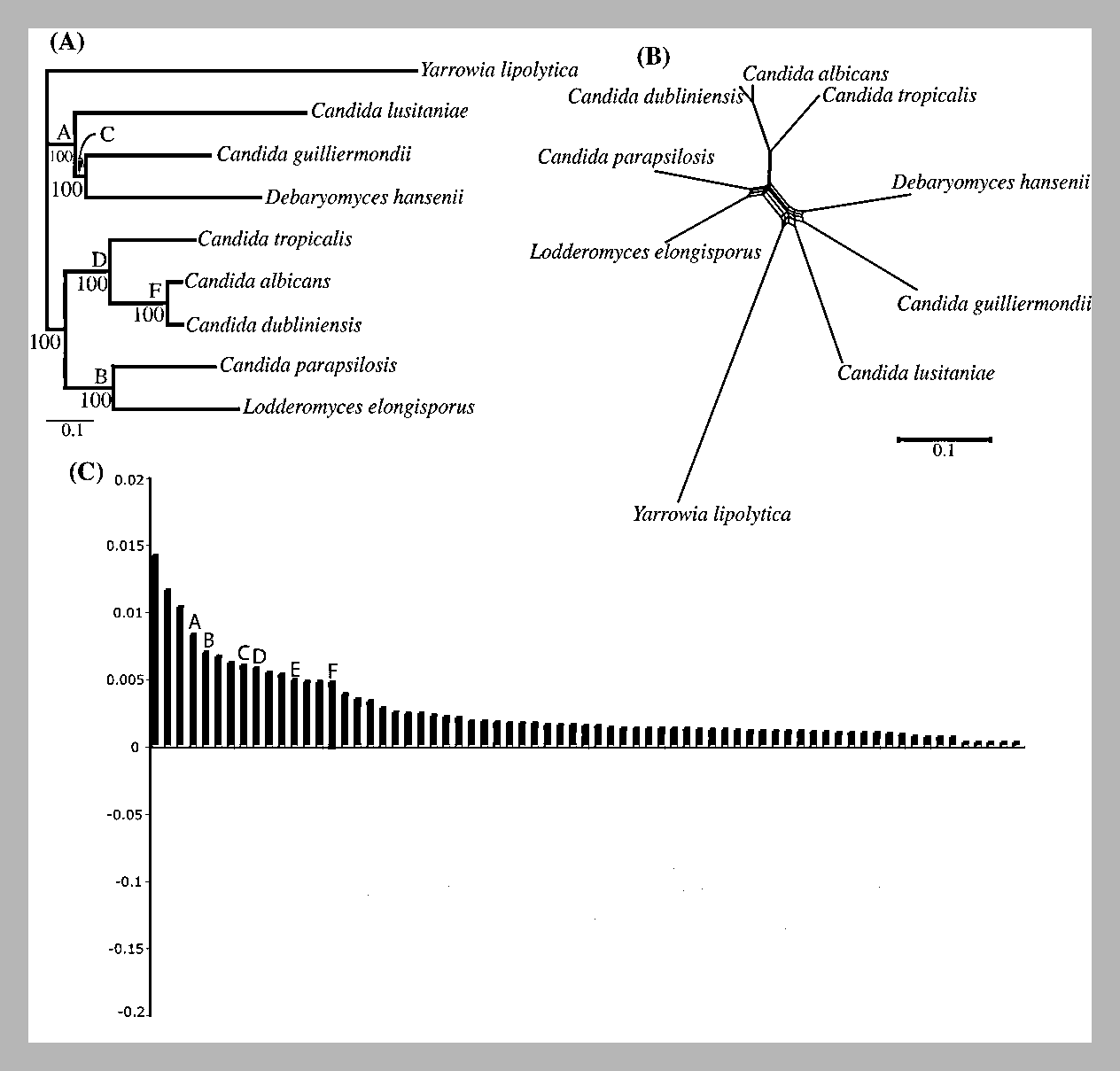

Supplement: Additional file 2 — ZIP files containing several folders, each of which with TreeSnatcher Plus snapshot files, the original image and a text file. [file 1471-2105-13-110-S2.zip › 1471-2148-6-99-5/1471-2148-6-99-5-l_b.PNG]

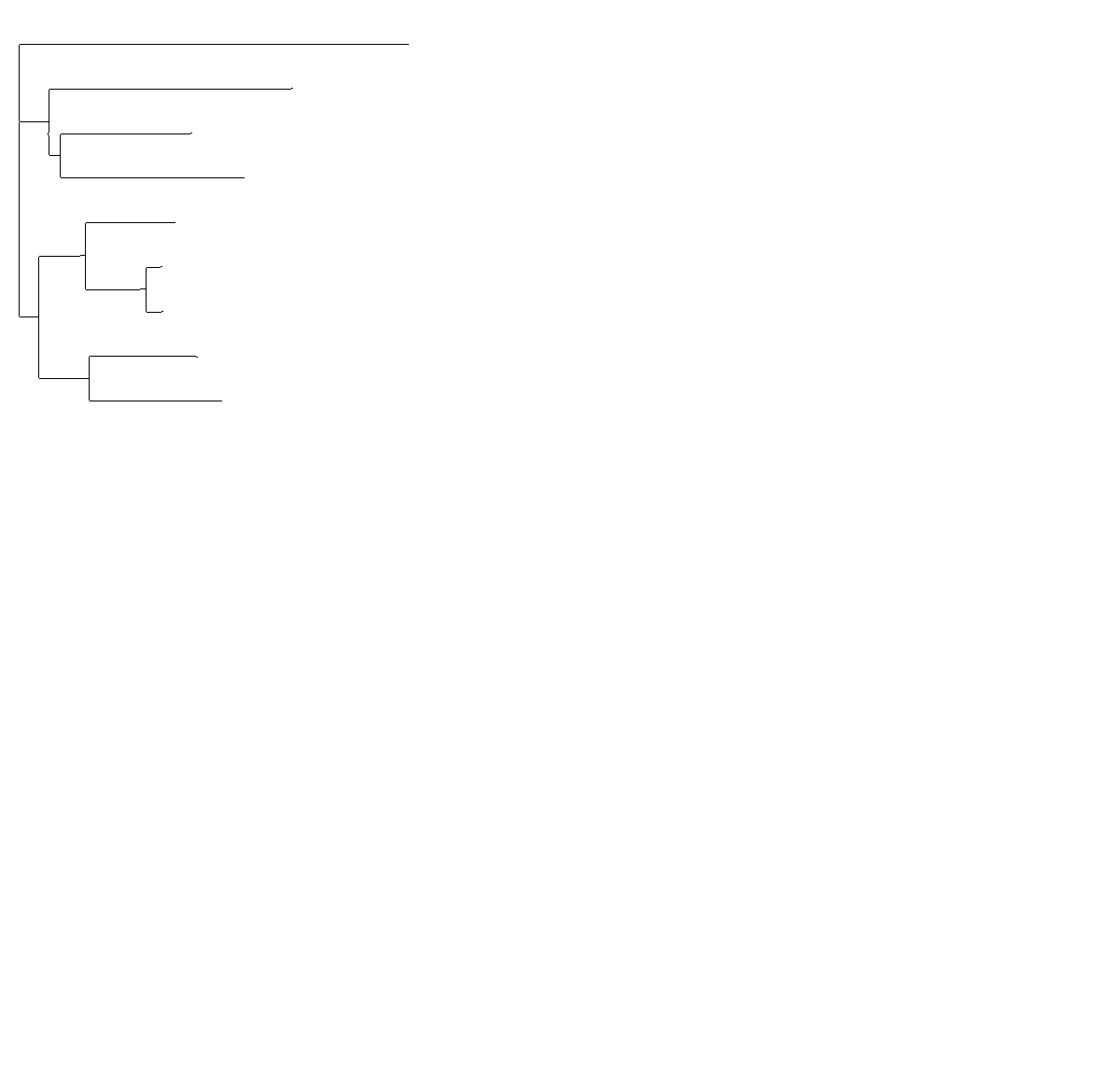

Supplement: Additional file 2 — ZIP files containing several folders, each of which with TreeSnatcher Plus snapshot files, the original image and a text file. [file 1471-2105-13-110-S2.zip › 1471-2148-6-99-5/1471-2148-6-99-5-l_c.PNG]

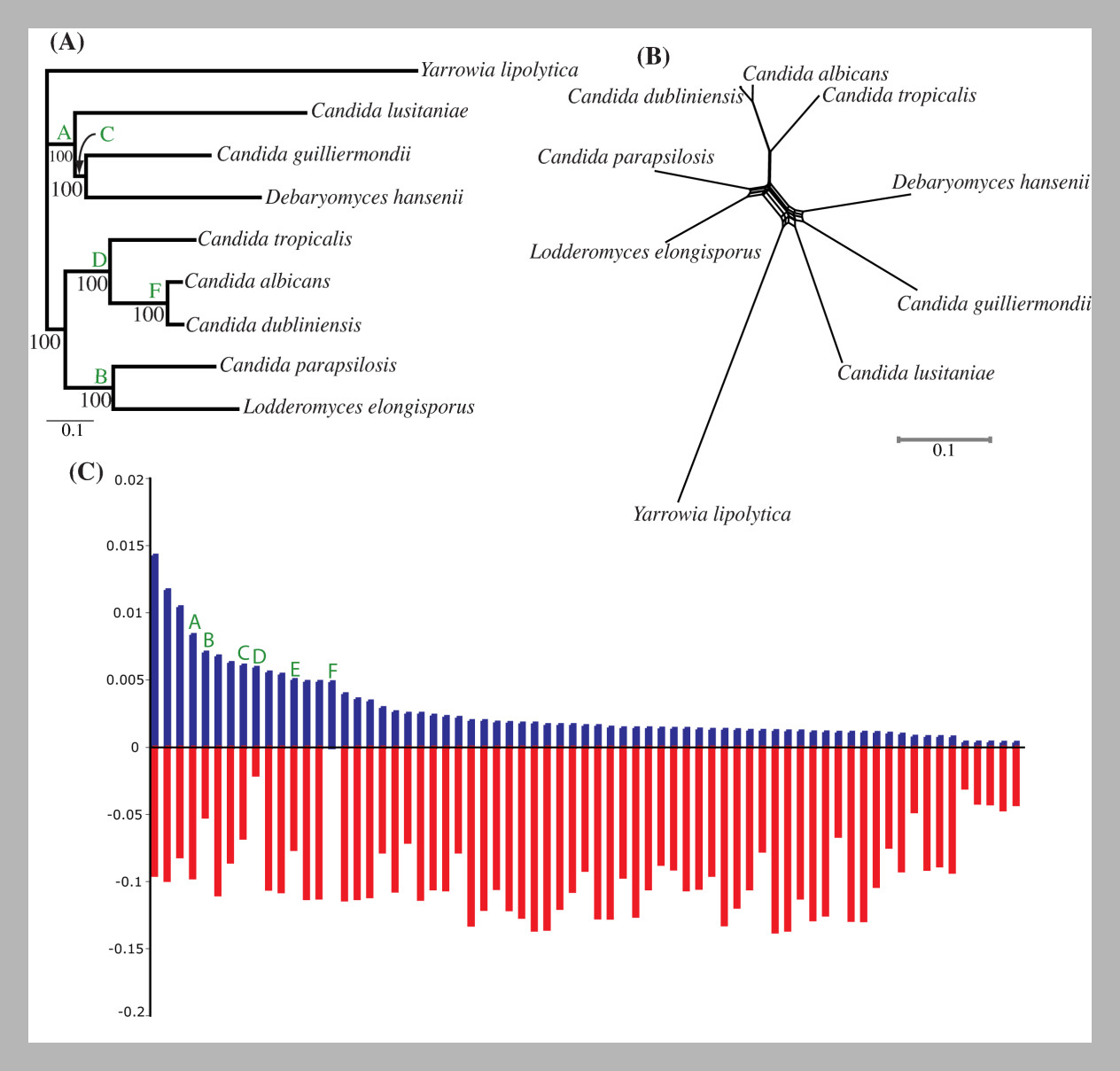

Supplement: Additional file 2 — ZIP files containing several folders, each of which with TreeSnatcher Plus snapshot files, the original image and a text file. [file 1471-2105-13-110-S2.zip › 1471-2148-6-99-5/1471-2148-6-99-5-l_o.PNG]

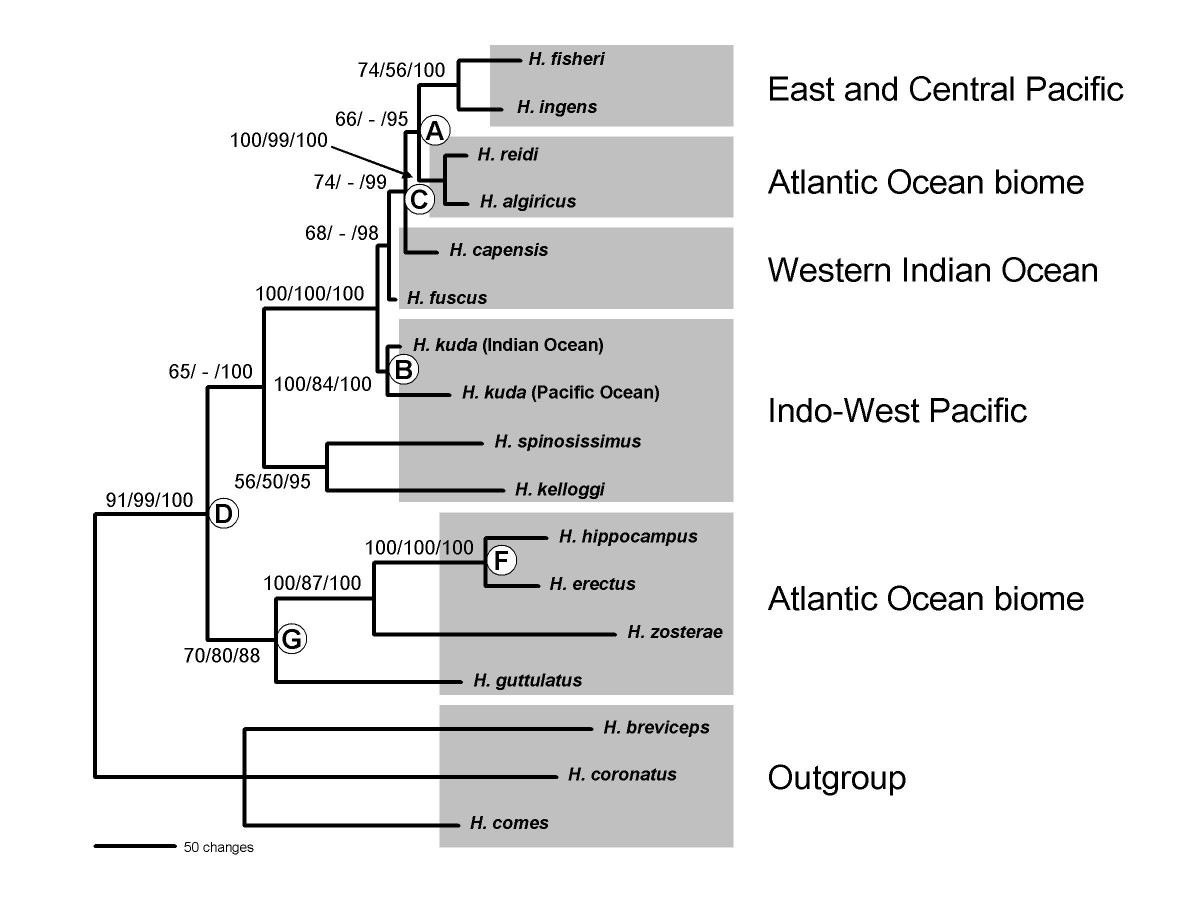

Supplement: Additional file 2 — ZIP files containing several folders, each of which with TreeSnatcher Plus snapshot files, the original image and a text file. [file 1471-2105-13-110-S2.zip › 1471-2148-7-138-2/1471-2148-7-138-2-l.jpg]

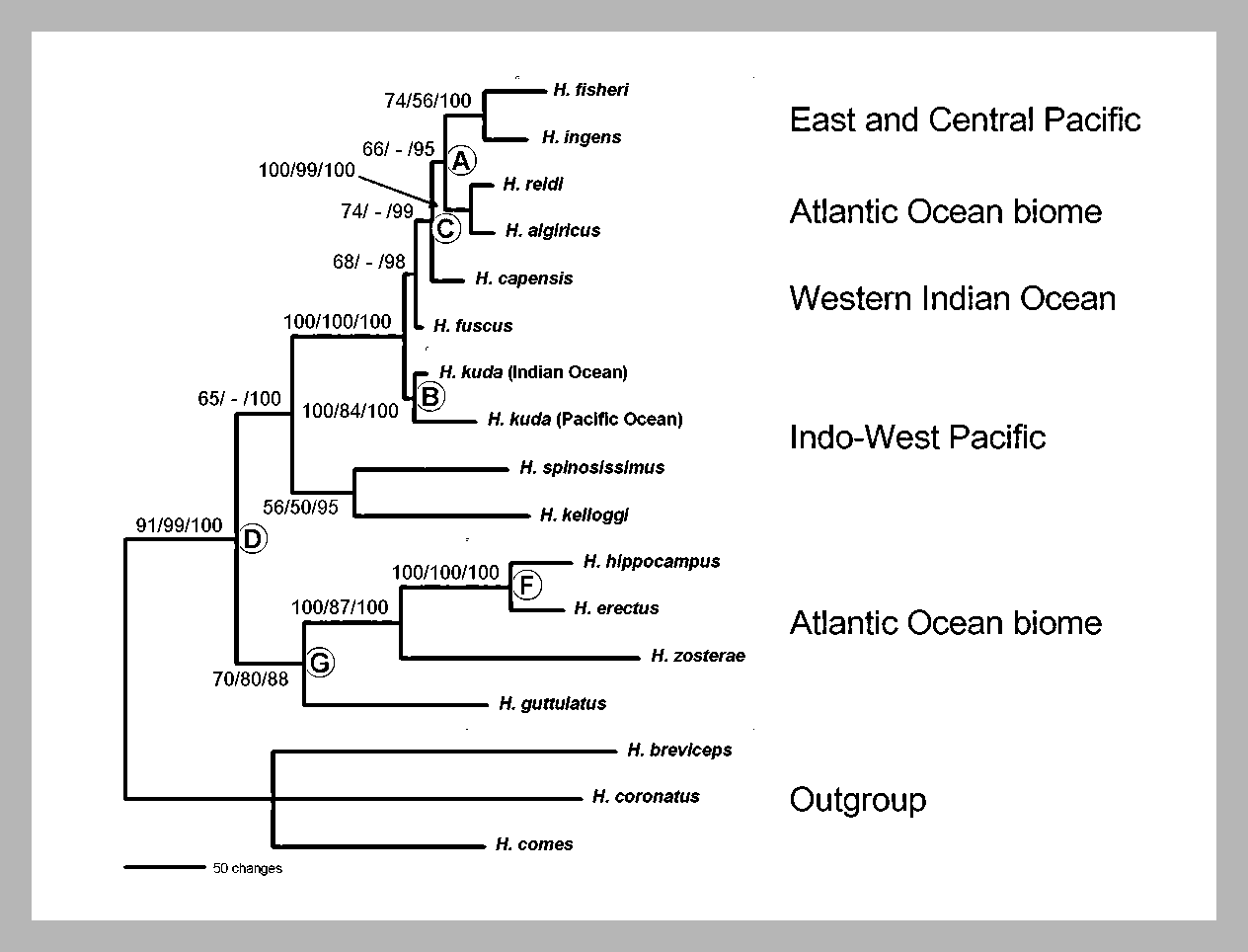

Supplement: Additional file 2 — ZIP files containing several folders, each of which with TreeSnatcher Plus snapshot files, the original image and a text file. [file 1471-2105-13-110-S2.zip › 1471-2148-7-138-2/1471-2148-7-138-2-l_b.PNG]

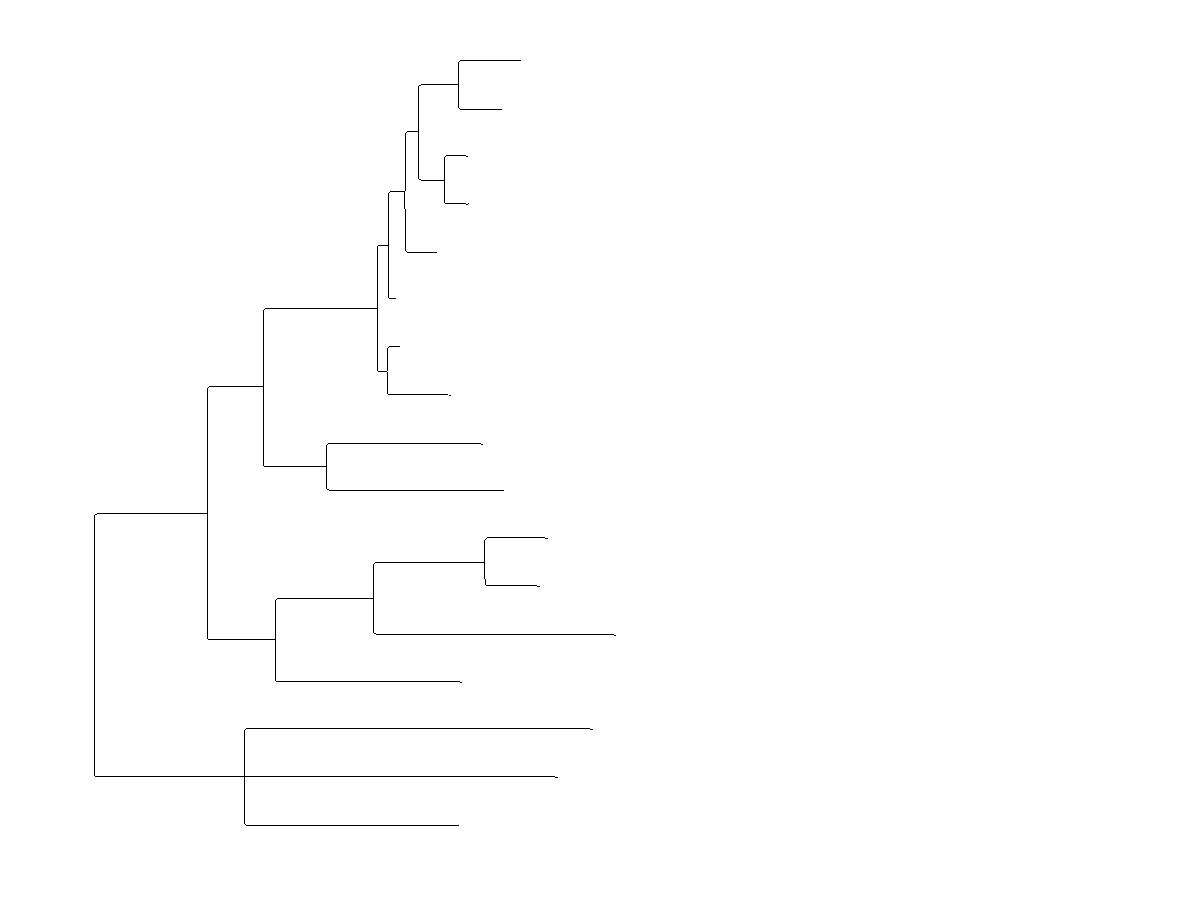

Supplement: Additional file 2 — ZIP files containing several folders, each of which with TreeSnatcher Plus snapshot files, the original image and a text file. [file 1471-2105-13-110-S2.zip › 1471-2148-7-138-2/1471-2148-7-138-2-l_c.PNG]

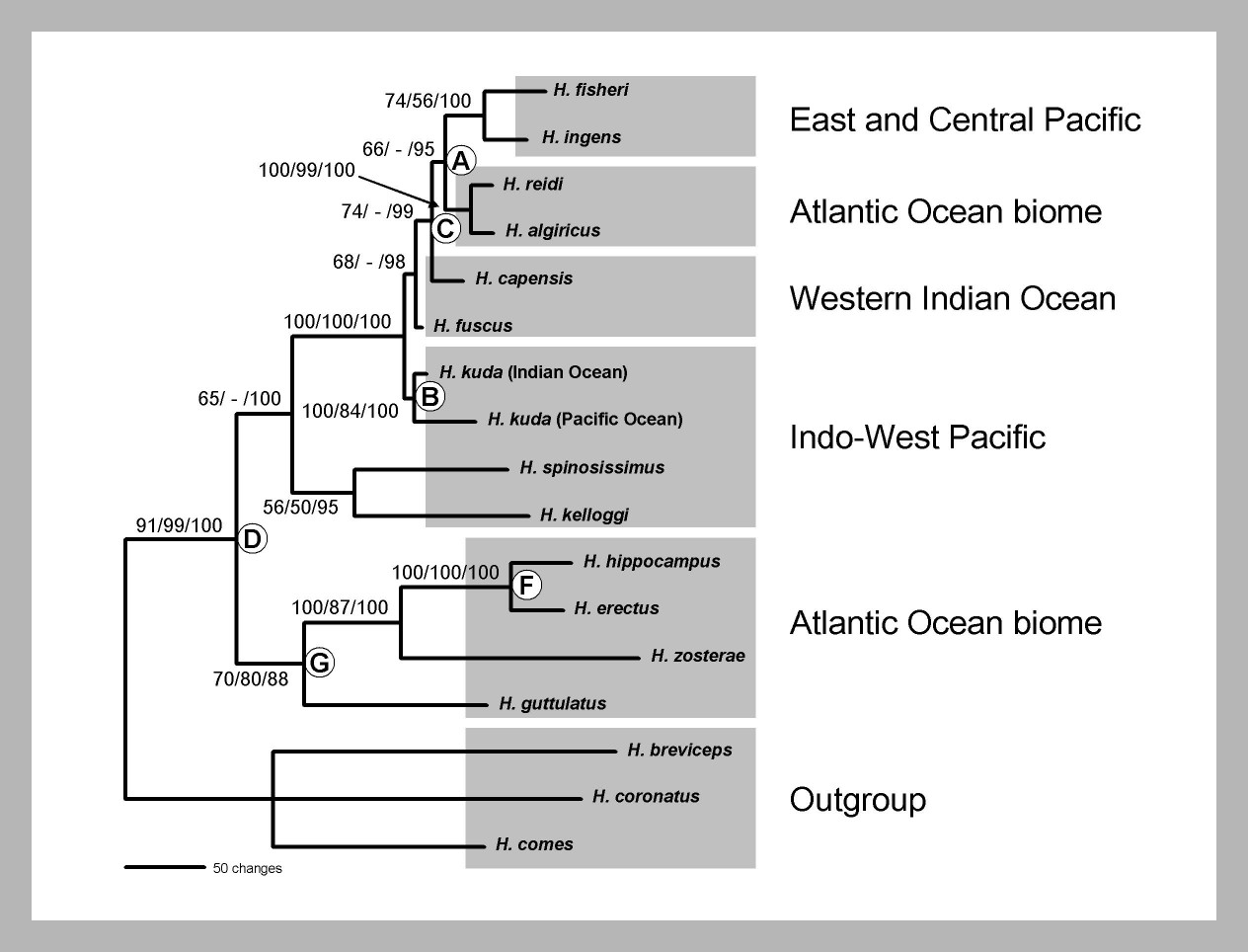

Supplement: Additional file 2 — ZIP files containing several folders, each of which with TreeSnatcher Plus snapshot files, the original image and a text file. [file 1471-2105-13-110-S2.zip › 1471-2148-7-138-2/1471-2148-7-138-2-l_o.PNG]

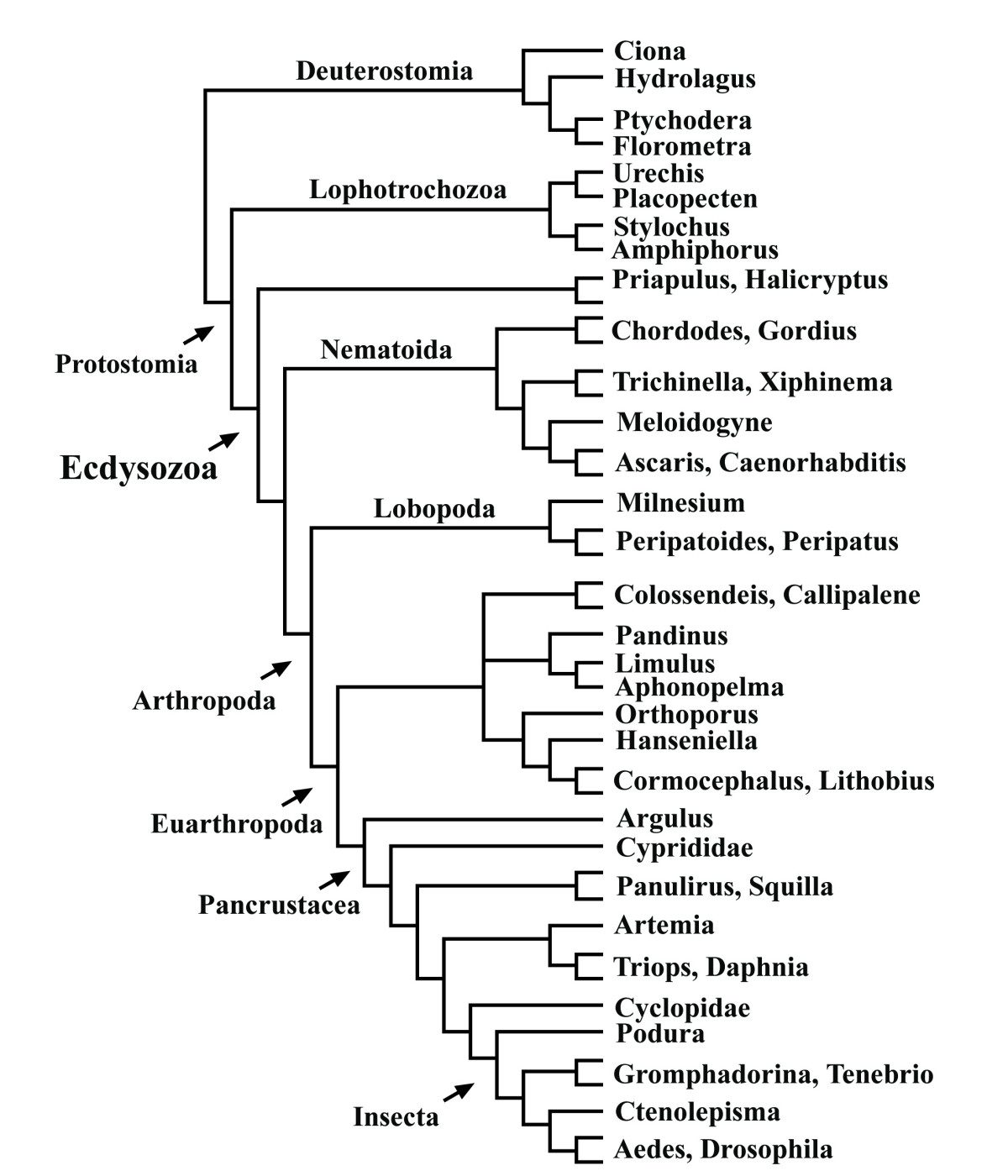

Supplement: Additional file 2 — ZIP files containing several folders, each of which with TreeSnatcher Plus snapshot files, the original image and a text file. [file 1471-2105-13-110-S2.zip › 1471-2148-7-147-17/1471-2148-7-147-17-l.jpg]

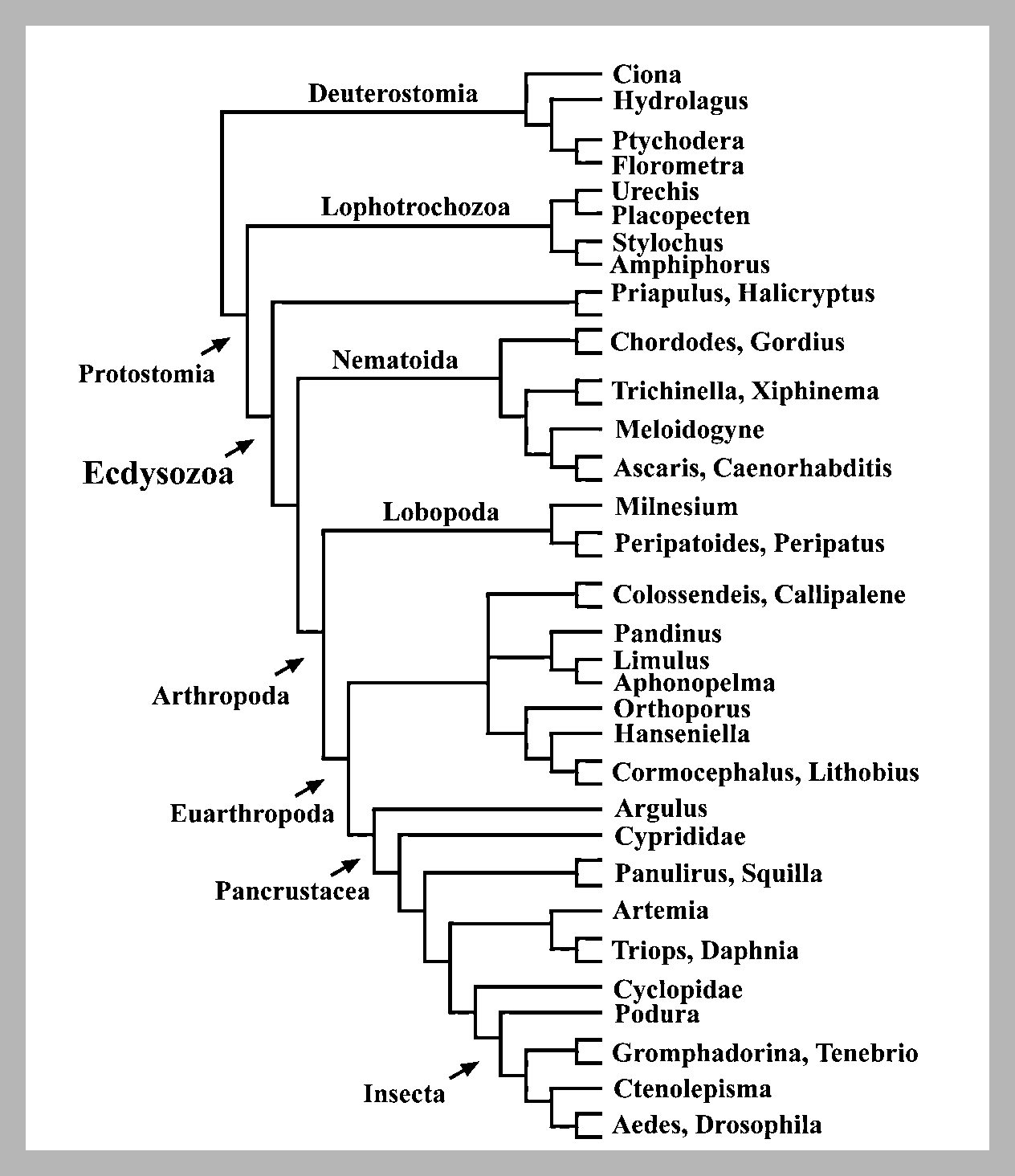

Supplement: Additional file 2 — ZIP files containing several folders, each of which with TreeSnatcher Plus snapshot files, the original image and a text file. [file 1471-2105-13-110-S2.zip › 1471-2148-7-147-17/1471-2148-7-147-17-l_b.PNG]

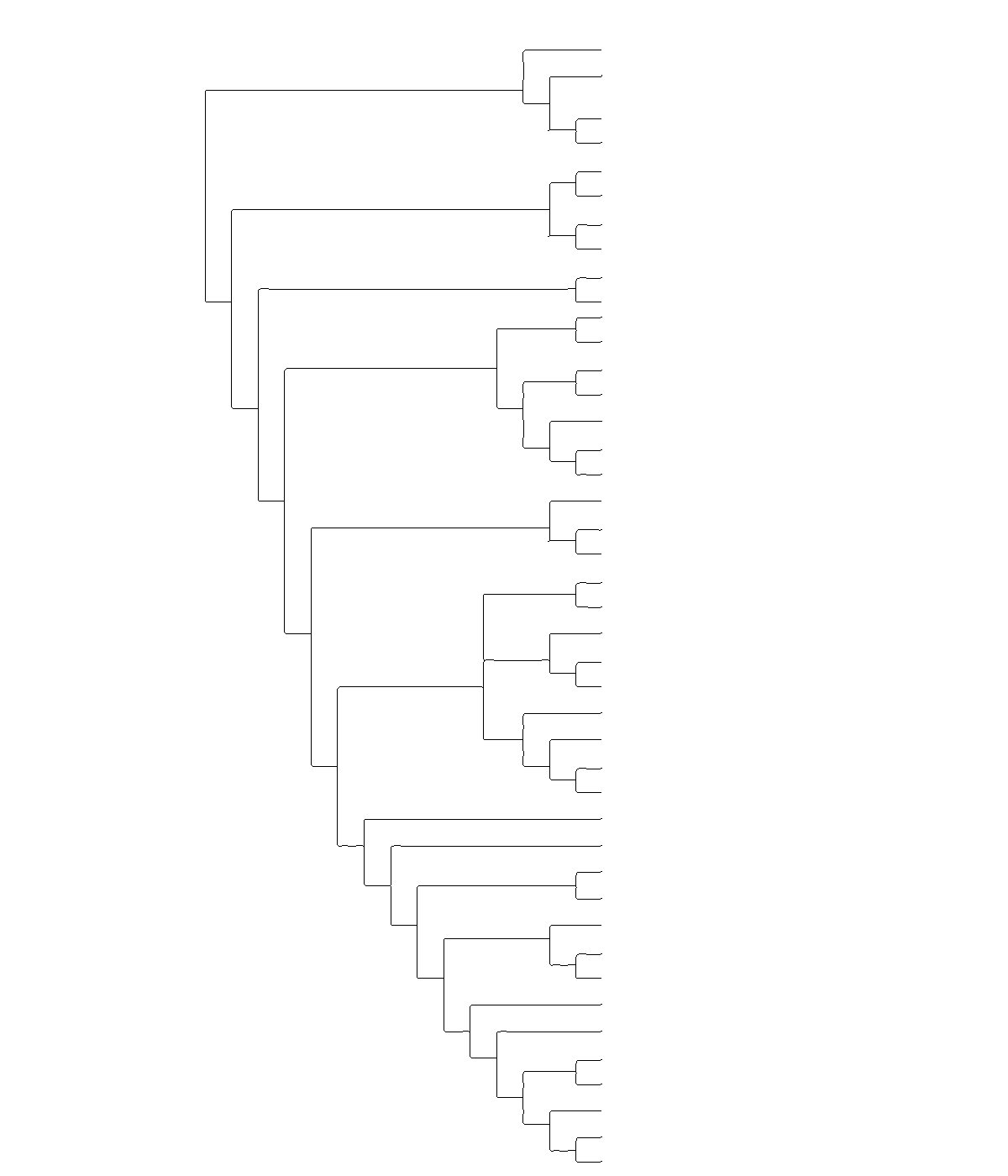

Supplement: Additional file 2 — ZIP files containing several folders, each of which with TreeSnatcher Plus snapshot files, the original image and a text file. [file 1471-2105-13-110-S2.zip › 1471-2148-7-147-17/1471-2148-7-147-17-l_c.PNG]

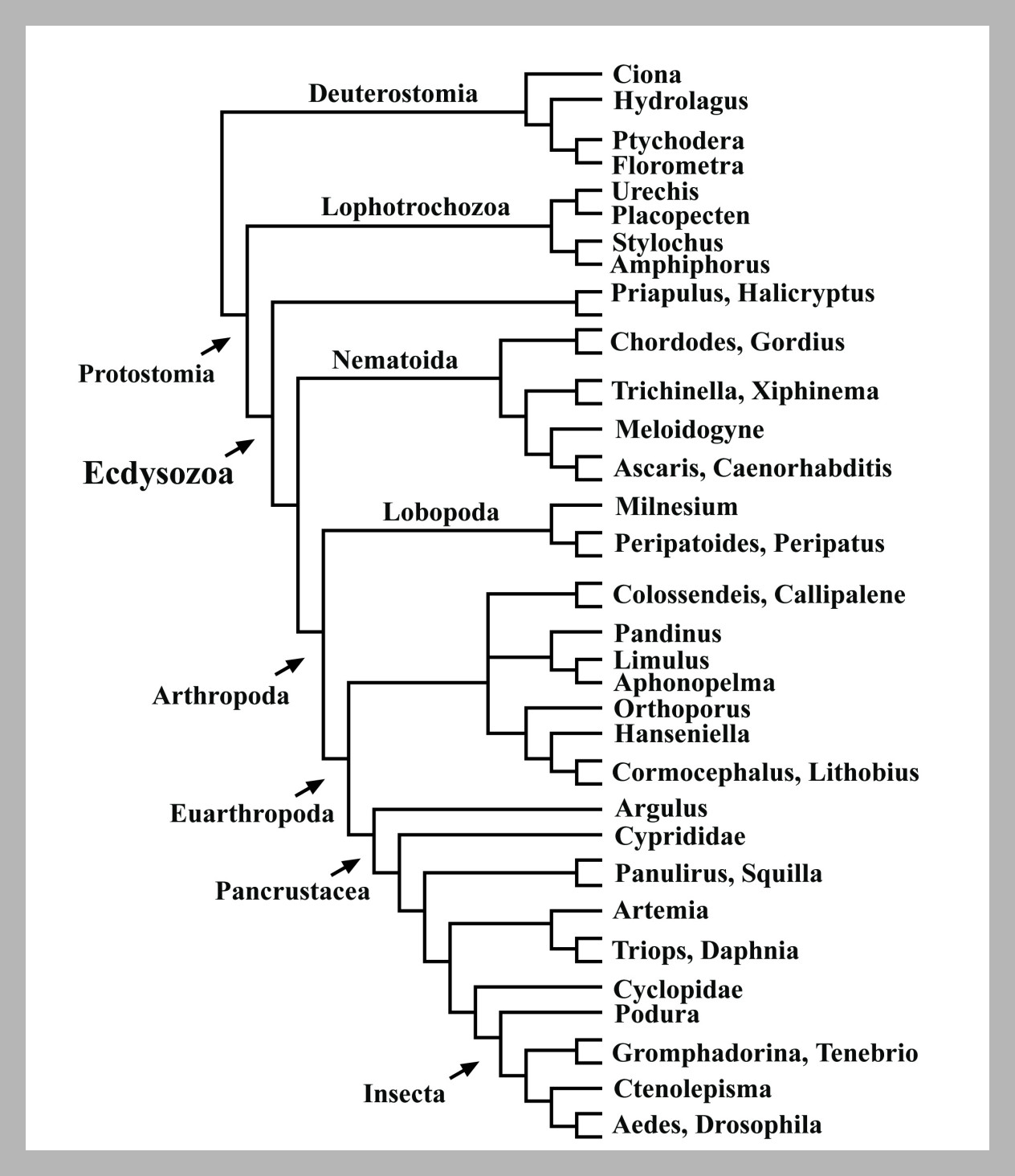

Supplement: Additional file 2 — ZIP files containing several folders, each of which with TreeSnatcher Plus snapshot files, the original image and a text file. [file 1471-2105-13-110-S2.zip › 1471-2148-7-147-17/1471-2148-7-147-17-l_o.PNG]

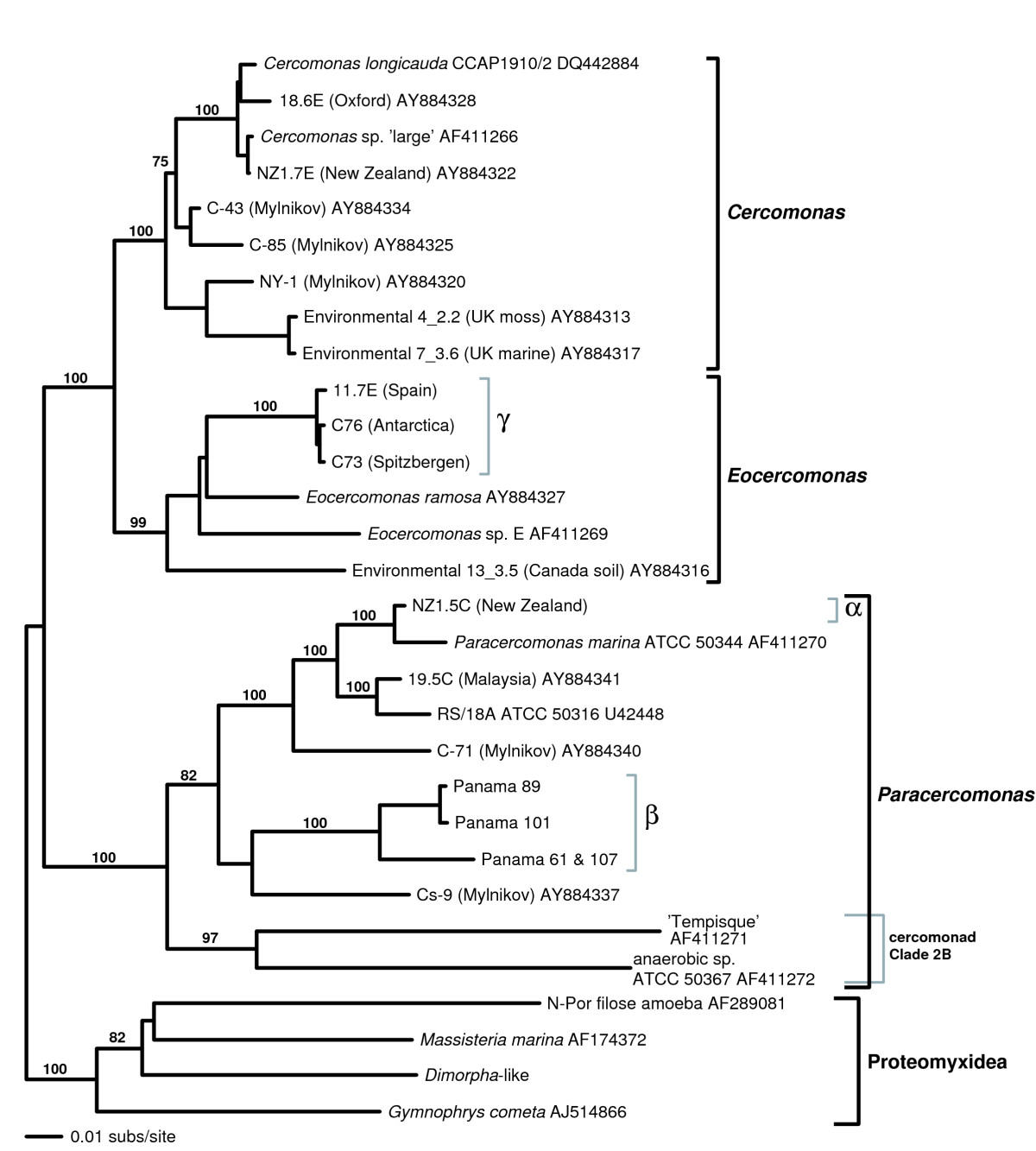

Supplement: Additional file 2 — ZIP files containing several folders, each of which with TreeSnatcher Plus snapshot files, the original image and a text file. [file 1471-2105-13-110-S2.zip › 1471-2148-7-162-2/1471-2148-7-162-2-l.jpg]

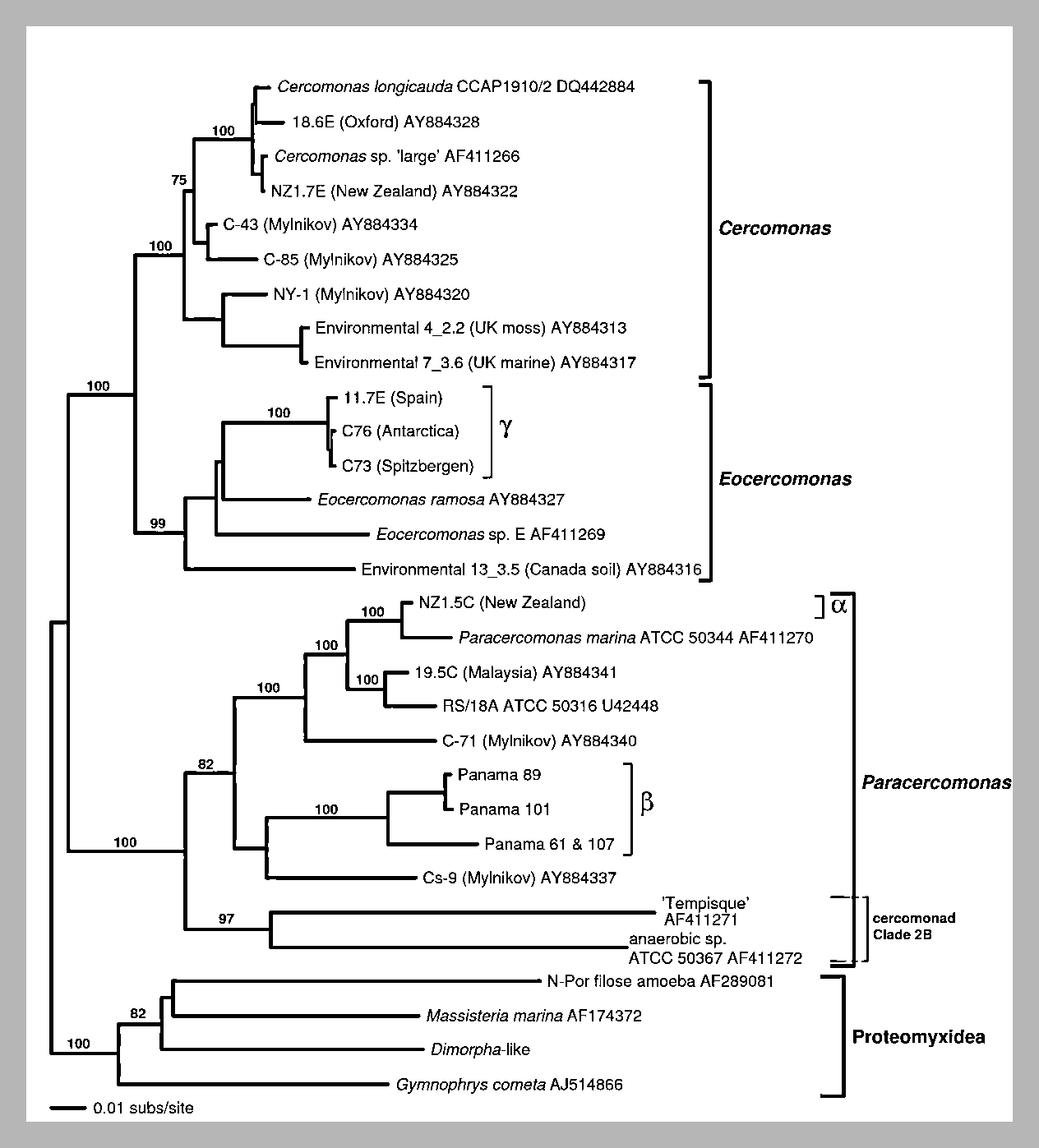

Supplement: Additional file 2 — ZIP files containing several folders, each of which with TreeSnatcher Plus snapshot files, the original image and a text file. [file 1471-2105-13-110-S2.zip › 1471-2148-7-162-2/1471-2148-7-162-2-l_b.PNG]

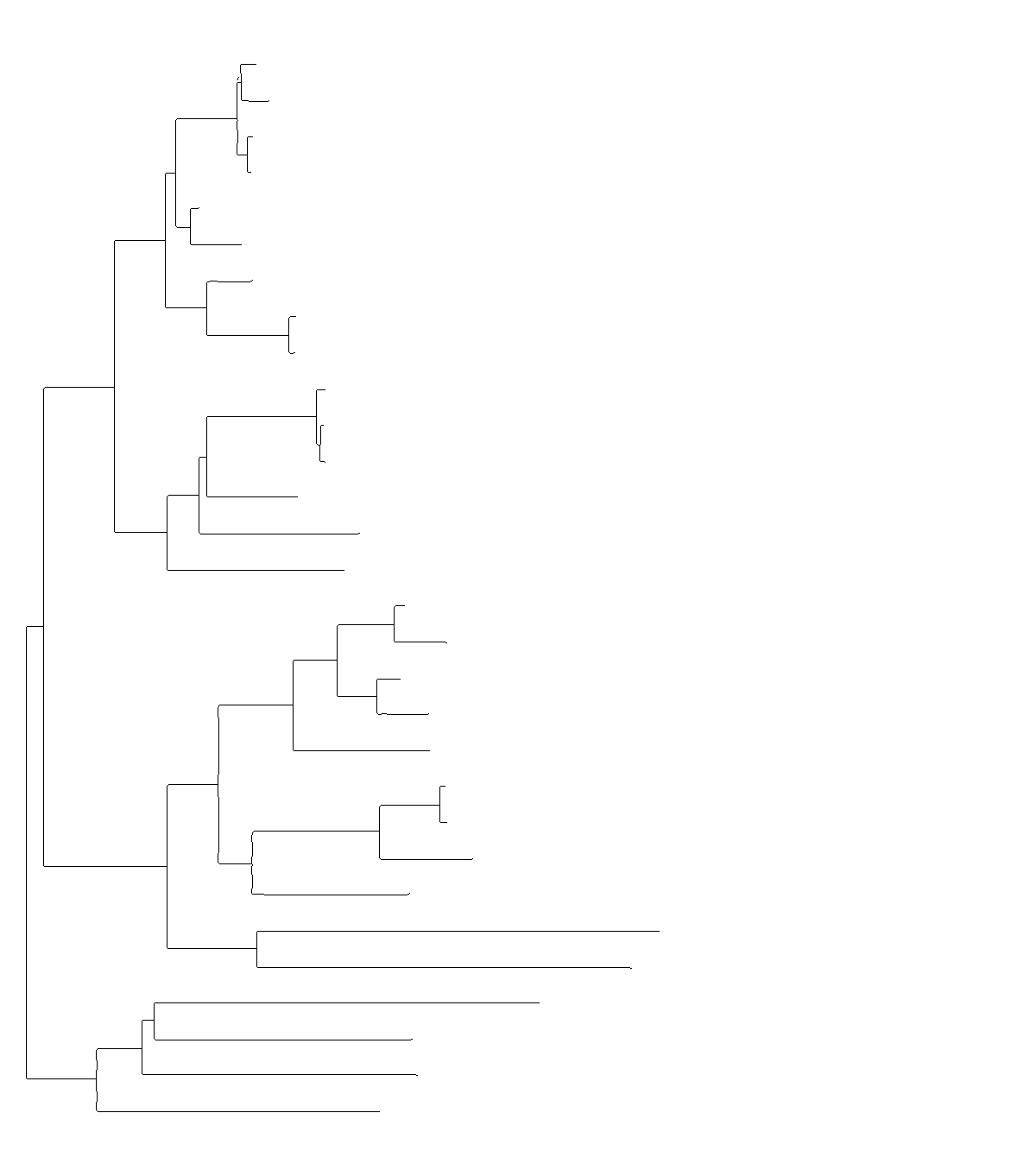

Supplement: Additional file 2 — ZIP files containing several folders, each of which with TreeSnatcher Plus snapshot files, the original image and a text file. [file 1471-2105-13-110-S2.zip › 1471-2148-7-162-2/1471-2148-7-162-2-l_c.PNG]

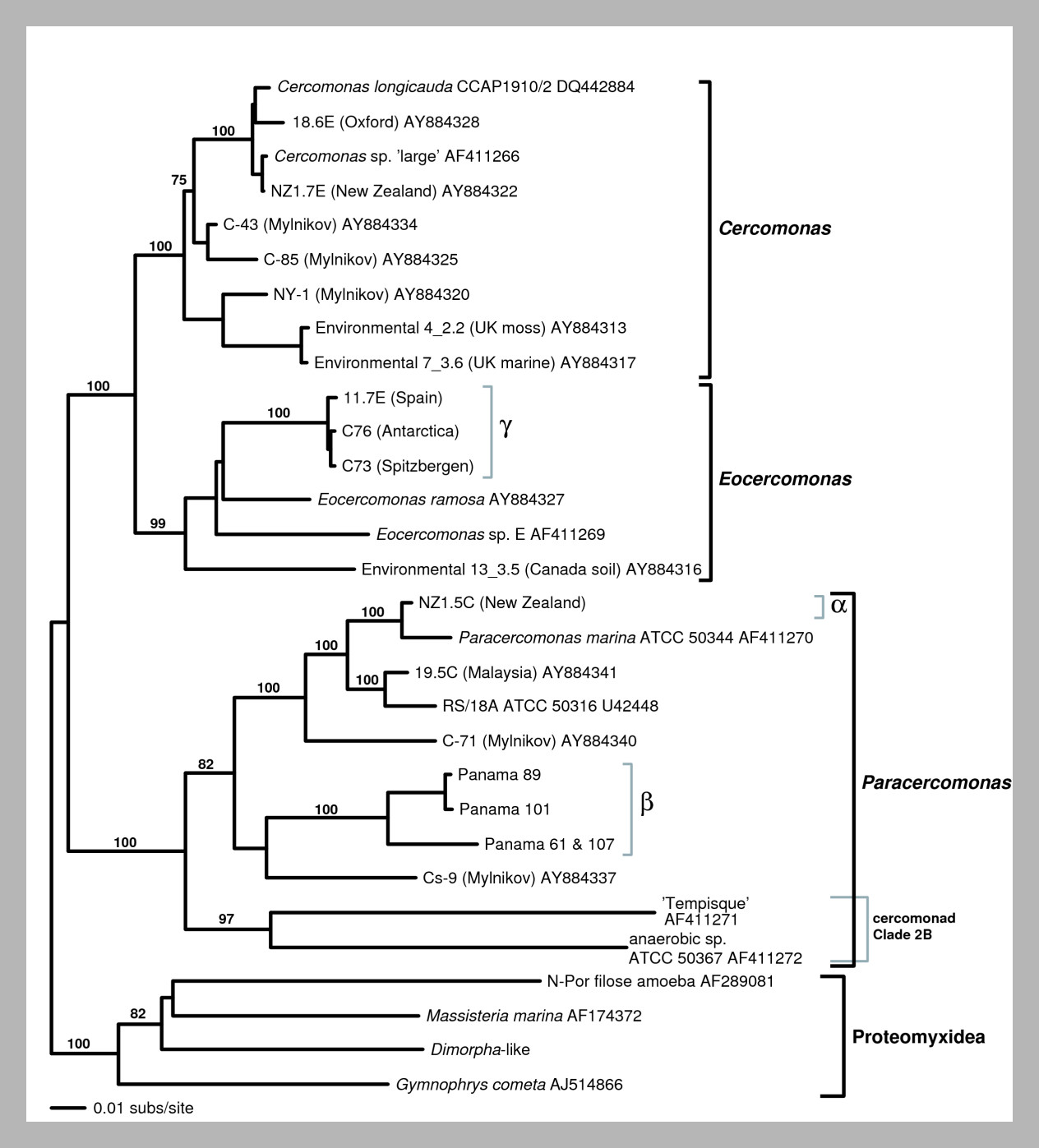

Supplement: Additional file 2 — ZIP files containing several folders, each of which with TreeSnatcher Plus snapshot files, the original image and a text file. [file 1471-2105-13-110-S2.zip › 1471-2148-7-162-2/1471-2148-7-162-2-l_o.PNG]

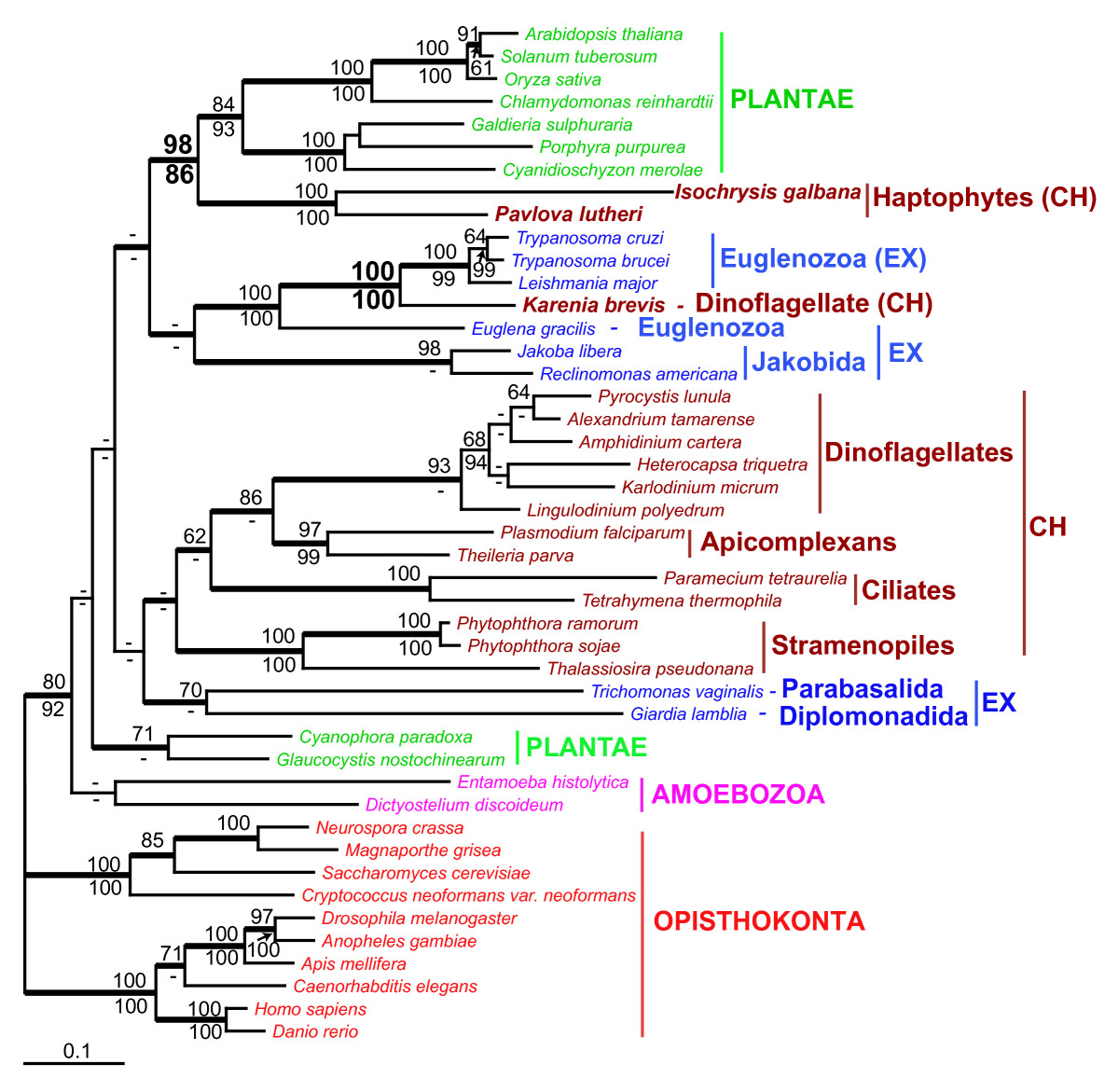

Supplement: Additional file 2 — ZIP files containing several folders, each of which with TreeSnatcher Plus snapshot files, the original image and a text file. [file 1471-2105-13-110-S2.zip › 1471-2148-7-173-7/1471-2148-7-173-7-l.jpg]

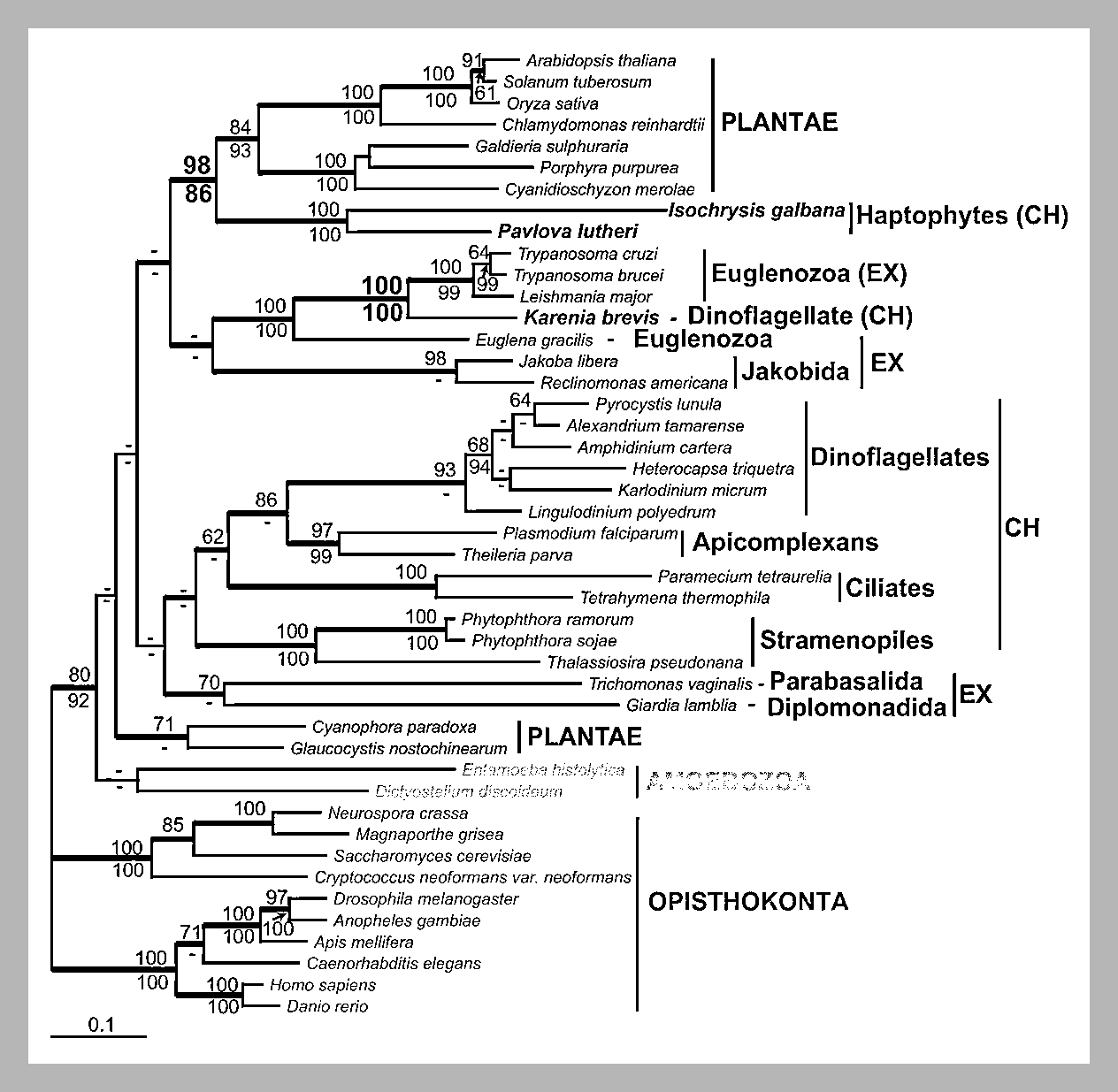

Supplement: Additional file 2 — ZIP files containing several folders, each of which with TreeSnatcher Plus snapshot files, the original image and a text file. [file 1471-2105-13-110-S2.zip › 1471-2148-7-173-7/1471-2148-7-173-7-l_b.PNG]

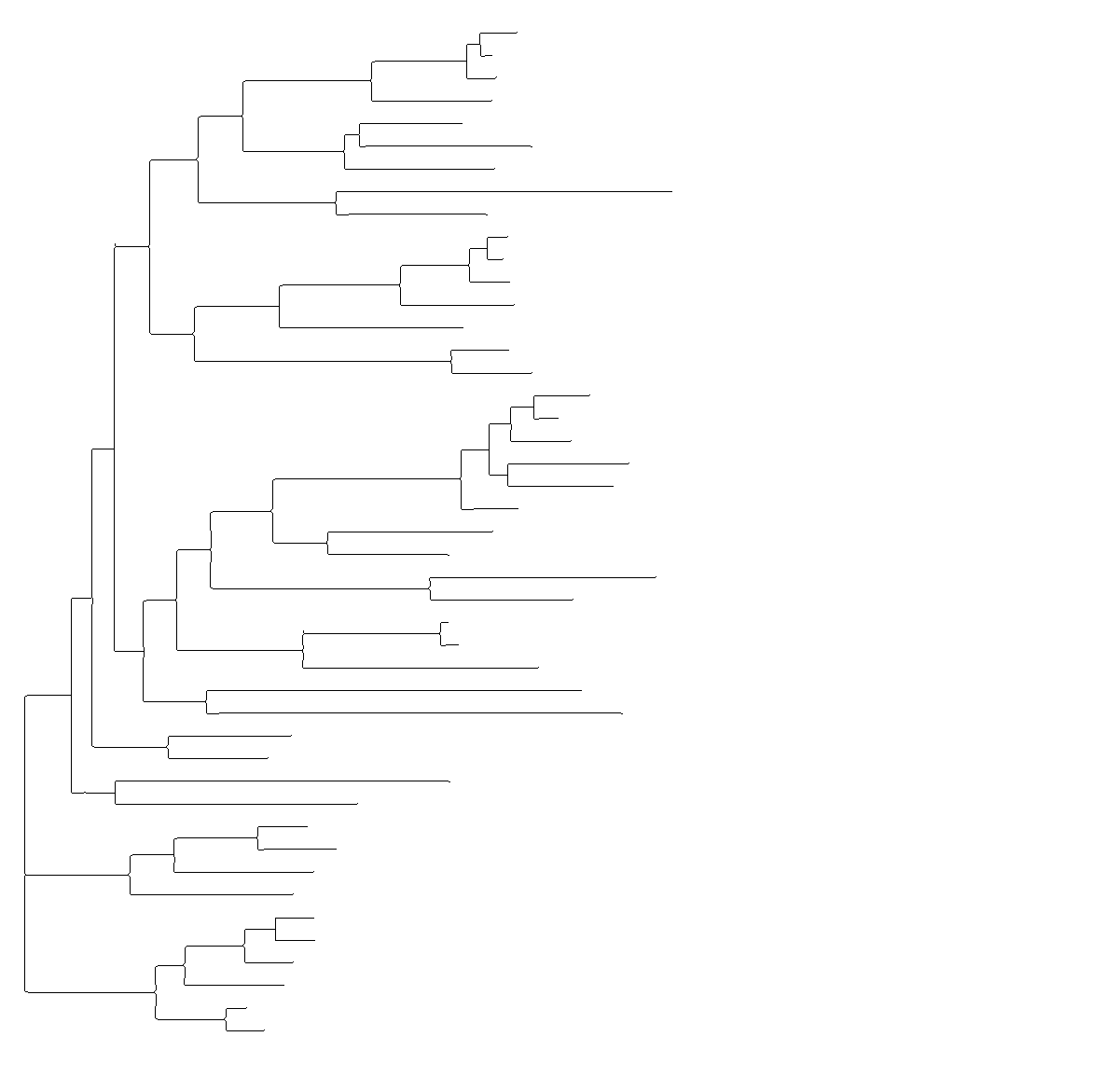

Supplement: Additional file 2 — ZIP files containing several folders, each of which with TreeSnatcher Plus snapshot files, the original image and a text file. [file 1471-2105-13-110-S2.zip › 1471-2148-7-173-7/1471-2148-7-173-7-l_c.PNG]

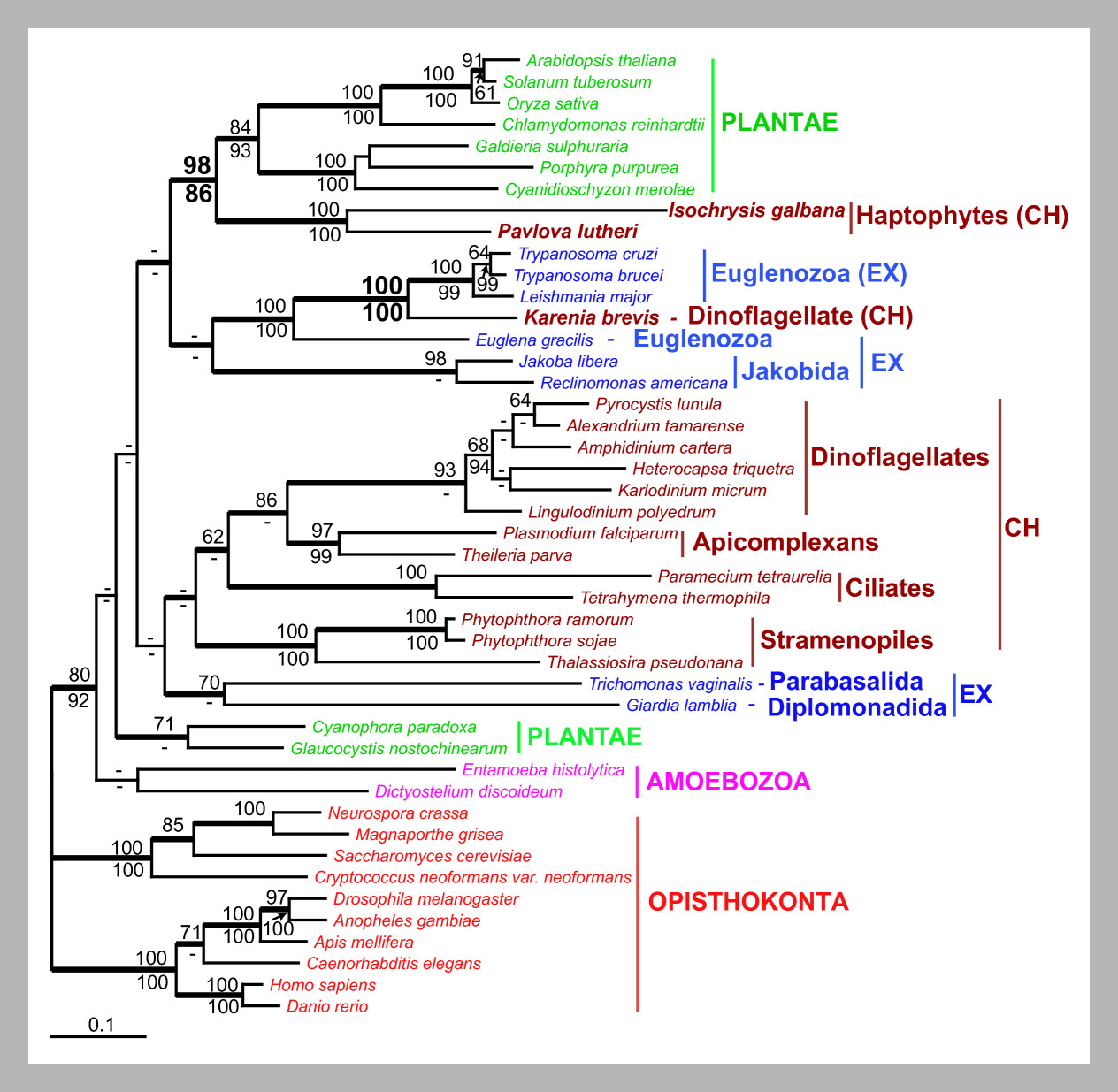

Supplement: Additional file 2 — ZIP files containing several folders, each of which with TreeSnatcher Plus snapshot files, the original image and a text file. [file 1471-2105-13-110-S2.zip › 1471-2148-7-173-7/1471-2148-7-173-7-l_o.PNG]

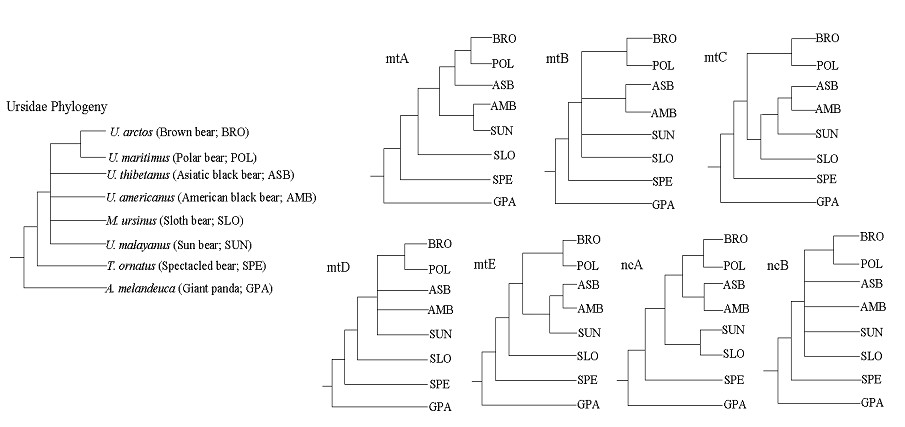

Supplement: Additional file 2 — ZIP files containing several folders, each of which with TreeSnatcher Plus snapshot files, the original image and a text file. [file 1471-2105-13-110-S2.zip › 1471-2148-7-198-1/1471-2148-7-198-1-l.jpg]

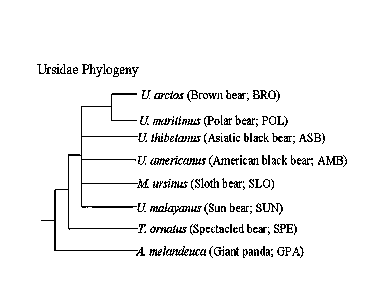

Supplement: Additional file 2 — ZIP files containing several folders, each of which with TreeSnatcher Plus snapshot files, the original image and a text file. [file 1471-2105-13-110-S2.zip › 1471-2148-7-198-1/1471-2148-7-198-1-l_b.PNG]

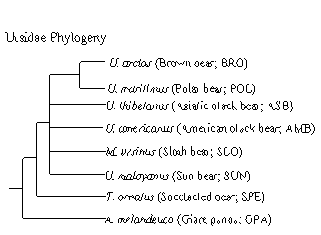

Supplement: Additional file 2 — ZIP files containing several folders, each of which with TreeSnatcher Plus snapshot files, the original image and a text file. [file 1471-2105-13-110-S2.zip › 1471-2148-7-198-1/1471-2148-7-198-1-l_c.PNG]

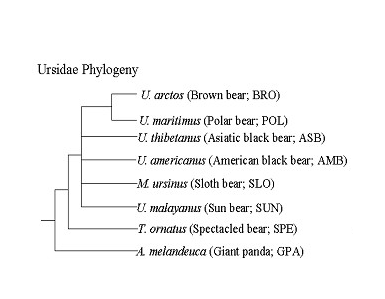

Supplement: Additional file 2 — ZIP files containing several folders, each of which with TreeSnatcher Plus snapshot files, the original image and a text file. [file 1471-2105-13-110-S2.zip › 1471-2148-7-198-1/1471-2148-7-198-1-l_o.PNG]

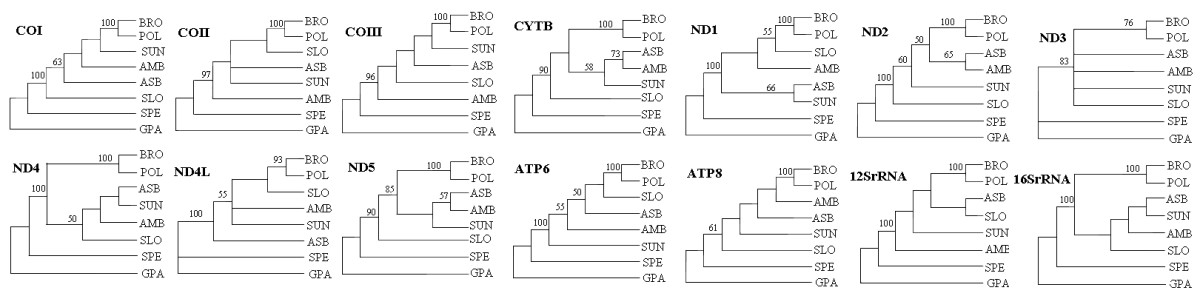

Supplement: Additional file 2 — ZIP files containing several folders, each of which with TreeSnatcher Plus snapshot files, the original image and a text file. [file 1471-2105-13-110-S2.zip › 1471-2148-7-198-4/1471-2148-7-198-4-l.jpg]

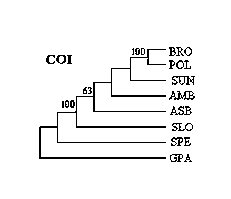

Supplement: Additional file 2 — ZIP files containing several folders, each of which with TreeSnatcher Plus snapshot files, the original image and a text file. [file 1471-2105-13-110-S2.zip › 1471-2148-7-198-4/1471-2148-7-198-4-l_b.PNG]

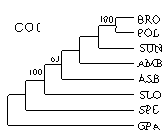

Supplement: Additional file 2 — ZIP files containing several folders, each of which with TreeSnatcher Plus snapshot files, the original image and a text file. [file 1471-2105-13-110-S2.zip › 1471-2148-7-198-4/1471-2148-7-198-4-l_c.PNG]

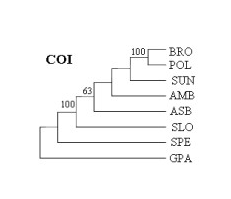

Supplement: Additional file 2 — ZIP files containing several folders, each of which with TreeSnatcher Plus snapshot files, the original image and a text file. [file 1471-2105-13-110-S2.zip › 1471-2148-7-198-4/1471-2148-7-198-4-l_o.PNG]

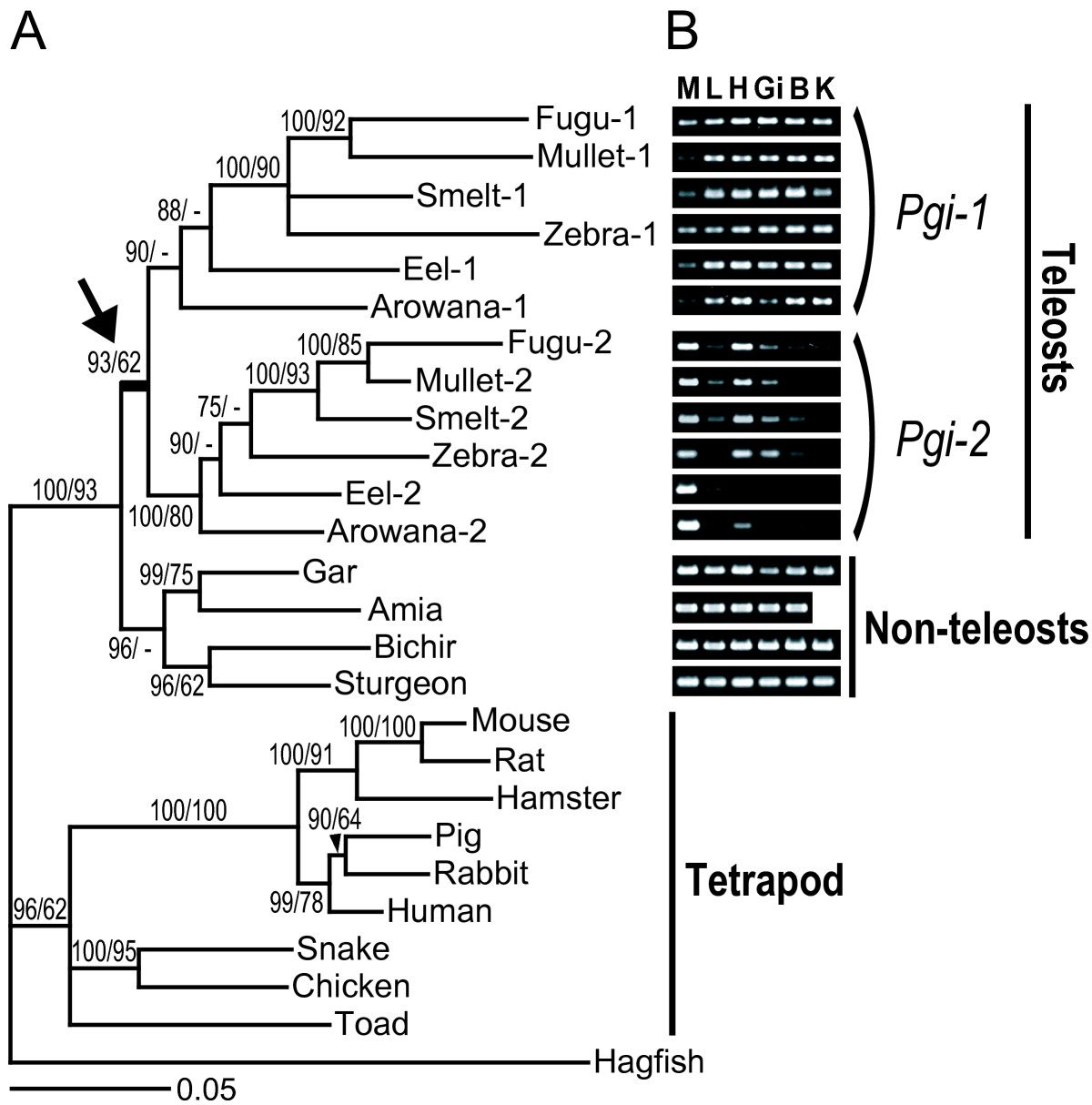

Supplement: Additional file 2 — ZIP files containing several folders, each of which with TreeSnatcher Plus snapshot files, the original image and a text file. [file 1471-2105-13-110-S2.zip › 1471-2148-7-204-1/1471-2148-7-204-1-l.jpg]

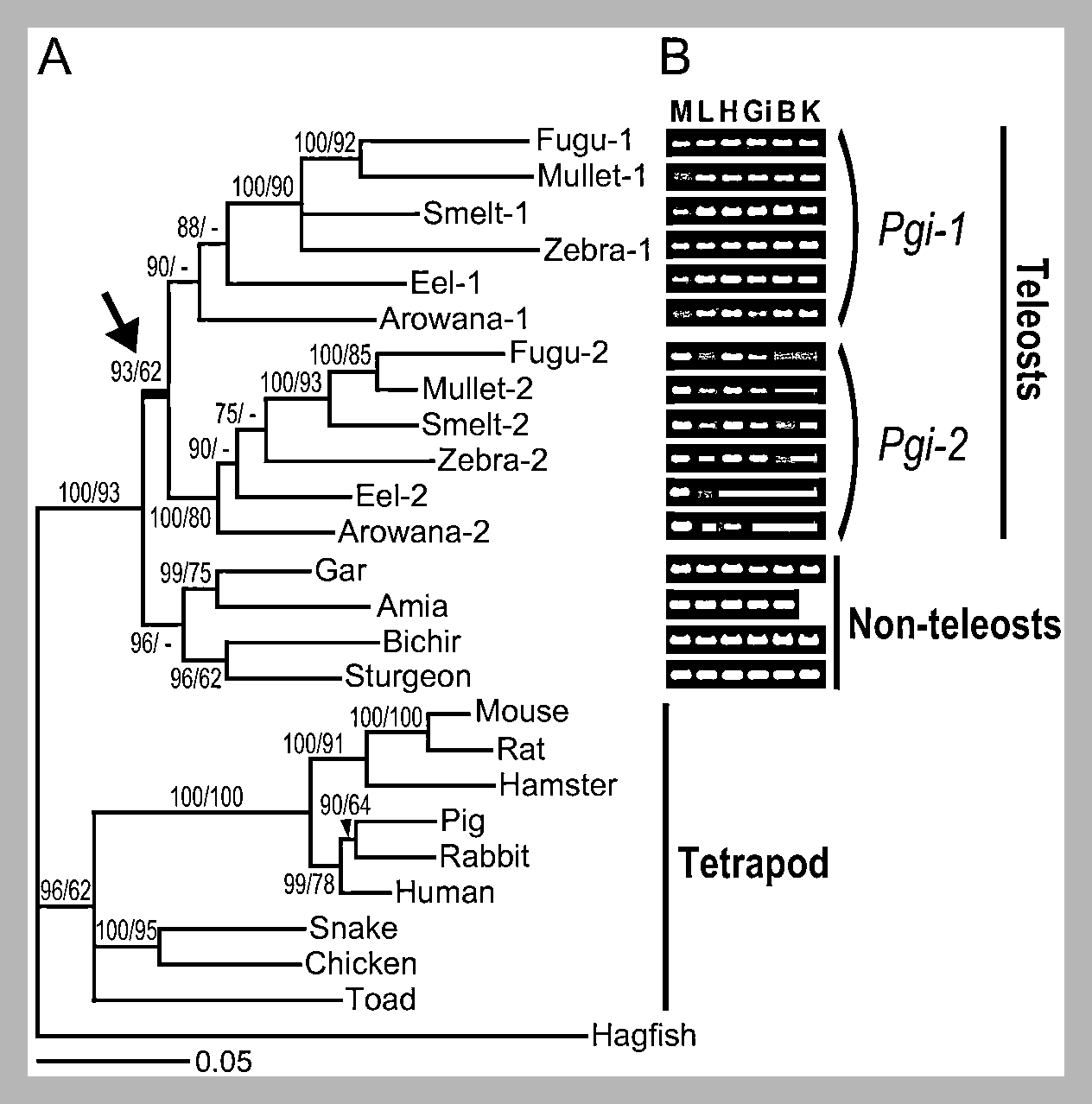

Supplement: Additional file 2 — ZIP files containing several folders, each of which with TreeSnatcher Plus snapshot files, the original image and a text file. [file 1471-2105-13-110-S2.zip › 1471-2148-7-204-1/1471-2148-7-204-1-l_b.PNG]

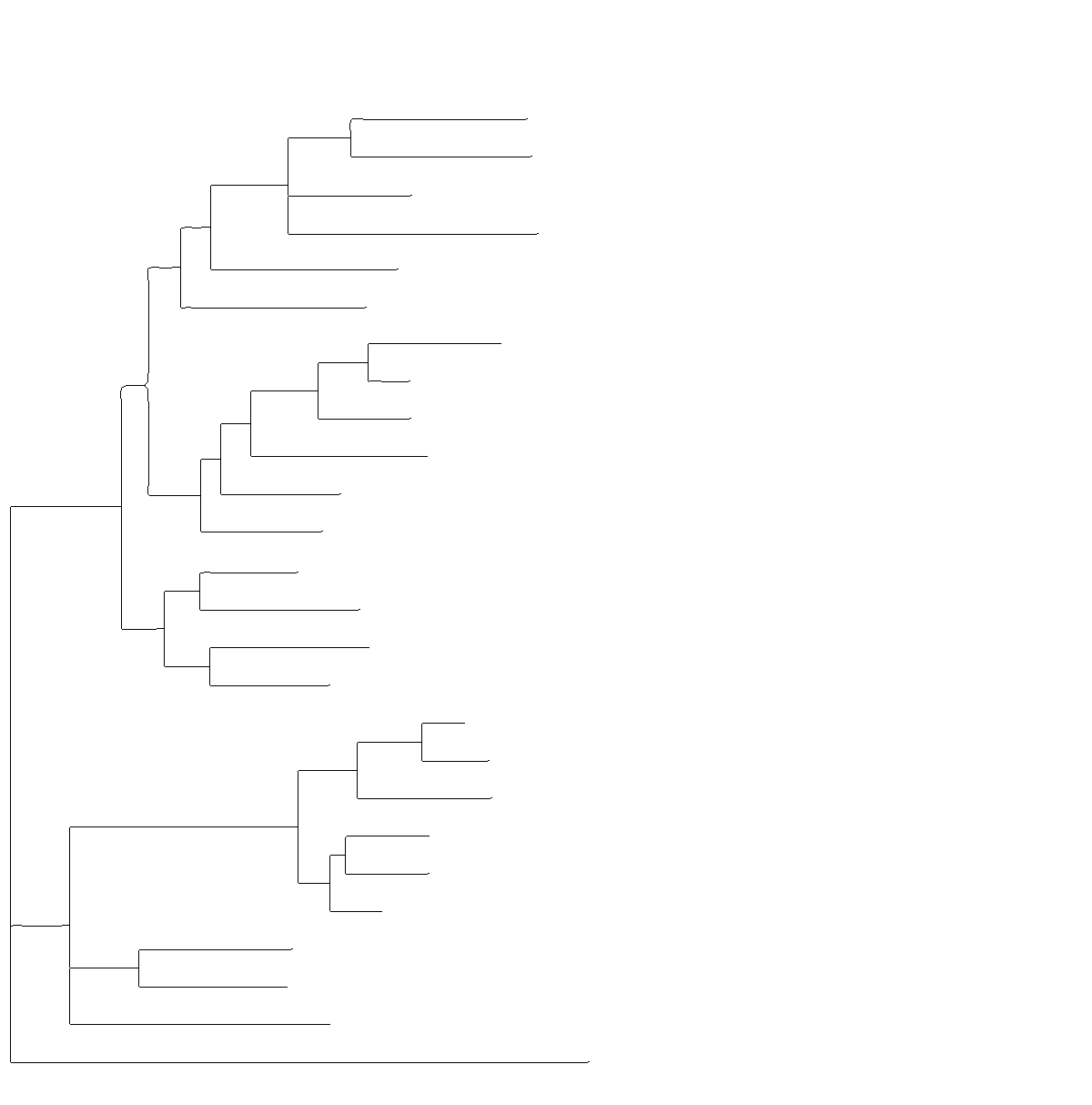

Supplement: Additional file 2 — ZIP files containing several folders, each of which with TreeSnatcher Plus snapshot files, the original image and a text file. [file 1471-2105-13-110-S2.zip › 1471-2148-7-204-1/1471-2148-7-204-1-l_c.PNG]

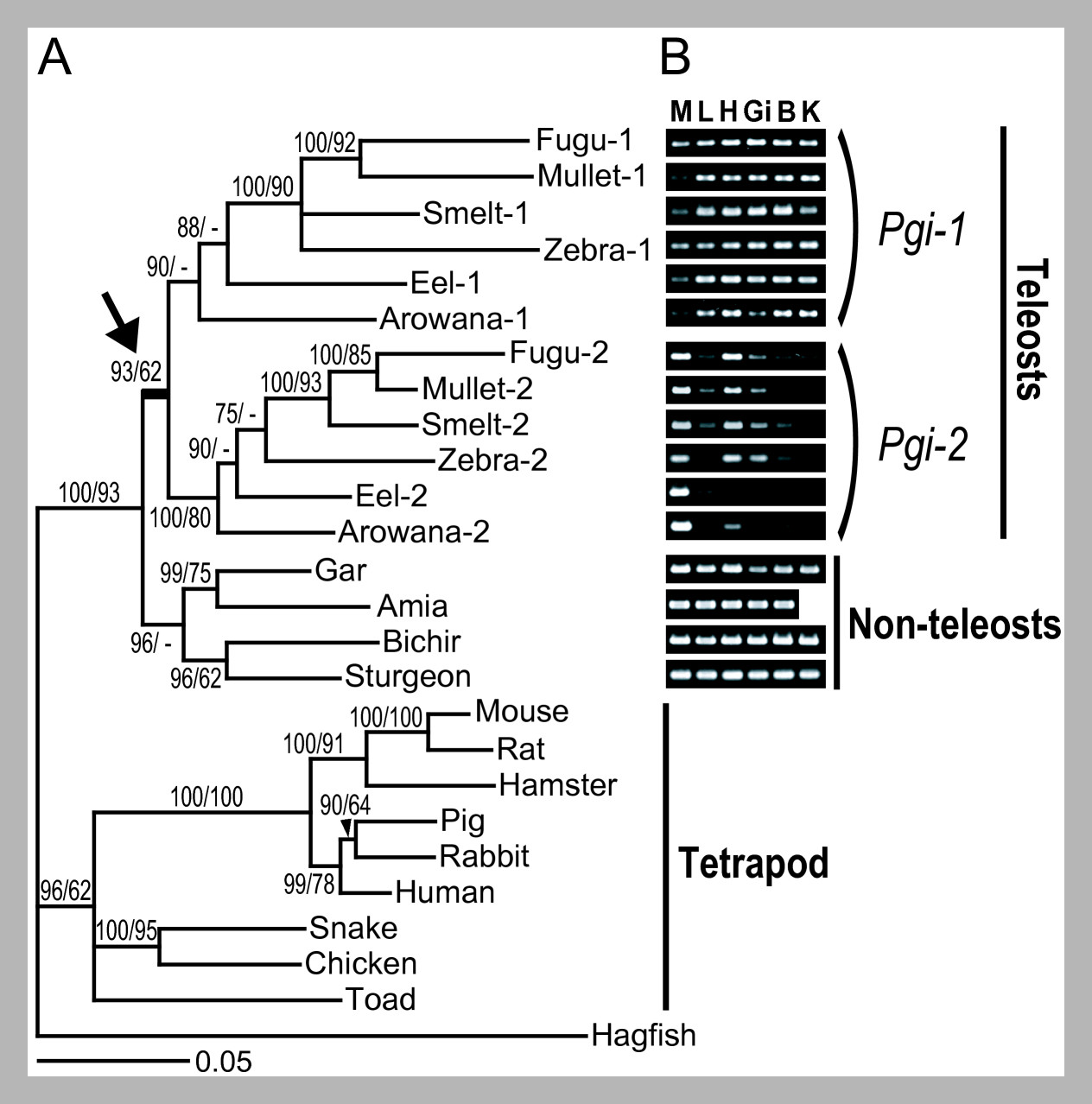

Supplement: Additional file 2 — ZIP files containing several folders, each of which with TreeSnatcher Plus snapshot files, the original image and a text file. [file 1471-2105-13-110-S2.zip › 1471-2148-7-204-1/1471-2148-7-204-1-l_o.PNG]

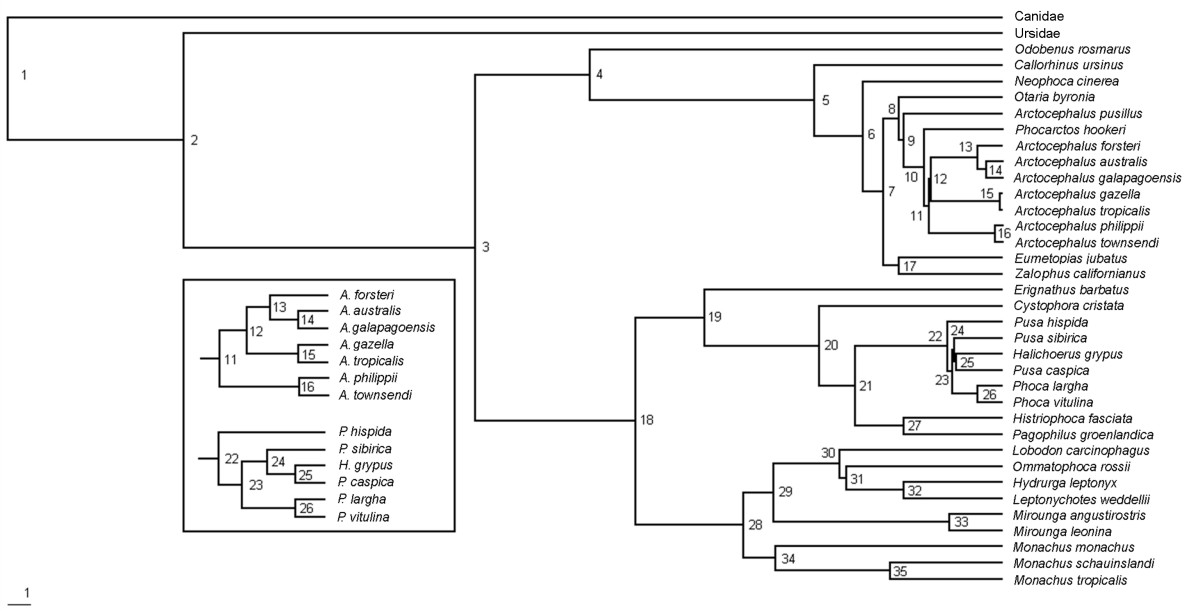

Supplement: Additional file 2 — ZIP files containing several folders, each of which with TreeSnatcher Plus snapshot files, the original image and a text file. [file 1471-2105-13-110-S2.zip › 1471-2148-7-216-1/1471-2148-7-216-1-l.jpg]

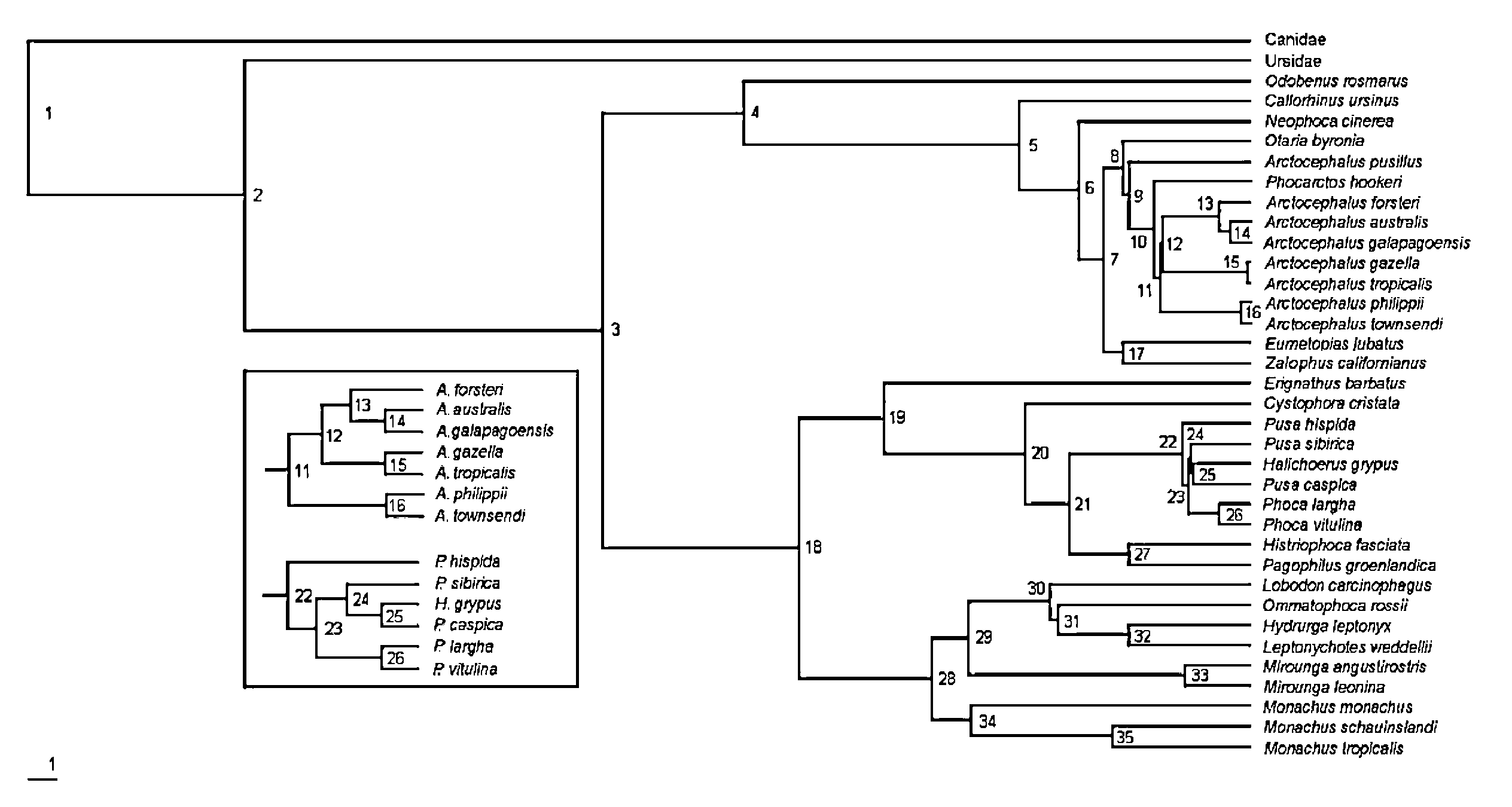

Supplement: Additional file 2 — ZIP files containing several folders, each of which with TreeSnatcher Plus snapshot files, the original image and a text file. [file 1471-2105-13-110-S2.zip › 1471-2148-7-216-1/1471-2148-7-216-1-l_b.PNG]

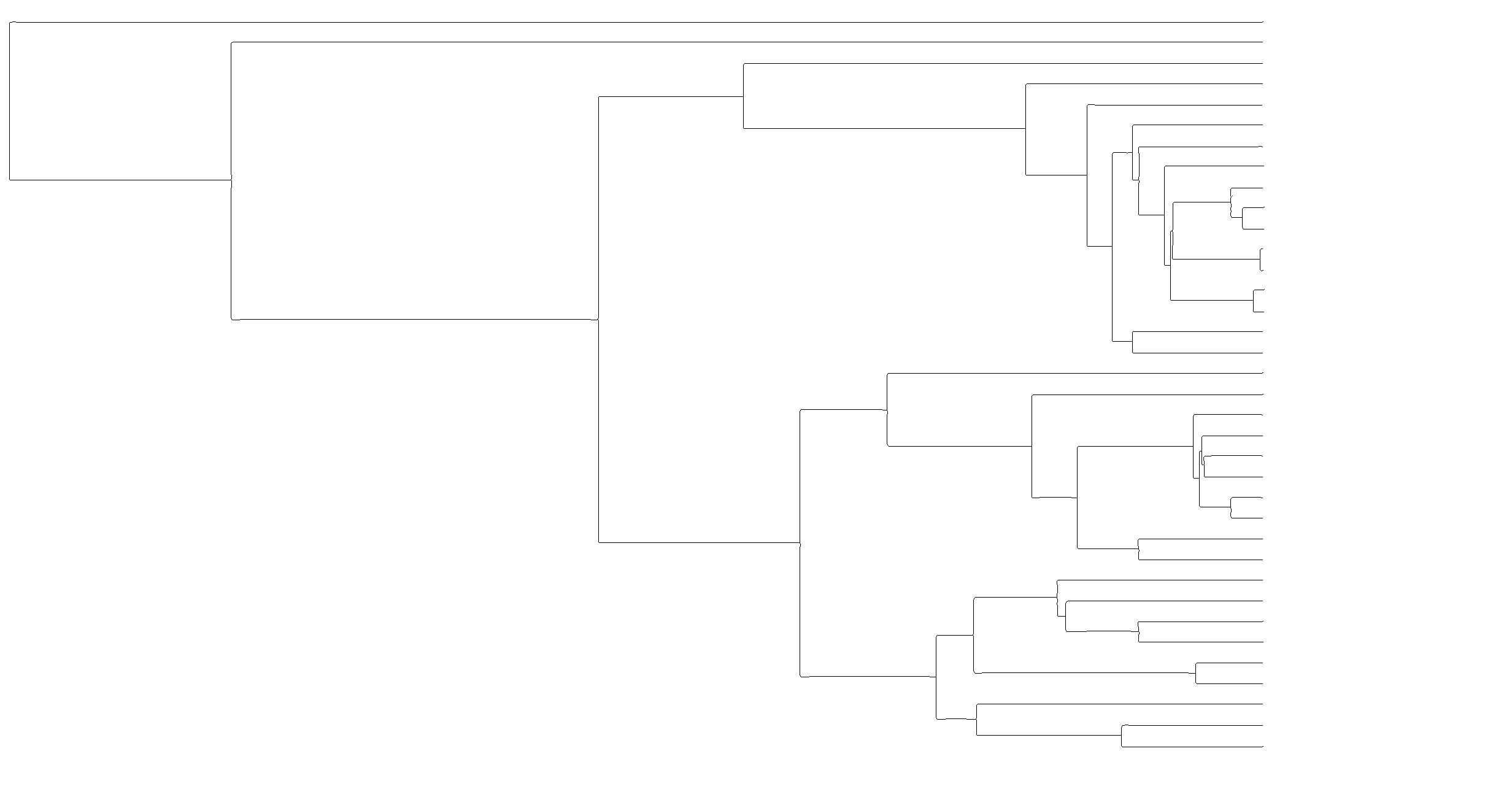

Supplement: Additional file 2 — ZIP files containing several folders, each of which with TreeSnatcher Plus snapshot files, the original image and a text file. [file 1471-2105-13-110-S2.zip › 1471-2148-7-216-1/1471-2148-7-216-1-l_c.PNG]

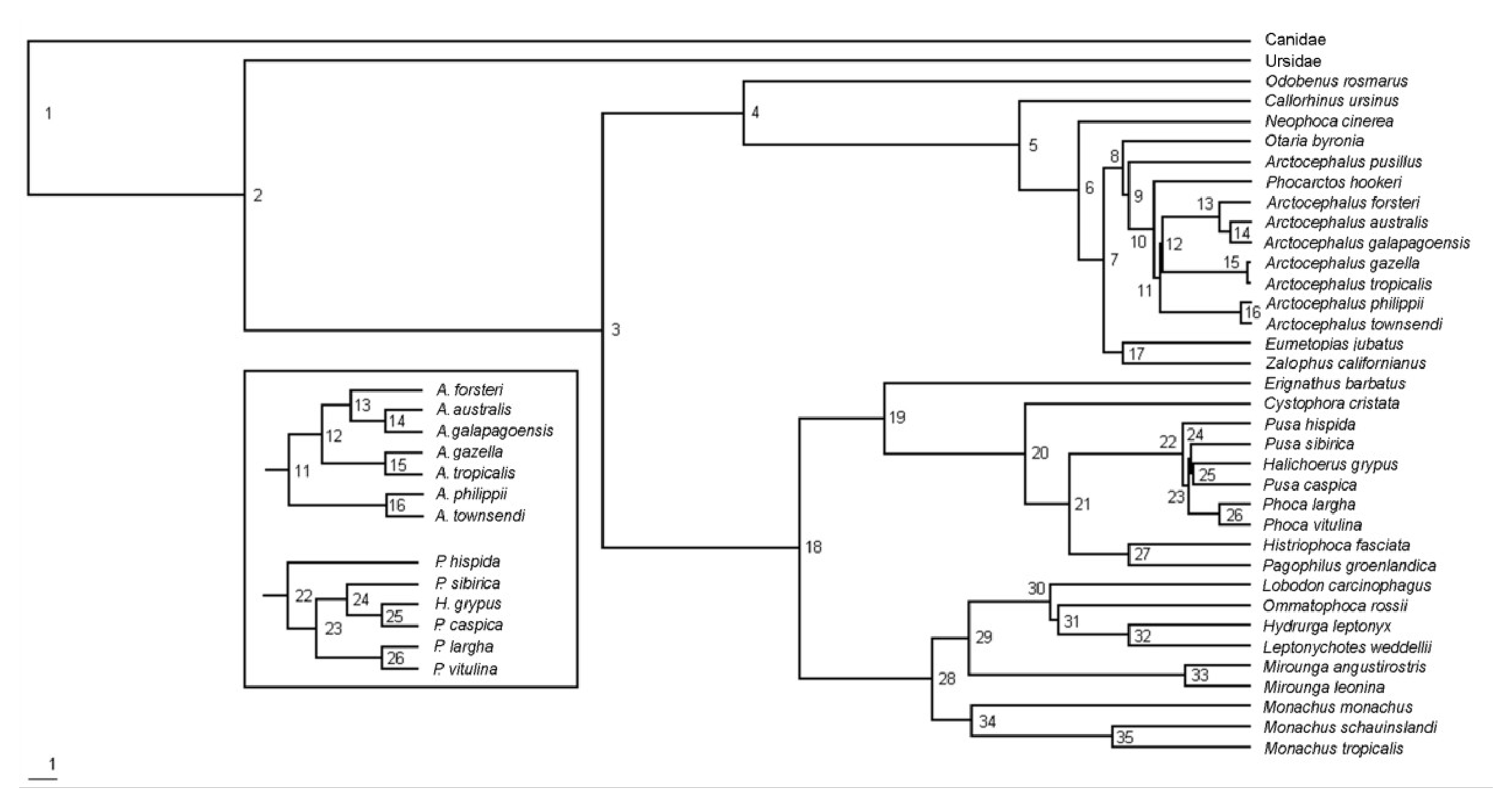

Supplement: Additional file 2 — ZIP files containing several folders, each of which with TreeSnatcher Plus snapshot files, the original image and a text file. [file 1471-2105-13-110-S2.zip › 1471-2148-7-216-1/1471-2148-7-216-1-l_o.PNG]

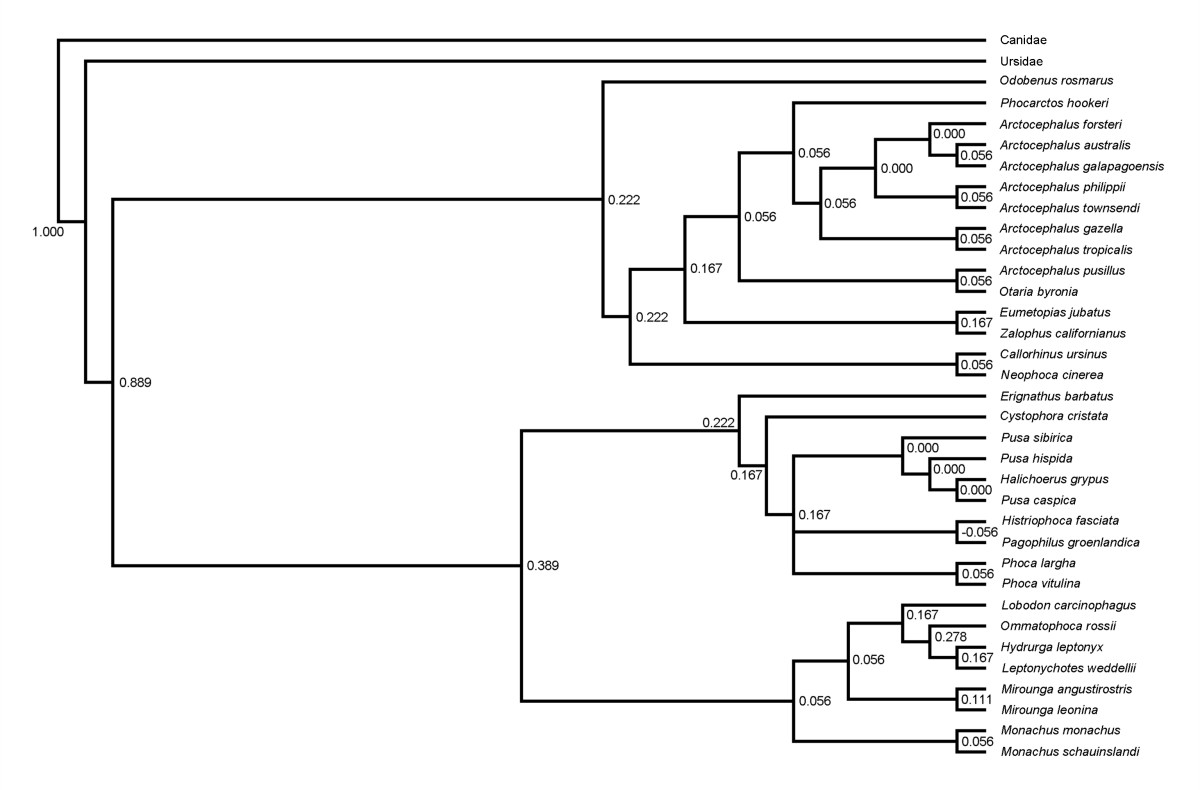

Supplement: Additional file 2 — ZIP files containing several folders, each of which with TreeSnatcher Plus snapshot files, the original image and a text file. [file 1471-2105-13-110-S2.zip › 1471-2148-7-216-2/1471-2148-7-216-2-l.jpg]

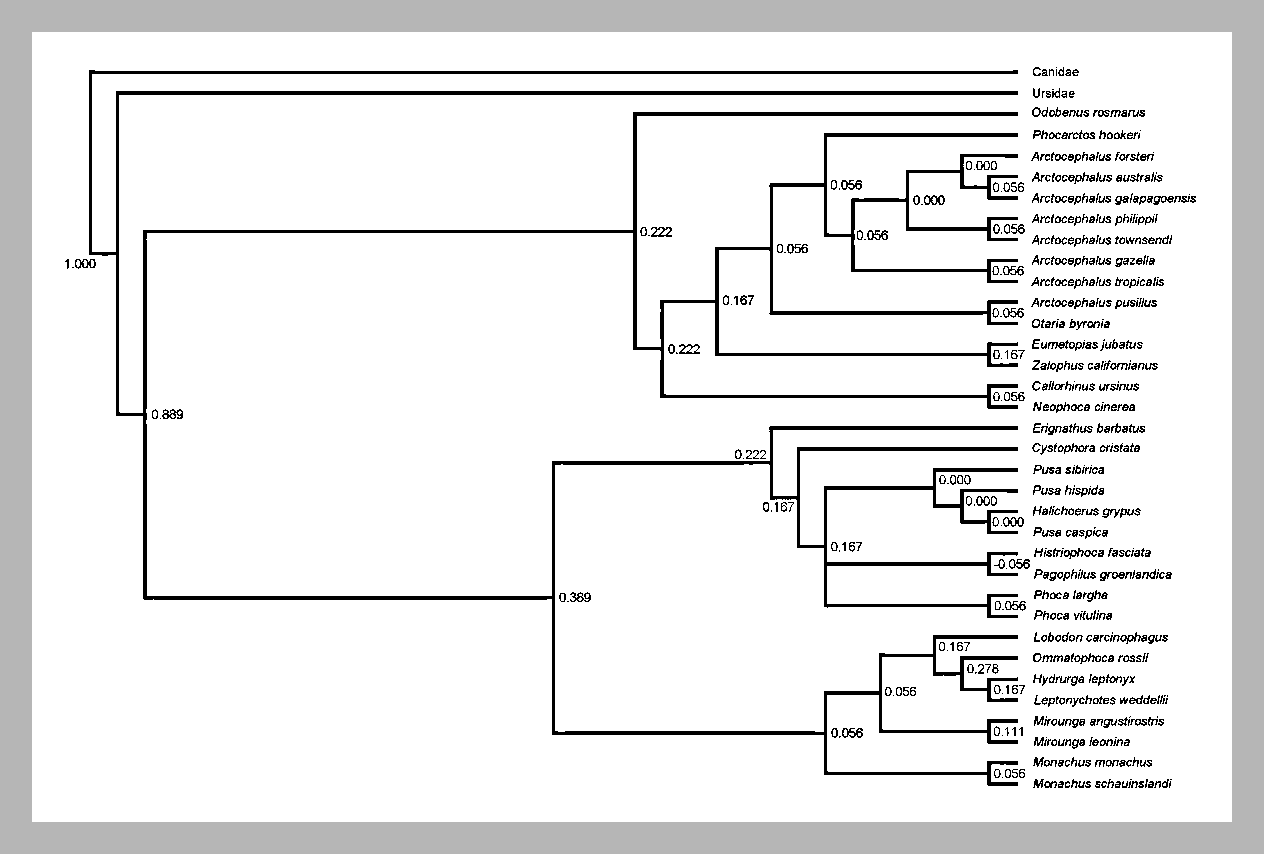

Supplement: Additional file 2 — ZIP files containing several folders, each of which with TreeSnatcher Plus snapshot files, the original image and a text file. [file 1471-2105-13-110-S2.zip › 1471-2148-7-216-2/1471-2148-7-216-2-l_b.PNG]

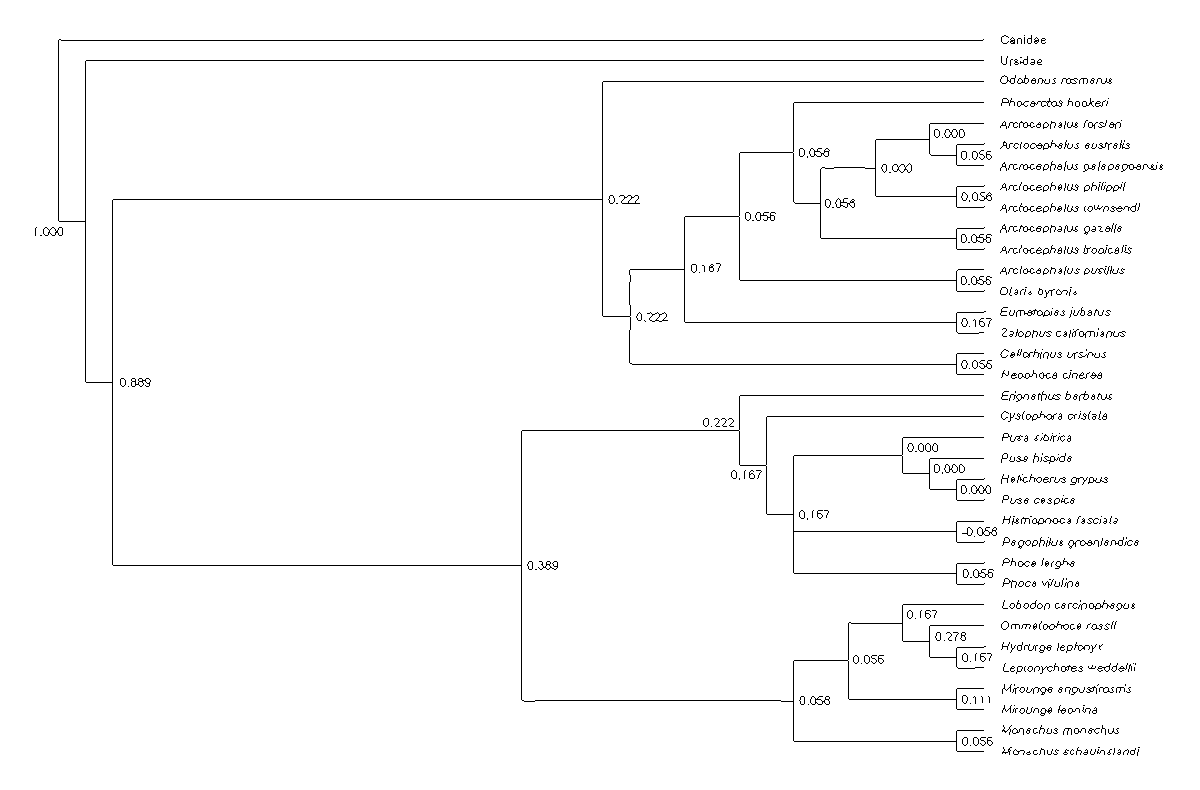

Supplement: Additional file 2 — ZIP files containing several folders, each of which with TreeSnatcher Plus snapshot files, the original image and a text file. [file 1471-2105-13-110-S2.zip › 1471-2148-7-216-2/1471-2148-7-216-2-l_c.PNG]

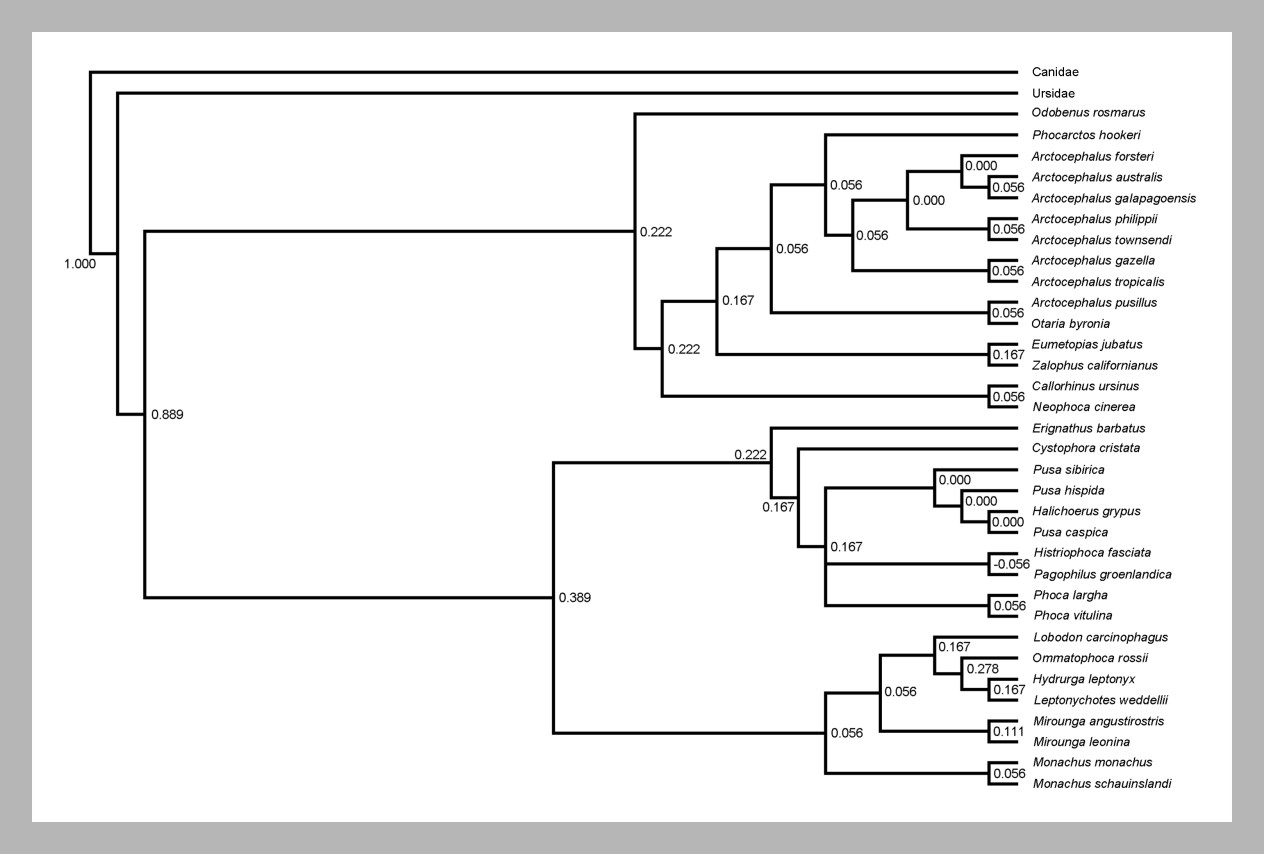

Supplement: Additional file 2 — ZIP files containing several folders, each of which with TreeSnatcher Plus snapshot files, the original image and a text file. [file 1471-2105-13-110-S2.zip › 1471-2148-7-216-2/1471-2148-7-216-2-l_o.PNG]

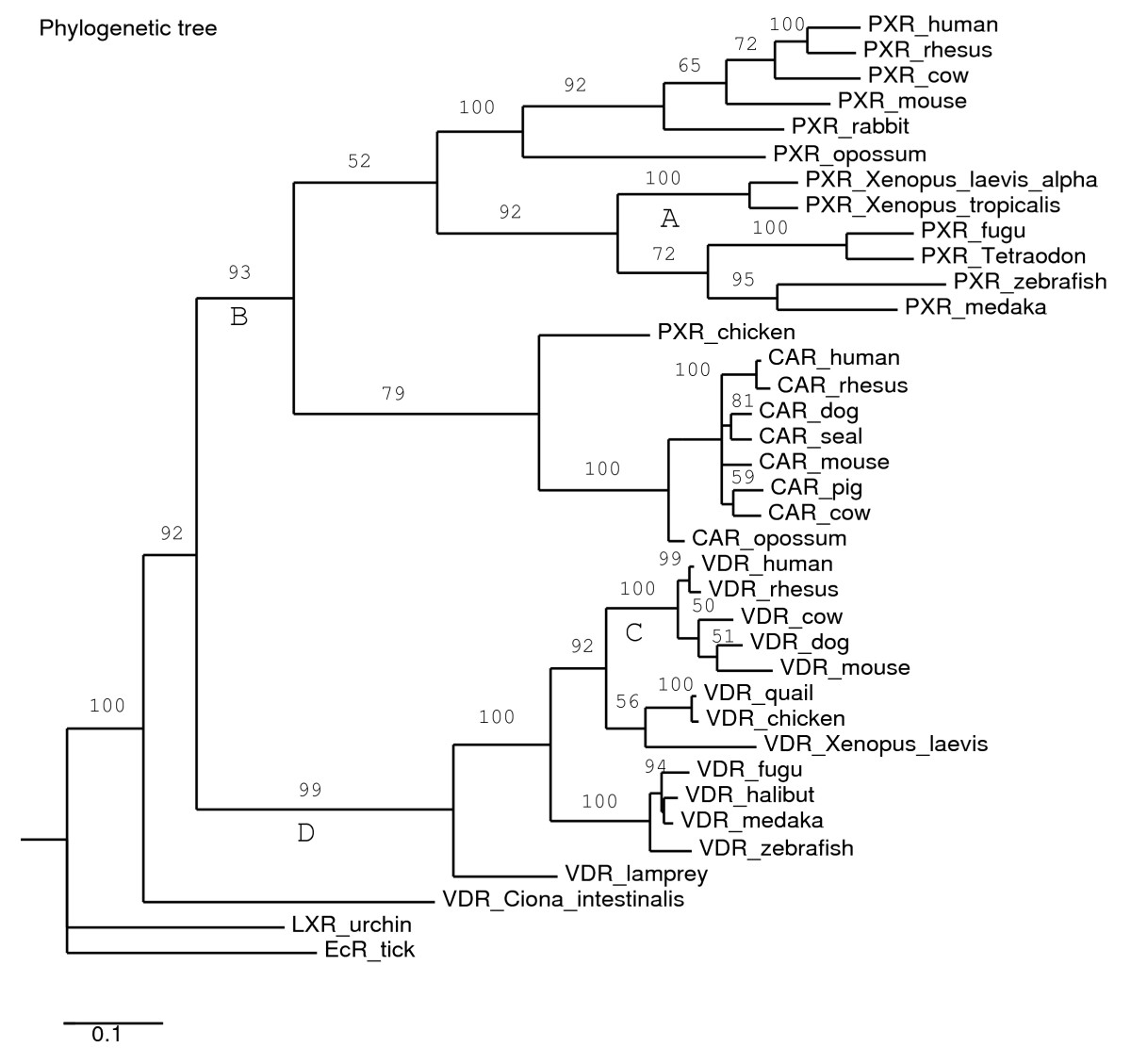

Supplement: Additional file 2 — ZIP files containing several folders, each of which with TreeSnatcher Plus snapshot files, the original image and a text file. [file 1471-2105-13-110-S2.zip › 1471-2148-7-222-4/1471-2148-7-222-4-l.jpg]

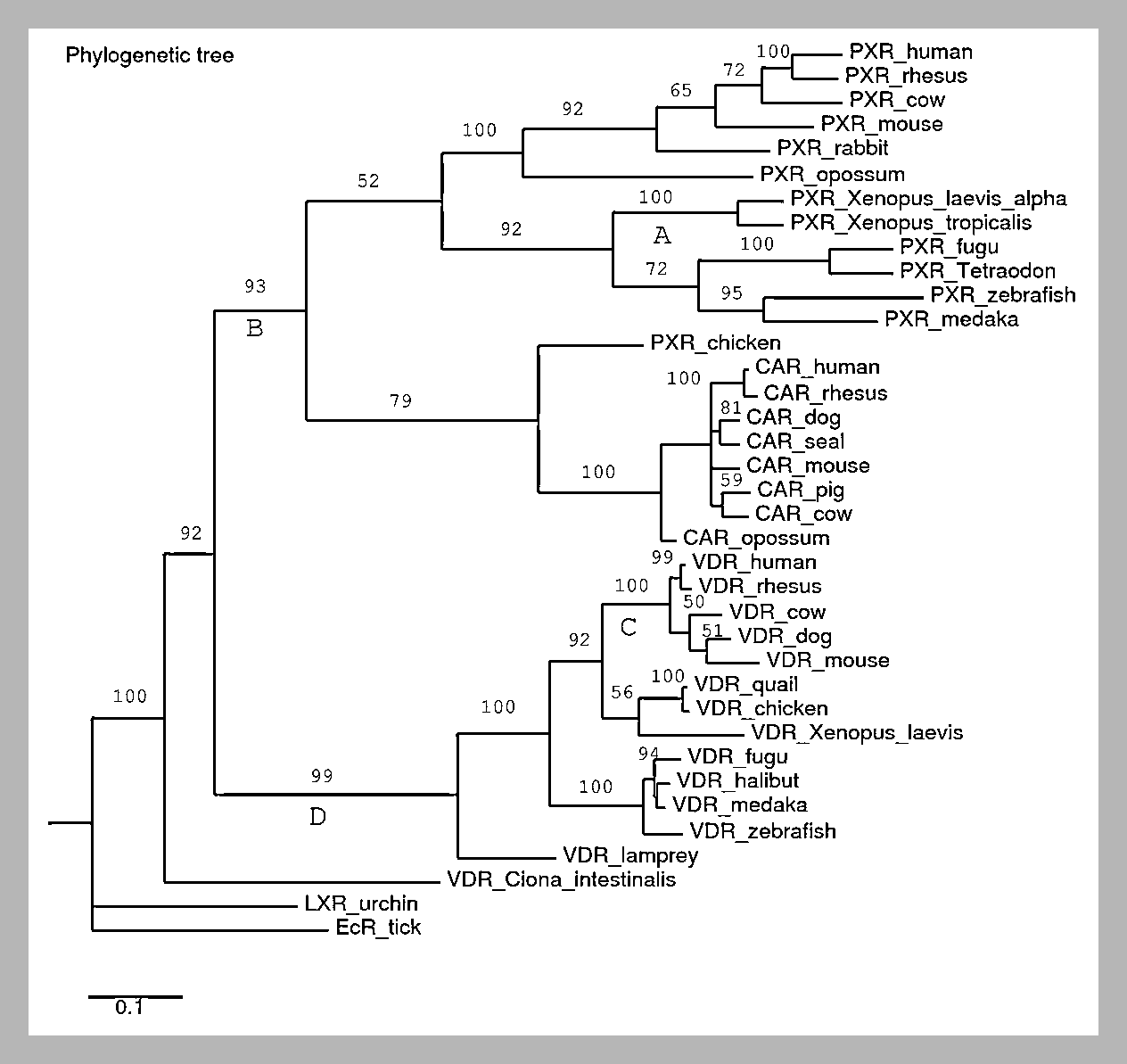

Supplement: Additional file 2 — ZIP files containing several folders, each of which with TreeSnatcher Plus snapshot files, the original image and a text file. [file 1471-2105-13-110-S2.zip › 1471-2148-7-222-4/1471-2148-7-222-4-l_b.PNG]

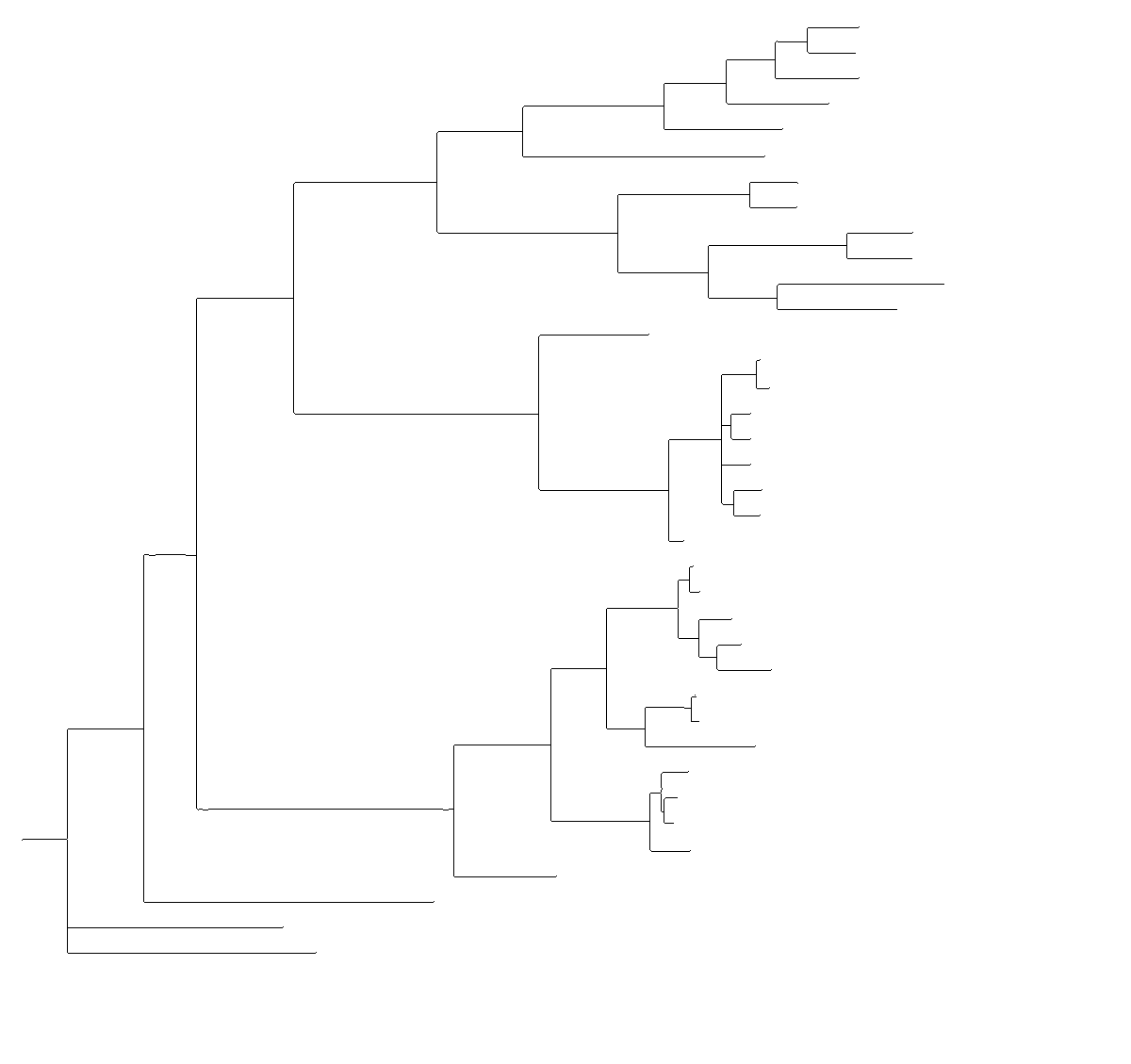

Supplement: Additional file 2 — ZIP files containing several folders, each of which with TreeSnatcher Plus snapshot files, the original image and a text file. [file 1471-2105-13-110-S2.zip › 1471-2148-7-222-4/1471-2148-7-222-4-l_c.PNG]

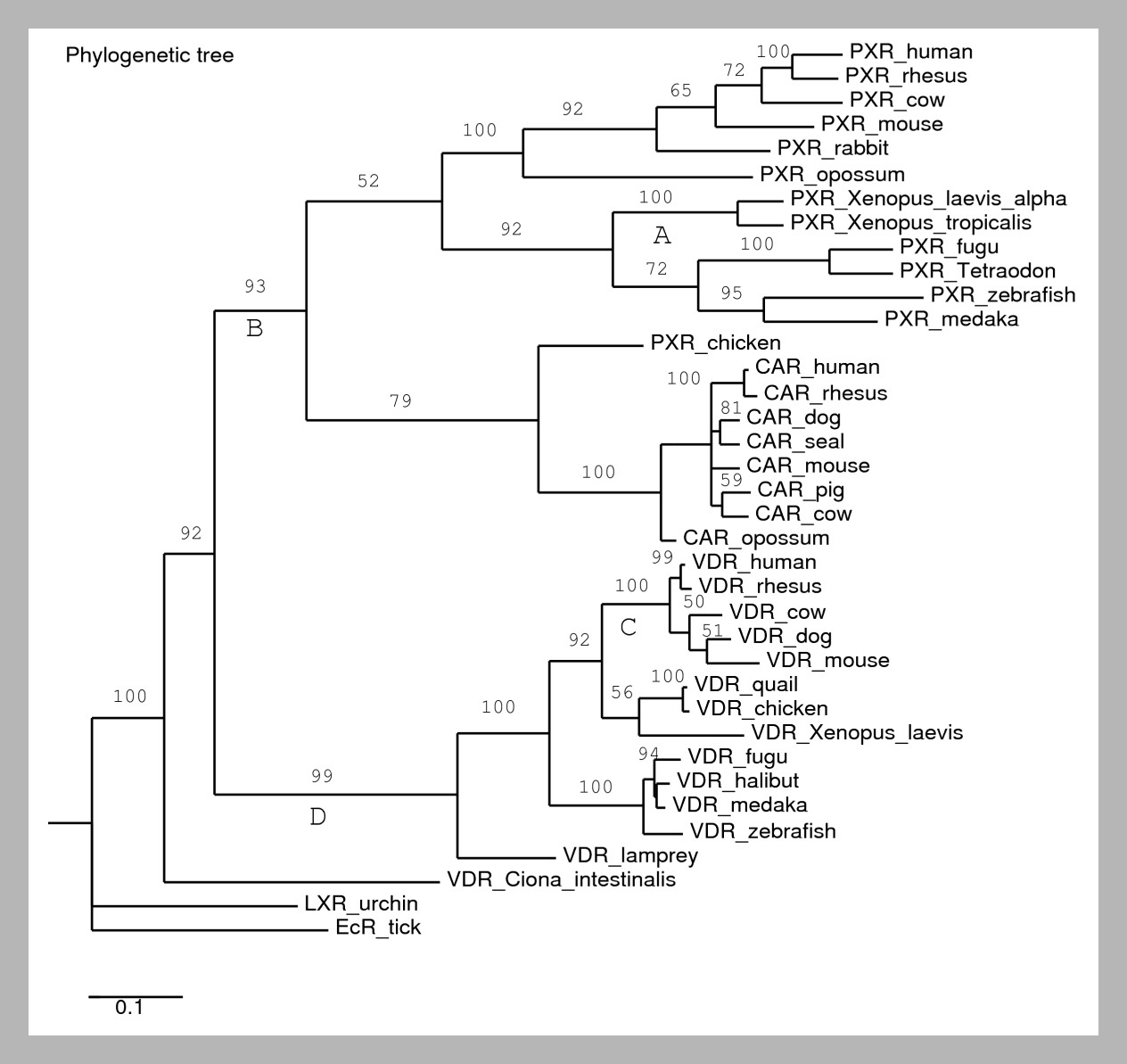

Supplement: Additional file 2 — ZIP files containing several folders, each of which with TreeSnatcher Plus snapshot files, the original image and a text file. [file 1471-2105-13-110-S2.zip › 1471-2148-7-222-4/1471-2148-7-222-4-l_o.PNG]

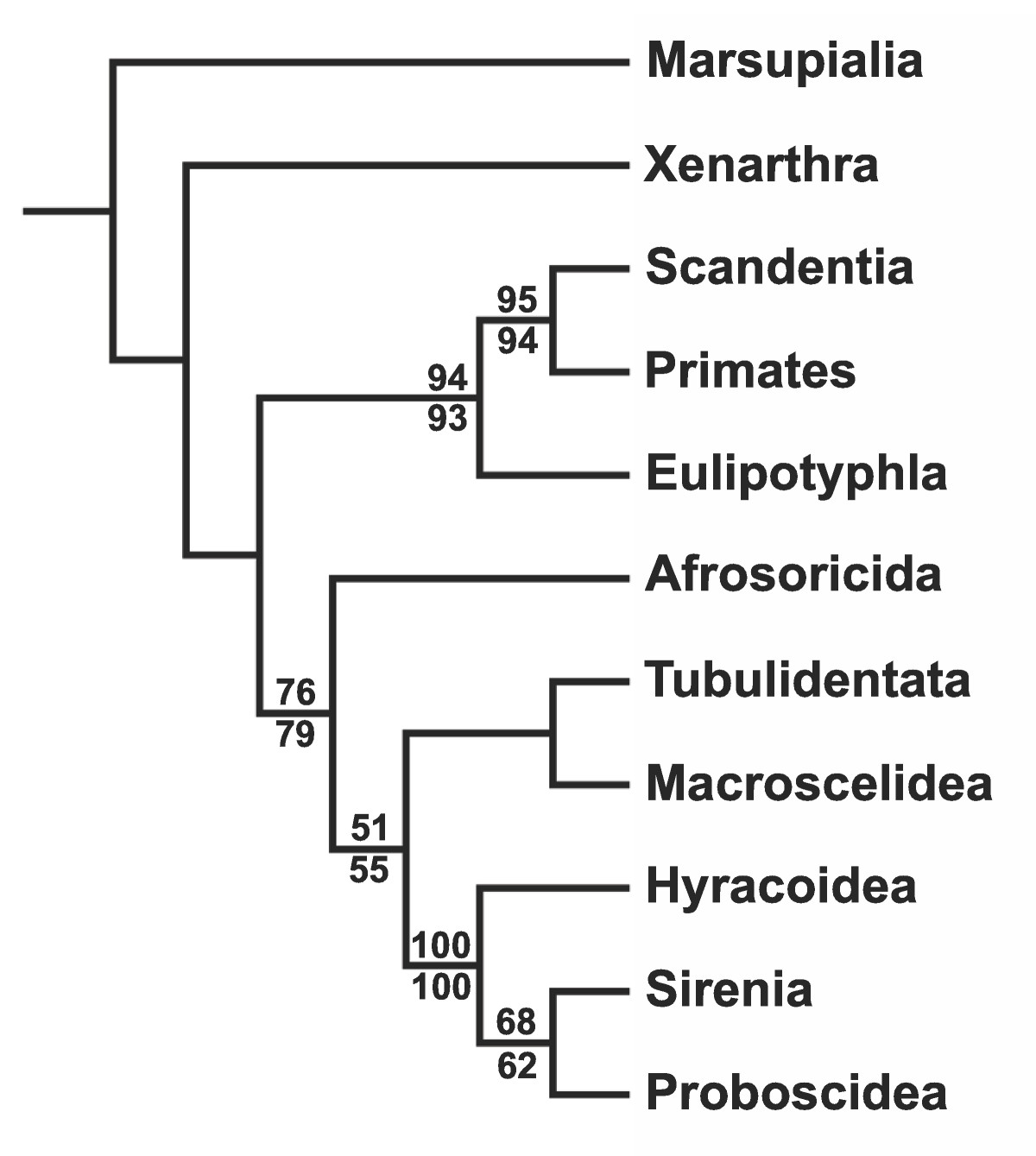

Supplement: Additional file 2 — ZIP files containing several folders, each of which with TreeSnatcher Plus snapshot files, the original image and a text file. [file 1471-2105-13-110-S2.zip › 1471-2148-7-224-1/1471-2148-7-224-1-l.jpg]

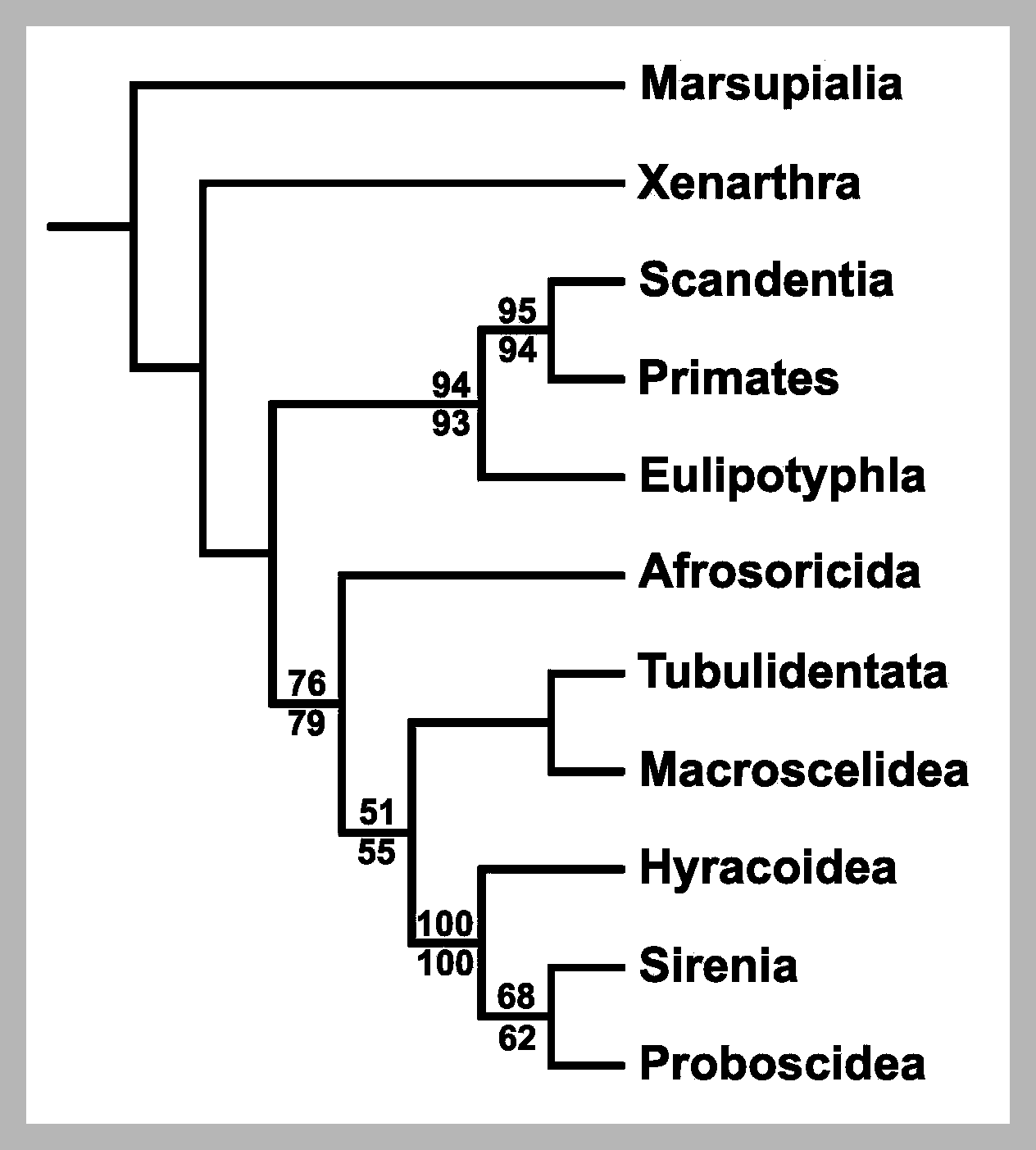

Supplement: Additional file 2 — ZIP files containing several folders, each of which with TreeSnatcher Plus snapshot files, the original image and a text file. [file 1471-2105-13-110-S2.zip › 1471-2148-7-224-1/1471-2148-7-224-1-l_b.PNG]

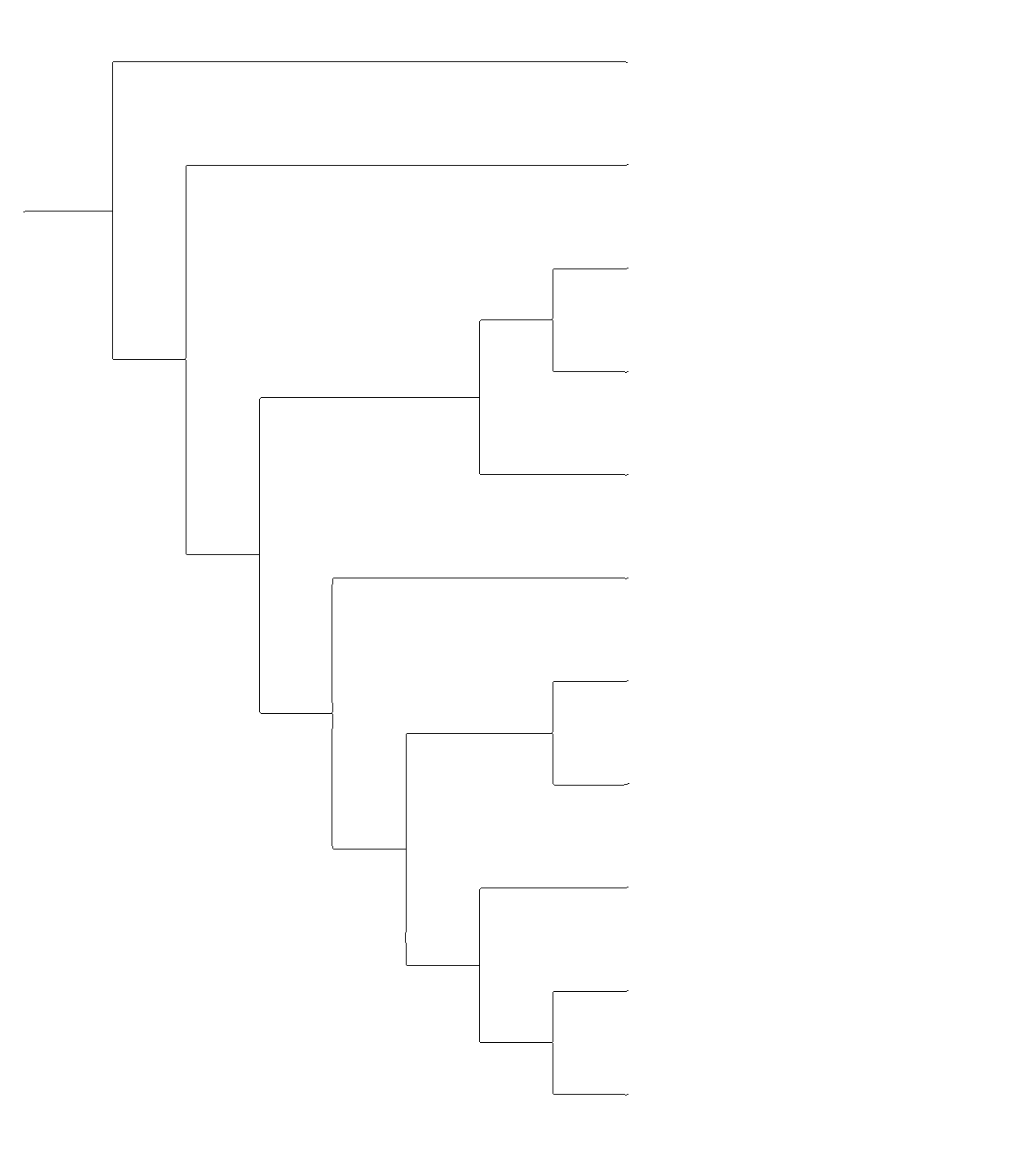

Supplement: Additional file 2 — ZIP files containing several folders, each of which with TreeSnatcher Plus snapshot files, the original image and a text file. [file 1471-2105-13-110-S2.zip › 1471-2148-7-224-1/1471-2148-7-224-1-l_c.PNG]

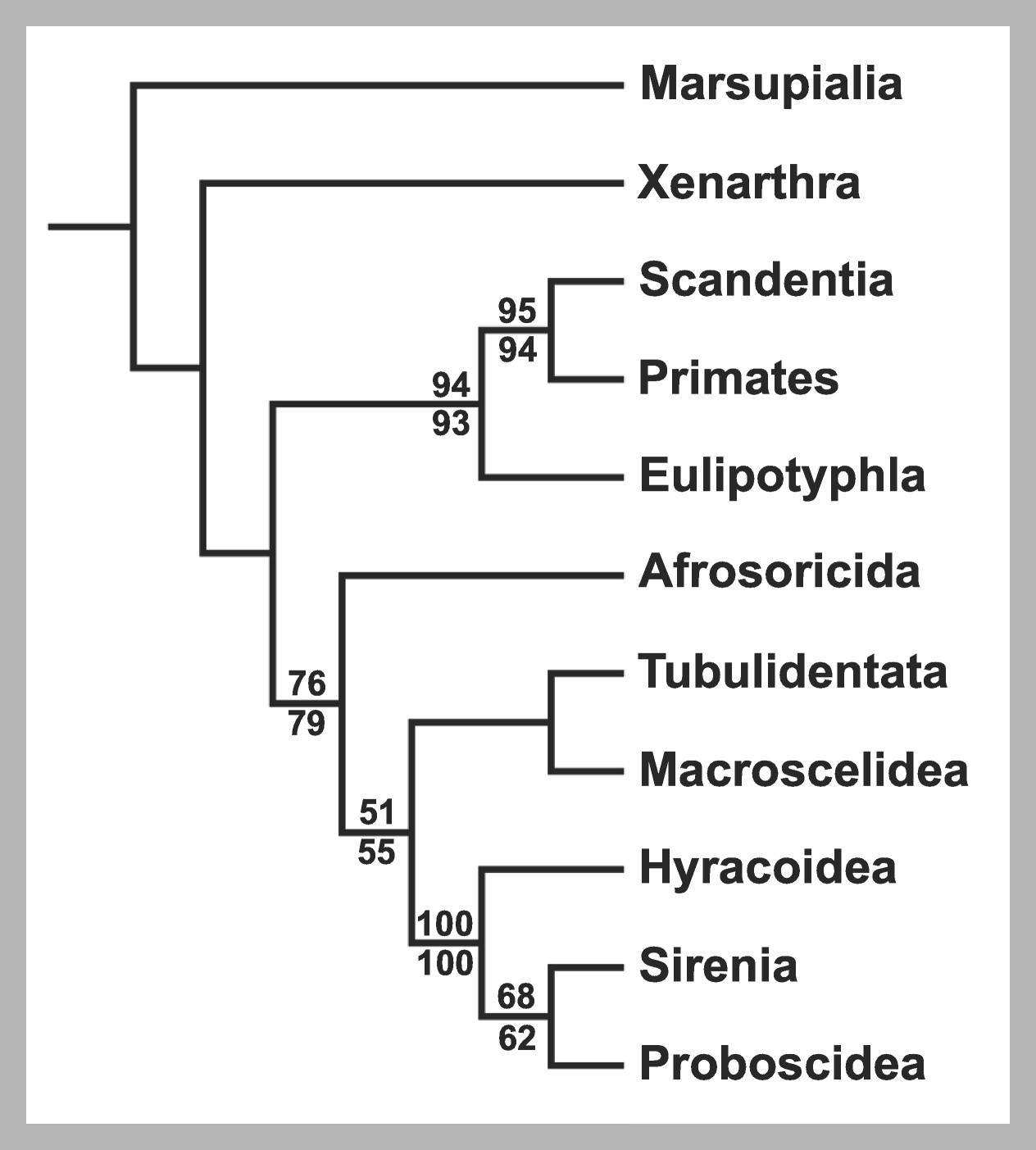

Supplement: Additional file 2 — ZIP files containing several folders, each of which with TreeSnatcher Plus snapshot files, the original image and a text file. [file 1471-2105-13-110-S2.zip › 1471-2148-7-224-1/1471-2148-7-224-1-l_o.PNG]

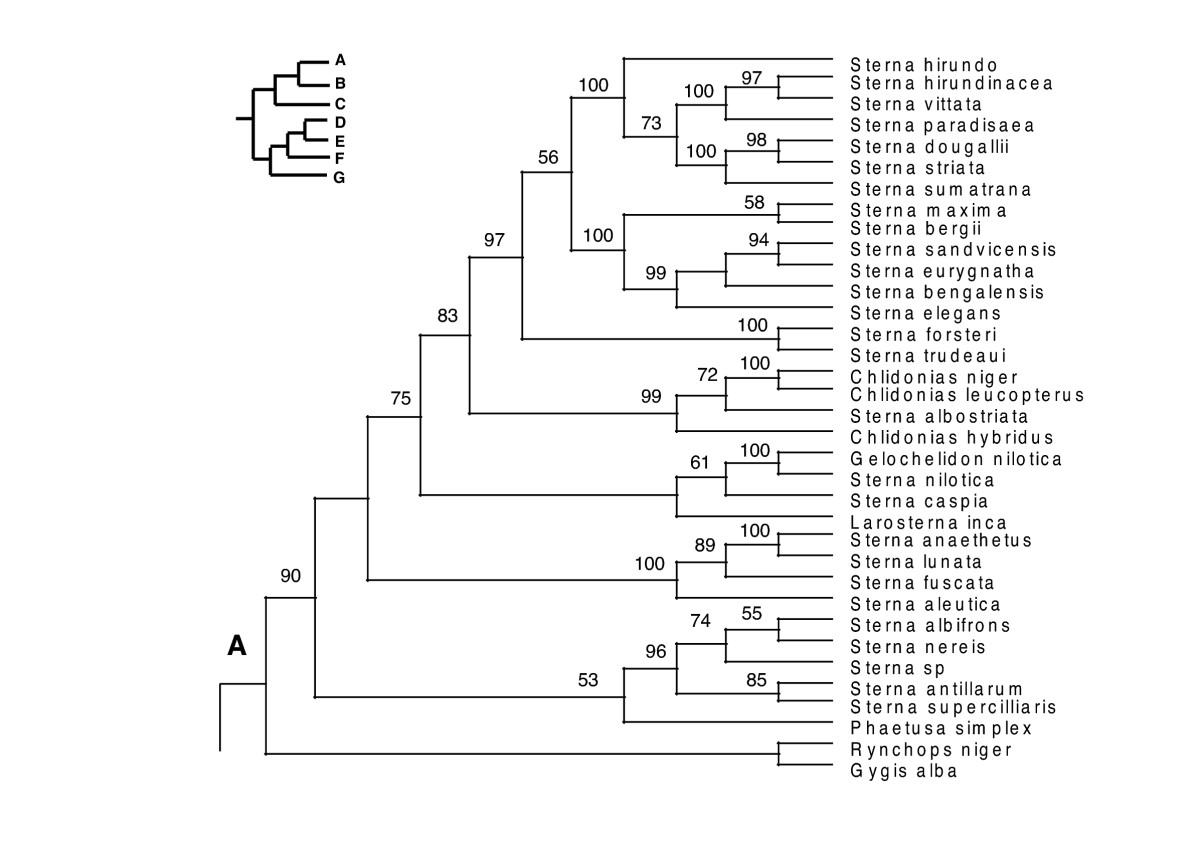

Supplement: Additional file 2 — ZIP files containing several folders, each of which with TreeSnatcher Plus snapshot files, the original image and a text file. [file 1471-2105-13-110-S2.zip › 1471-2148-7-227-2/1471-2148-7-227-2-l.jpg]

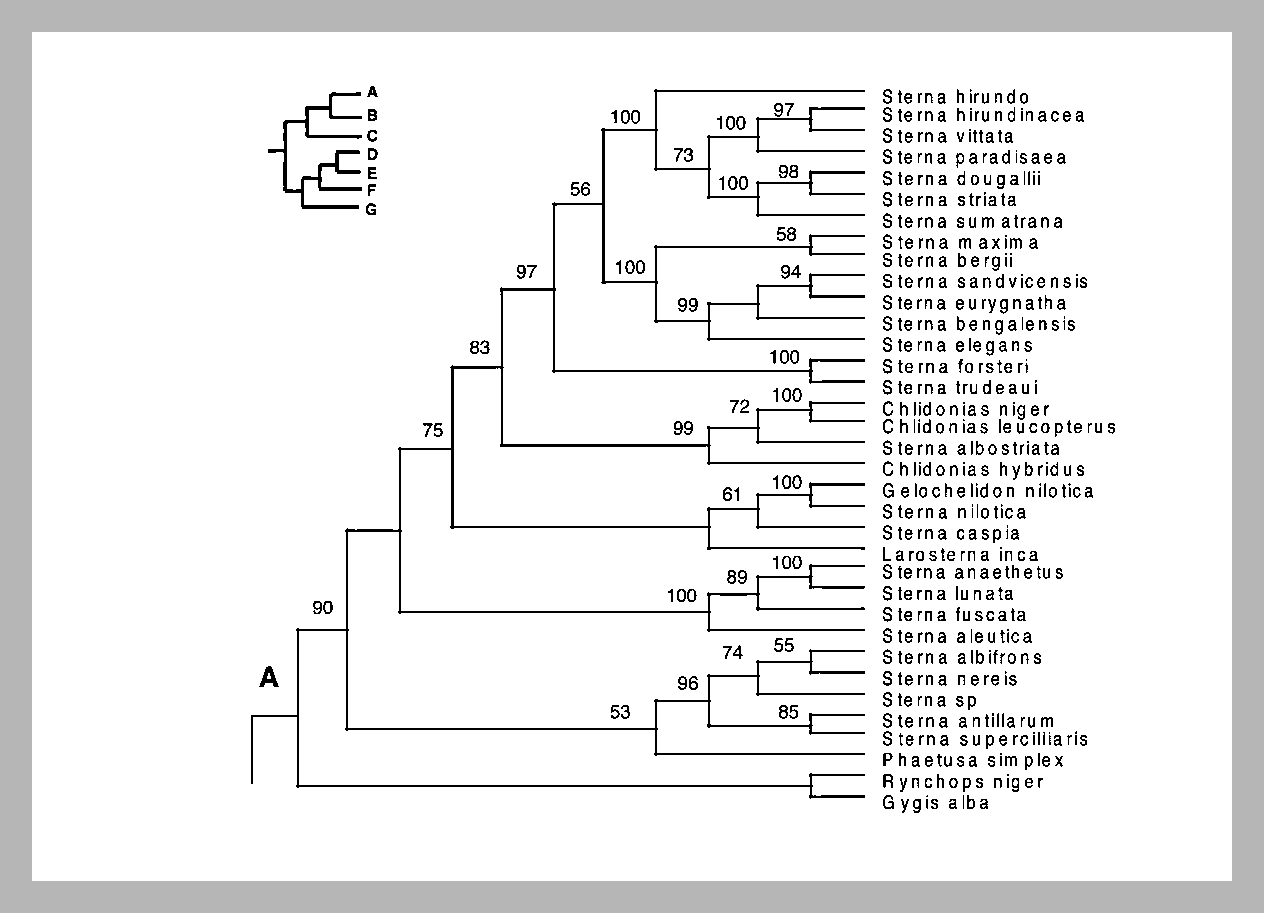

Supplement: Additional file 2 — ZIP files containing several folders, each of which with TreeSnatcher Plus snapshot files, the original image and a text file. [file 1471-2105-13-110-S2.zip › 1471-2148-7-227-2/1471-2148-7-227-2-l_b.PNG]

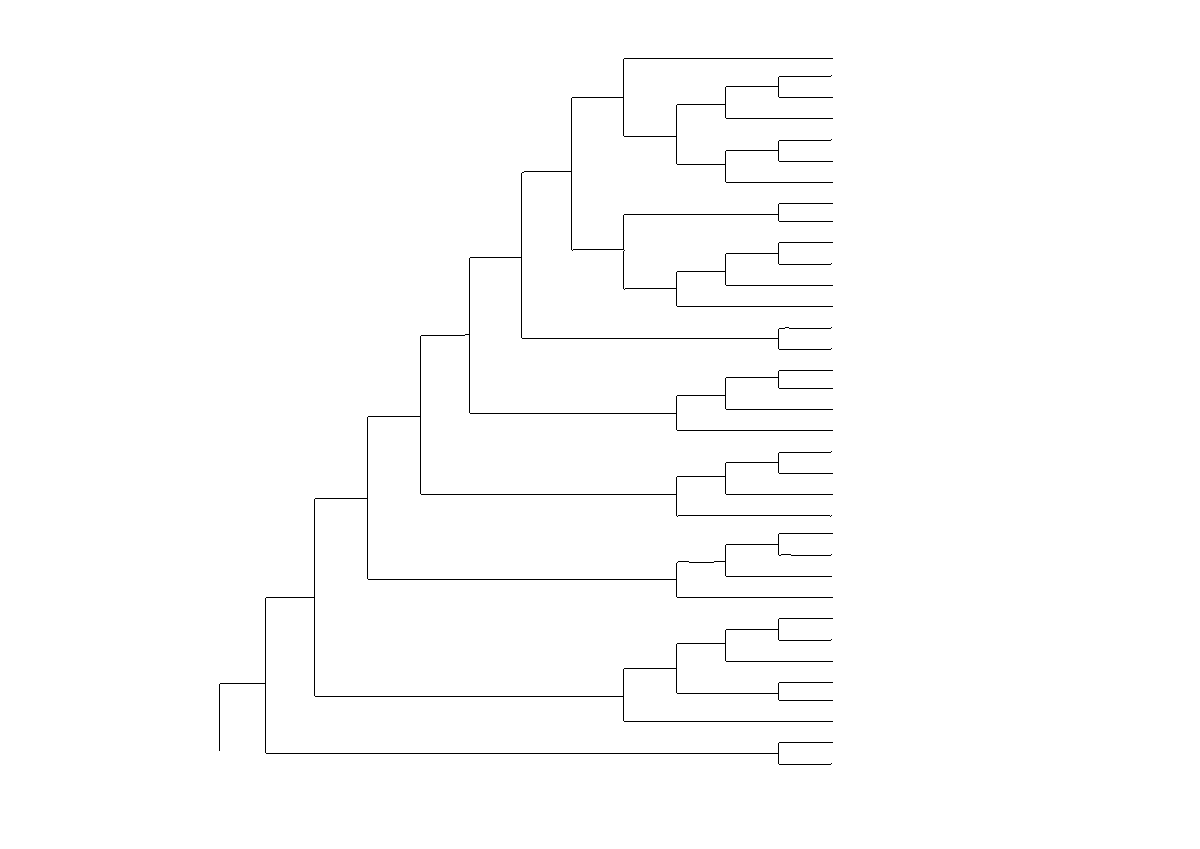

Supplement: Additional file 2 — ZIP files containing several folders, each of which with TreeSnatcher Plus snapshot files, the original image and a text file. [file 1471-2105-13-110-S2.zip › 1471-2148-7-227-2/1471-2148-7-227-2-l_c.PNG]

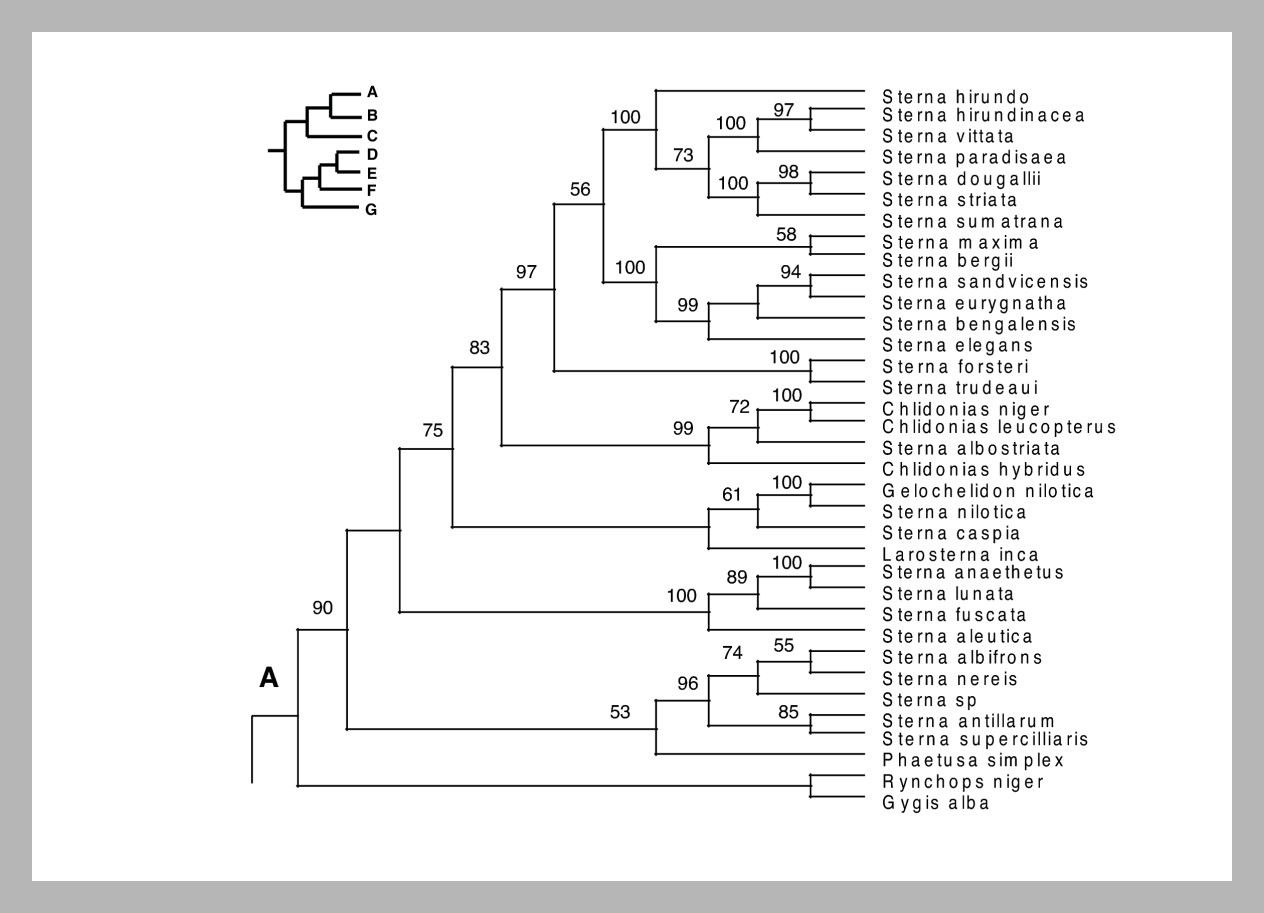

Supplement: Additional file 2 — ZIP files containing several folders, each of which with TreeSnatcher Plus snapshot files, the original image and a text file. [file 1471-2105-13-110-S2.zip › 1471-2148-7-227-2/1471-2148-7-227-2-l_o.PNG]

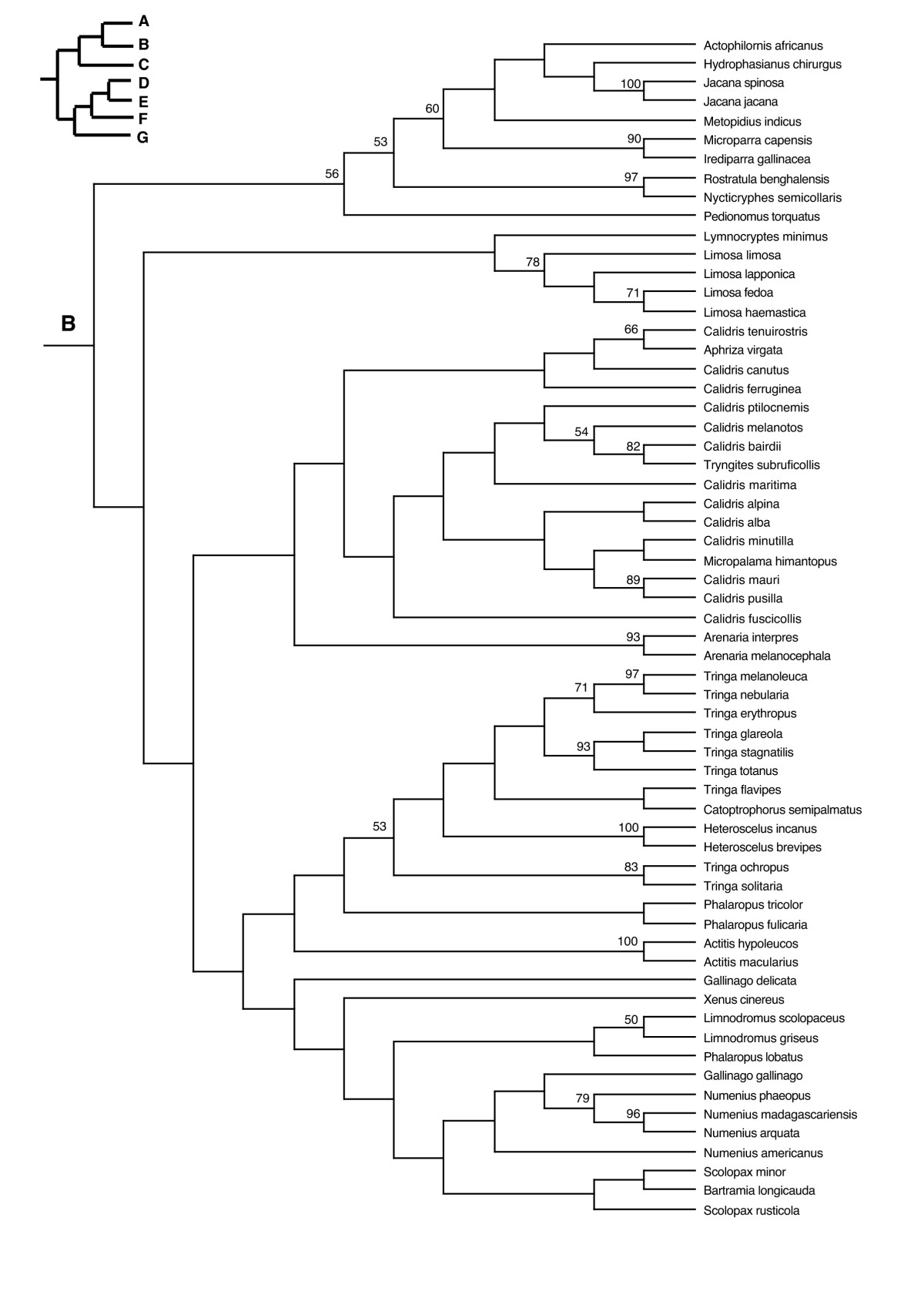

Supplement: Additional file 2 — ZIP files containing several folders, each of which with TreeSnatcher Plus snapshot files, the original image and a text file. [file 1471-2105-13-110-S2.zip › 1471-2148-7-227-3/1471-2148-7-227-3-l.jpg]

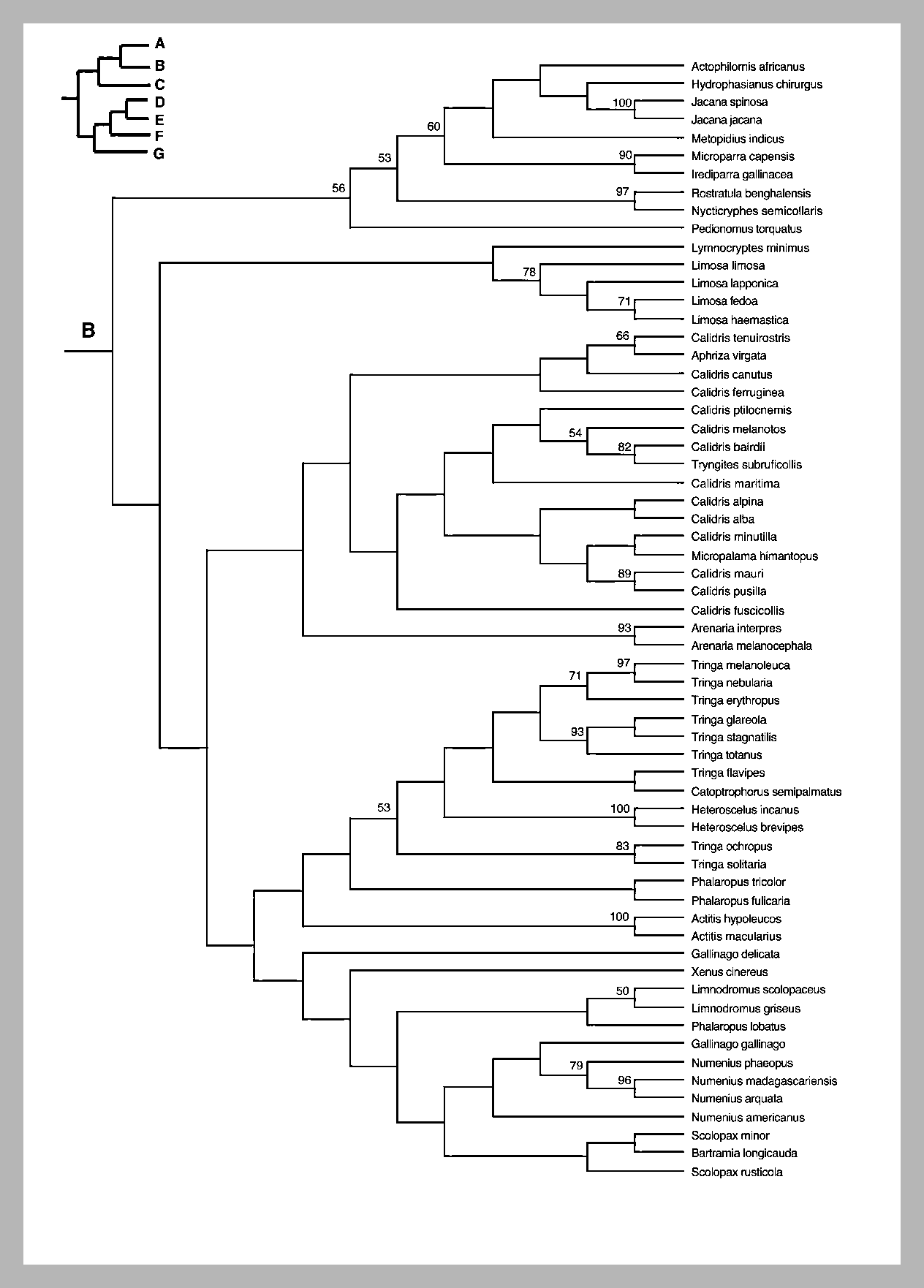

Supplement: Additional file 2 — ZIP files containing several folders, each of which with TreeSnatcher Plus snapshot files, the original image and a text file. [file 1471-2105-13-110-S2.zip › 1471-2148-7-227-3/1471-2148-7-227-3-l_b.PNG]

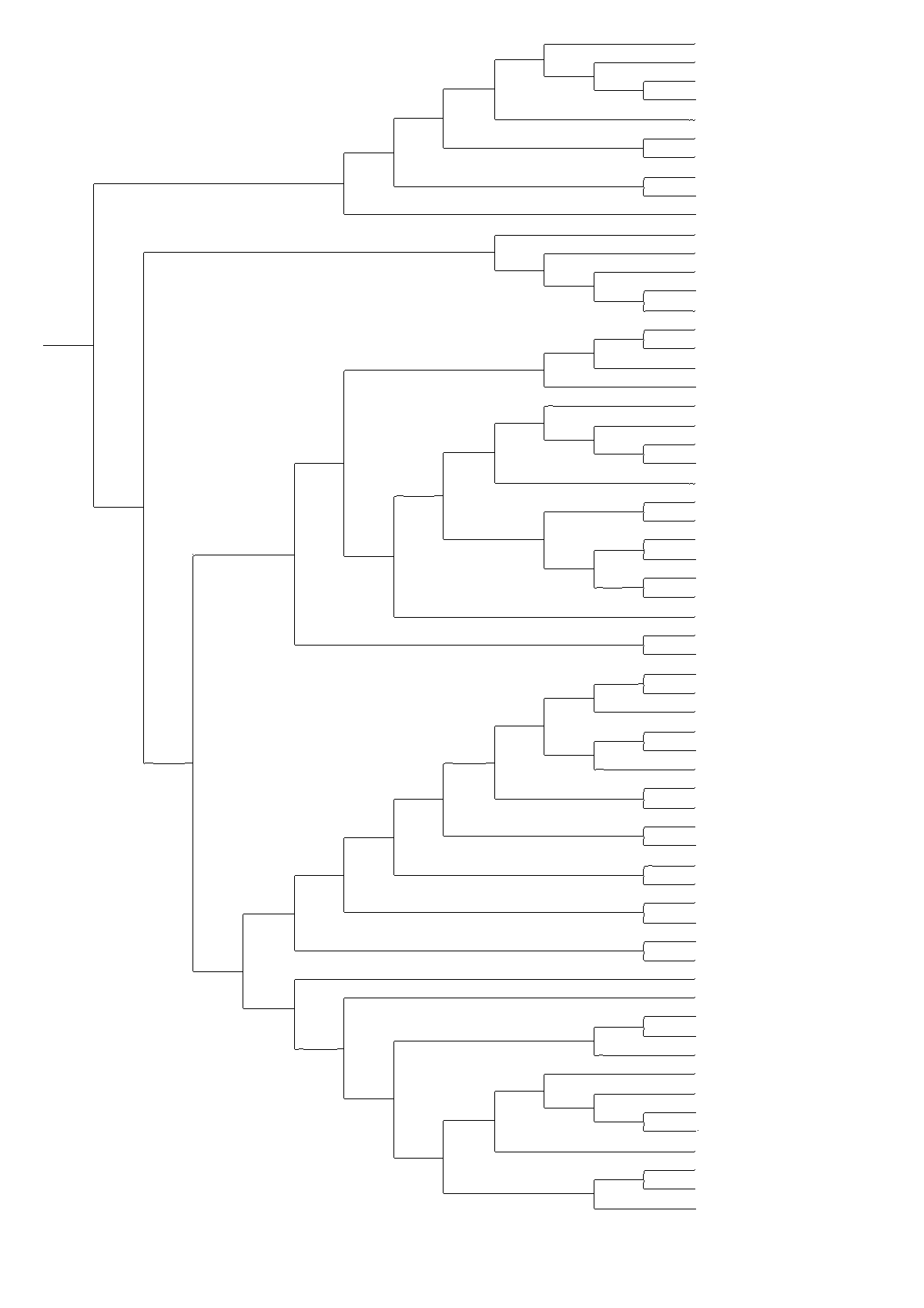

Supplement: Additional file 2 — ZIP files containing several folders, each of which with TreeSnatcher Plus snapshot files, the original image and a text file. [file 1471-2105-13-110-S2.zip › 1471-2148-7-227-3/1471-2148-7-227-3-l_c.PNG]

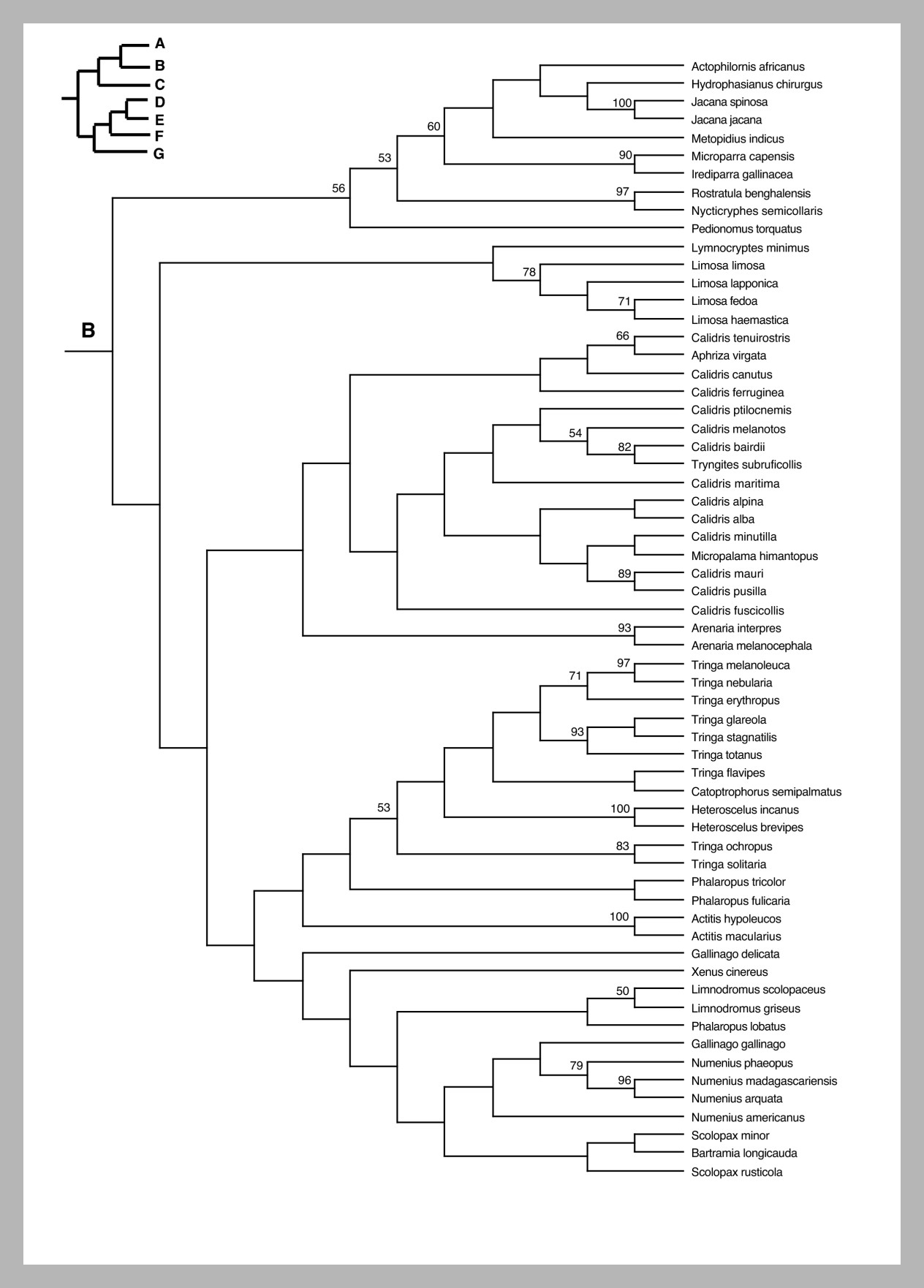

Supplement: Additional file 2 — ZIP files containing several folders, each of which with TreeSnatcher Plus snapshot files, the original image and a text file. [file 1471-2105-13-110-S2.zip › 1471-2148-7-227-3/1471-2148-7-227-3-l_o.PNG]

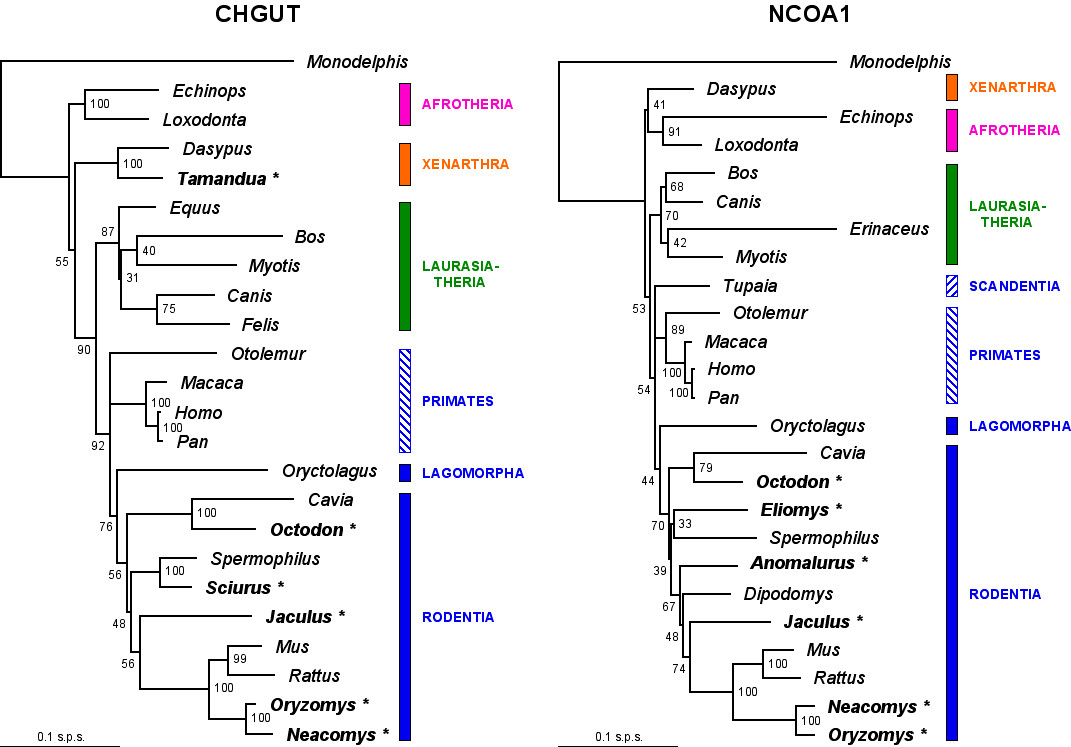

Supplement: Additional file 2 — ZIP files containing several folders, each of which with TreeSnatcher Plus snapshot files, the original image and a text file. [file 1471-2105-13-110-S2.zip › 1471-2148-7-241-6/1471-2148-7-241-6-l.jpg]

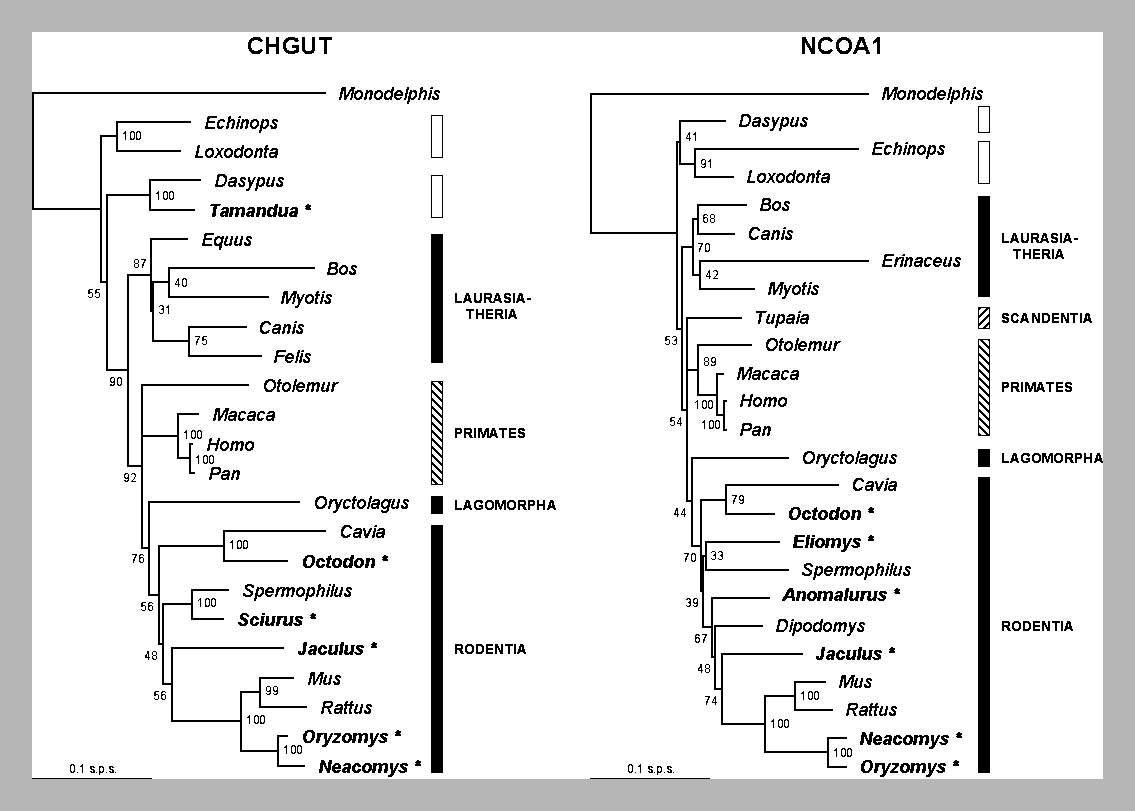

Supplement: Additional file 2 — ZIP files containing several folders, each of which with TreeSnatcher Plus snapshot files, the original image and a text file. [file 1471-2105-13-110-S2.zip › 1471-2148-7-241-6/1471-2148-7-241-6-l_b.PNG]

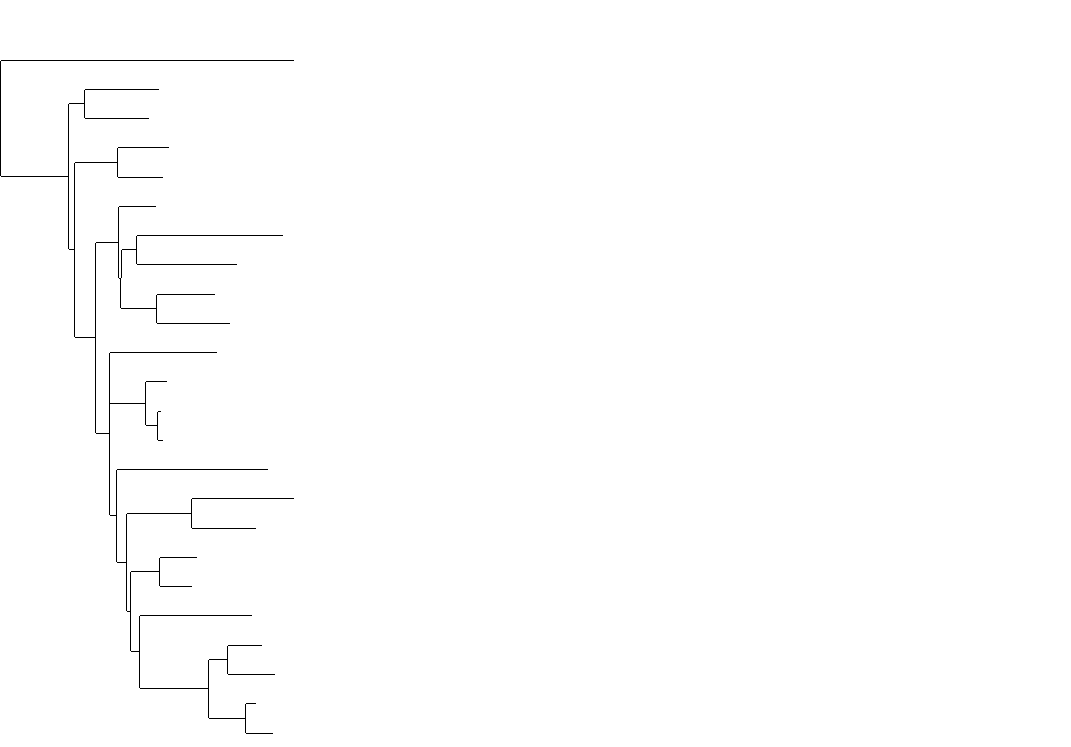

Supplement: Additional file 2 — ZIP files containing several folders, each of which with TreeSnatcher Plus snapshot files, the original image and a text file. [file 1471-2105-13-110-S2.zip › 1471-2148-7-241-6/1471-2148-7-241-6-l_c.PNG]

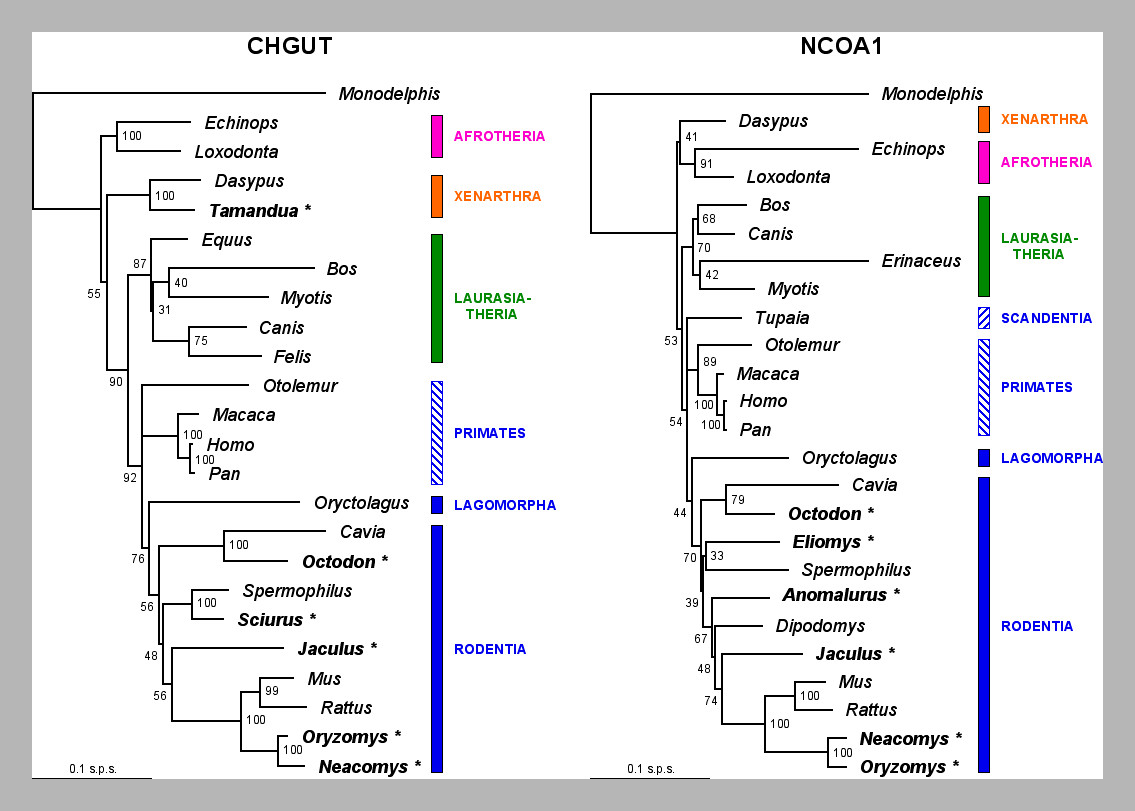

Supplement: Additional file 2 — ZIP files containing several folders, each of which with TreeSnatcher Plus snapshot files, the original image and a text file. [file 1471-2105-13-110-S2.zip › 1471-2148-7-241-6/1471-2148-7-241-6-l_o.PNG]

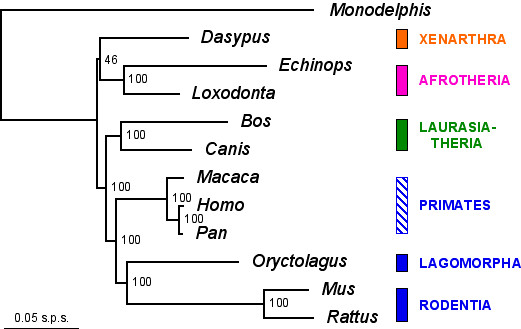

Supplement: Additional file 2 — ZIP files containing several folders, each of which with TreeSnatcher Plus snapshot files, the original image and a text file. [file 1471-2105-13-110-S2.zip › 1471-2148-7-241-7/1471-2148-7-241-7-l.jpg]

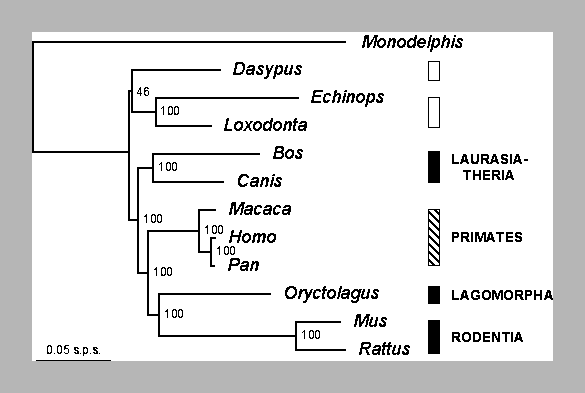

Supplement: Additional file 2 — ZIP files containing several folders, each of which with TreeSnatcher Plus snapshot files, the original image and a text file. [file 1471-2105-13-110-S2.zip › 1471-2148-7-241-7/1471-2148-7-241-7-l_b.PNG]

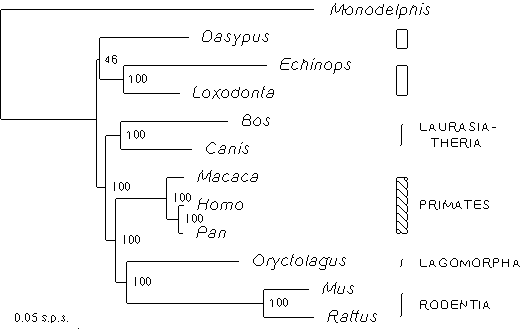

Supplement: Additional file 2 — ZIP files containing several folders, each of which with TreeSnatcher Plus snapshot files, the original image and a text file. [file 1471-2105-13-110-S2.zip › 1471-2148-7-241-7/1471-2148-7-241-7-l_c.PNG]

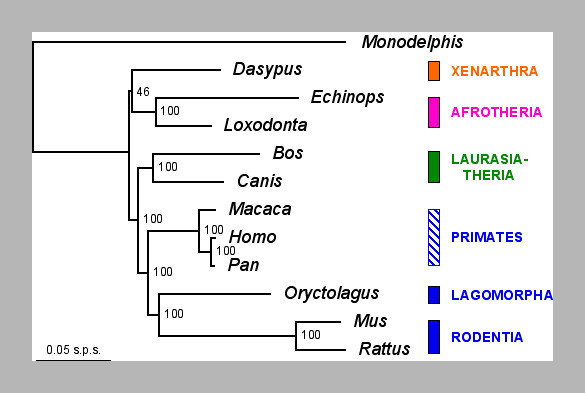

Supplement: Additional file 2 — ZIP files containing several folders, each of which with TreeSnatcher Plus snapshot files, the original image and a text file. [file 1471-2105-13-110-S2.zip › 1471-2148-7-241-7/1471-2148-7-241-7-l_o.PNG]

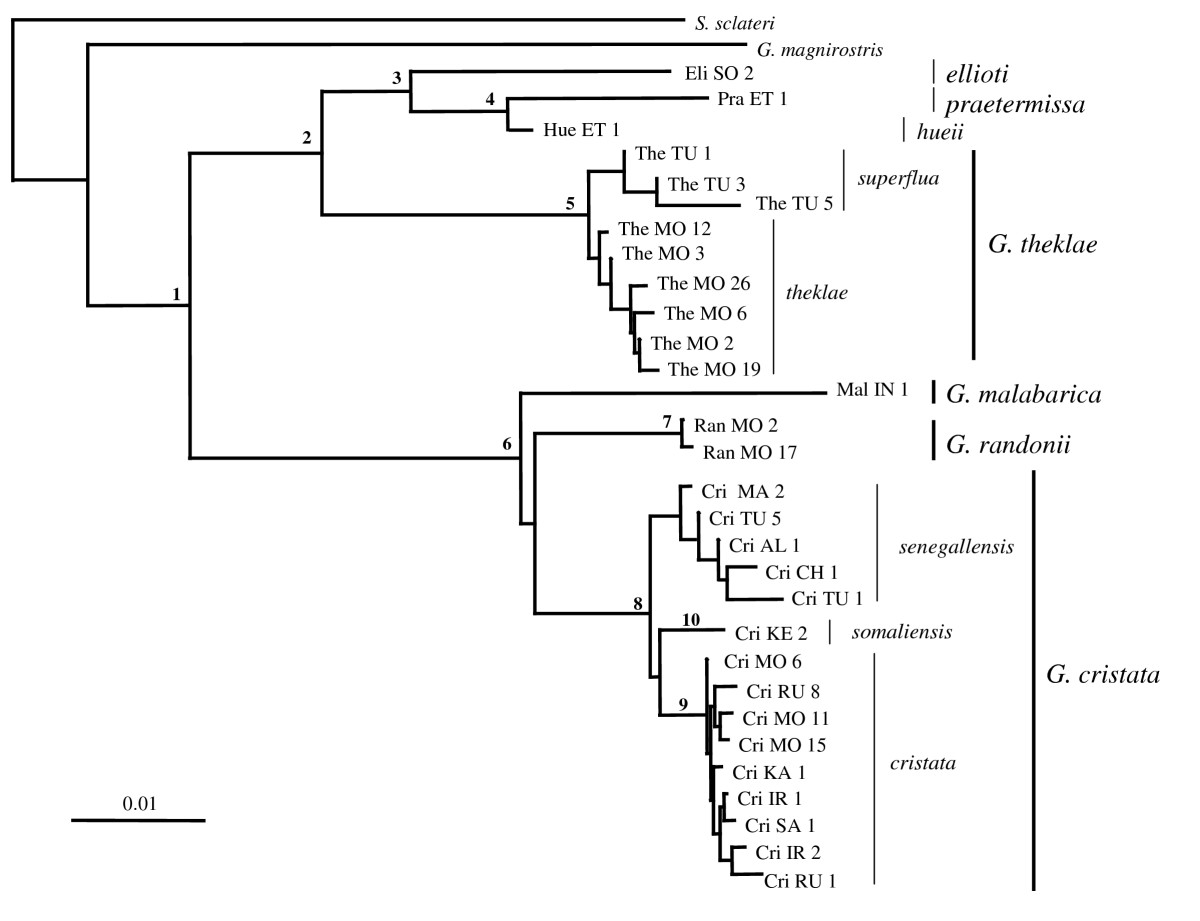

Supplement: Additional file 2 — ZIP files containing several folders, each of which with TreeSnatcher Plus snapshot files, the original image and a text file. [file 1471-2105-13-110-S2.zip › 1471-2148-8-32-2/1471-2148-8-32-2-l.jpg]

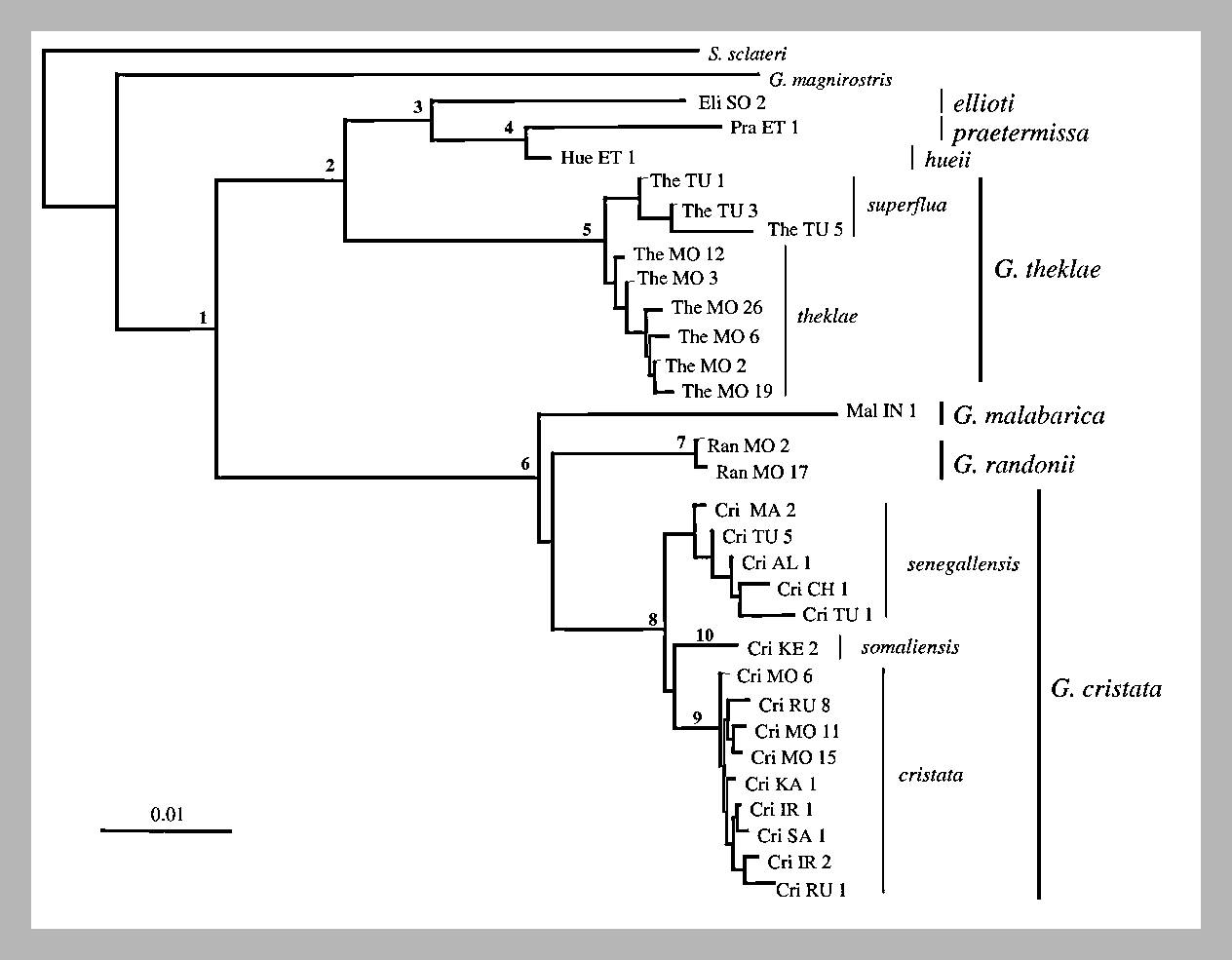

Supplement: Additional file 2 — ZIP files containing several folders, each of which with TreeSnatcher Plus snapshot files, the original image and a text file. [file 1471-2105-13-110-S2.zip › 1471-2148-8-32-2/1471-2148-8-32-2_b.PNG]

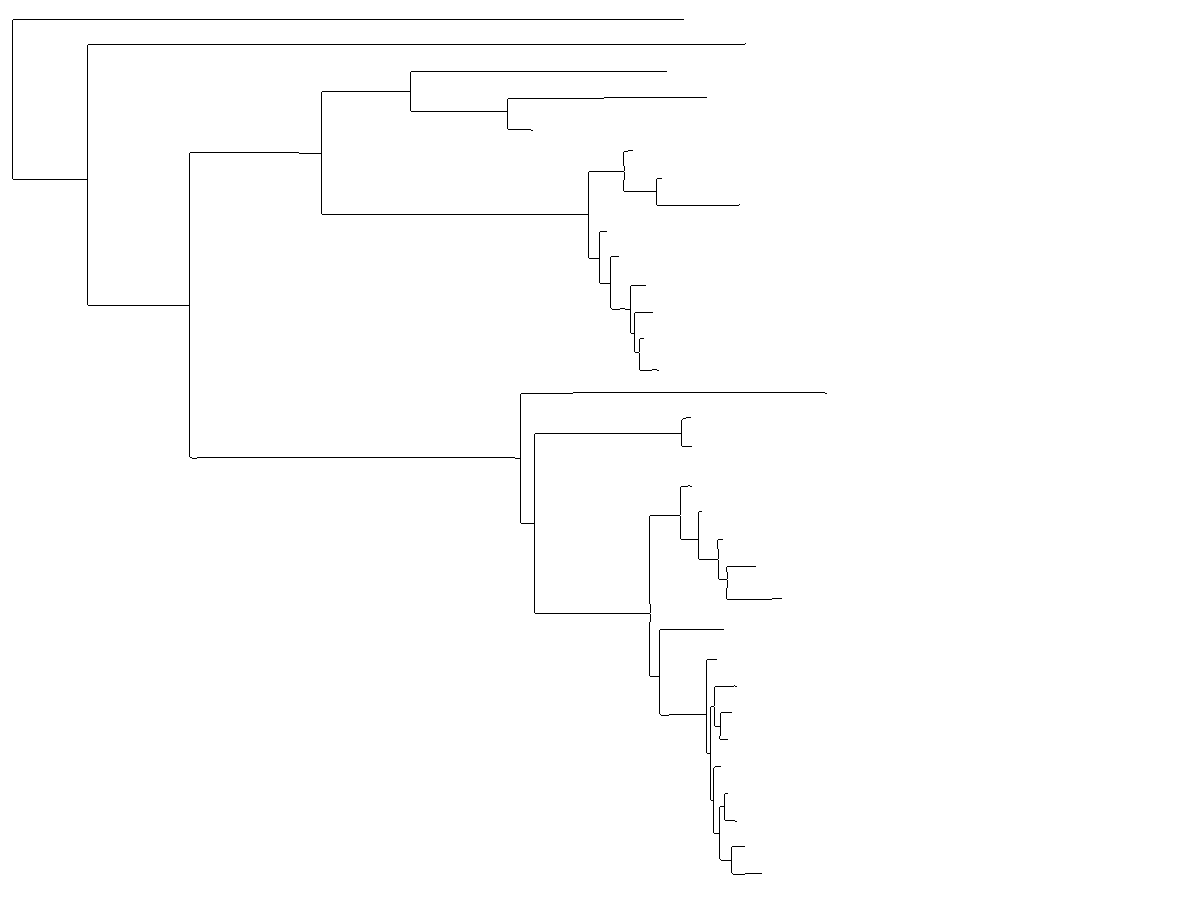

Supplement: Additional file 2 — ZIP files containing several folders, each of which with TreeSnatcher Plus snapshot files, the original image and a text file. [file 1471-2105-13-110-S2.zip › 1471-2148-8-32-2/1471-2148-8-32-2_c.PNG]

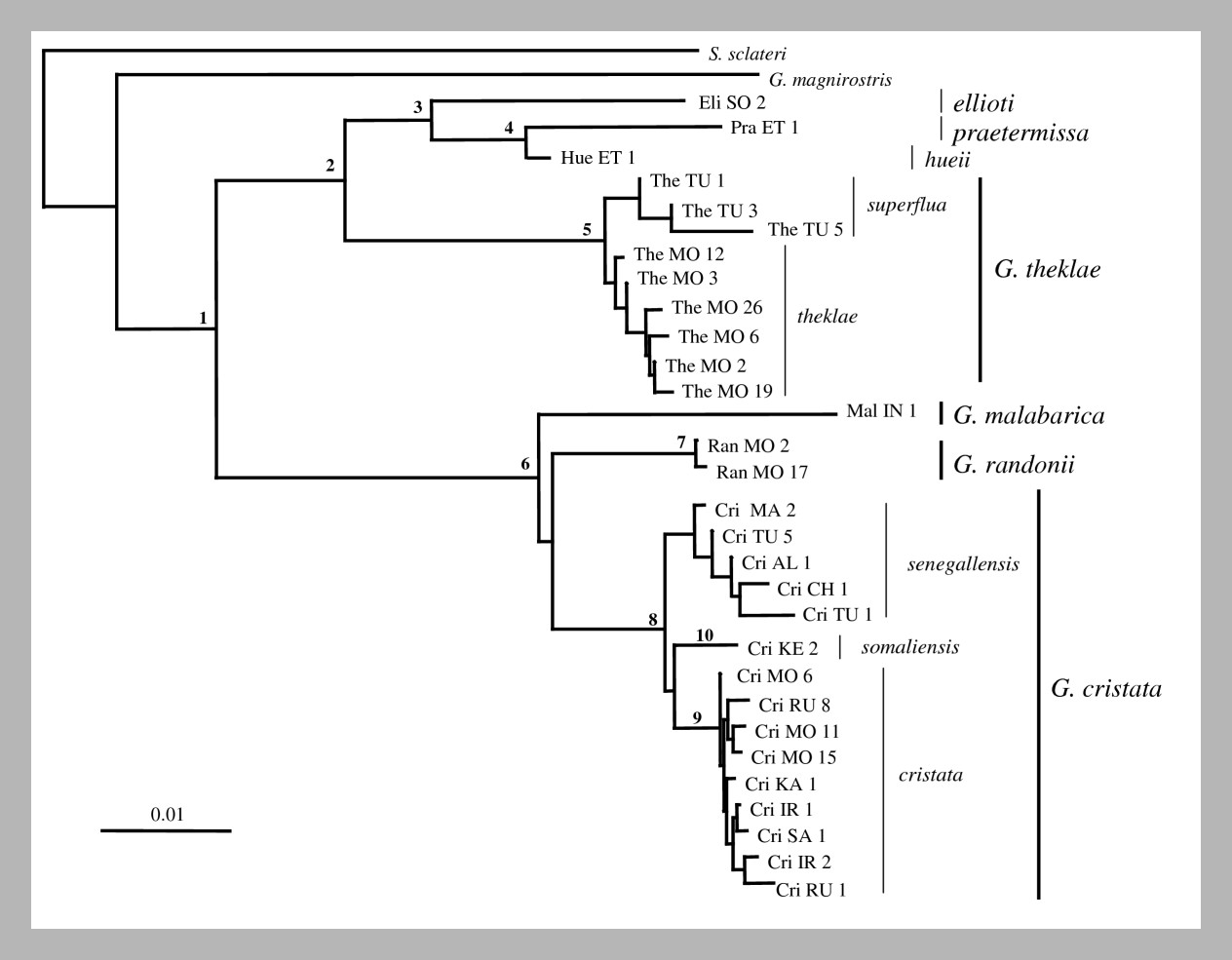

Supplement: Additional file 2 — ZIP files containing several folders, each of which with TreeSnatcher Plus snapshot files, the original image and a text file. [file 1471-2105-13-110-S2.zip › 1471-2148-8-32-2/1471-2148-8-32-2_o.PNG]

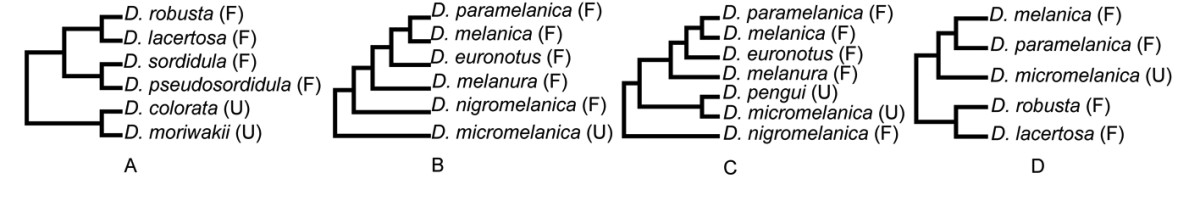

Supplement: Additional file 2 — ZIP files containing several folders, each of which with TreeSnatcher Plus snapshot files, the original image and a text file. [file 1471-2105-13-110-S2.zip › 1471-2148-8-33-1/1471-2148-8-33-1-l.jpg]

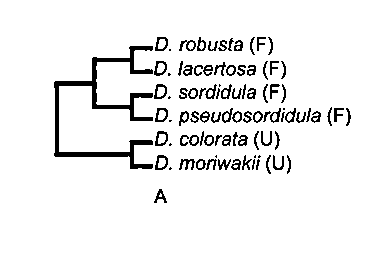

Supplement: Additional file 2 — ZIP files containing several folders, each of which with TreeSnatcher Plus snapshot files, the original image and a text file. [file 1471-2105-13-110-S2.zip › 1471-2148-8-33-1/1471-2148-8-33-1-l_b.PNG]

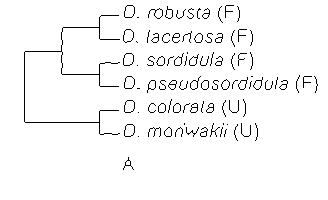

Supplement: Additional file 2 — ZIP files containing several folders, each of which with TreeSnatcher Plus snapshot files, the original image and a text file. [file 1471-2105-13-110-S2.zip › 1471-2148-8-33-1/1471-2148-8-33-1-l_c.PNG]

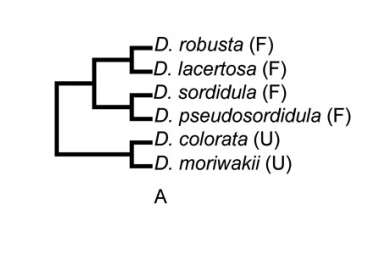

Supplement: Additional file 2 — ZIP files containing several folders, each of which with TreeSnatcher Plus snapshot files, the original image and a text file. [file 1471-2105-13-110-S2.zip › 1471-2148-8-33-1/1471-2148-8-33-1-l_o.PNG]

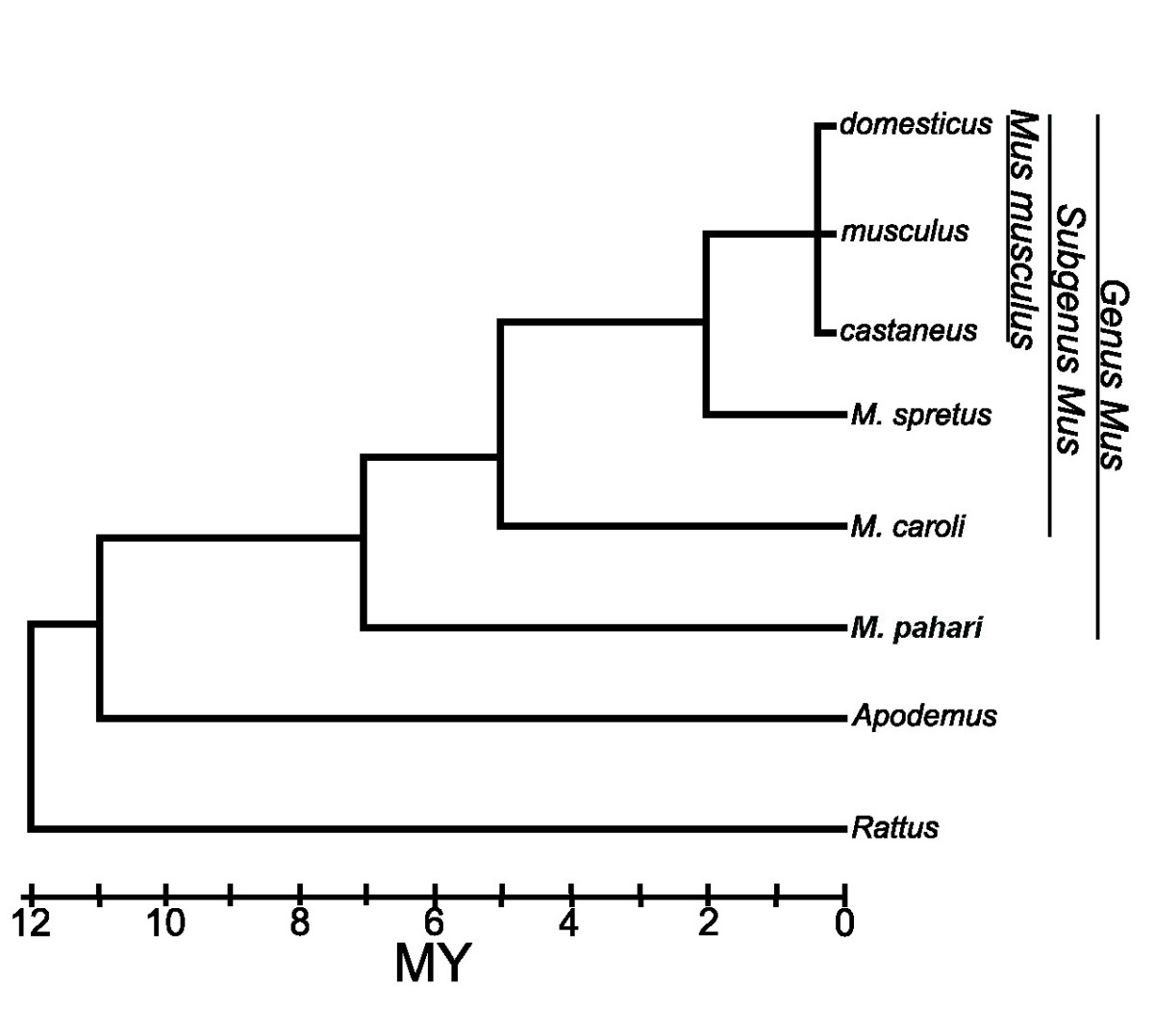

Supplement: Additional file 2 — ZIP files containing several folders, each of which with TreeSnatcher Plus snapshot files, the original image and a text file. [file 1471-2105-13-110-S2.zip › 1471-2148-8-46-1/1471-2148-8-46-1-l.jpg]

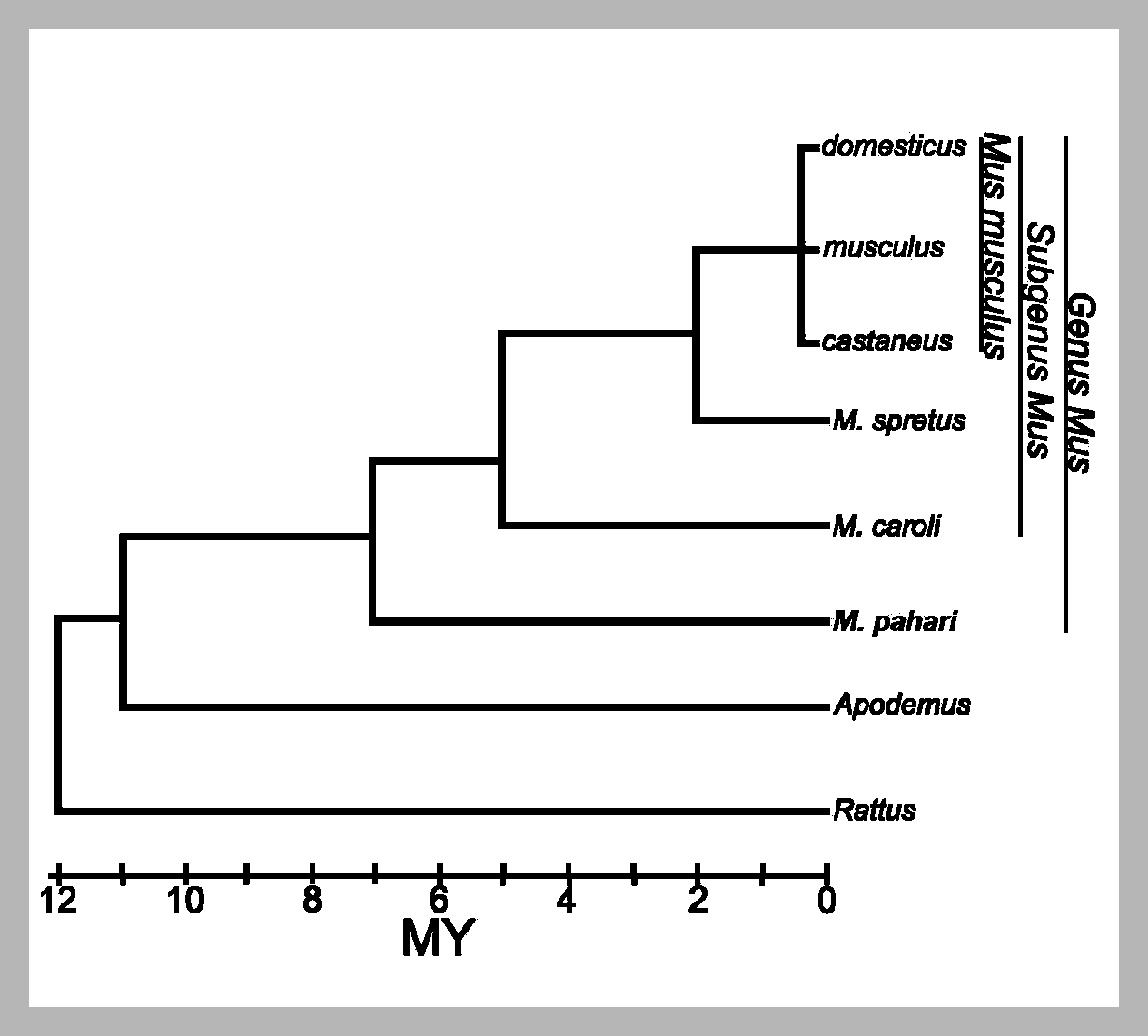

Supplement: Additional file 2 — ZIP files containing several folders, each of which with TreeSnatcher Plus snapshot files, the original image and a text file. [file 1471-2105-13-110-S2.zip › 1471-2148-8-46-1/1471-2148-8-46-1-l_b.PNG]

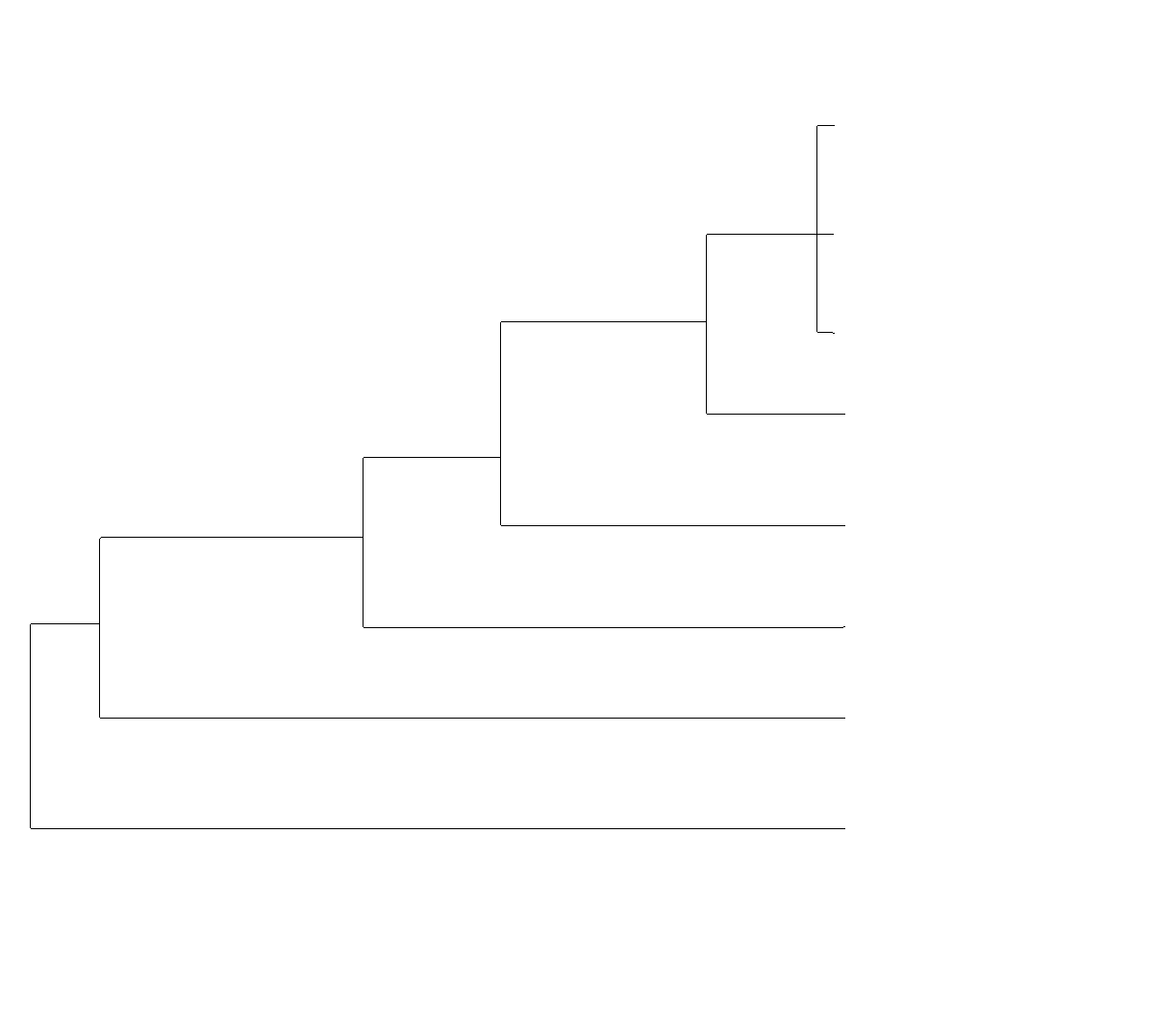

Supplement: Additional file 2 — ZIP files containing several folders, each of which with TreeSnatcher Plus snapshot files, the original image and a text file. [file 1471-2105-13-110-S2.zip › 1471-2148-8-46-1/1471-2148-8-46-1-l_c.PNG]

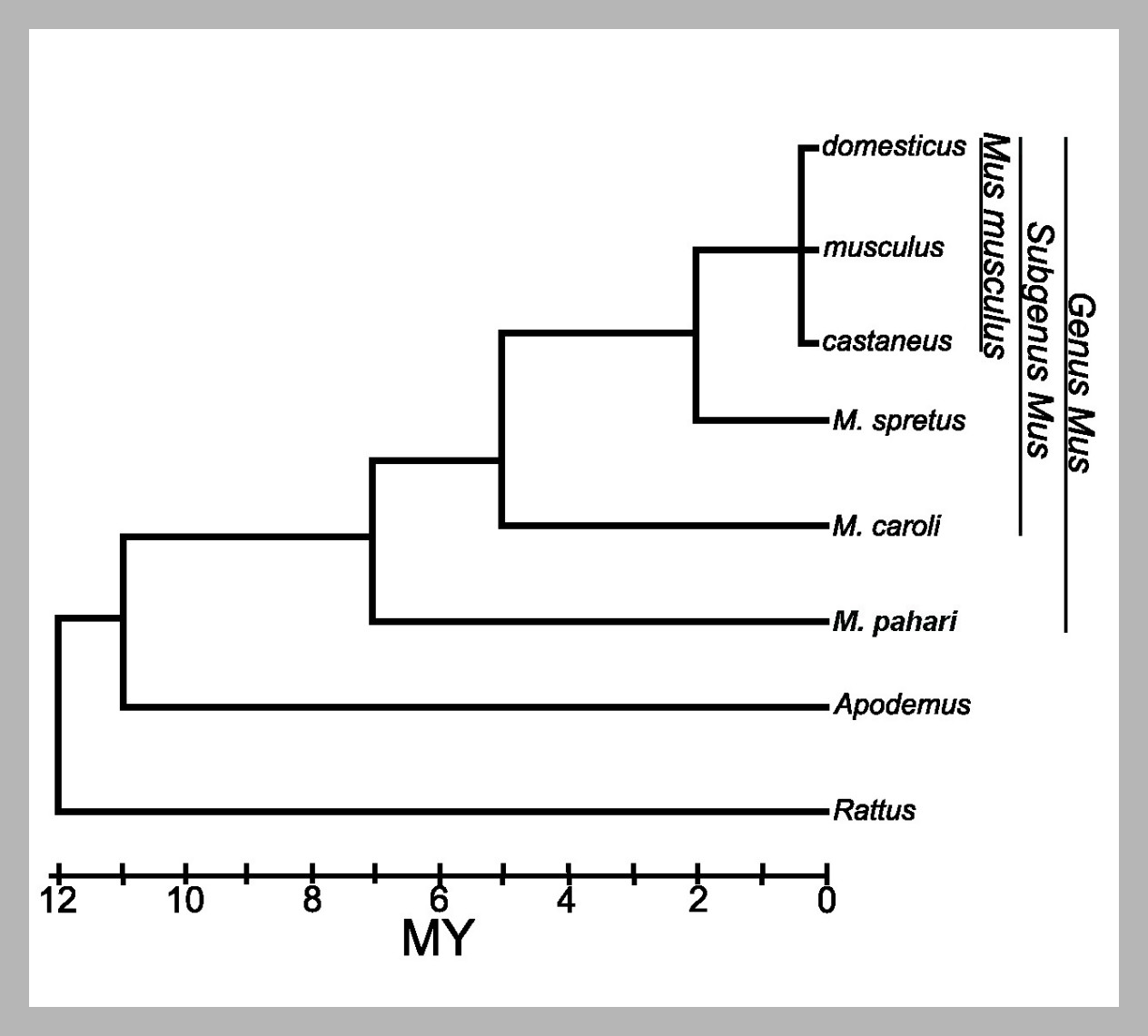

Supplement: Additional file 2 — ZIP files containing several folders, each of which with TreeSnatcher Plus snapshot files, the original image and a text file. [file 1471-2105-13-110-S2.zip › 1471-2148-8-46-1/1471-2148-8-46-1-l_o.PNG]
