# Supplementary figures and images for: TreeSnatcher plus: capturing phylogenetic trees from images (part 2 of 5)
Source: BMC Bioinformatics. 2012 May 24;13:110. doi: 10.1186/1471-2105-13-110 (PMC3411374; doi:10.1186/1471-2105-13-110)

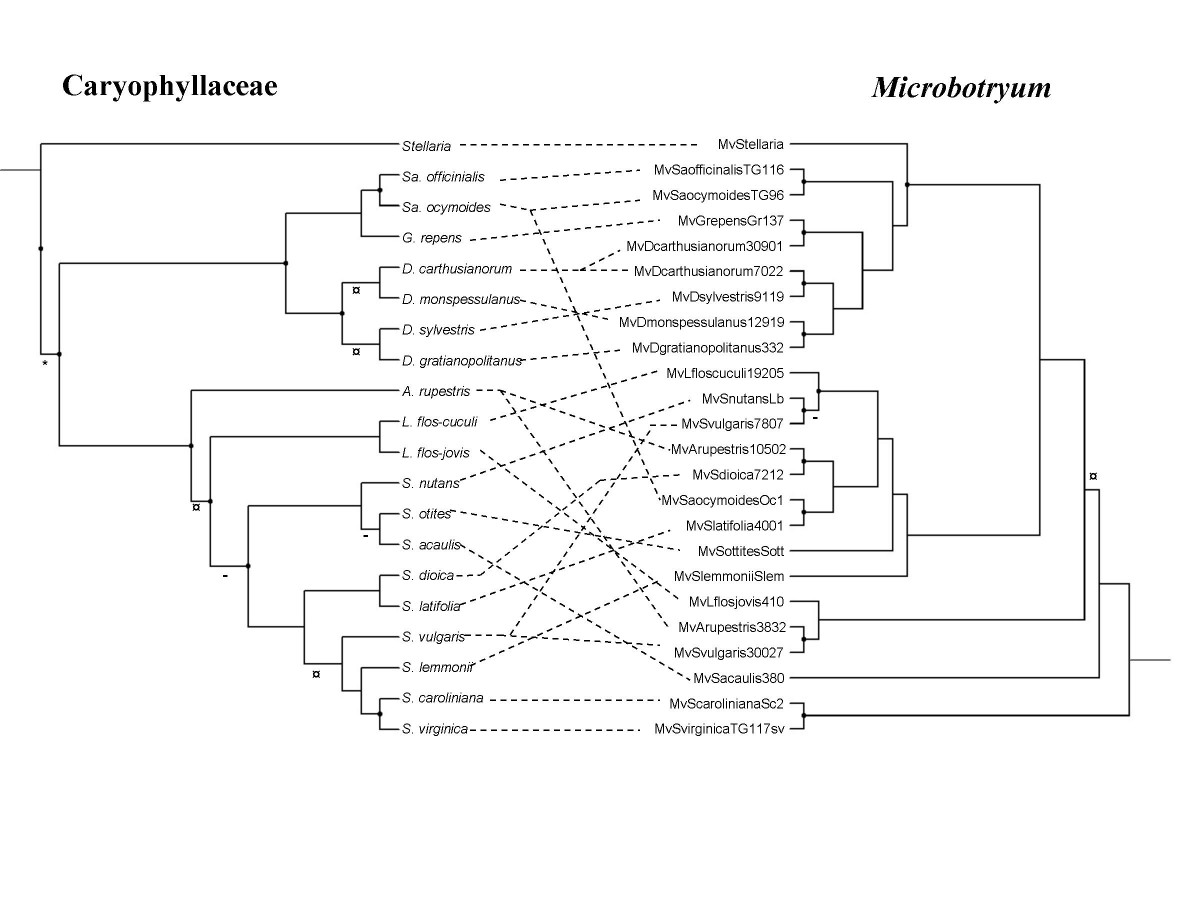

Supplement: Additional file 3 — ZIP files containing several folders, each of which with TreeSnatcher Plus snapshot files, the original image and a text file. [file 1471-2105-13-110-S3.zip › 1471-2148-8-100-3/1471-2148-8-100-3-l.jpg]

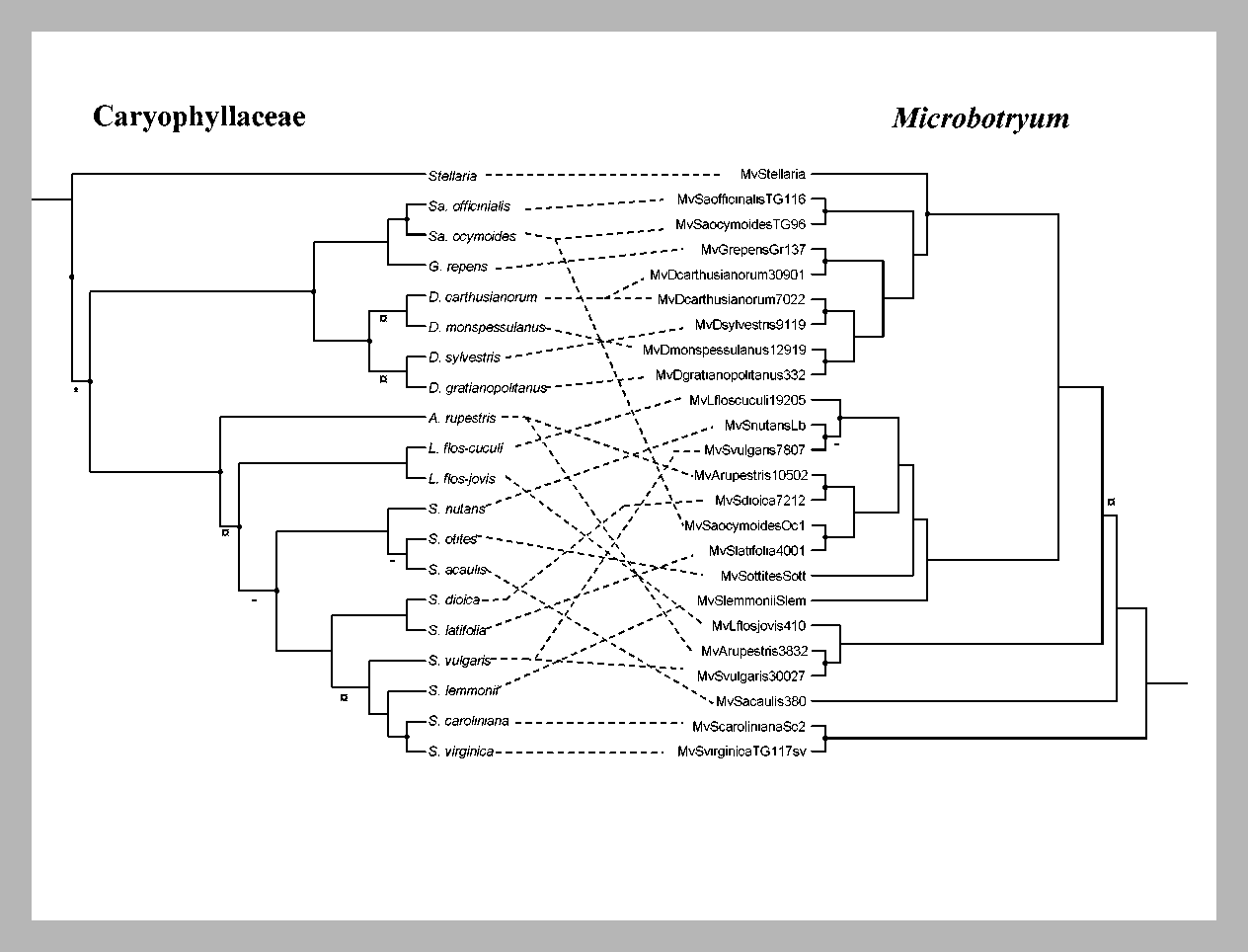

Supplement: Additional file 3 — ZIP files containing several folders, each of which with TreeSnatcher Plus snapshot files, the original image and a text file. [file 1471-2105-13-110-S3.zip › 1471-2148-8-100-3/1471-2148-8-100-3-l_b.PNG]

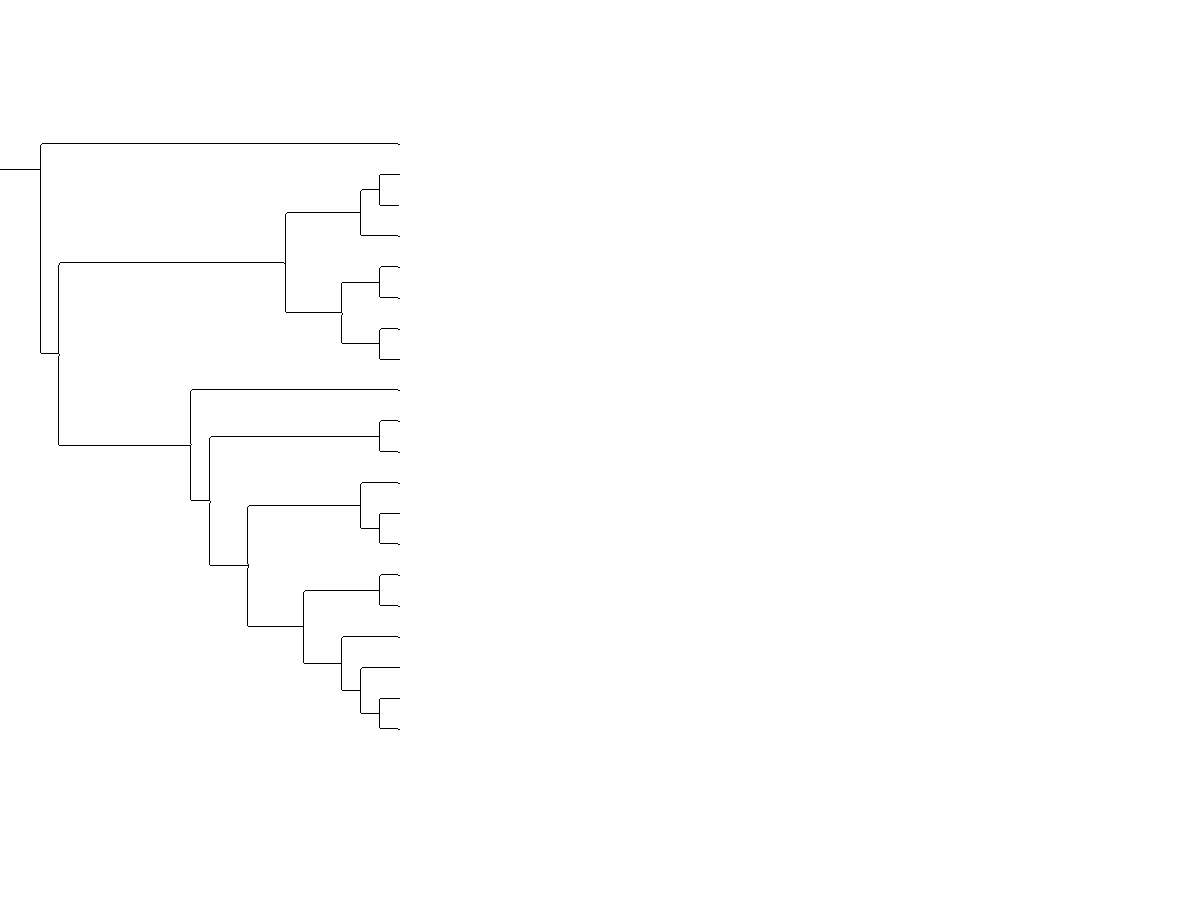

Supplement: Additional file 3 — ZIP files containing several folders, each of which with TreeSnatcher Plus snapshot files, the original image and a text file. [file 1471-2105-13-110-S3.zip › 1471-2148-8-100-3/1471-2148-8-100-3-l_c.PNG]

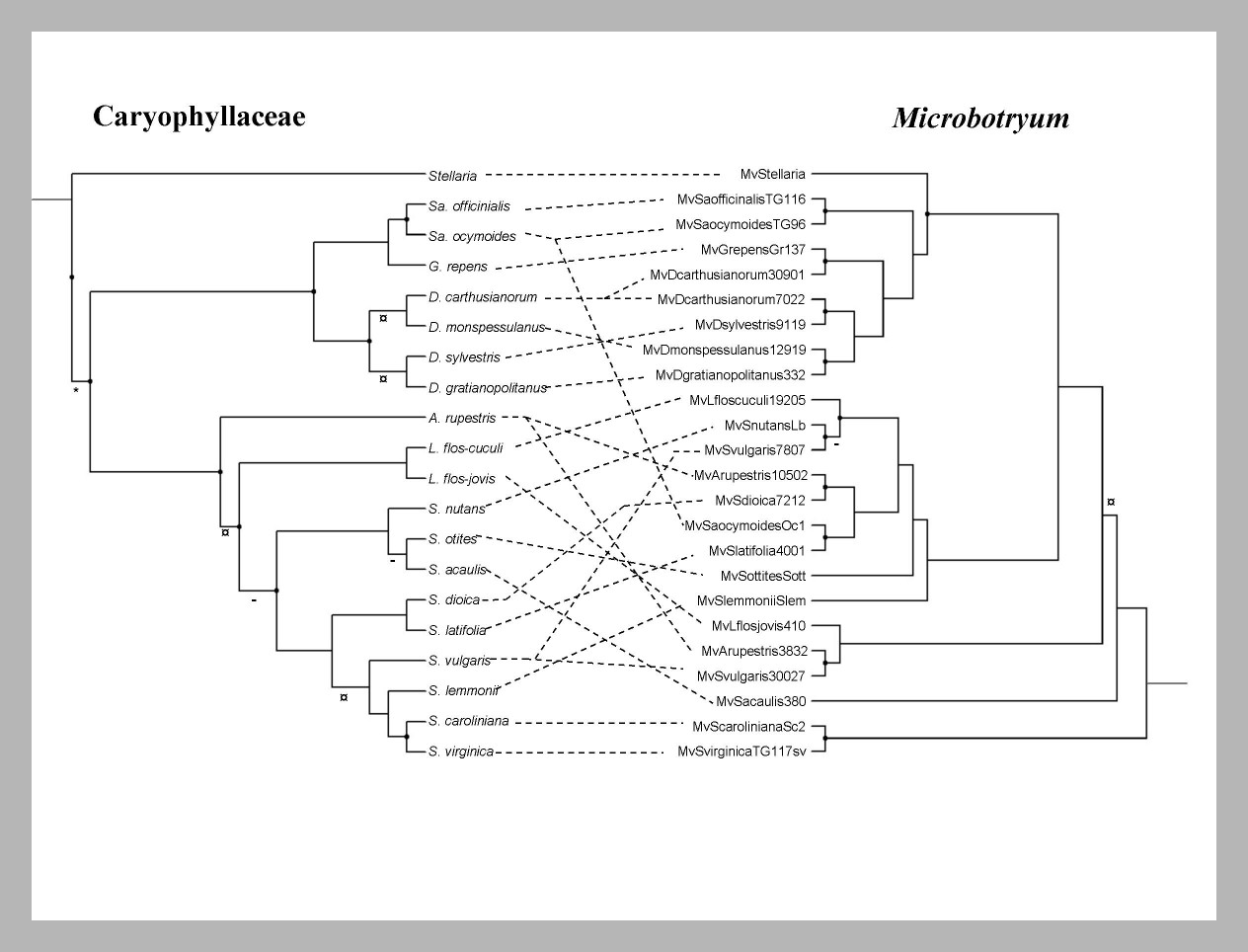

Supplement: Additional file 3 — ZIP files containing several folders, each of which with TreeSnatcher Plus snapshot files, the original image and a text file. [file 1471-2105-13-110-S3.zip › 1471-2148-8-100-3/1471-2148-8-100-3-l_o.PNG]

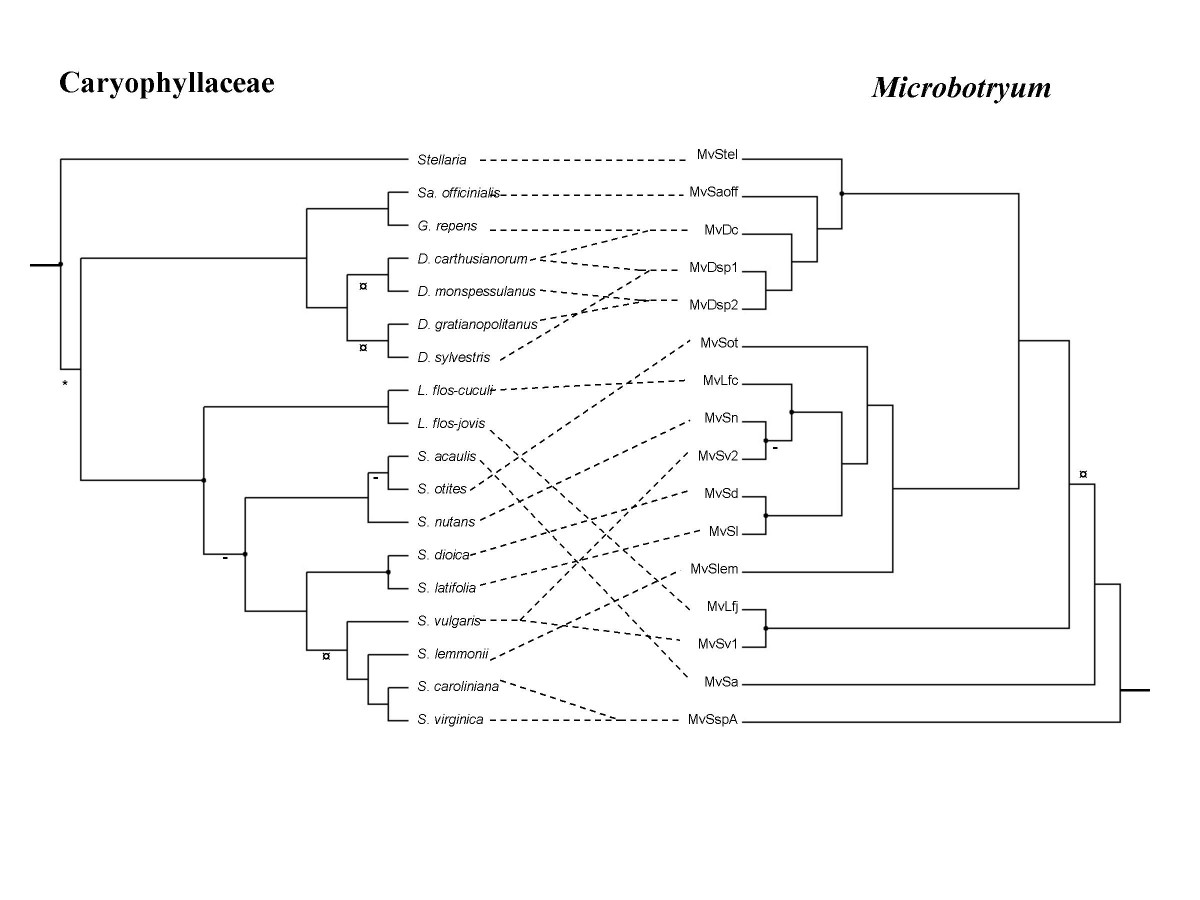

Supplement: Additional file 3 — ZIP files containing several folders, each of which with TreeSnatcher Plus snapshot files, the original image and a text file. [file 1471-2105-13-110-S3.zip › 1471-2148-8-100-4/1471-2148-8-100-4-l.jpg]

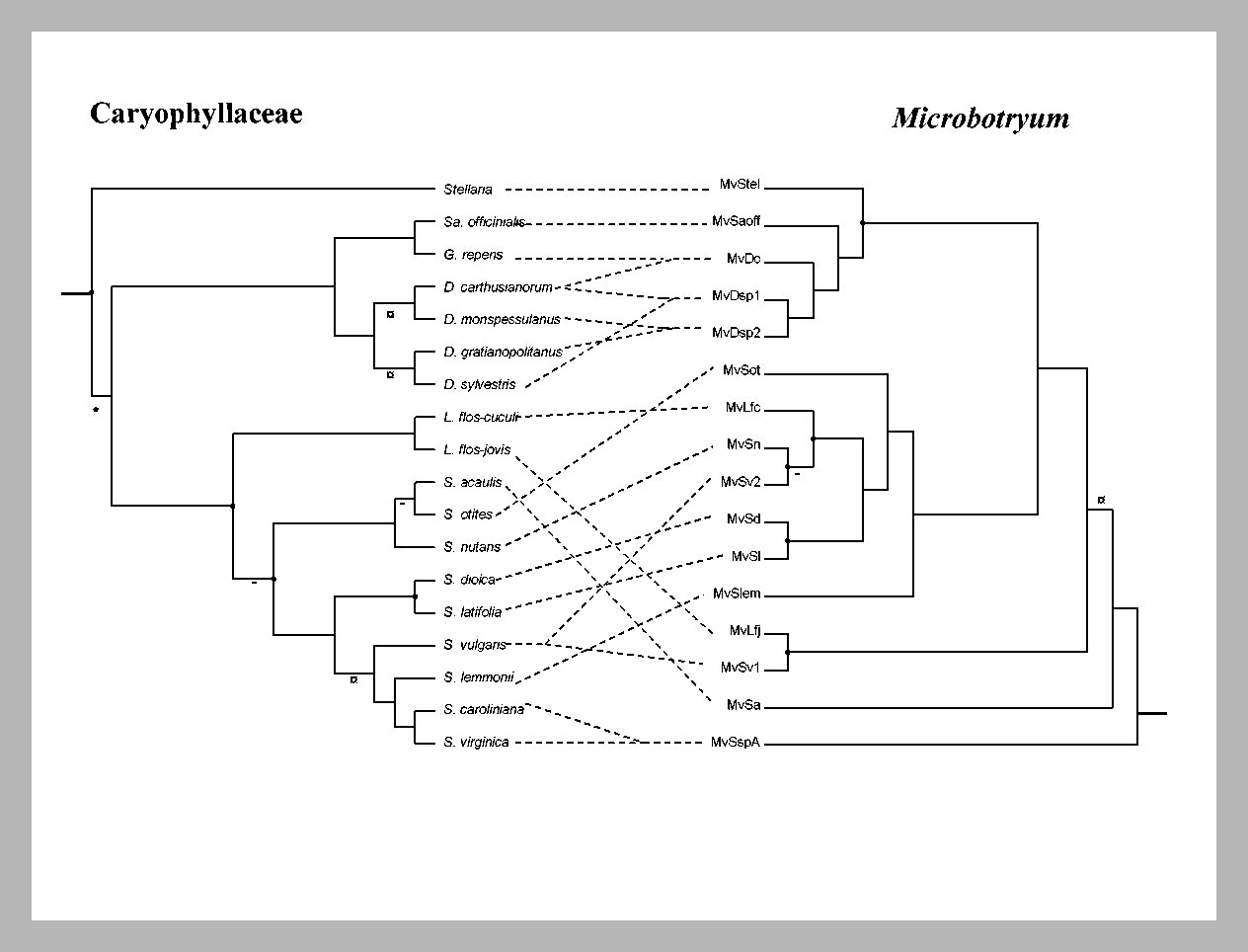

Supplement: Additional file 3 — ZIP files containing several folders, each of which with TreeSnatcher Plus snapshot files, the original image and a text file. [file 1471-2105-13-110-S3.zip › 1471-2148-8-100-4/1471-2148-8-100-4-l_b.PNG]

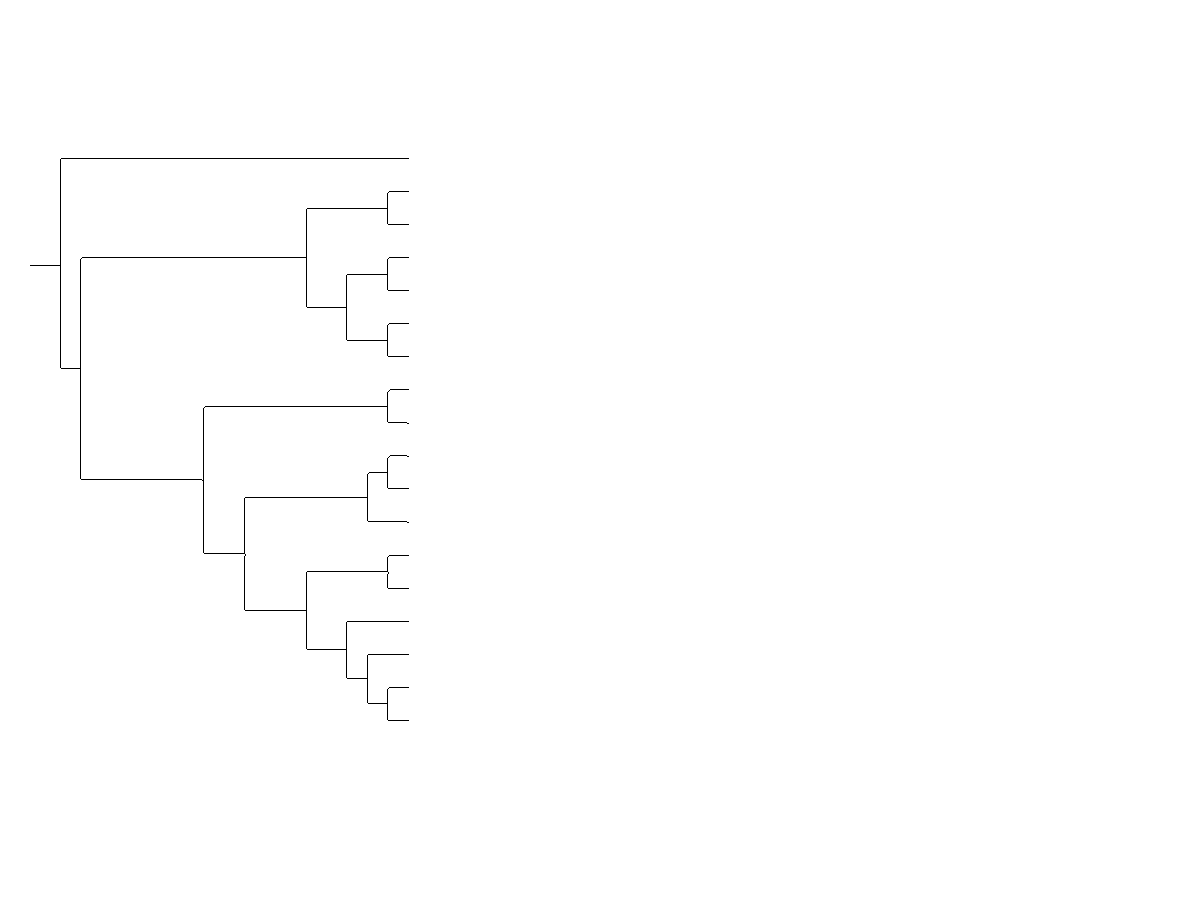

Supplement: Additional file 3 — ZIP files containing several folders, each of which with TreeSnatcher Plus snapshot files, the original image and a text file. [file 1471-2105-13-110-S3.zip › 1471-2148-8-100-4/1471-2148-8-100-4-l_c.PNG]

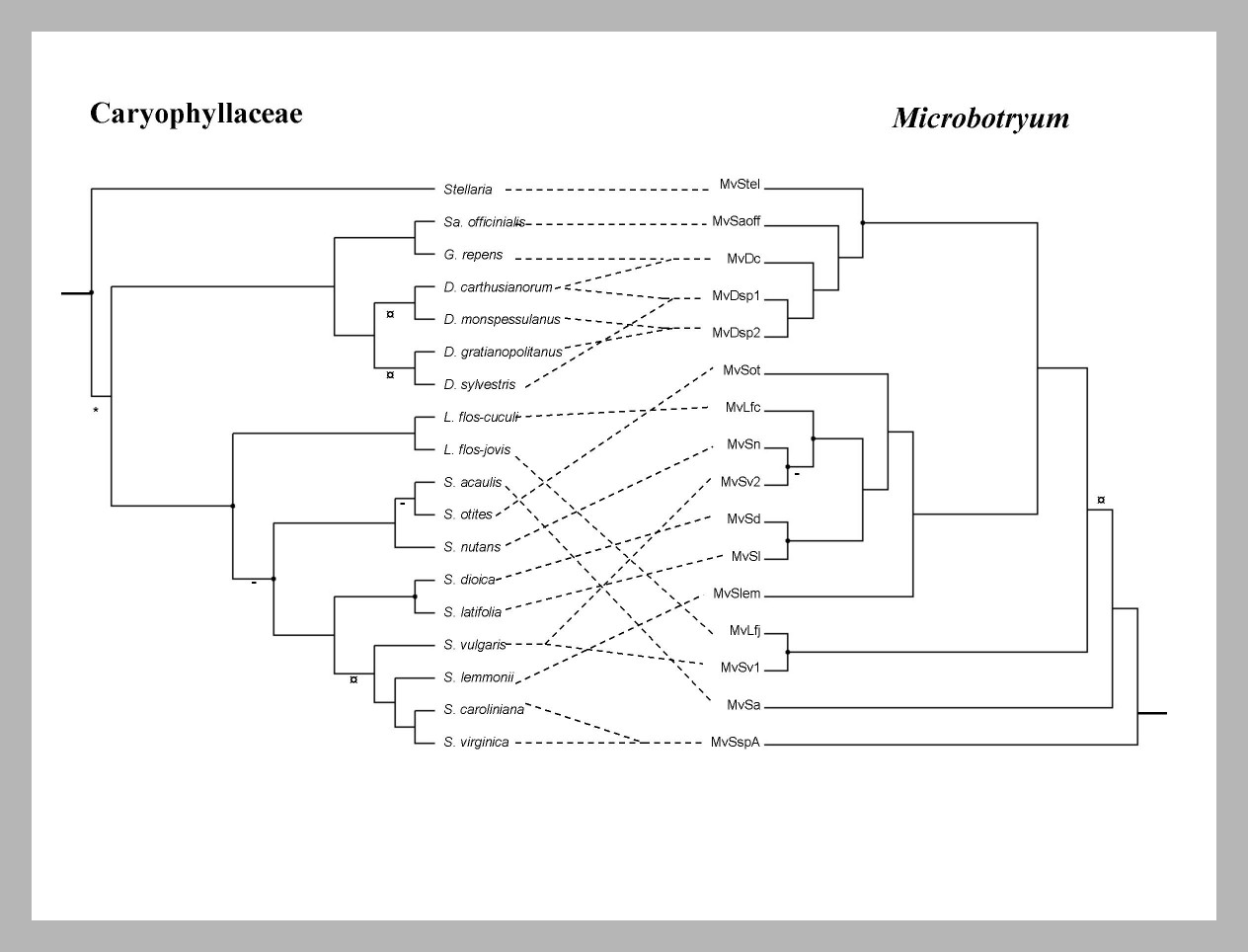

Supplement: Additional file 3 — ZIP files containing several folders, each of which with TreeSnatcher Plus snapshot files, the original image and a text file. [file 1471-2105-13-110-S3.zip › 1471-2148-8-100-4/1471-2148-8-100-4-l_o.PNG]

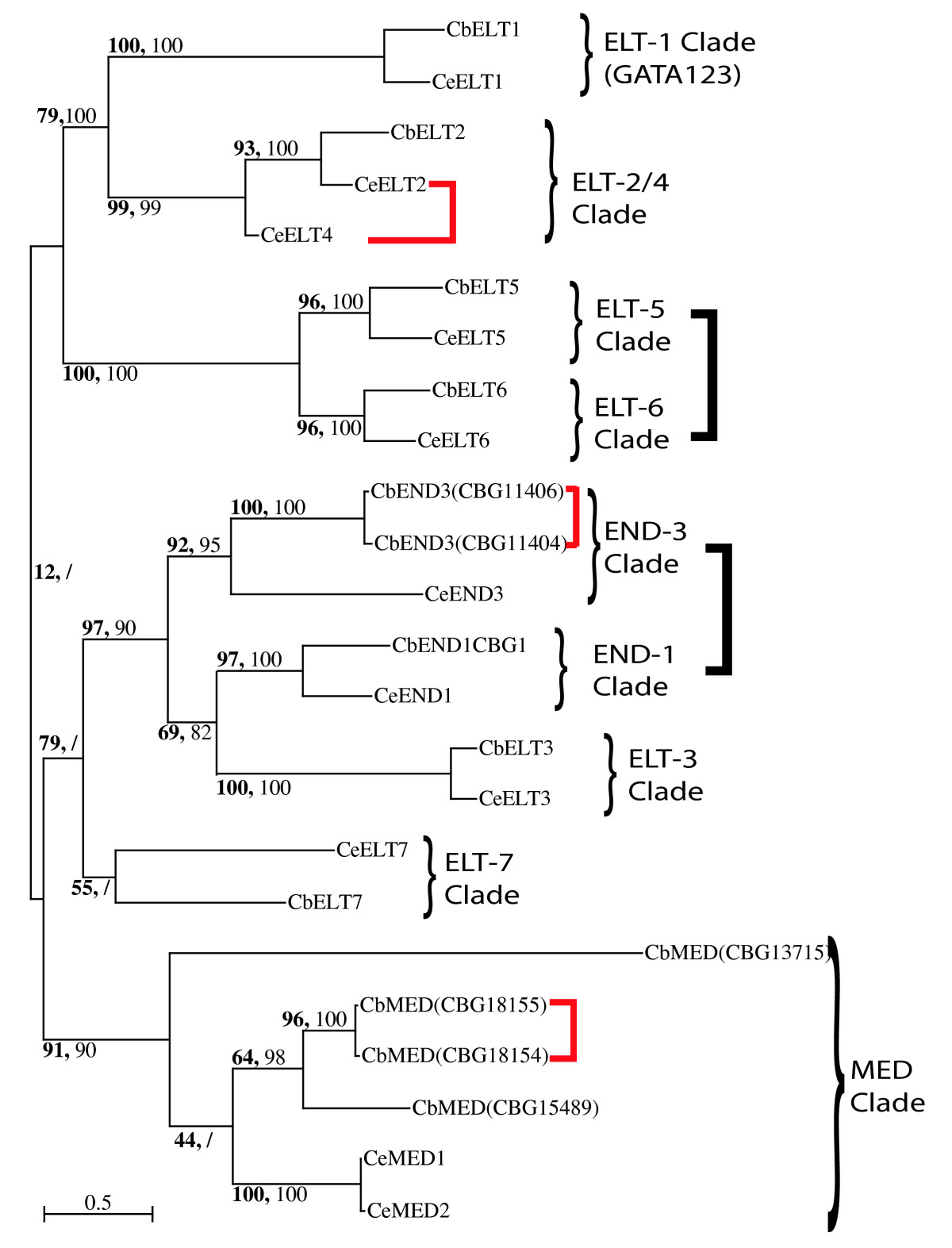

Supplement: Additional file 3 — ZIP files containing several folders, each of which with TreeSnatcher Plus snapshot files, the original image and a text file. [file 1471-2105-13-110-S3.zip › 1471-2148-8-112-4/1471-2148-8-112-4-l.jpg]

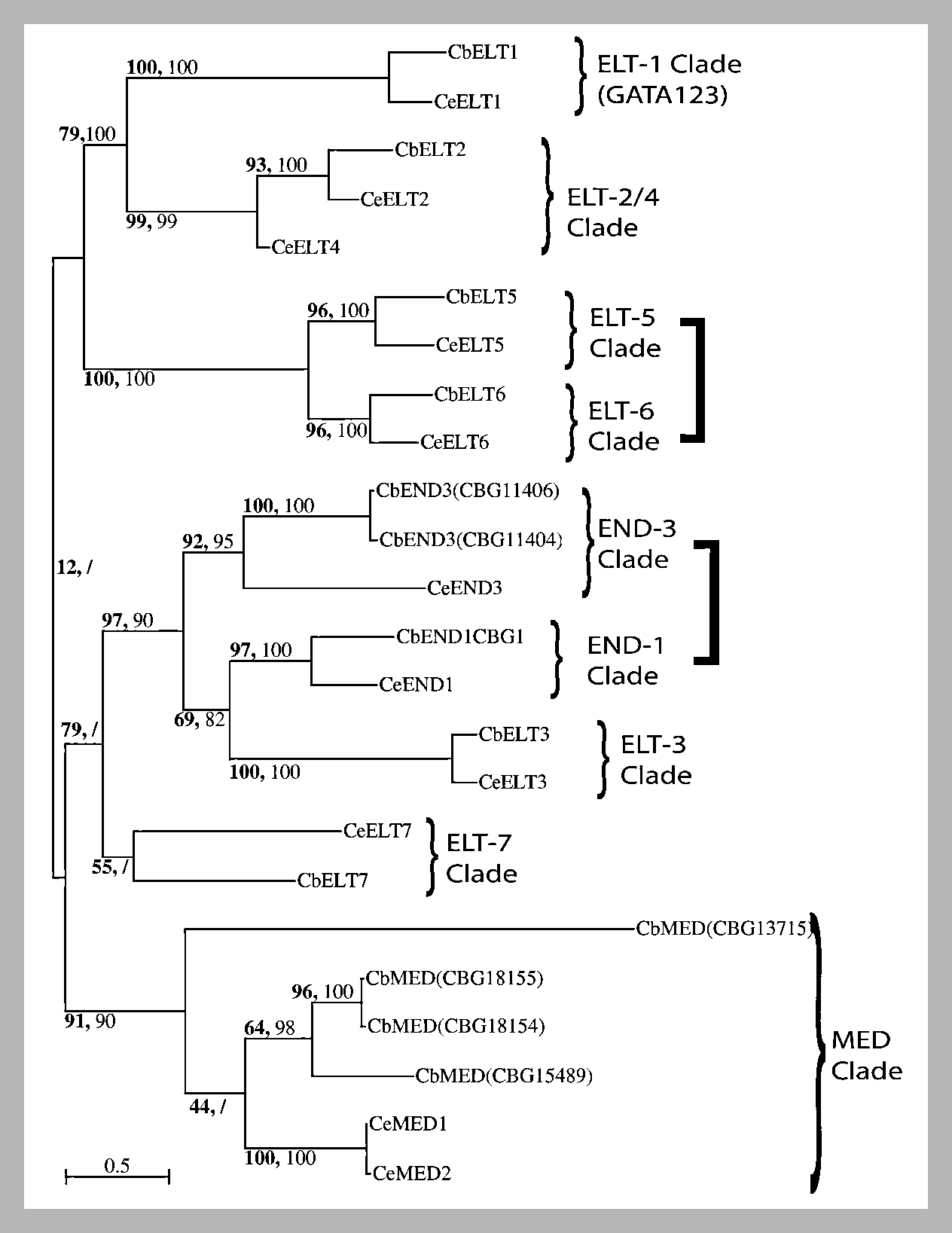

Supplement: Additional file 3 — ZIP files containing several folders, each of which with TreeSnatcher Plus snapshot files, the original image and a text file. [file 1471-2105-13-110-S3.zip › 1471-2148-8-112-4/1471-2148-8-112-4-l_b.PNG]

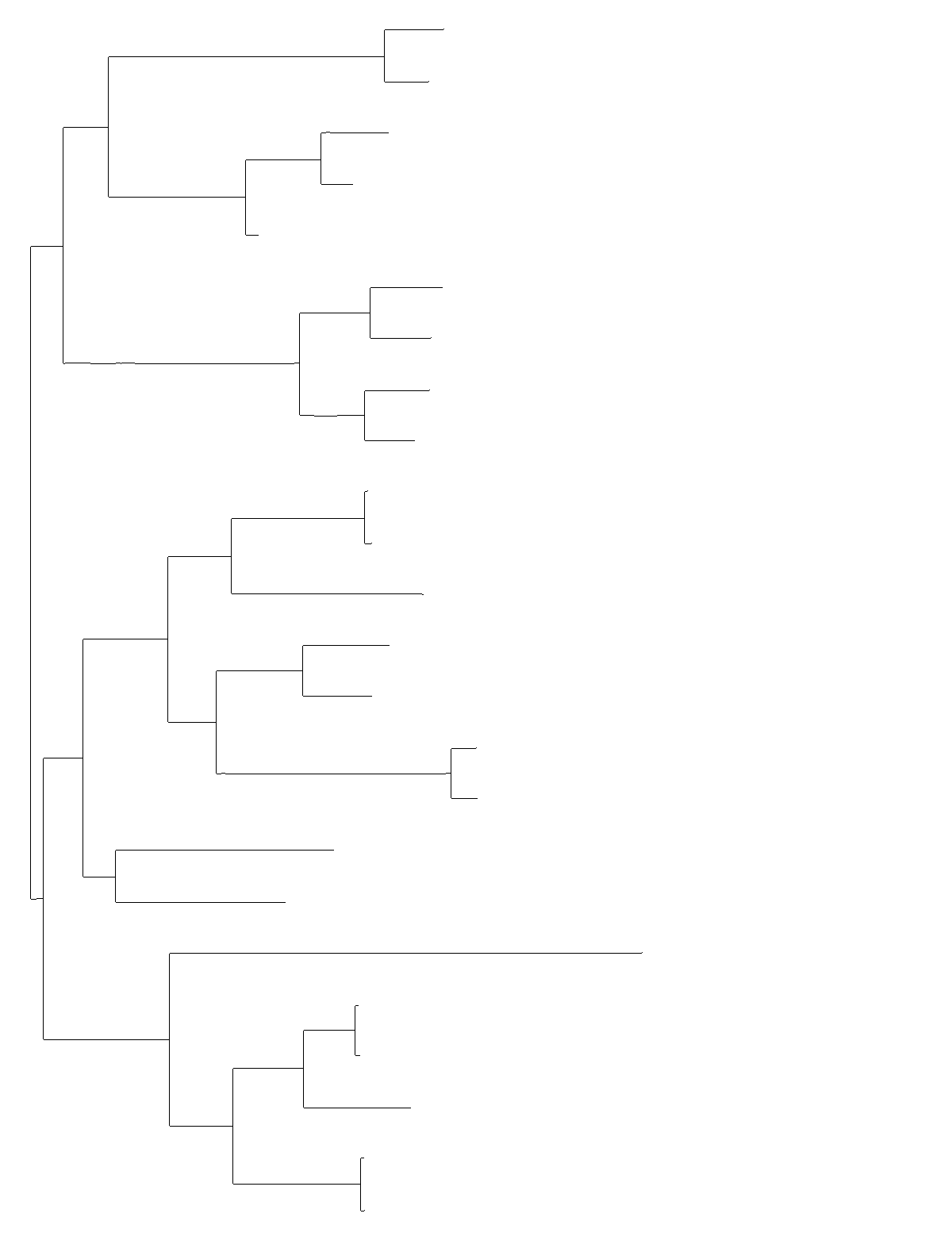

Supplement: Additional file 3 — ZIP files containing several folders, each of which with TreeSnatcher Plus snapshot files, the original image and a text file. [file 1471-2105-13-110-S3.zip › 1471-2148-8-112-4/1471-2148-8-112-4-l_c.PNG]

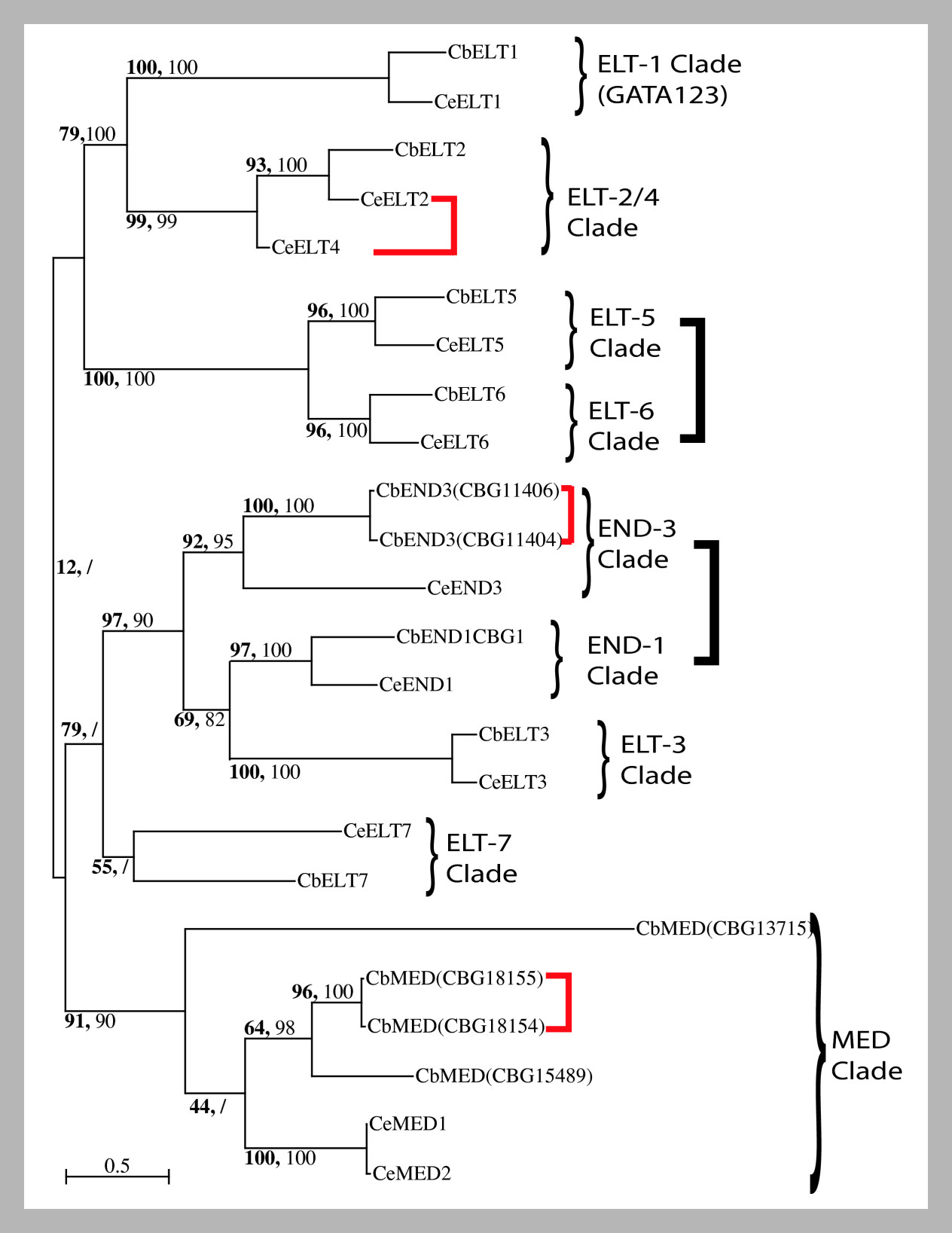

Supplement: Additional file 3 — ZIP files containing several folders, each of which with TreeSnatcher Plus snapshot files, the original image and a text file. [file 1471-2105-13-110-S3.zip › 1471-2148-8-112-4/1471-2148-8-112-4-l_o.PNG]

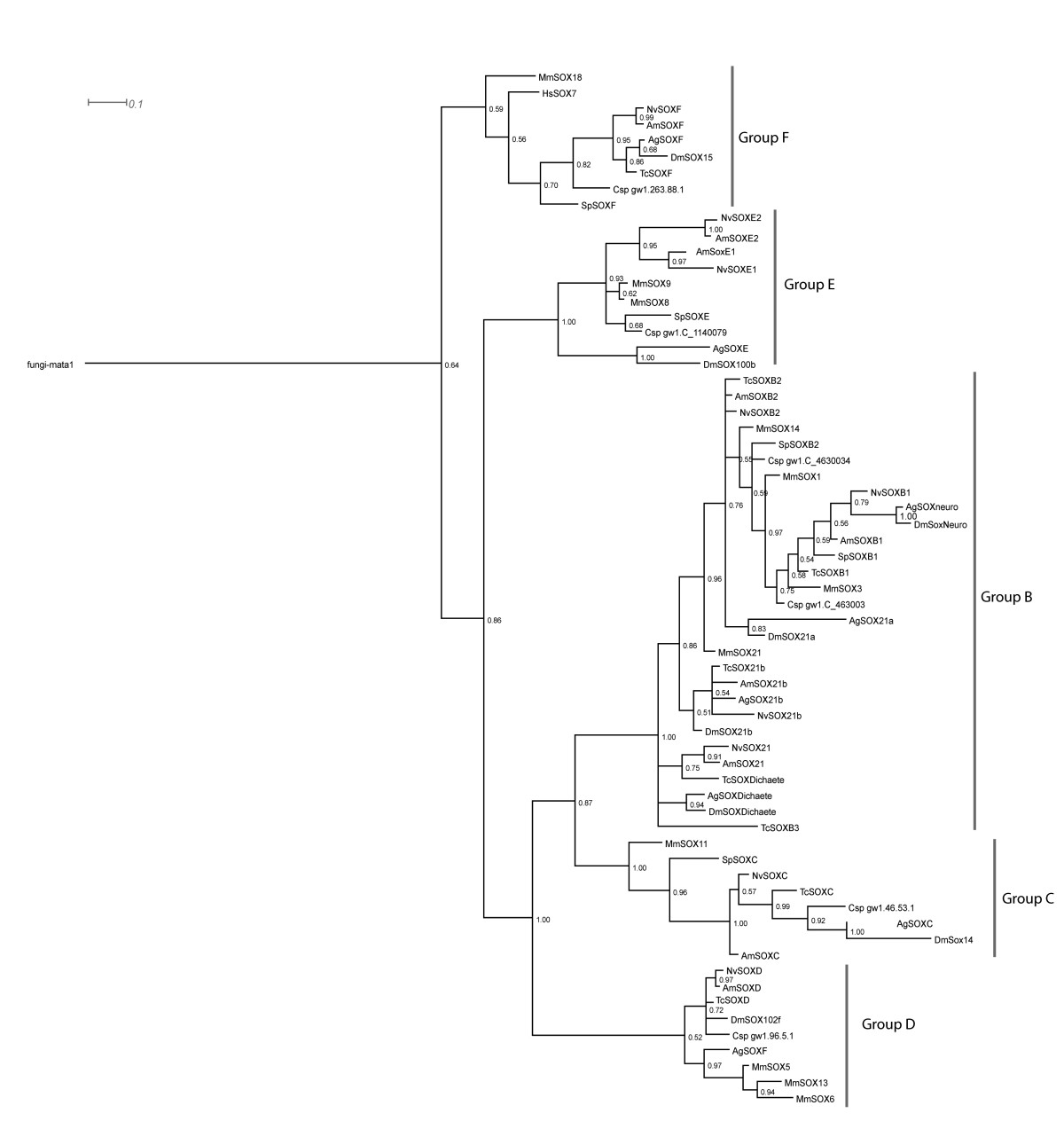

Supplement: Additional file 3 — ZIP files containing several folders, each of which with TreeSnatcher Plus snapshot files, the original image and a text file. [file 1471-2105-13-110-S3.zip › 1471-2148-8-120-1/1471-2148-8-120-1-l.jpg]

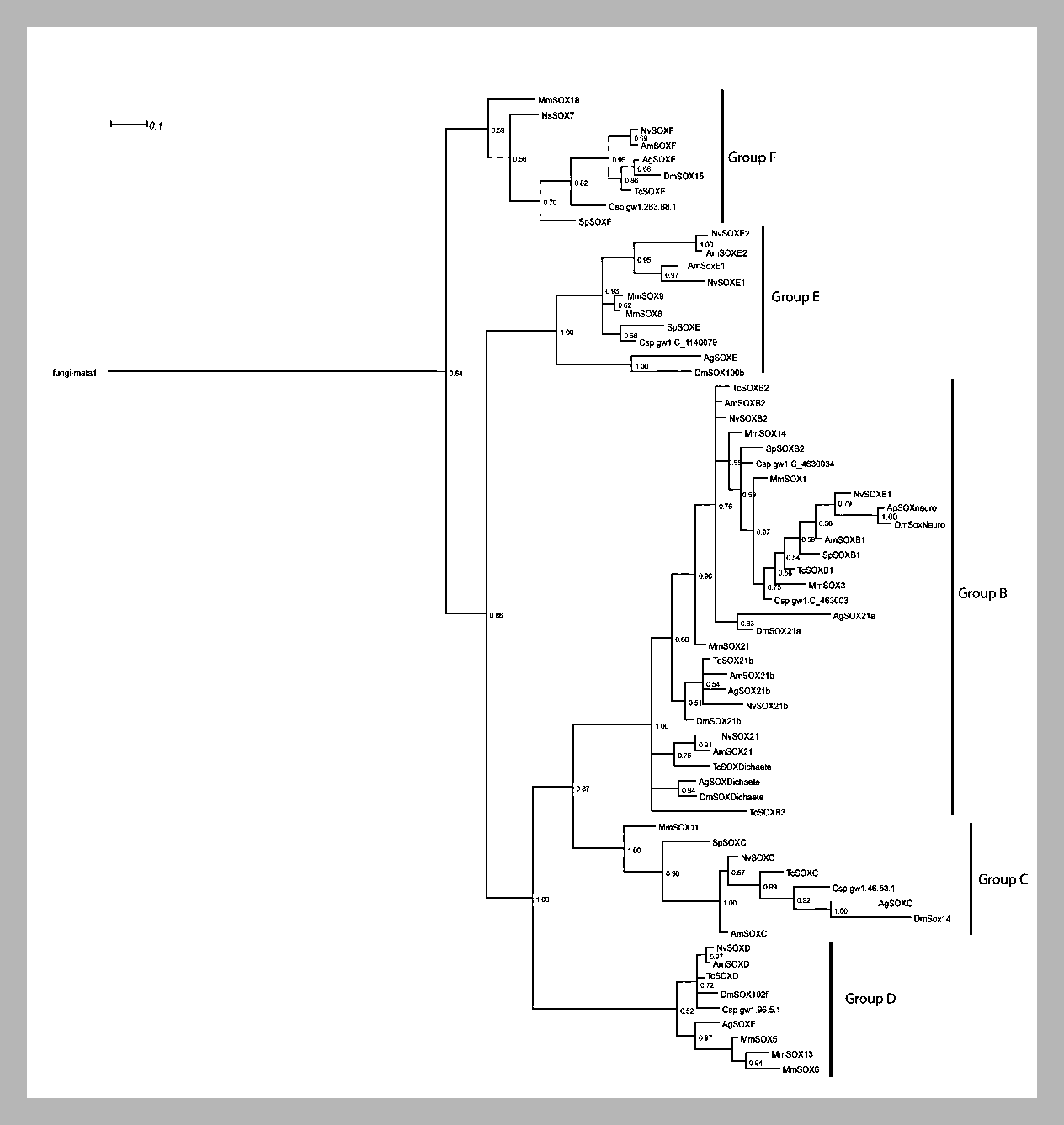

Supplement: Additional file 3 — ZIP files containing several folders, each of which with TreeSnatcher Plus snapshot files, the original image and a text file. [file 1471-2105-13-110-S3.zip › 1471-2148-8-120-1/1471-2148-8-120-1-l_b.PNG]

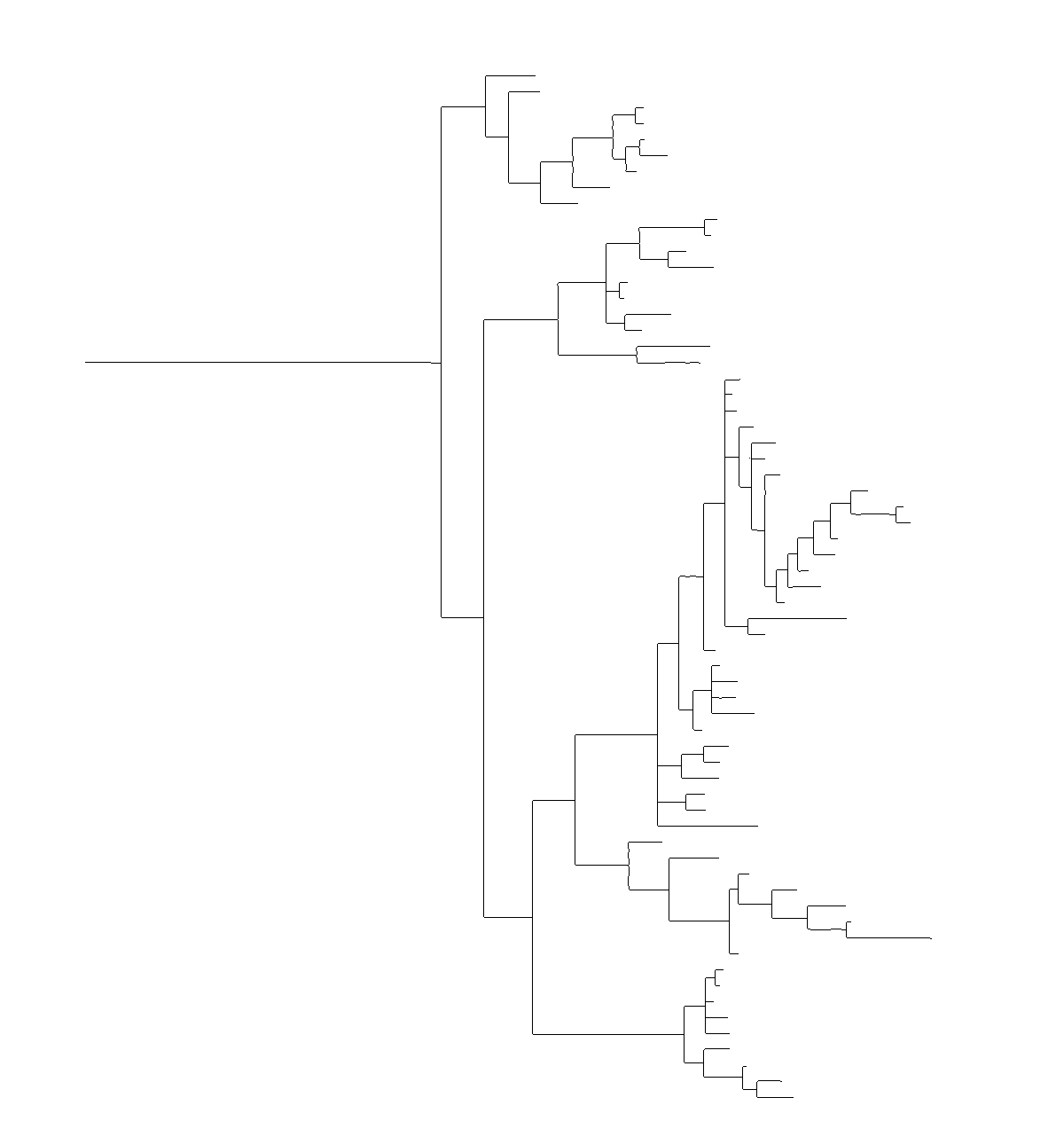

Supplement: Additional file 3 — ZIP files containing several folders, each of which with TreeSnatcher Plus snapshot files, the original image and a text file. [file 1471-2105-13-110-S3.zip › 1471-2148-8-120-1/1471-2148-8-120-1-l_c.PNG]

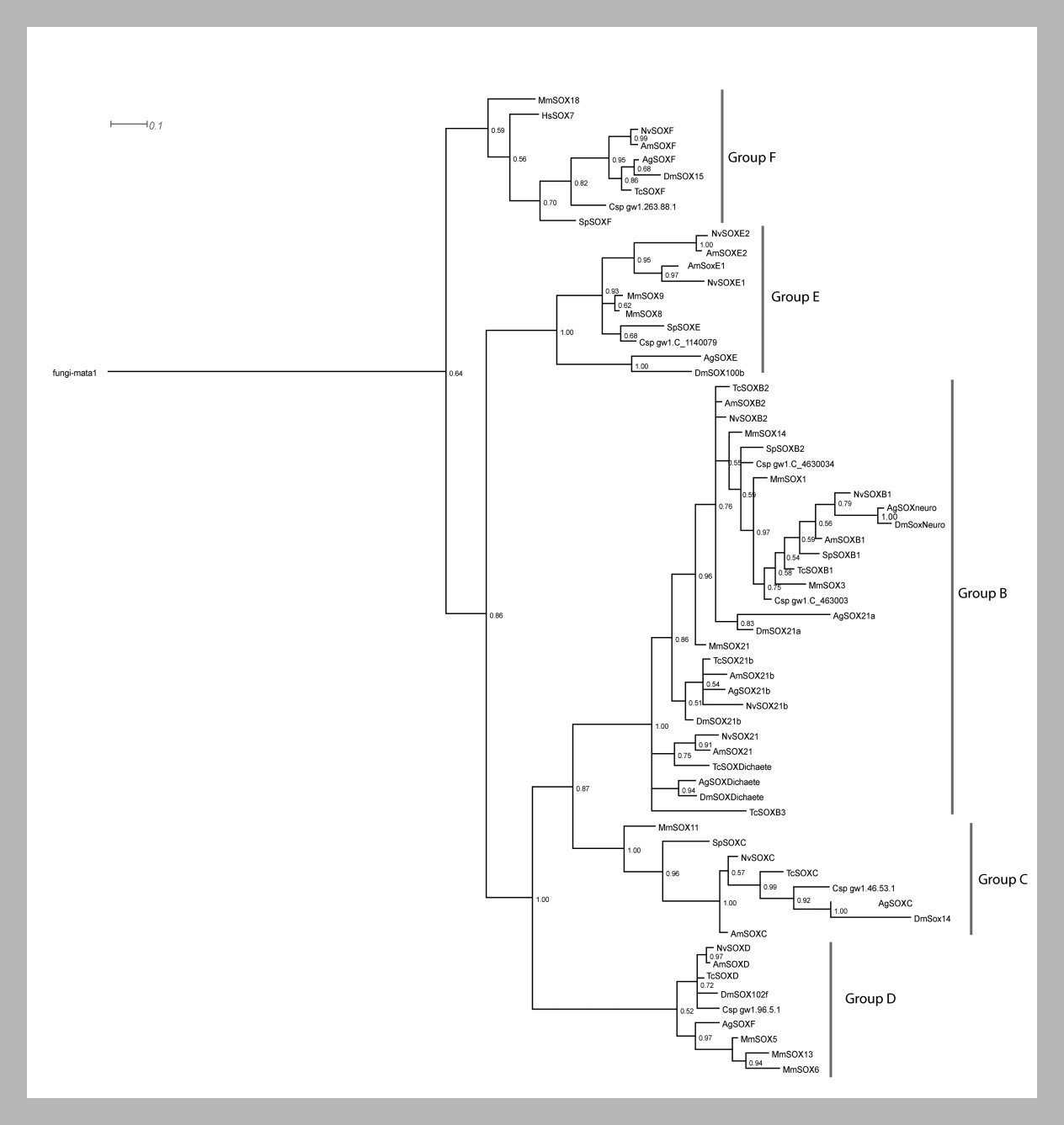

Supplement: Additional file 3 — ZIP files containing several folders, each of which with TreeSnatcher Plus snapshot files, the original image and a text file. [file 1471-2105-13-110-S3.zip › 1471-2148-8-120-1/1471-2148-8-120-1-l_o.PNG]

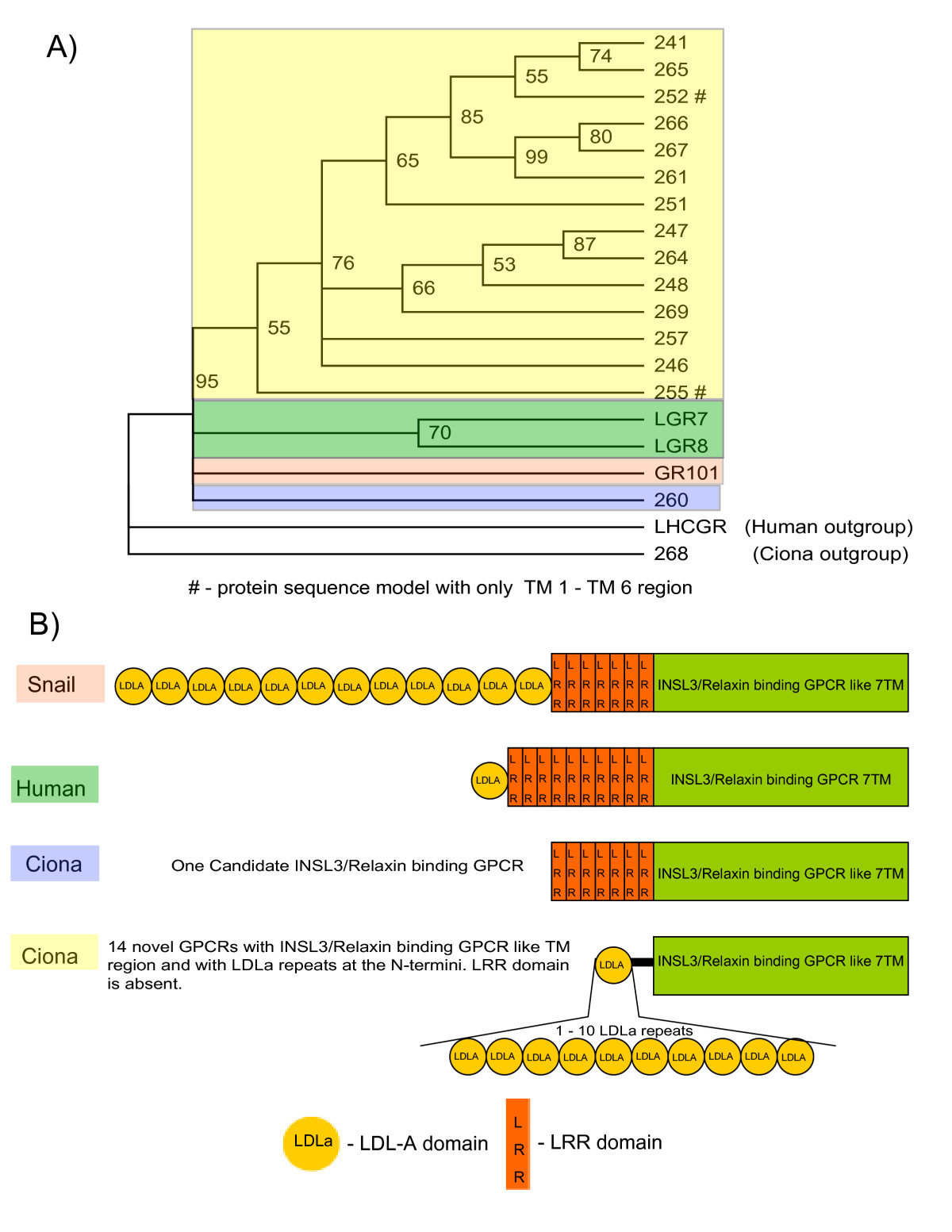

Supplement: Additional file 3 — ZIP files containing several folders, each of which with TreeSnatcher Plus snapshot files, the original image and a text file. [file 1471-2105-13-110-S3.zip › 1471-2148-8-129-4/1471-2148-8-129-4-l.jpg]

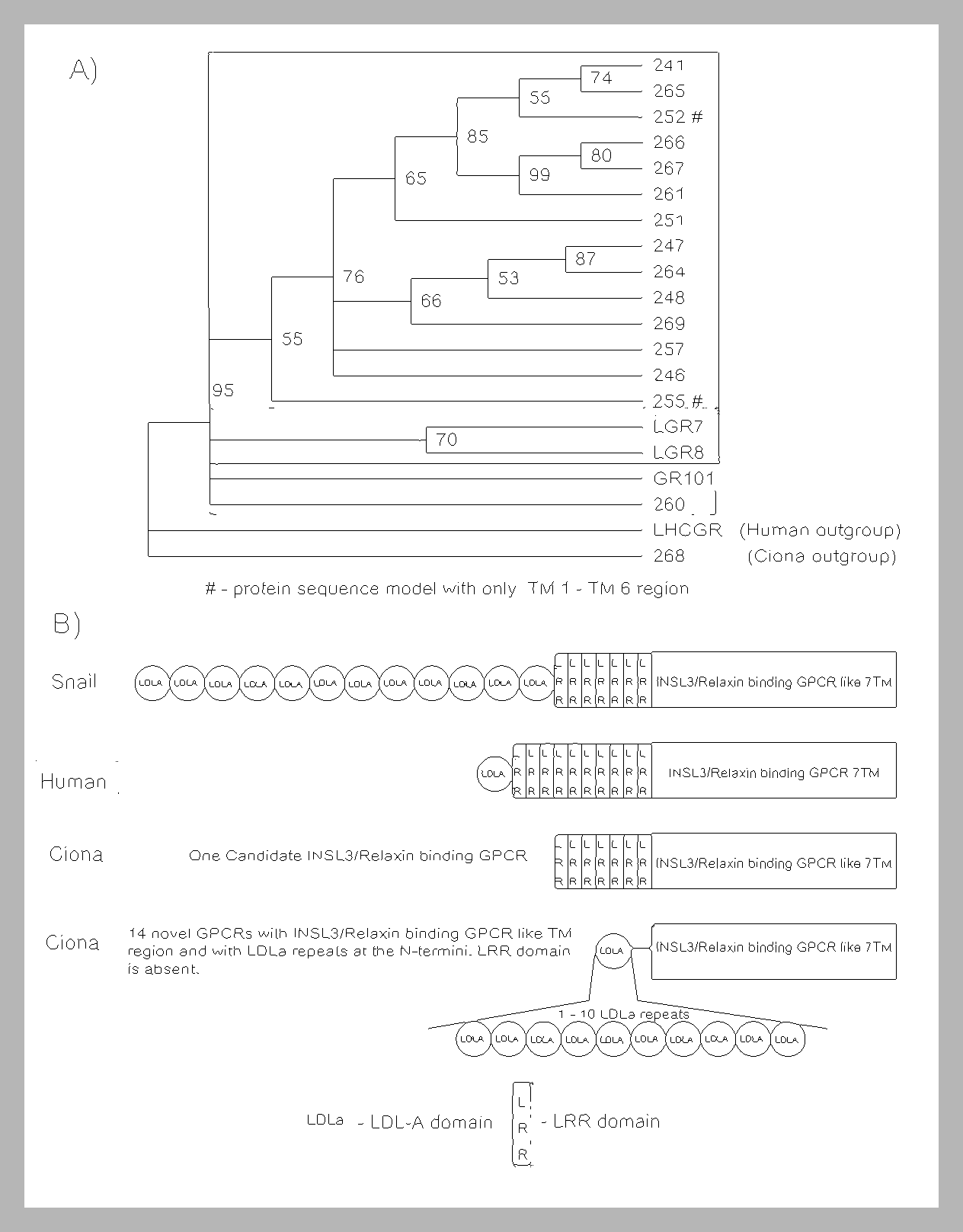

Supplement: Additional file 3 — ZIP files containing several folders, each of which with TreeSnatcher Plus snapshot files, the original image and a text file. [file 1471-2105-13-110-S3.zip › 1471-2148-8-129-4/1471-2148-8-129-4-l_b.PNG]

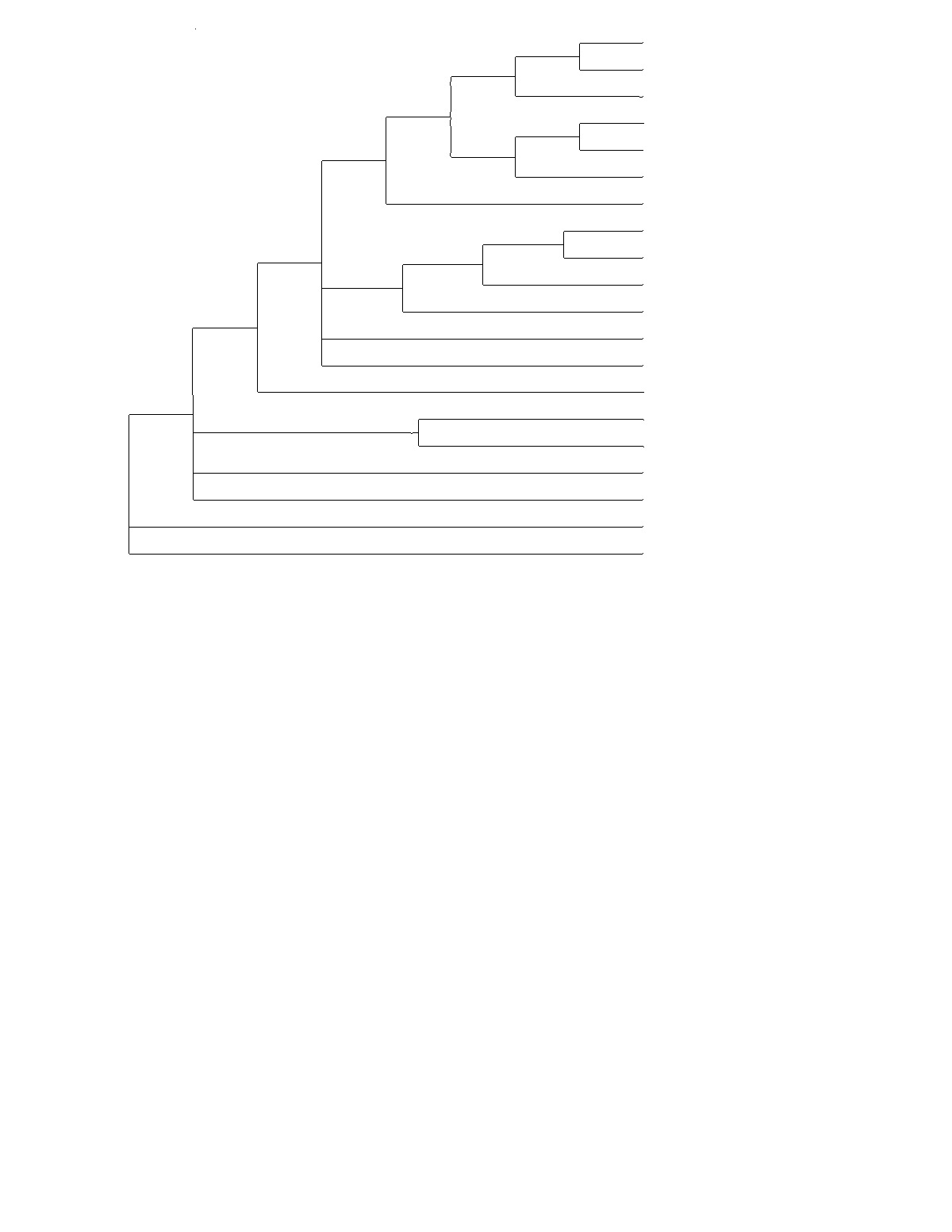

Supplement: Additional file 3 — ZIP files containing several folders, each of which with TreeSnatcher Plus snapshot files, the original image and a text file. [file 1471-2105-13-110-S3.zip › 1471-2148-8-129-4/1471-2148-8-129-4-l_c.PNG]

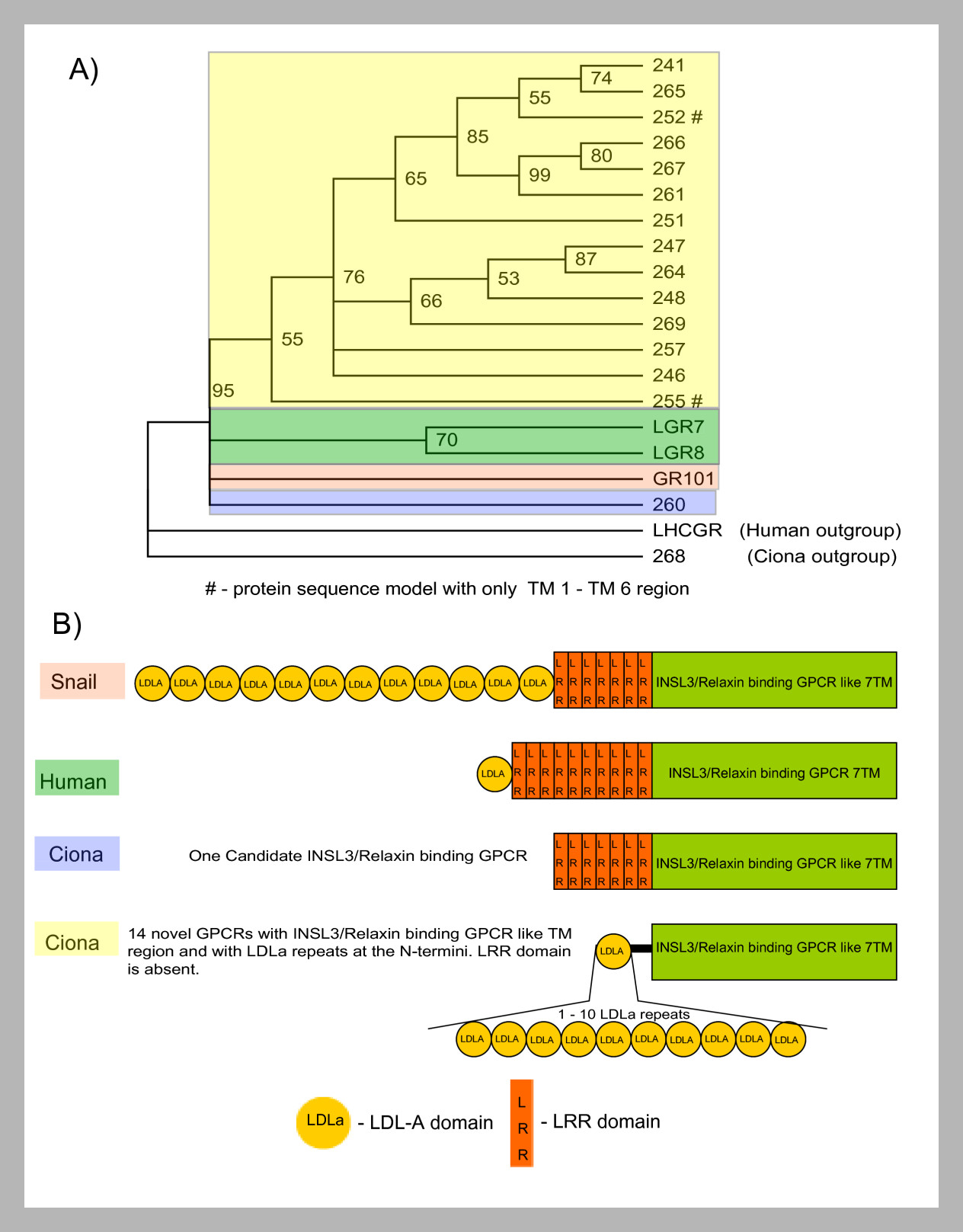

Supplement: Additional file 3 — ZIP files containing several folders, each of which with TreeSnatcher Plus snapshot files, the original image and a text file. [file 1471-2105-13-110-S3.zip › 1471-2148-8-129-4/1471-2148-8-129-4-l_o.PNG]

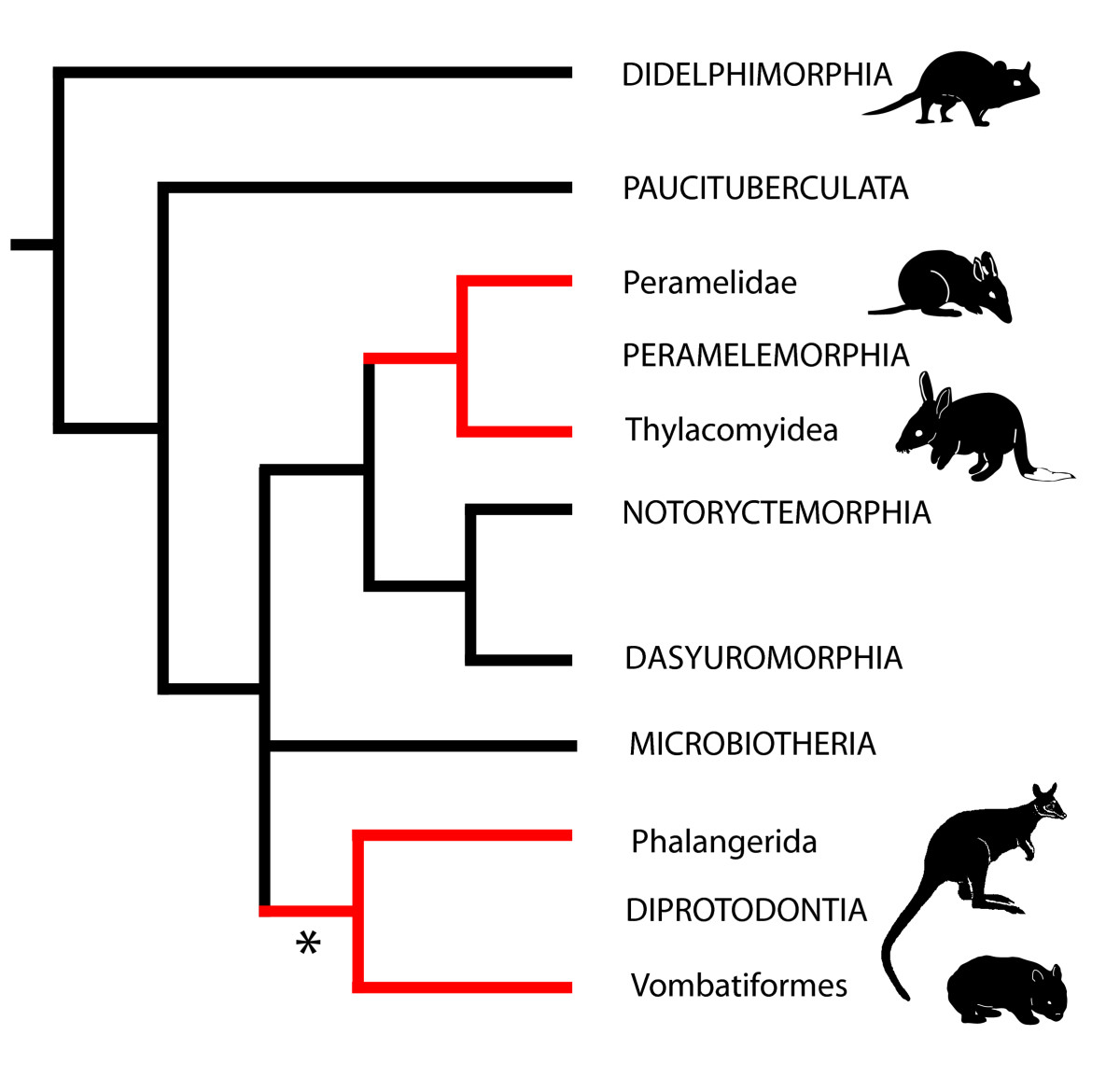

Supplement: Additional file 3 — ZIP files containing several folders, each of which with TreeSnatcher Plus snapshot files, the original image and a text file. [file 1471-2105-13-110-S3.zip › 1471-2148-8-160-1/1471-2148-8-160-1-l.jpg]

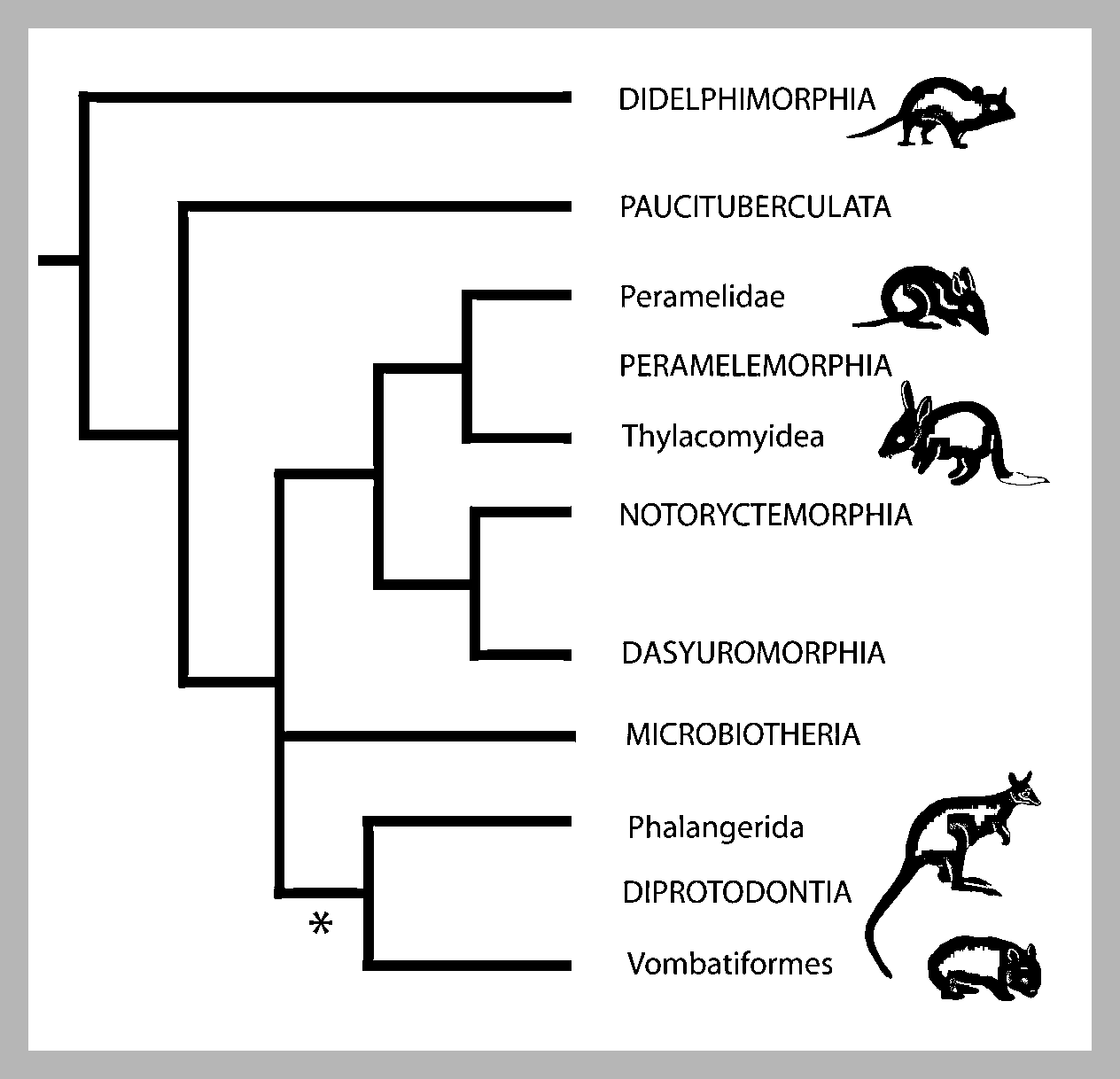

Supplement: Additional file 3 — ZIP files containing several folders, each of which with TreeSnatcher Plus snapshot files, the original image and a text file. [file 1471-2105-13-110-S3.zip › 1471-2148-8-160-1/1471-2148-8-160-1-l_b.PNG]

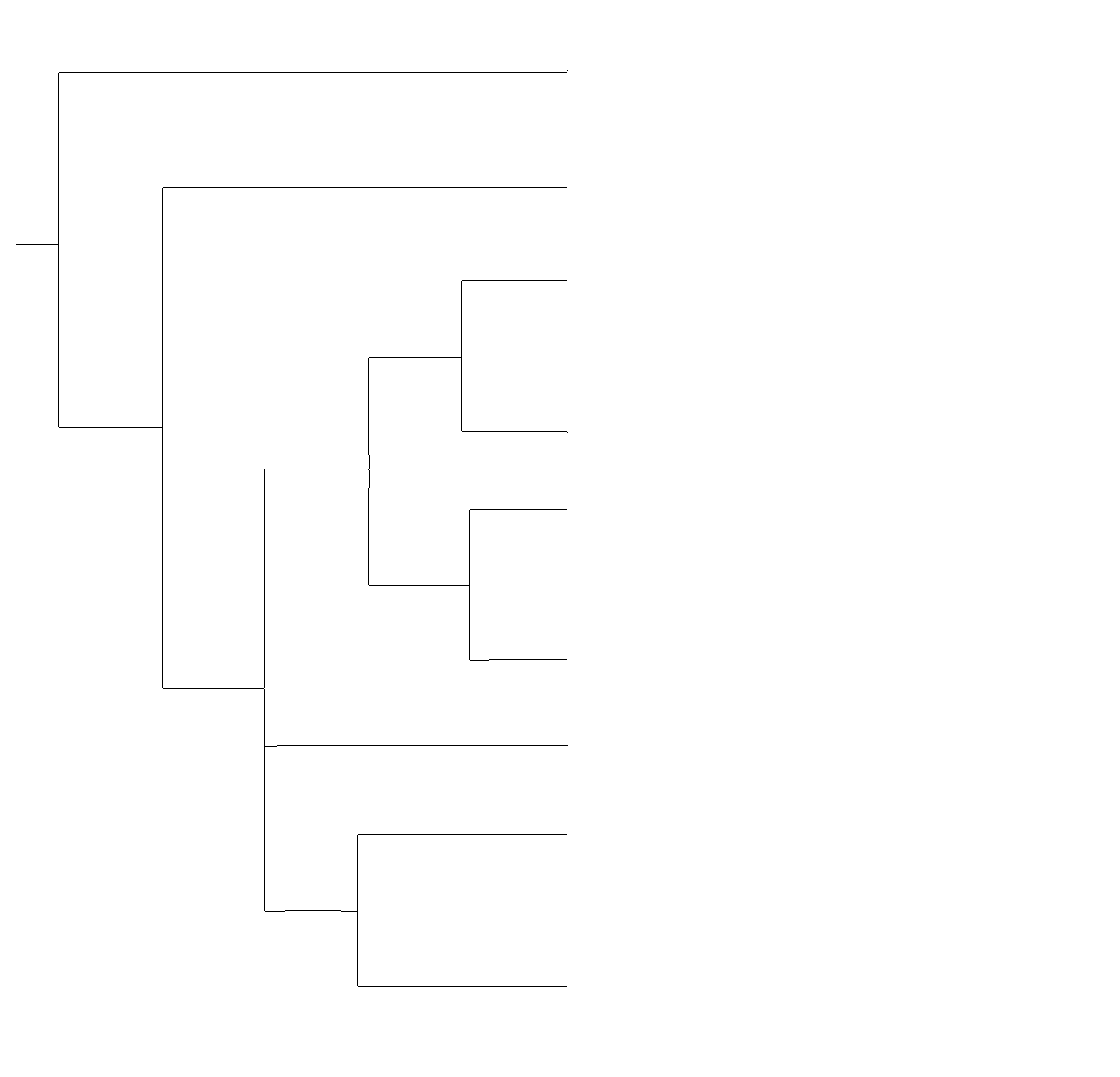

Supplement: Additional file 3 — ZIP files containing several folders, each of which with TreeSnatcher Plus snapshot files, the original image and a text file. [file 1471-2105-13-110-S3.zip › 1471-2148-8-160-1/1471-2148-8-160-1-l_c.PNG]

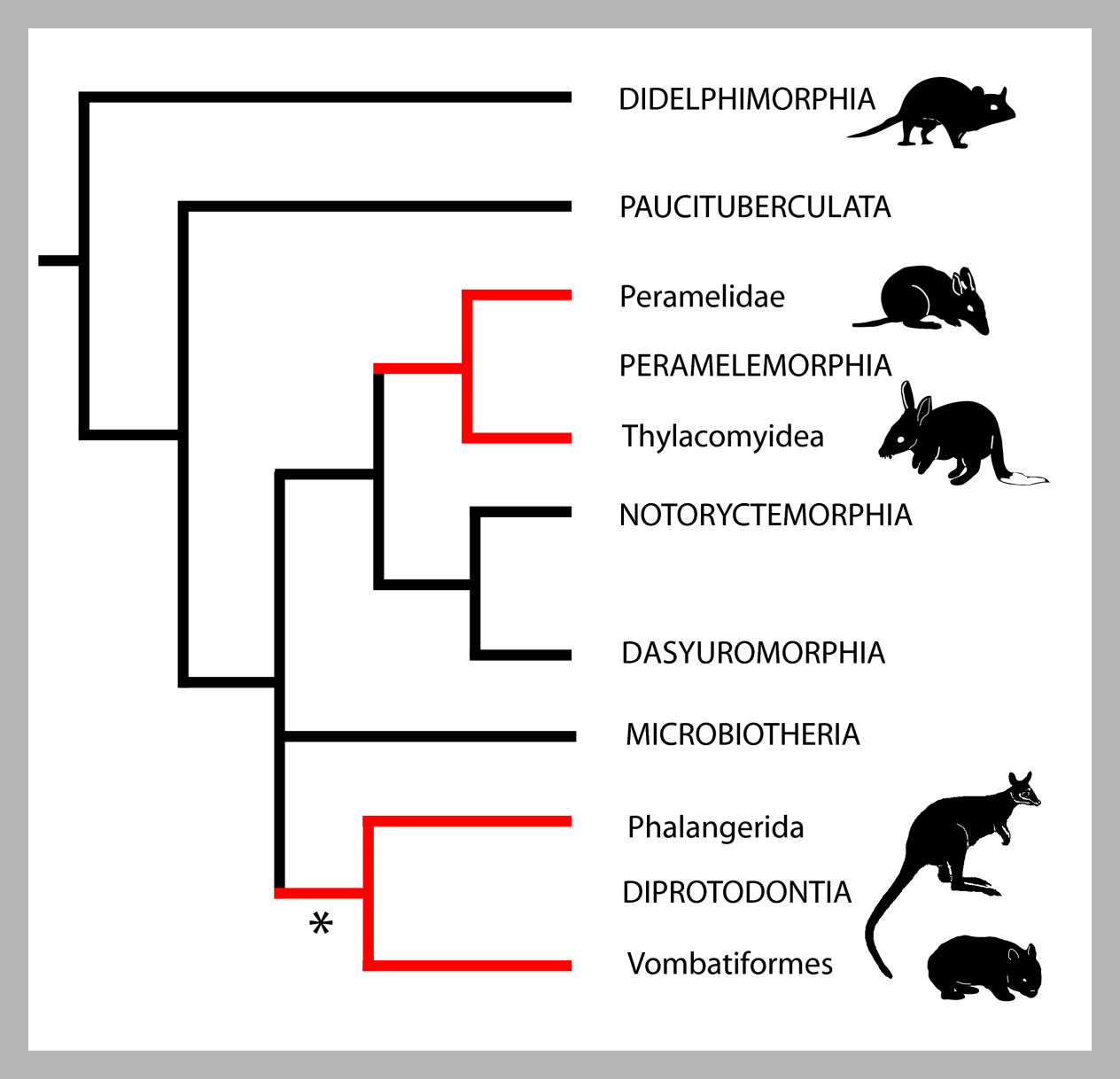

Supplement: Additional file 3 — ZIP files containing several folders, each of which with TreeSnatcher Plus snapshot files, the original image and a text file. [file 1471-2105-13-110-S3.zip › 1471-2148-8-160-1/1471-2148-8-160-1-l_o.PNG]

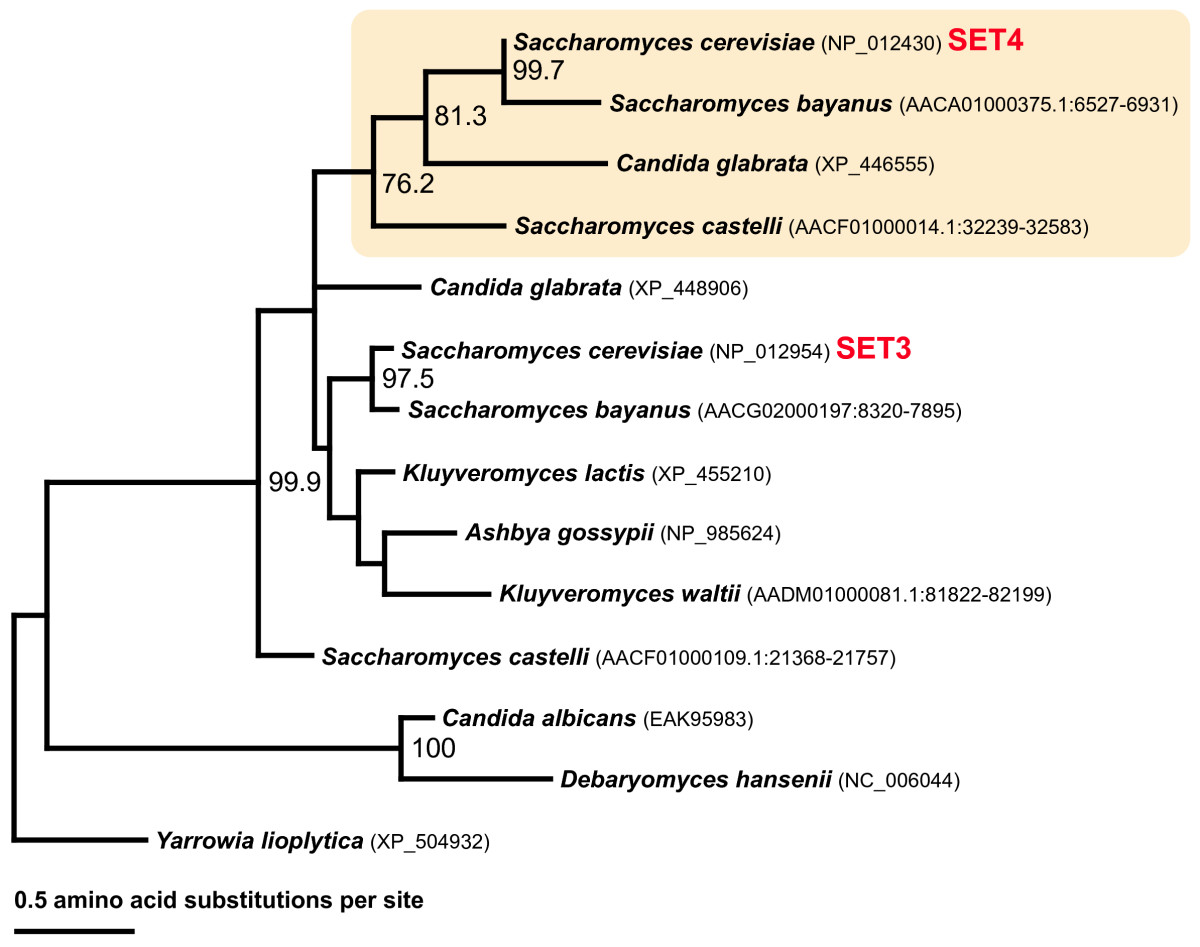

Supplement: Additional file 3 — ZIP files containing several folders, each of which with TreeSnatcher Plus snapshot files, the original image and a text file. [file 1471-2105-13-110-S3.zip › 1471-2148-8-190-6/1471-2148-8-190-6-l.jpg]

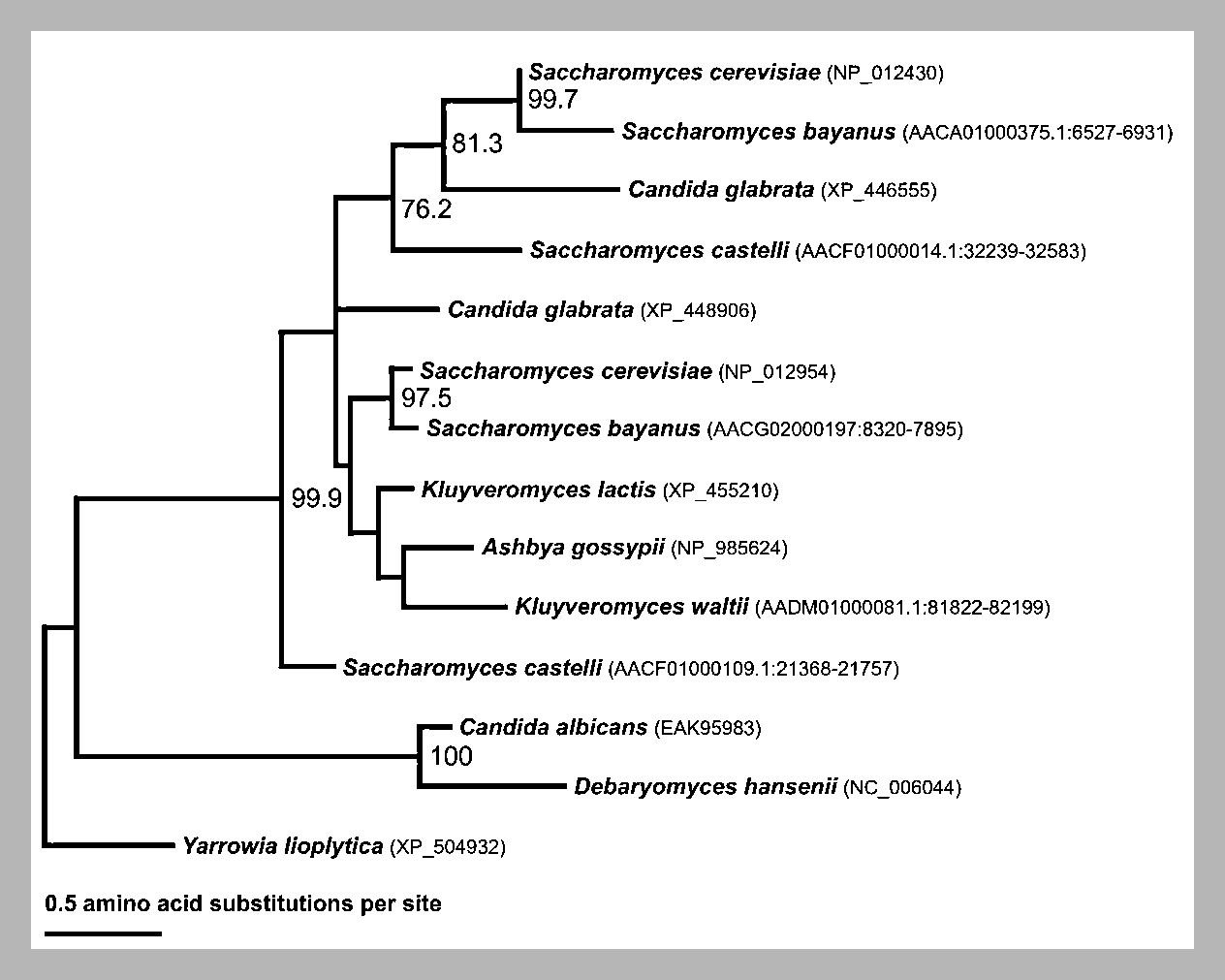

Supplement: Additional file 3 — ZIP files containing several folders, each of which with TreeSnatcher Plus snapshot files, the original image and a text file. [file 1471-2105-13-110-S3.zip › 1471-2148-8-190-6/1471-2148-8-190-6-l_b.PNG]

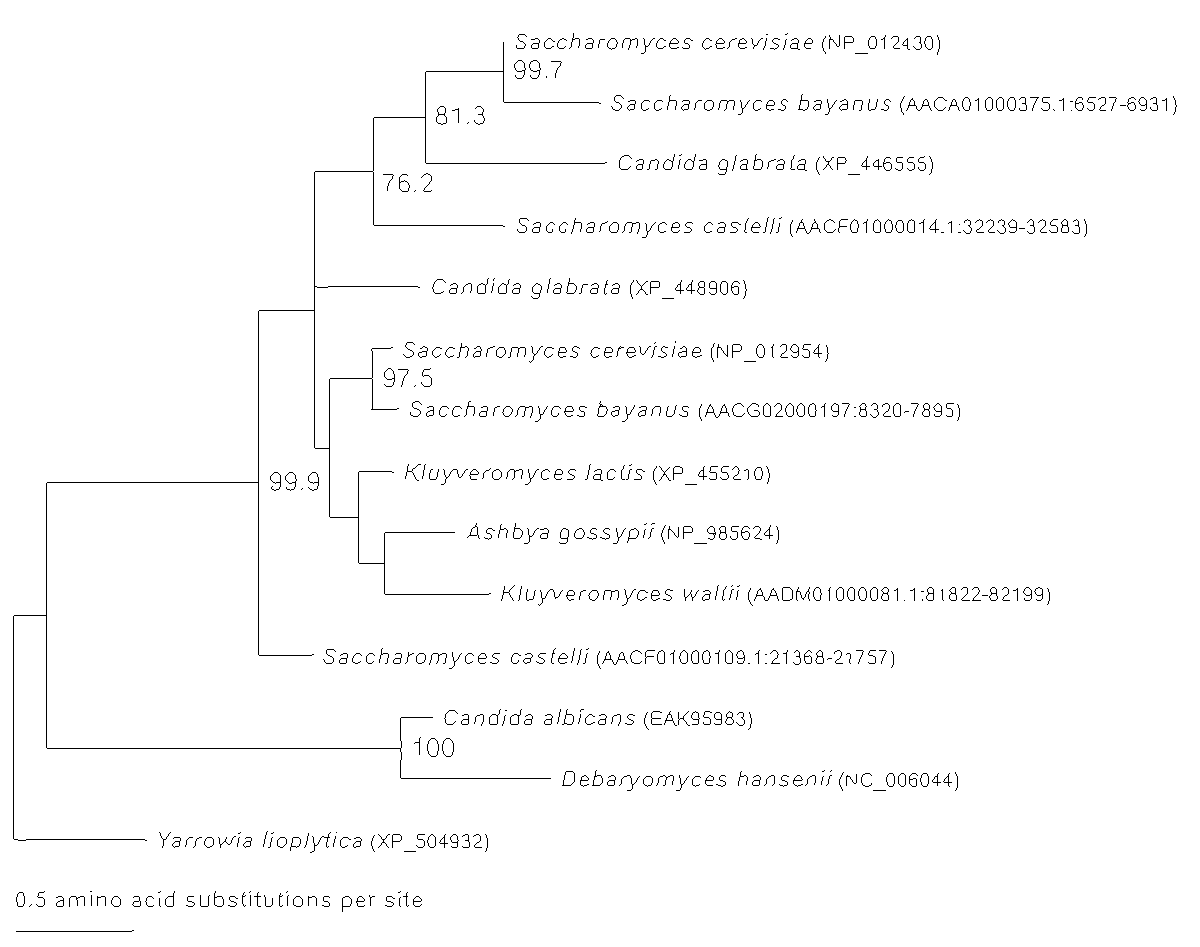

Supplement: Additional file 3 — ZIP files containing several folders, each of which with TreeSnatcher Plus snapshot files, the original image and a text file. [file 1471-2105-13-110-S3.zip › 1471-2148-8-190-6/1471-2148-8-190-6-l_c.PNG]

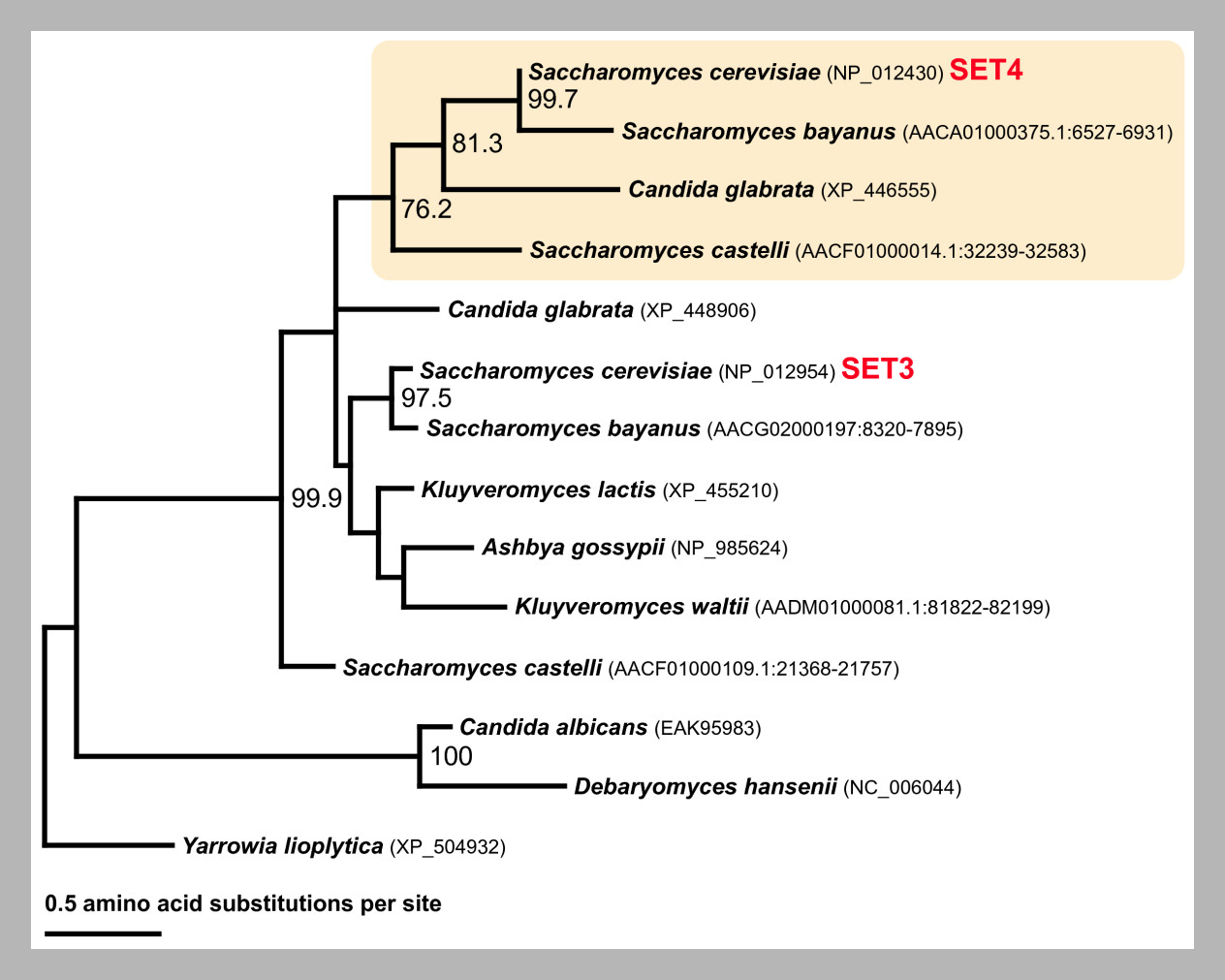

Supplement: Additional file 3 — ZIP files containing several folders, each of which with TreeSnatcher Plus snapshot files, the original image and a text file. [file 1471-2105-13-110-S3.zip › 1471-2148-8-190-6/1471-2148-8-190-6-l_o.PNG]

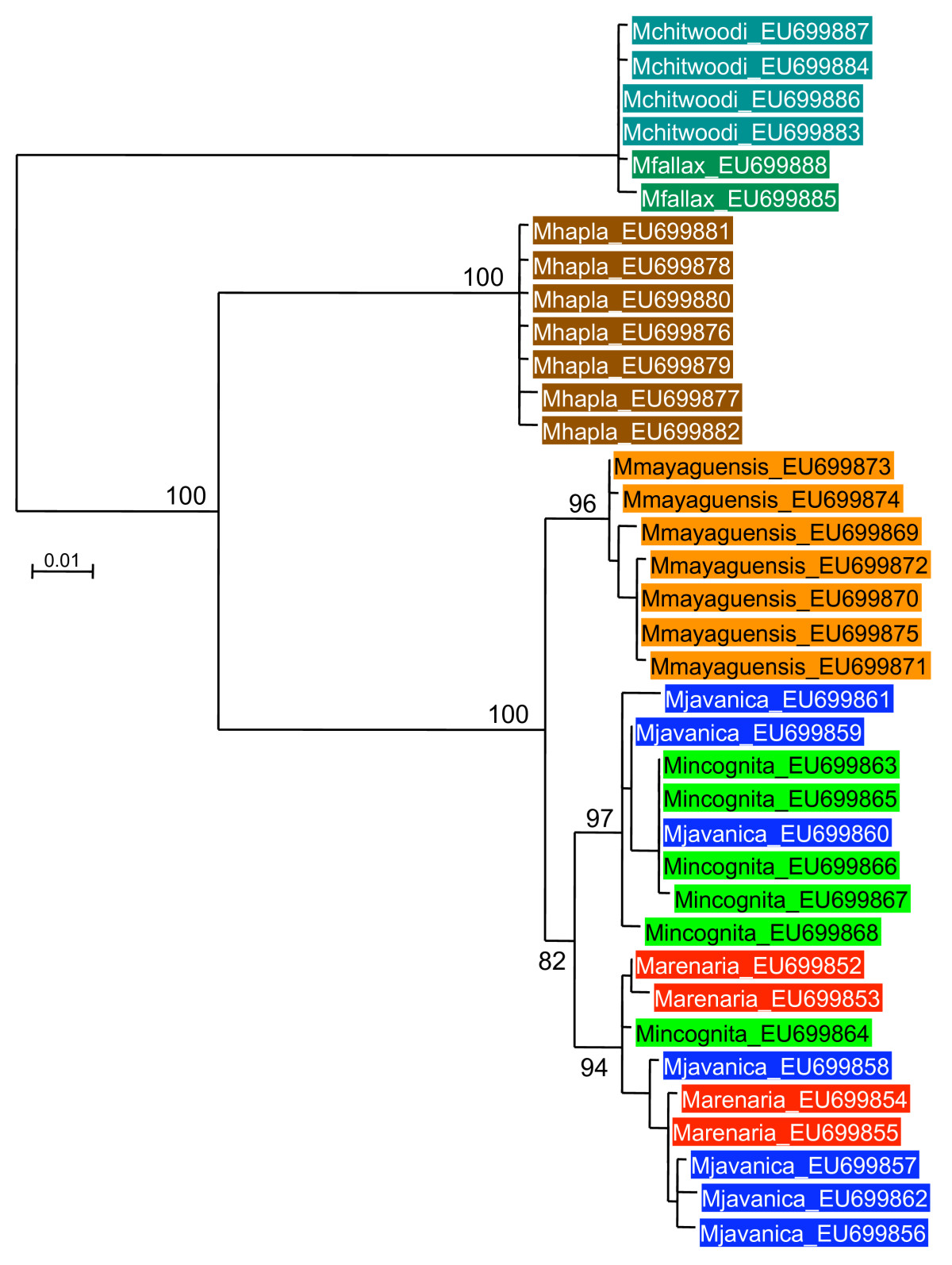

Supplement: Additional file 3 — ZIP files containing several folders, each of which with TreeSnatcher Plus snapshot files, the original image and a text file. [file 1471-2105-13-110-S3.zip › 1471-2148-8-194-2/1471-2148-8-194-2-l.jpg]

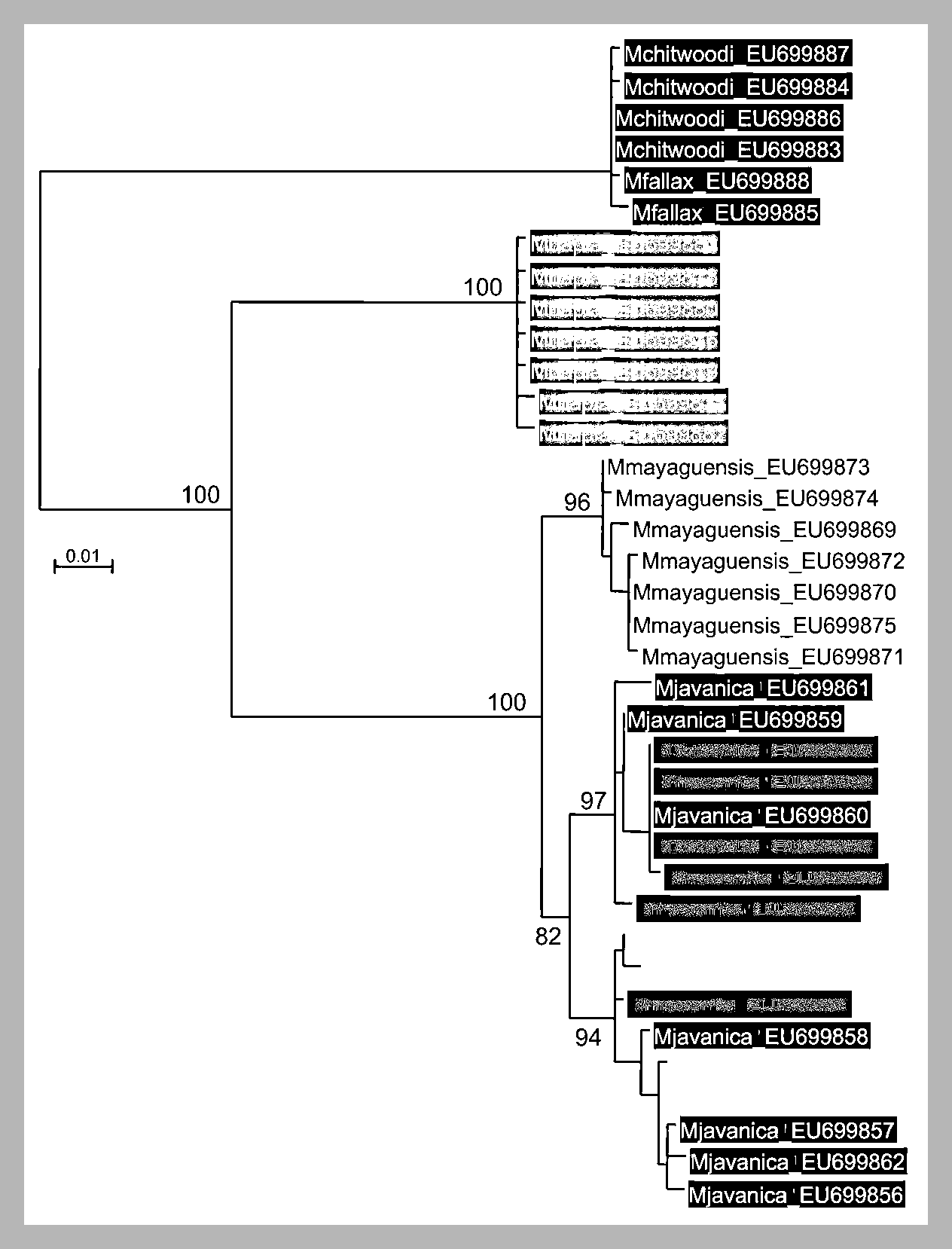

Supplement: Additional file 3 — ZIP files containing several folders, each of which with TreeSnatcher Plus snapshot files, the original image and a text file. [file 1471-2105-13-110-S3.zip › 1471-2148-8-194-2/1471-2148-8-194-2-l_b.PNG]

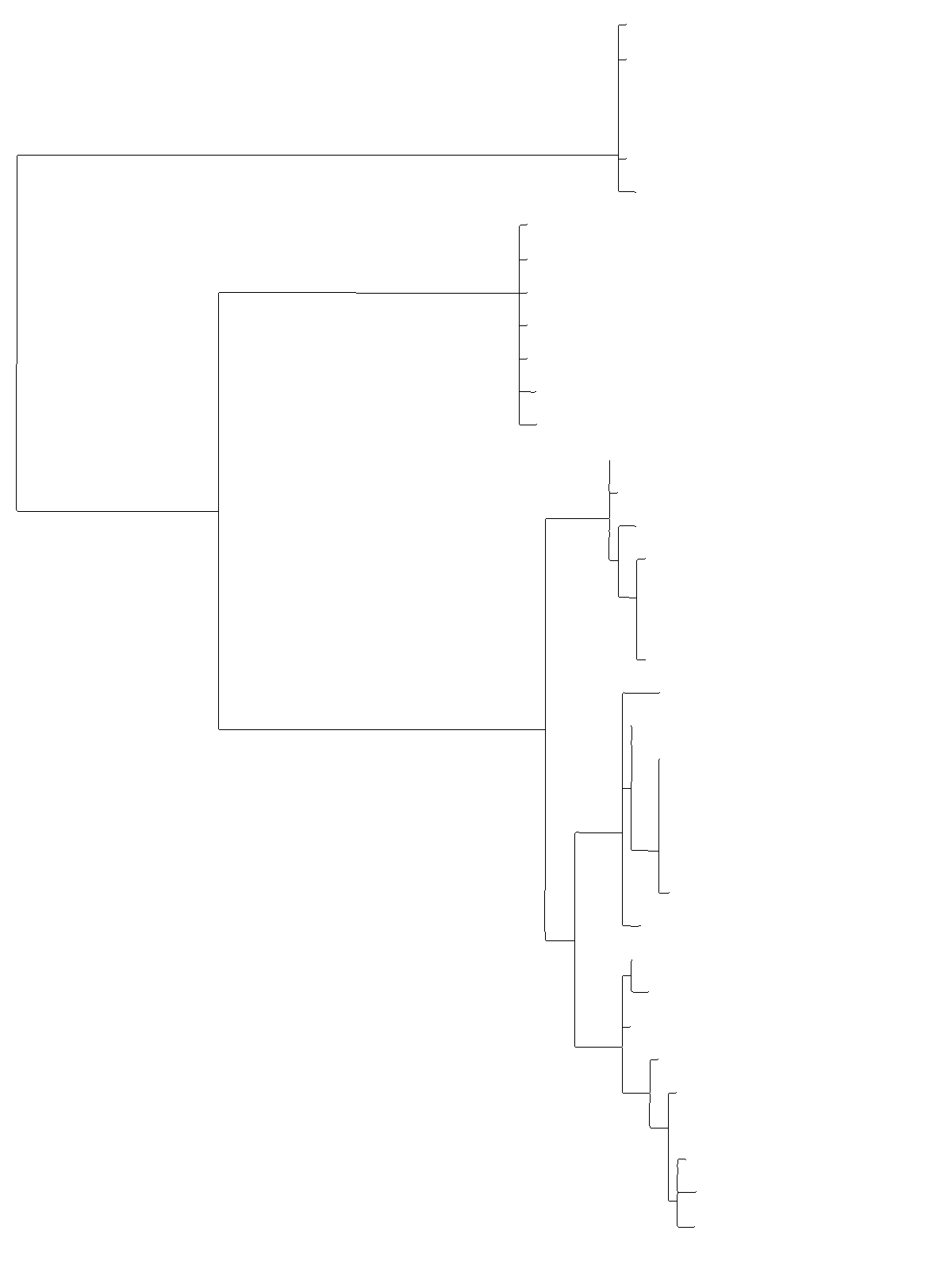

Supplement: Additional file 3 — ZIP files containing several folders, each of which with TreeSnatcher Plus snapshot files, the original image and a text file. [file 1471-2105-13-110-S3.zip › 1471-2148-8-194-2/1471-2148-8-194-2-l_c.PNG]

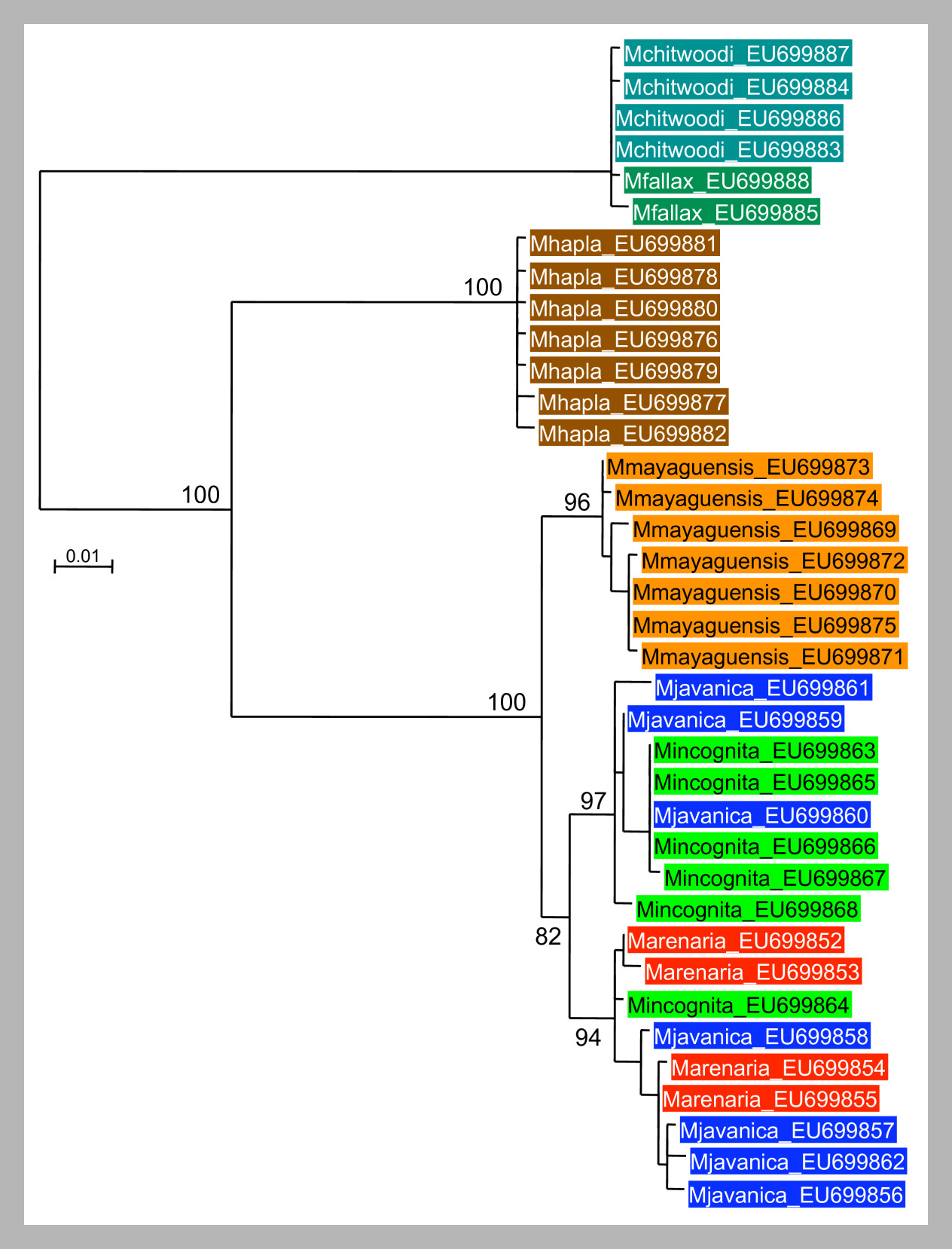

Supplement: Additional file 3 — ZIP files containing several folders, each of which with TreeSnatcher Plus snapshot files, the original image and a text file. [file 1471-2105-13-110-S3.zip › 1471-2148-8-194-2/1471-2148-8-194-2-l_o.PNG]

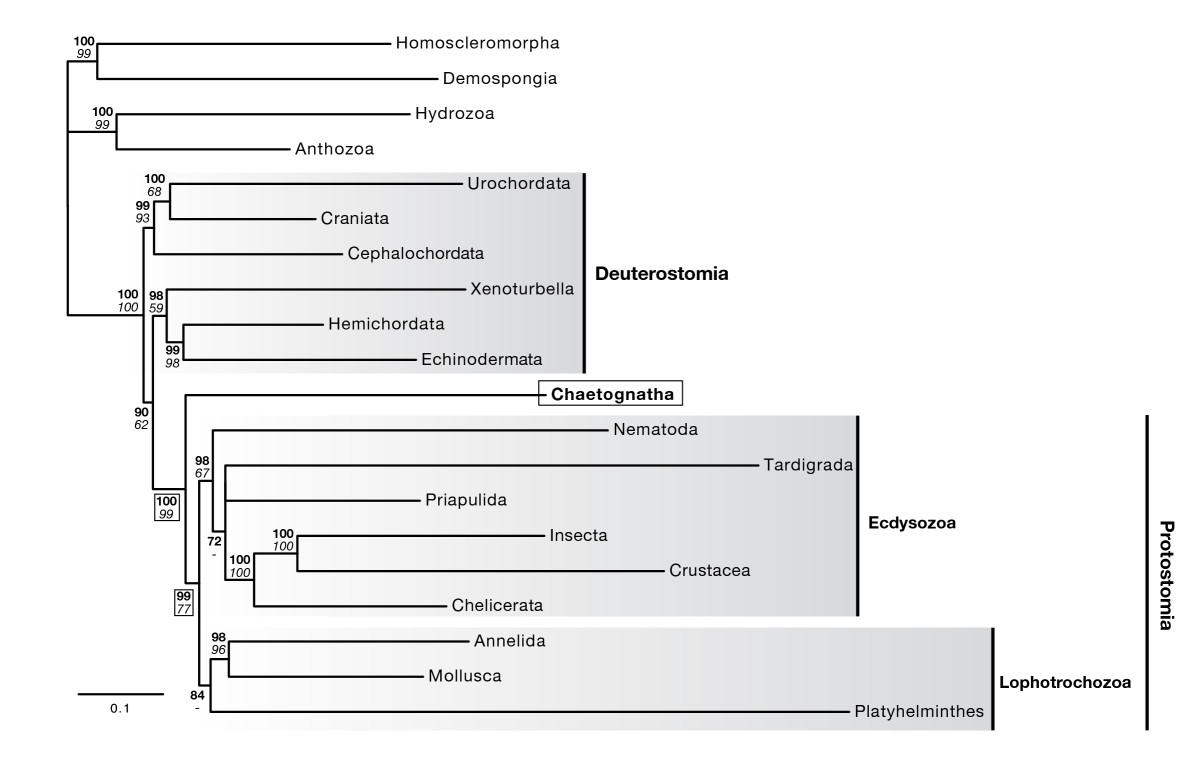

Supplement: Additional file 3 — ZIP files containing several folders, each of which with TreeSnatcher Plus snapshot files, the original image and a text file. [file 1471-2105-13-110-S3.zip › 1471-2148-8-251-2/1471-2148-8-251-2-l.jpg]

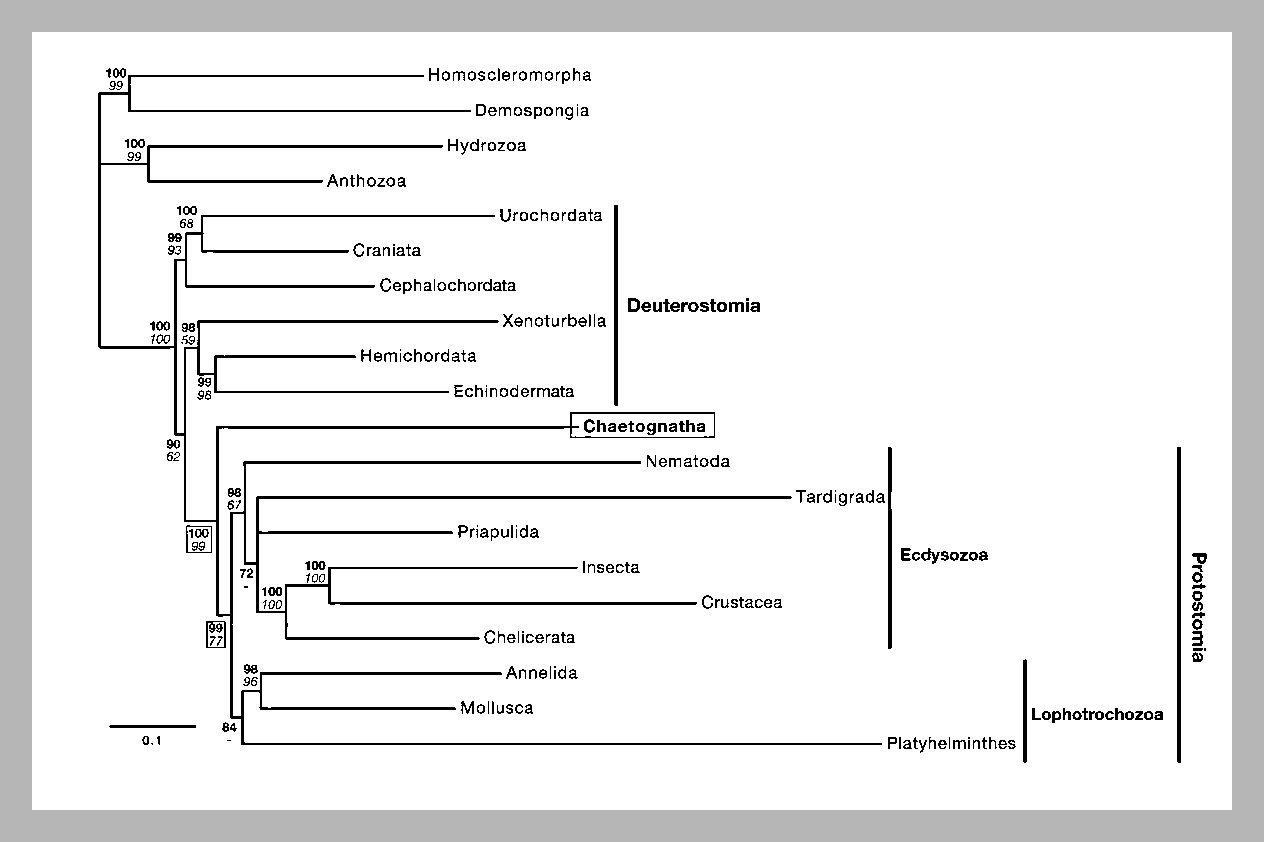

Supplement: Additional file 3 — ZIP files containing several folders, each of which with TreeSnatcher Plus snapshot files, the original image and a text file. [file 1471-2105-13-110-S3.zip › 1471-2148-8-251-2/1471-2148-8-251-2-l_b.PNG]

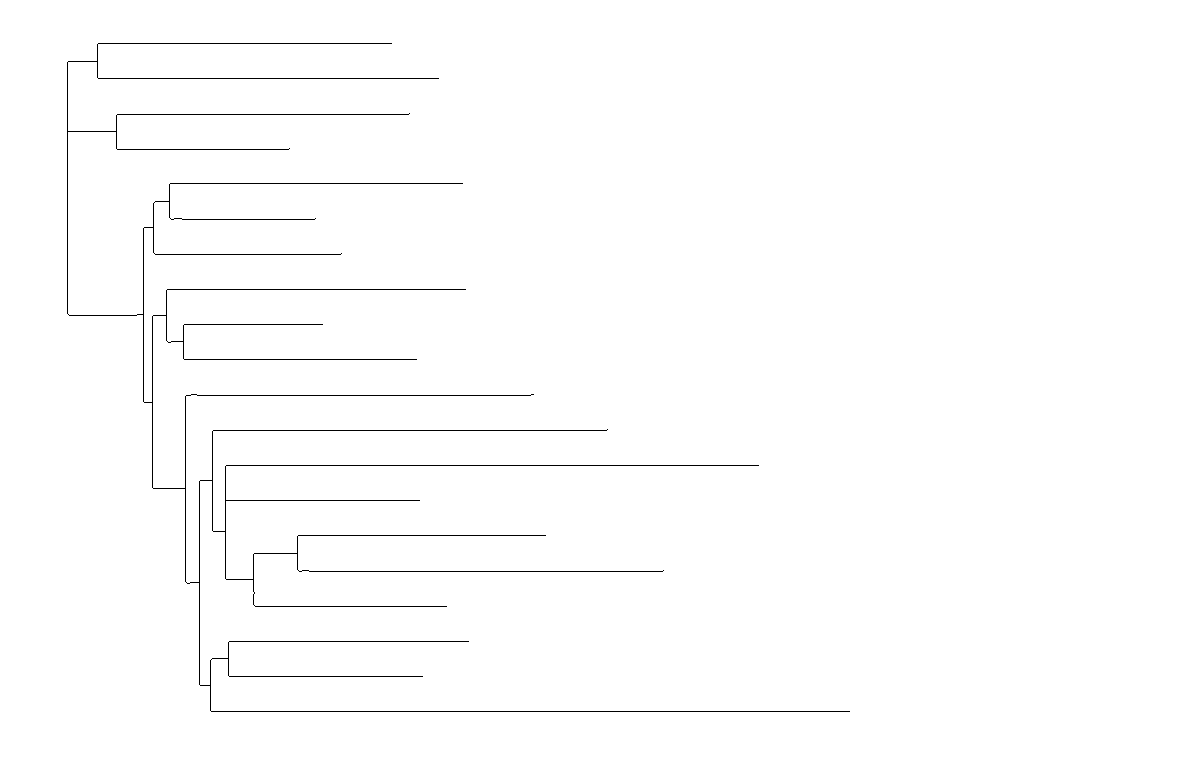

Supplement: Additional file 3 — ZIP files containing several folders, each of which with TreeSnatcher Plus snapshot files, the original image and a text file. [file 1471-2105-13-110-S3.zip › 1471-2148-8-251-2/1471-2148-8-251-2-l_c.PNG]

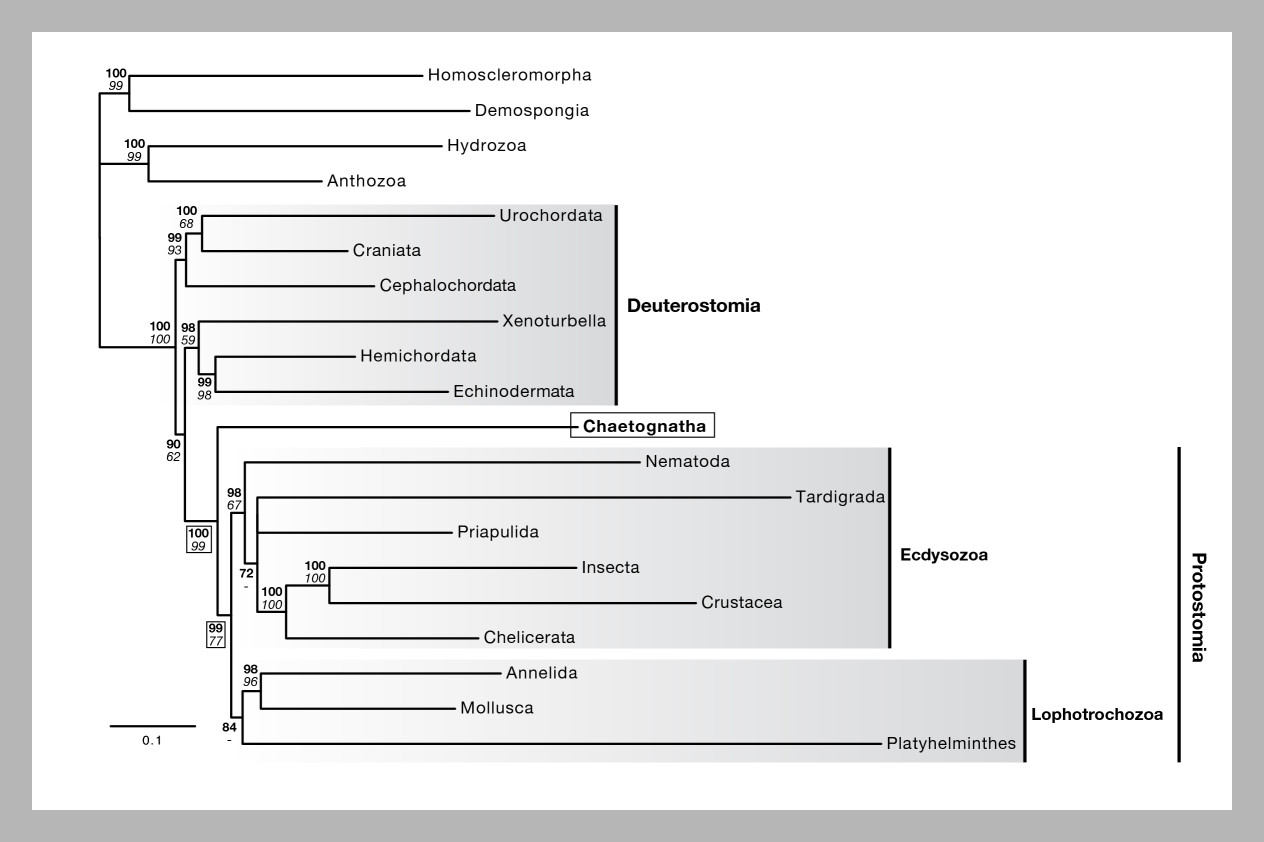

Supplement: Additional file 3 — ZIP files containing several folders, each of which with TreeSnatcher Plus snapshot files, the original image and a text file. [file 1471-2105-13-110-S3.zip › 1471-2148-8-251-2/1471-2148-8-251-2-l_o.PNG]

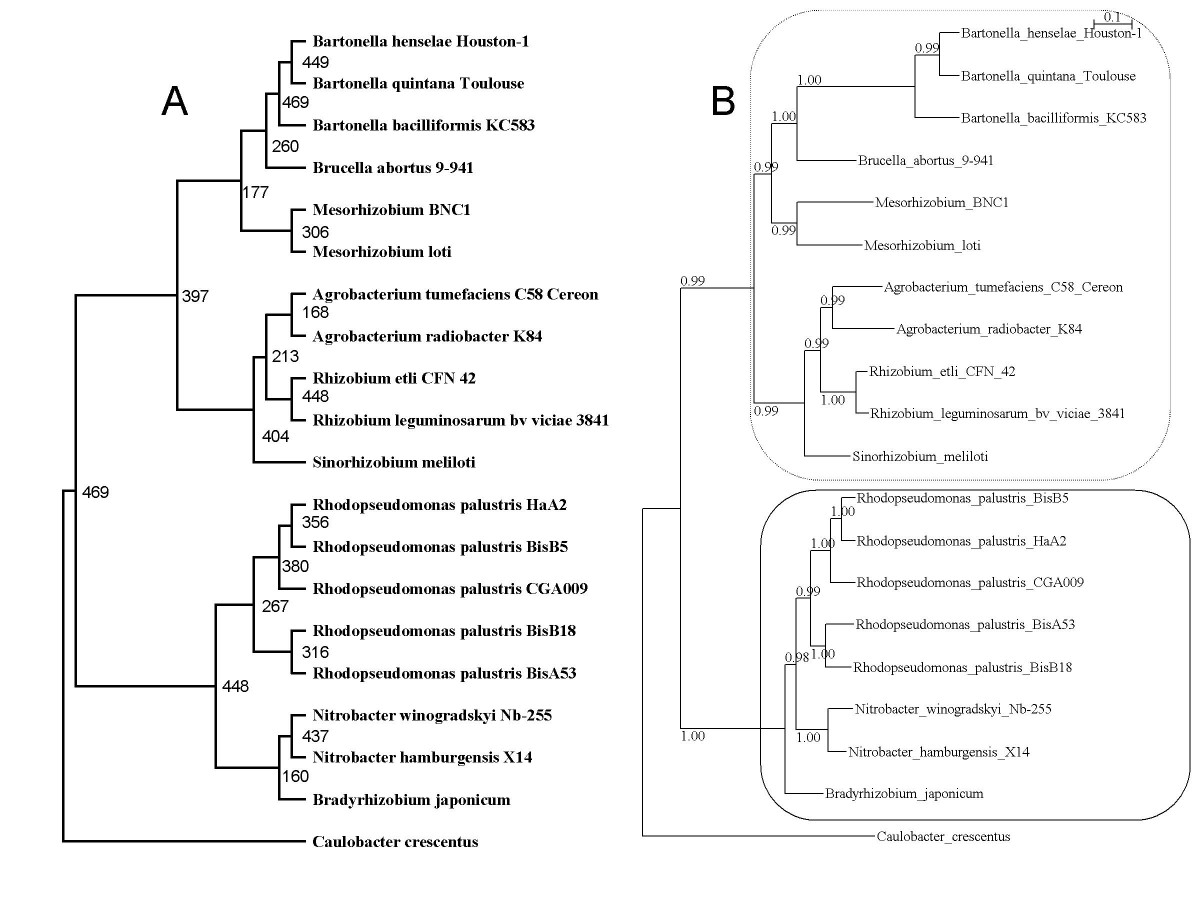

Supplement: Additional file 3 — ZIP files containing several folders, each of which with TreeSnatcher Plus snapshot files, the original image and a text file. [file 1471-2105-13-110-S3.zip › 1471-2148-8-300-1/1471-2148-8-300-1-l.jpg]

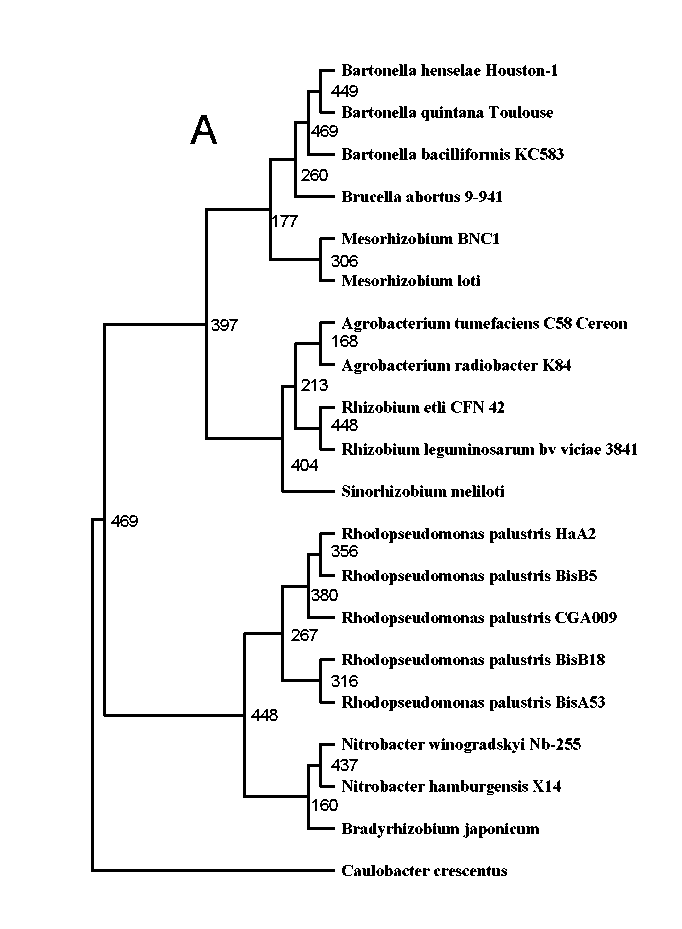

Supplement: Additional file 3 — ZIP files containing several folders, each of which with TreeSnatcher Plus snapshot files, the original image and a text file. [file 1471-2105-13-110-S3.zip › 1471-2148-8-300-1/1471-2148-8-300-1-l_b.PNG]

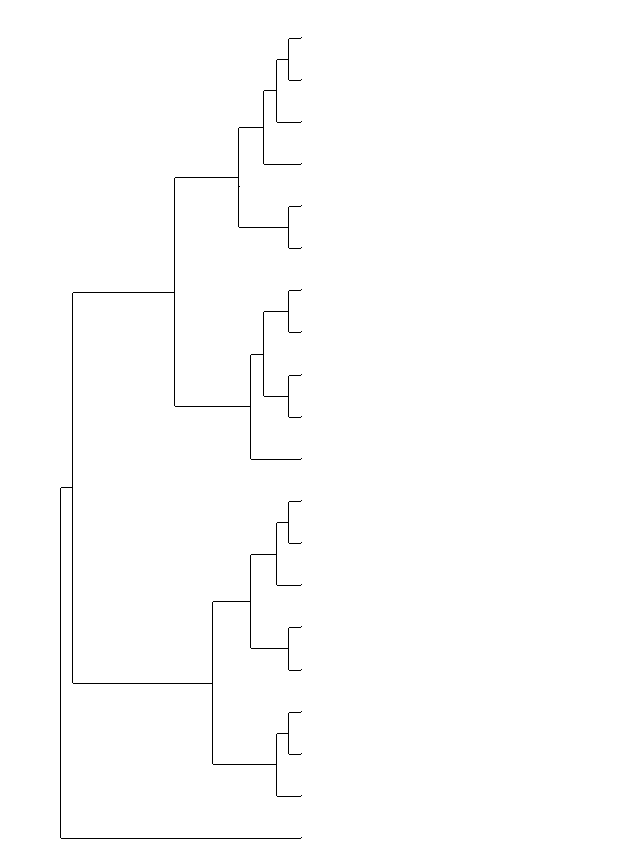

Supplement: Additional file 3 — ZIP files containing several folders, each of which with TreeSnatcher Plus snapshot files, the original image and a text file. [file 1471-2105-13-110-S3.zip › 1471-2148-8-300-1/1471-2148-8-300-1-l_c.PNG]

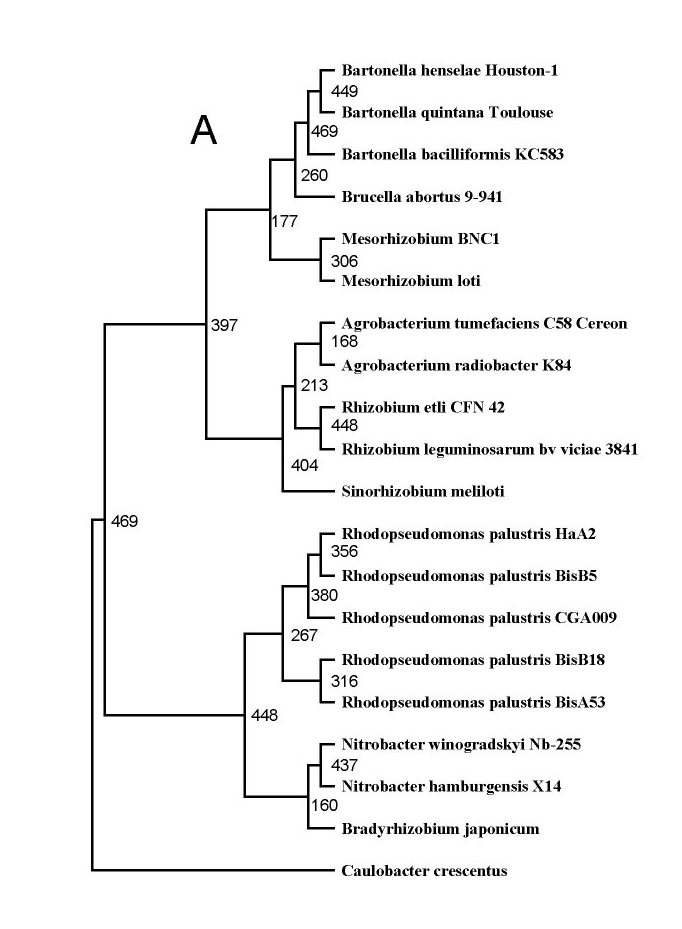

Supplement: Additional file 3 — ZIP files containing several folders, each of which with TreeSnatcher Plus snapshot files, the original image and a text file. [file 1471-2105-13-110-S3.zip › 1471-2148-8-300-1/1471-2148-8-300-1-l_o.PNG]

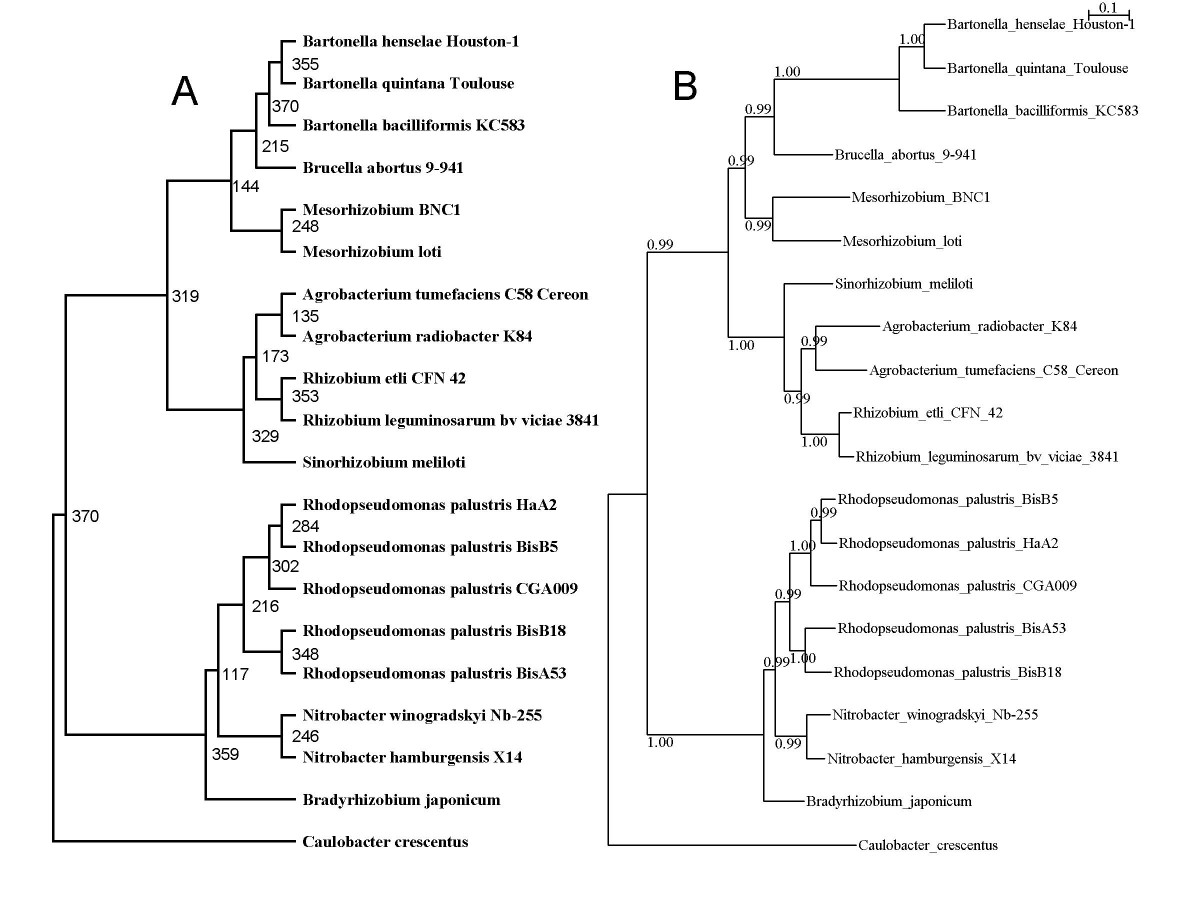

Supplement: Additional file 3 — ZIP files containing several folders, each of which with TreeSnatcher Plus snapshot files, the original image and a text file. [file 1471-2105-13-110-S3.zip › 1471-2148-8-300-2/1471-2148-8-300-2-l.jpg]

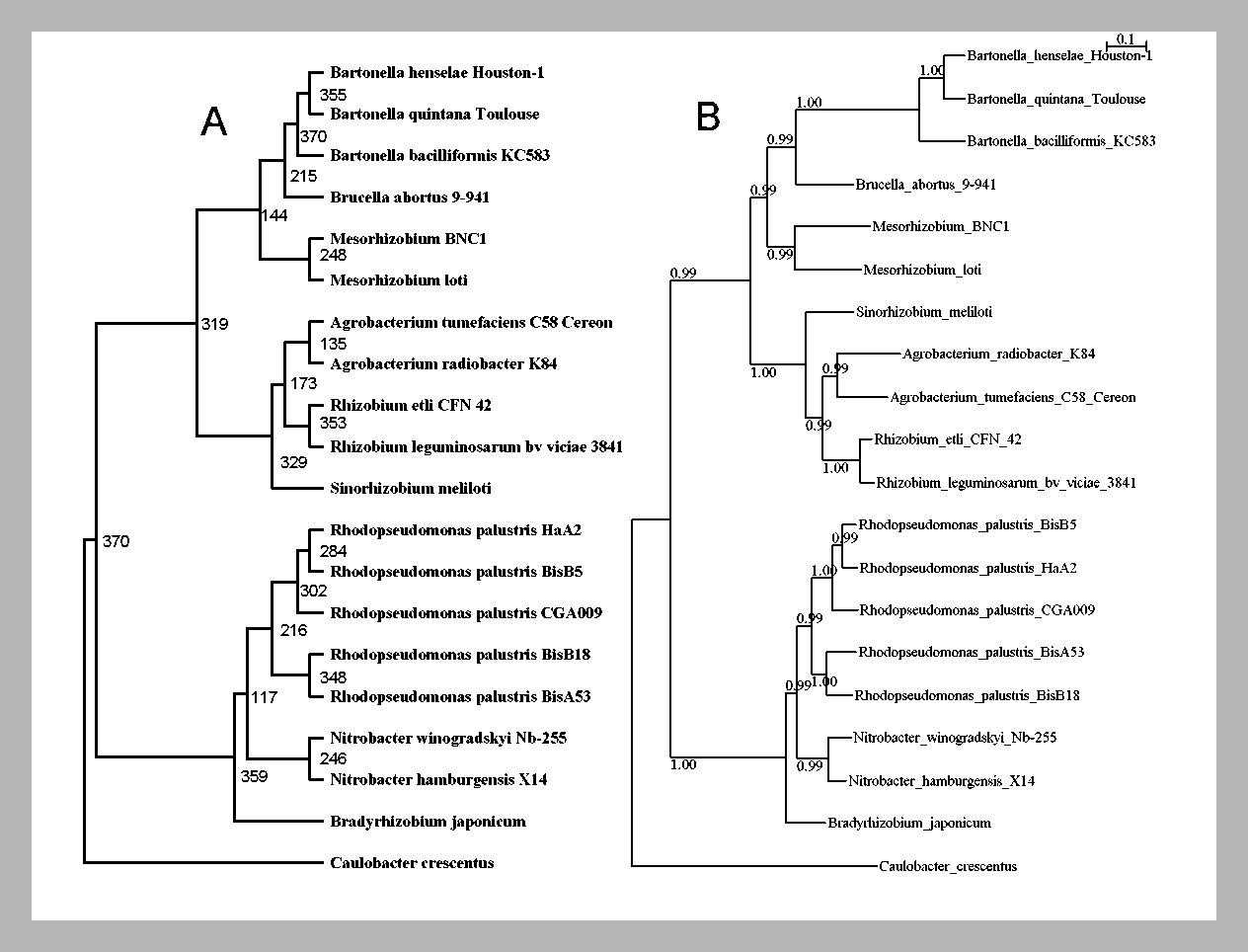

Supplement: Additional file 3 — ZIP files containing several folders, each of which with TreeSnatcher Plus snapshot files, the original image and a text file. [file 1471-2105-13-110-S3.zip › 1471-2148-8-300-2/1471-2148-8-300-2-l_b.PNG]

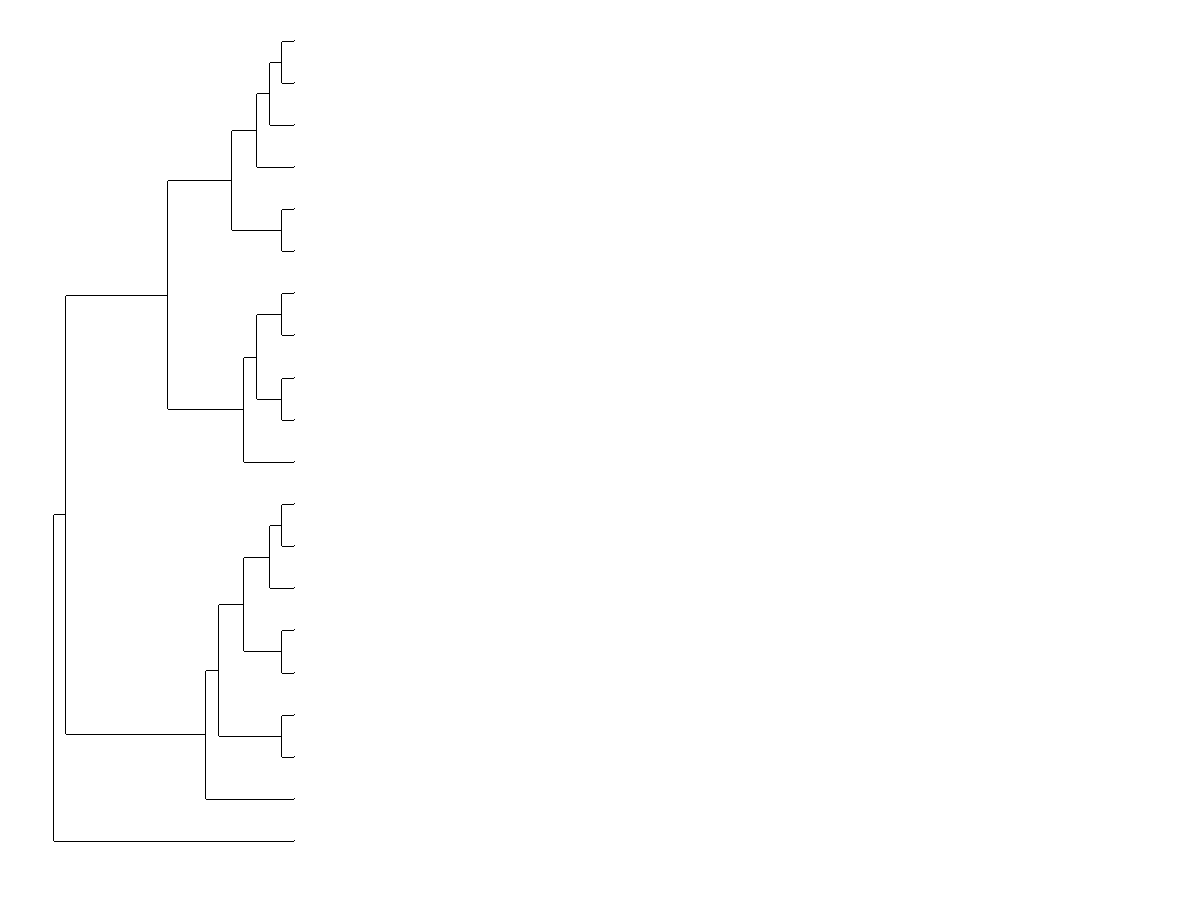

Supplement: Additional file 3 — ZIP files containing several folders, each of which with TreeSnatcher Plus snapshot files, the original image and a text file. [file 1471-2105-13-110-S3.zip › 1471-2148-8-300-2/1471-2148-8-300-2-l_c.PNG]

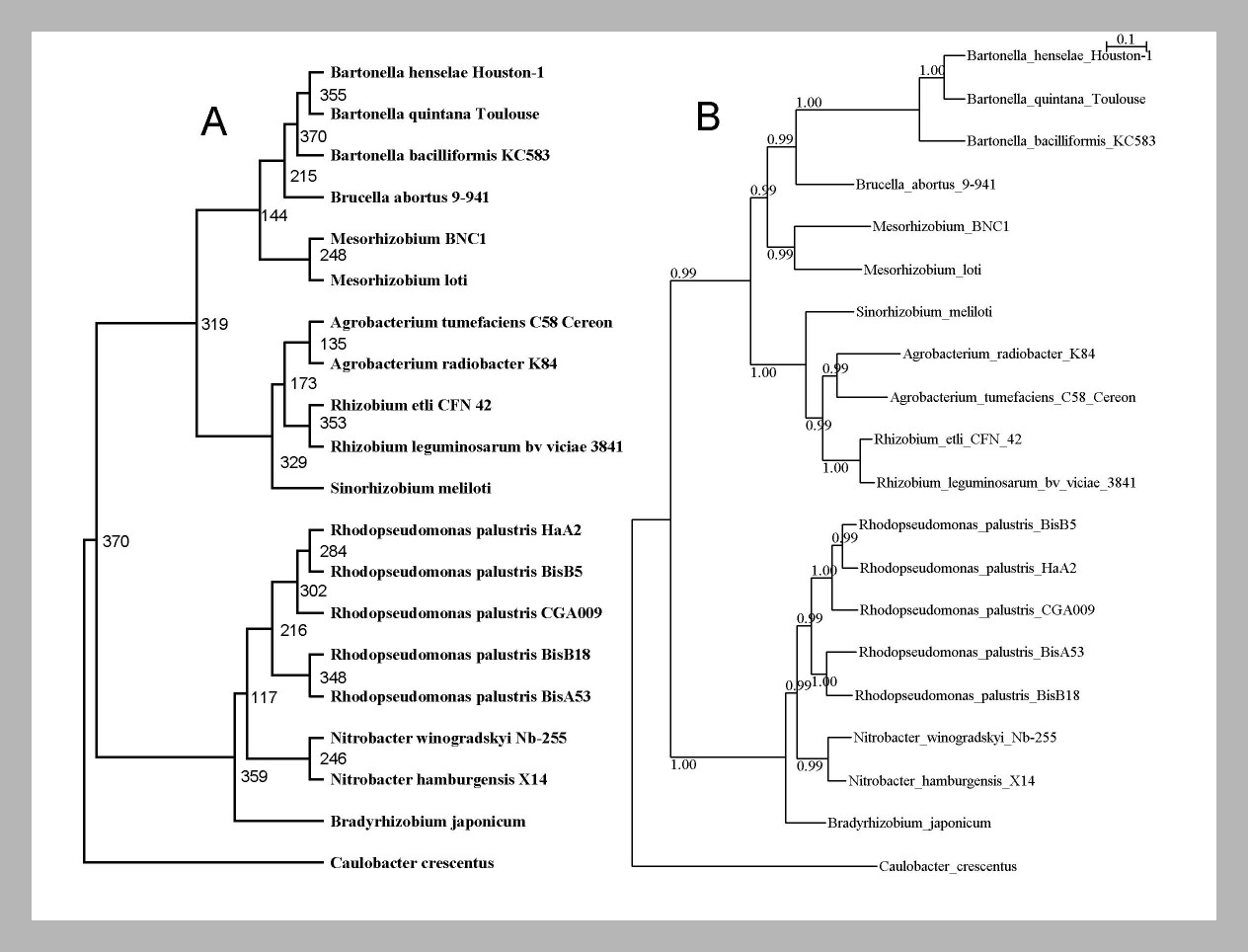

Supplement: Additional file 3 — ZIP files containing several folders, each of which with TreeSnatcher Plus snapshot files, the original image and a text file. [file 1471-2105-13-110-S3.zip › 1471-2148-8-300-2/1471-2148-8-300-2-l_o.PNG]

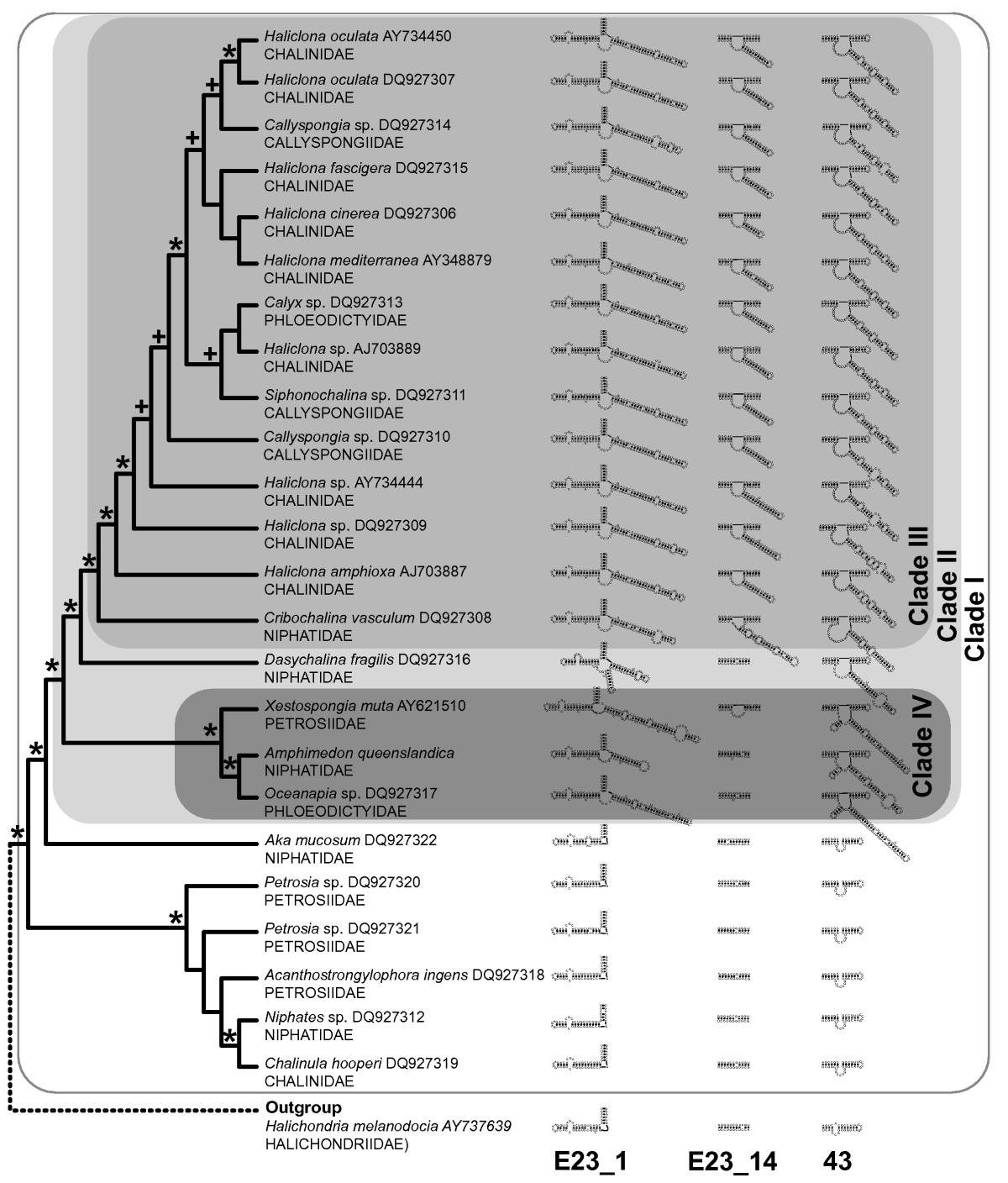

Supplement: Additional file 3 — ZIP files containing several folders, each of which with TreeSnatcher Plus snapshot files, the original image and a text file. [file 1471-2105-13-110-S3.zip › 1471-2148-8-69-6/1471-2148-8-69-6-l.jpg]

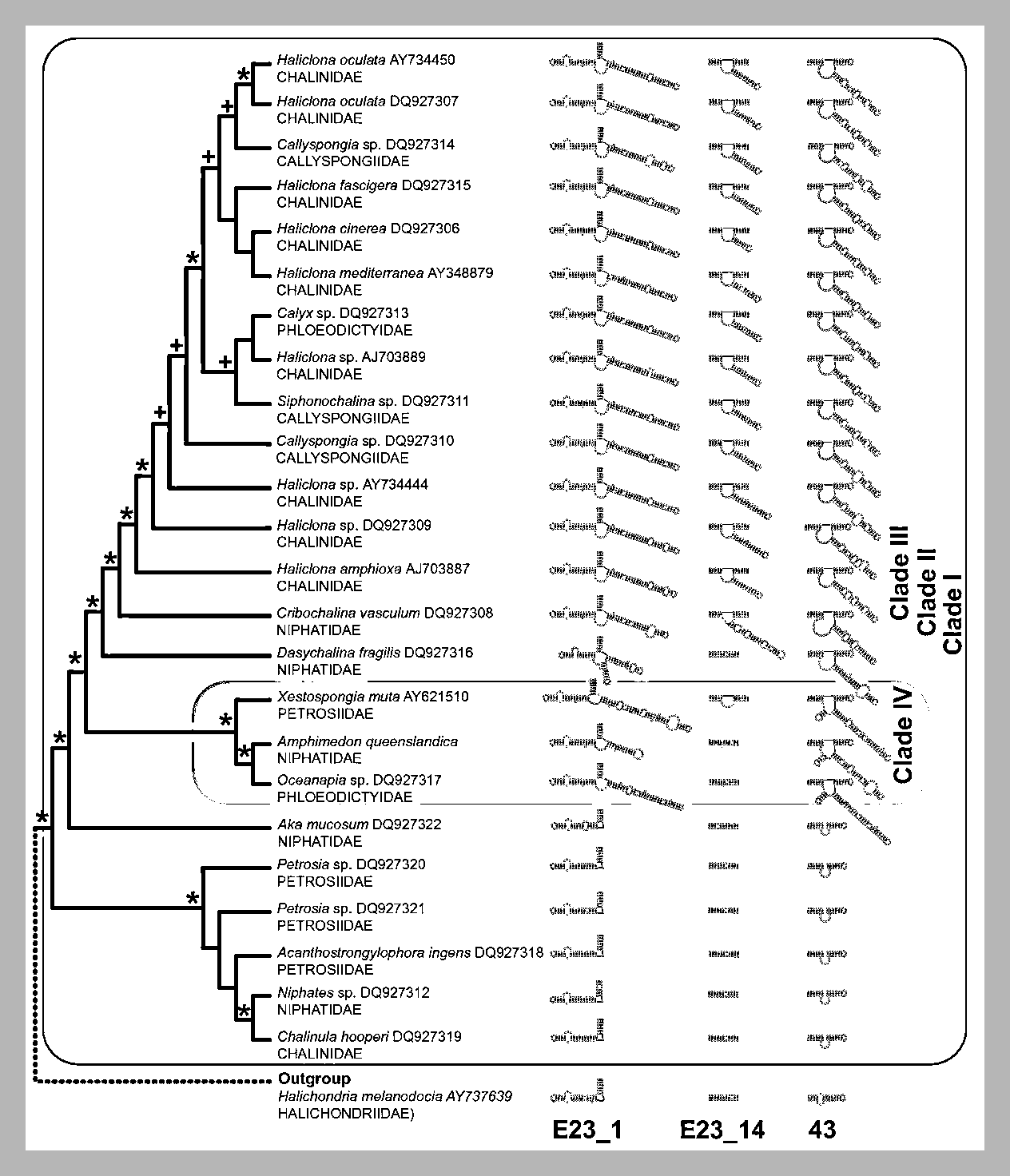

Supplement: Additional file 3 — ZIP files containing several folders, each of which with TreeSnatcher Plus snapshot files, the original image and a text file. [file 1471-2105-13-110-S3.zip › 1471-2148-8-69-6/1471-2148-8-69-6-l_b.PNG]

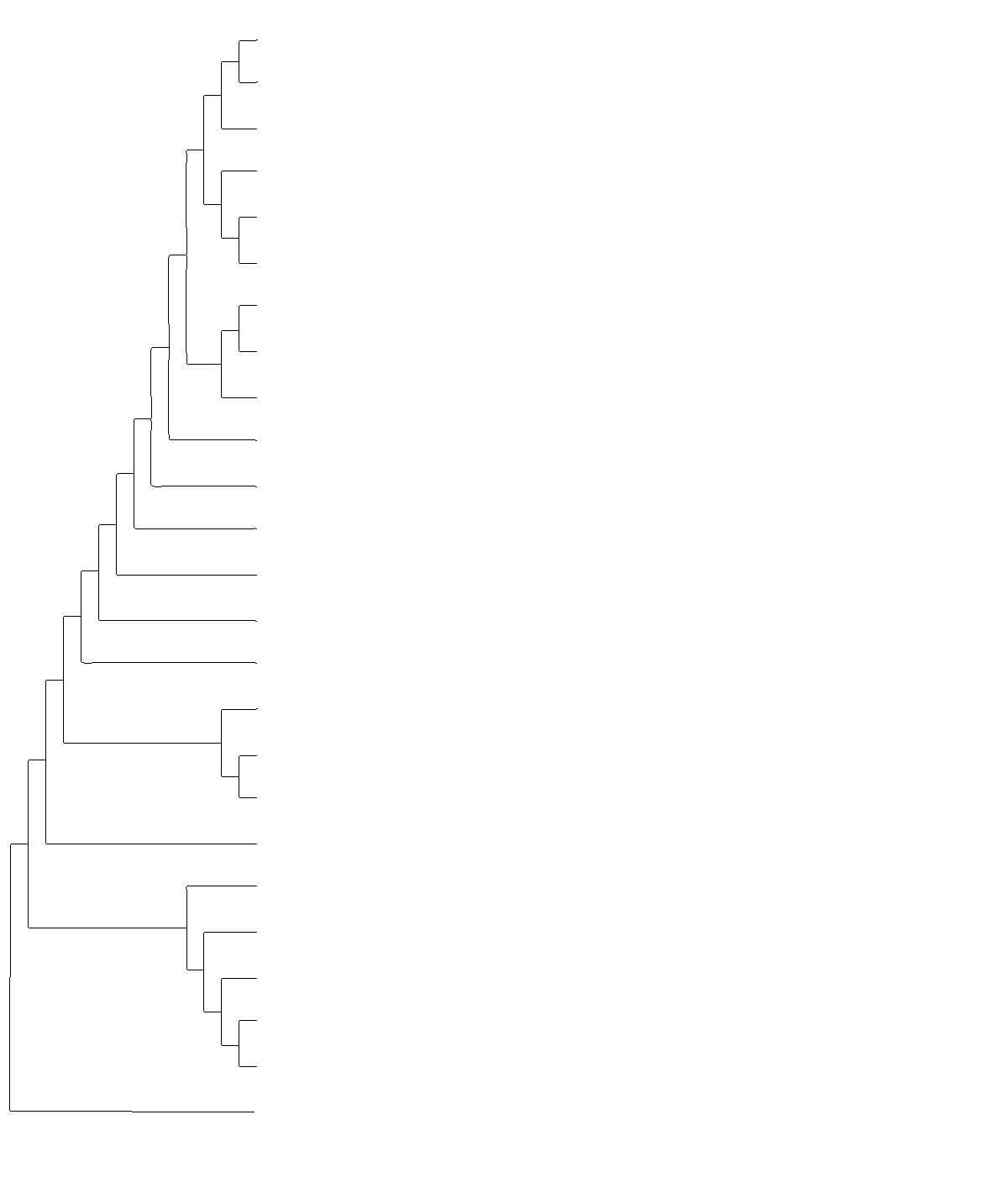

Supplement: Additional file 3 — ZIP files containing several folders, each of which with TreeSnatcher Plus snapshot files, the original image and a text file. [file 1471-2105-13-110-S3.zip › 1471-2148-8-69-6/1471-2148-8-69-6-l_c.PNG]

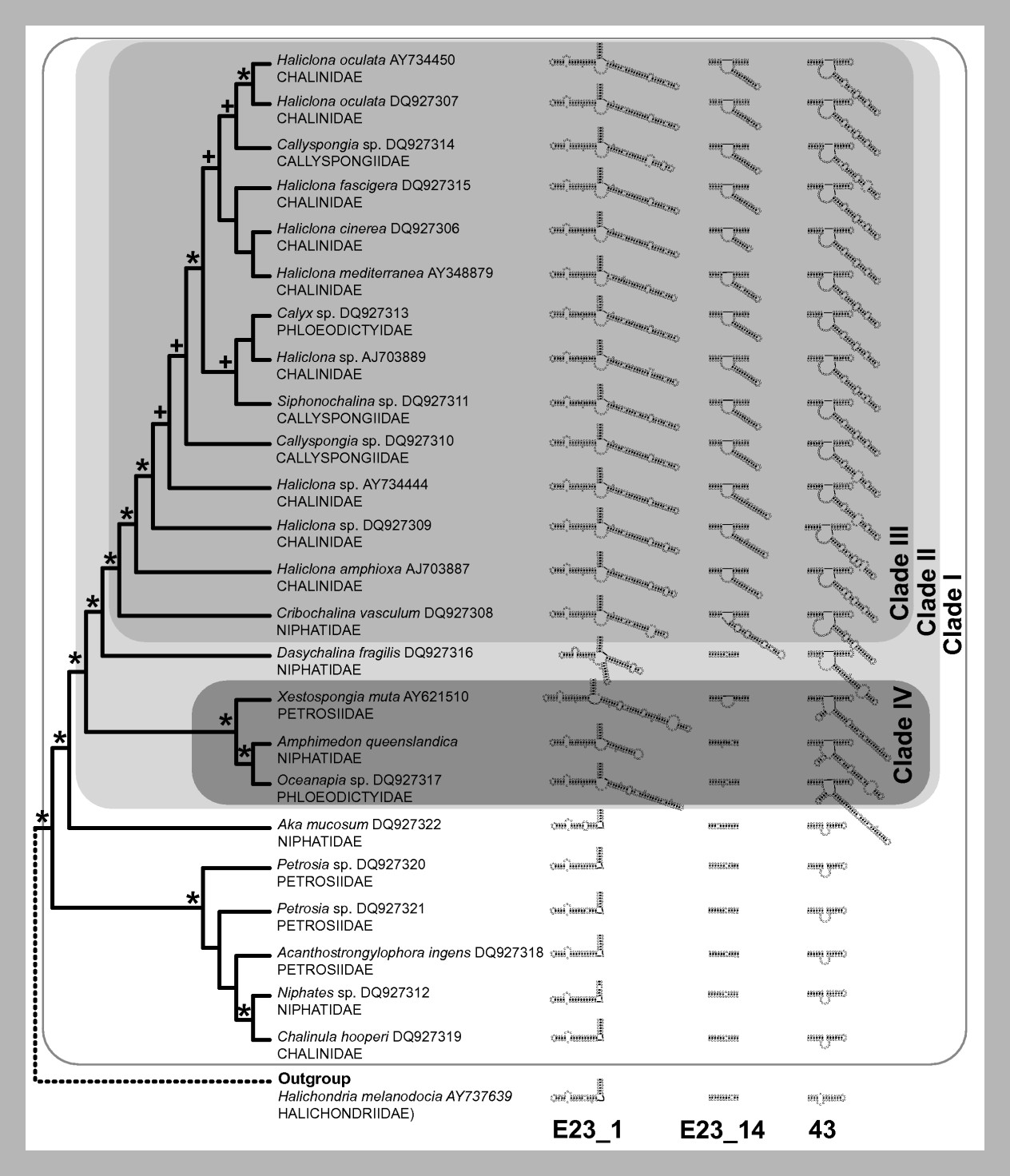

Supplement: Additional file 3 — ZIP files containing several folders, each of which with TreeSnatcher Plus snapshot files, the original image and a text file. [file 1471-2105-13-110-S3.zip › 1471-2148-8-69-6/1471-2148-8-69-6-l_o.PNG]

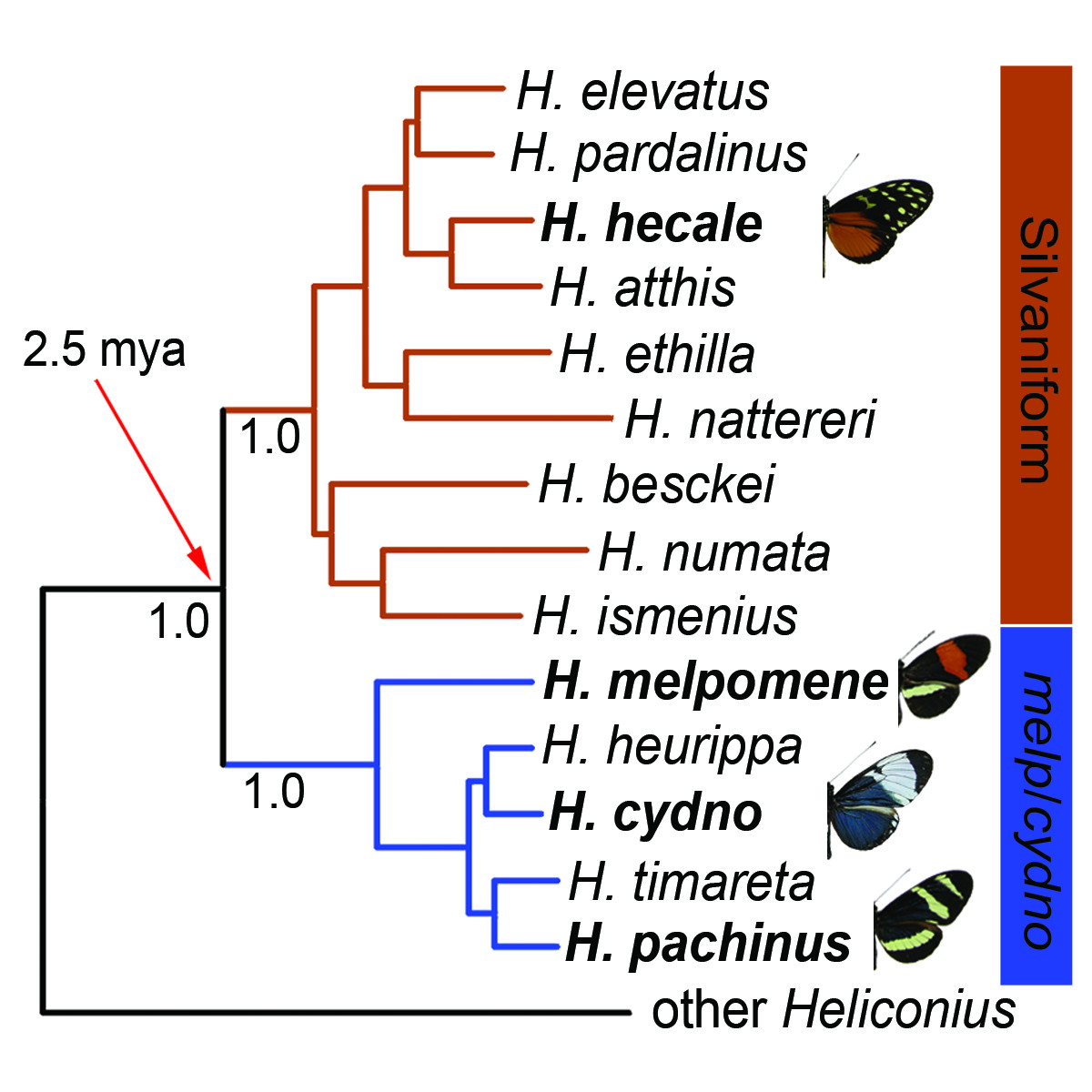

Supplement: Additional file 3 — ZIP files containing several folders, each of which with TreeSnatcher Plus snapshot files, the original image and a text file. [file 1471-2105-13-110-S3.zip › 1471-2148-8-98-1/1471-2148-8-98-1-l.jpg]

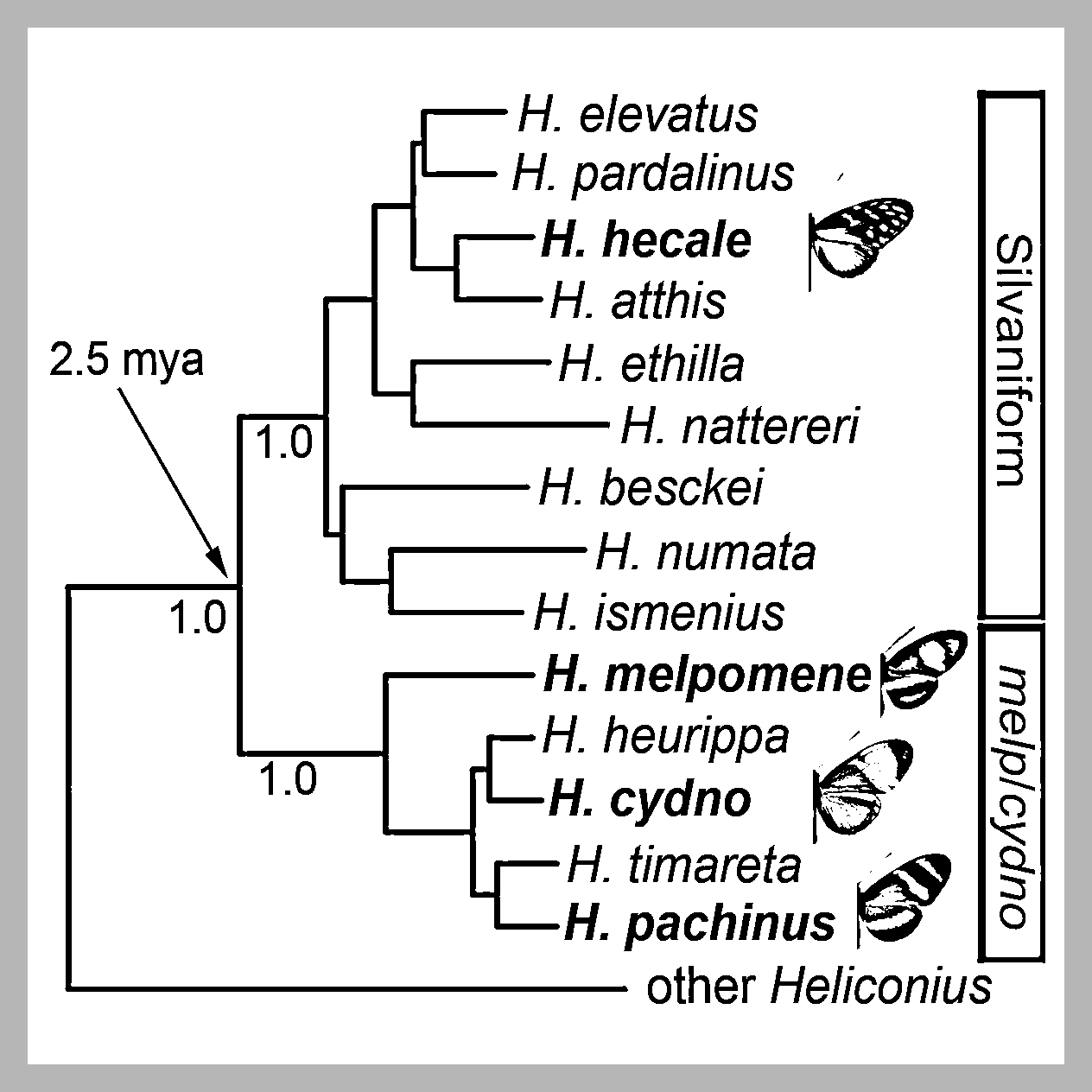

Supplement: Additional file 3 — ZIP files containing several folders, each of which with TreeSnatcher Plus snapshot files, the original image and a text file. [file 1471-2105-13-110-S3.zip › 1471-2148-8-98-1/1471-2148-8-98-1_b.PNG]

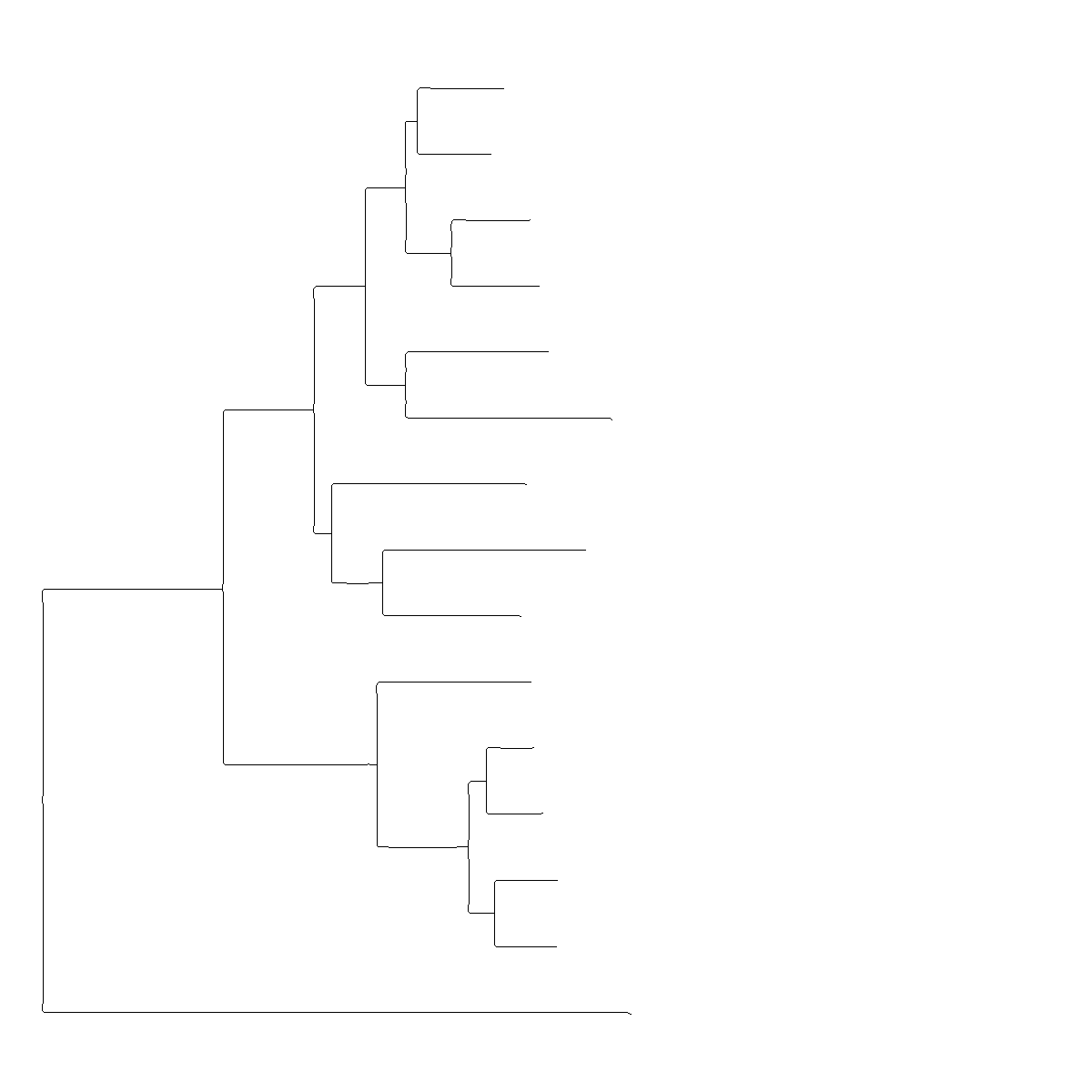

Supplement: Additional file 3 — ZIP files containing several folders, each of which with TreeSnatcher Plus snapshot files, the original image and a text file. [file 1471-2105-13-110-S3.zip › 1471-2148-8-98-1/1471-2148-8-98-1_c.PNG]

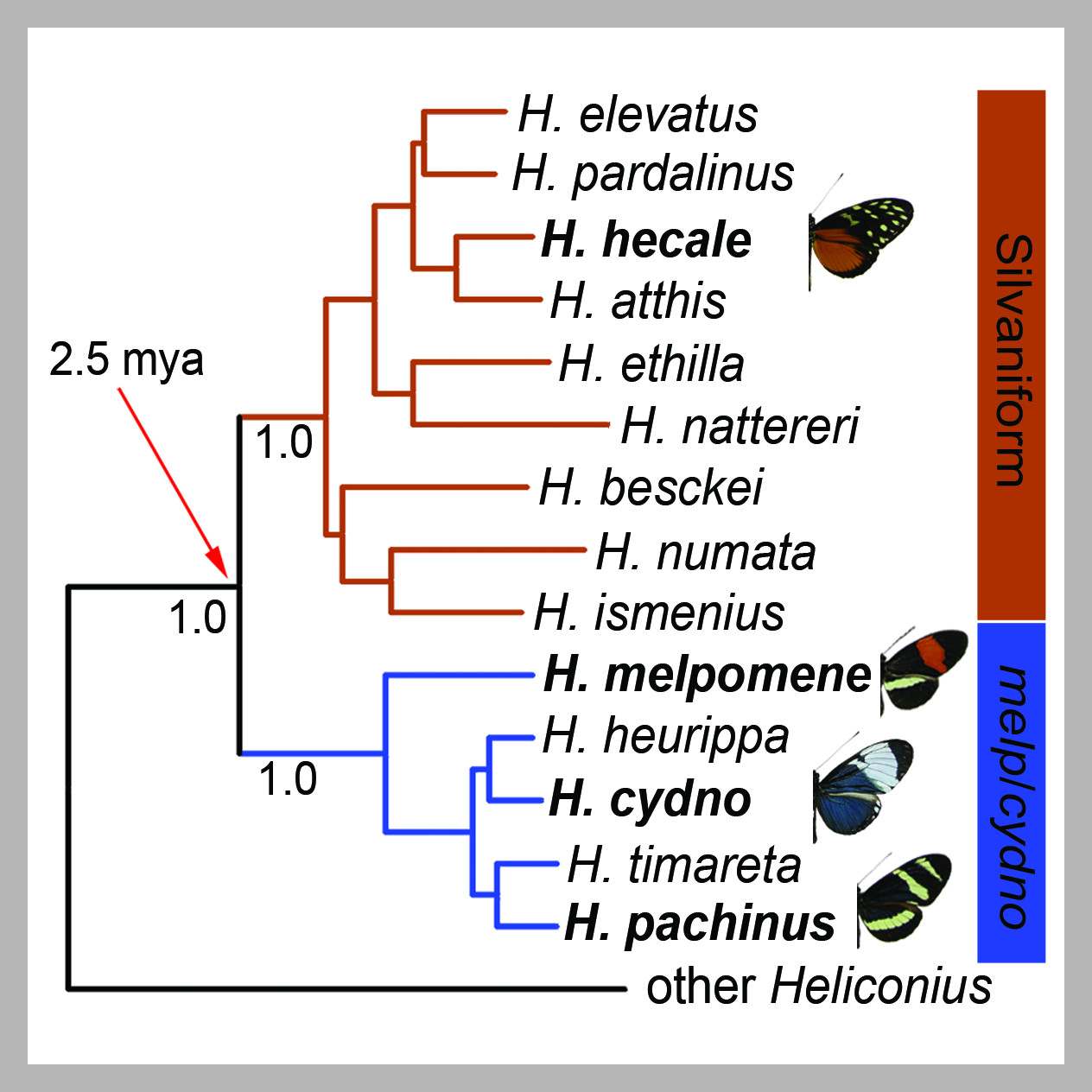

Supplement: Additional file 3 — ZIP files containing several folders, each of which with TreeSnatcher Plus snapshot files, the original image and a text file. [file 1471-2105-13-110-S3.zip › 1471-2148-8-98-1/1471-2148-8-98-1_o.PNG]

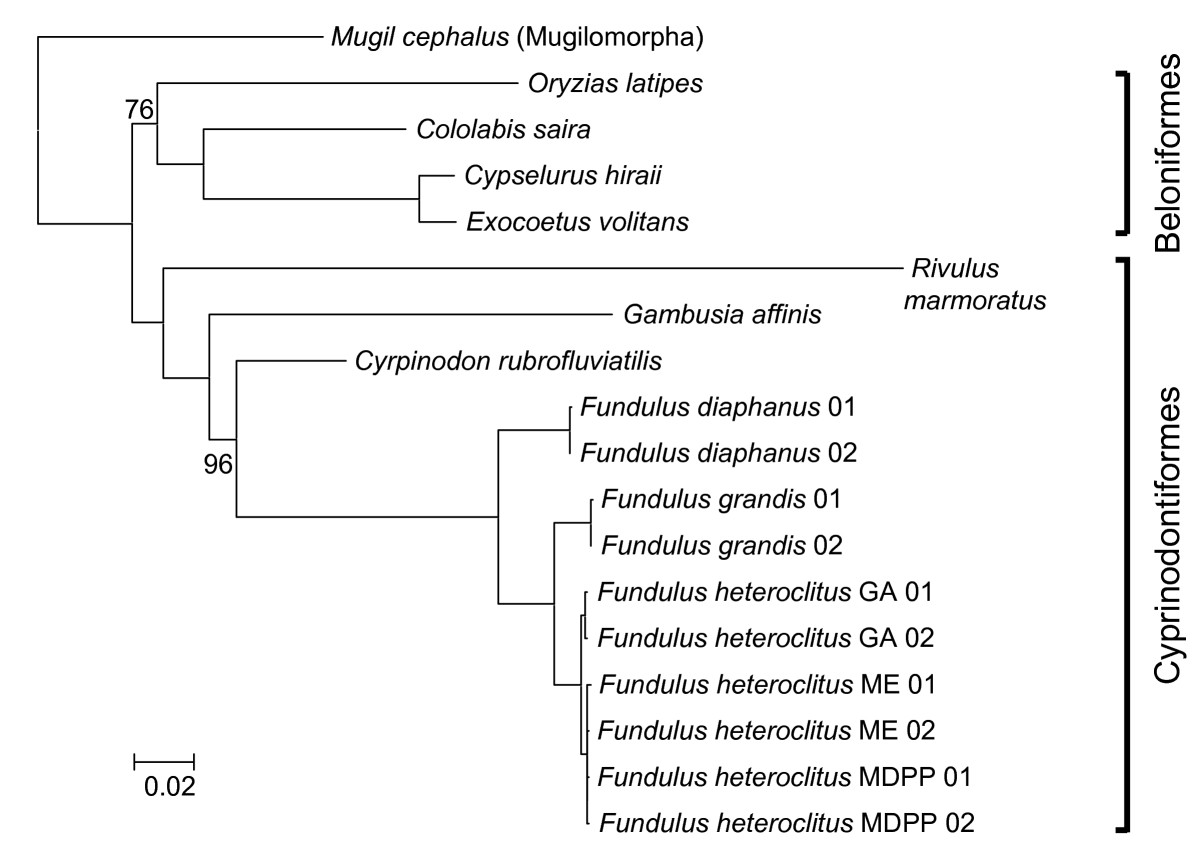

Supplement: Additional file 3 — ZIP files containing several folders, each of which with TreeSnatcher Plus snapshot files, the original image and a text file. [file 1471-2105-13-110-S3.zip › 1471-2148-9-11-1/1471-2148-9-11-1-l.jpg]

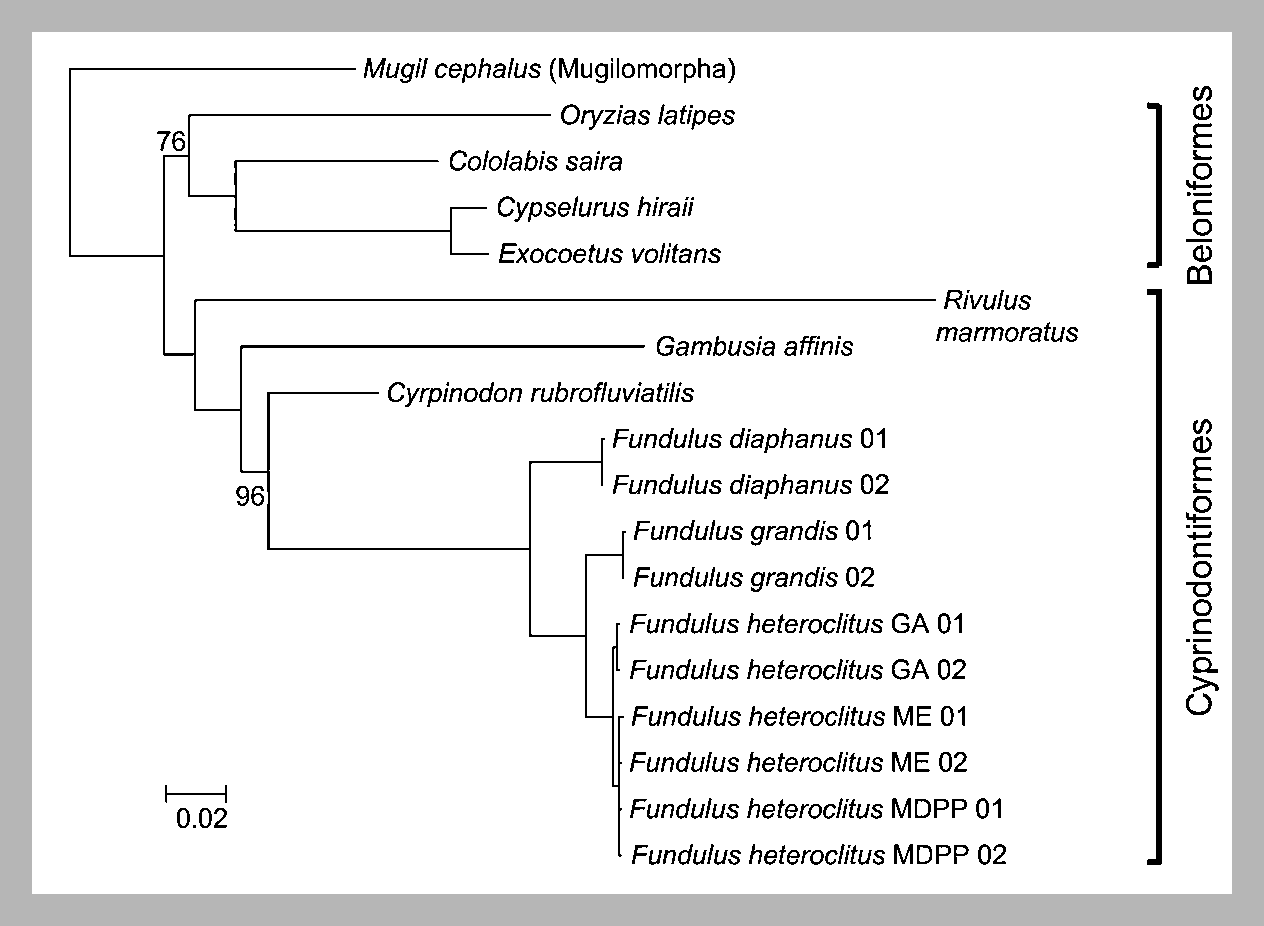

Supplement: Additional file 3 — ZIP files containing several folders, each of which with TreeSnatcher Plus snapshot files, the original image and a text file. [file 1471-2105-13-110-S3.zip › 1471-2148-9-11-1/1471-2148-9-11-1-l_b.PNG]

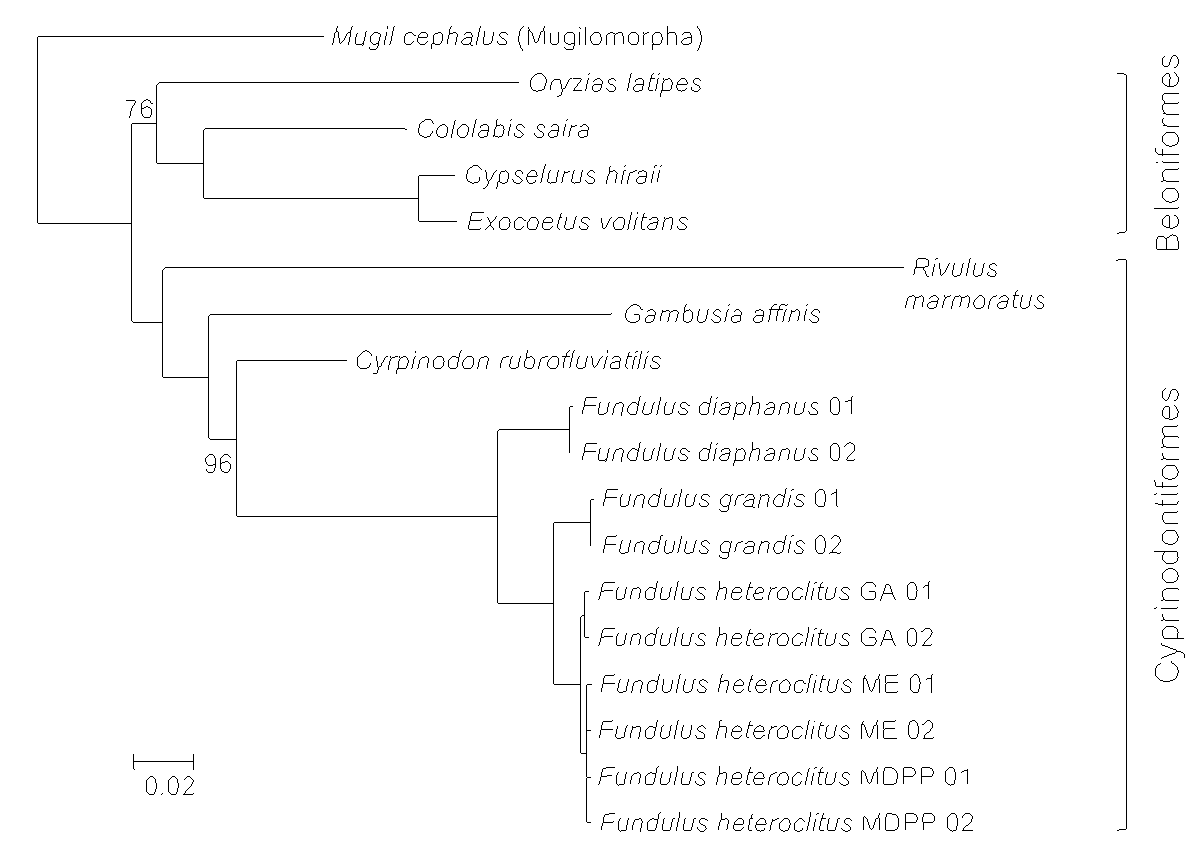

Supplement: Additional file 3 — ZIP files containing several folders, each of which with TreeSnatcher Plus snapshot files, the original image and a text file. [file 1471-2105-13-110-S3.zip › 1471-2148-9-11-1/1471-2148-9-11-1-l_c.PNG]

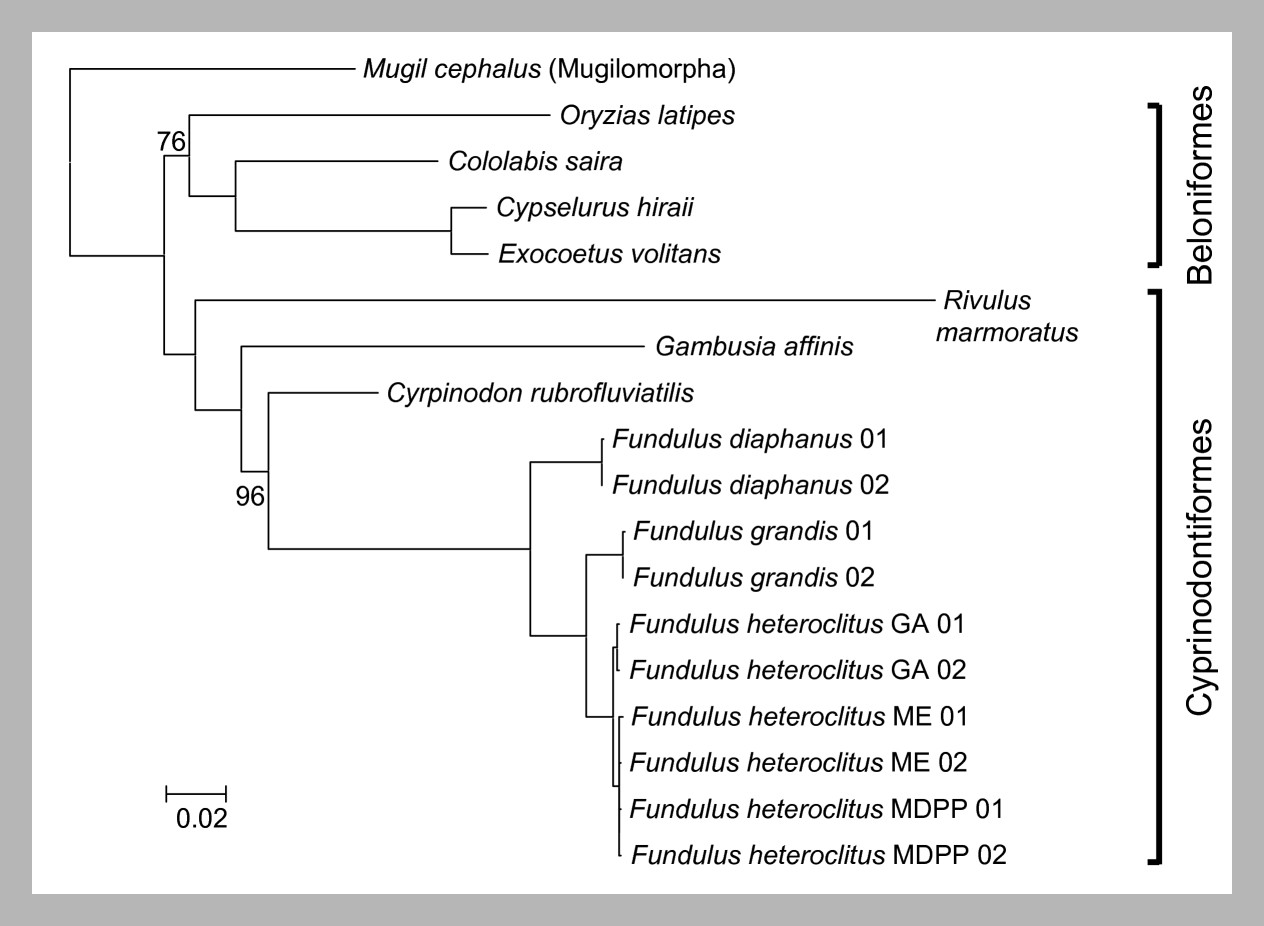

Supplement: Additional file 3 — ZIP files containing several folders, each of which with TreeSnatcher Plus snapshot files, the original image and a text file. [file 1471-2105-13-110-S3.zip › 1471-2148-9-11-1/1471-2148-9-11-1-l_o.PNG]

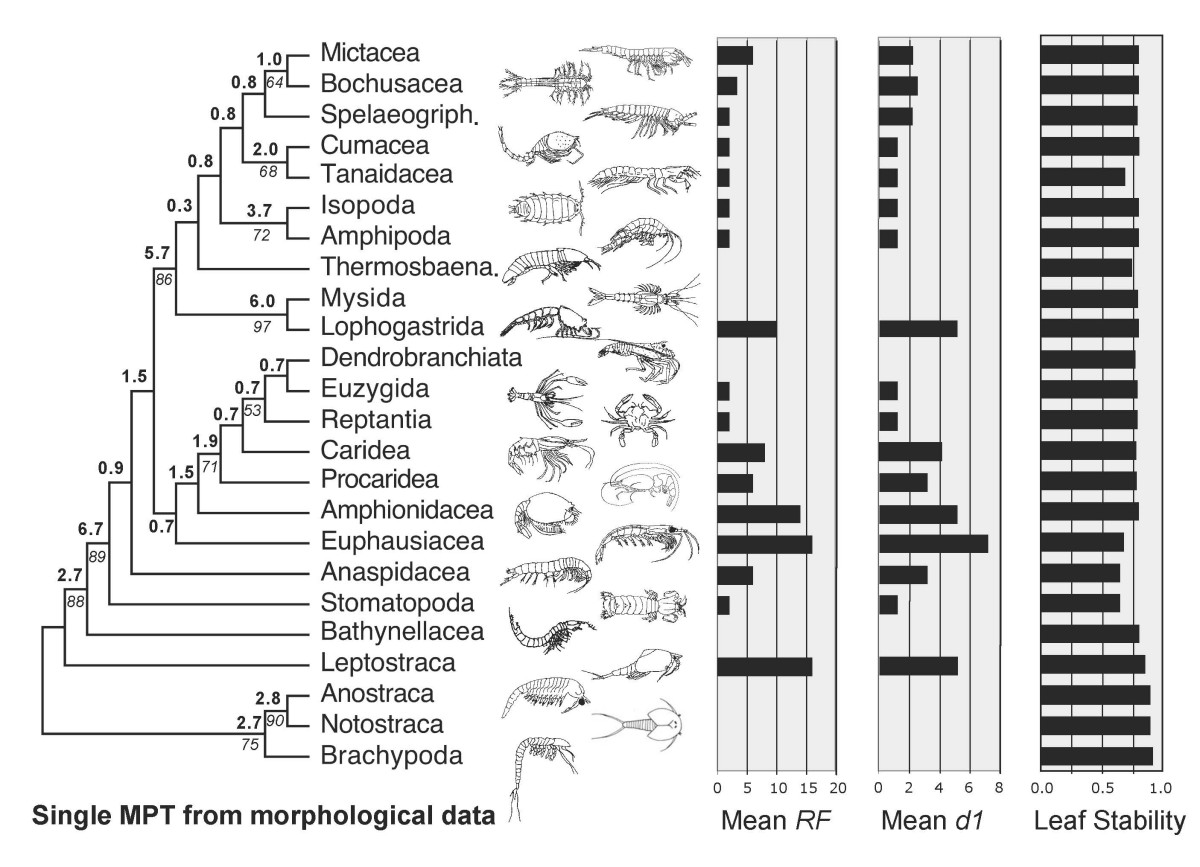

Supplement: Additional file 3 — ZIP files containing several folders, each of which with TreeSnatcher Plus snapshot files, the original image and a text file. [file 1471-2105-13-110-S3.zip › 1471-2148-9-21-1/1471-2148-9-21-1-l.jpg]

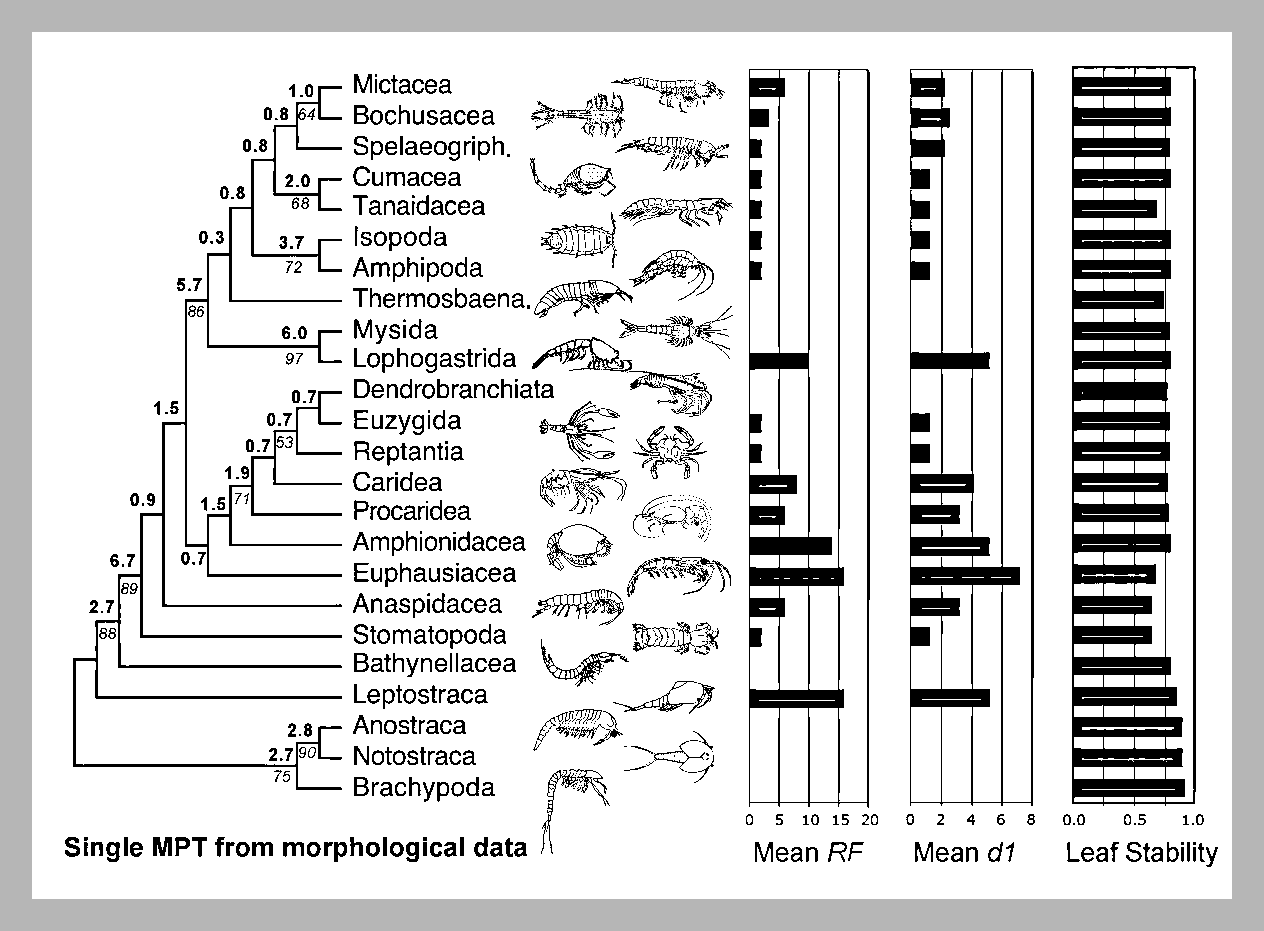

Supplement: Additional file 3 — ZIP files containing several folders, each of which with TreeSnatcher Plus snapshot files, the original image and a text file. [file 1471-2105-13-110-S3.zip › 1471-2148-9-21-1/1471-2148-9-21-1-l_b.PNG]

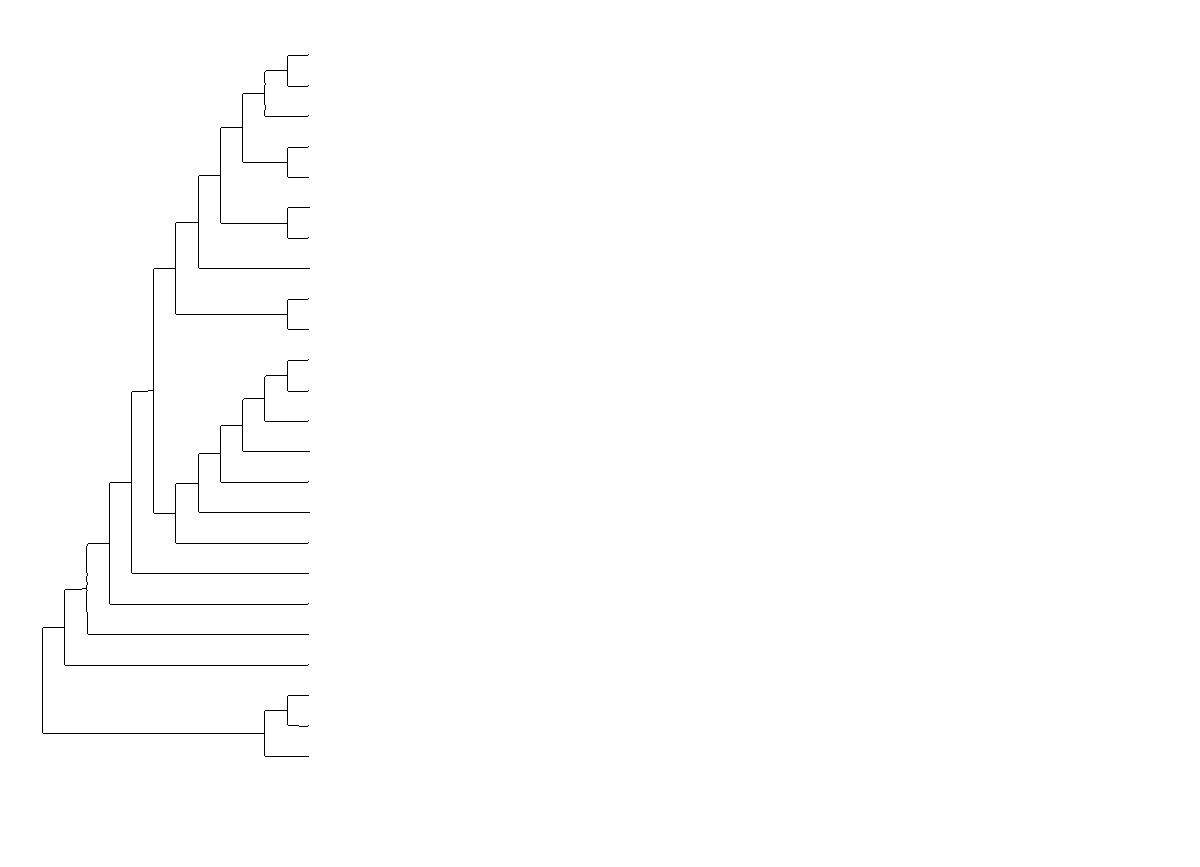

Supplement: Additional file 3 — ZIP files containing several folders, each of which with TreeSnatcher Plus snapshot files, the original image and a text file. [file 1471-2105-13-110-S3.zip › 1471-2148-9-21-1/1471-2148-9-21-1-l_c.PNG]

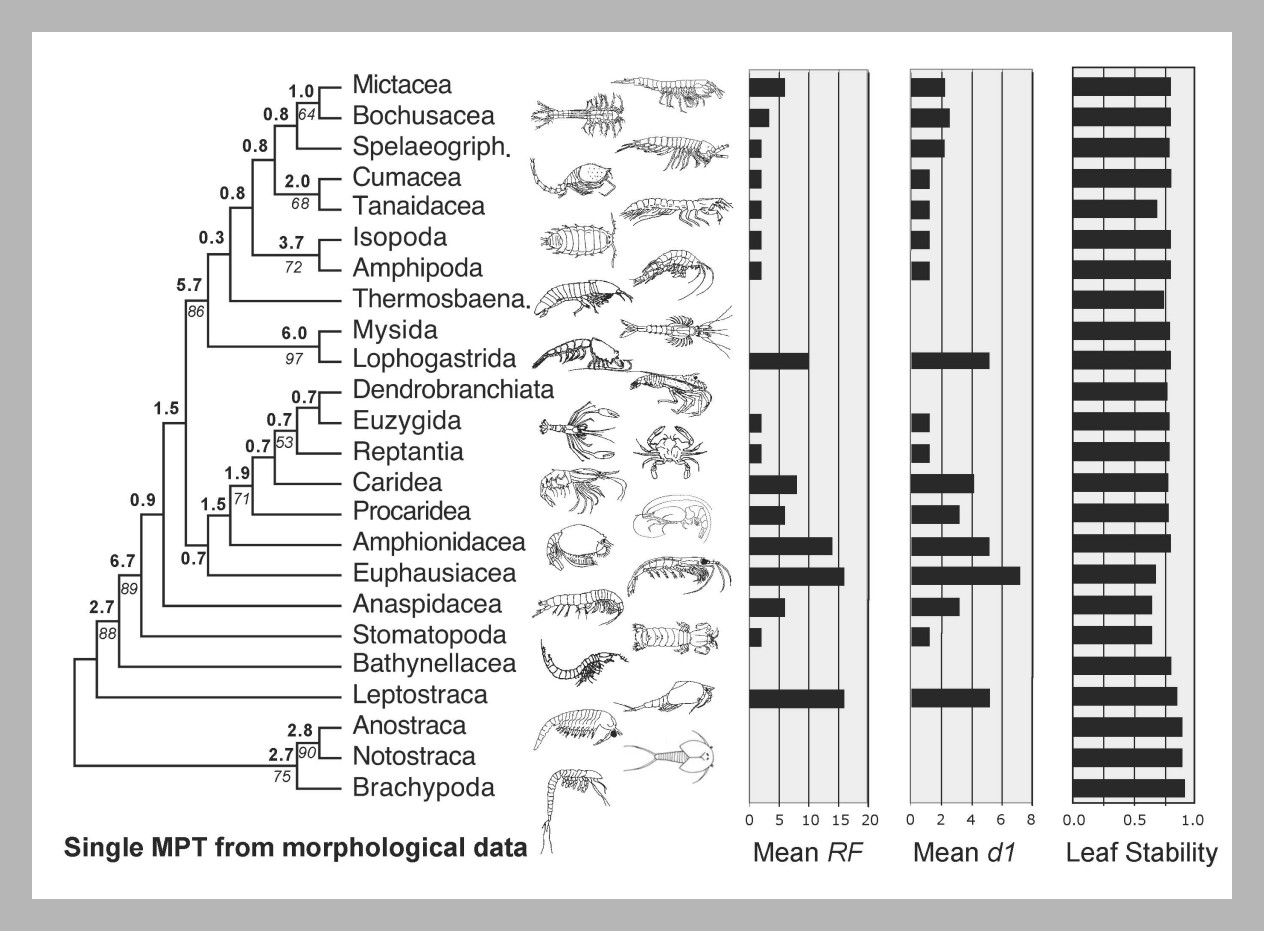

Supplement: Additional file 3 — ZIP files containing several folders, each of which with TreeSnatcher Plus snapshot files, the original image and a text file. [file 1471-2105-13-110-S3.zip › 1471-2148-9-21-1/1471-2148-9-21-1-l_o.PNG]

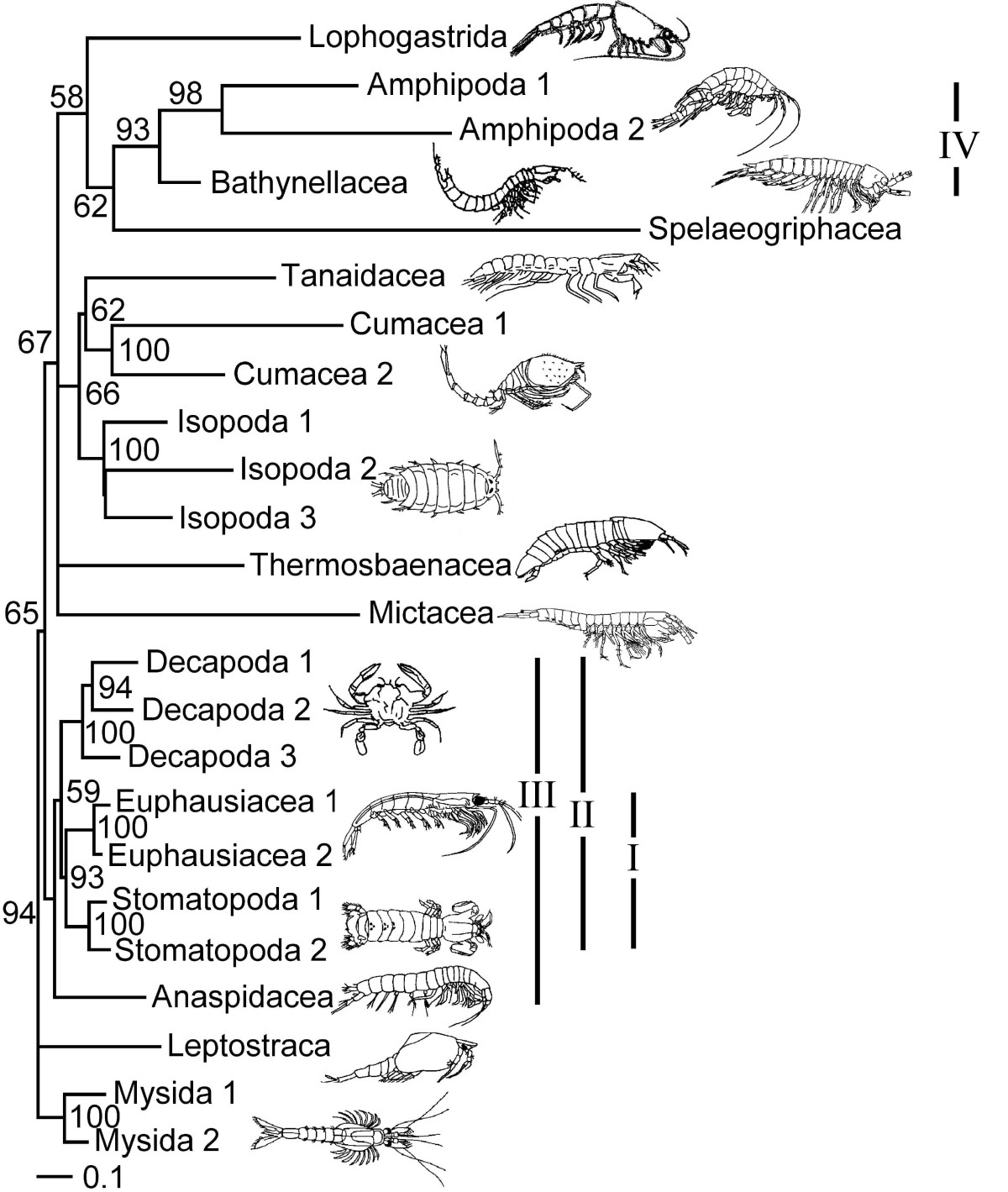

Supplement: Additional file 3 — ZIP files containing several folders, each of which with TreeSnatcher Plus snapshot files, the original image and a text file. [file 1471-2105-13-110-S3.zip › 1471-2148-9-21-8/1471-2148-9-21-8-l.jpg]

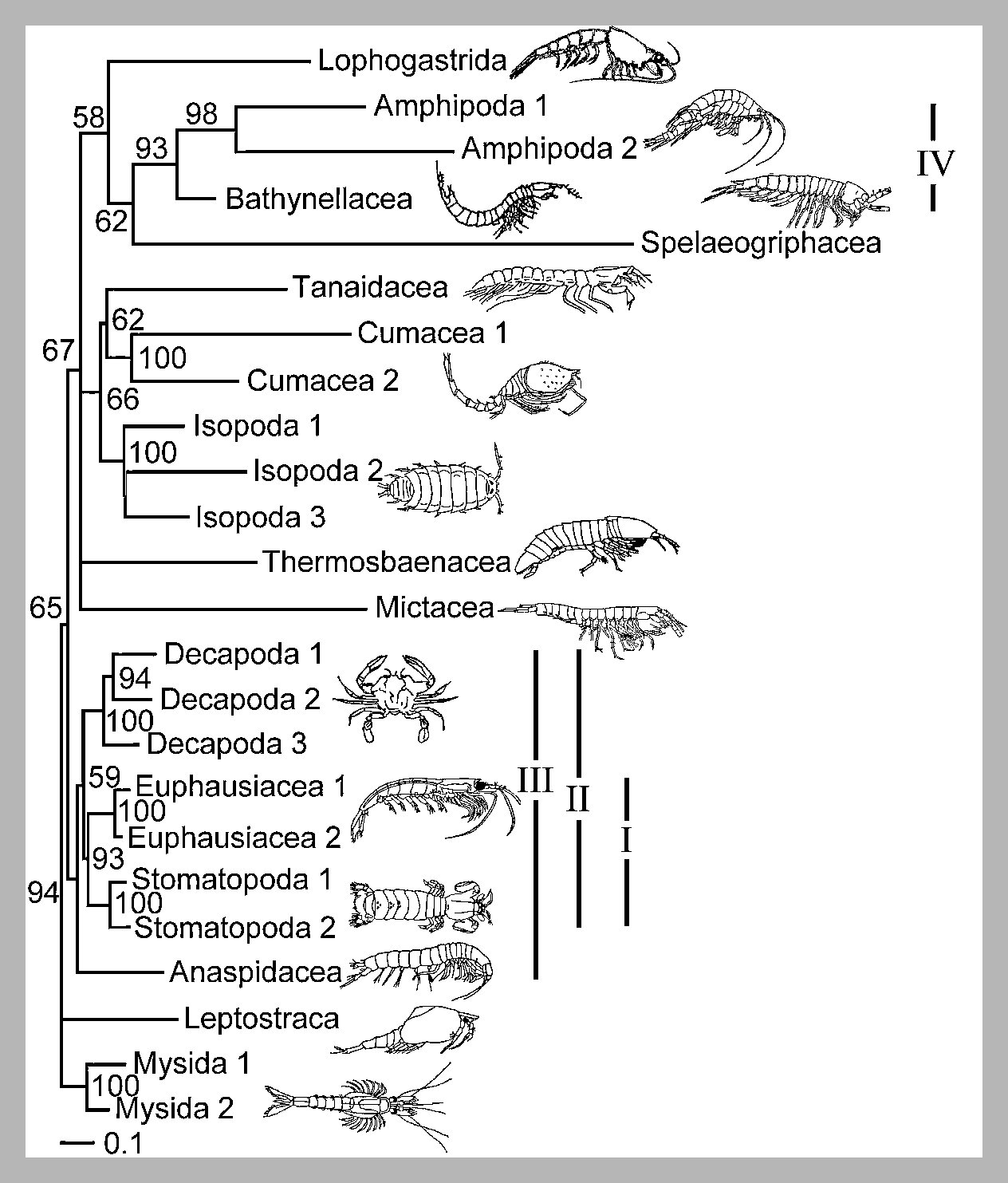

Supplement: Additional file 3 — ZIP files containing several folders, each of which with TreeSnatcher Plus snapshot files, the original image and a text file. [file 1471-2105-13-110-S3.zip › 1471-2148-9-21-8/1471-2148-9-21-8-l_b.PNG]

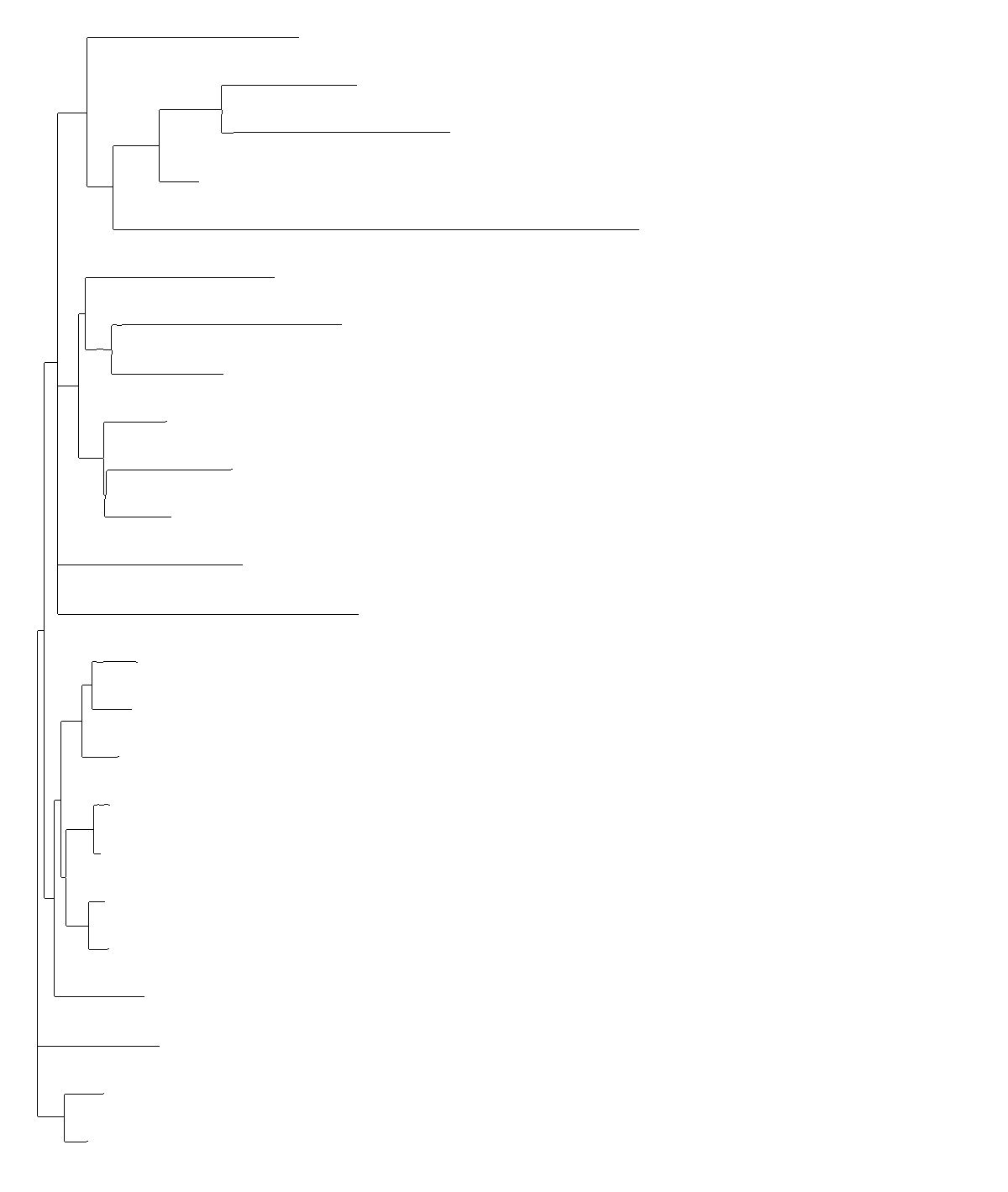

Supplement: Additional file 3 — ZIP files containing several folders, each of which with TreeSnatcher Plus snapshot files, the original image and a text file. [file 1471-2105-13-110-S3.zip › 1471-2148-9-21-8/1471-2148-9-21-8-l_c.PNG]

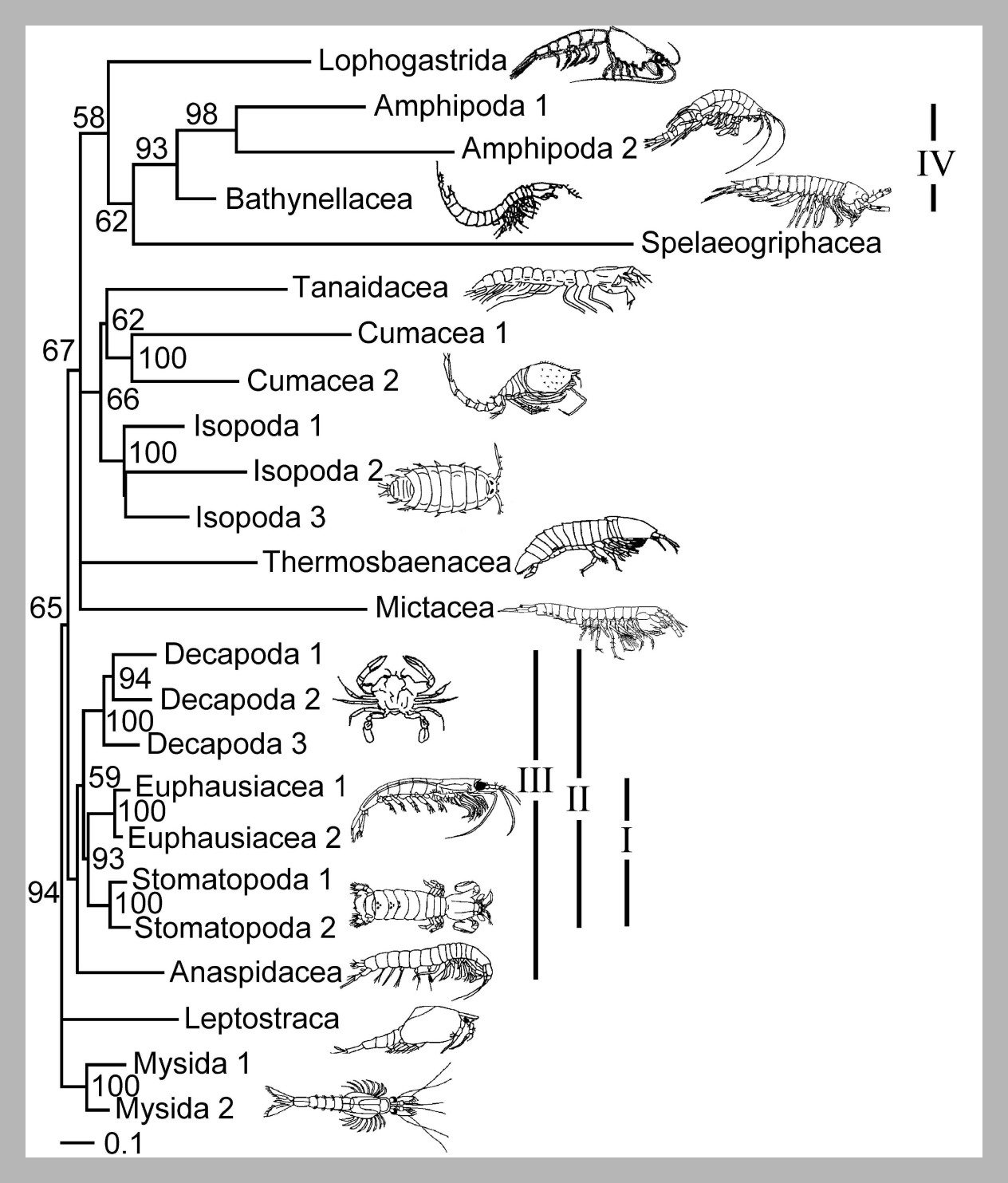

Supplement: Additional file 3 — ZIP files containing several folders, each of which with TreeSnatcher Plus snapshot files, the original image and a text file. [file 1471-2105-13-110-S3.zip › 1471-2148-9-21-8/1471-2148-9-21-8-l_o.PNG]

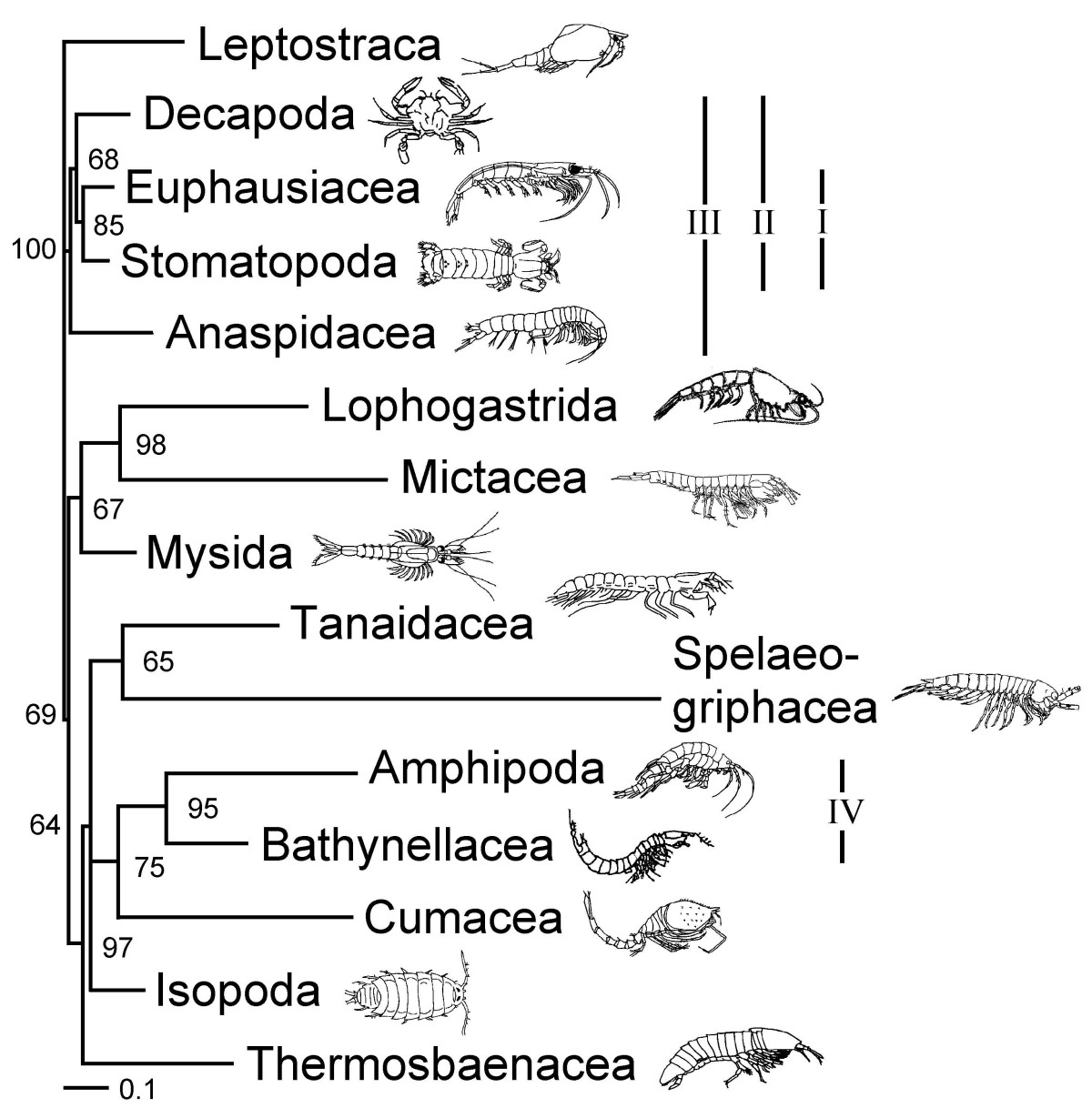

Supplement: Additional file 3 — ZIP files containing several folders, each of which with TreeSnatcher Plus snapshot files, the original image and a text file. [file 1471-2105-13-110-S3.zip › 1471-2148-9-21-9/1471-2148-9-21-9-l.jpg]

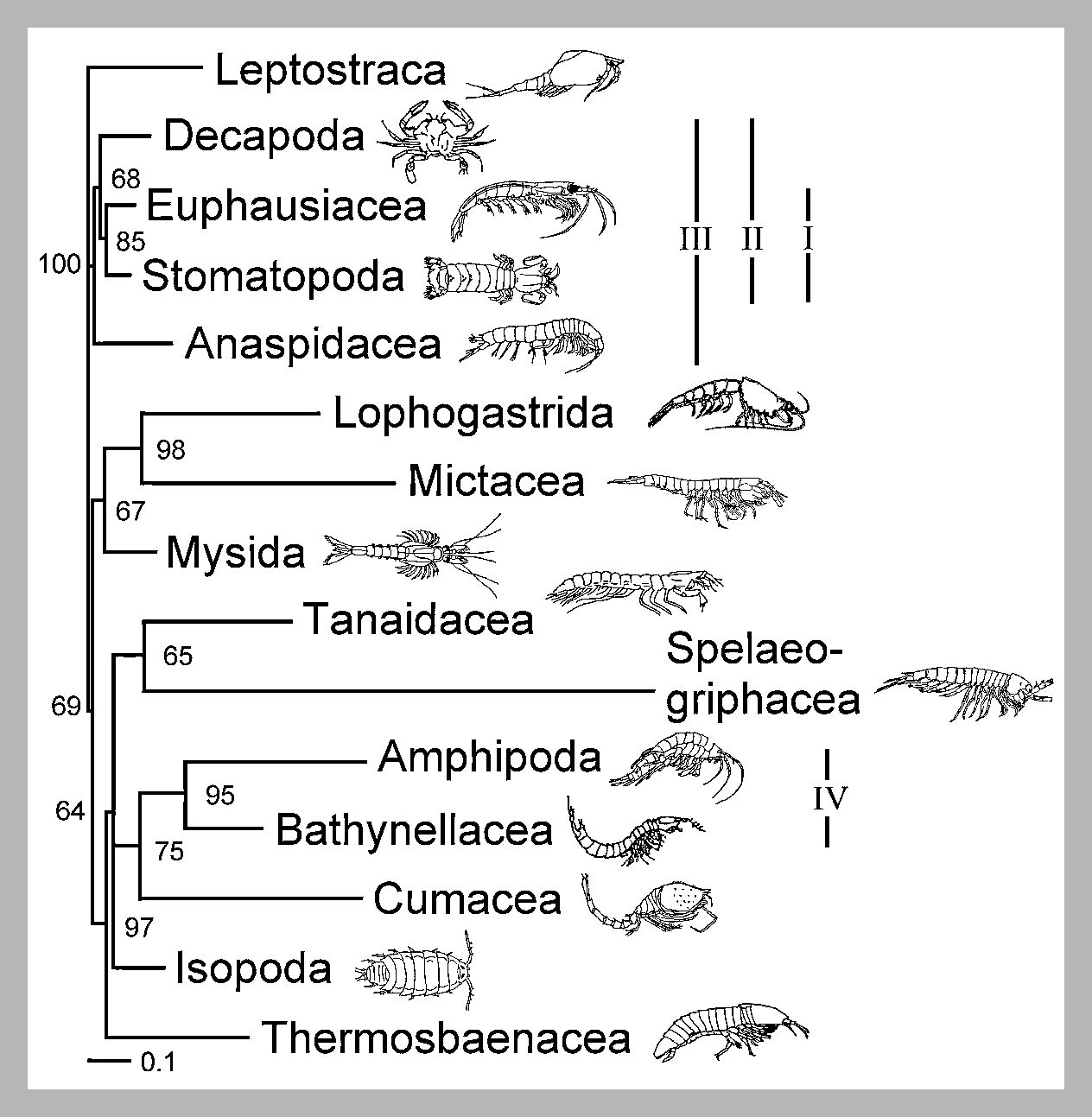

Supplement: Additional file 3 — ZIP files containing several folders, each of which with TreeSnatcher Plus snapshot files, the original image and a text file. [file 1471-2105-13-110-S3.zip › 1471-2148-9-21-9/1471-2148-9-21-9-l_b.PNG]

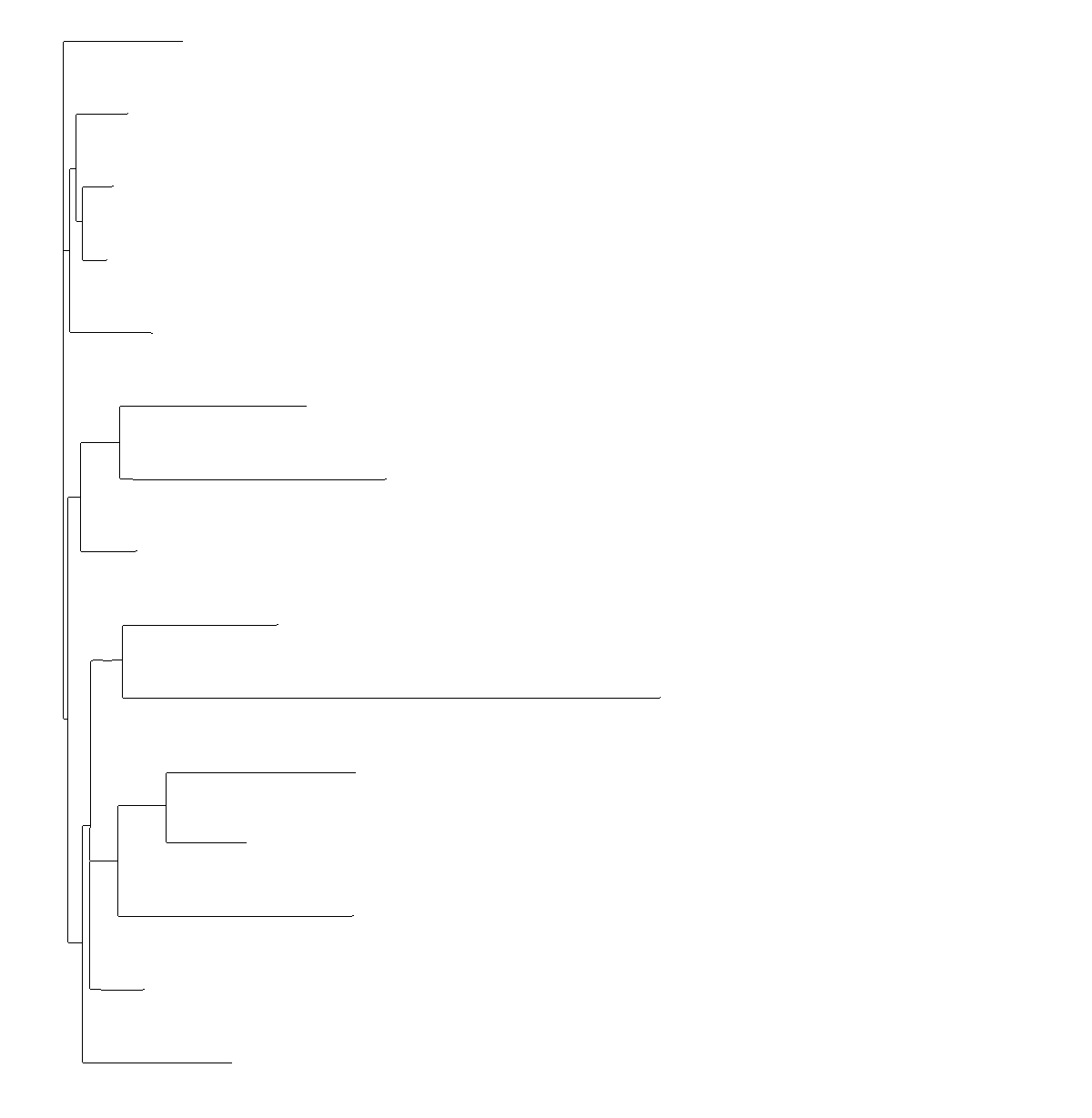

Supplement: Additional file 3 — ZIP files containing several folders, each of which with TreeSnatcher Plus snapshot files, the original image and a text file. [file 1471-2105-13-110-S3.zip › 1471-2148-9-21-9/1471-2148-9-21-9-l_c.PNG]

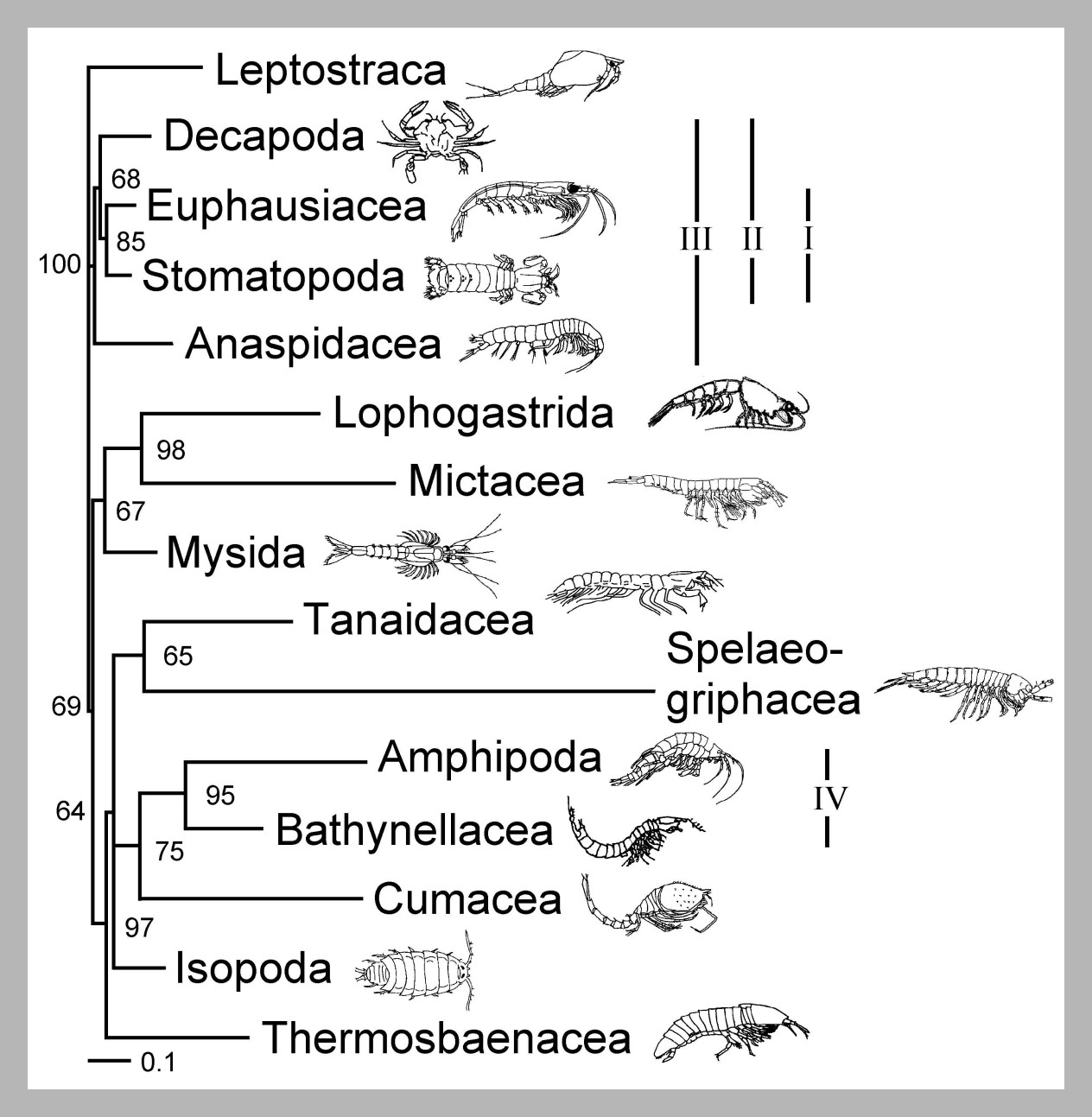

Supplement: Additional file 3 — ZIP files containing several folders, each of which with TreeSnatcher Plus snapshot files, the original image and a text file. [file 1471-2105-13-110-S3.zip › 1471-2148-9-21-9/1471-2148-9-21-9-l_o.PNG]

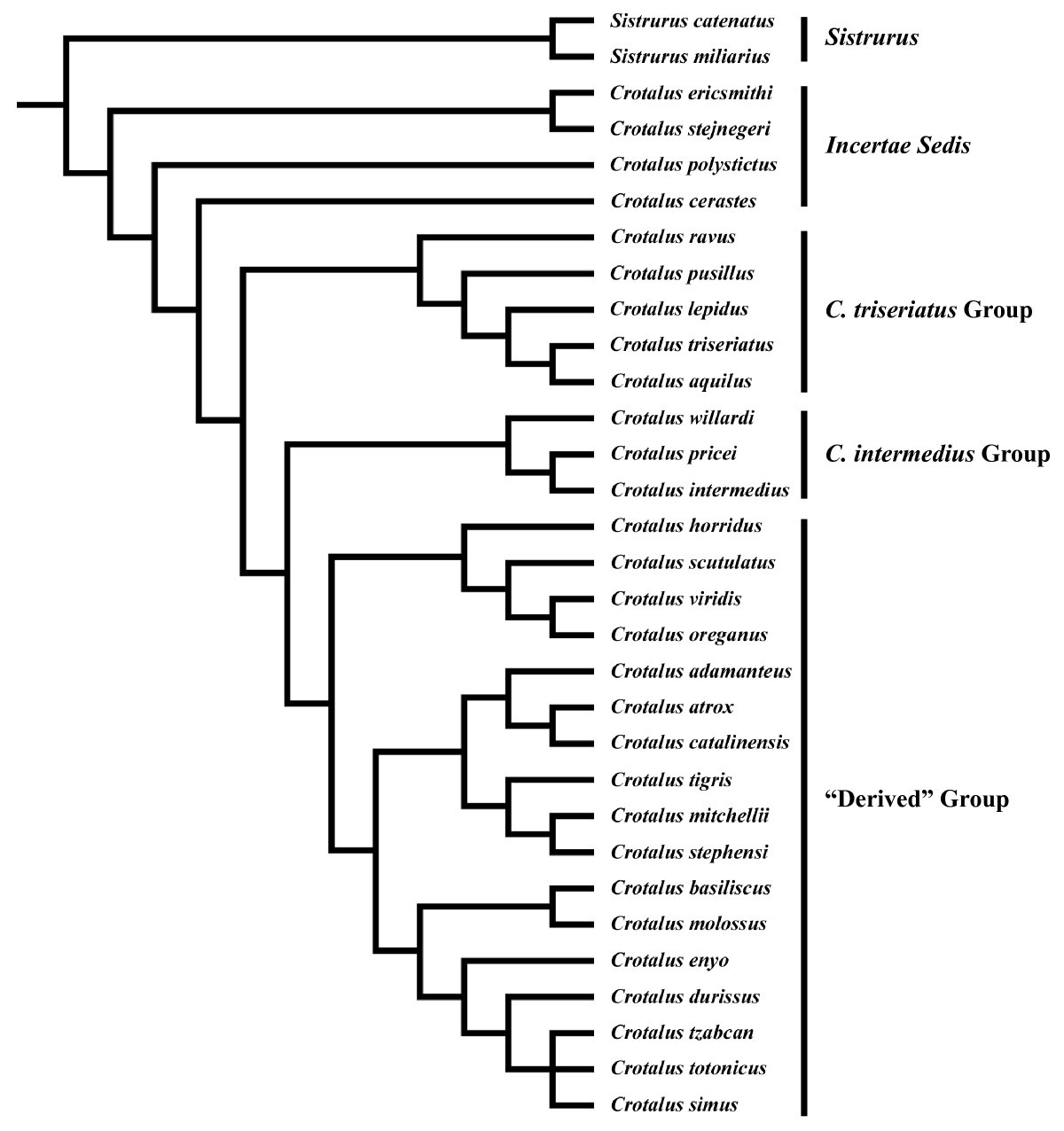

Supplement: Additional file 3 — ZIP files containing several folders, each of which with TreeSnatcher Plus snapshot files, the original image and a text file. [file 1471-2105-13-110-S3.zip › 1471-2148-9-35-2/1471-2148-9-35-2-l.jpg]

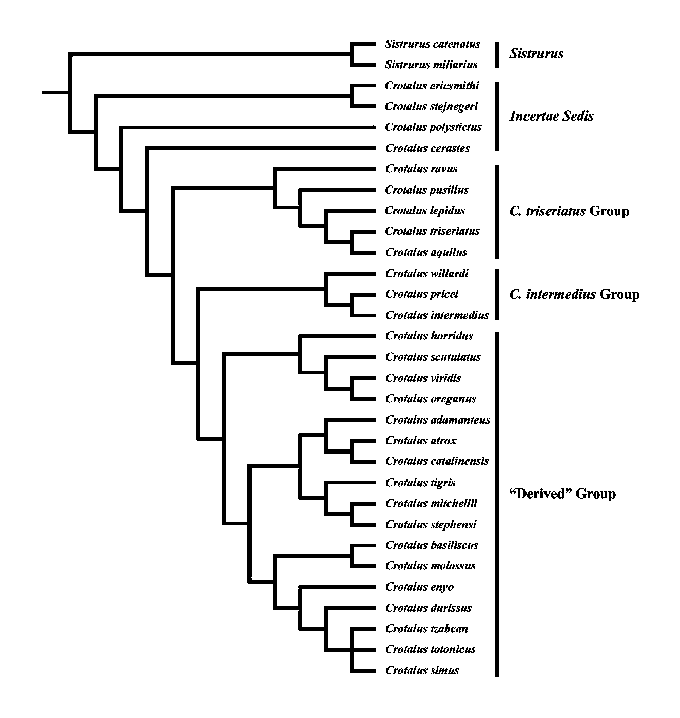

Supplement: Additional file 3 — ZIP files containing several folders, each of which with TreeSnatcher Plus snapshot files, the original image and a text file. [file 1471-2105-13-110-S3.zip › 1471-2148-9-35-2/1471-2148-9-35-2-l_b.PNG]

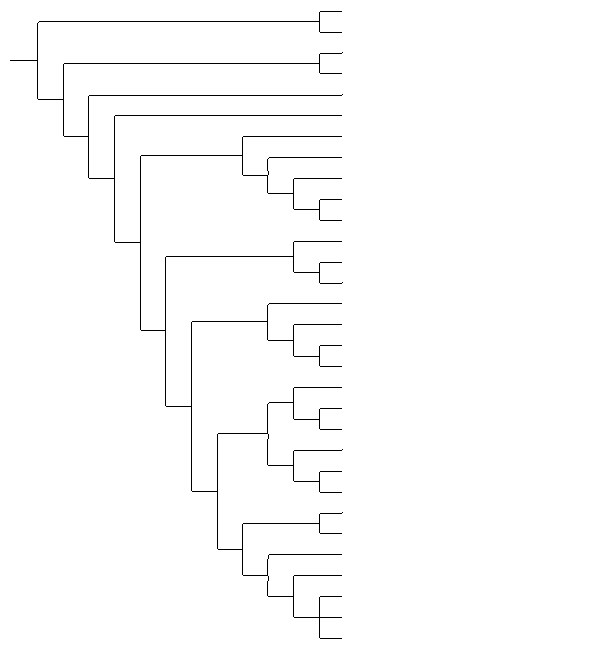

Supplement: Additional file 3 — ZIP files containing several folders, each of which with TreeSnatcher Plus snapshot files, the original image and a text file. [file 1471-2105-13-110-S3.zip › 1471-2148-9-35-2/1471-2148-9-35-2-l_c.PNG]

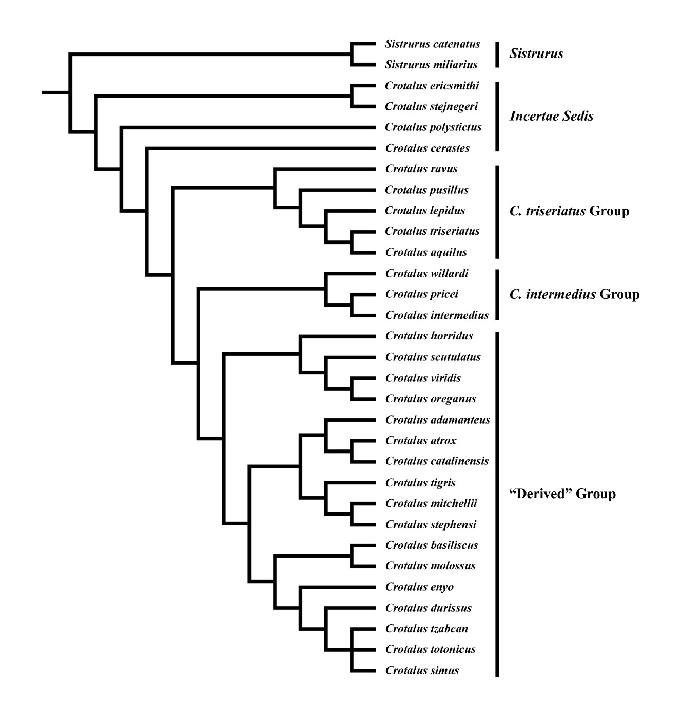

Supplement: Additional file 3 — ZIP files containing several folders, each of which with TreeSnatcher Plus snapshot files, the original image and a text file. [file 1471-2105-13-110-S3.zip › 1471-2148-9-35-2/1471-2148-9-35-2-l_o.PNG]

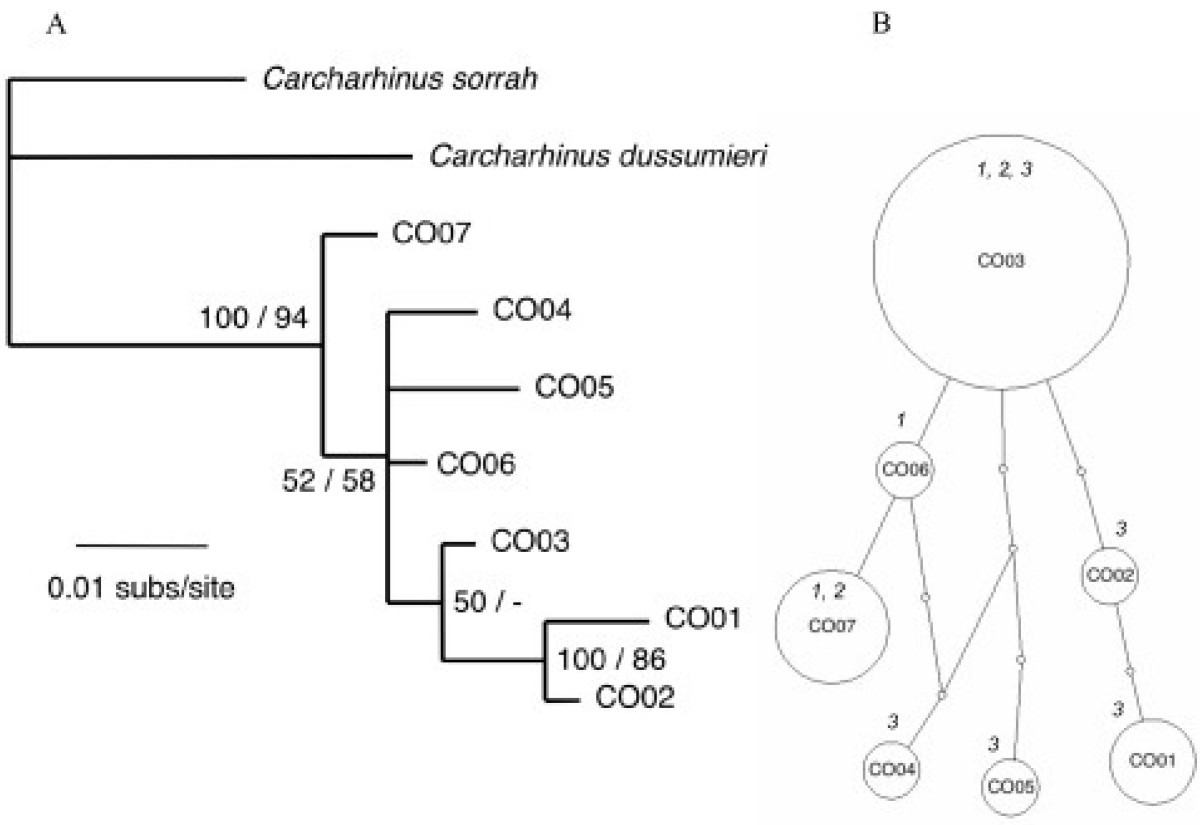

Supplement: Additional file 3 — ZIP files containing several folders, each of which with TreeSnatcher Plus snapshot files, the original image and a text file. [file 1471-2105-13-110-S3.zip › 1471-2148-9-40-2/1471-2148-9-40-2-l.jpg]

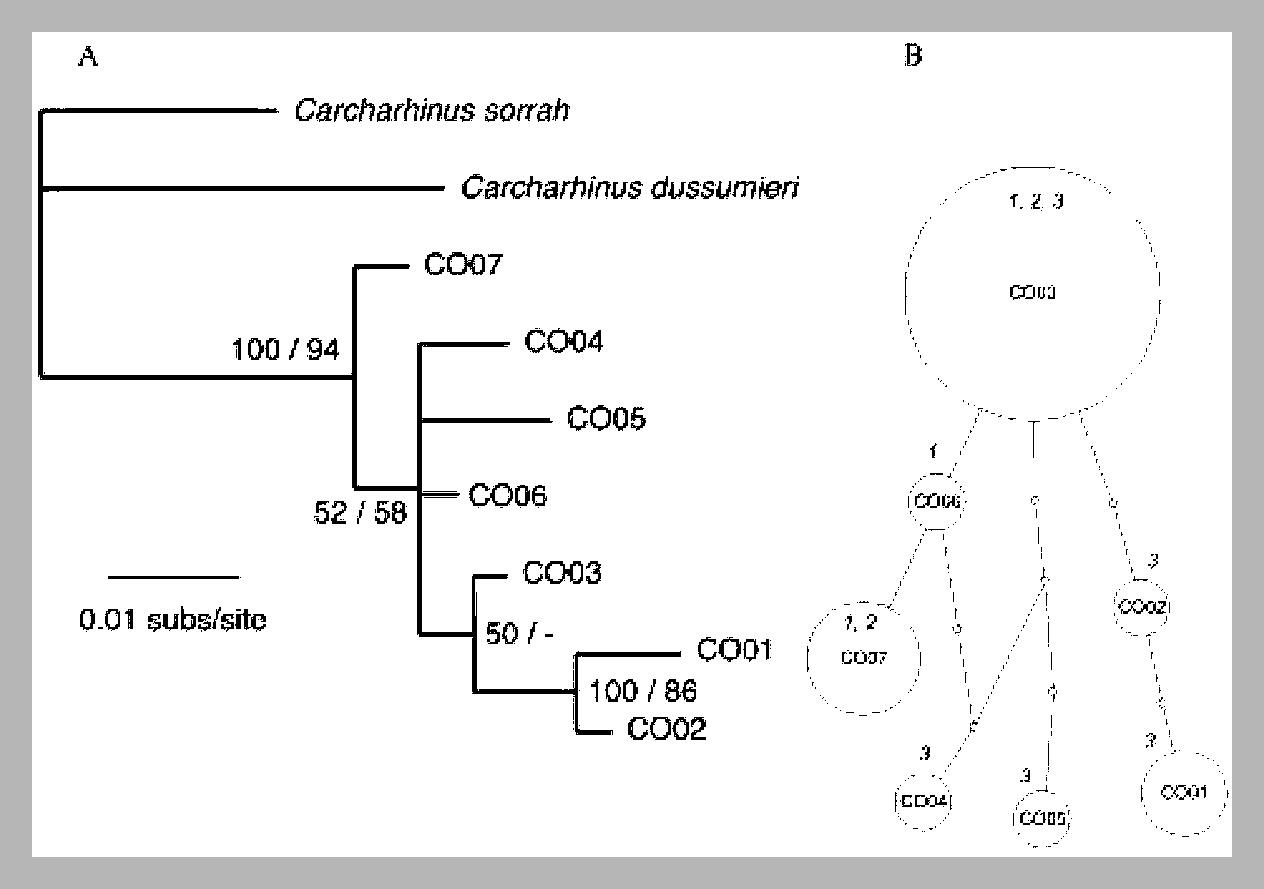

Supplement: Additional file 3 — ZIP files containing several folders, each of which with TreeSnatcher Plus snapshot files, the original image and a text file. [file 1471-2105-13-110-S3.zip › 1471-2148-9-40-2/1471-2148-9-40-2-l_b.PNG]

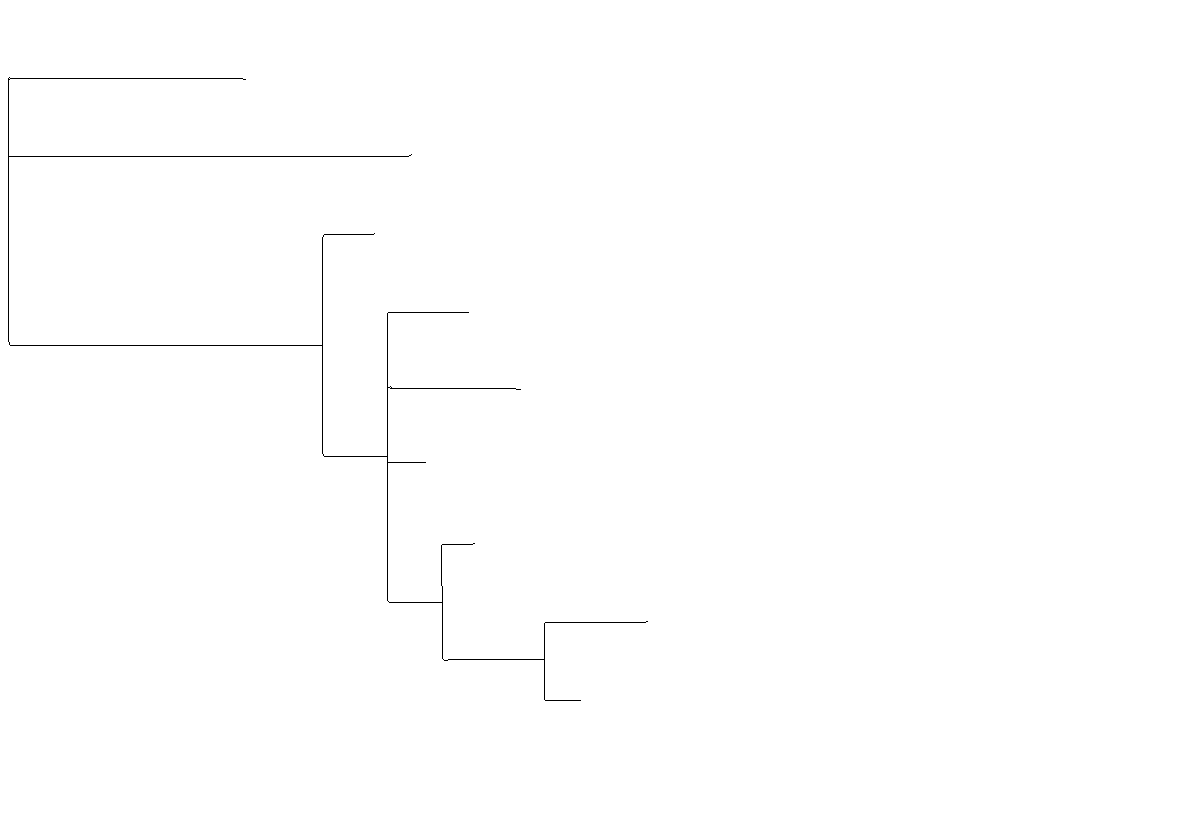

Supplement: Additional file 3 — ZIP files containing several folders, each of which with TreeSnatcher Plus snapshot files, the original image and a text file. [file 1471-2105-13-110-S3.zip › 1471-2148-9-40-2/1471-2148-9-40-2-l_c.PNG]

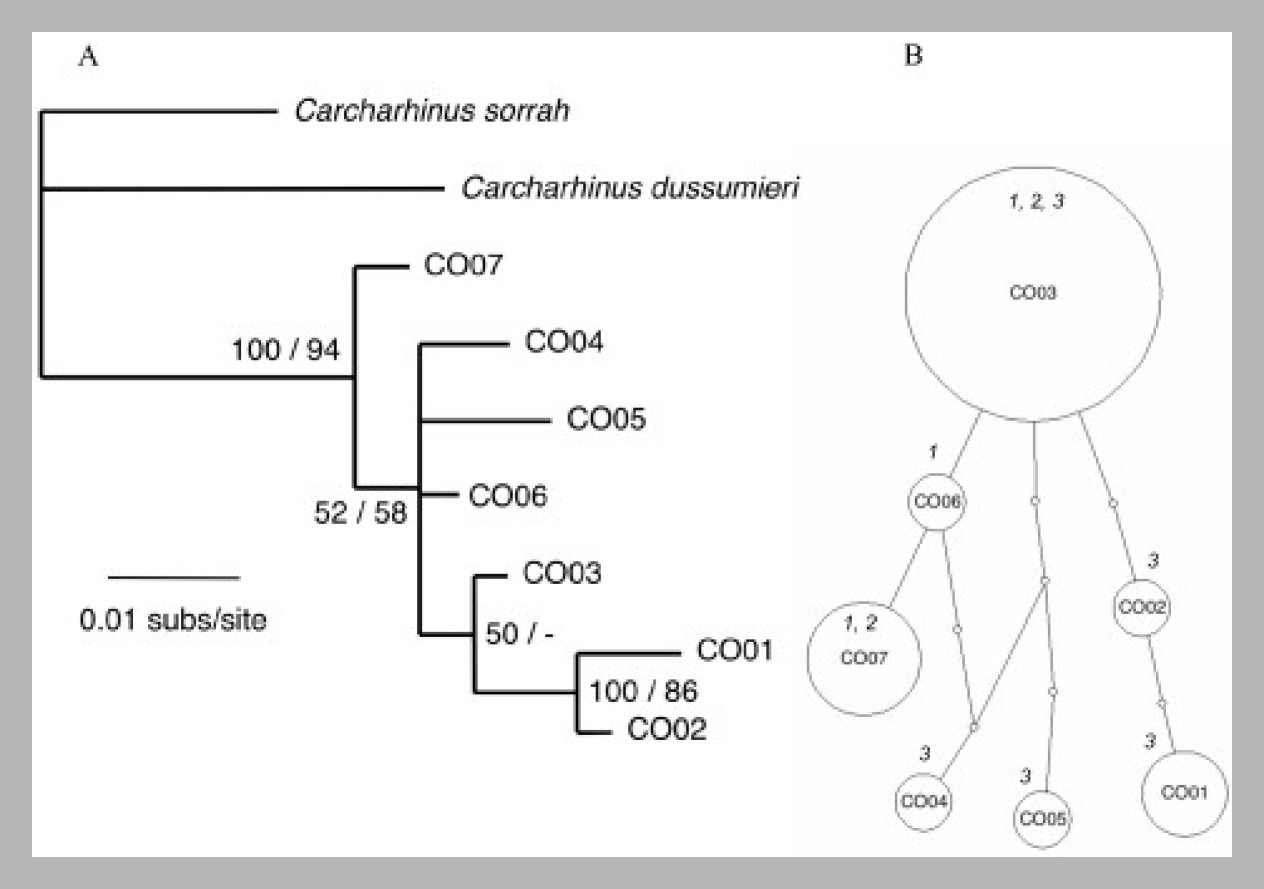

Supplement: Additional file 3 — ZIP files containing several folders, each of which with TreeSnatcher Plus snapshot files, the original image and a text file. [file 1471-2105-13-110-S3.zip › 1471-2148-9-40-2/1471-2148-9-40-2-l_o.PNG]

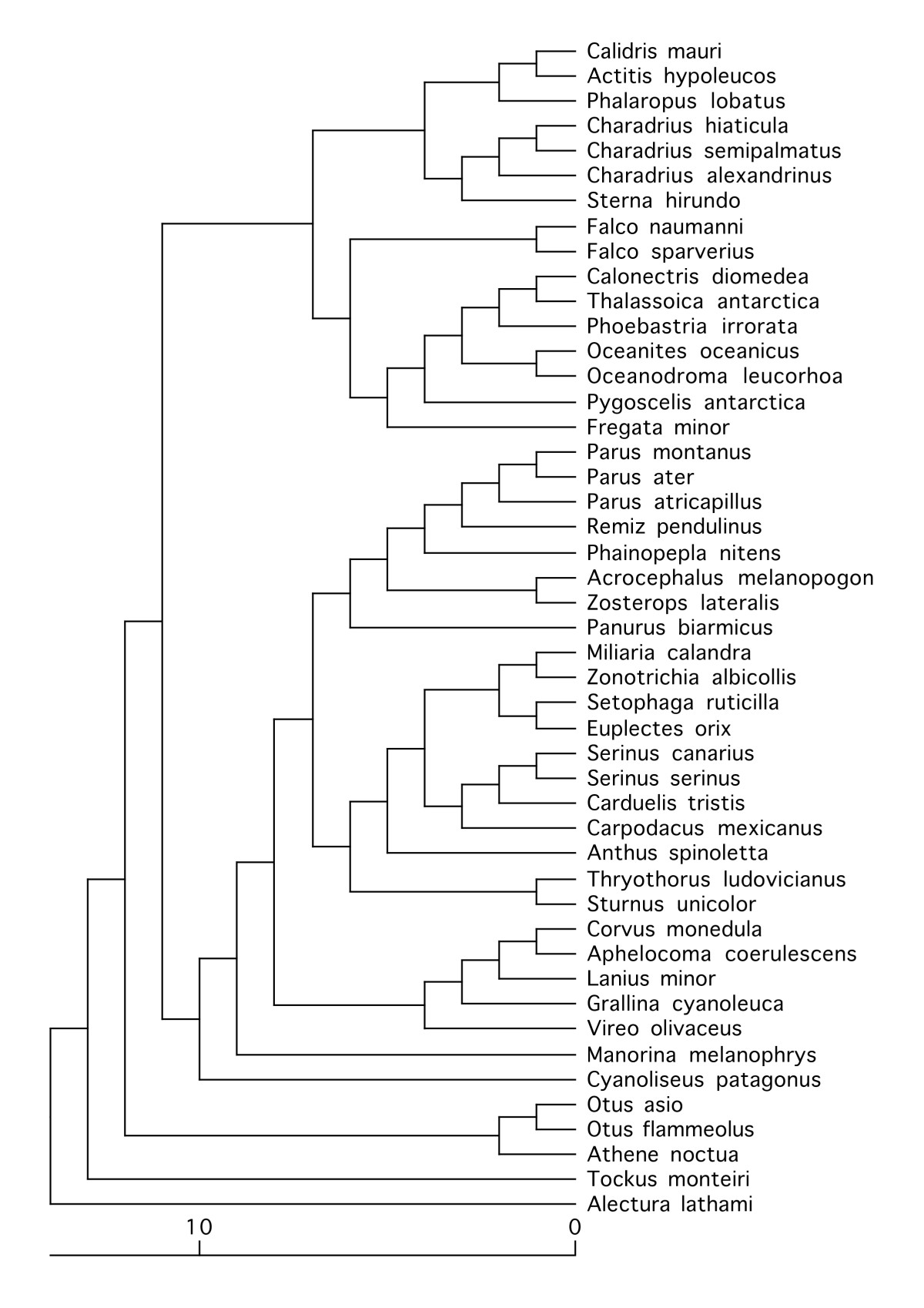

Supplement: Additional file 3 — ZIP files containing several folders, each of which with TreeSnatcher Plus snapshot files, the original image and a text file. [file 1471-2105-13-110-S3.zip › 1471-2148-9-5-3/1471-2148-9-5-3-l.jpg]

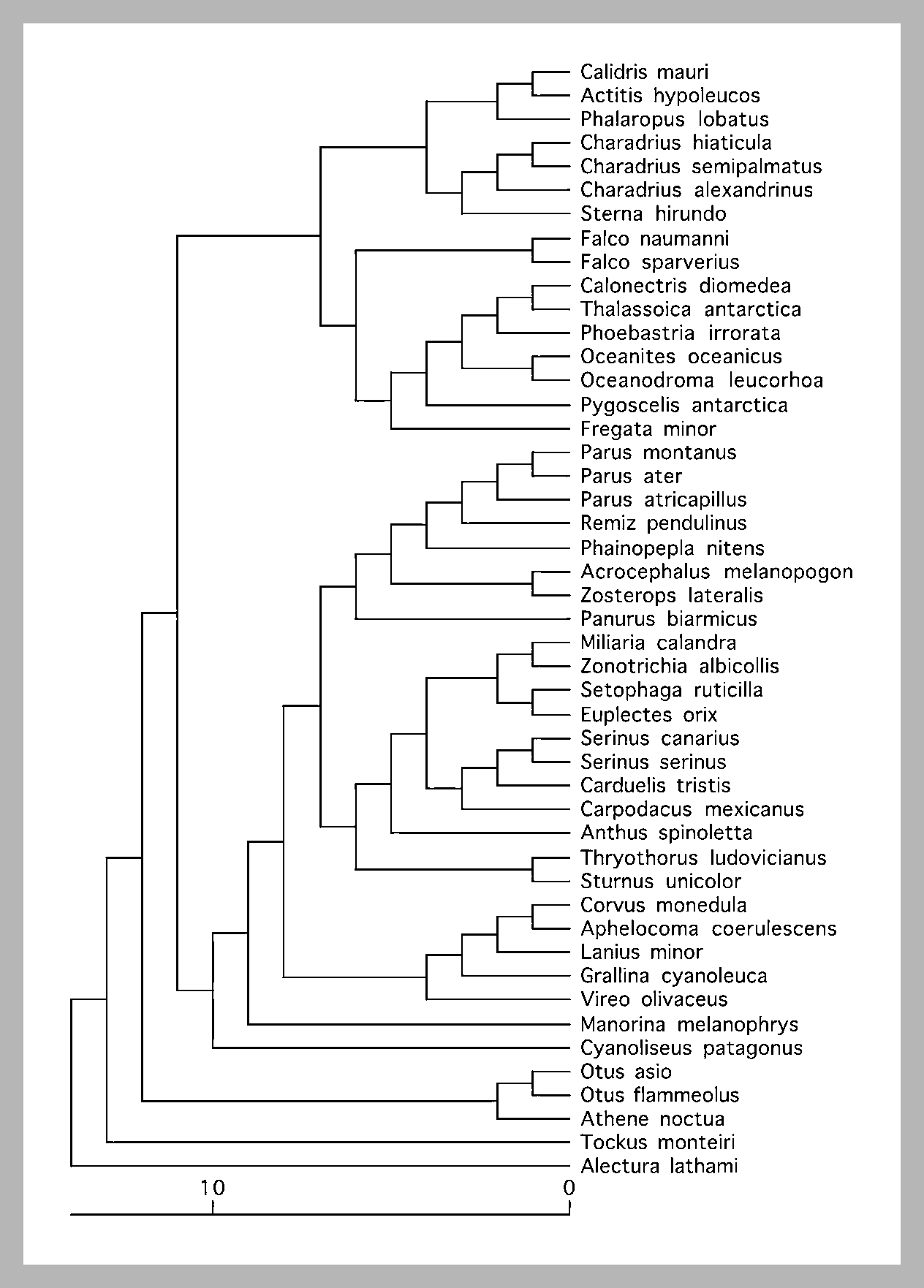

Supplement: Additional file 3 — ZIP files containing several folders, each of which with TreeSnatcher Plus snapshot files, the original image and a text file. [file 1471-2105-13-110-S3.zip › 1471-2148-9-5-3/1471-2148-9-5-3-l_b.PNG]

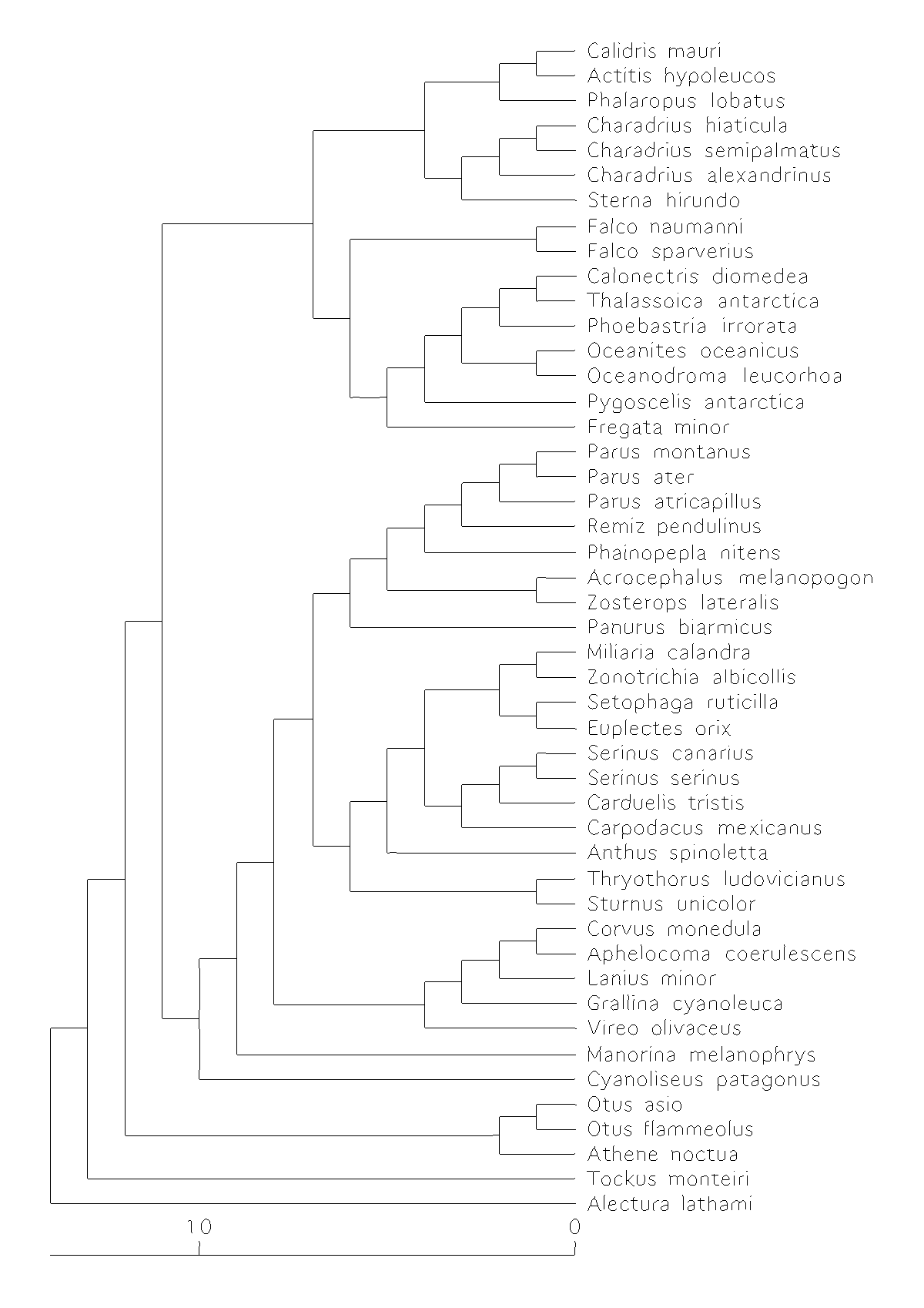

Supplement: Additional file 3 — ZIP files containing several folders, each of which with TreeSnatcher Plus snapshot files, the original image and a text file. [file 1471-2105-13-110-S3.zip › 1471-2148-9-5-3/1471-2148-9-5-3-l_c.PNG]

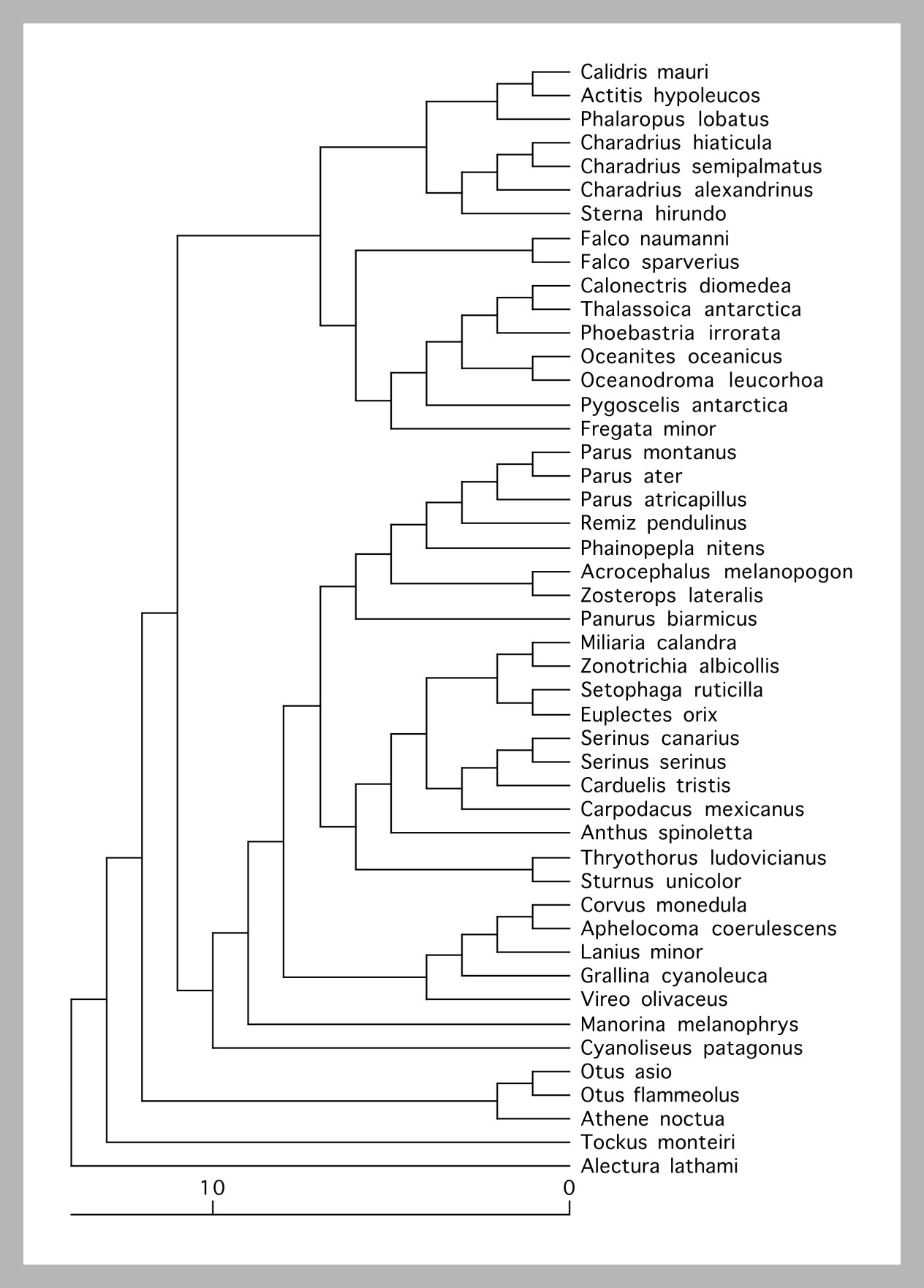

Supplement: Additional file 3 — ZIP files containing several folders, each of which with TreeSnatcher Plus snapshot files, the original image and a text file. [file 1471-2105-13-110-S3.zip › 1471-2148-9-5-3/1471-2148-9-5-3-l_o.PNG]

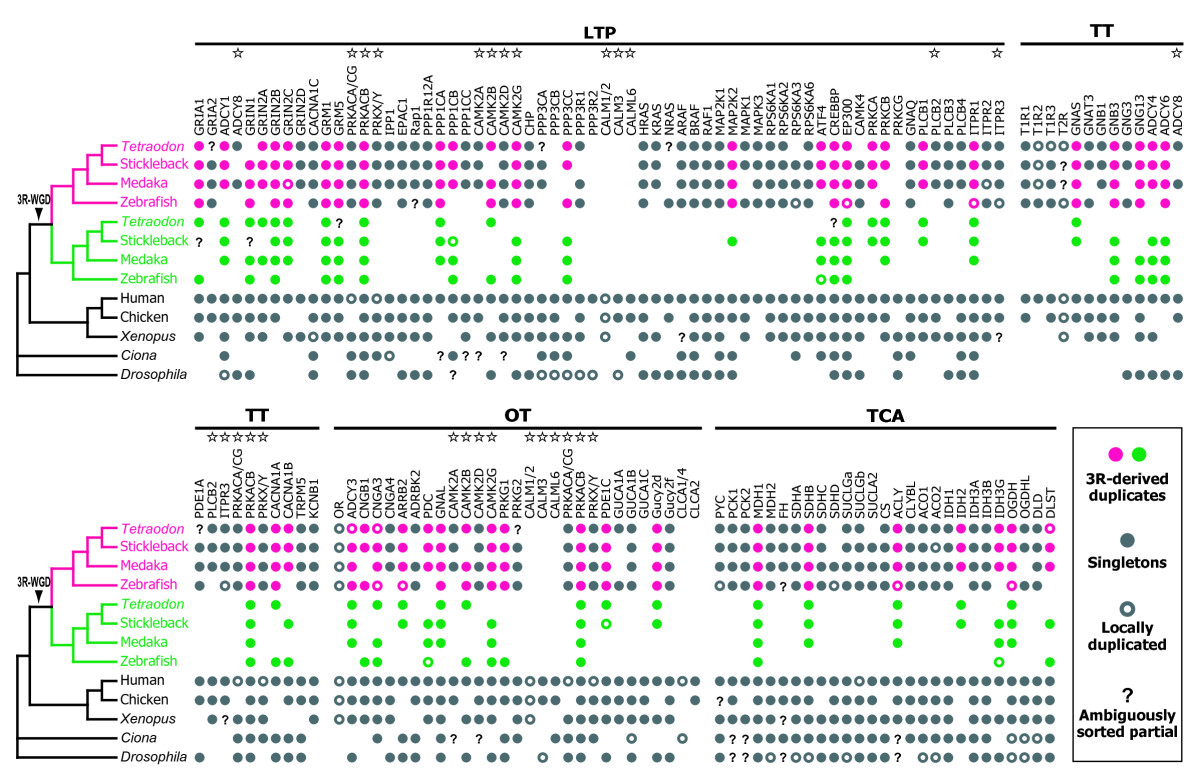

Supplement: Additional file 4 — ZIP files containing several folders, each of which with TreeSnatcher Plus snapshot files, the original image and a text file. [file 1471-2105-13-110-S4.zip › 1471-2148-9-127-2/1471-2148-9-127-2-l.jpg]

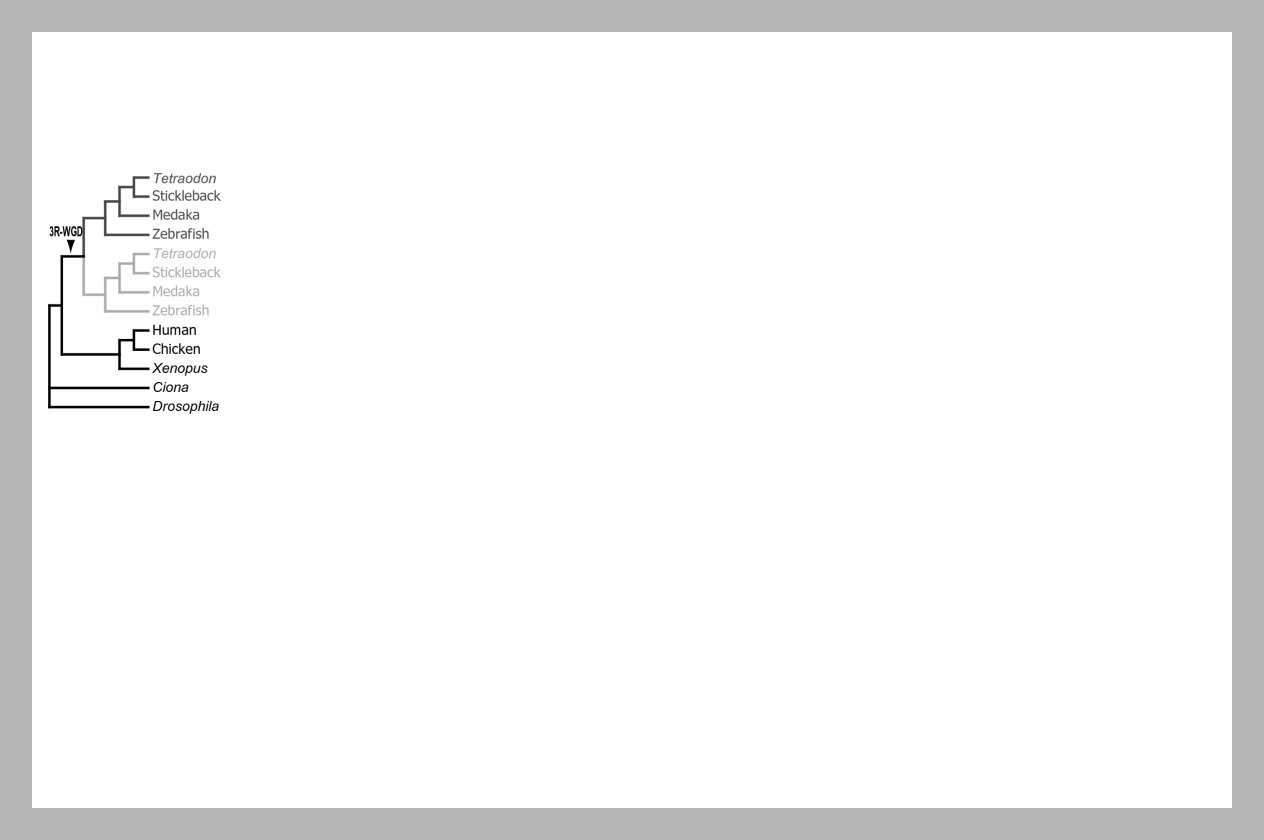

Supplement: Additional file 4 — ZIP files containing several folders, each of which with TreeSnatcher Plus snapshot files, the original image and a text file. [file 1471-2105-13-110-S4.zip › 1471-2148-9-127-2/1471-2148-9-127-2-l_b.PNG]

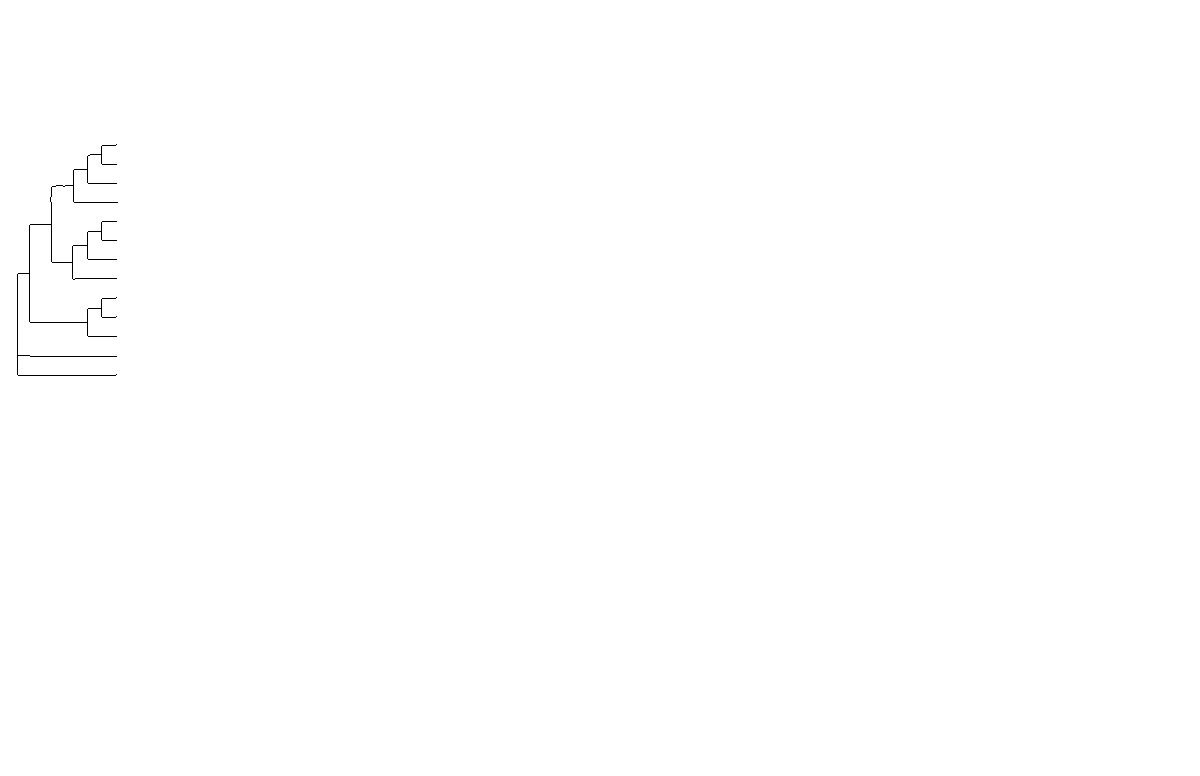

Supplement: Additional file 4 — ZIP files containing several folders, each of which with TreeSnatcher Plus snapshot files, the original image and a text file. [file 1471-2105-13-110-S4.zip › 1471-2148-9-127-2/1471-2148-9-127-2-l_c.PNG]

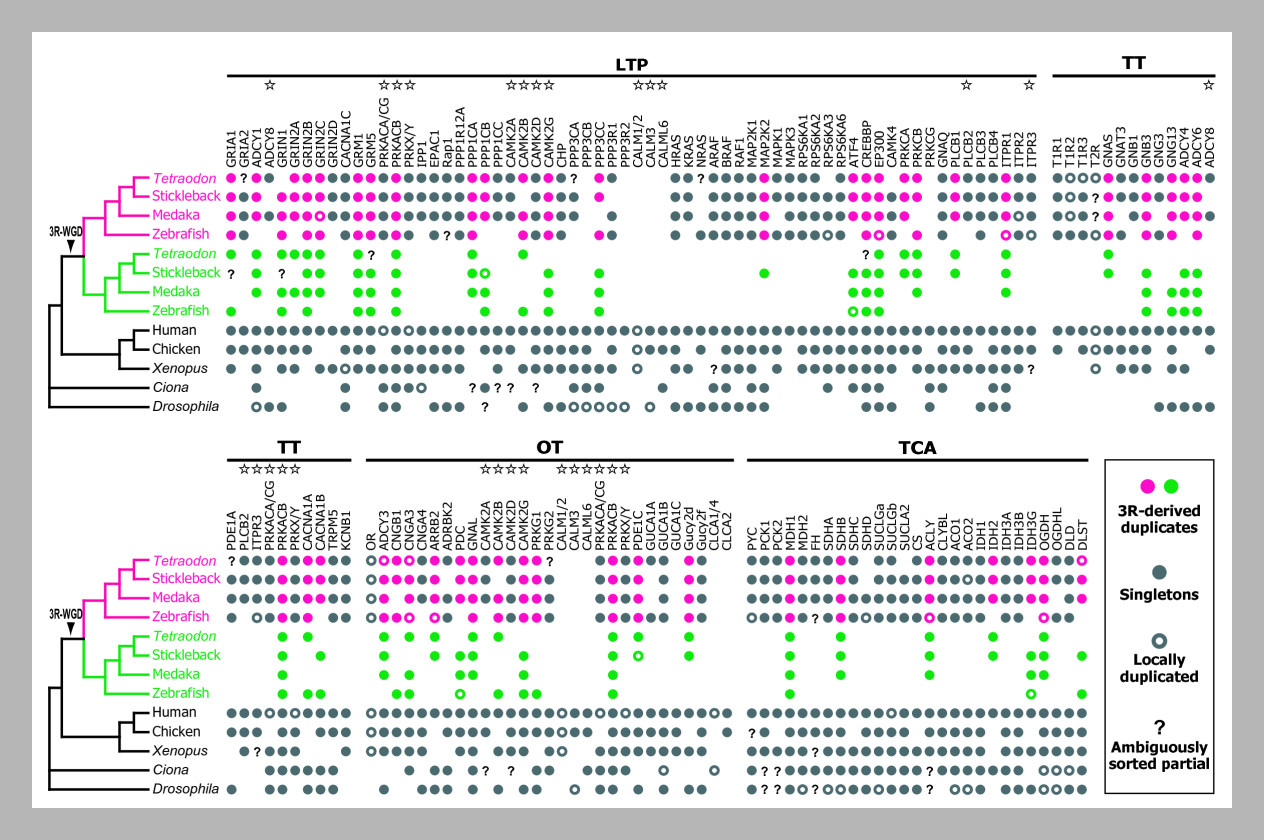

Supplement: Additional file 4 — ZIP files containing several folders, each of which with TreeSnatcher Plus snapshot files, the original image and a text file. [file 1471-2105-13-110-S4.zip › 1471-2148-9-127-2/1471-2148-9-127-2-l_o.PNG]

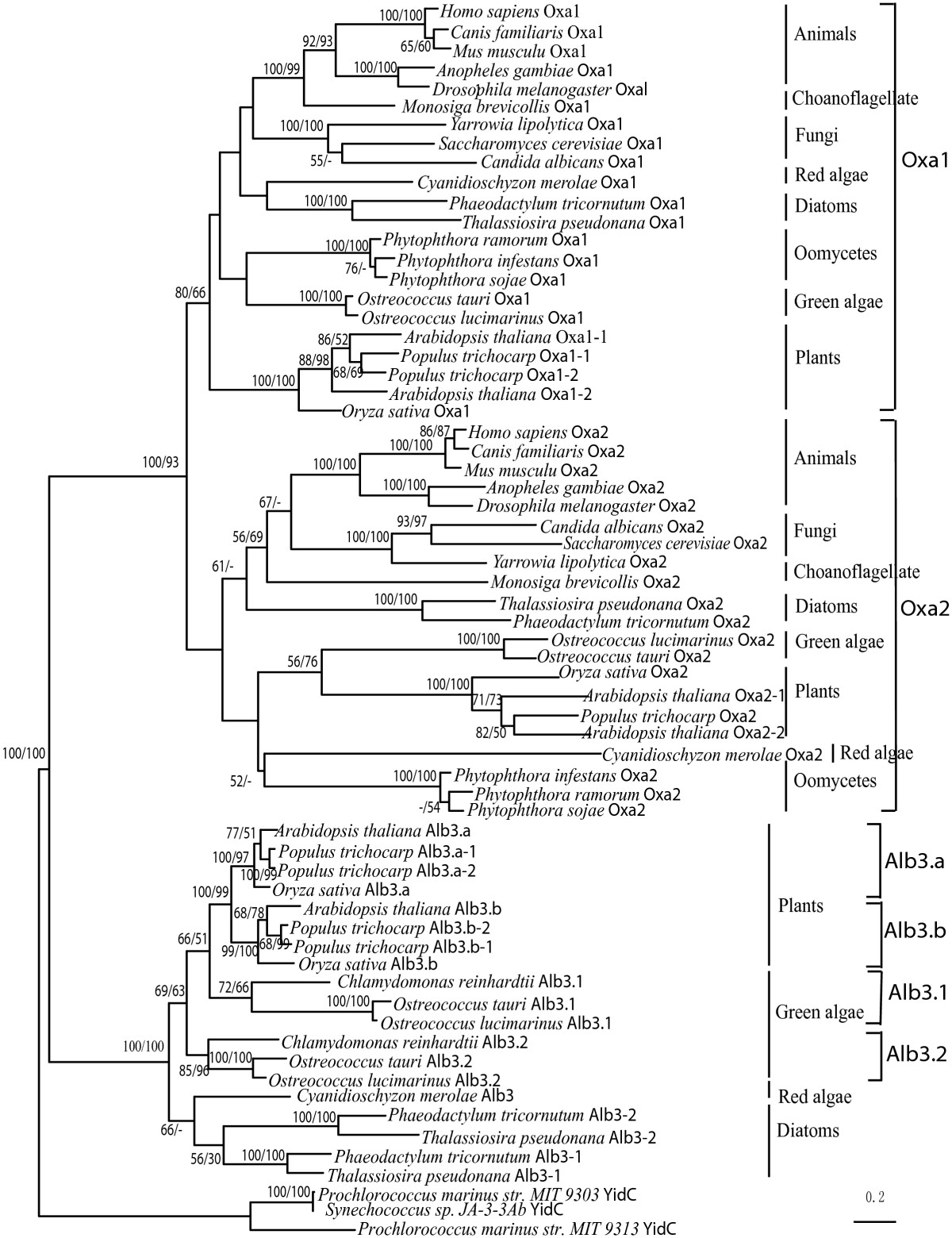

Supplement: Additional file 4 — ZIP files containing several folders, each of which with TreeSnatcher Plus snapshot files, the original image and a text file. [file 1471-2105-13-110-S4.zip › 1471-2148-9-137-2/1471-2148-9-137-2-l.jpg]

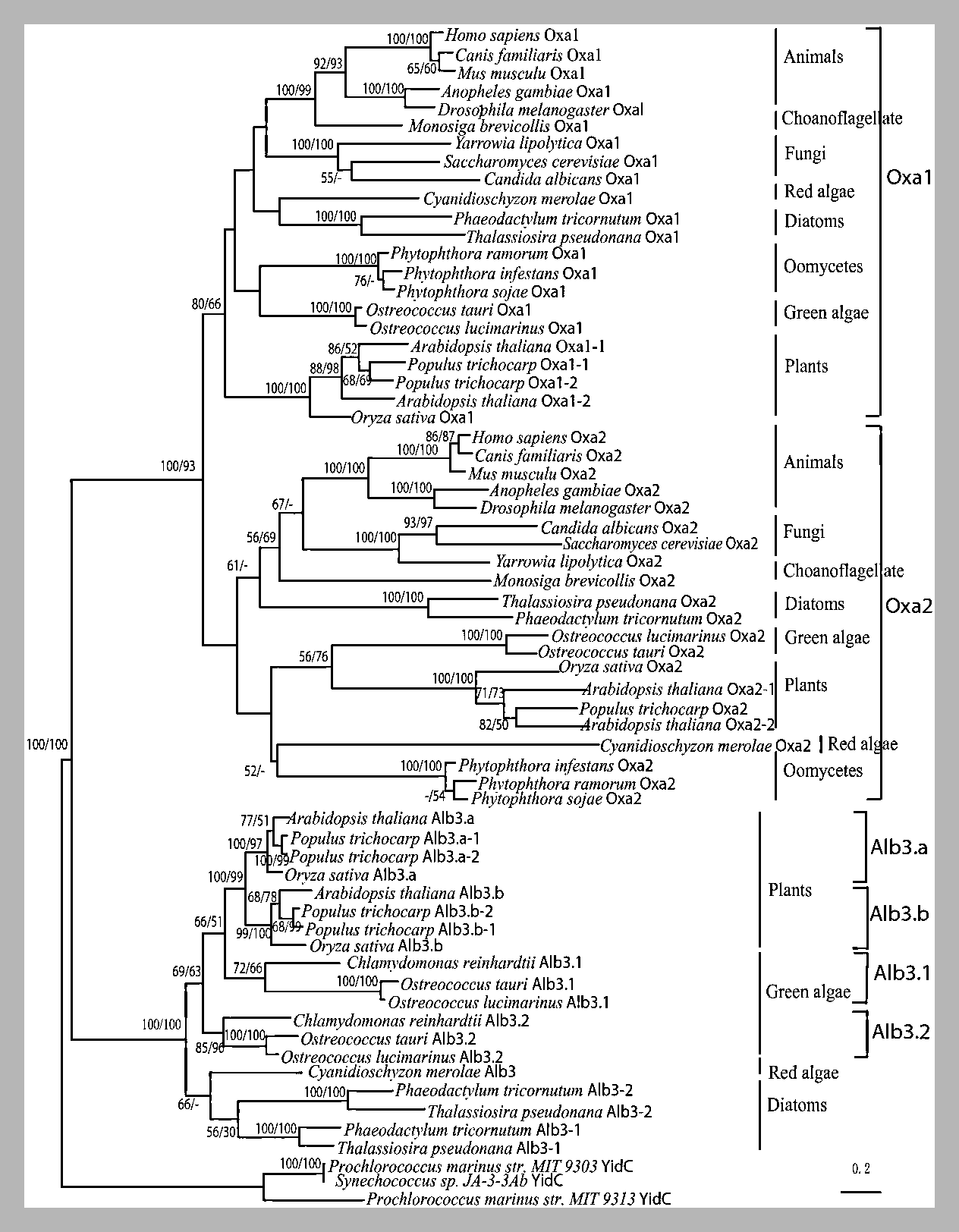

Supplement: Additional file 4 — ZIP files containing several folders, each of which with TreeSnatcher Plus snapshot files, the original image and a text file. [file 1471-2105-13-110-S4.zip › 1471-2148-9-137-2/1471-2148-9-137-2-l_b.PNG]

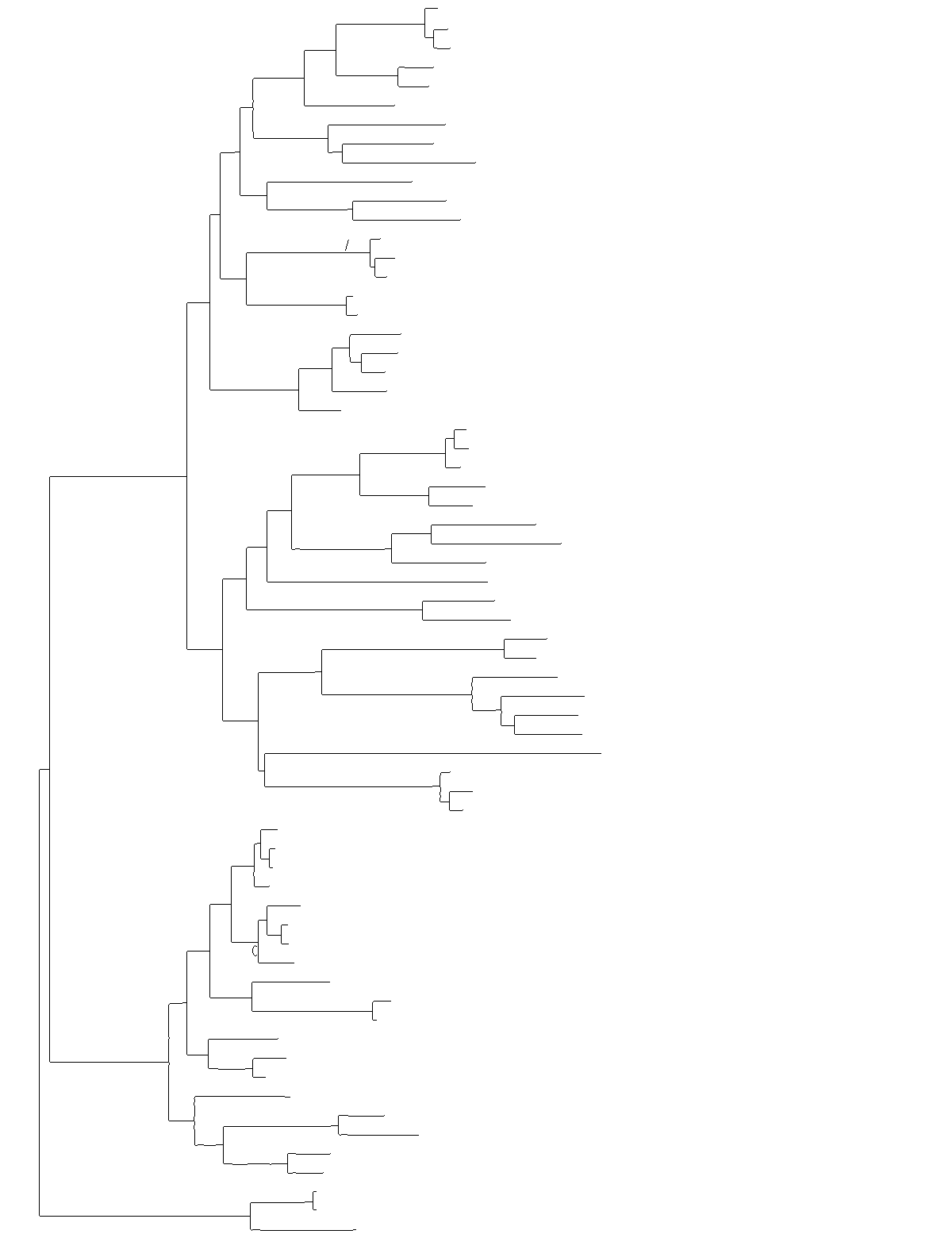

Supplement: Additional file 4 — ZIP files containing several folders, each of which with TreeSnatcher Plus snapshot files, the original image and a text file. [file 1471-2105-13-110-S4.zip › 1471-2148-9-137-2/1471-2148-9-137-2-l_c.PNG]

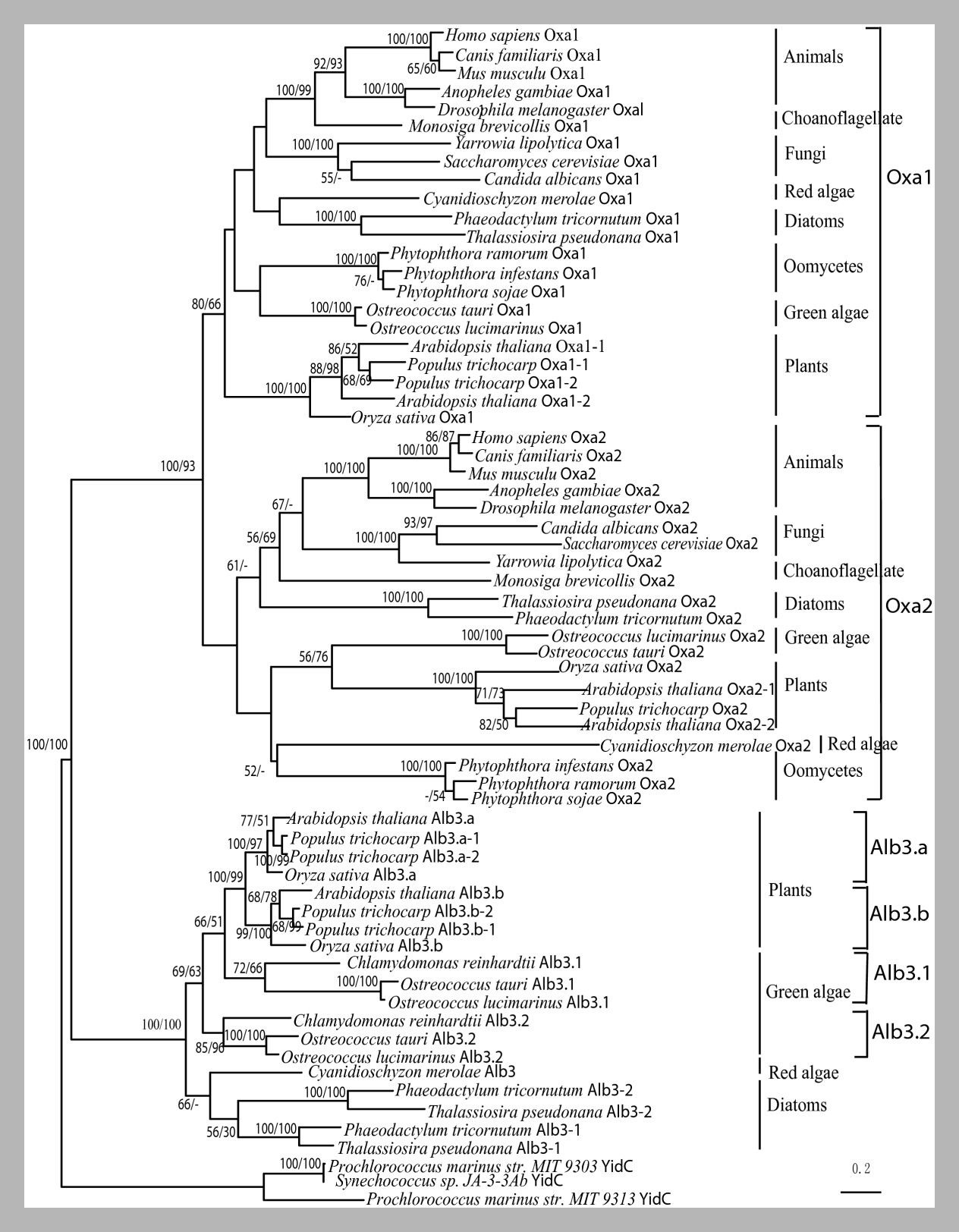

Supplement: Additional file 4 — ZIP files containing several folders, each of which with TreeSnatcher Plus snapshot files, the original image and a text file. [file 1471-2105-13-110-S4.zip › 1471-2148-9-137-2/1471-2148-9-137-2-l_o.PNG]

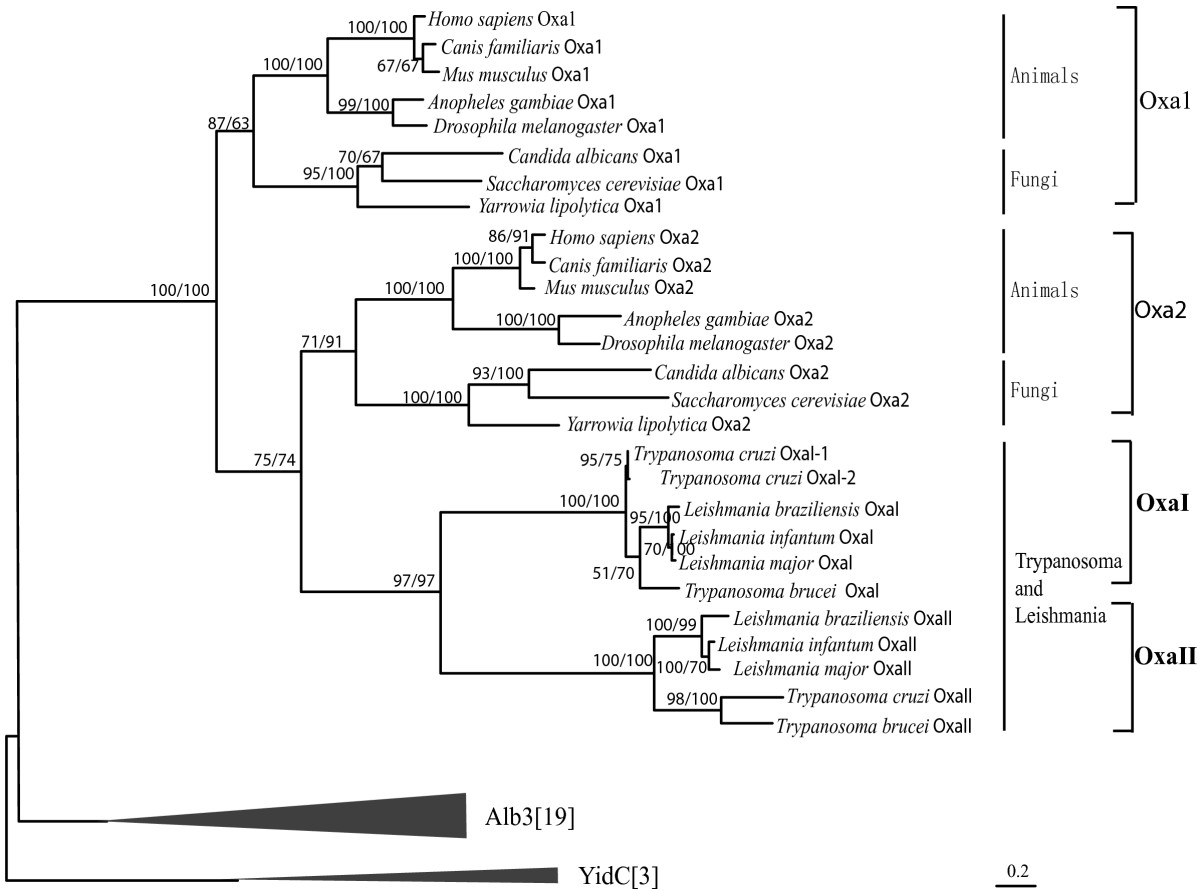

Supplement: Additional file 4 — ZIP files containing several folders, each of which with TreeSnatcher Plus snapshot files, the original image and a text file. [file 1471-2105-13-110-S4.zip › 1471-2148-9-137-3/1471-2148-9-137-3-l.jpg]

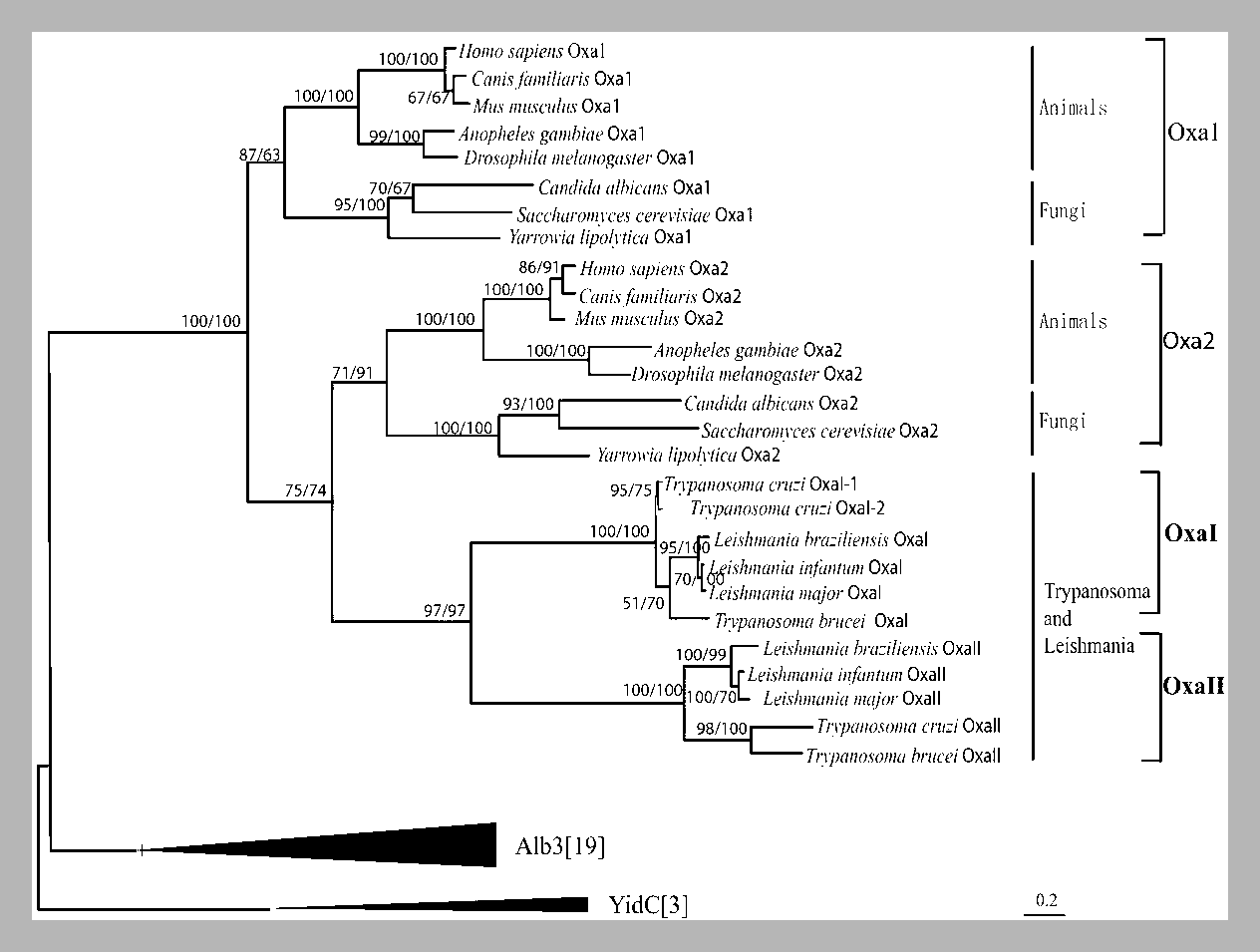

Supplement: Additional file 4 — ZIP files containing several folders, each of which with TreeSnatcher Plus snapshot files, the original image and a text file. [file 1471-2105-13-110-S4.zip › 1471-2148-9-137-3/1471-2148-9-137-3-l_b.PNG]

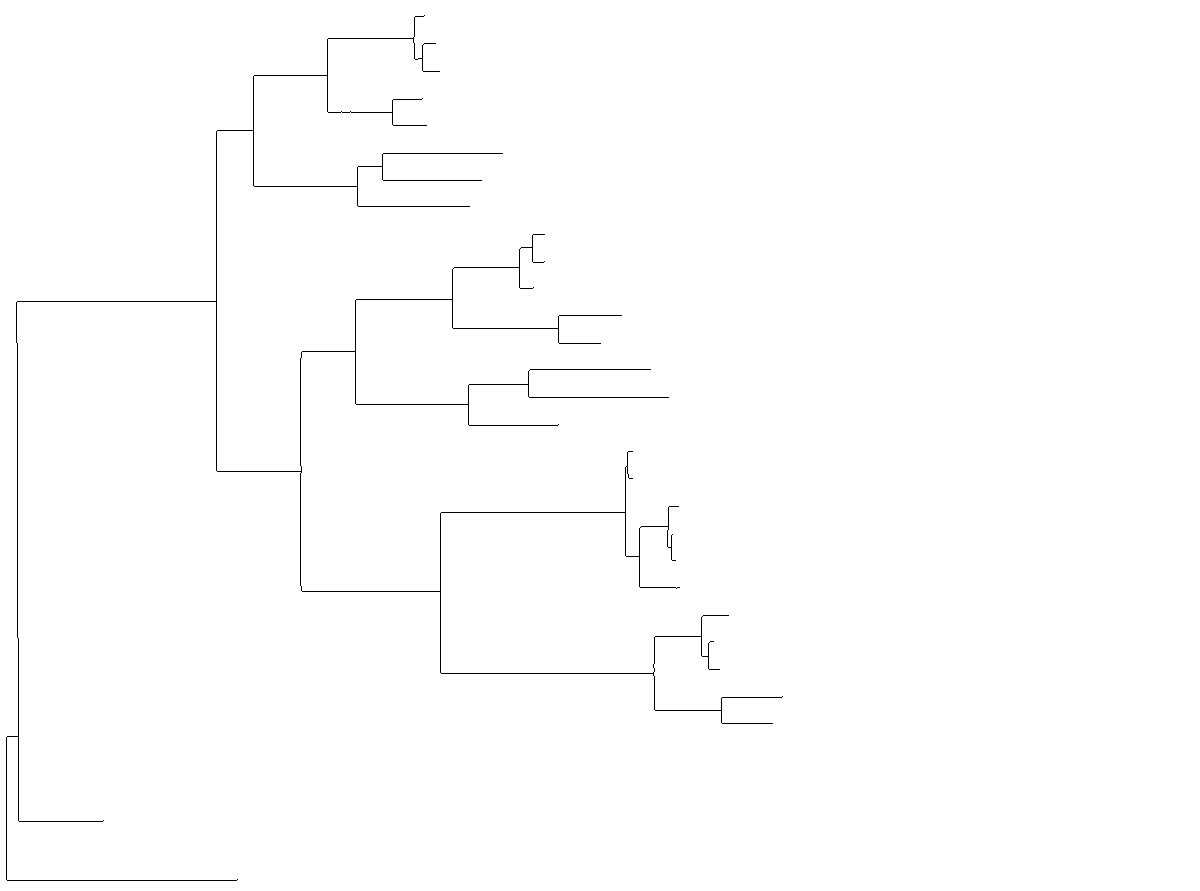

Supplement: Additional file 4 — ZIP files containing several folders, each of which with TreeSnatcher Plus snapshot files, the original image and a text file. [file 1471-2105-13-110-S4.zip › 1471-2148-9-137-3/1471-2148-9-137-3-l_c.PNG]

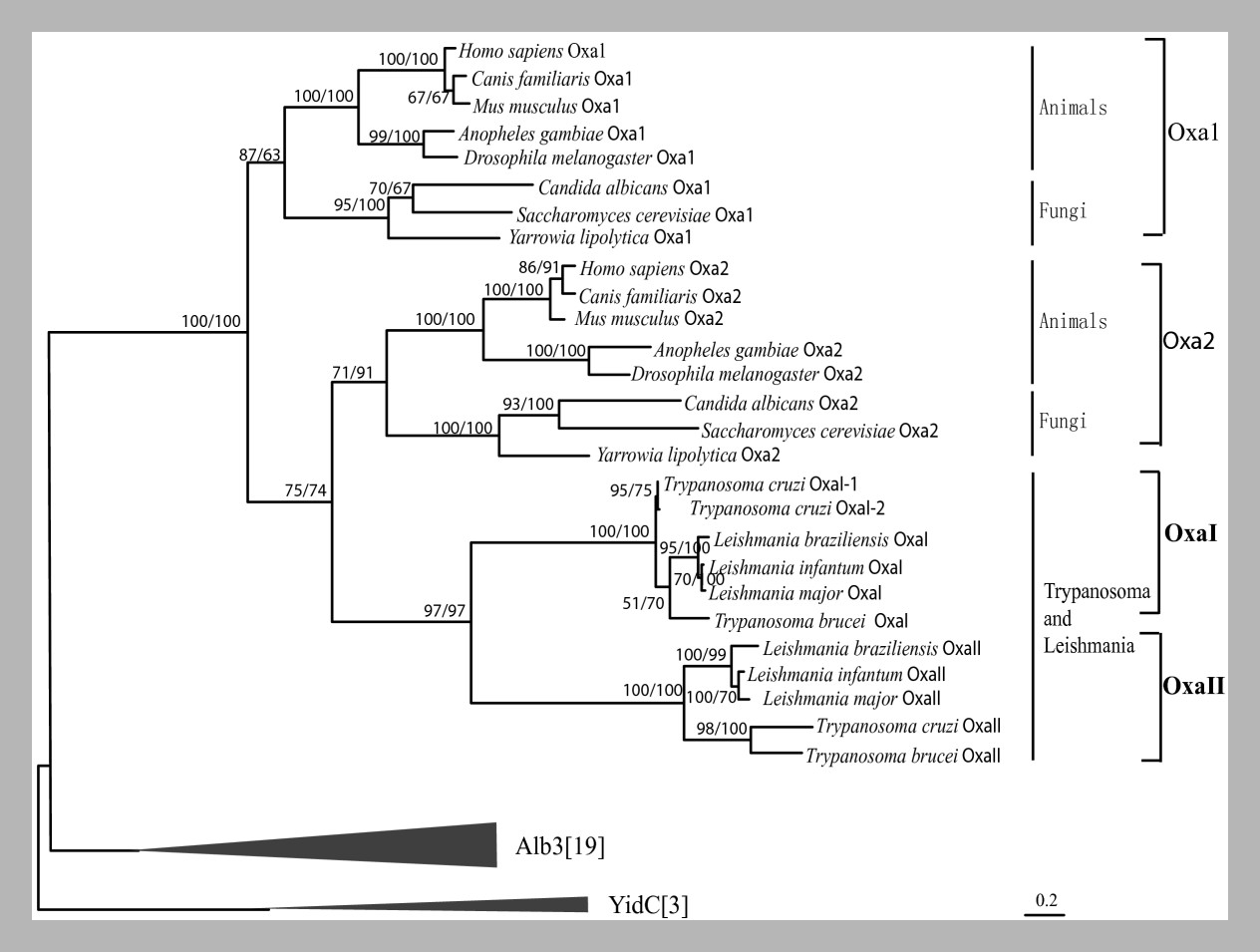

Supplement: Additional file 4 — ZIP files containing several folders, each of which with TreeSnatcher Plus snapshot files, the original image and a text file. [file 1471-2105-13-110-S4.zip › 1471-2148-9-137-3/1471-2148-9-137-3-l_o.PNG]

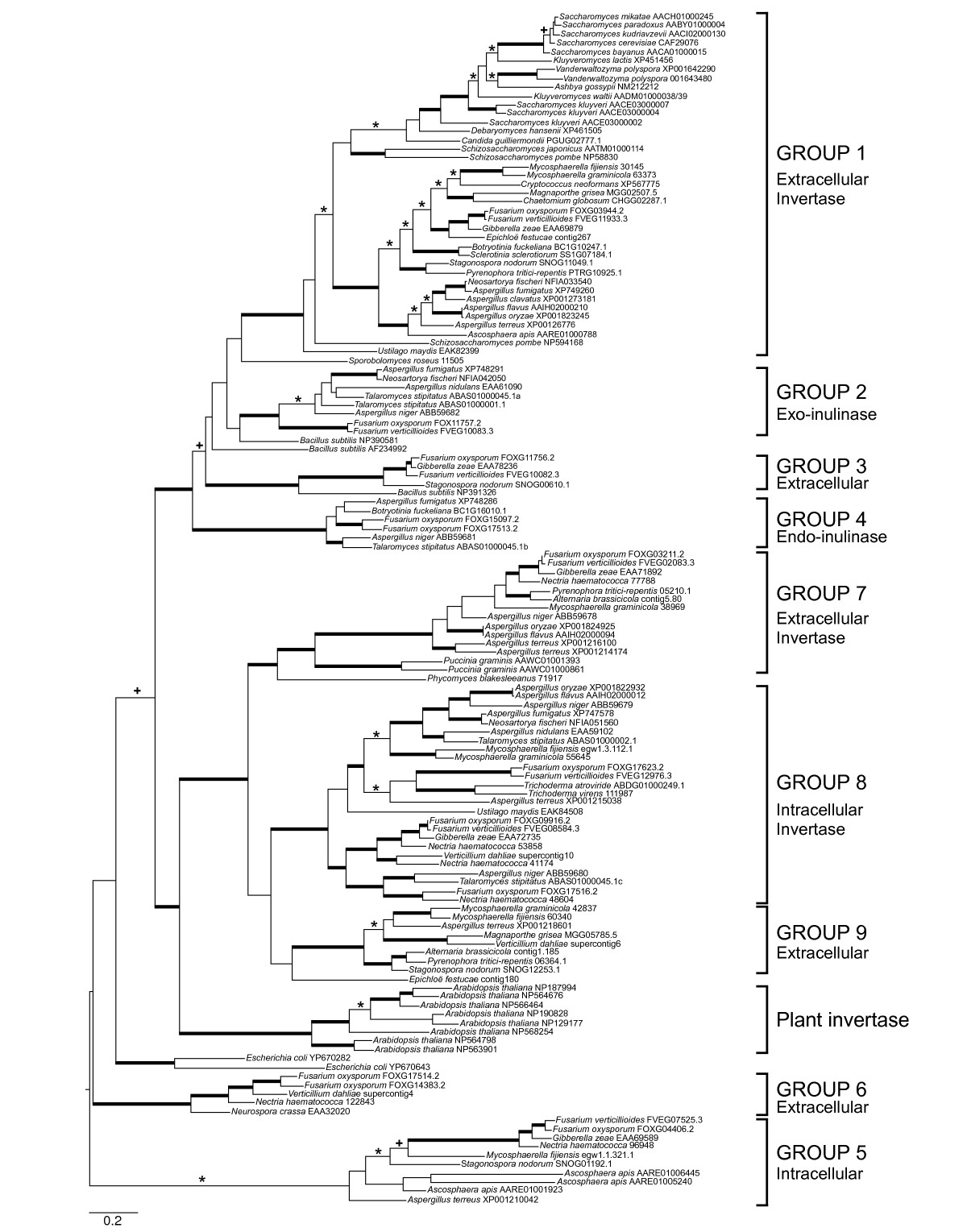

Supplement: Additional file 4 — ZIP files containing several folders, each of which with TreeSnatcher Plus snapshot files, the original image and a text file. [file 1471-2105-13-110-S4.zip › 1471-2148-9-148-1/1471-2148-9-148-1-l.jpg]

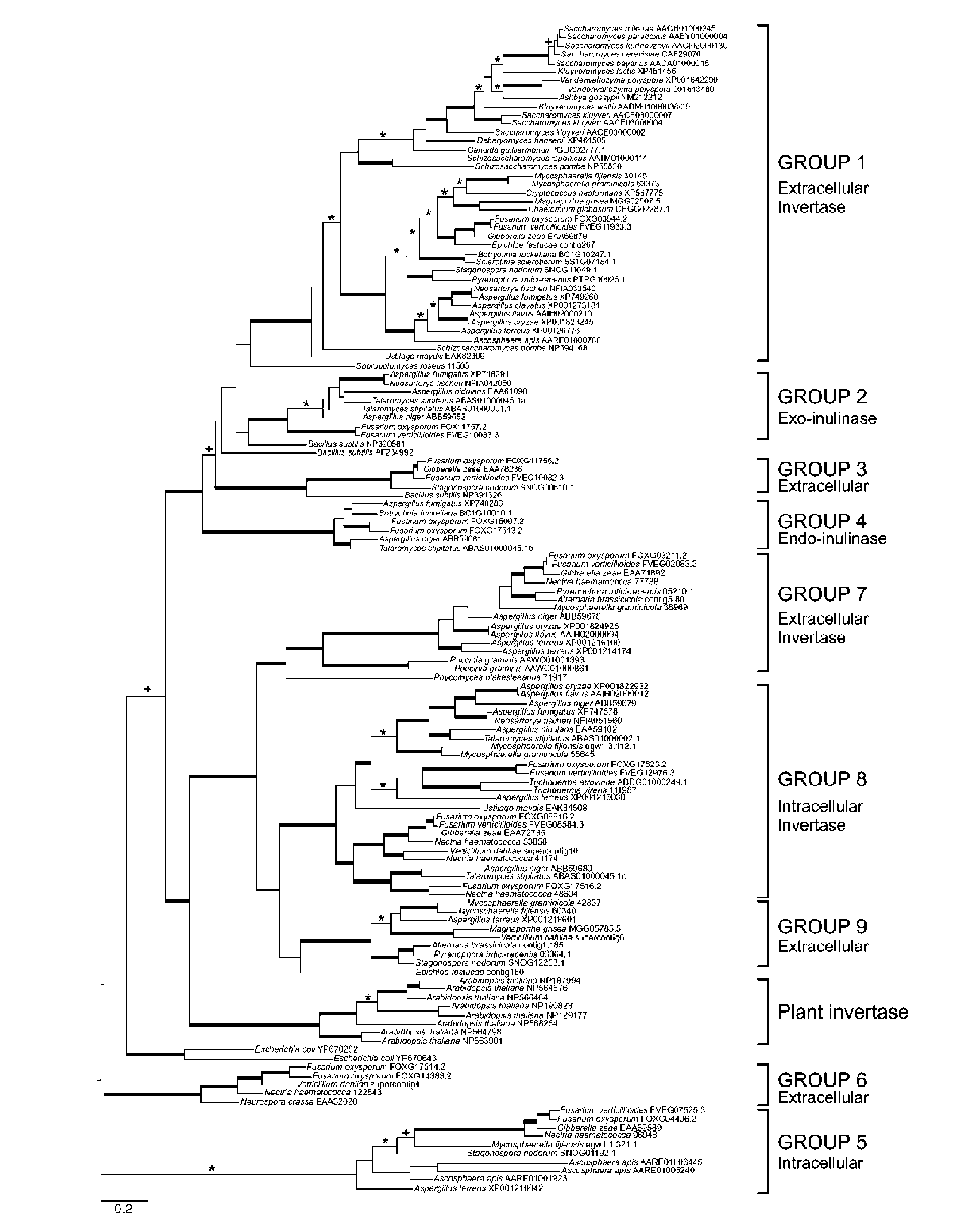

Supplement: Additional file 4 — ZIP files containing several folders, each of which with TreeSnatcher Plus snapshot files, the original image and a text file. [file 1471-2105-13-110-S4.zip › 1471-2148-9-148-1/1471-2148-9-148-1-l_b.PNG]

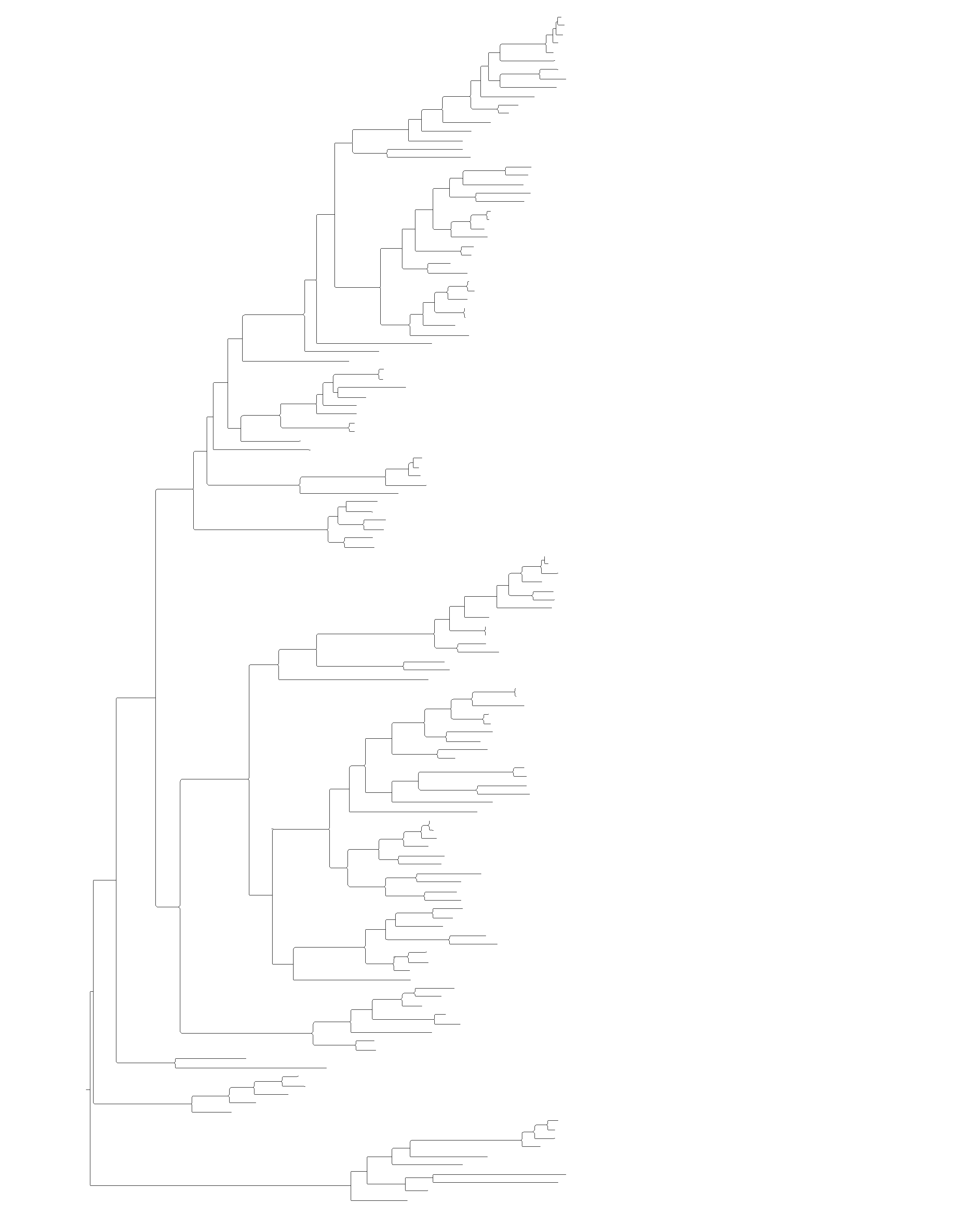

Supplement: Additional file 4 — ZIP files containing several folders, each of which with TreeSnatcher Plus snapshot files, the original image and a text file. [file 1471-2105-13-110-S4.zip › 1471-2148-9-148-1/1471-2148-9-148-1-l_c.PNG]

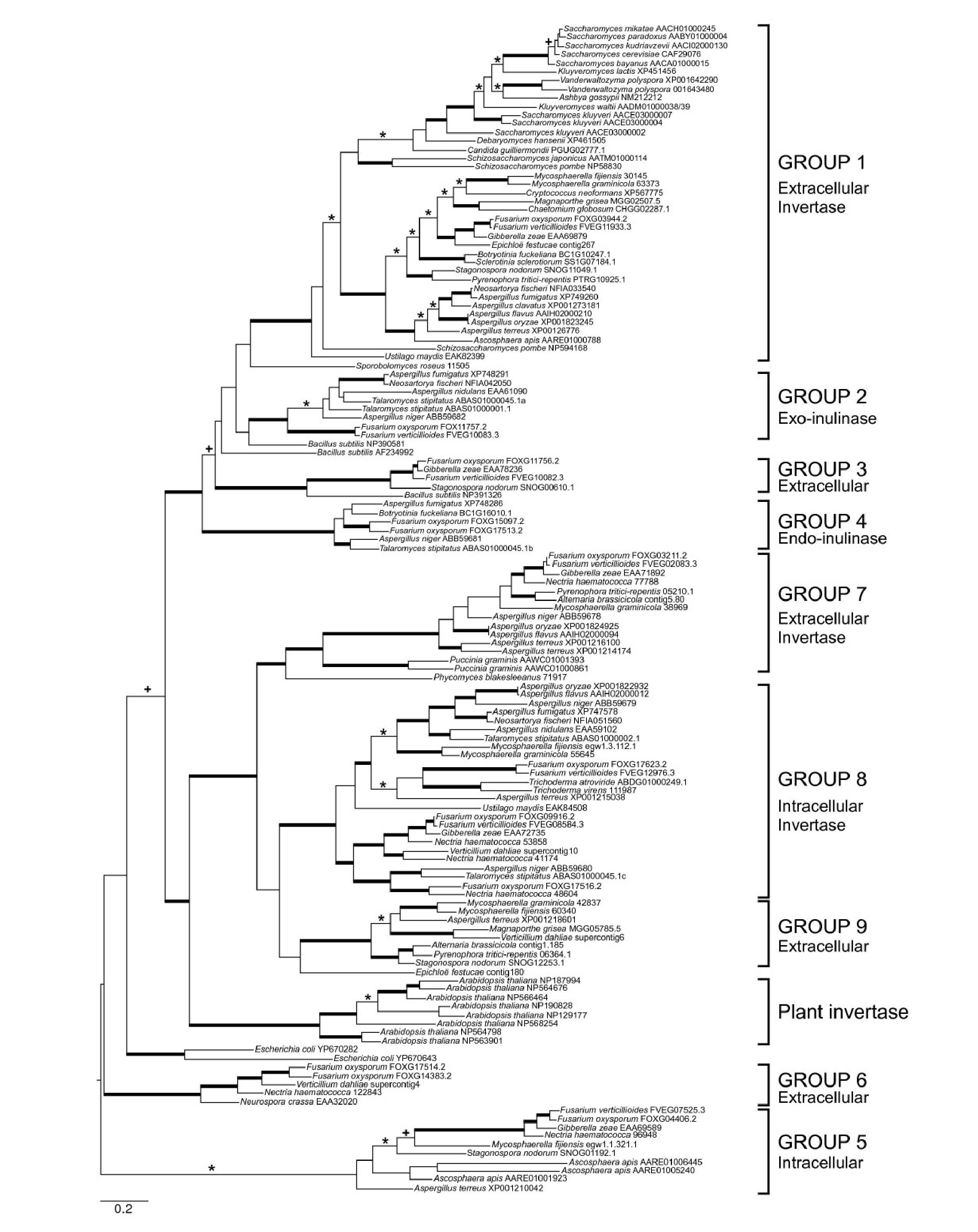

Supplement: Additional file 4 — ZIP files containing several folders, each of which with TreeSnatcher Plus snapshot files, the original image and a text file. [file 1471-2105-13-110-S4.zip › 1471-2148-9-148-1/1471-2148-9-148-1-l_o.PNG]

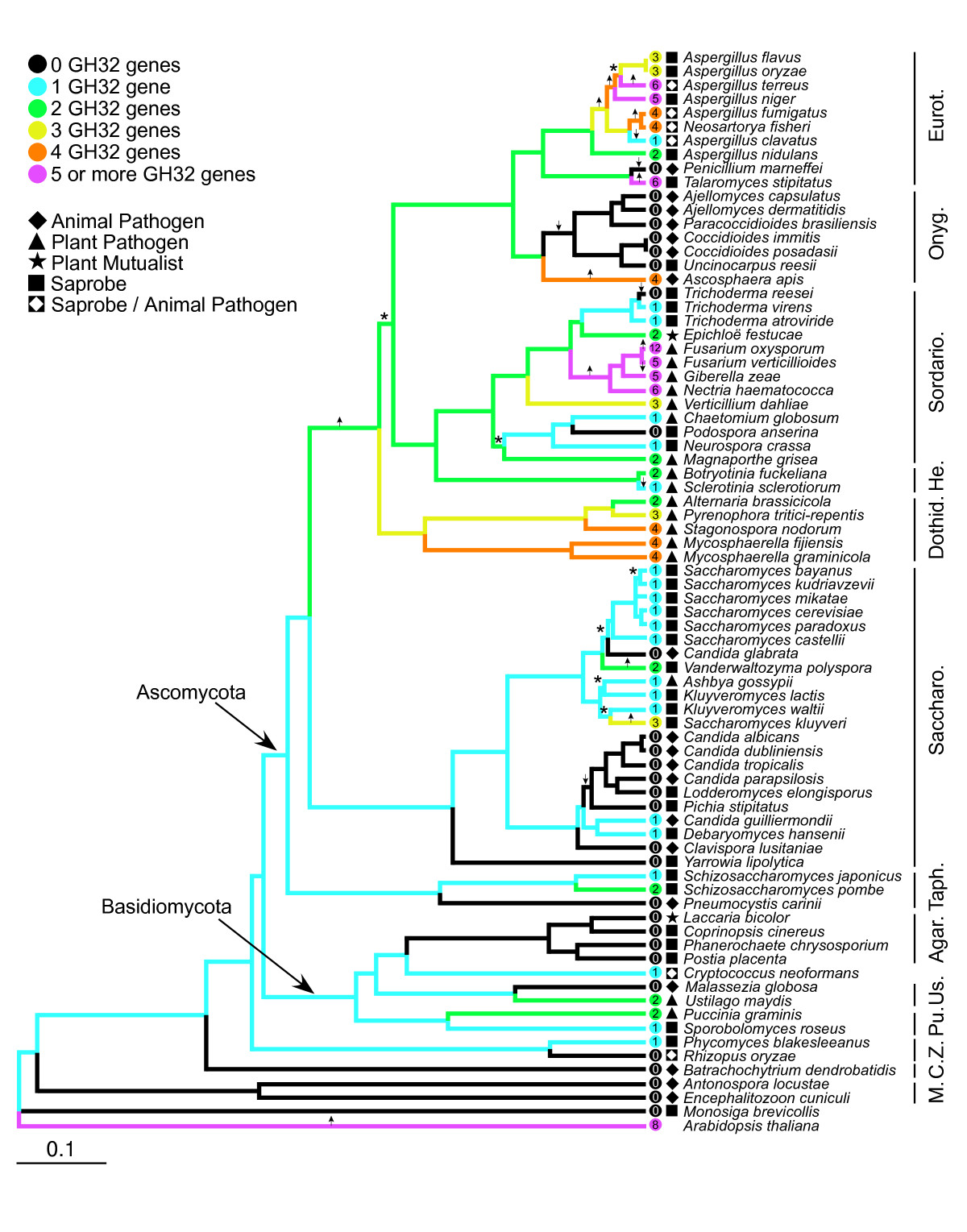

Supplement: Additional file 4 — ZIP files containing several folders, each of which with TreeSnatcher Plus snapshot files, the original image and a text file. [file 1471-2105-13-110-S4.zip › 1471-2148-9-148-3/1471-2148-9-148-3-l.jpg]

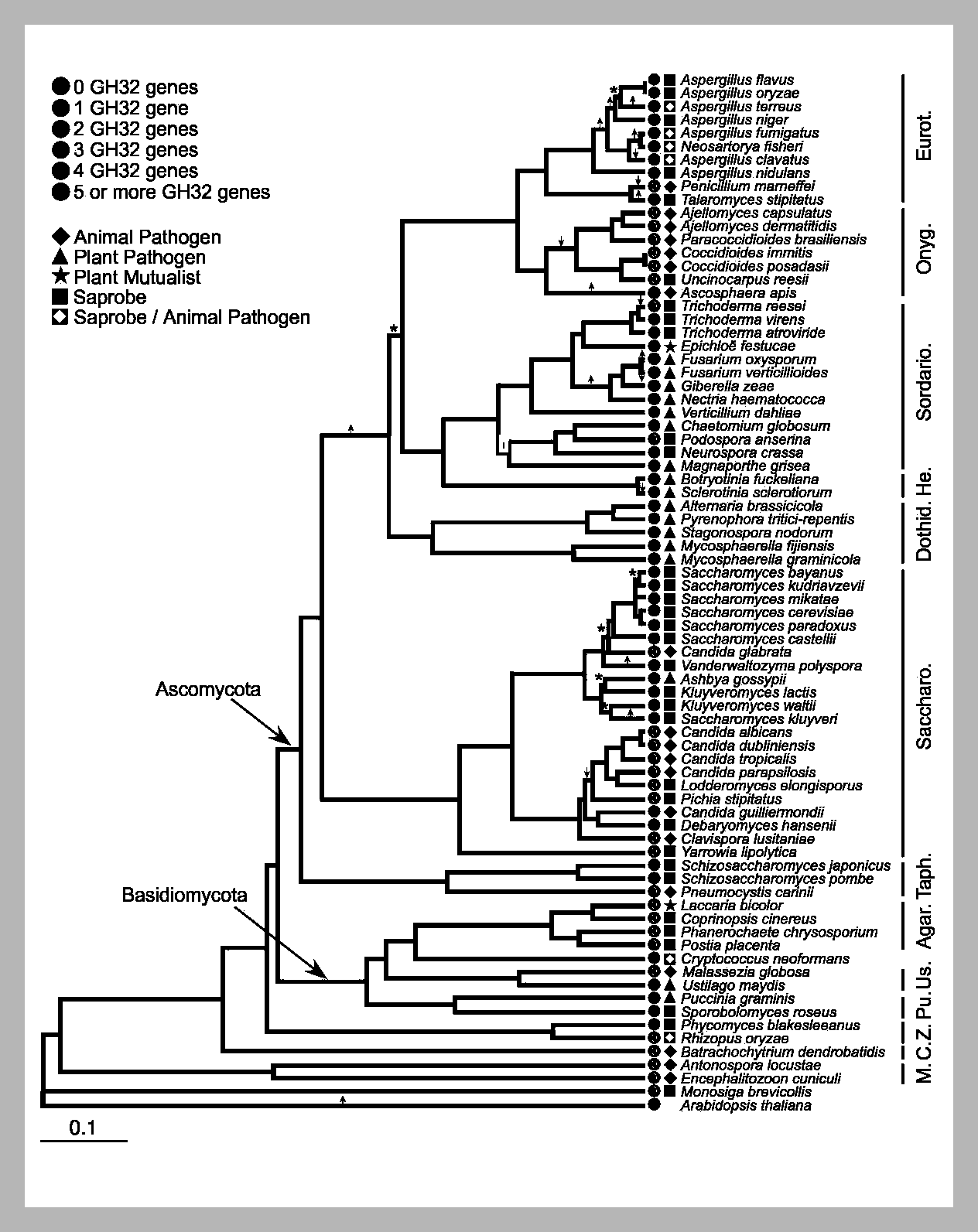

Supplement: Additional file 4 — ZIP files containing several folders, each of which with TreeSnatcher Plus snapshot files, the original image and a text file. [file 1471-2105-13-110-S4.zip › 1471-2148-9-148-3/1471-2148-9-148-3-l_b.PNG]

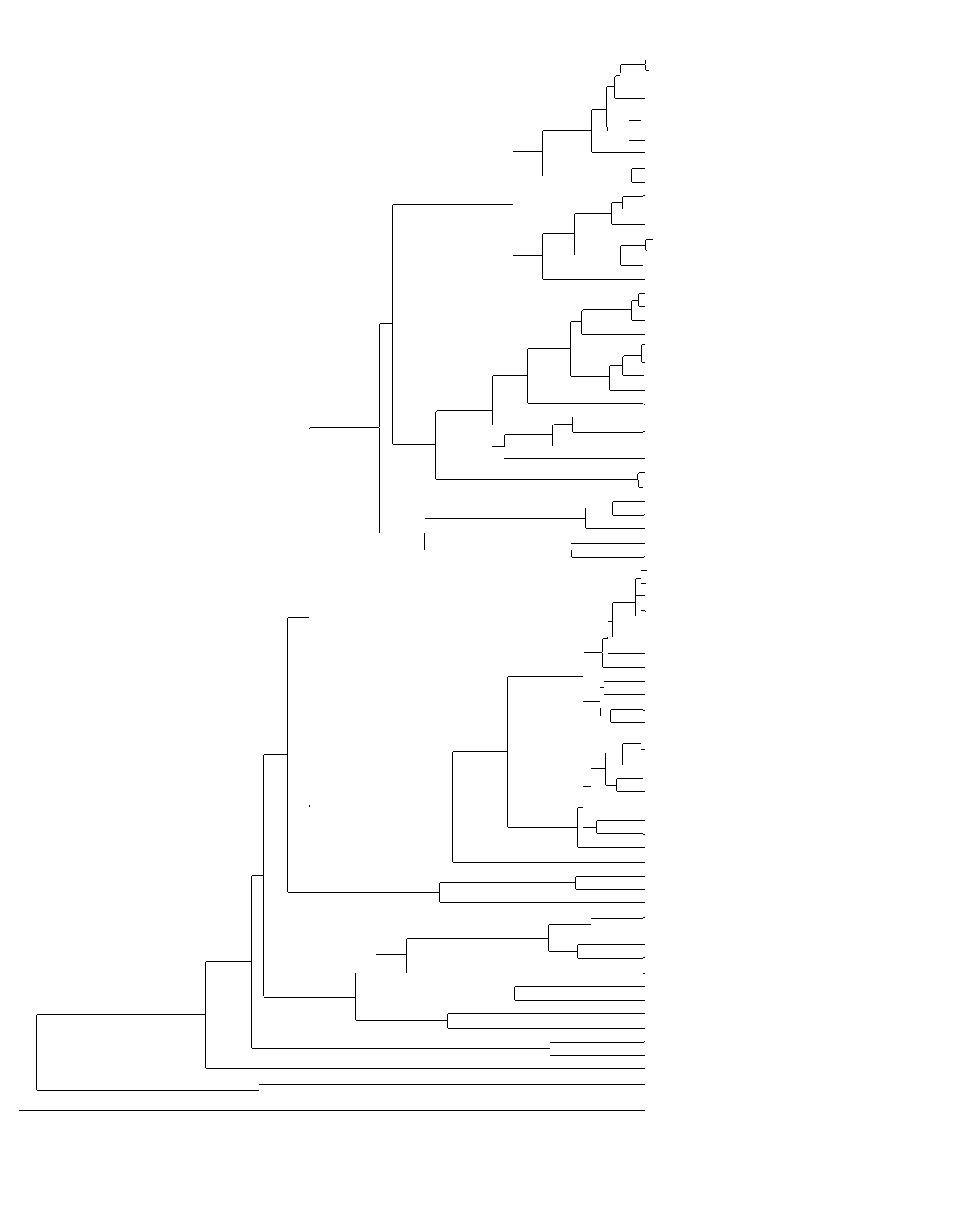

Supplement: Additional file 4 — ZIP files containing several folders, each of which with TreeSnatcher Plus snapshot files, the original image and a text file. [file 1471-2105-13-110-S4.zip › 1471-2148-9-148-3/1471-2148-9-148-3-l_c.PNG]

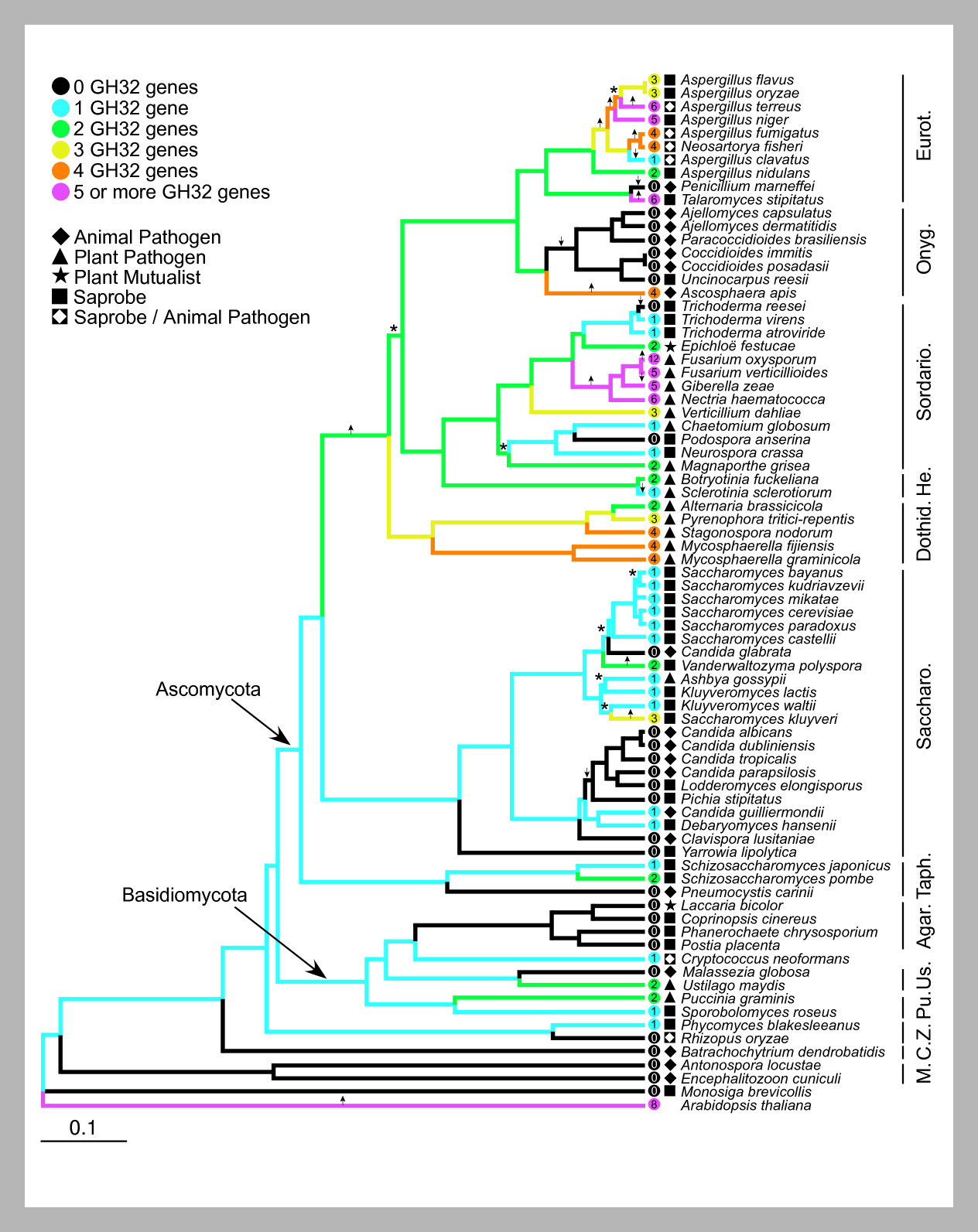

Supplement: Additional file 4 — ZIP files containing several folders, each of which with TreeSnatcher Plus snapshot files, the original image and a text file. [file 1471-2105-13-110-S4.zip › 1471-2148-9-148-3/1471-2148-9-148-3-l_o.PNG]
