# Supplementary figures and images for: TreeSnatcher plus: capturing phylogenetic trees from images (part 4 of 5)
Source: BMC Bioinformatics. 2012 May 24;13:110. doi: 10.1186/1471-2105-13-110 (PMC3411374; doi:10.1186/1471-2105-13-110)

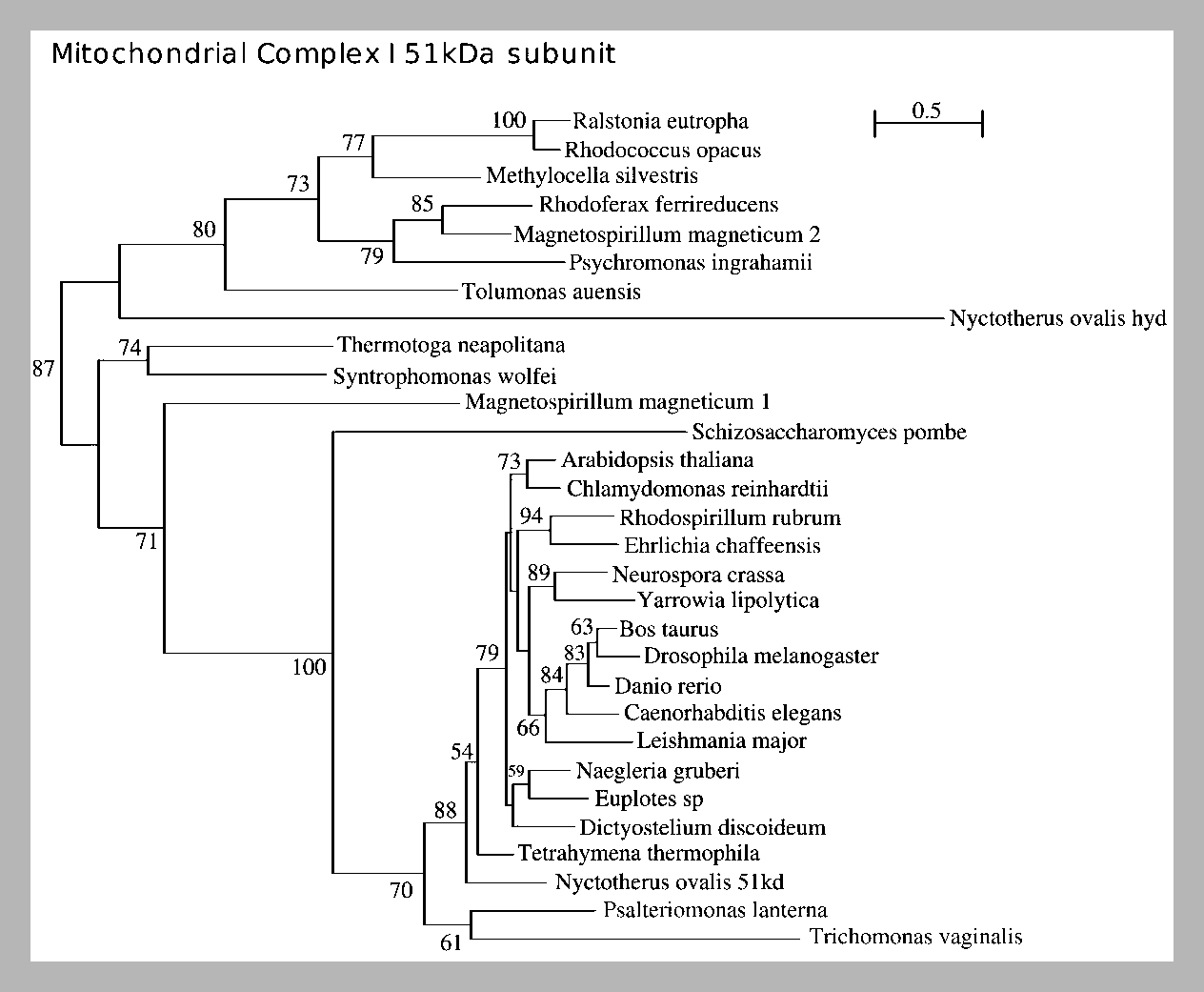

Supplement: Additional file 5 — ZIP files containing several folders, each of which with TreeSnatcher Plus snapshot files, the original image and a text file. [file 1471-2105-13-110-S5.zip › 1471-2148-9-287-10/1471-2148-9-287-10-l_b.PNG]

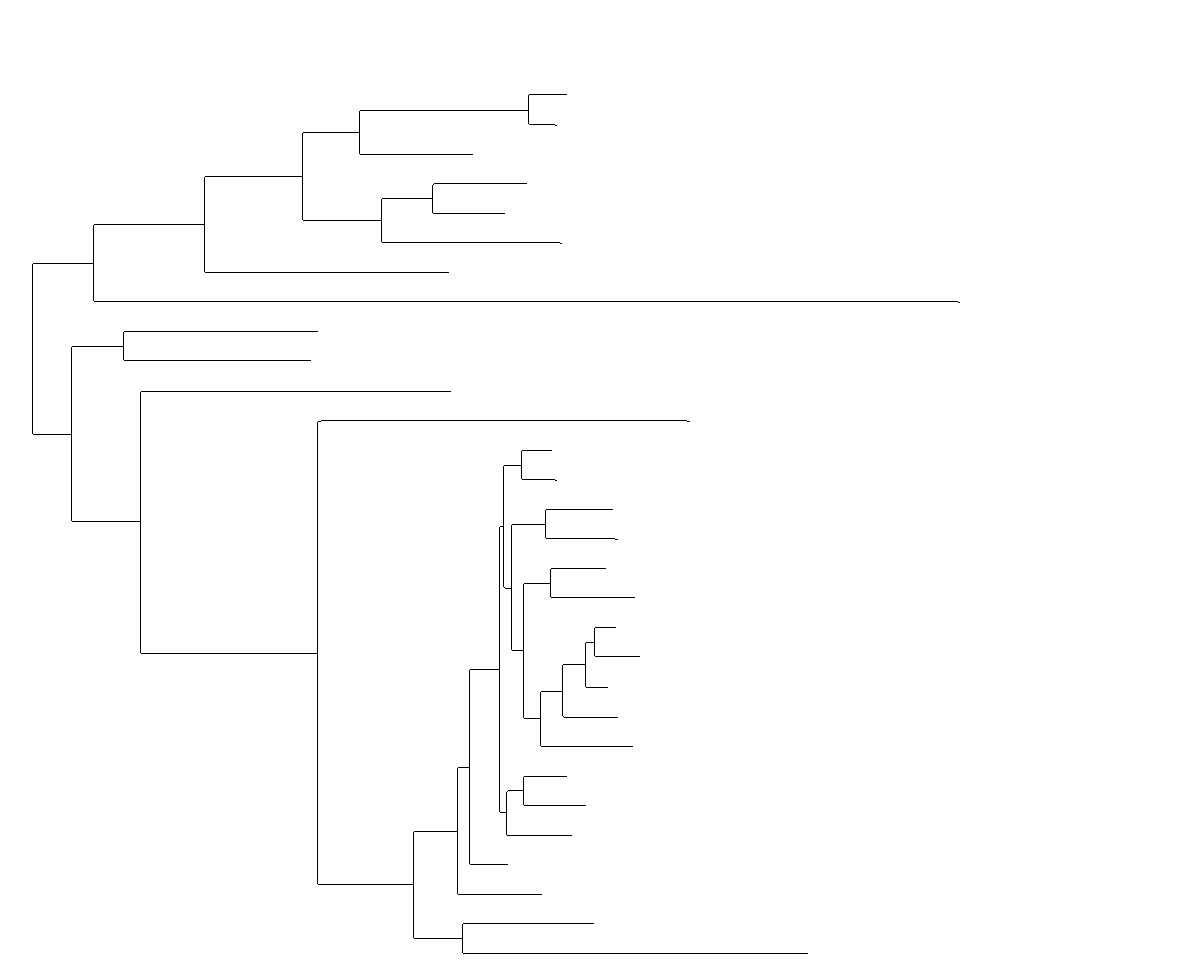

Supplement: Additional file 5 — ZIP files containing several folders, each of which with TreeSnatcher Plus snapshot files, the original image and a text file. [file 1471-2105-13-110-S5.zip › 1471-2148-9-287-10/1471-2148-9-287-10-l_c.PNG]

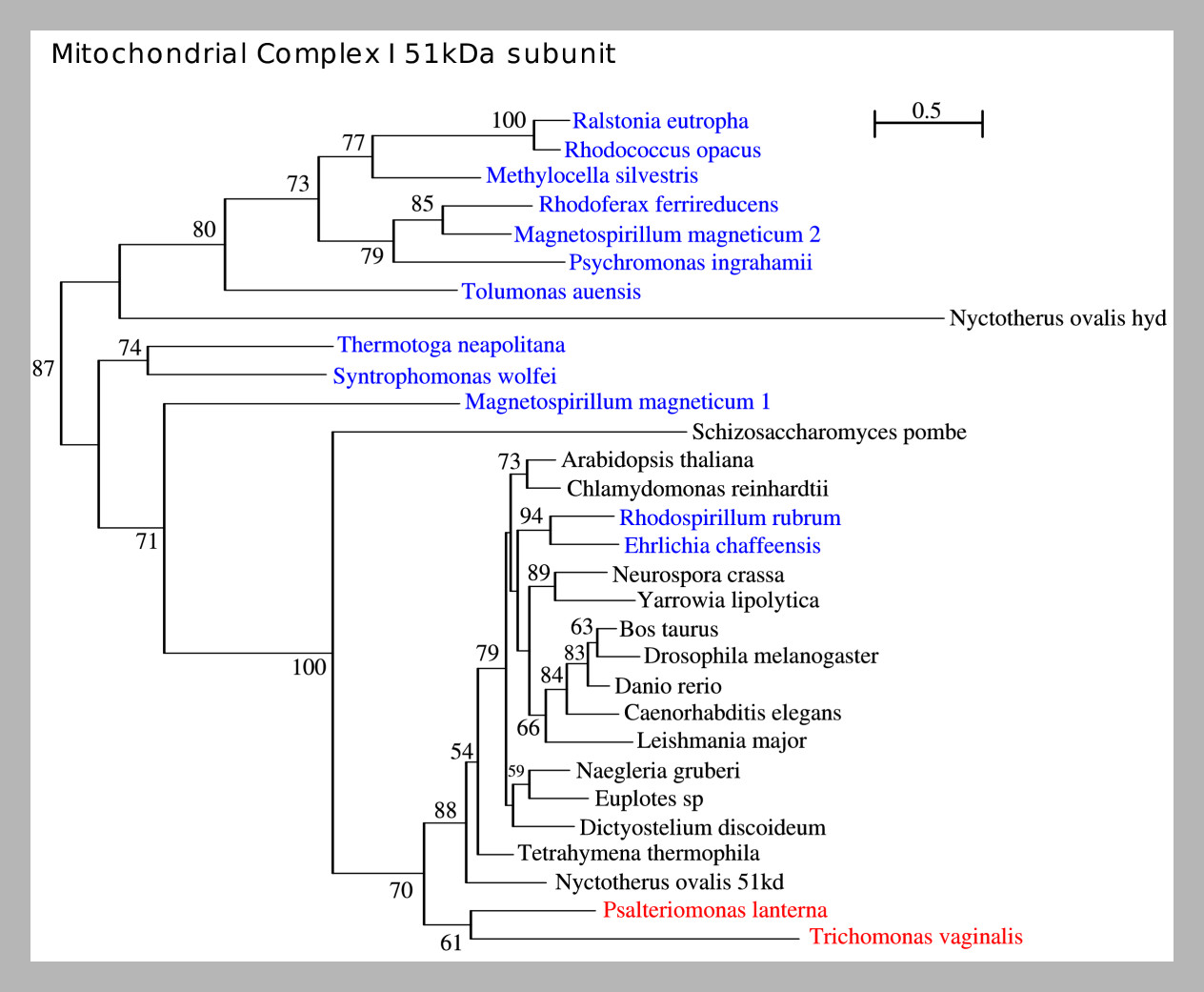

Supplement: Additional file 5 — ZIP files containing several folders, each of which with TreeSnatcher Plus snapshot files, the original image and a text file. [file 1471-2105-13-110-S5.zip › 1471-2148-9-287-10/1471-2148-9-287-10-l_o.PNG]

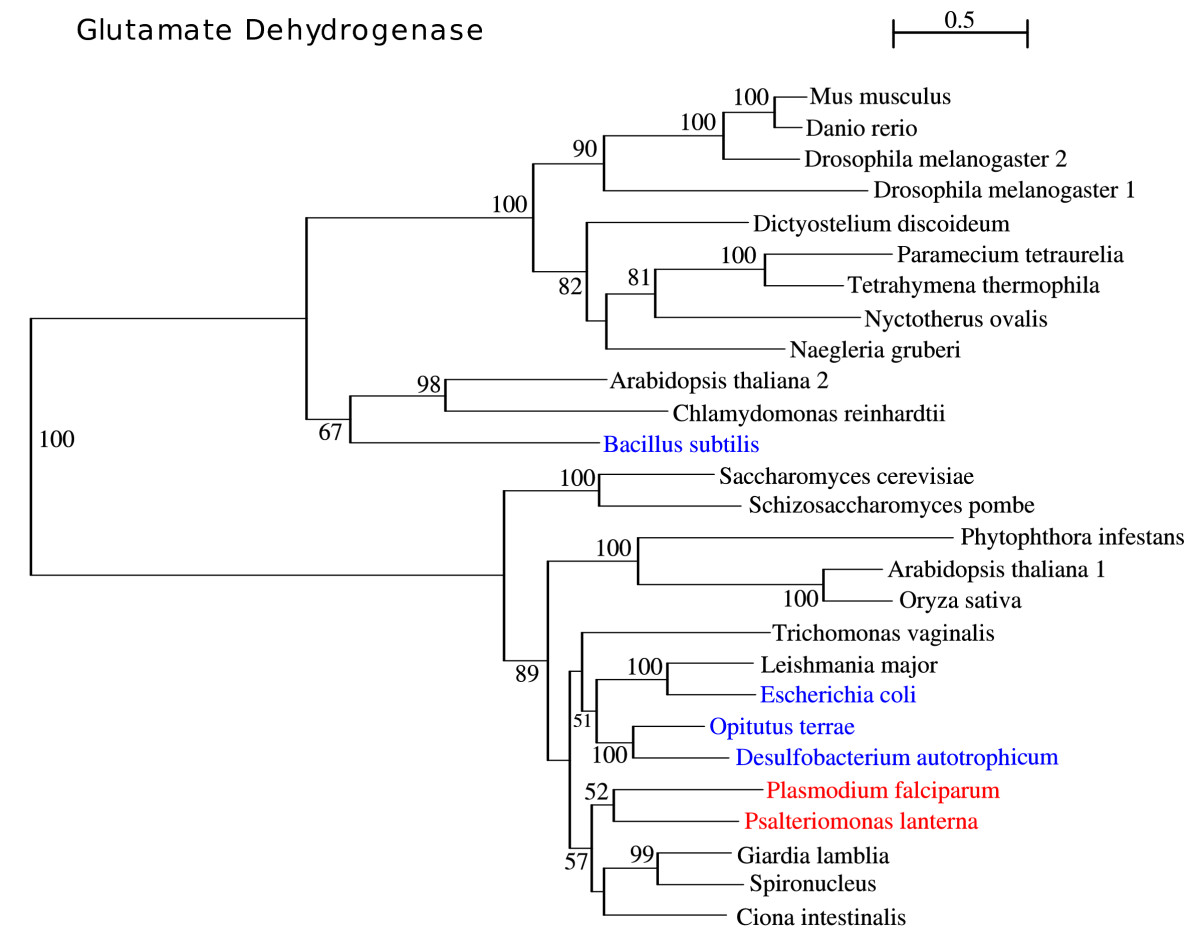

Supplement: Additional file 5 — ZIP files containing several folders, each of which with TreeSnatcher Plus snapshot files, the original image and a text file. [file 1471-2105-13-110-S5.zip › 1471-2148-9-287-11/1471-2148-9-287-11-l.jpg]

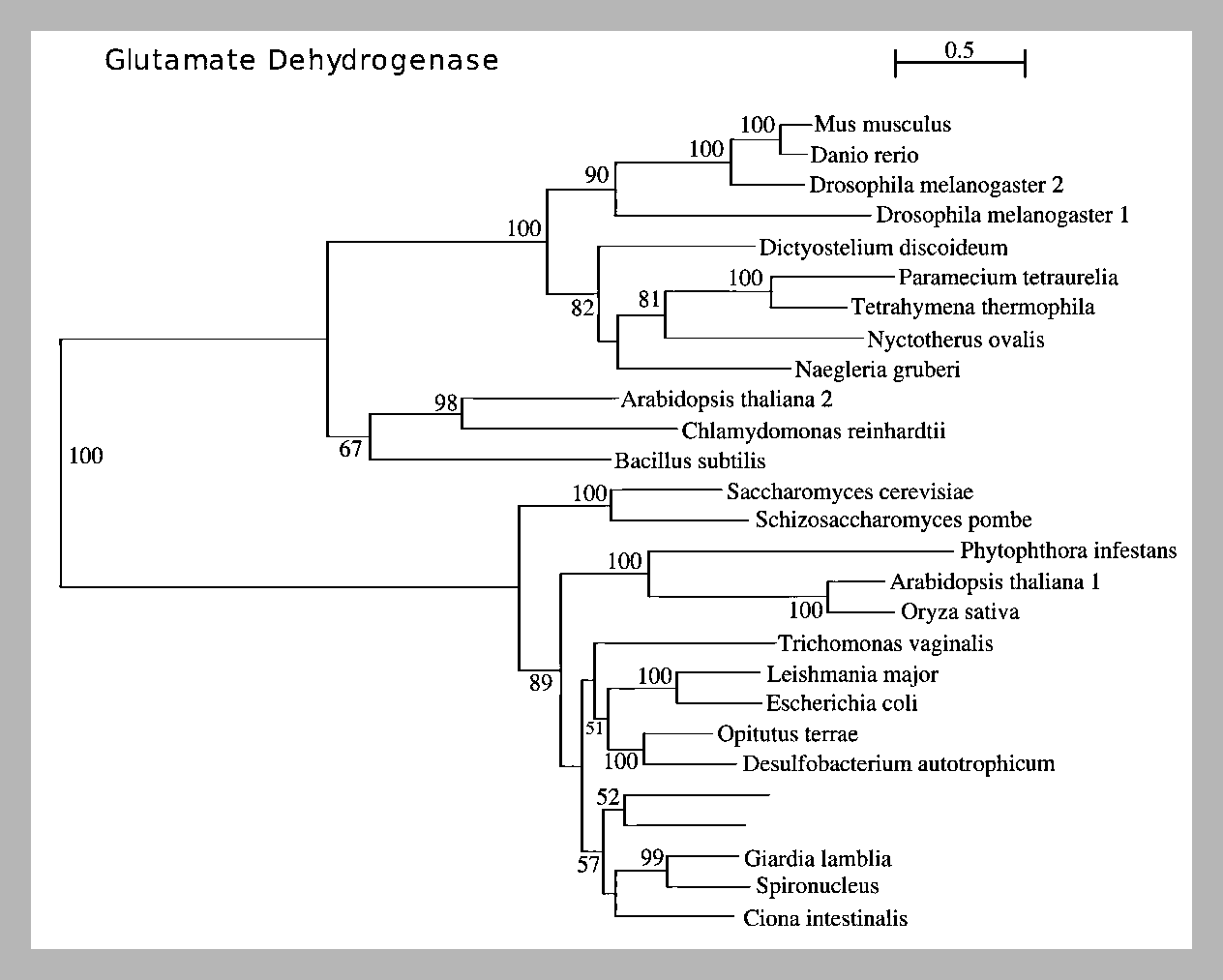

Supplement: Additional file 5 — ZIP files containing several folders, each of which with TreeSnatcher Plus snapshot files, the original image and a text file. [file 1471-2105-13-110-S5.zip › 1471-2148-9-287-11/1471-2148-9-287-11-l_b.PNG]

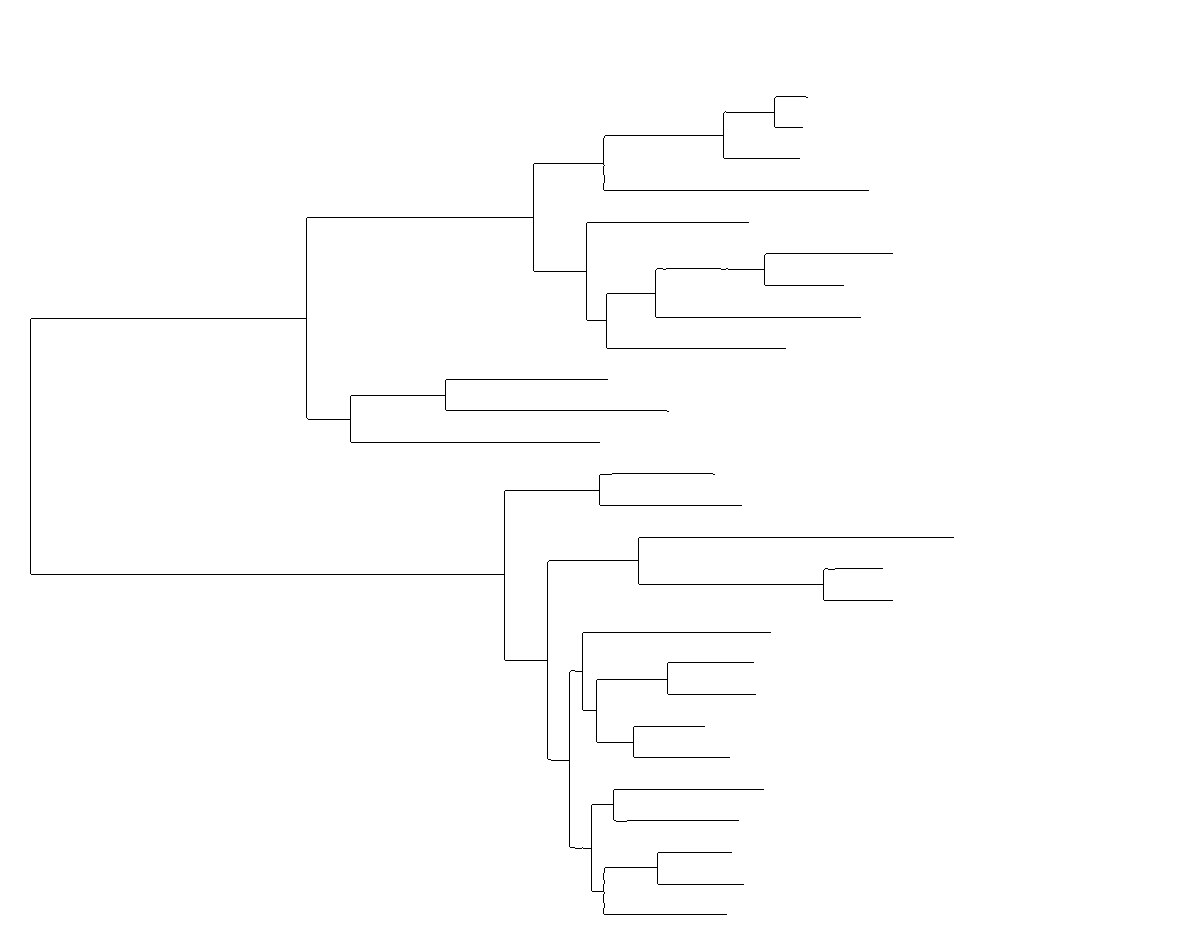

Supplement: Additional file 5 — ZIP files containing several folders, each of which with TreeSnatcher Plus snapshot files, the original image and a text file. [file 1471-2105-13-110-S5.zip › 1471-2148-9-287-11/1471-2148-9-287-11-l_c.PNG]

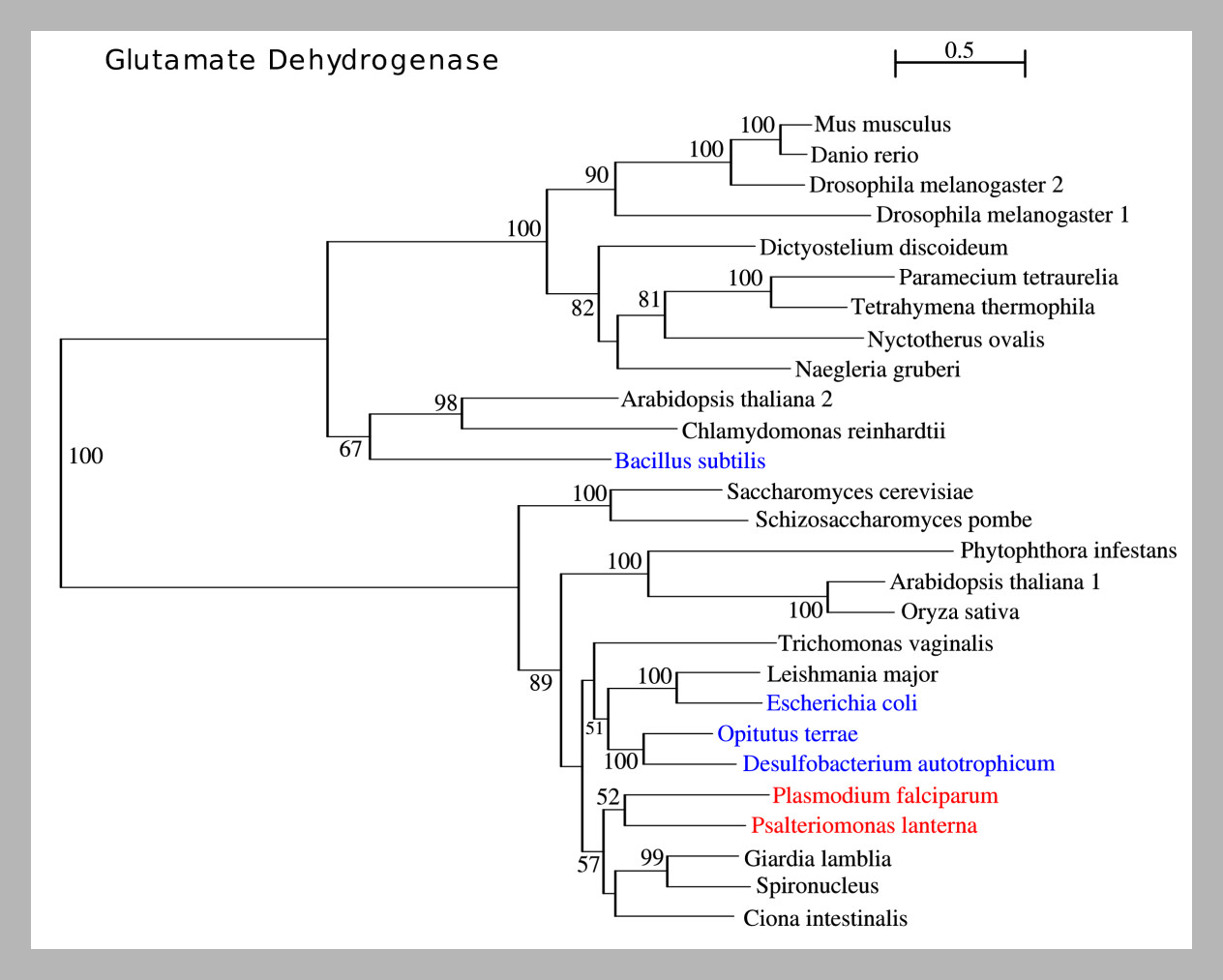

Supplement: Additional file 5 — ZIP files containing several folders, each of which with TreeSnatcher Plus snapshot files, the original image and a text file. [file 1471-2105-13-110-S5.zip › 1471-2148-9-287-11/1471-2148-9-287-11-l_o.PNG]

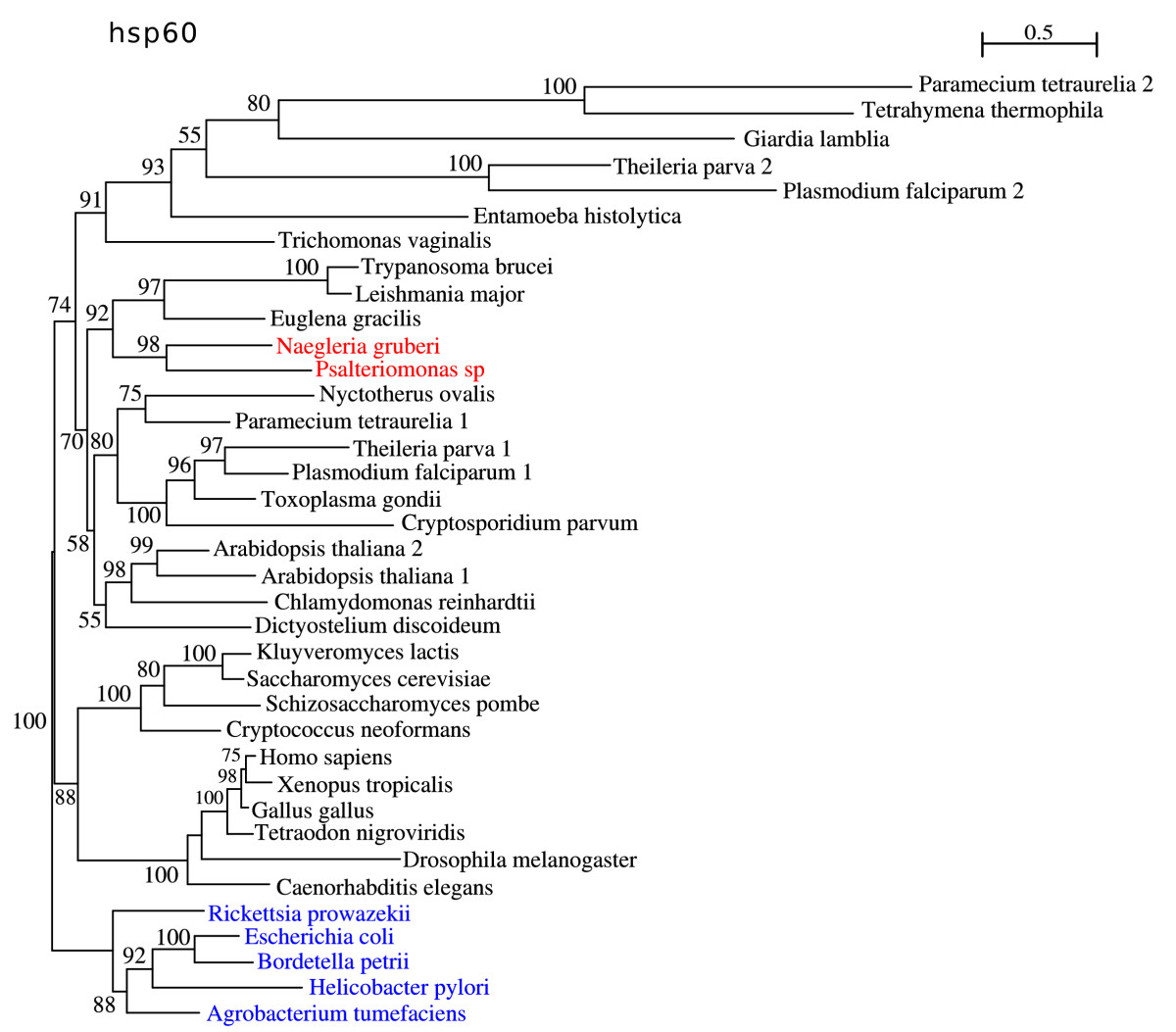

Supplement: Additional file 5 — ZIP files containing several folders, each of which with TreeSnatcher Plus snapshot files, the original image and a text file. [file 1471-2105-13-110-S5.zip › 1471-2148-9-287-5/1471-2148-9-287-5-l.jpg]

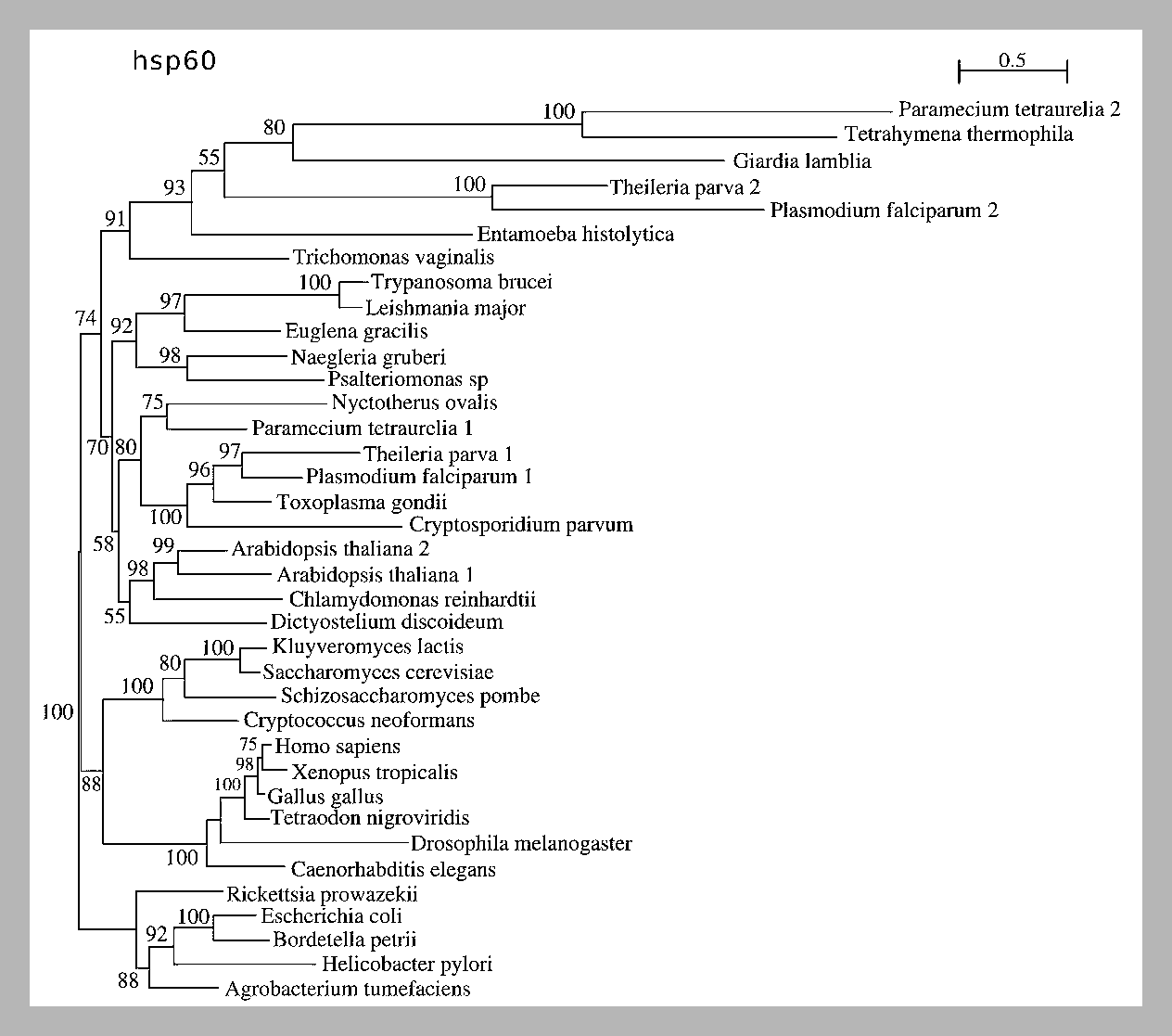

Supplement: Additional file 5 — ZIP files containing several folders, each of which with TreeSnatcher Plus snapshot files, the original image and a text file. [file 1471-2105-13-110-S5.zip › 1471-2148-9-287-5/1471-2148-9-287-5-l_b.PNG]

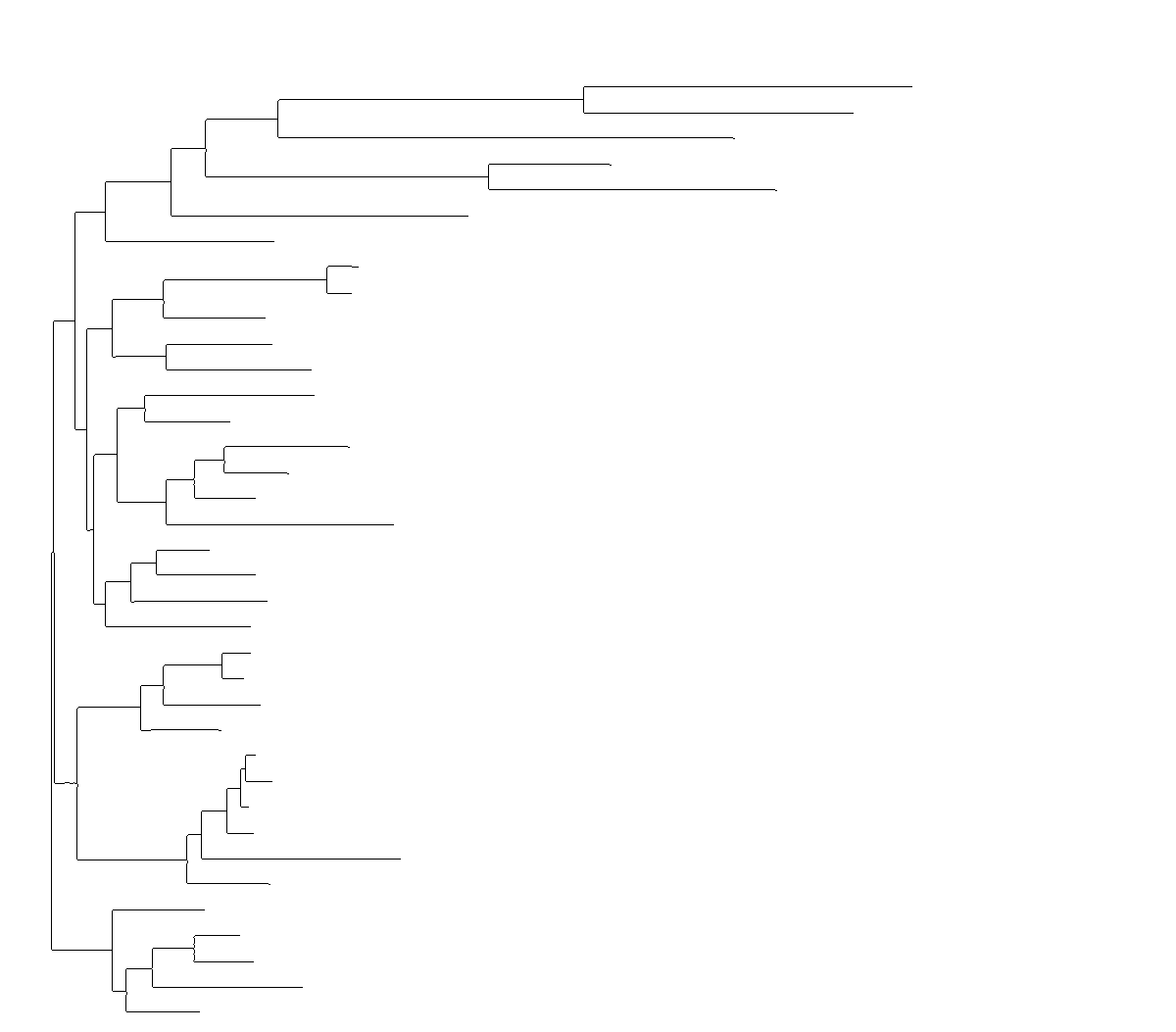

Supplement: Additional file 5 — ZIP files containing several folders, each of which with TreeSnatcher Plus snapshot files, the original image and a text file. [file 1471-2105-13-110-S5.zip › 1471-2148-9-287-5/1471-2148-9-287-5-l_c.PNG]

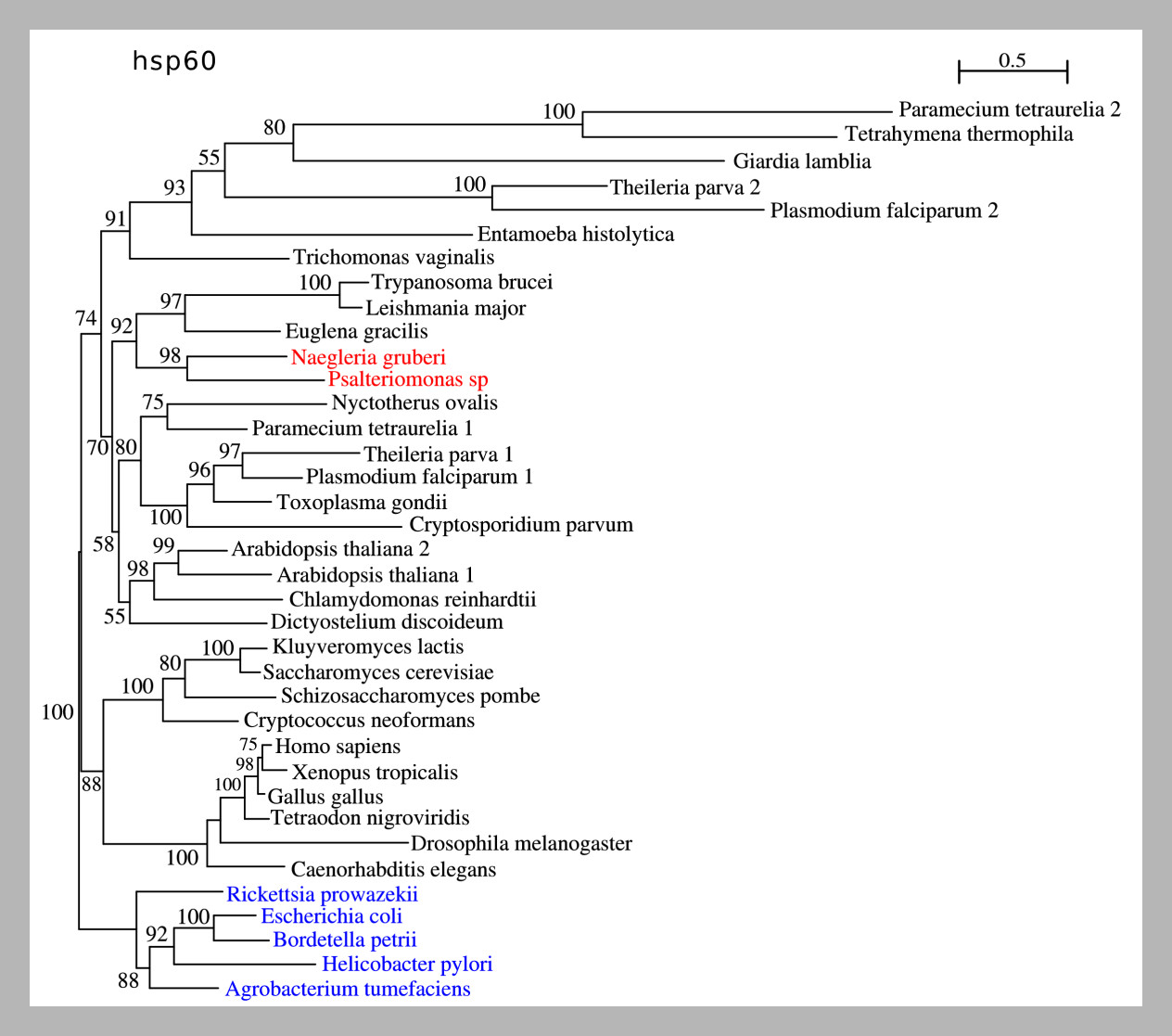

Supplement: Additional file 5 — ZIP files containing several folders, each of which with TreeSnatcher Plus snapshot files, the original image and a text file. [file 1471-2105-13-110-S5.zip › 1471-2148-9-287-5/1471-2148-9-287-5-l_o.PNG]

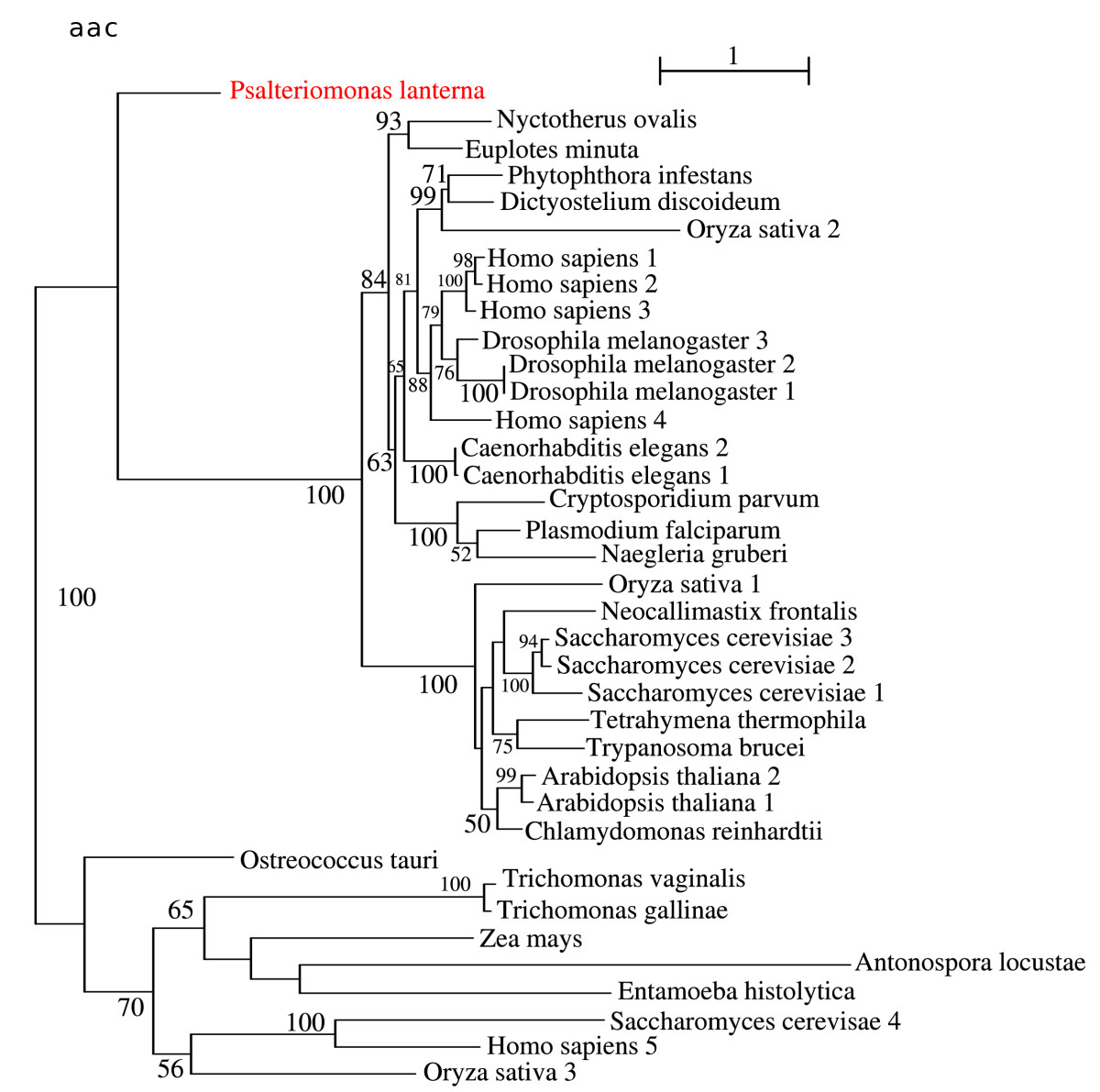

Supplement: Additional file 5 — ZIP files containing several folders, each of which with TreeSnatcher Plus snapshot files, the original image and a text file. [file 1471-2105-13-110-S5.zip › 1471-2148-9-287-6/1471-2148-9-287-6-l.jpg]

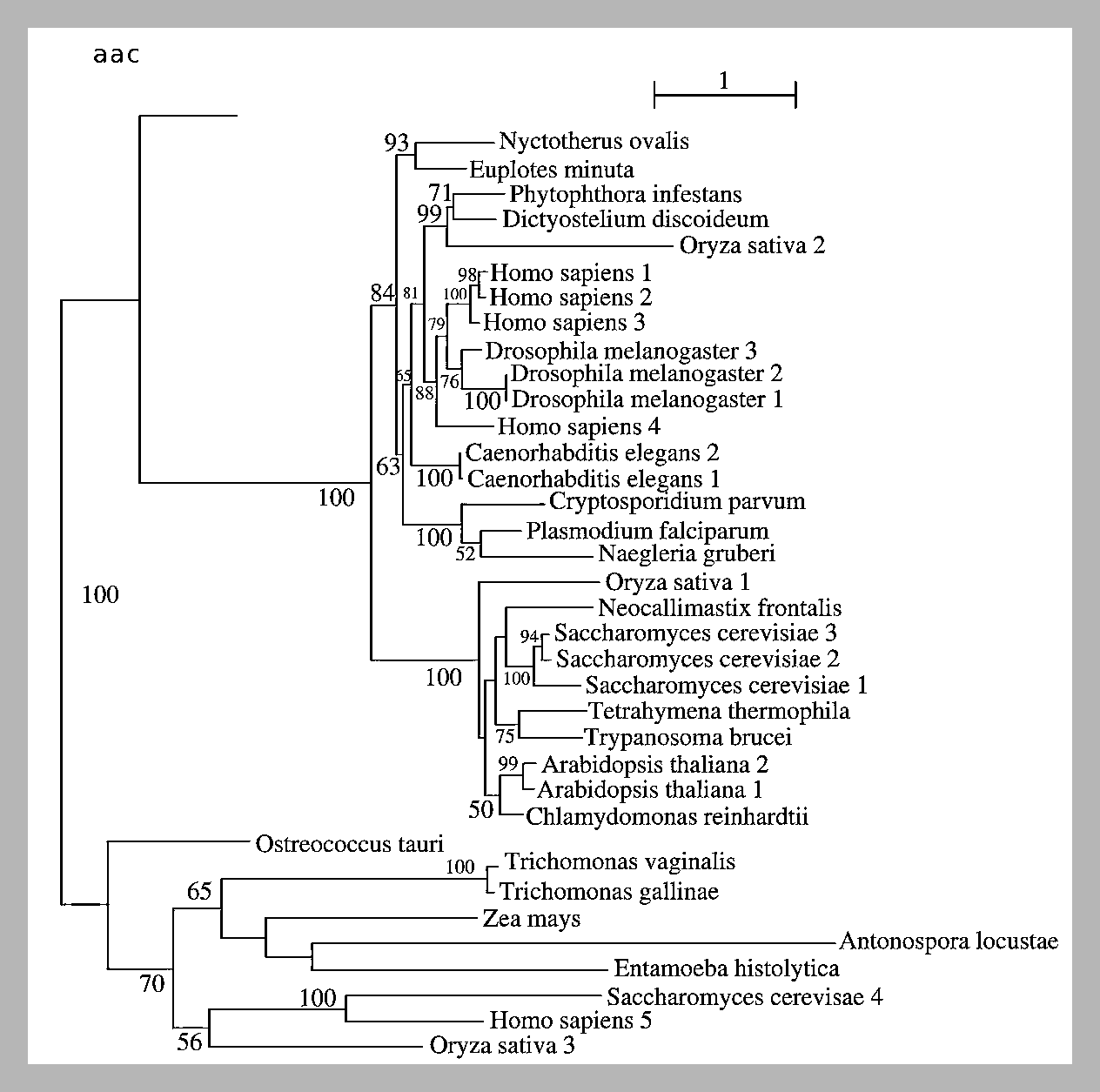

Supplement: Additional file 5 — ZIP files containing several folders, each of which with TreeSnatcher Plus snapshot files, the original image and a text file. [file 1471-2105-13-110-S5.zip › 1471-2148-9-287-6/1471-2148-9-287-6-l_b.PNG]

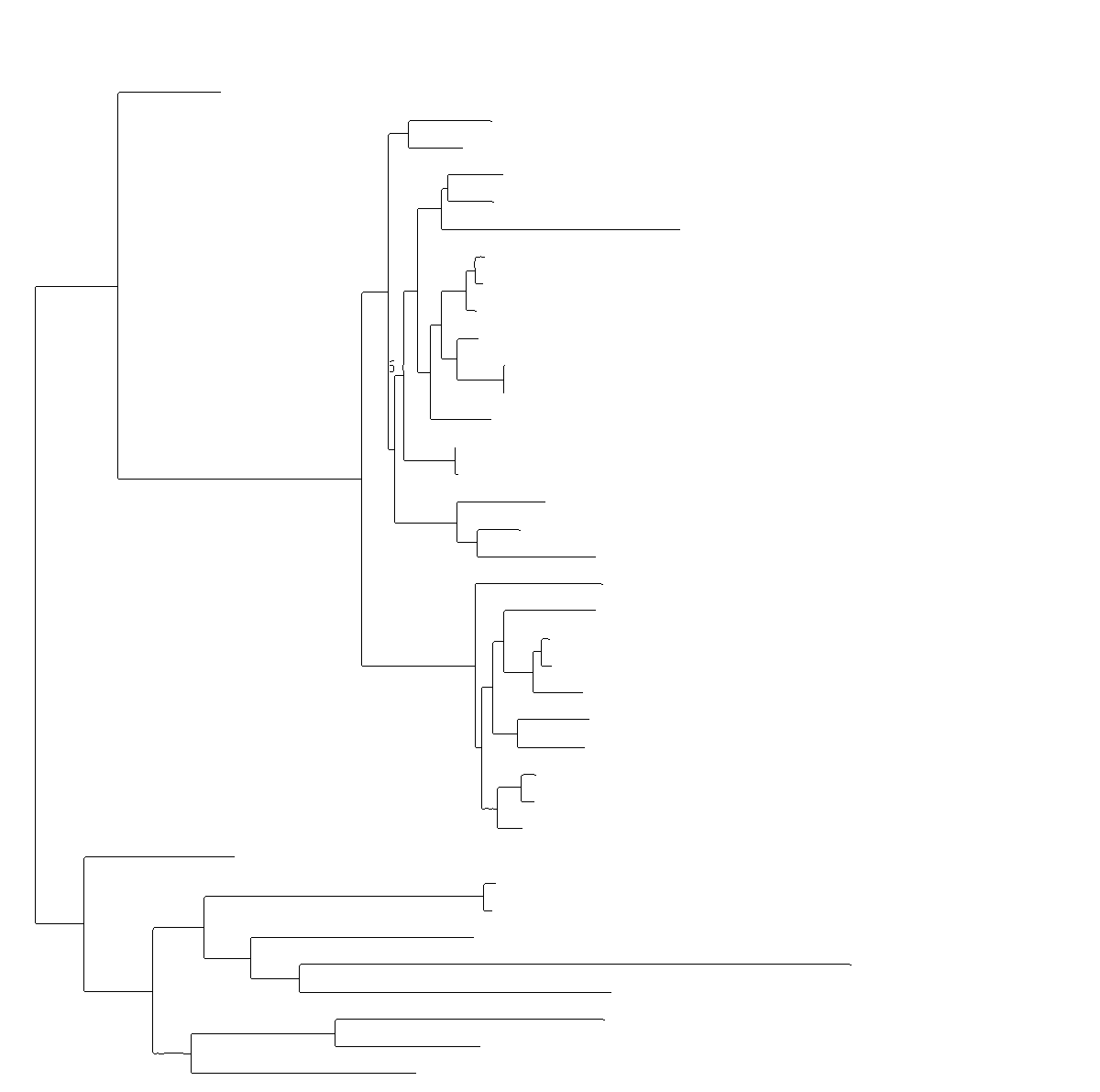

Supplement: Additional file 5 — ZIP files containing several folders, each of which with TreeSnatcher Plus snapshot files, the original image and a text file. [file 1471-2105-13-110-S5.zip › 1471-2148-9-287-6/1471-2148-9-287-6-l_c.PNG]

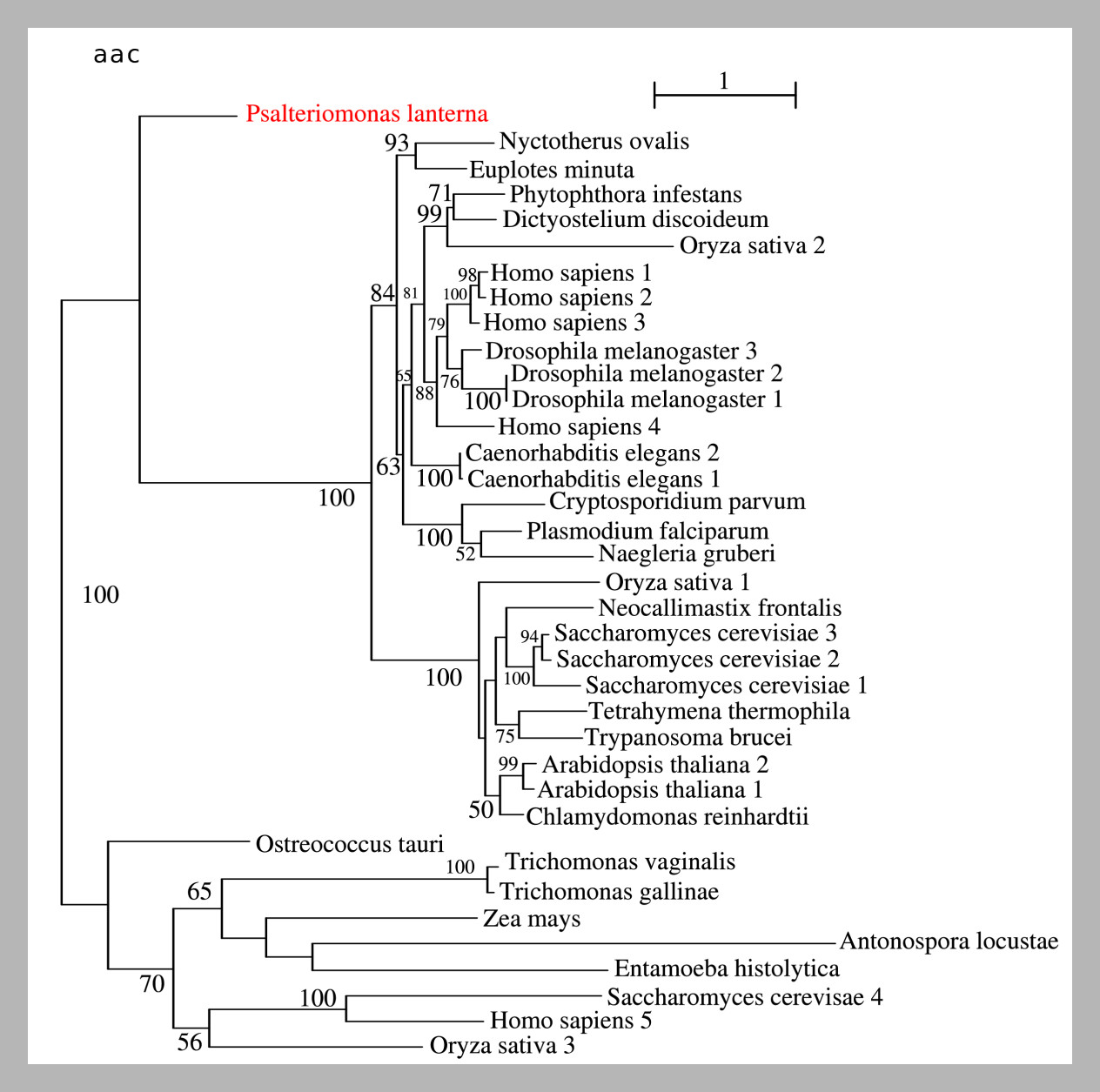

Supplement: Additional file 5 — ZIP files containing several folders, each of which with TreeSnatcher Plus snapshot files, the original image and a text file. [file 1471-2105-13-110-S5.zip › 1471-2148-9-287-6/1471-2148-9-287-6-l_o.PNG]

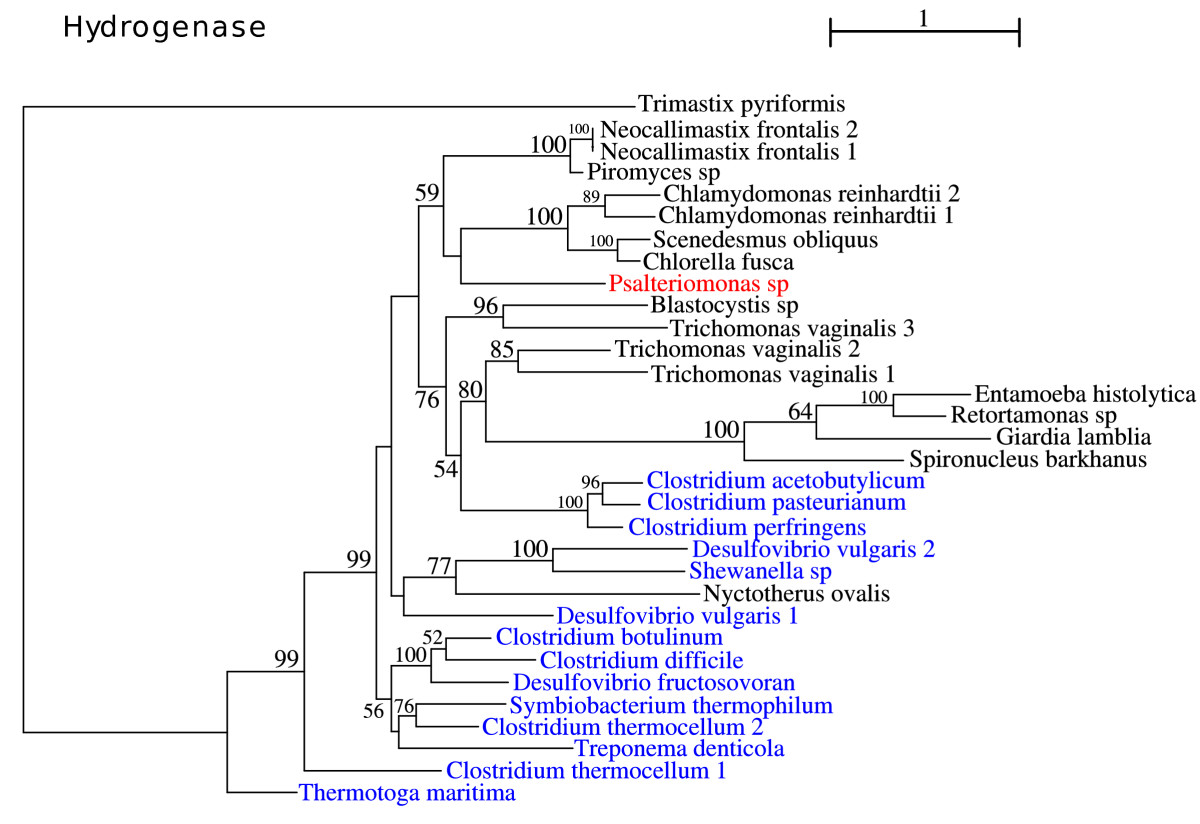

Supplement: Additional file 5 — ZIP files containing several folders, each of which with TreeSnatcher Plus snapshot files, the original image and a text file. [file 1471-2105-13-110-S5.zip › 1471-2148-9-287-7/1471-2148-9-287-7-l.jpg]

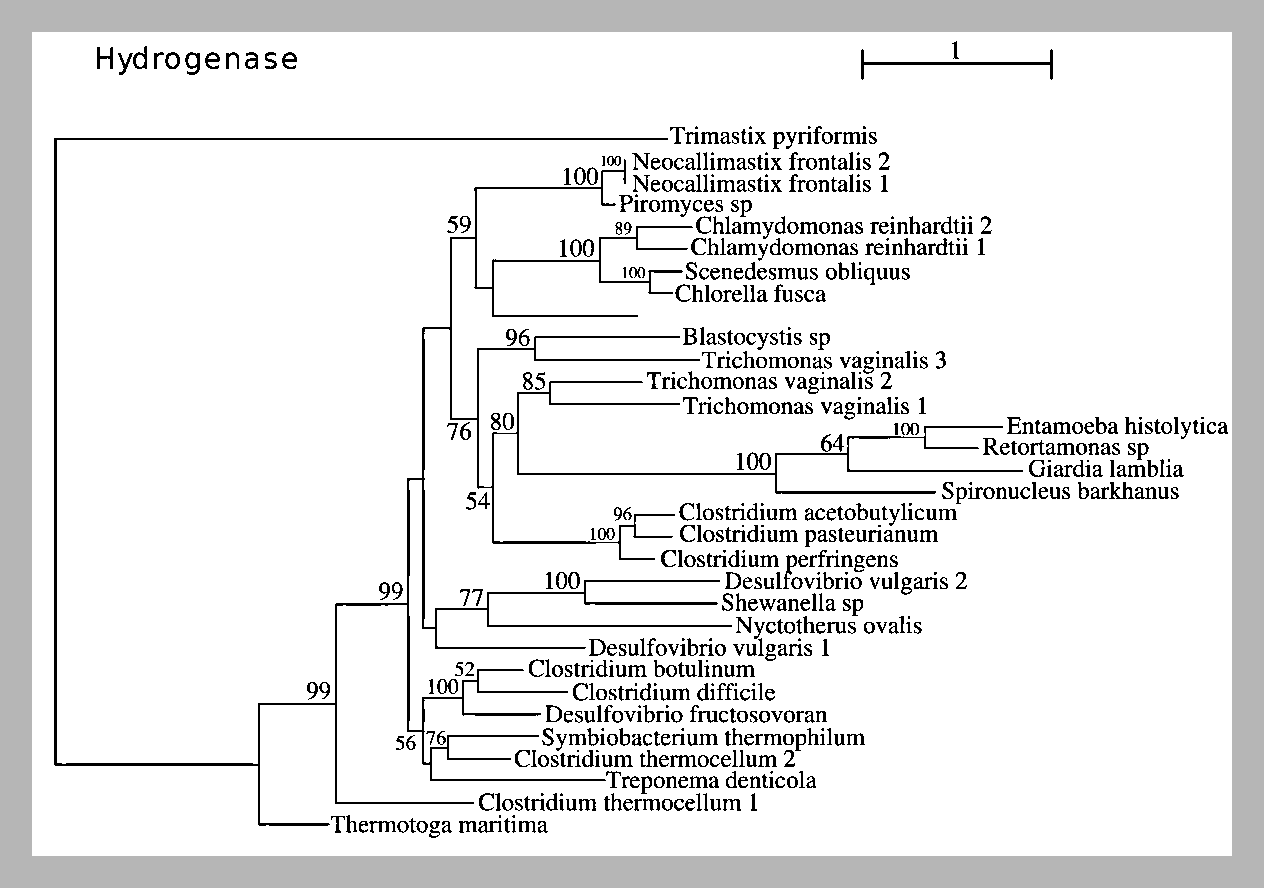

Supplement: Additional file 5 — ZIP files containing several folders, each of which with TreeSnatcher Plus snapshot files, the original image and a text file. [file 1471-2105-13-110-S5.zip › 1471-2148-9-287-7/1471-2148-9-287-7-l_b.PNG]

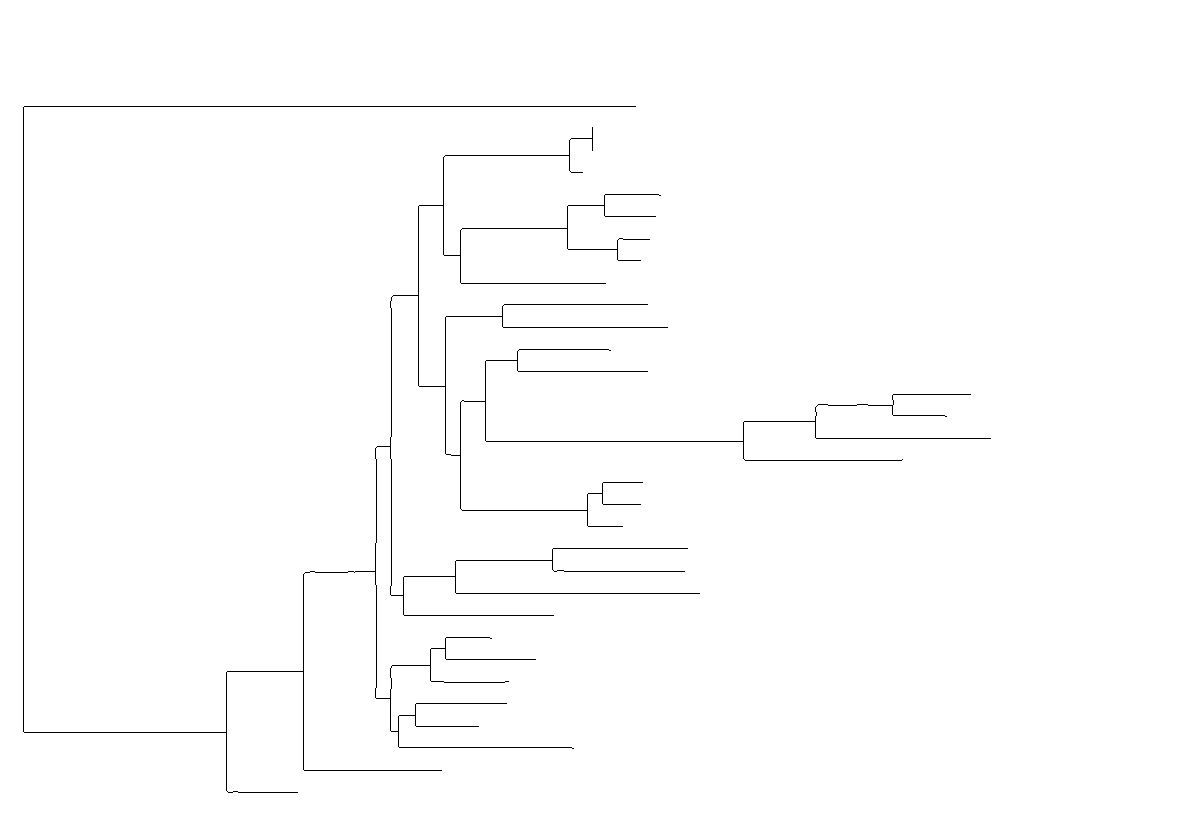

Supplement: Additional file 5 — ZIP files containing several folders, each of which with TreeSnatcher Plus snapshot files, the original image and a text file. [file 1471-2105-13-110-S5.zip › 1471-2148-9-287-7/1471-2148-9-287-7-l_c.PNG]

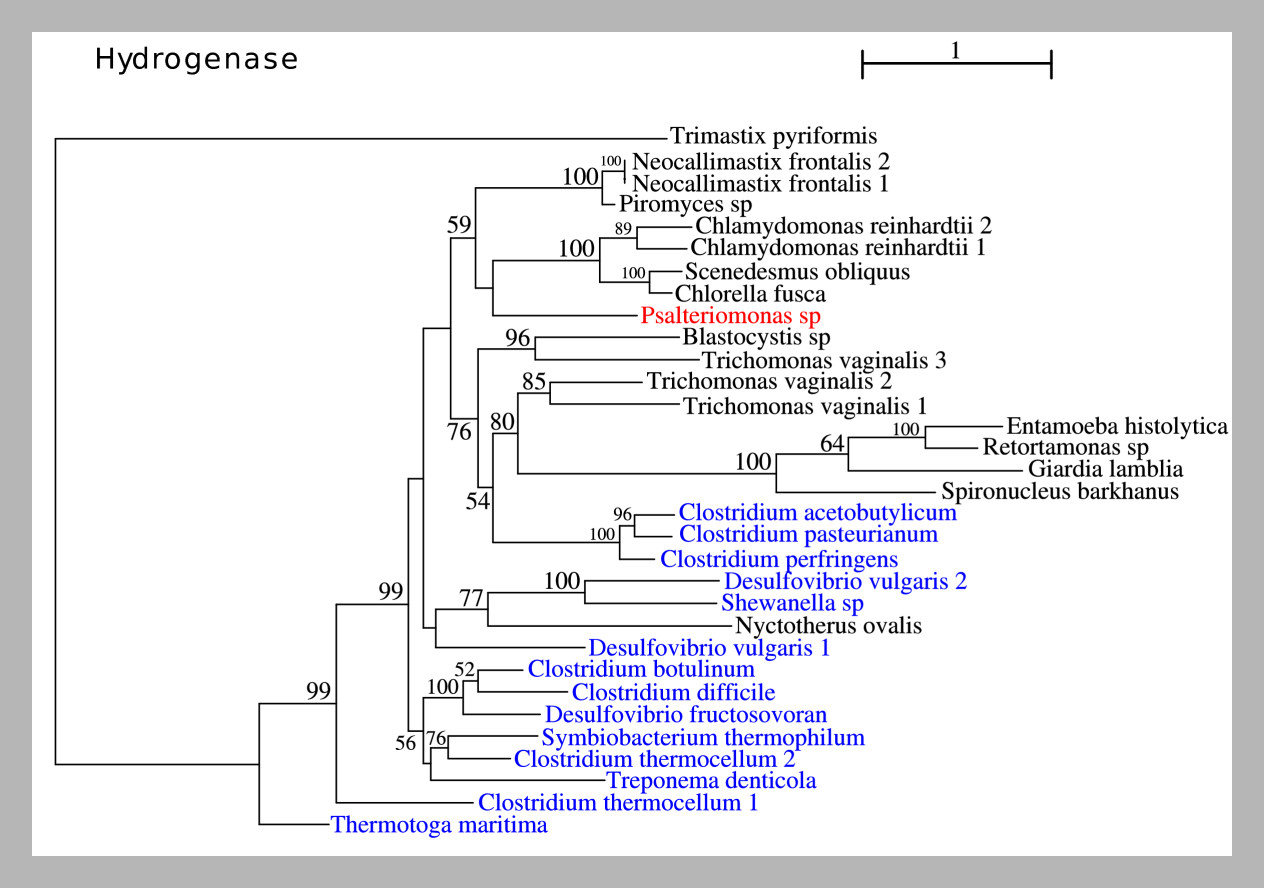

Supplement: Additional file 5 — ZIP files containing several folders, each of which with TreeSnatcher Plus snapshot files, the original image and a text file. [file 1471-2105-13-110-S5.zip › 1471-2148-9-287-7/1471-2148-9-287-7-l_o.PNG]

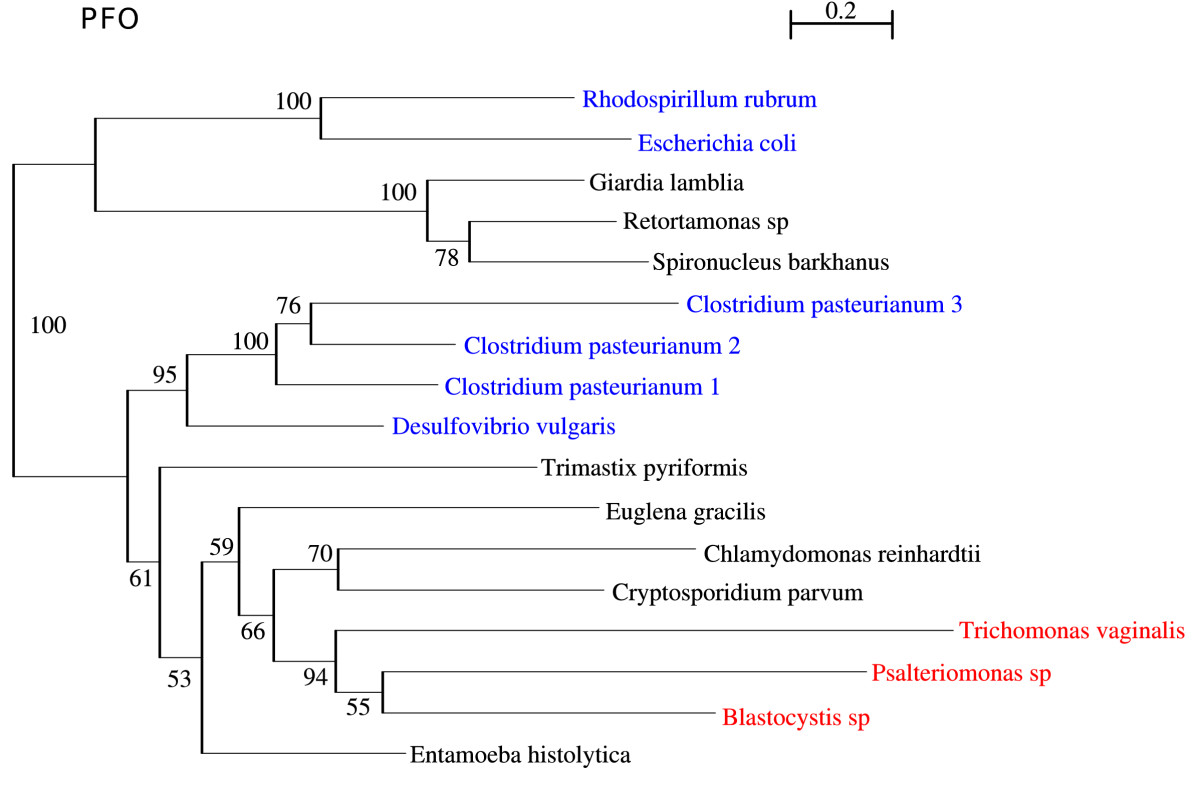

Supplement: Additional file 5 — ZIP files containing several folders, each of which with TreeSnatcher Plus snapshot files, the original image and a text file. [file 1471-2105-13-110-S5.zip › 1471-2148-9-287-8/1471-2148-9-287-8-l.jpg]

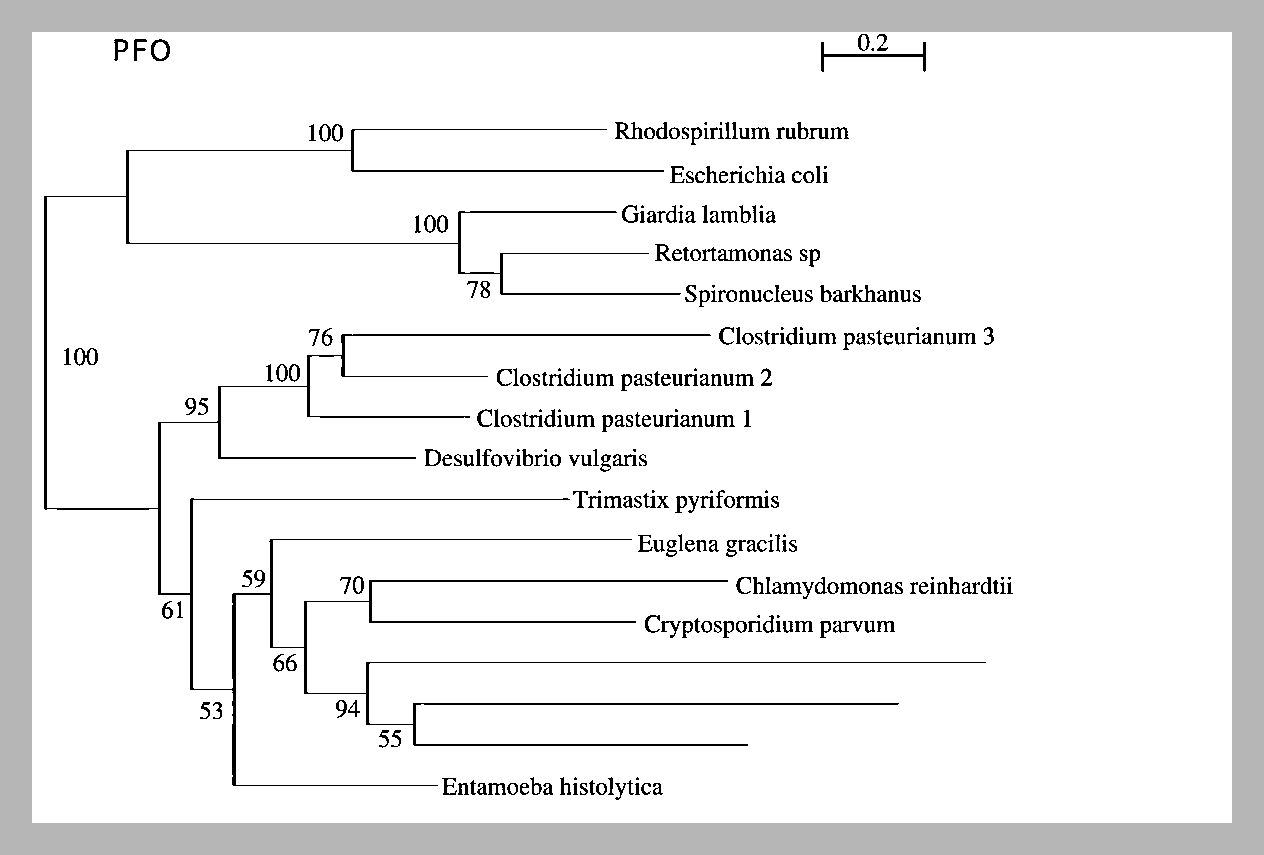

Supplement: Additional file 5 — ZIP files containing several folders, each of which with TreeSnatcher Plus snapshot files, the original image and a text file. [file 1471-2105-13-110-S5.zip › 1471-2148-9-287-8/1471-2148-9-287-8-l_b.PNG]

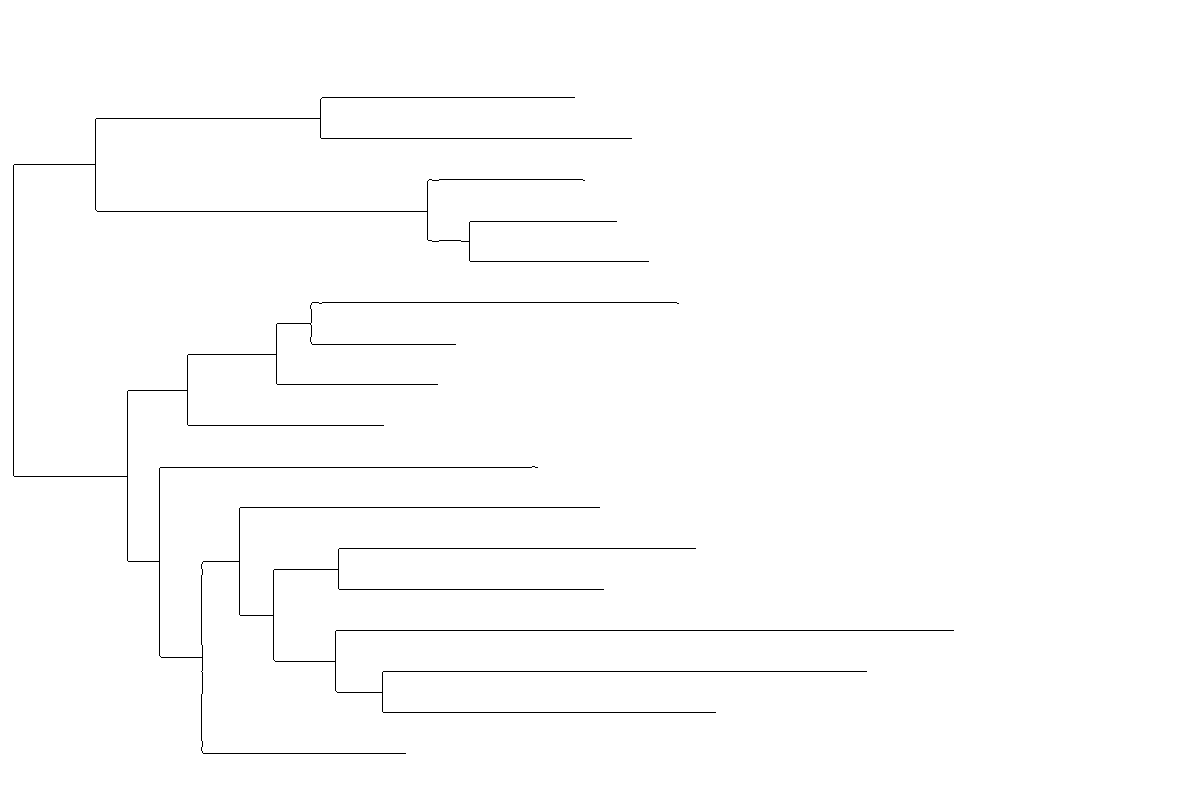

Supplement: Additional file 5 — ZIP files containing several folders, each of which with TreeSnatcher Plus snapshot files, the original image and a text file. [file 1471-2105-13-110-S5.zip › 1471-2148-9-287-8/1471-2148-9-287-8-l_c.PNG]

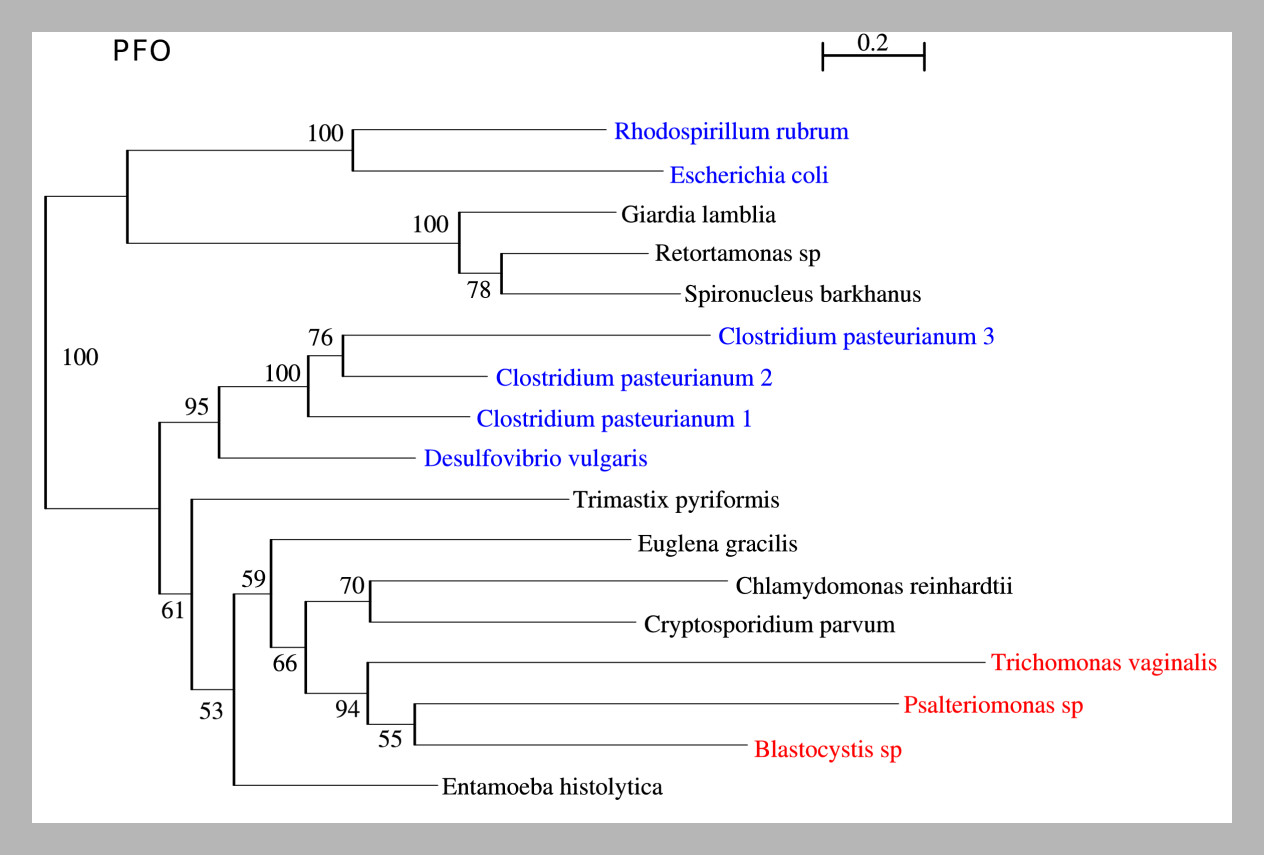

Supplement: Additional file 5 — ZIP files containing several folders, each of which with TreeSnatcher Plus snapshot files, the original image and a text file. [file 1471-2105-13-110-S5.zip › 1471-2148-9-287-8/1471-2148-9-287-8-l_o.PNG]

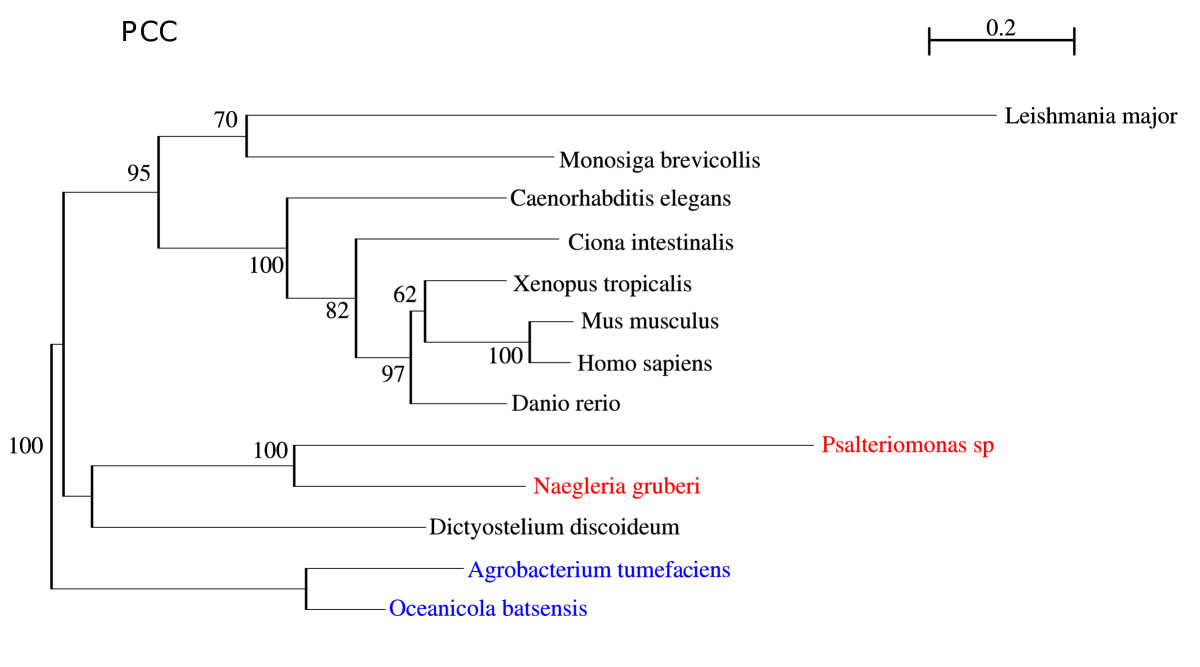

Supplement: Additional file 5 — ZIP files containing several folders, each of which with TreeSnatcher Plus snapshot files, the original image and a text file. [file 1471-2105-13-110-S5.zip › 1471-2148-9-287-9/1471-2148-9-287-9-l.jpg]

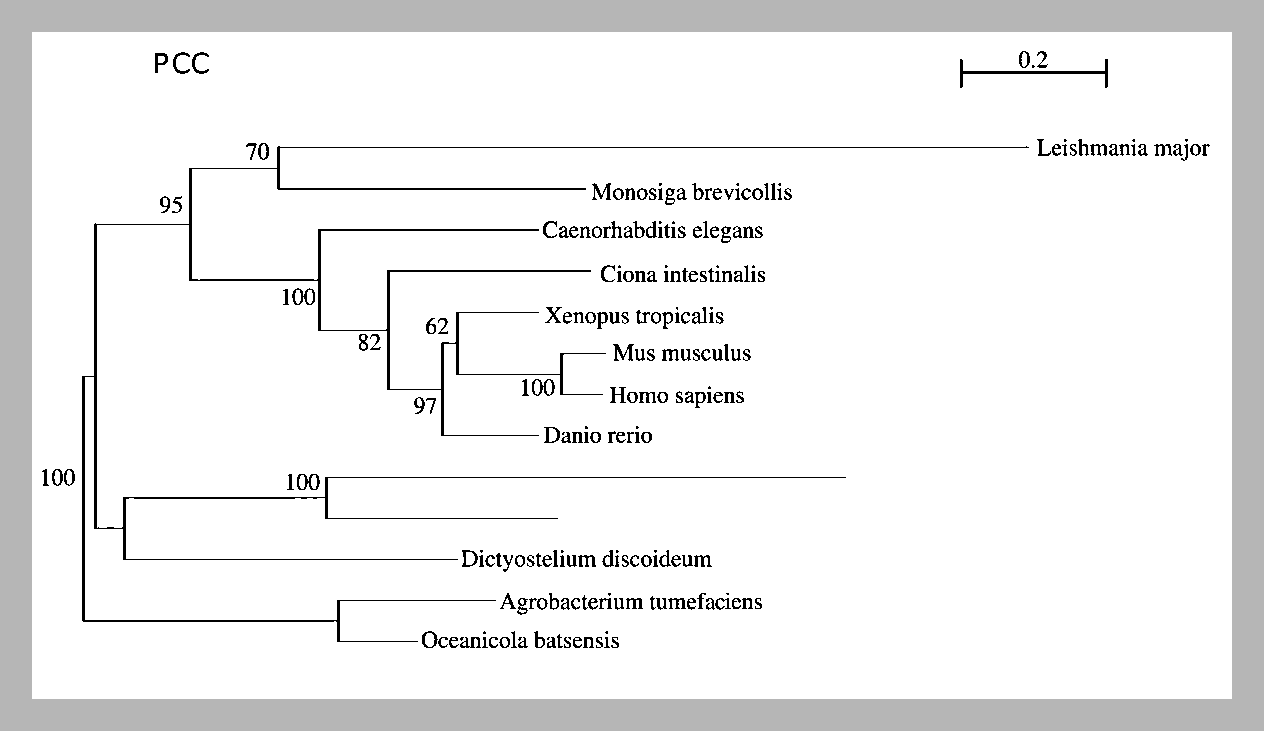

Supplement: Additional file 5 — ZIP files containing several folders, each of which with TreeSnatcher Plus snapshot files, the original image and a text file. [file 1471-2105-13-110-S5.zip › 1471-2148-9-287-9/1471-2148-9-287-9-l_b.PNG]

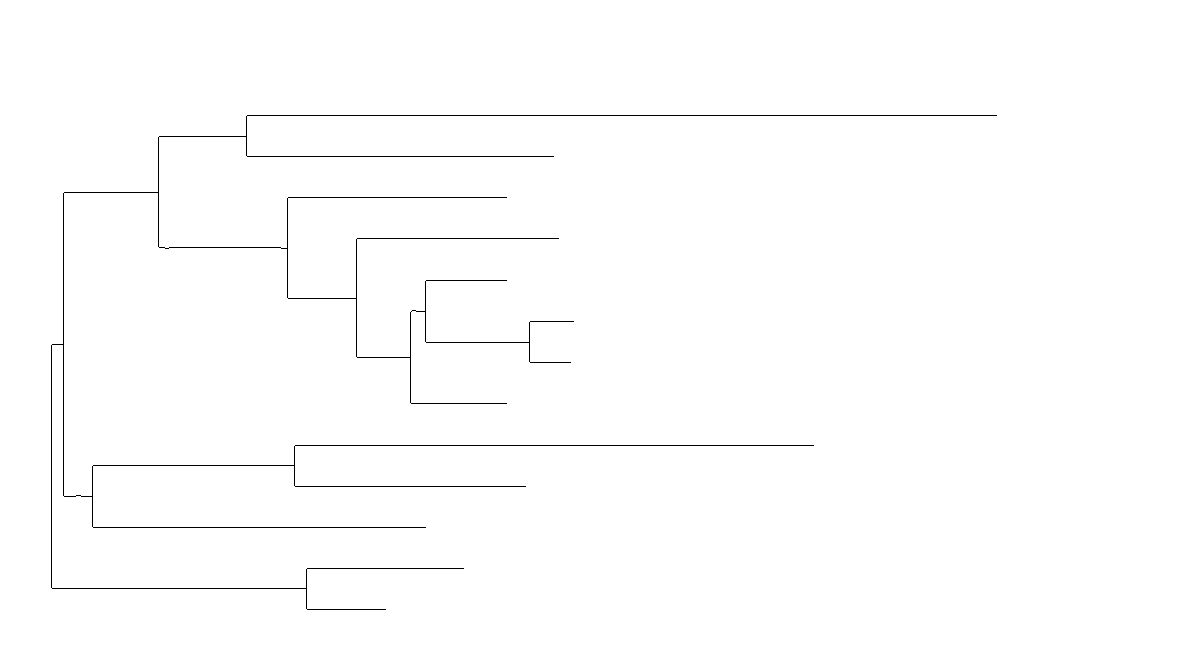

Supplement: Additional file 5 — ZIP files containing several folders, each of which with TreeSnatcher Plus snapshot files, the original image and a text file. [file 1471-2105-13-110-S5.zip › 1471-2148-9-287-9/1471-2148-9-287-9-l_c.PNG]

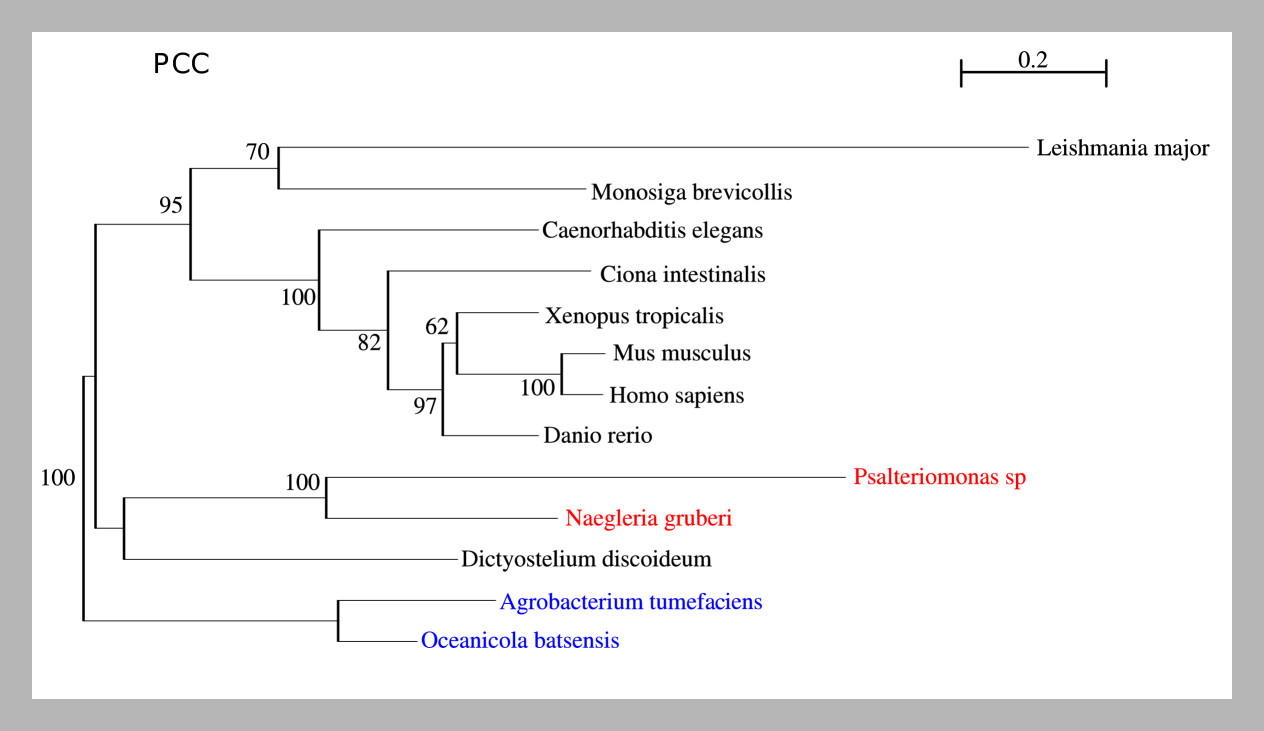

Supplement: Additional file 5 — ZIP files containing several folders, each of which with TreeSnatcher Plus snapshot files, the original image and a text file. [file 1471-2105-13-110-S5.zip › 1471-2148-9-287-9/1471-2148-9-287-9-l_o.PNG]

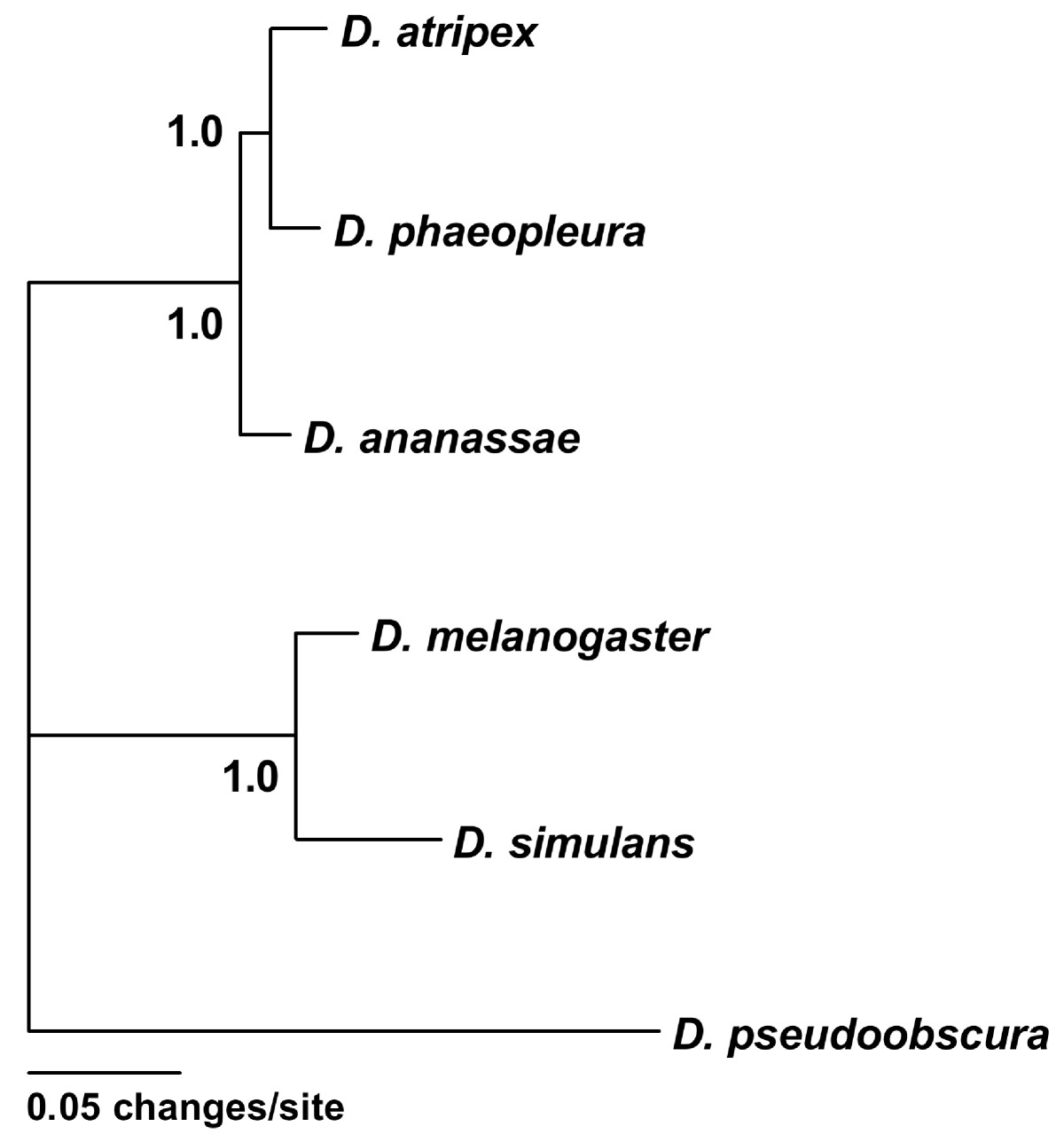

Supplement: Additional file 5 — ZIP files containing several folders, each of which with TreeSnatcher Plus snapshot files, the original image and a text file. [file 1471-2105-13-110-S5.zip › 1471-2148-9-291-2/1471-2148-9-291-2-l.jpg]

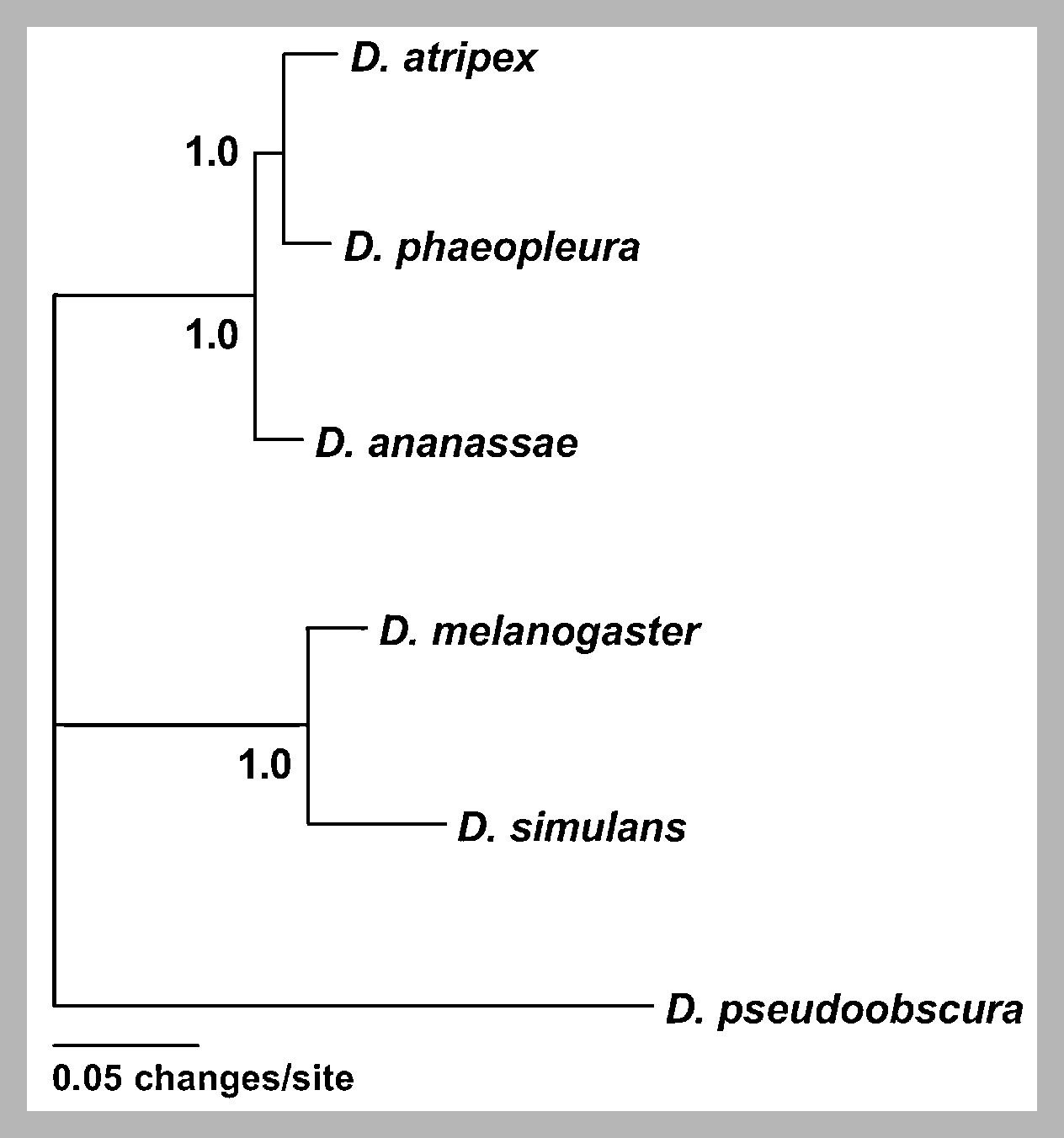

Supplement: Additional file 5 — ZIP files containing several folders, each of which with TreeSnatcher Plus snapshot files, the original image and a text file. [file 1471-2105-13-110-S5.zip › 1471-2148-9-291-2/1471-2148-9-291-2-l_b.PNG]

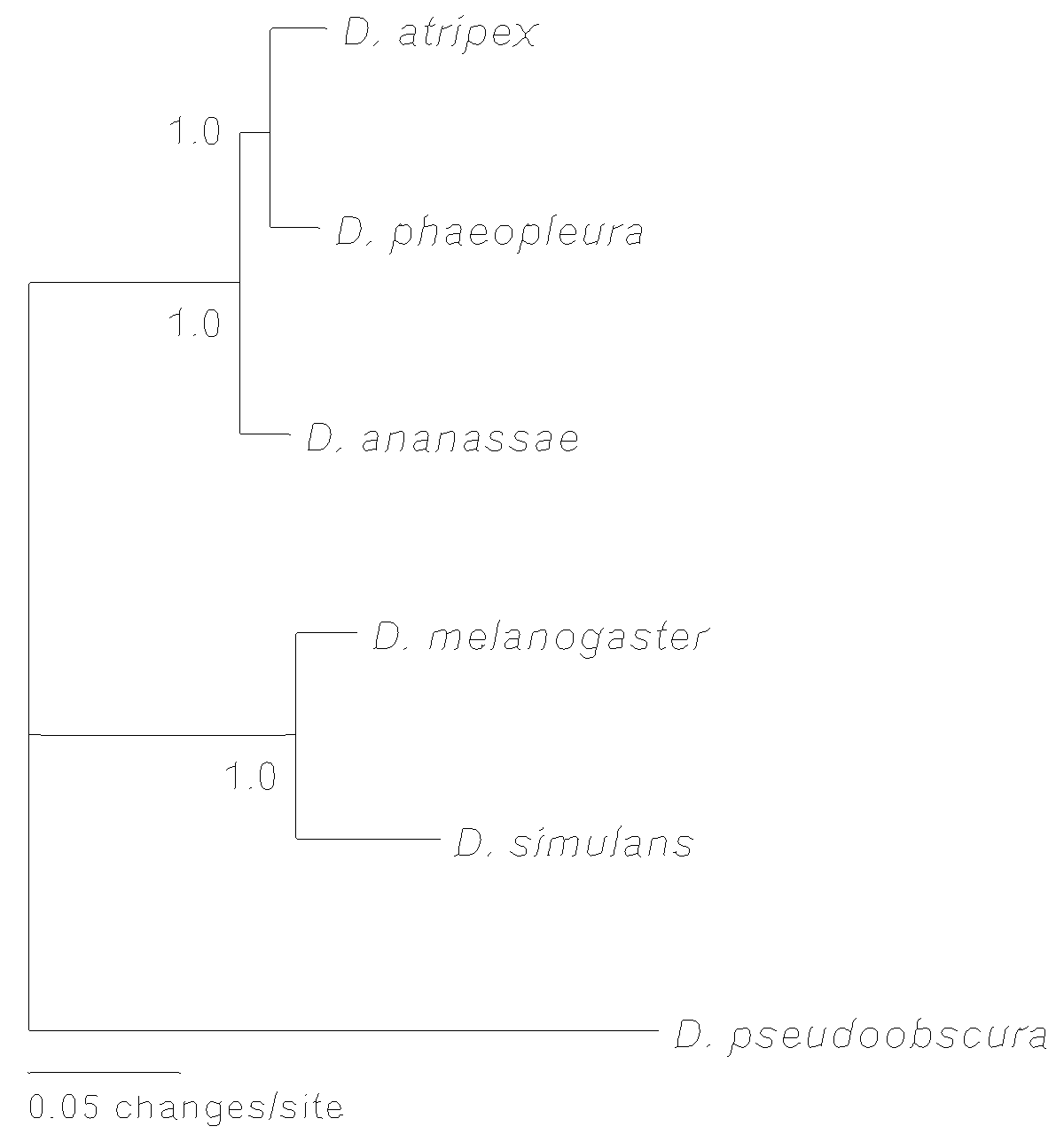

Supplement: Additional file 5 — ZIP files containing several folders, each of which with TreeSnatcher Plus snapshot files, the original image and a text file. [file 1471-2105-13-110-S5.zip › 1471-2148-9-291-2/1471-2148-9-291-2-l_c.PNG]

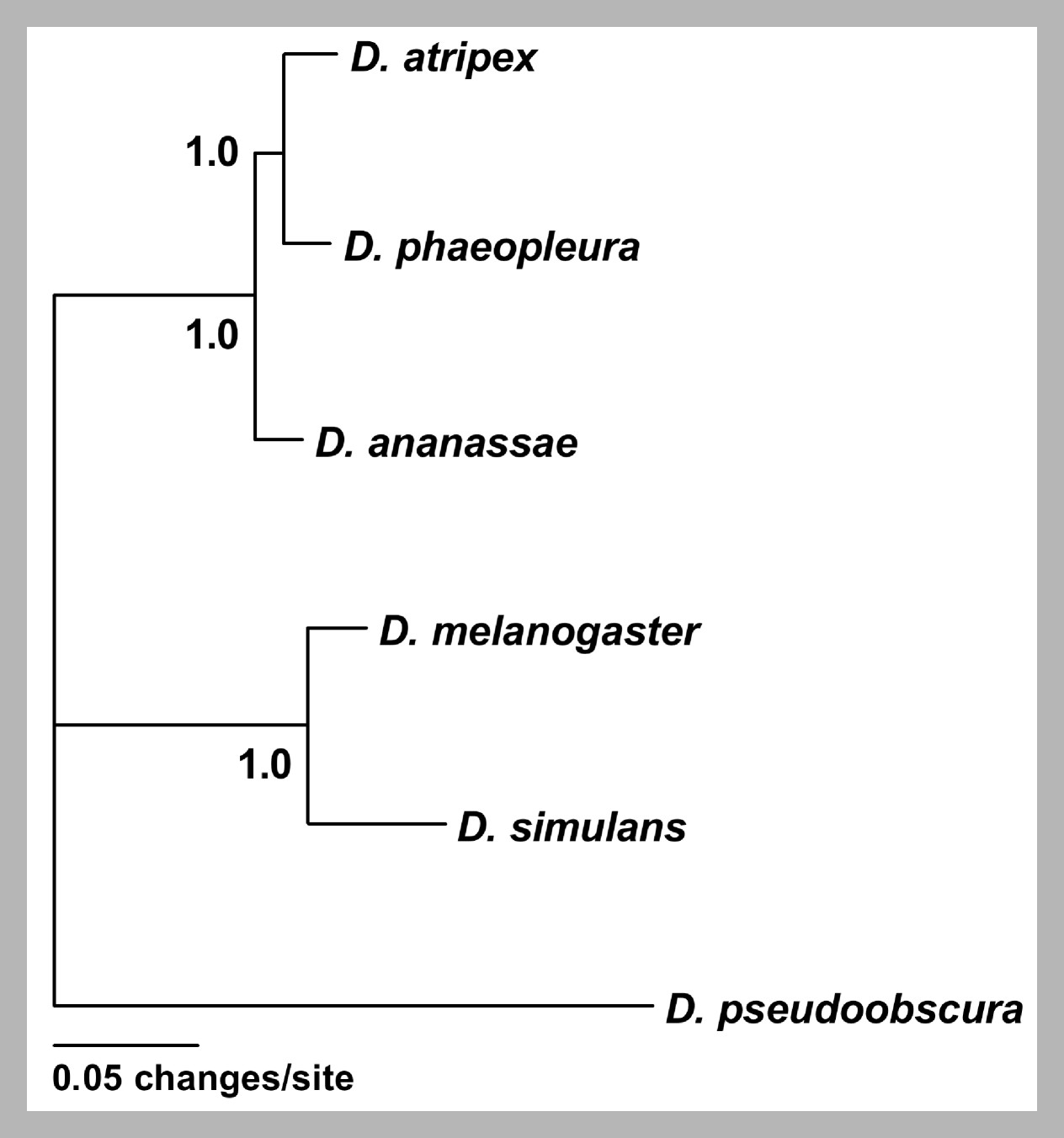

Supplement: Additional file 5 — ZIP files containing several folders, each of which with TreeSnatcher Plus snapshot files, the original image and a text file. [file 1471-2105-13-110-S5.zip › 1471-2148-9-291-2/1471-2148-9-291-2-l_o.PNG]

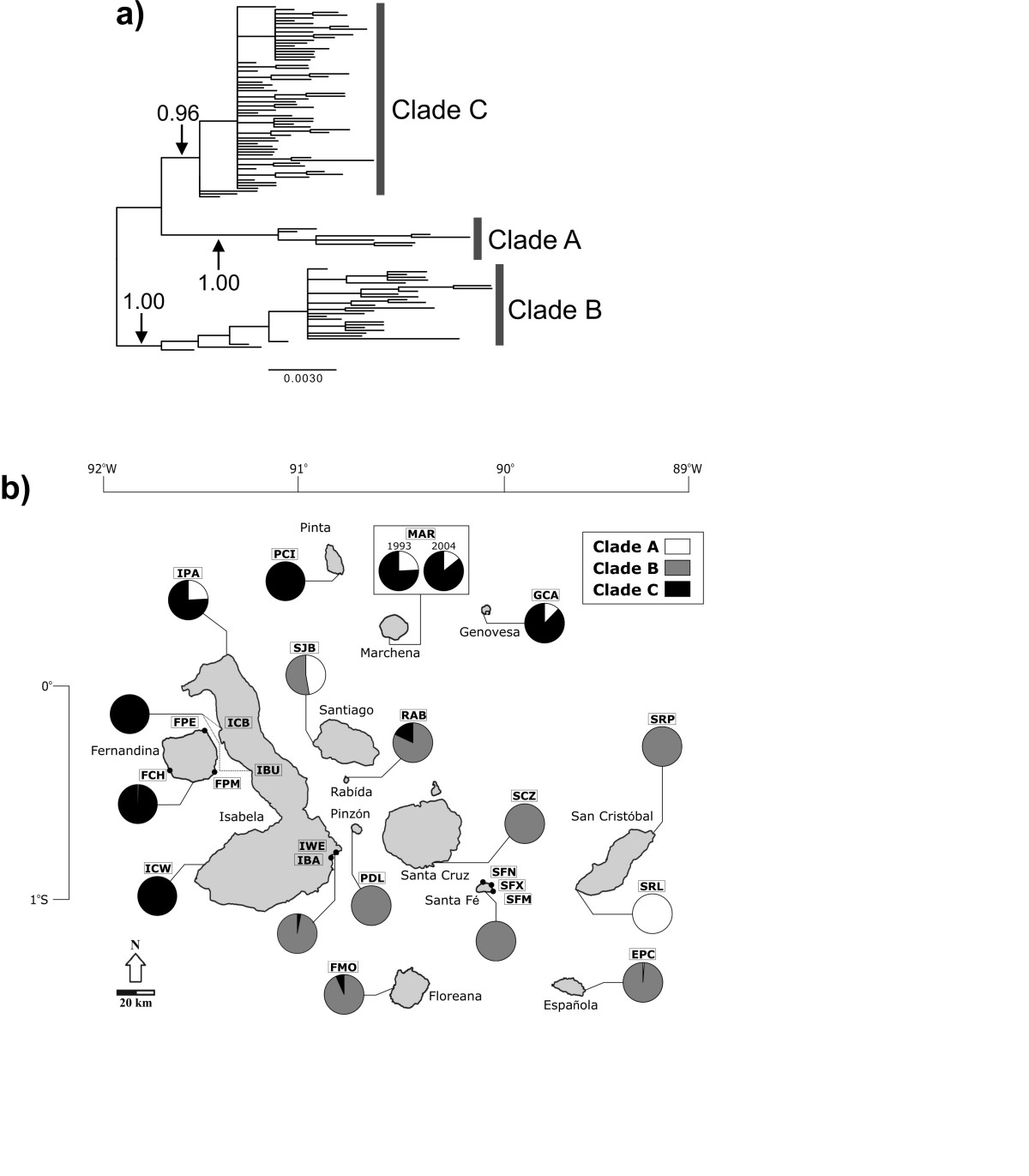

Supplement: Additional file 5 — ZIP files containing several folders, each of which with TreeSnatcher Plus snapshot files, the original image and a text file. [file 1471-2105-13-110-S5.zip › 1471-2148-9-297-1/1471-2148-9-297-1-l.jpg]

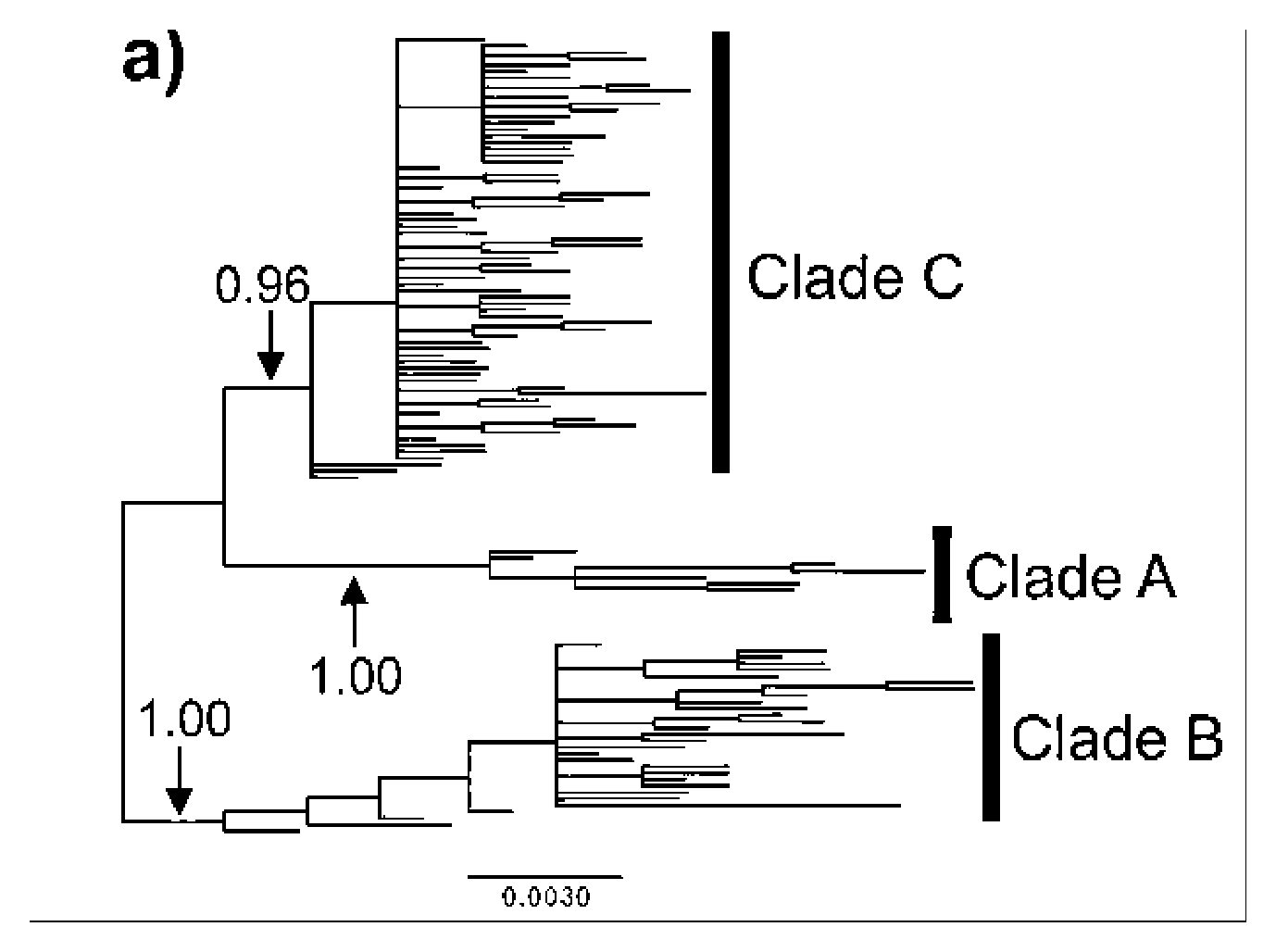

Supplement: Additional file 5 — ZIP files containing several folders, each of which with TreeSnatcher Plus snapshot files, the original image and a text file. [file 1471-2105-13-110-S5.zip › 1471-2148-9-297-1/1471-2148-9-297-1-l_b.PNG]

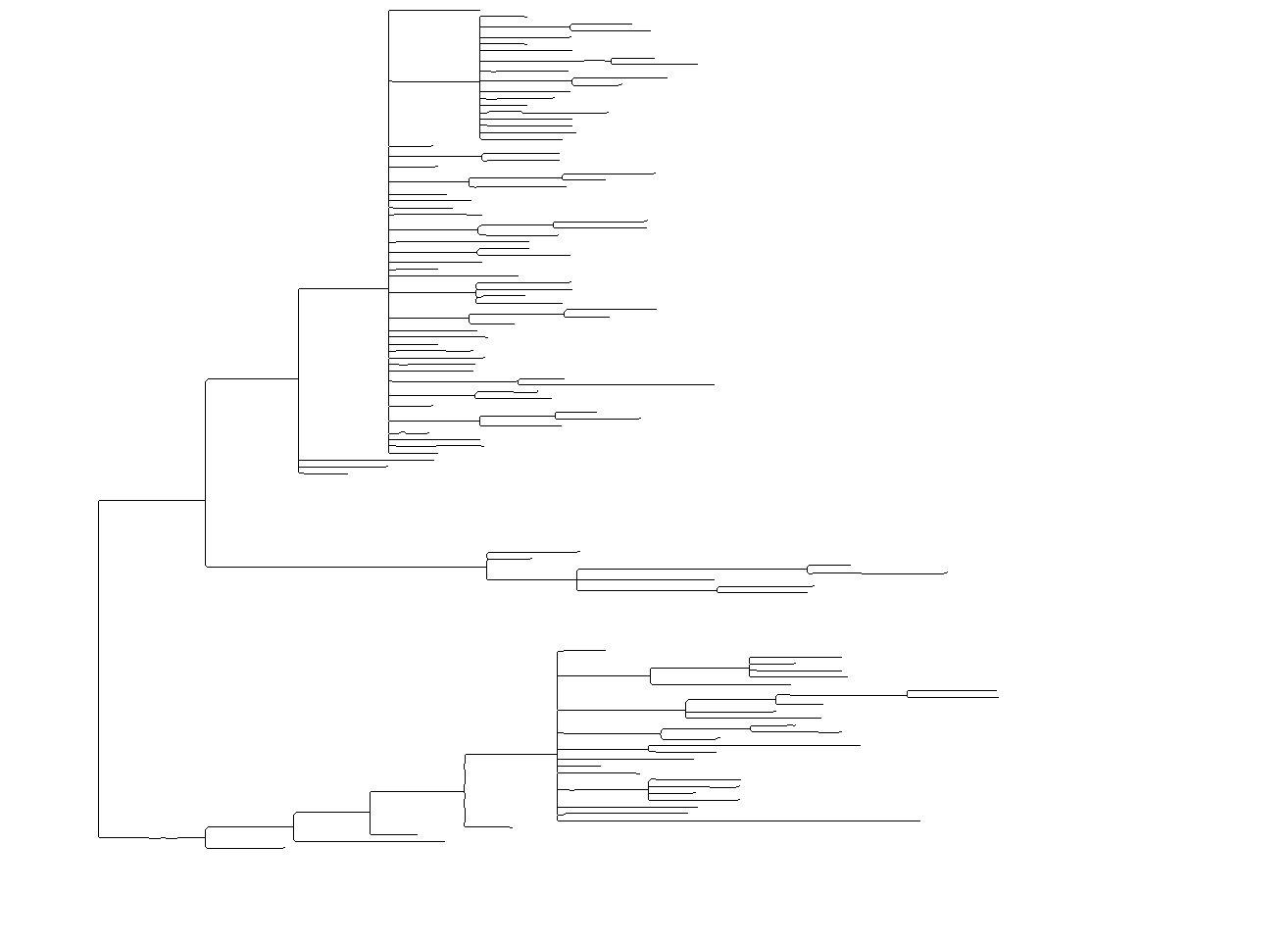

Supplement: Additional file 5 — ZIP files containing several folders, each of which with TreeSnatcher Plus snapshot files, the original image and a text file. [file 1471-2105-13-110-S5.zip › 1471-2148-9-297-1/1471-2148-9-297-1-l_c.PNG]

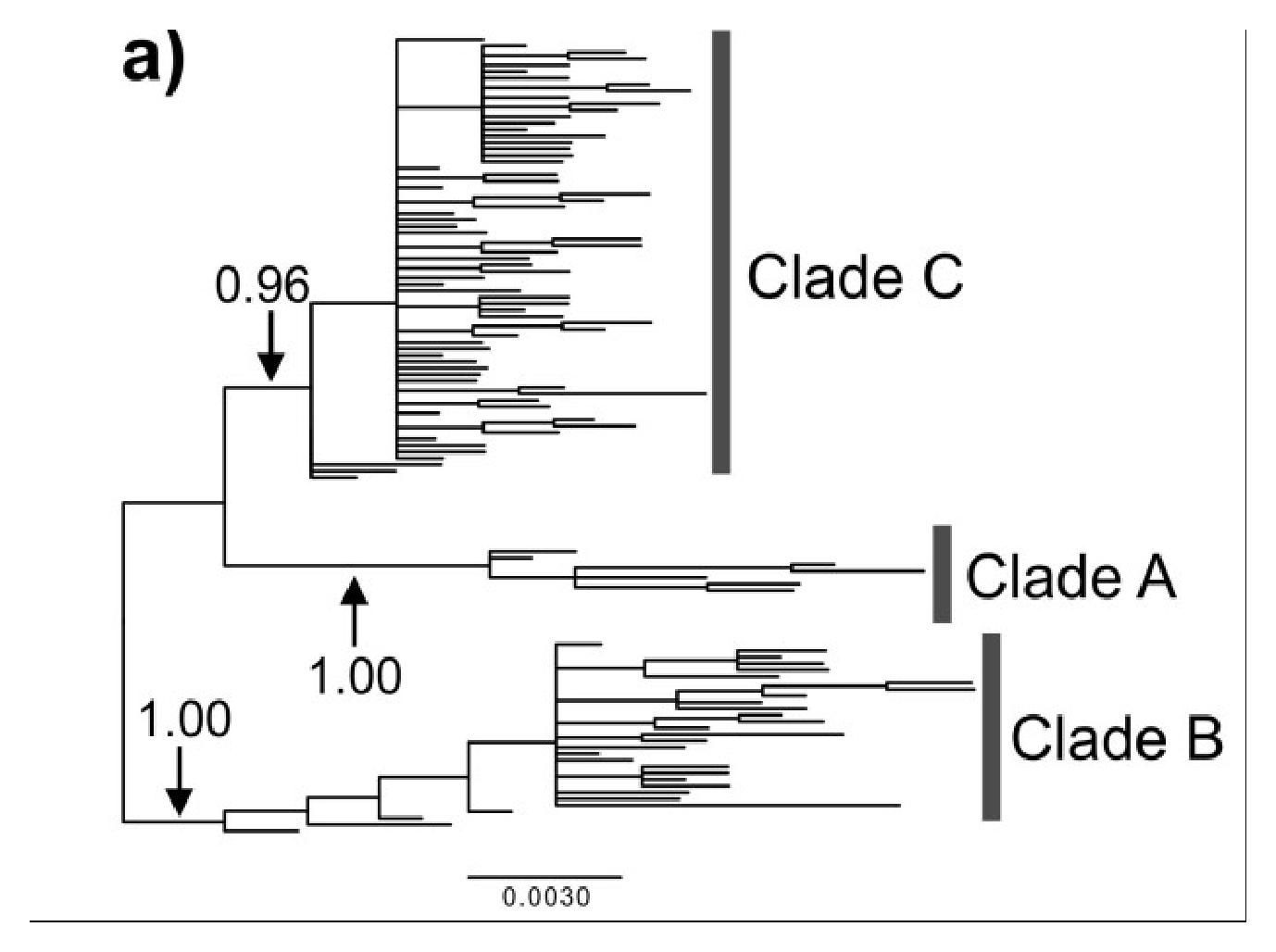

Supplement: Additional file 5 — ZIP files containing several folders, each of which with TreeSnatcher Plus snapshot files, the original image and a text file. [file 1471-2105-13-110-S5.zip › 1471-2148-9-297-1/1471-2148-9-297-1-l_o.PNG]

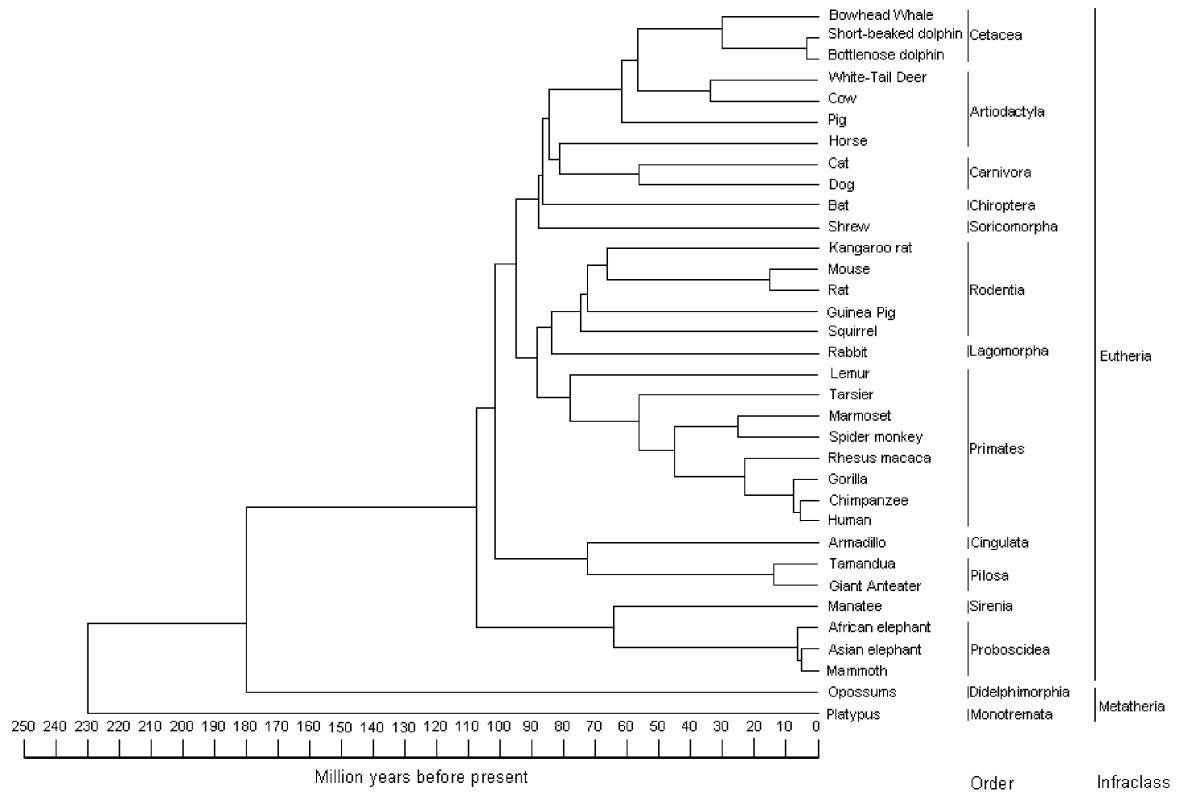

Supplement: Additional file 5 — ZIP files containing several folders, each of which with TreeSnatcher Plus snapshot files, the original image and a text file. [file 1471-2105-13-110-S5.zip › 1471-2148-9-299-1/1471-2148-9-299-1-l.jpg]

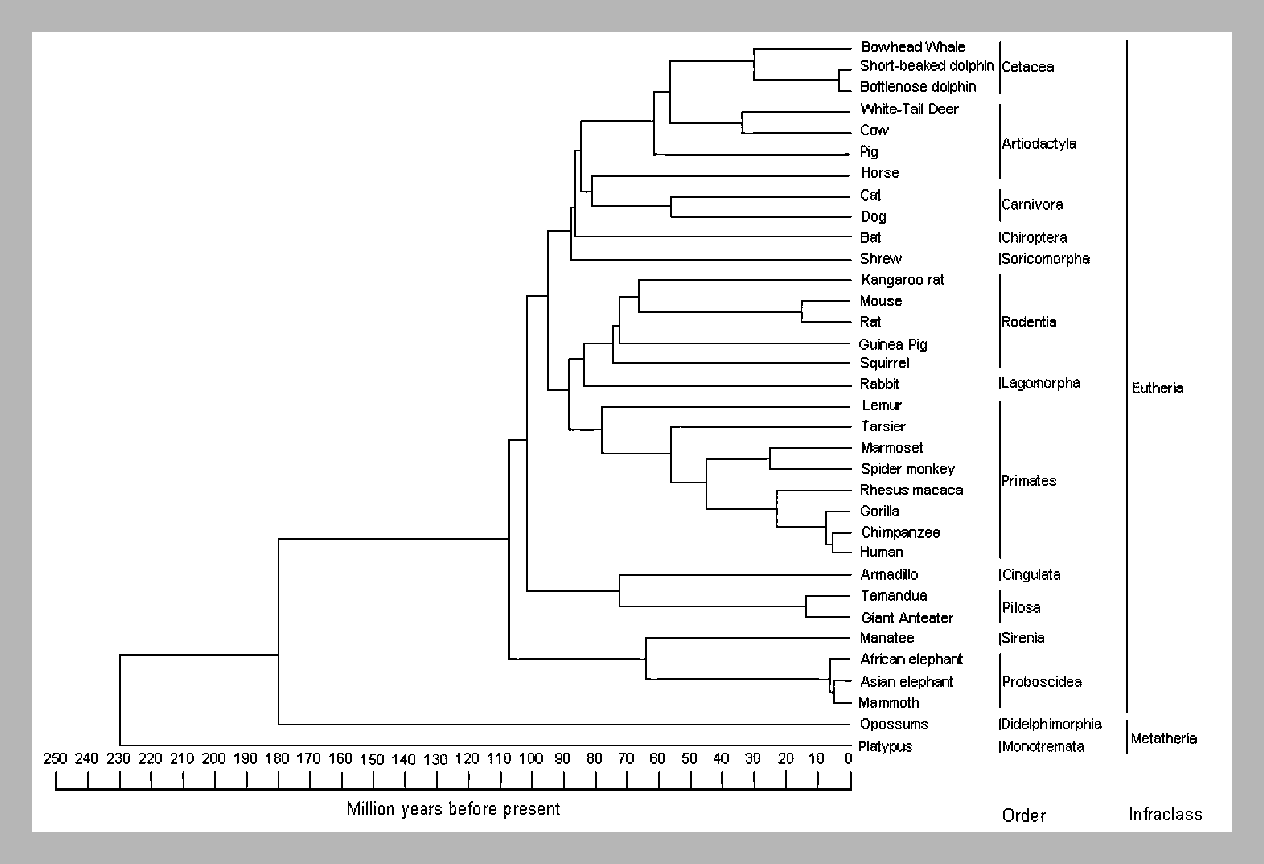

Supplement: Additional file 5 — ZIP files containing several folders, each of which with TreeSnatcher Plus snapshot files, the original image and a text file. [file 1471-2105-13-110-S5.zip › 1471-2148-9-299-1/1471-2148-9-299-1-l_b.PNG]

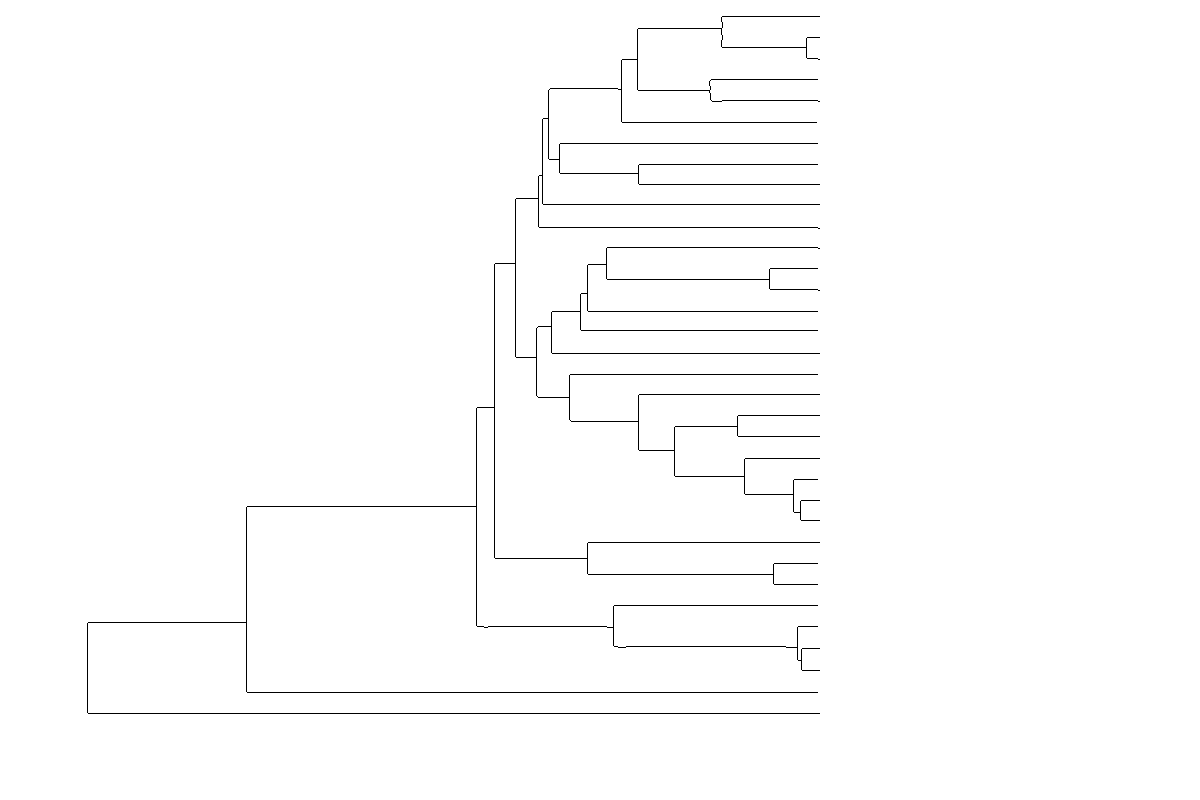

Supplement: Additional file 5 — ZIP files containing several folders, each of which with TreeSnatcher Plus snapshot files, the original image and a text file. [file 1471-2105-13-110-S5.zip › 1471-2148-9-299-1/1471-2148-9-299-1-l_c.PNG]

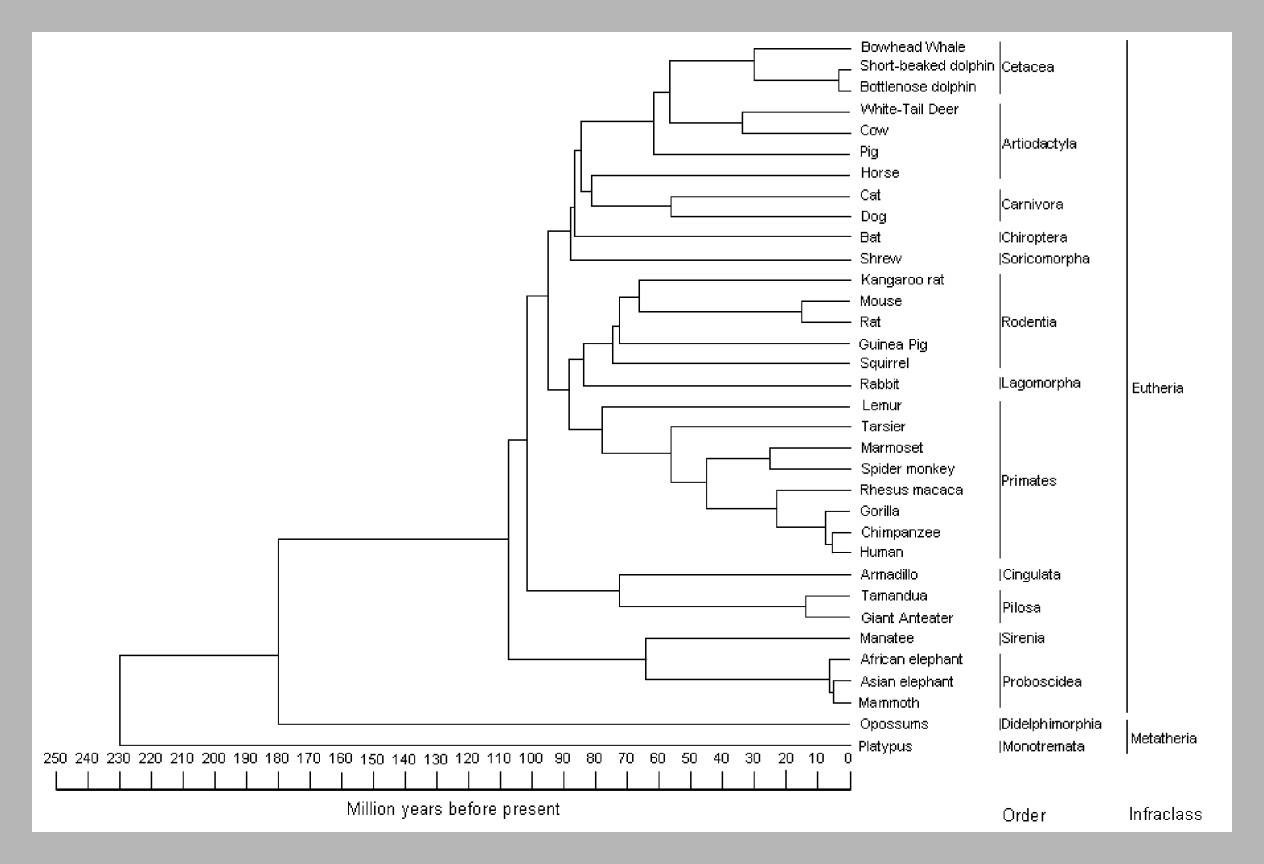

Supplement: Additional file 5 — ZIP files containing several folders, each of which with TreeSnatcher Plus snapshot files, the original image and a text file. [file 1471-2105-13-110-S5.zip › 1471-2148-9-299-1/1471-2148-9-299-1-l_o.PNG]

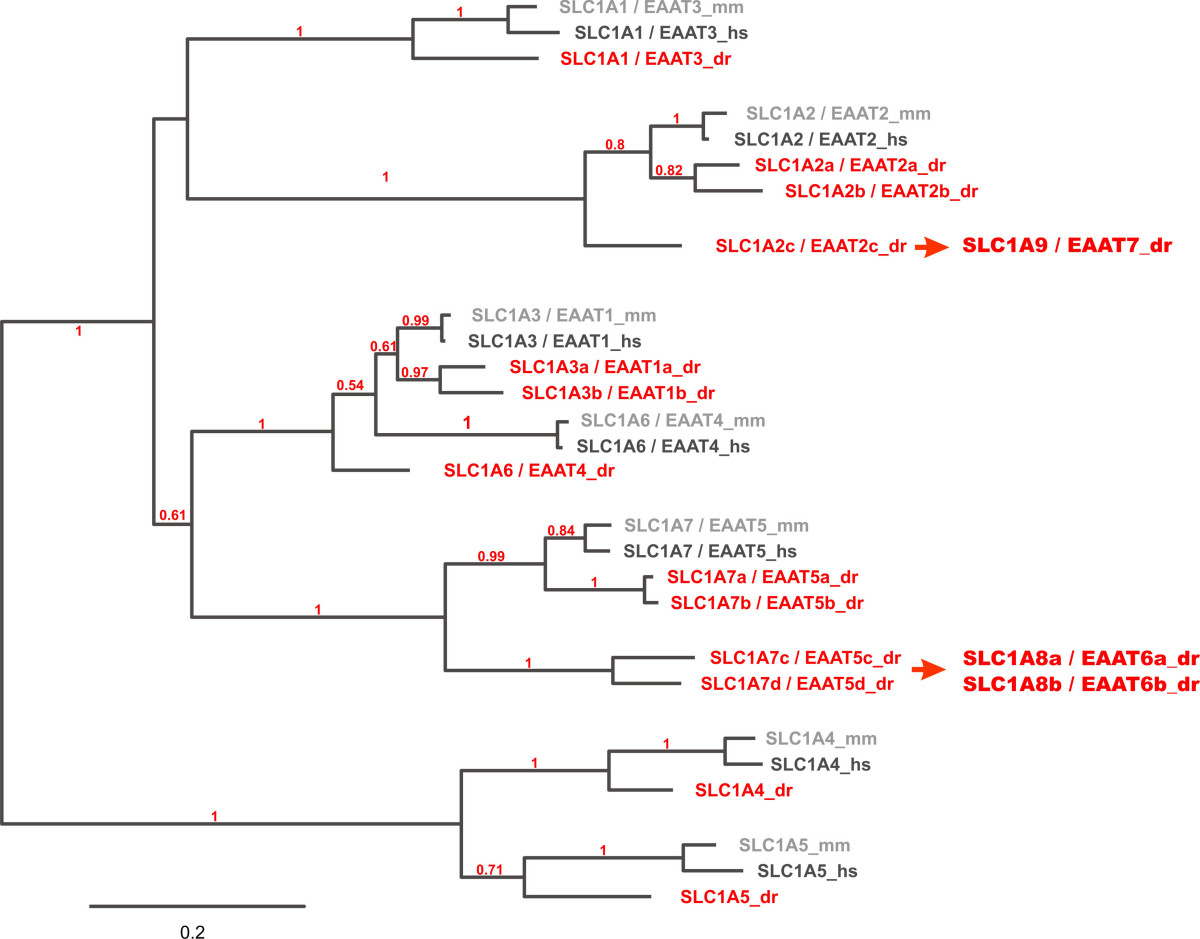

Supplement: Additional file 6 — ZIP files containing several folders, each of which with TreeSnatcher Plus snapshot files, the original image and a text file. [file 1471-2105-13-110-S6.zip › 1471-2148-10-117-2/1471-2148-10-117-2-l.jpg]

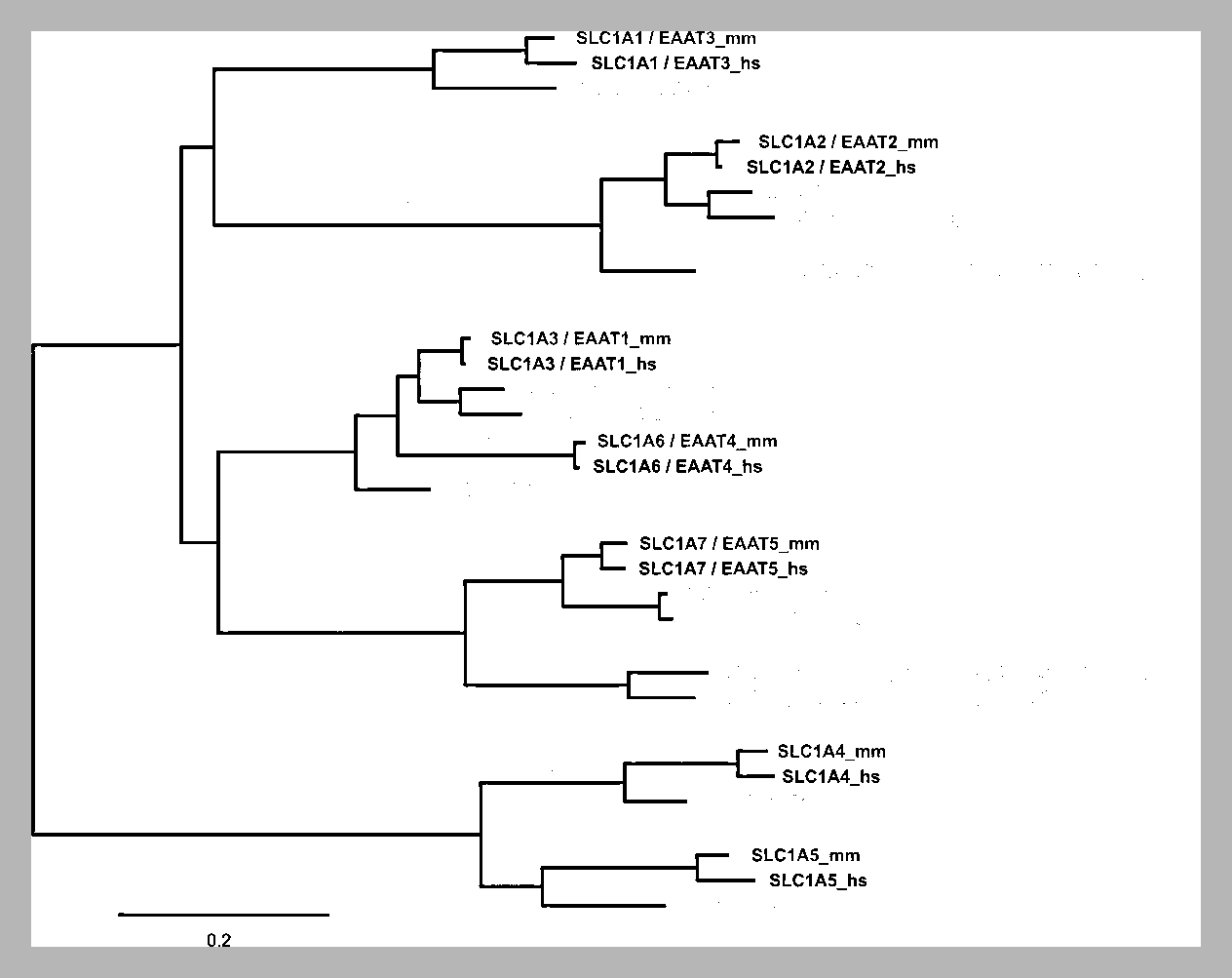

Supplement: Additional file 6 — ZIP files containing several folders, each of which with TreeSnatcher Plus snapshot files, the original image and a text file. [file 1471-2105-13-110-S6.zip › 1471-2148-10-117-2/1471-2148-10-117-2-l_b.PNG]

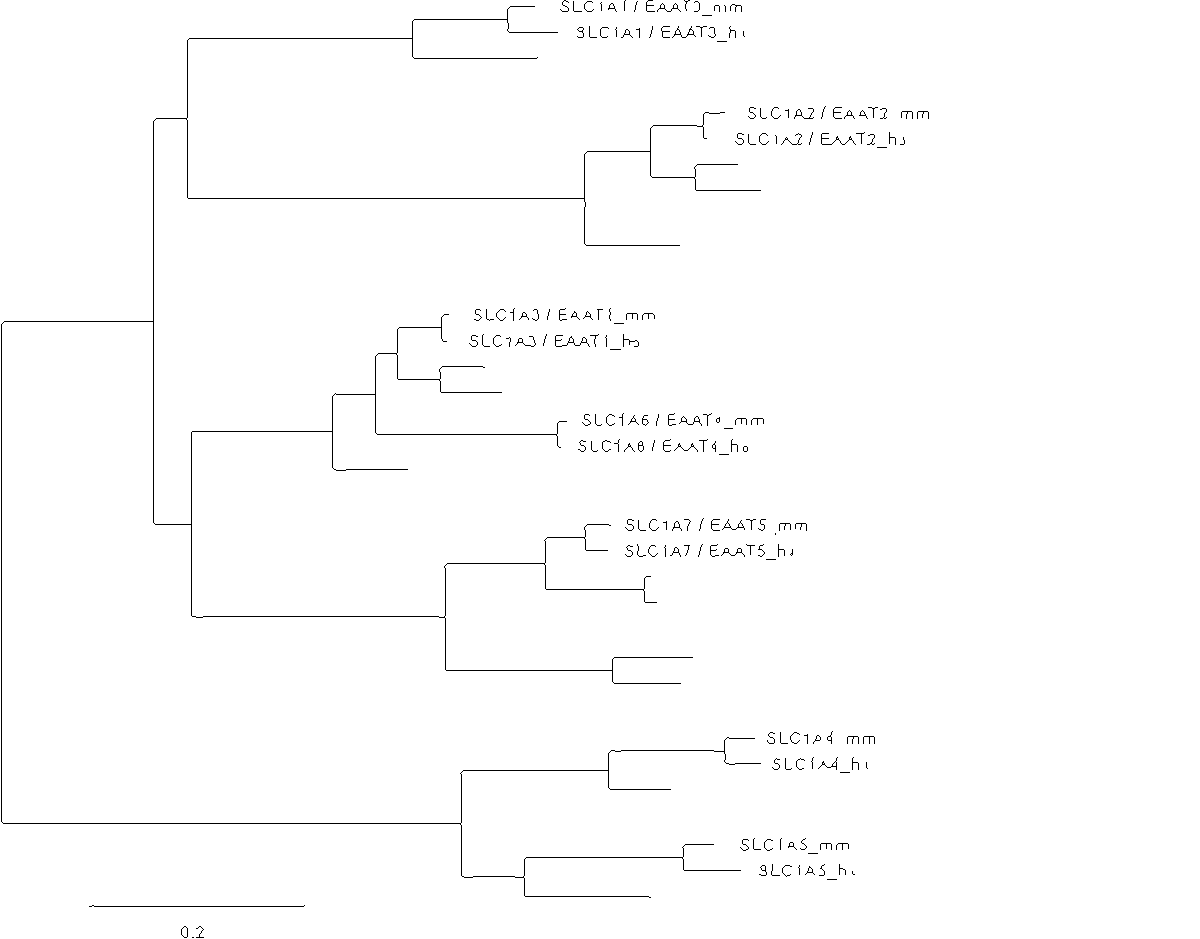

Supplement: Additional file 6 — ZIP files containing several folders, each of which with TreeSnatcher Plus snapshot files, the original image and a text file. [file 1471-2105-13-110-S6.zip › 1471-2148-10-117-2/1471-2148-10-117-2-l_c.PNG]

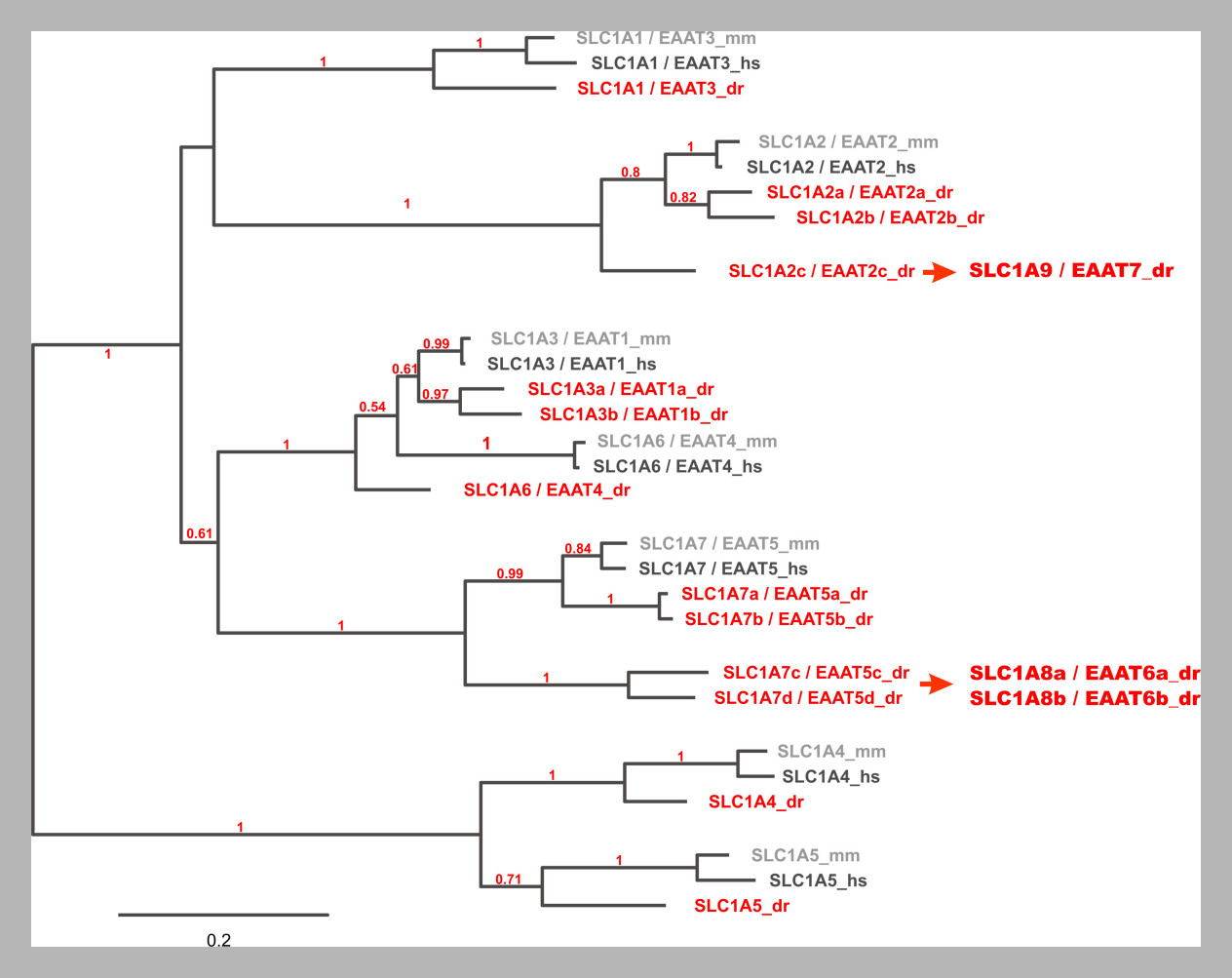

Supplement: Additional file 6 — ZIP files containing several folders, each of which with TreeSnatcher Plus snapshot files, the original image and a text file. [file 1471-2105-13-110-S6.zip › 1471-2148-10-117-2/1471-2148-10-117-2-l_o.PNG]

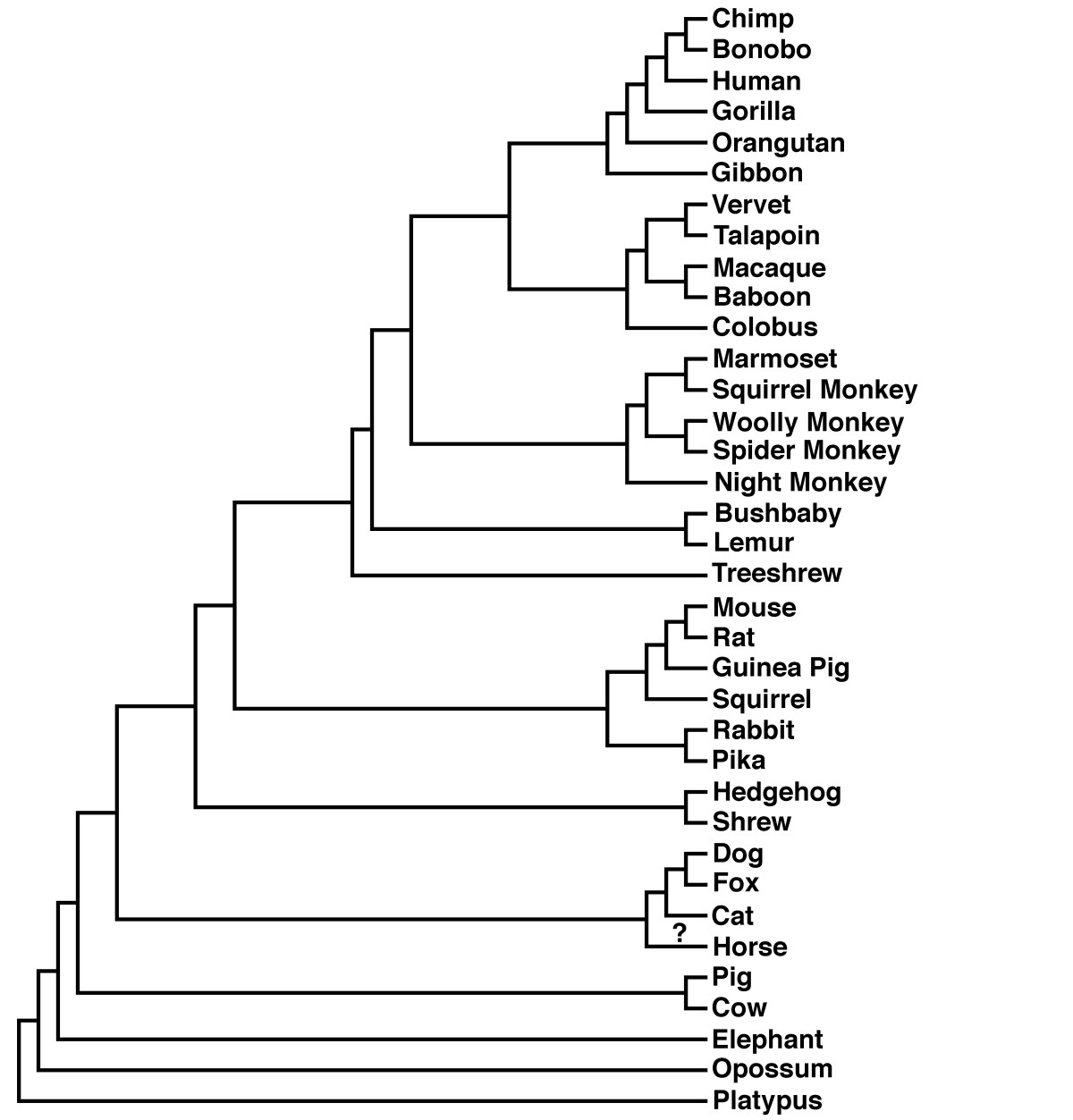

Supplement: Additional file 6 — ZIP files containing several folders, each of which with TreeSnatcher Plus snapshot files, the original image and a text file. [file 1471-2105-13-110-S6.zip › 1471-2148-10-39-1/1471-2148-10-39-1-l.jpg]

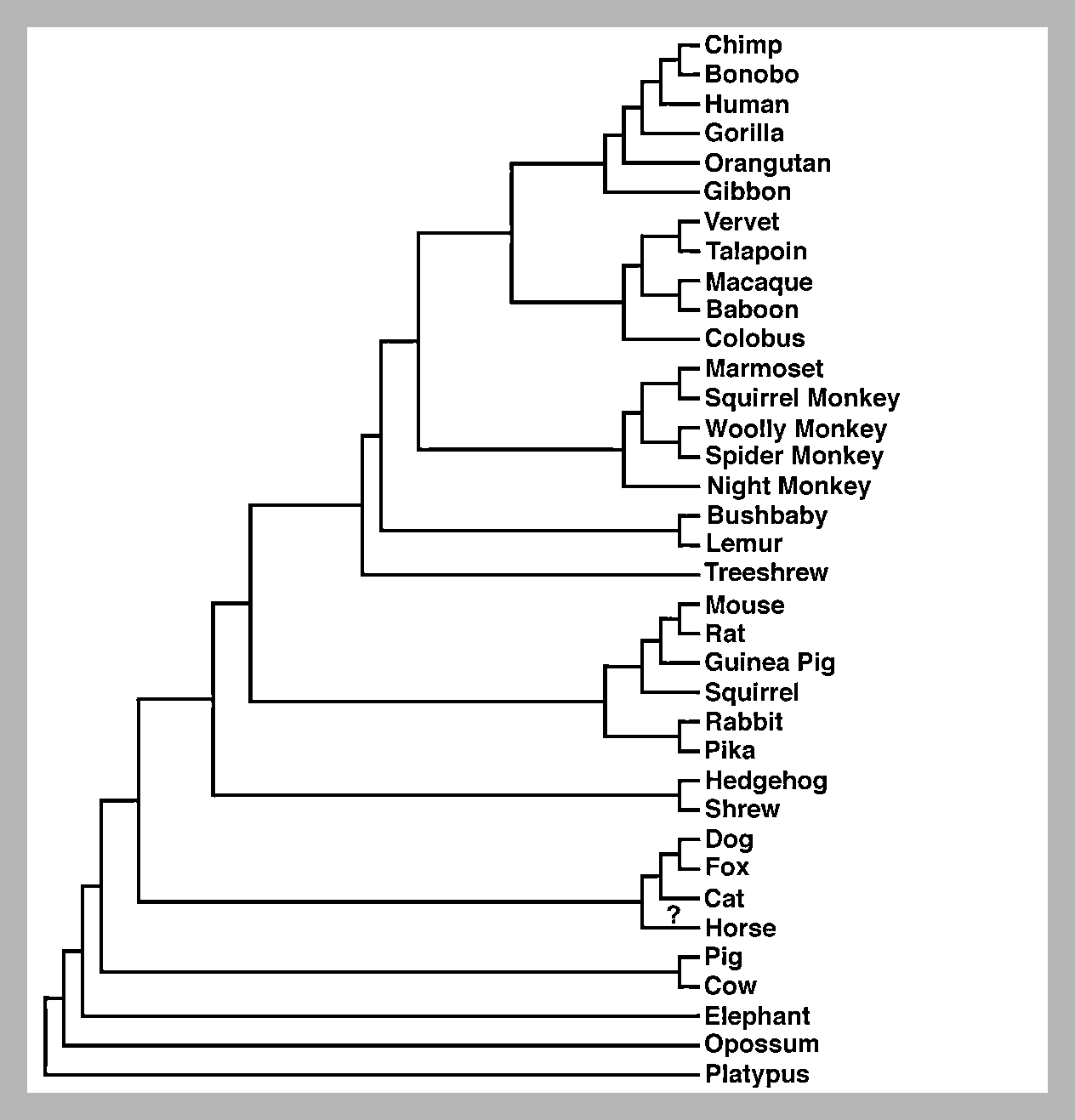

Supplement: Additional file 6 — ZIP files containing several folders, each of which with TreeSnatcher Plus snapshot files, the original image and a text file. [file 1471-2105-13-110-S6.zip › 1471-2148-10-39-1/1471-2148-10-39-1-l_b.PNG]

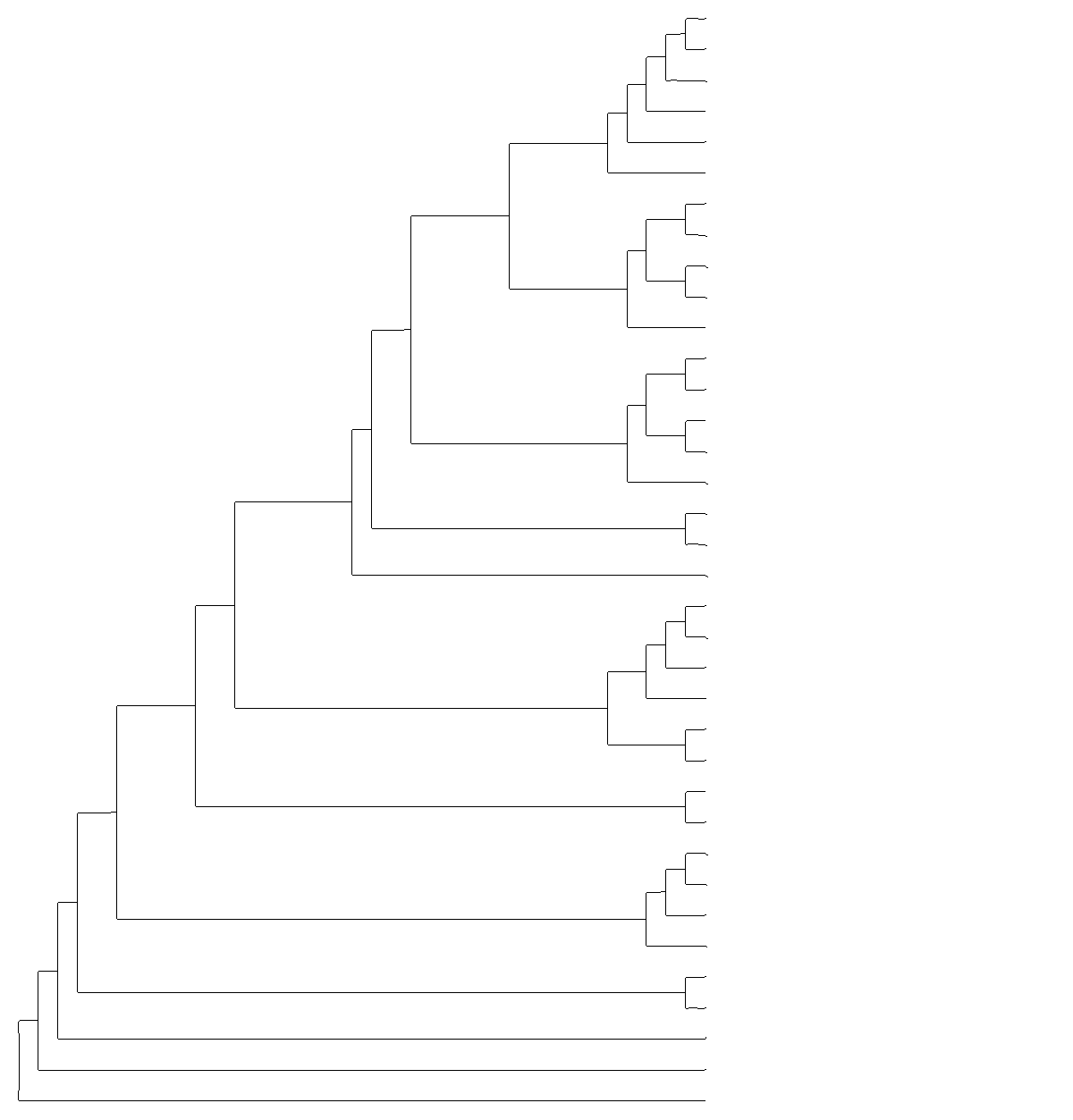

Supplement: Additional file 6 — ZIP files containing several folders, each of which with TreeSnatcher Plus snapshot files, the original image and a text file. [file 1471-2105-13-110-S6.zip › 1471-2148-10-39-1/1471-2148-10-39-1-l_c.PNG]

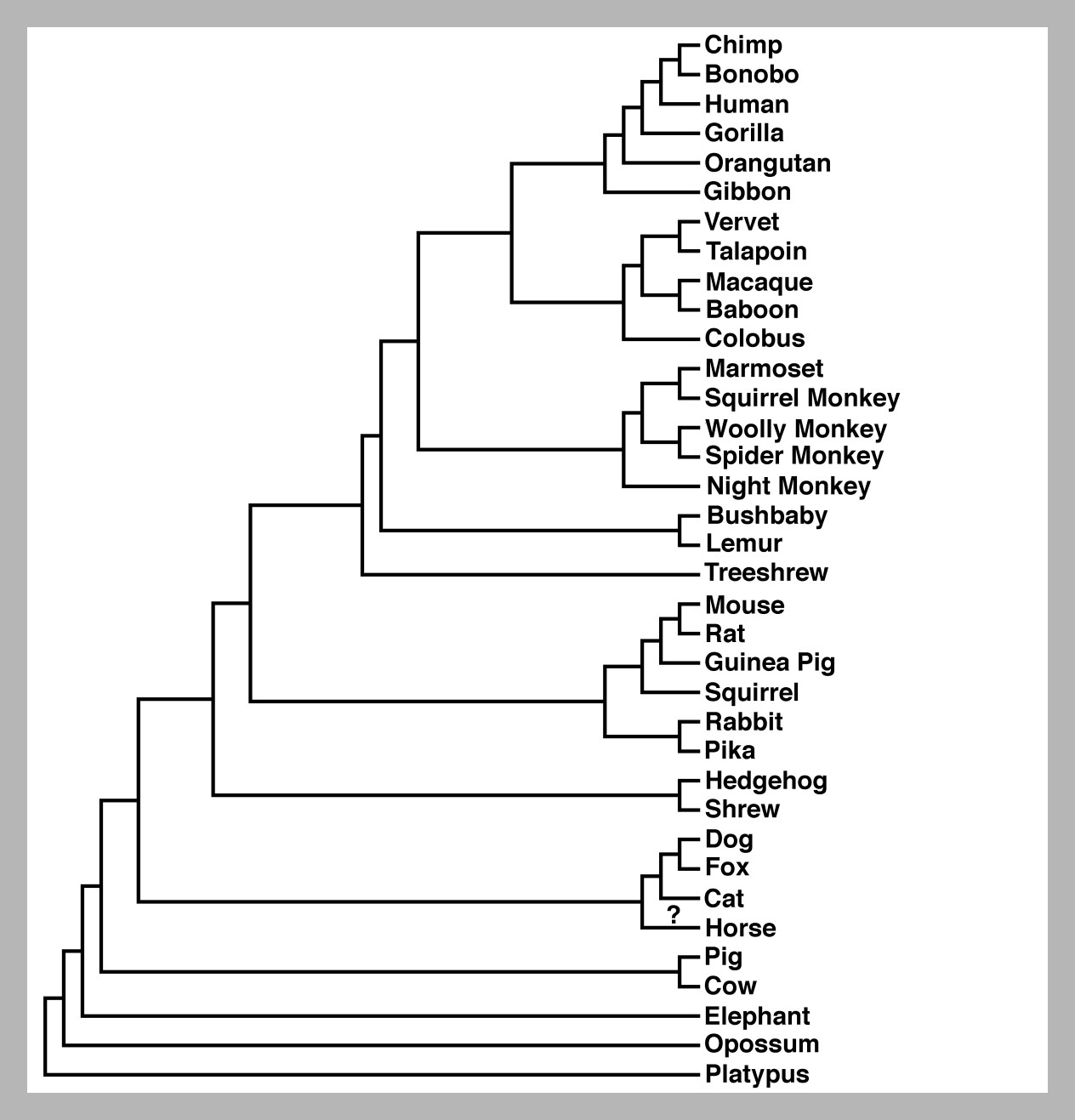

Supplement: Additional file 6 — ZIP files containing several folders, each of which with TreeSnatcher Plus snapshot files, the original image and a text file. [file 1471-2105-13-110-S6.zip › 1471-2148-10-39-1/1471-2148-10-39-1-l_o.PNG]

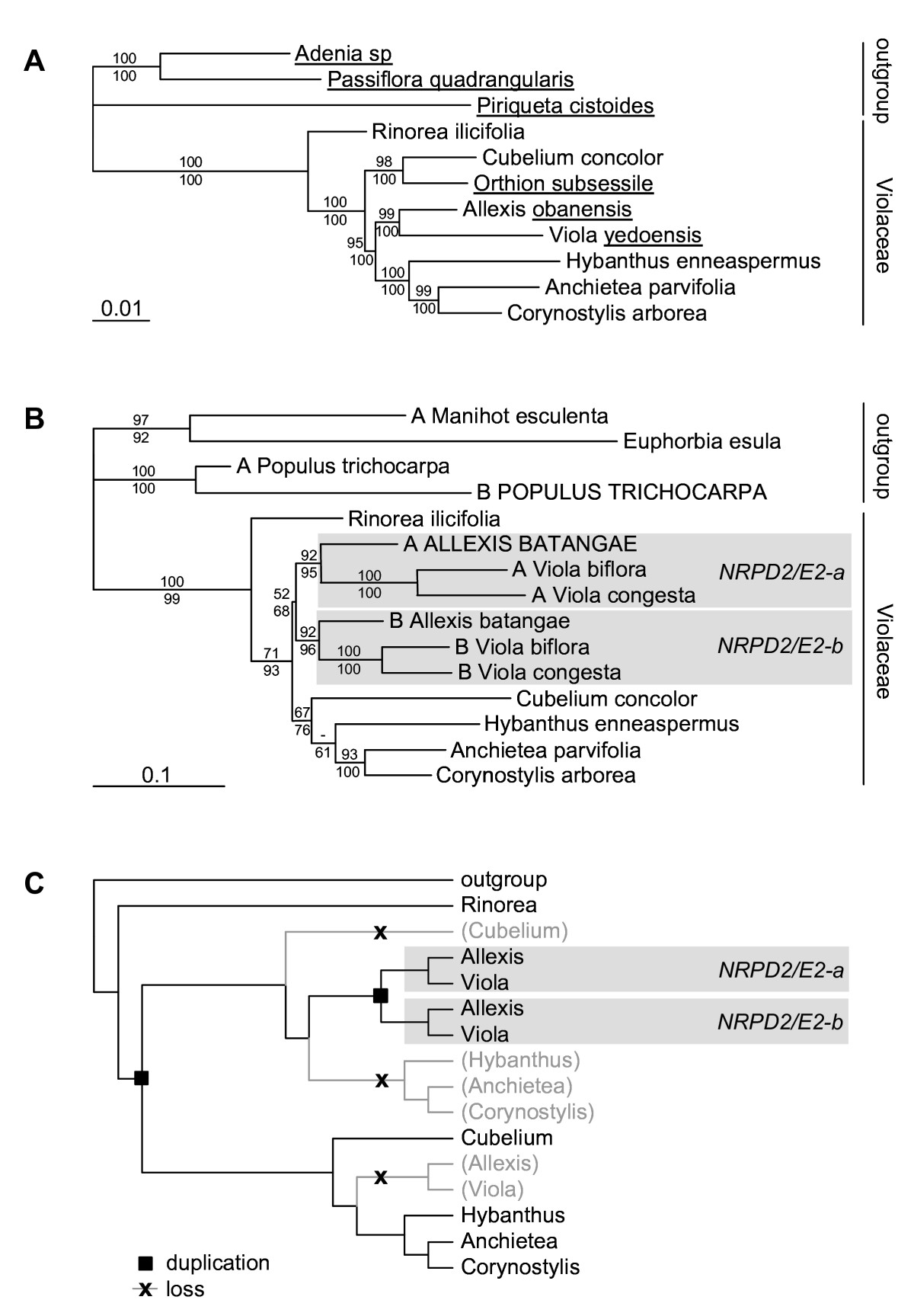

Supplement: Additional file 6 — ZIP files containing several folders, each of which with TreeSnatcher Plus snapshot files, the original image and a text file. [file 1471-2105-13-110-S6.zip › 1471-2148-10-45-2/1471-2148-10-45-2-l.jpg]

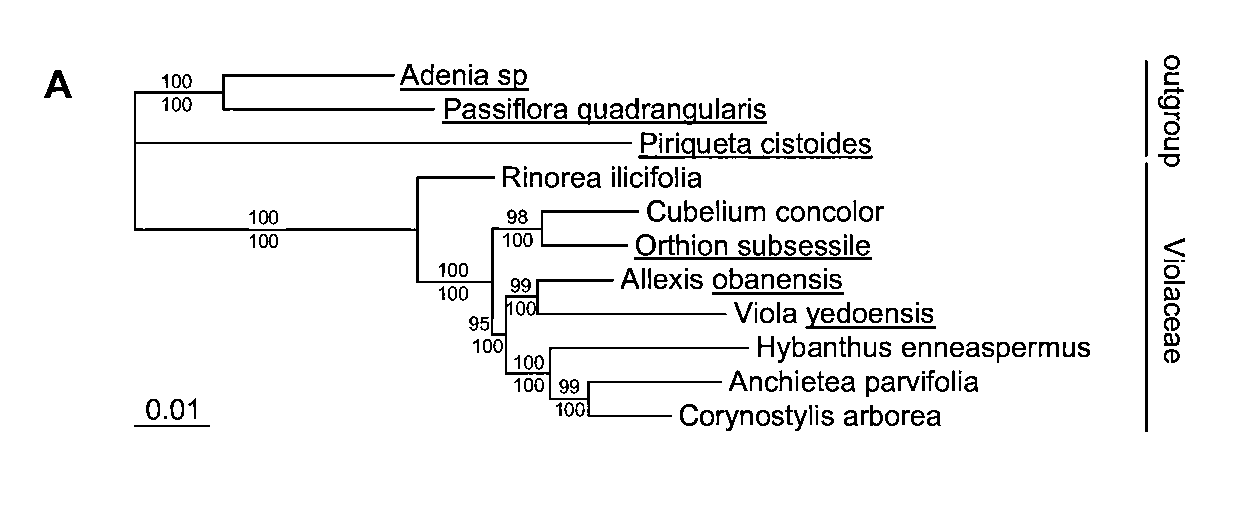

Supplement: Additional file 6 — ZIP files containing several folders, each of which with TreeSnatcher Plus snapshot files, the original image and a text file. [file 1471-2105-13-110-S6.zip › 1471-2148-10-45-2/1471-2148-10-45-2-l_1st_b.PNG]

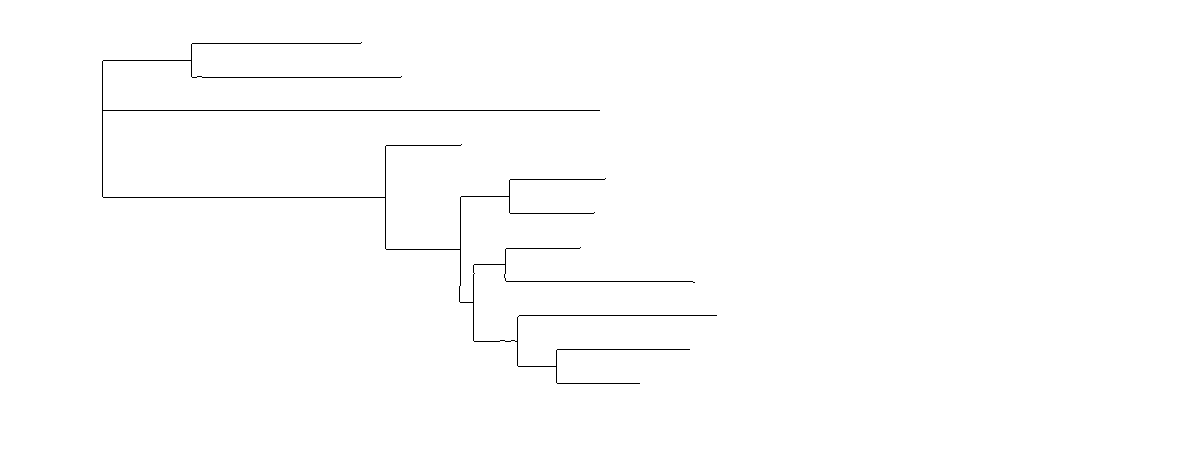

Supplement: Additional file 6 — ZIP files containing several folders, each of which with TreeSnatcher Plus snapshot files, the original image and a text file. [file 1471-2105-13-110-S6.zip › 1471-2148-10-45-2/1471-2148-10-45-2-l_1st_c.PNG]

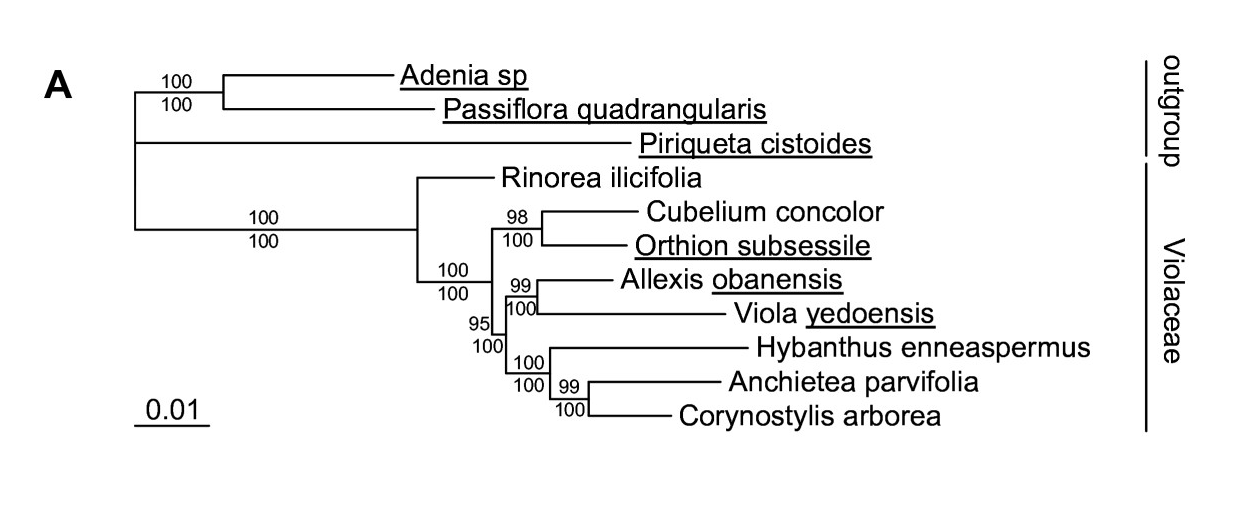

Supplement: Additional file 6 — ZIP files containing several folders, each of which with TreeSnatcher Plus snapshot files, the original image and a text file. [file 1471-2105-13-110-S6.zip › 1471-2148-10-45-2/1471-2148-10-45-2-l_1st_o.PNG]

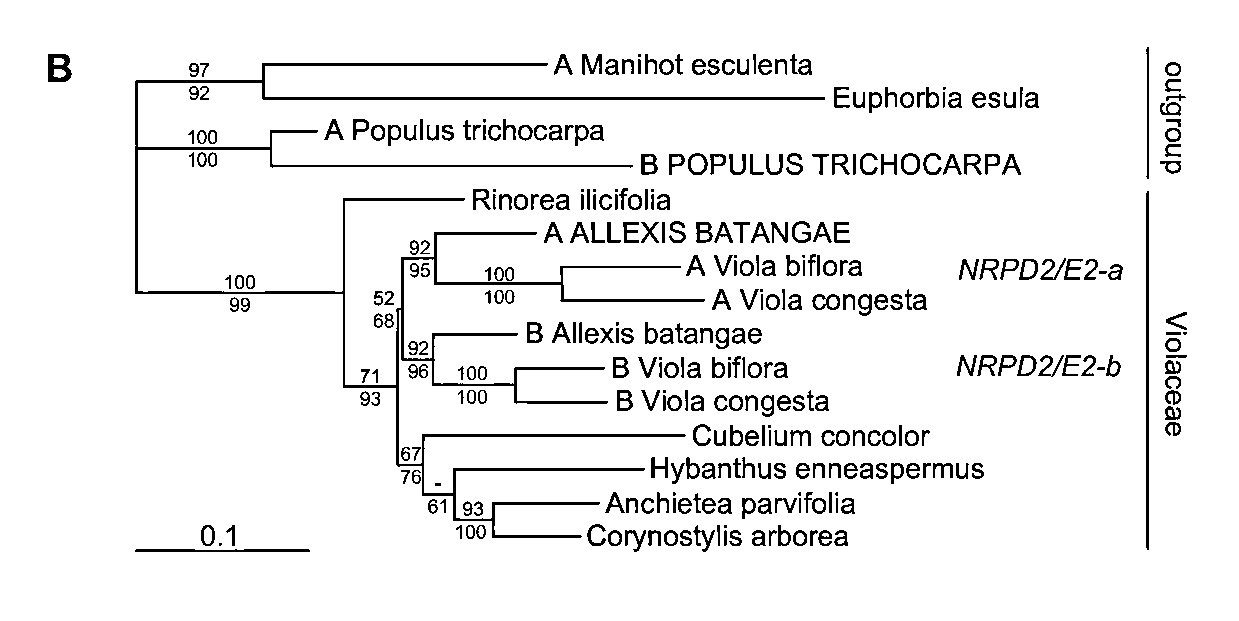

Supplement: Additional file 6 — ZIP files containing several folders, each of which with TreeSnatcher Plus snapshot files, the original image and a text file. [file 1471-2105-13-110-S6.zip › 1471-2148-10-45-2/1471-2148-10-45-2-l_2nd_b.PNG]

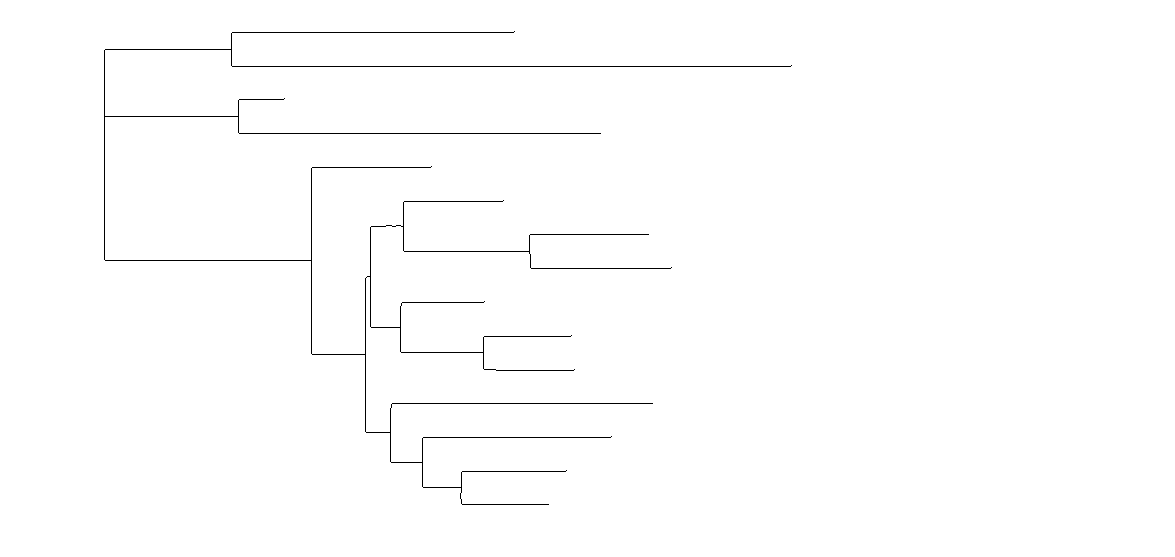

Supplement: Additional file 6 — ZIP files containing several folders, each of which with TreeSnatcher Plus snapshot files, the original image and a text file. [file 1471-2105-13-110-S6.zip › 1471-2148-10-45-2/1471-2148-10-45-2-l_2nd_c.PNG]

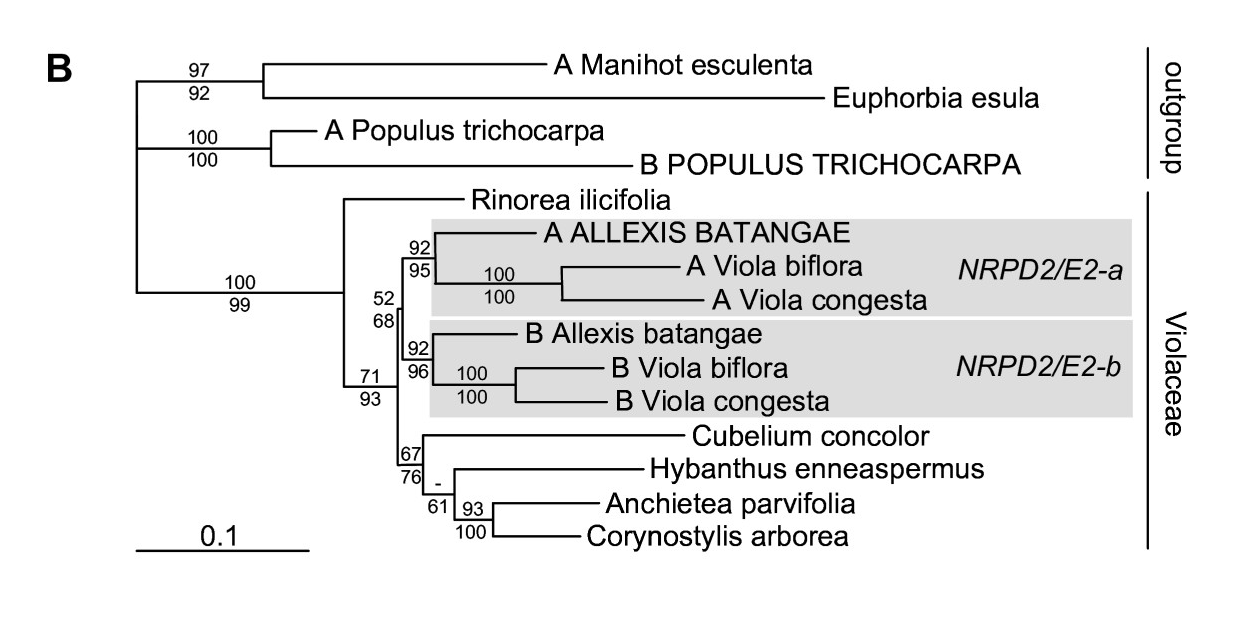

Supplement: Additional file 6 — ZIP files containing several folders, each of which with TreeSnatcher Plus snapshot files, the original image and a text file. [file 1471-2105-13-110-S6.zip › 1471-2148-10-45-2/1471-2148-10-45-2-l_2nd_o.PNG]

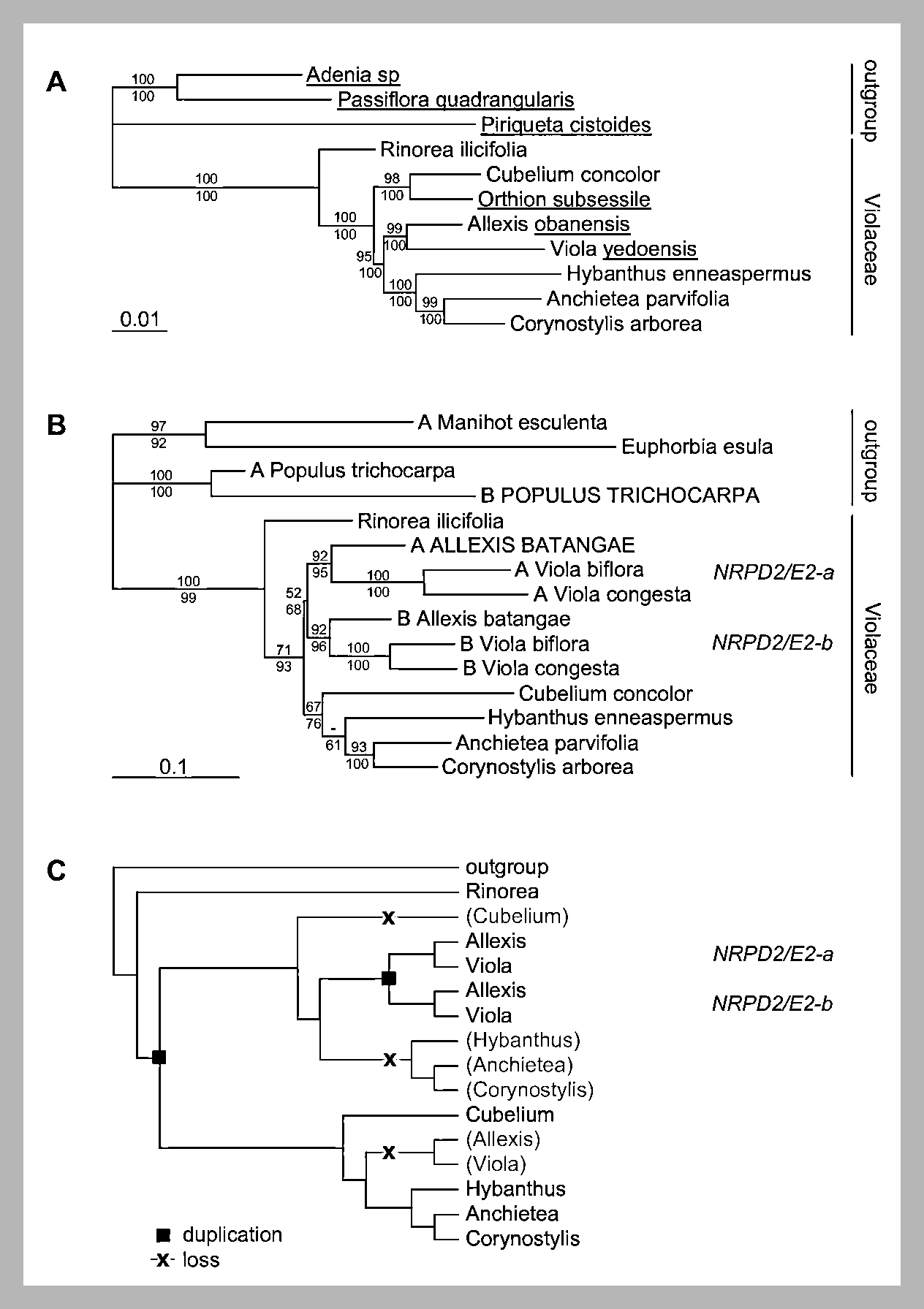

Supplement: Additional file 6 — ZIP files containing several folders, each of which with TreeSnatcher Plus snapshot files, the original image and a text file. [file 1471-2105-13-110-S6.zip › 1471-2148-10-45-2/1471-2148-10-45-2-l_3rd_b.PNG]

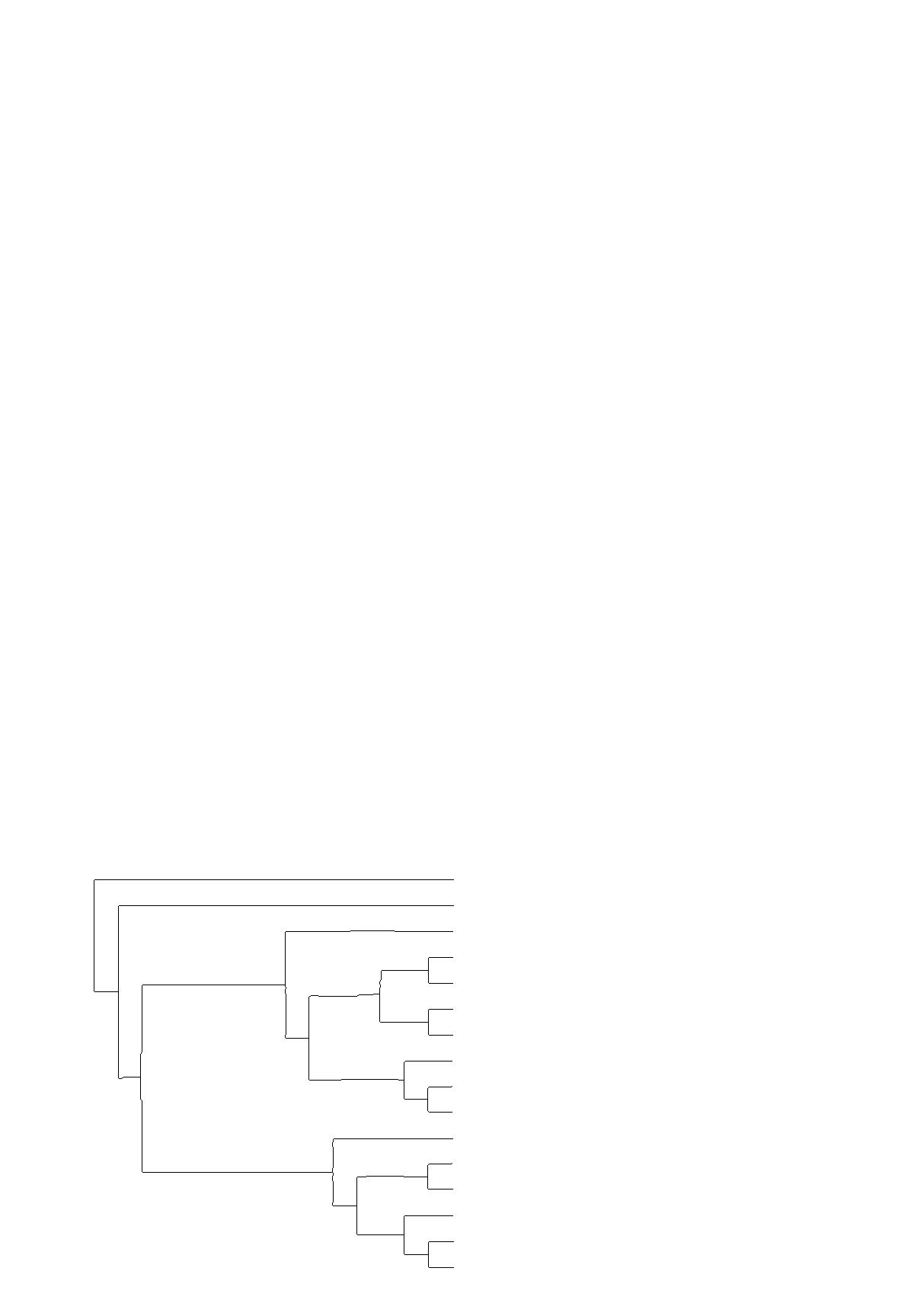

Supplement: Additional file 6 — ZIP files containing several folders, each of which with TreeSnatcher Plus snapshot files, the original image and a text file. [file 1471-2105-13-110-S6.zip › 1471-2148-10-45-2/1471-2148-10-45-2-l_3rd_c.PNG]

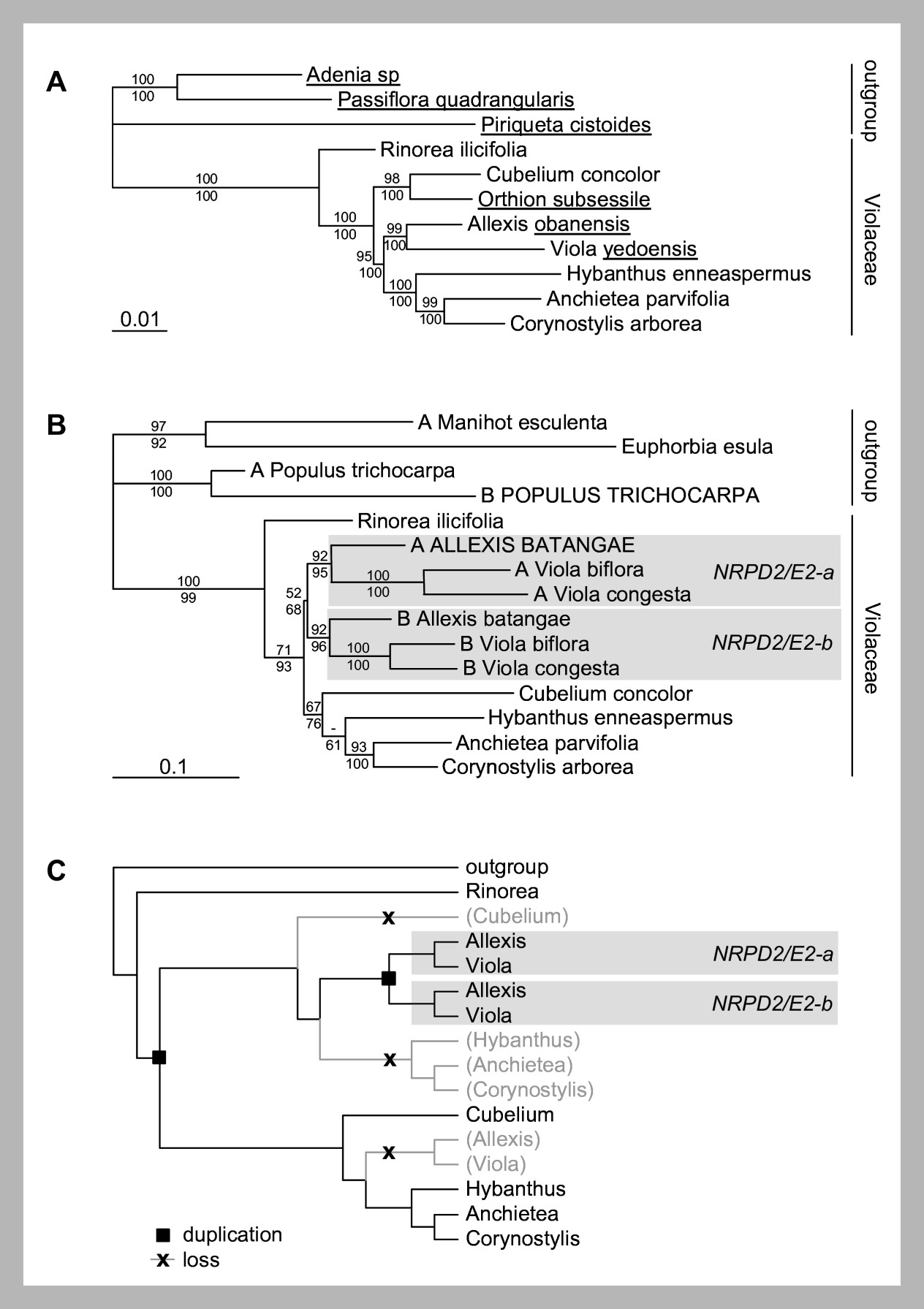

Supplement: Additional file 6 — ZIP files containing several folders, each of which with TreeSnatcher Plus snapshot files, the original image and a text file. [file 1471-2105-13-110-S6.zip › 1471-2148-10-45-2/1471-2148-10-45-2-l_3rd_o.PNG]

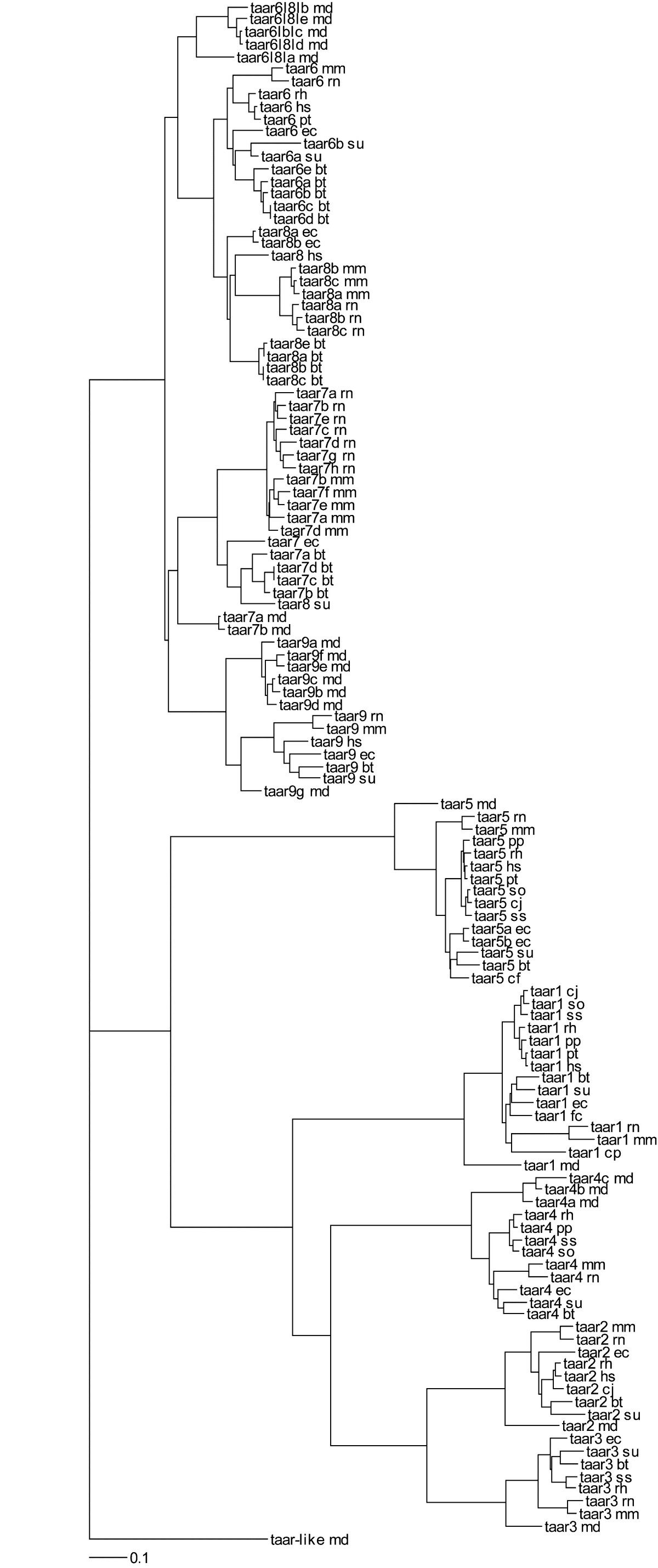

Supplement: Additional file 6 — ZIP files containing several folders, each of which with TreeSnatcher Plus snapshot files, the original image and a text file. [file 1471-2105-13-110-S6.zip › 1471-2148-10-51-1/1471-2148-10-51-1-l.jpg]

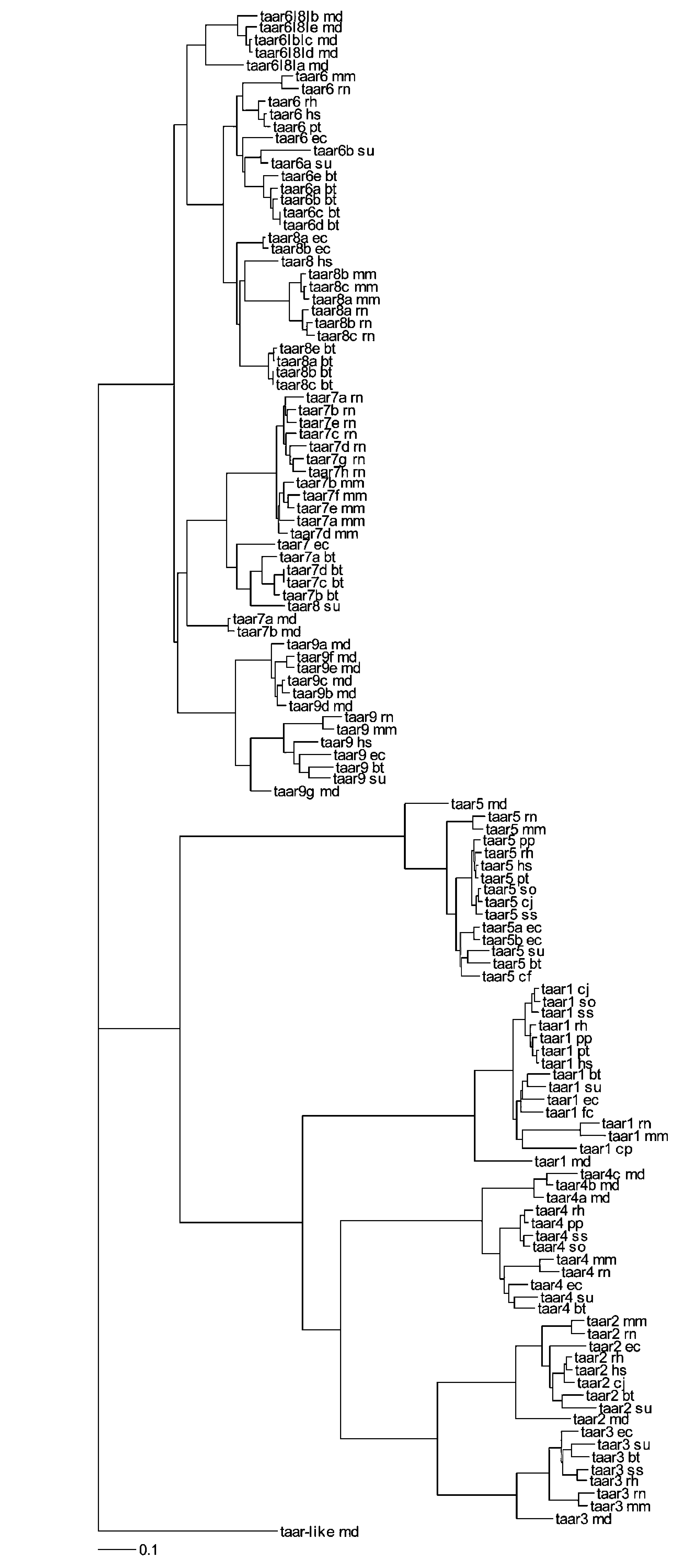

Supplement: Additional file 6 — ZIP files containing several folders, each of which with TreeSnatcher Plus snapshot files, the original image and a text file. [file 1471-2105-13-110-S6.zip › 1471-2148-10-51-1/1471-2148-10-51-1-l_b.PNG]

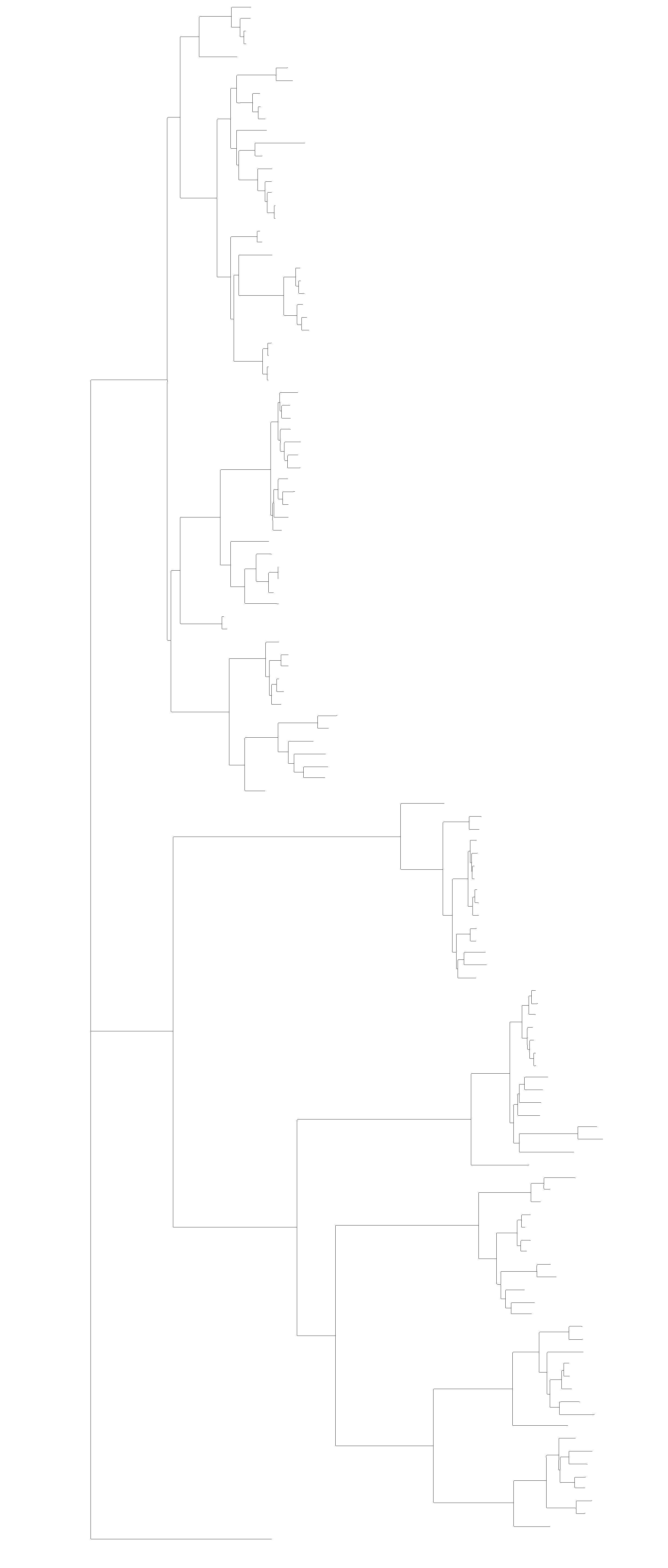

Supplement: Additional file 6 — ZIP files containing several folders, each of which with TreeSnatcher Plus snapshot files, the original image and a text file. [file 1471-2105-13-110-S6.zip › 1471-2148-10-51-1/1471-2148-10-51-1-l_c.PNG]

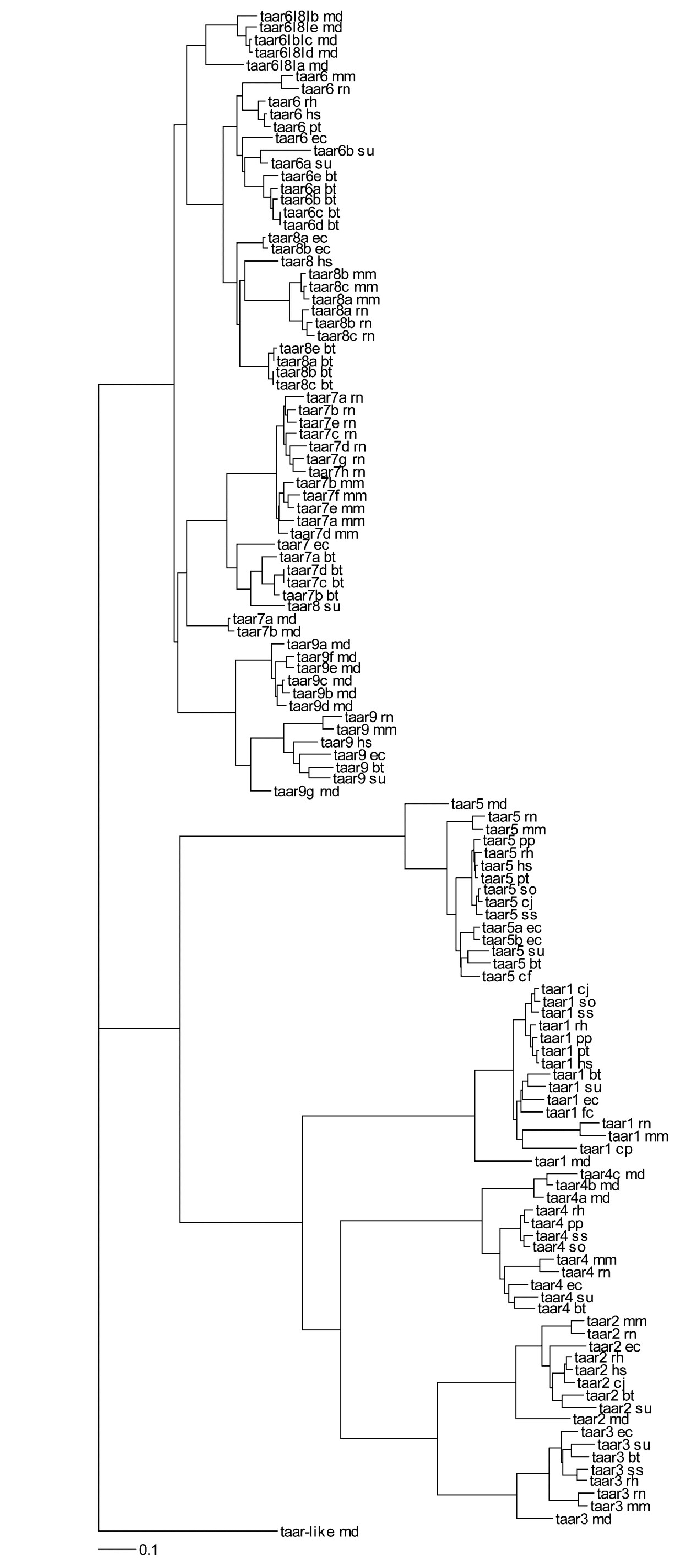

Supplement: Additional file 6 — ZIP files containing several folders, each of which with TreeSnatcher Plus snapshot files, the original image and a text file. [file 1471-2105-13-110-S6.zip › 1471-2148-10-51-1/1471-2148-10-51-1-l_o.PNG]

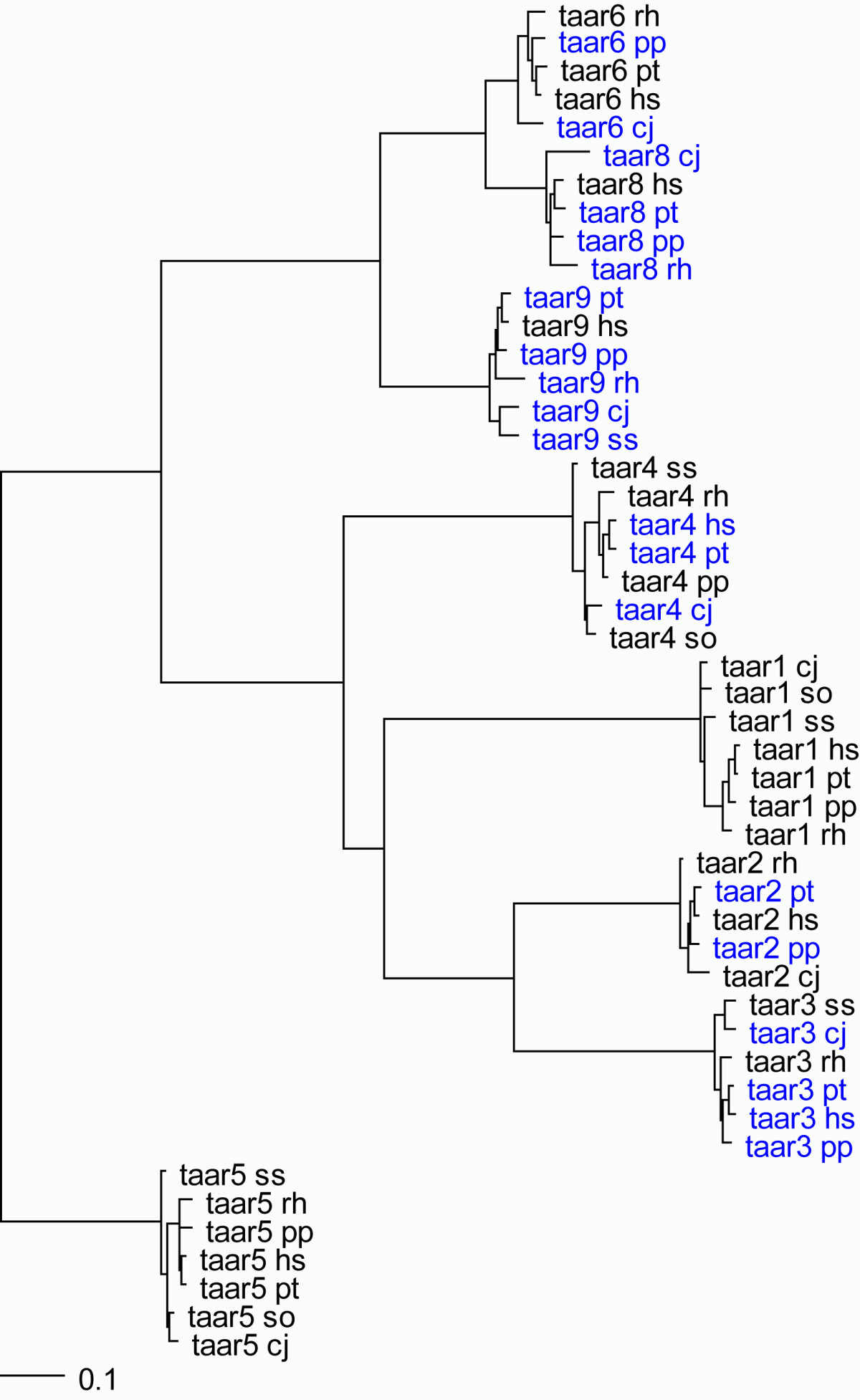

Supplement: Additional file 6 — ZIP files containing several folders, each of which with TreeSnatcher Plus snapshot files, the original image and a text file. [file 1471-2105-13-110-S6.zip › 1471-2148-10-51-2/1471-2148-10-51-2-l.jpg]

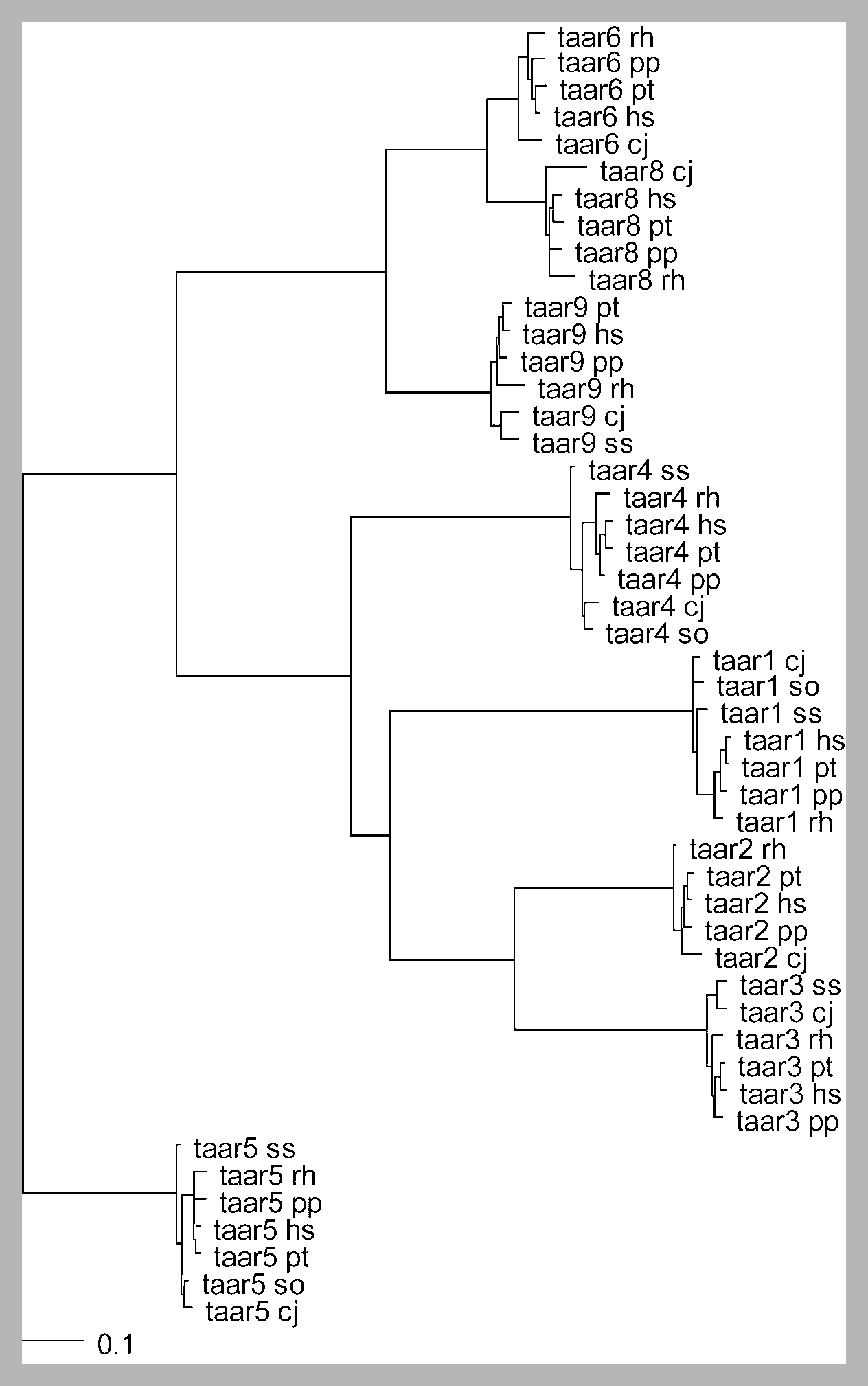

Supplement: Additional file 6 — ZIP files containing several folders, each of which with TreeSnatcher Plus snapshot files, the original image and a text file. [file 1471-2105-13-110-S6.zip › 1471-2148-10-51-2/1471-2148-10-51-2-l_b.PNG]

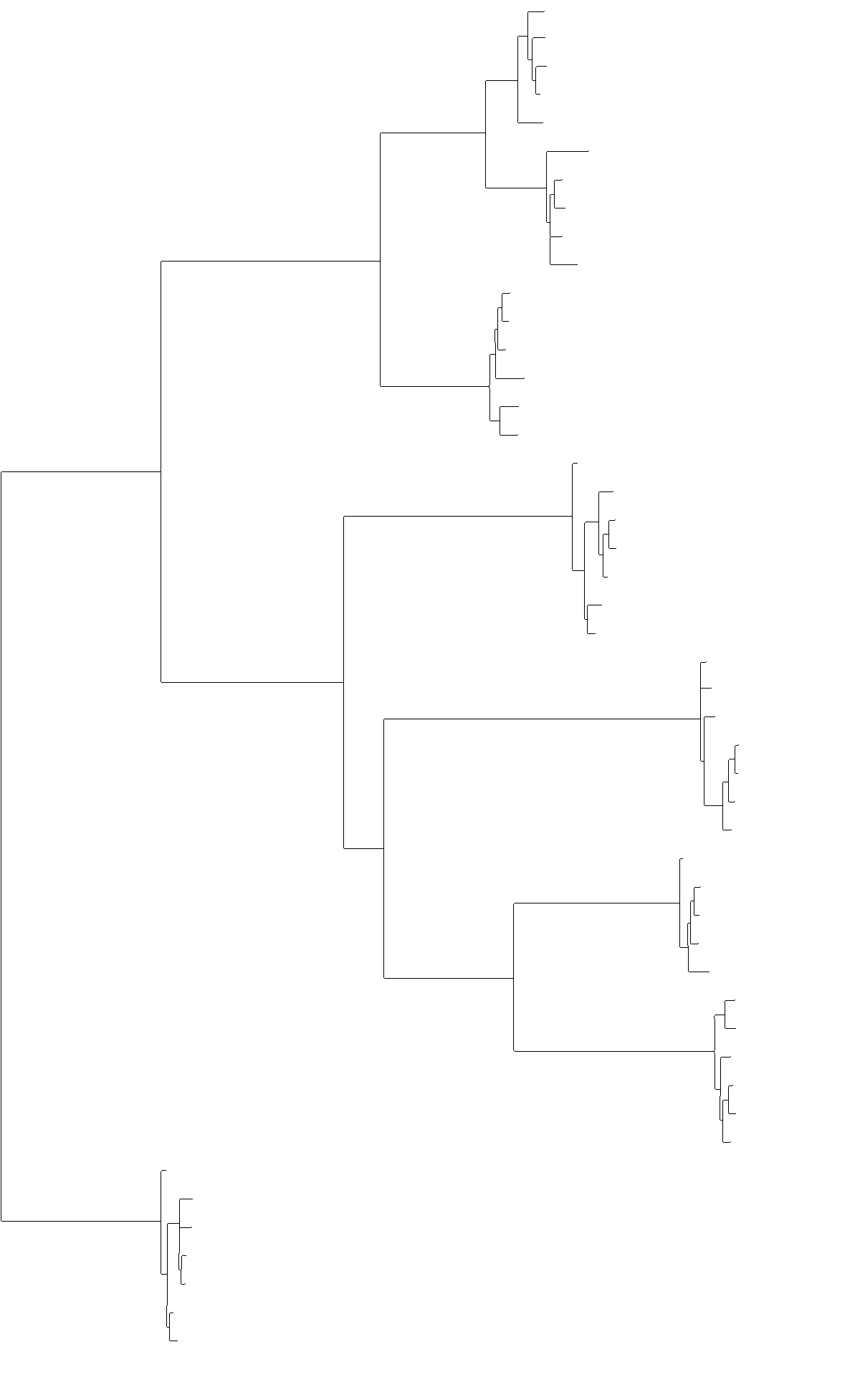

Supplement: Additional file 6 — ZIP files containing several folders, each of which with TreeSnatcher Plus snapshot files, the original image and a text file. [file 1471-2105-13-110-S6.zip › 1471-2148-10-51-2/1471-2148-10-51-2-l_c.PNG]

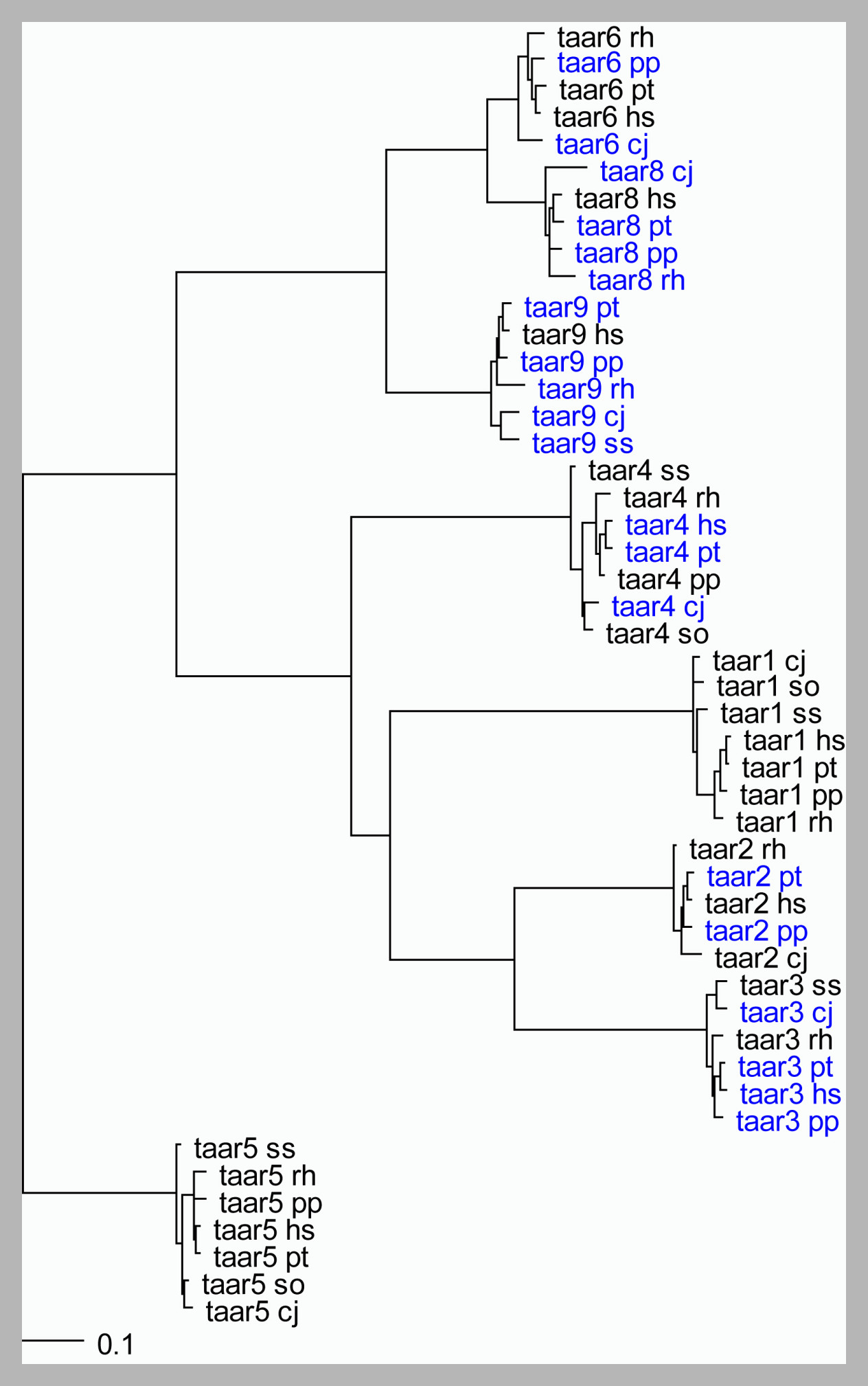

Supplement: Additional file 6 — ZIP files containing several folders, each of which with TreeSnatcher Plus snapshot files, the original image and a text file. [file 1471-2105-13-110-S6.zip › 1471-2148-10-51-2/1471-2148-10-51-2-l_o.PNG]

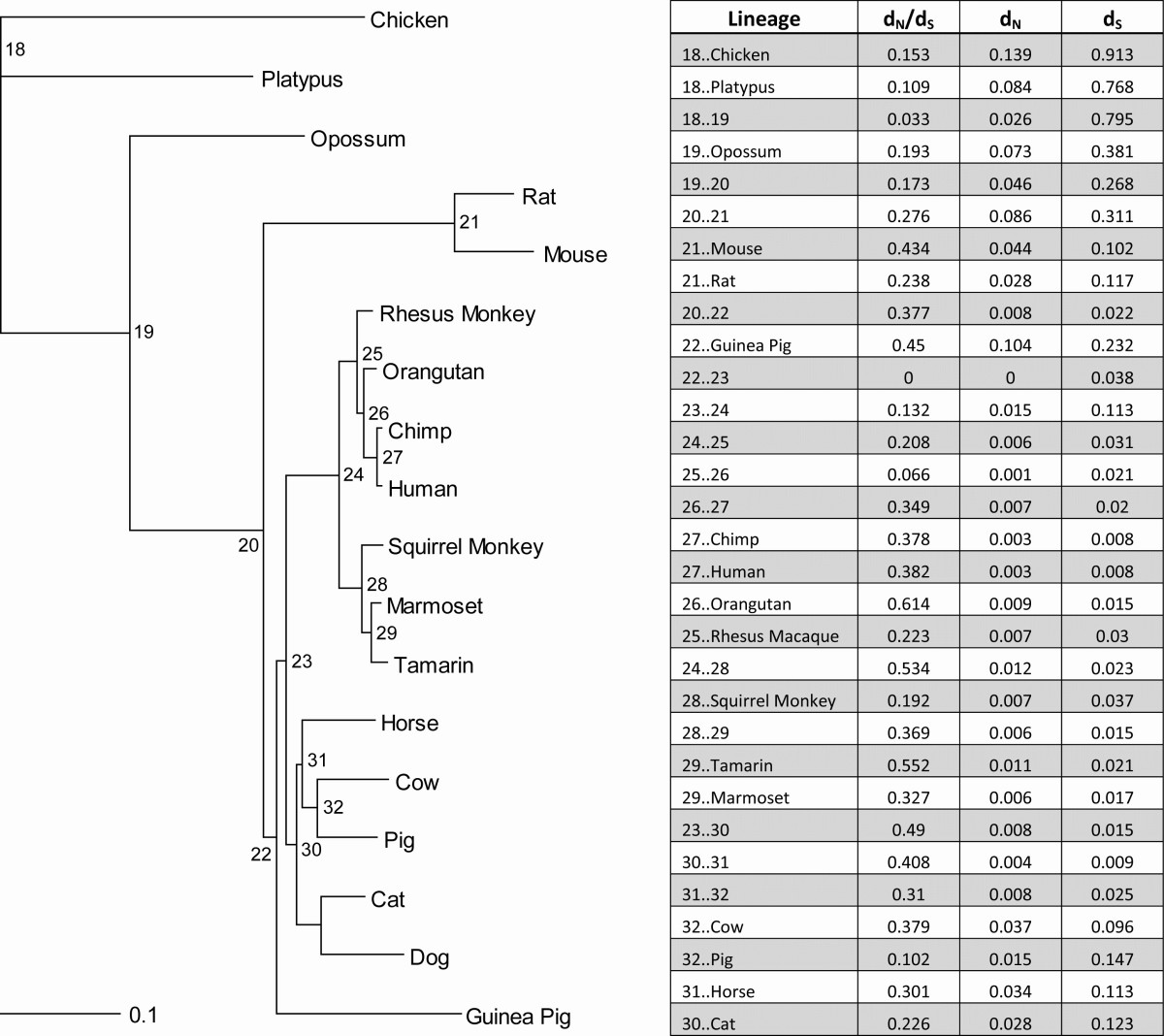

Supplement: Additional file 6 — ZIP files containing several folders, each of which with TreeSnatcher Plus snapshot files, the original image and a text file. [file 1471-2105-13-110-S6.zip › 1471-2148-10-51-3/1471-2148-10-51-3-l.jpg]

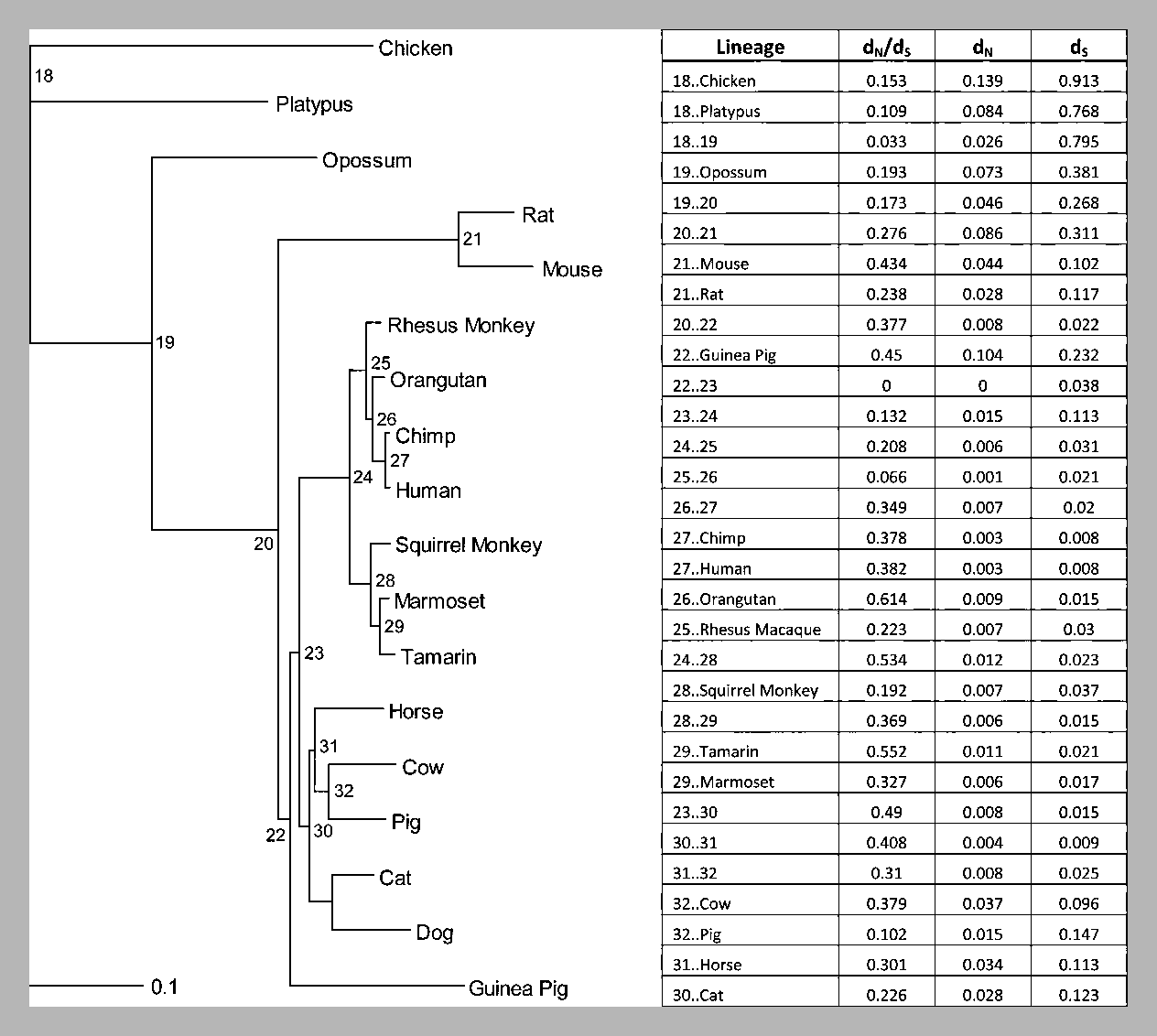

Supplement: Additional file 6 — ZIP files containing several folders, each of which with TreeSnatcher Plus snapshot files, the original image and a text file. [file 1471-2105-13-110-S6.zip › 1471-2148-10-51-3/1471-2148-10-51-3-l_b.PNG]

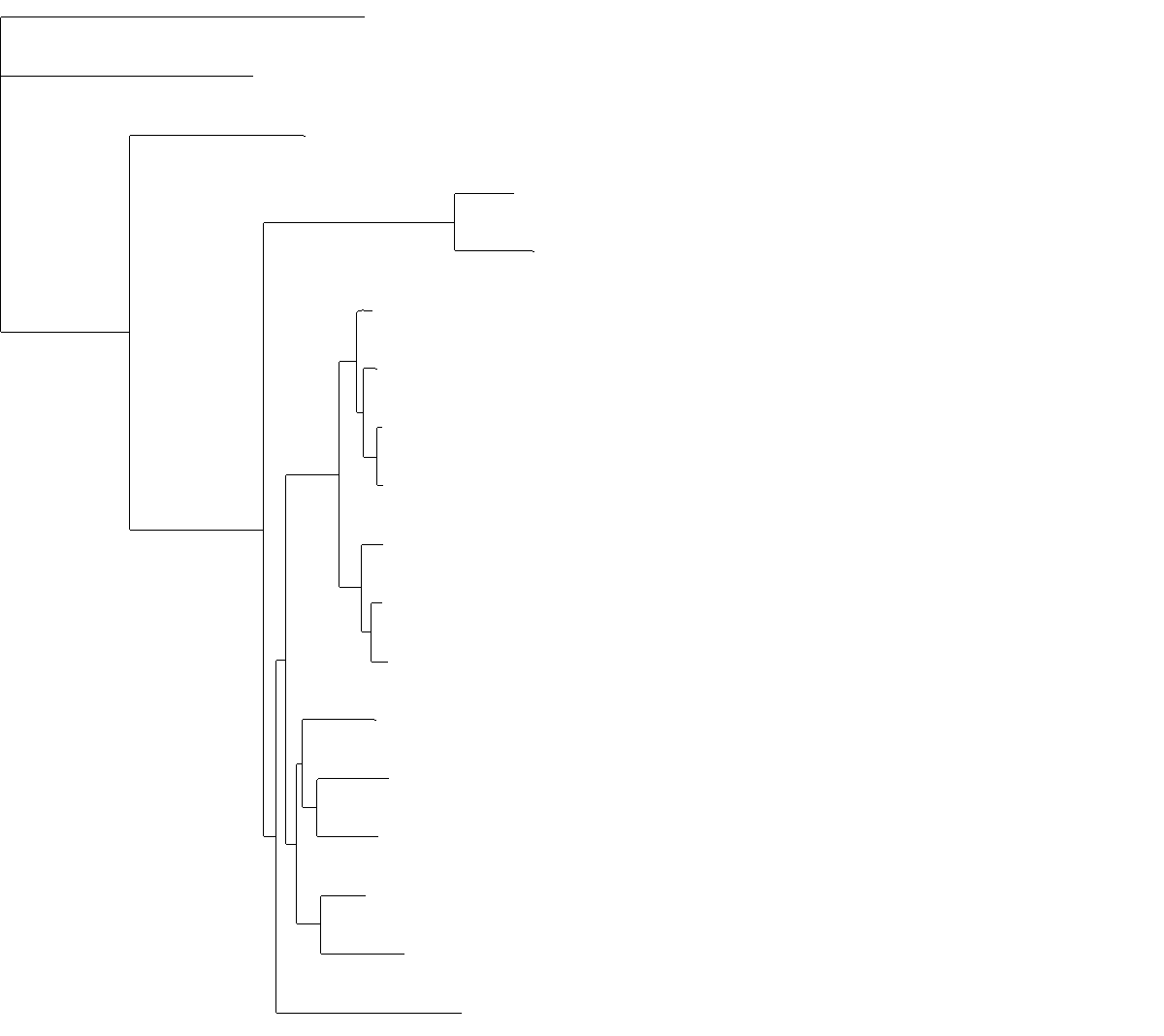

Supplement: Additional file 6 — ZIP files containing several folders, each of which with TreeSnatcher Plus snapshot files, the original image and a text file. [file 1471-2105-13-110-S6.zip › 1471-2148-10-51-3/1471-2148-10-51-3-l_c.PNG]

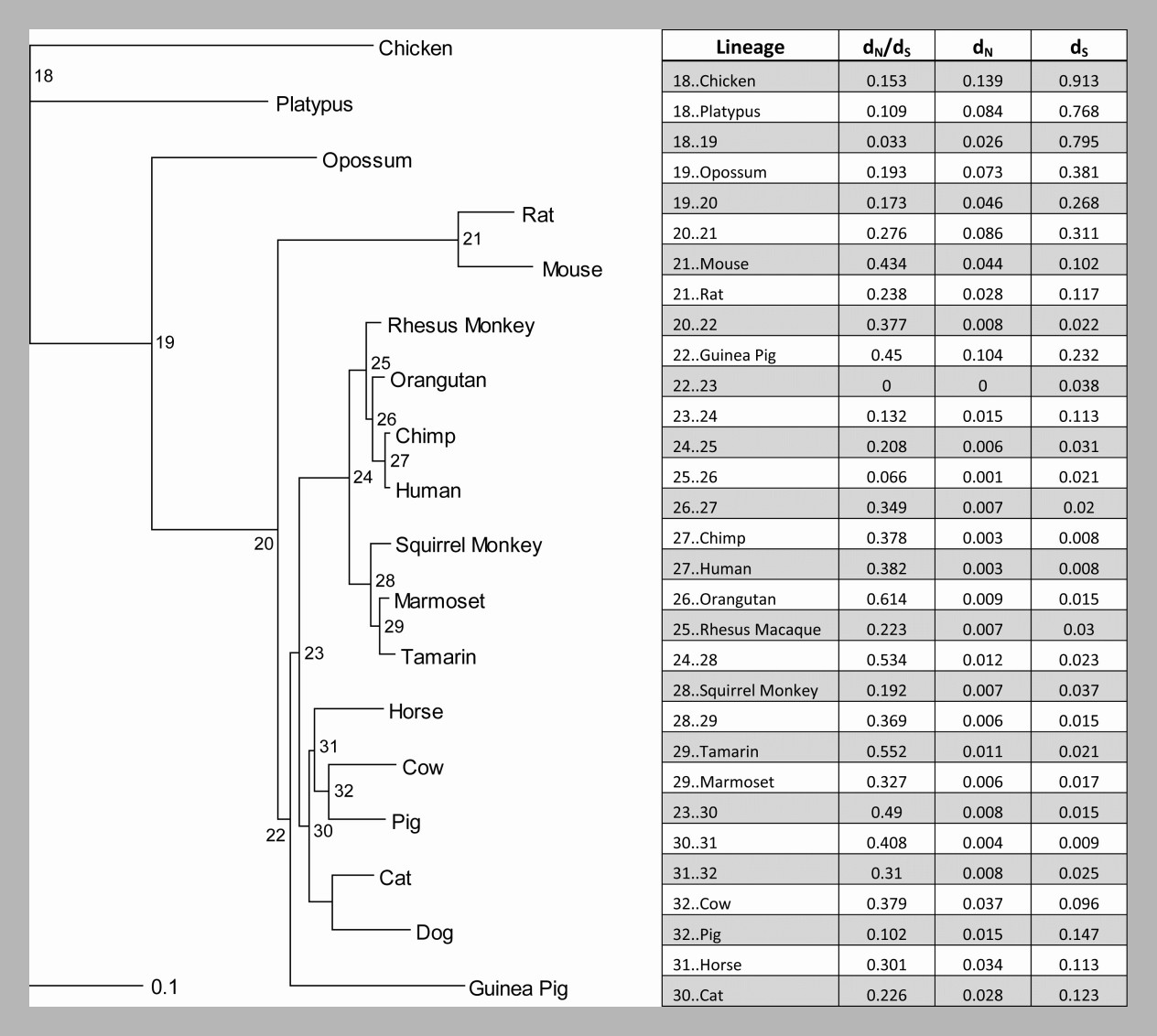

Supplement: Additional file 6 — ZIP files containing several folders, each of which with TreeSnatcher Plus snapshot files, the original image and a text file. [file 1471-2105-13-110-S6.zip › 1471-2148-10-51-3/1471-2148-10-51-3-l_o.PNG]

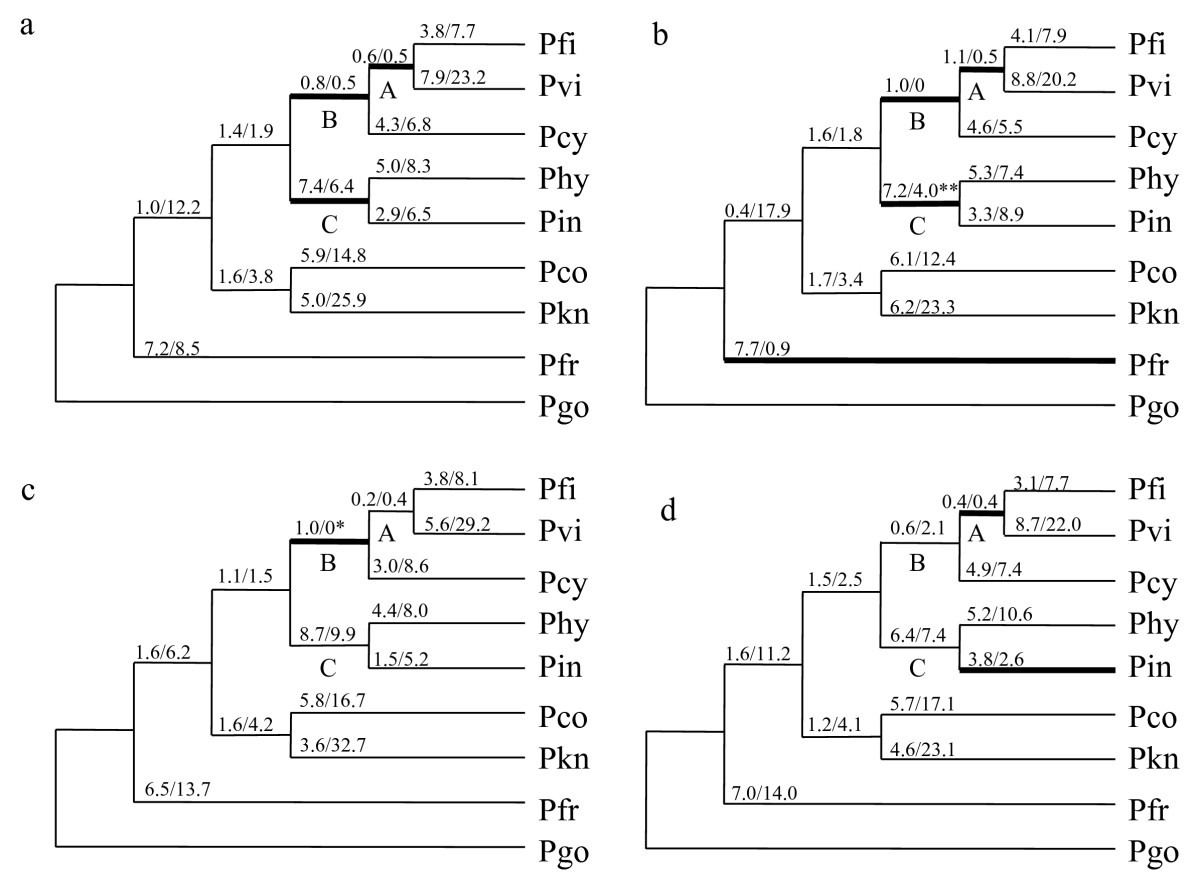

Supplement: Additional file 6 — ZIP files containing several folders, each of which with TreeSnatcher Plus snapshot files, the original image and a text file. [file 1471-2105-13-110-S6.zip › 1471-2148-10-52-2/1471-2148-10-52-2-l.jpg]

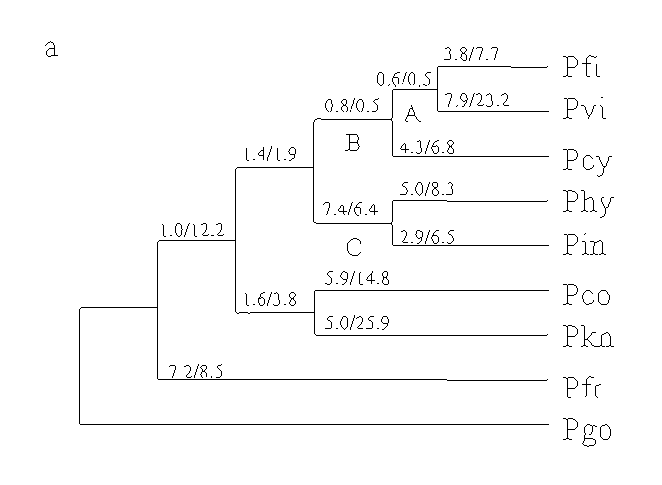

Supplement: Additional file 6 — ZIP files containing several folders, each of which with TreeSnatcher Plus snapshot files, the original image and a text file. [file 1471-2105-13-110-S6.zip › 1471-2148-10-52-2/1471-2148-10-52-2-l_b.PNG]

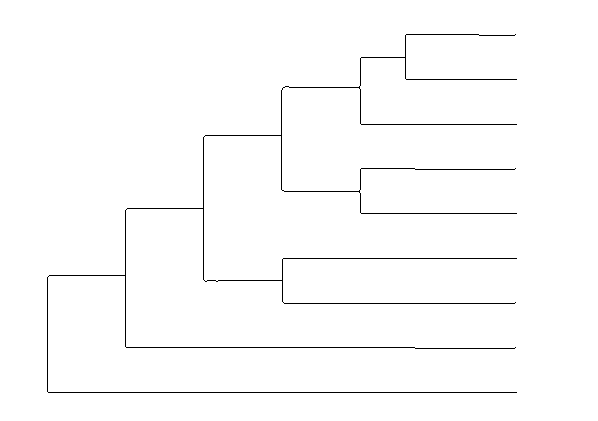

Supplement: Additional file 6 — ZIP files containing several folders, each of which with TreeSnatcher Plus snapshot files, the original image and a text file. [file 1471-2105-13-110-S6.zip › 1471-2148-10-52-2/1471-2148-10-52-2-l_c.PNG]

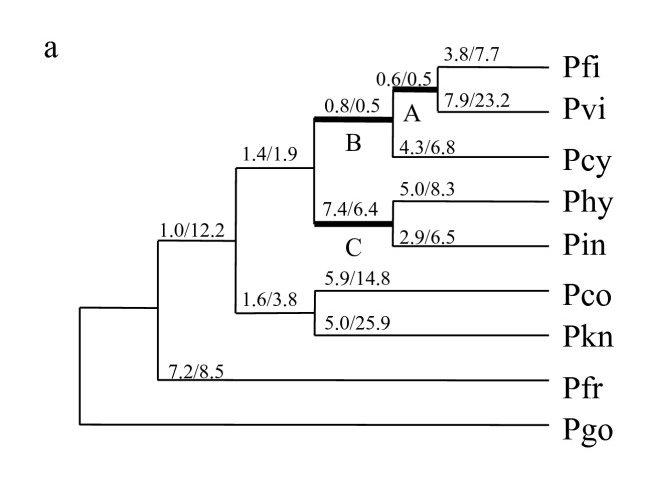

Supplement: Additional file 6 — ZIP files containing several folders, each of which with TreeSnatcher Plus snapshot files, the original image and a text file. [file 1471-2105-13-110-S6.zip › 1471-2148-10-52-2/1471-2148-10-52-2-l_o.PNG]

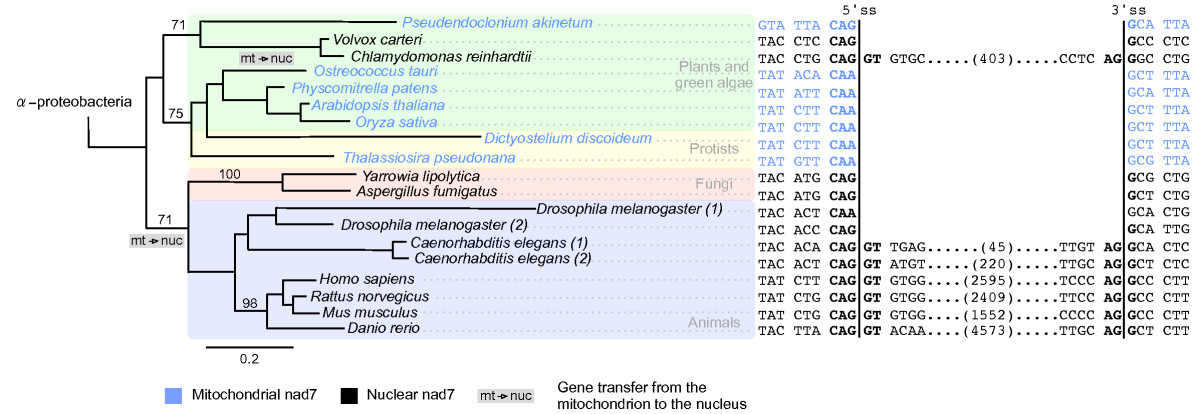

Supplement: Additional file 6 — ZIP files containing several folders, each of which with TreeSnatcher Plus snapshot files, the original image and a text file. [file 1471-2105-13-110-S6.zip › 1471-2148-10-57-4/1471-2148-10-57-4-l.jpg]

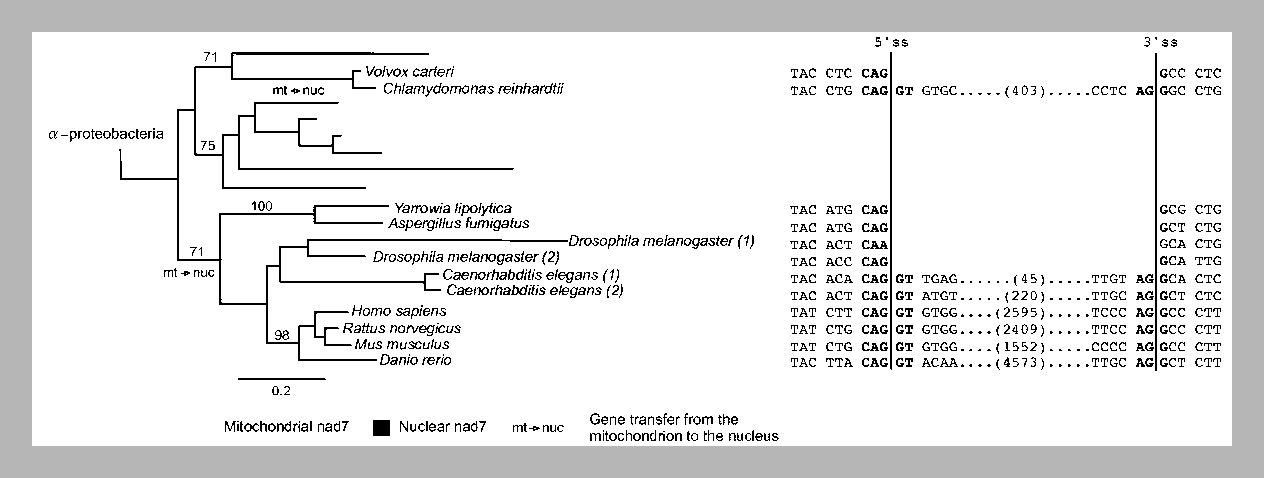

Supplement: Additional file 6 — ZIP files containing several folders, each of which with TreeSnatcher Plus snapshot files, the original image and a text file. [file 1471-2105-13-110-S6.zip › 1471-2148-10-57-4/1471-2148-10-57-4-l_b.PNG]

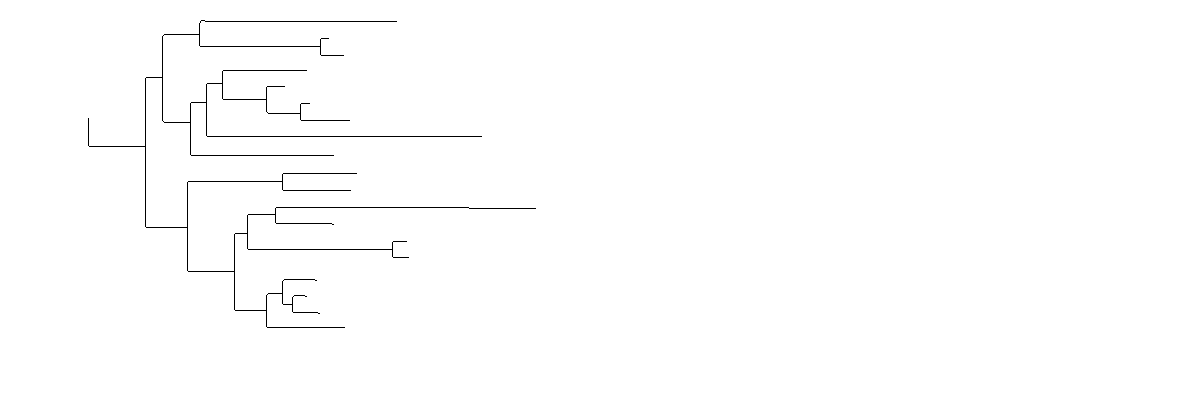

Supplement: Additional file 6 — ZIP files containing several folders, each of which with TreeSnatcher Plus snapshot files, the original image and a text file. [file 1471-2105-13-110-S6.zip › 1471-2148-10-57-4/1471-2148-10-57-4-l_c.PNG]

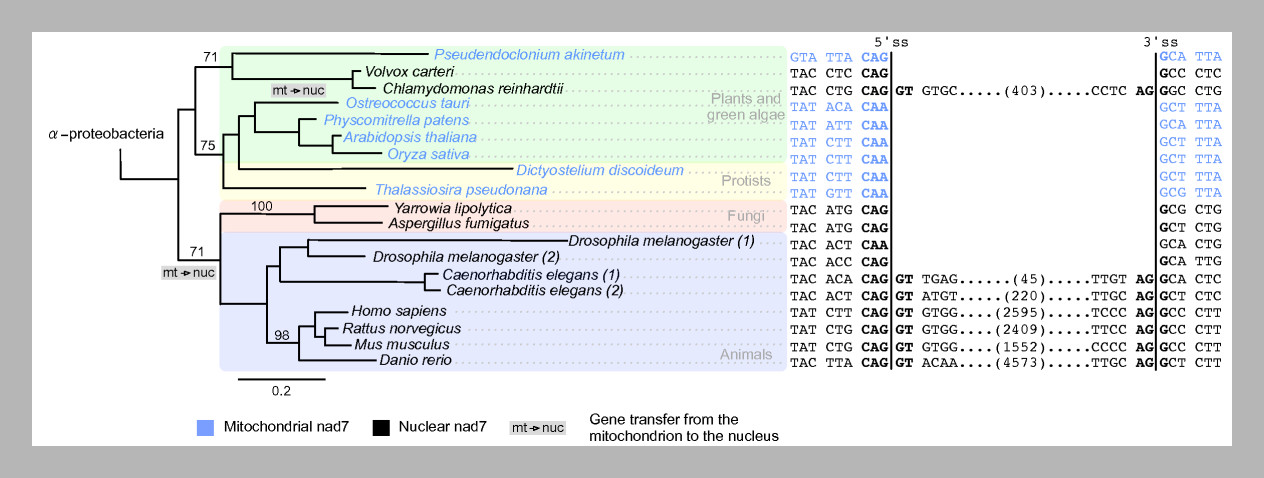

Supplement: Additional file 6 — ZIP files containing several folders, each of which with TreeSnatcher Plus snapshot files, the original image and a text file. [file 1471-2105-13-110-S6.zip › 1471-2148-10-57-4/1471-2148-10-57-4-l_o.PNG]

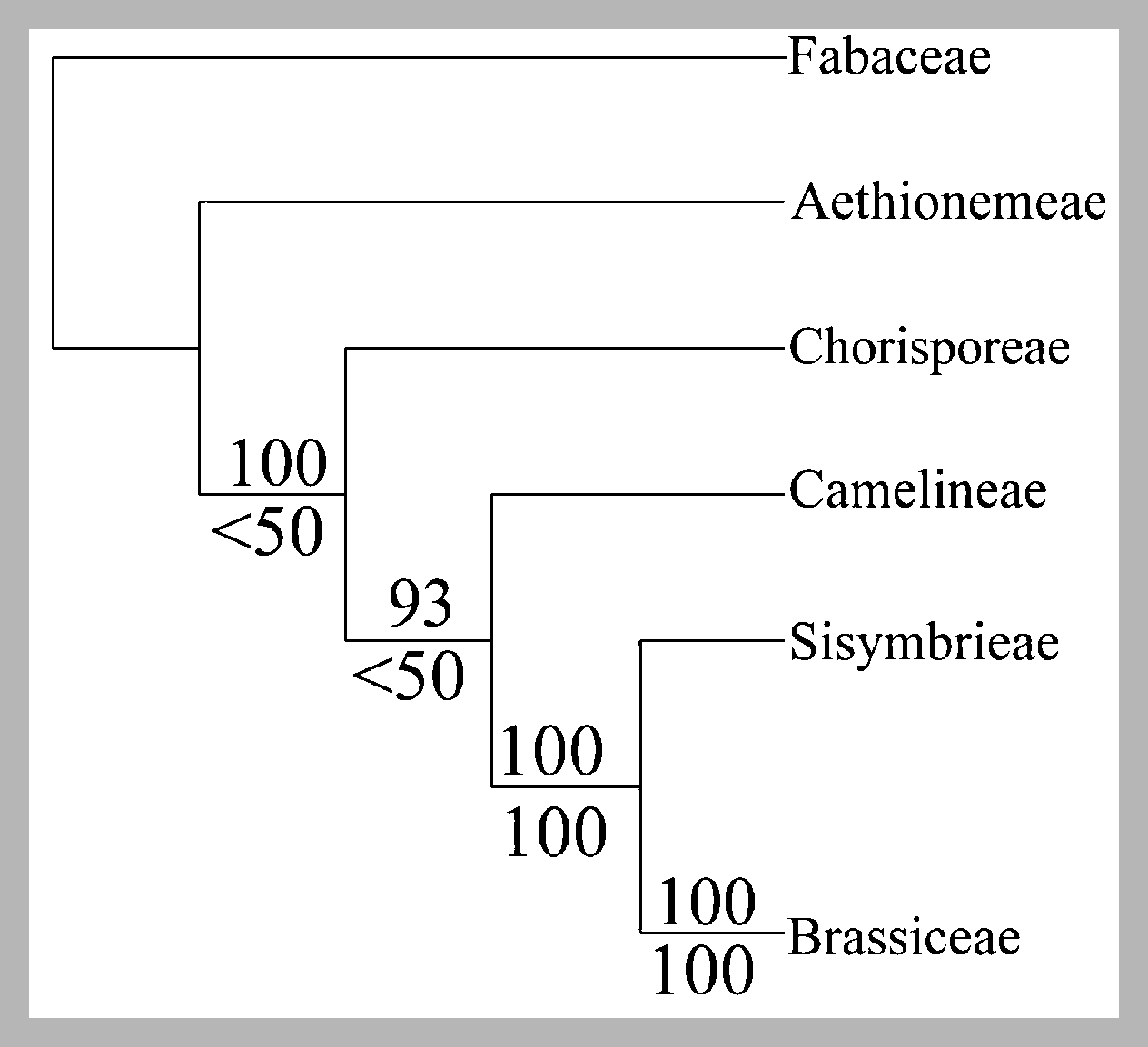

Supplement: Additional file 6 — ZIP files containing several folders, each of which with TreeSnatcher Plus snapshot files, the original image and a text file. [file 1471-2105-13-110-S6.zip › 1471-2148-10-61-4/1471-2148-10-61-4-l_b.PNG]

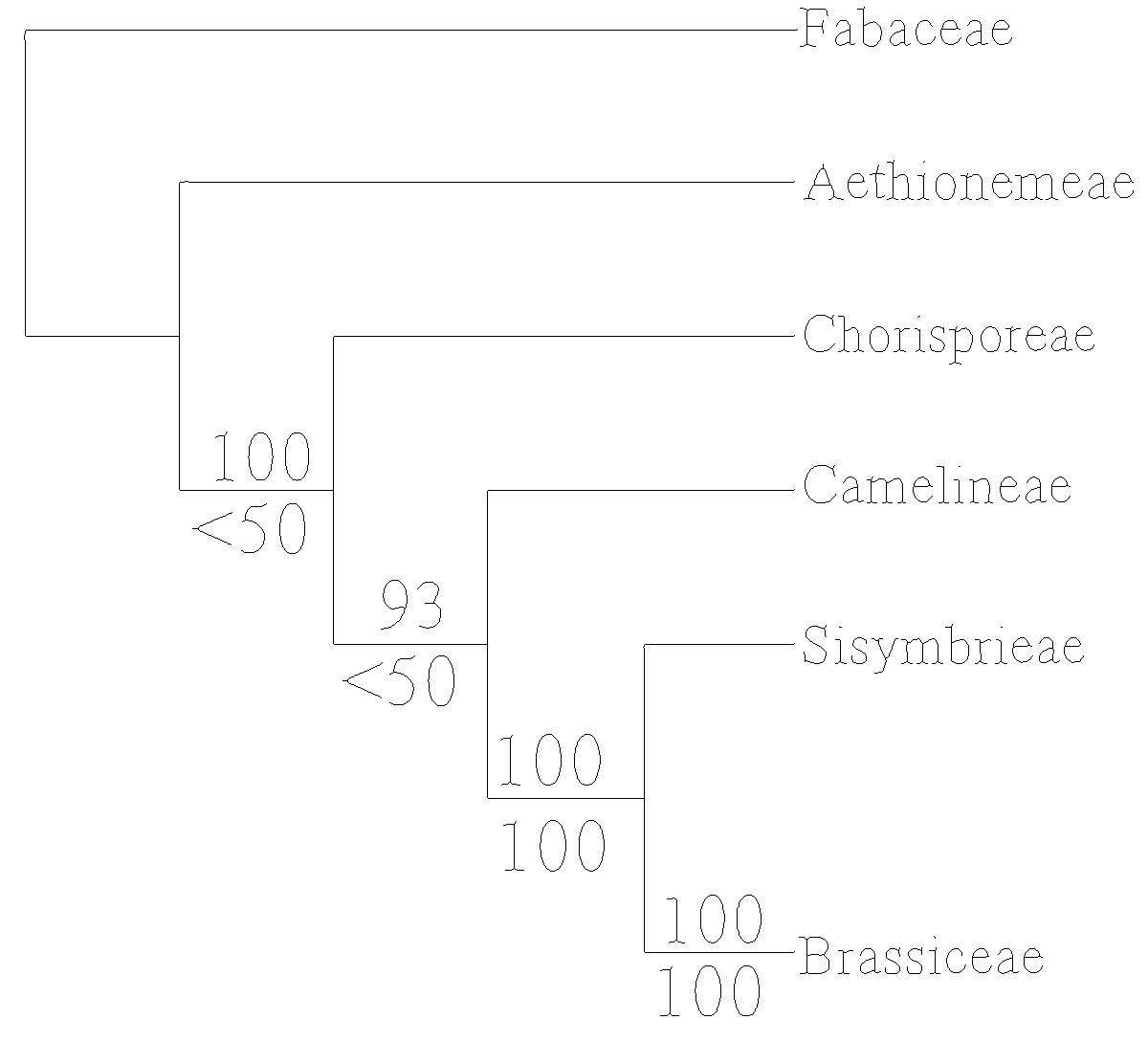

Supplement: Additional file 6 — ZIP files containing several folders, each of which with TreeSnatcher Plus snapshot files, the original image and a text file. [file 1471-2105-13-110-S6.zip › 1471-2148-10-61-4/1471-2148-10-61-4-l_c.PNG]

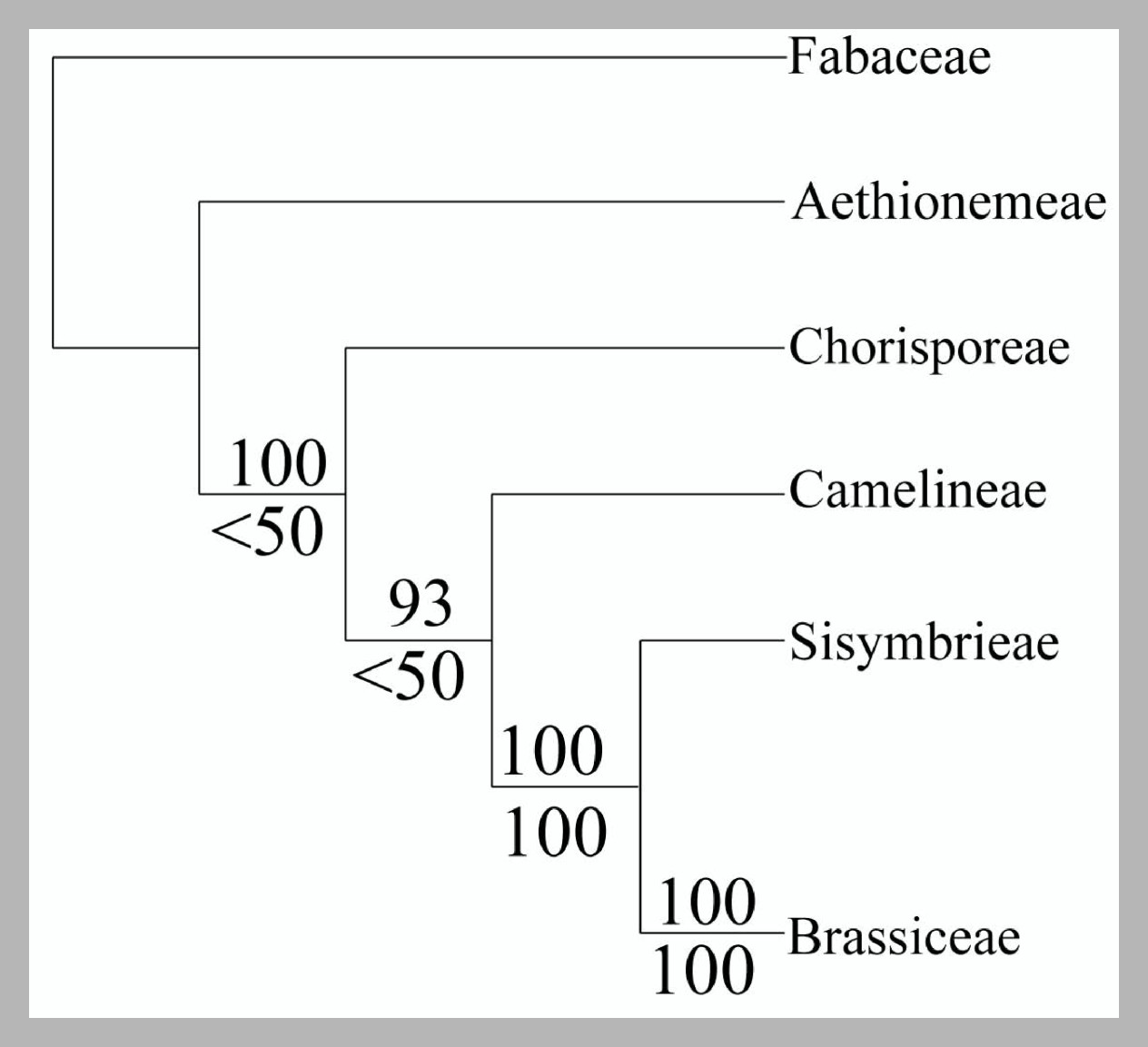

Supplement: Additional file 6 — ZIP files containing several folders, each of which with TreeSnatcher Plus snapshot files, the original image and a text file. [file 1471-2105-13-110-S6.zip › 1471-2148-10-61-4/1471-2148-10-61-4-l_o.PNG]

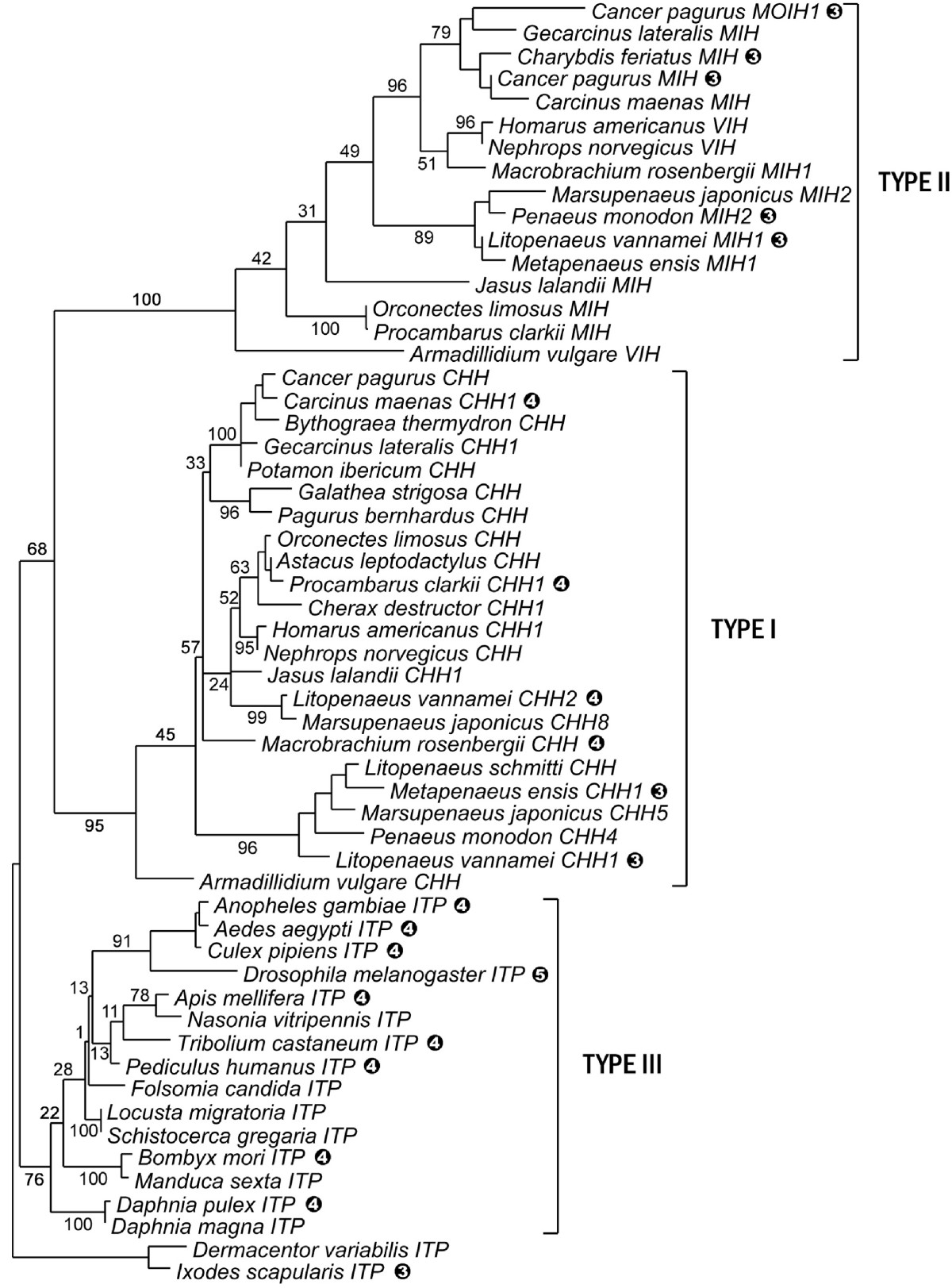

Supplement: Additional file 6 — ZIP files containing several folders, each of which with TreeSnatcher Plus snapshot files, the original image and a text file. [file 1471-2105-13-110-S6.zip › 1471-2148-10-62-5/1471-2148-10-62-5-l.jpg]

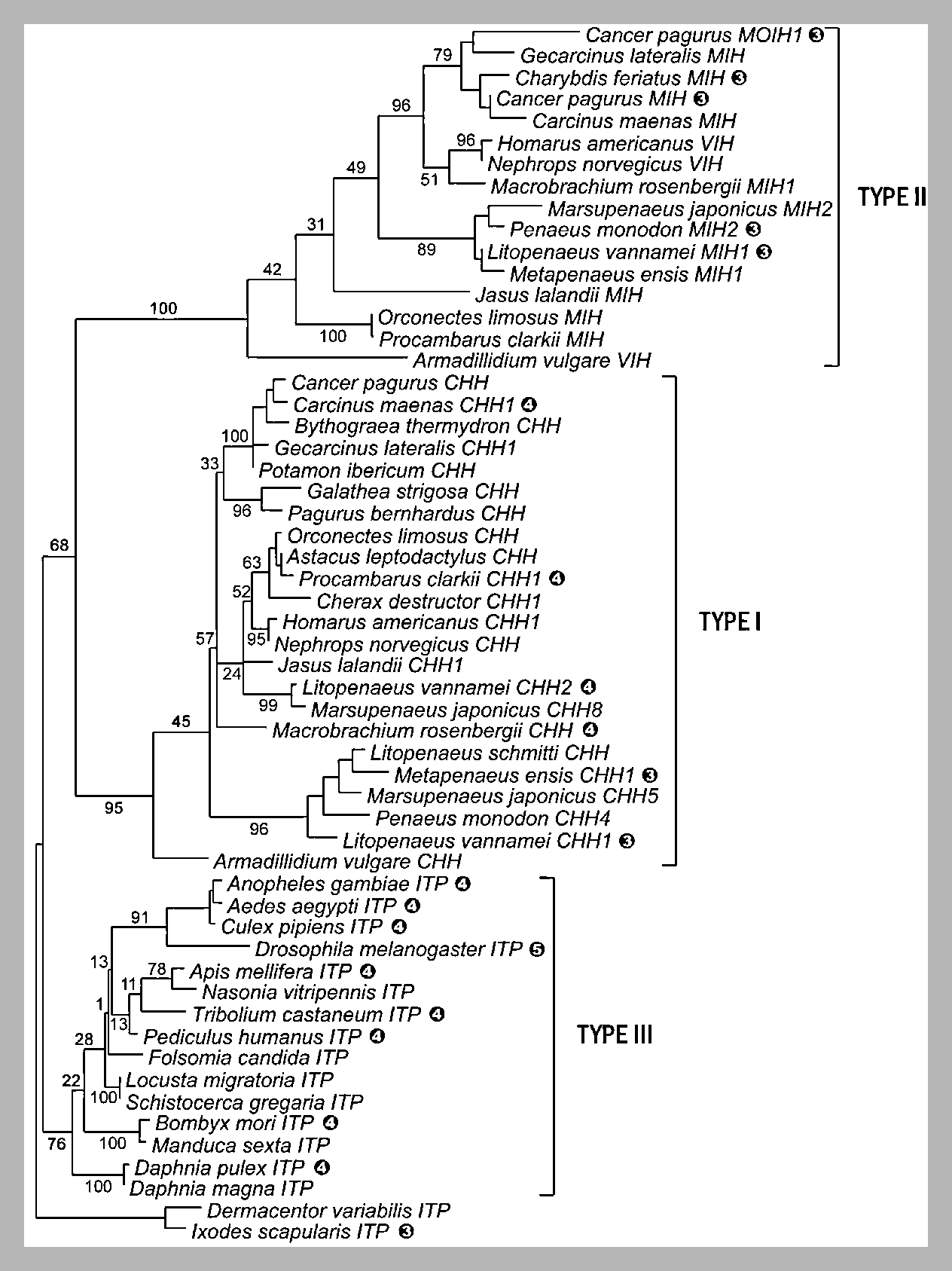

Supplement: Additional file 6 — ZIP files containing several folders, each of which with TreeSnatcher Plus snapshot files, the original image and a text file. [file 1471-2105-13-110-S6.zip › 1471-2148-10-62-5/1471-2148-10-62-5-l_b.PNG]

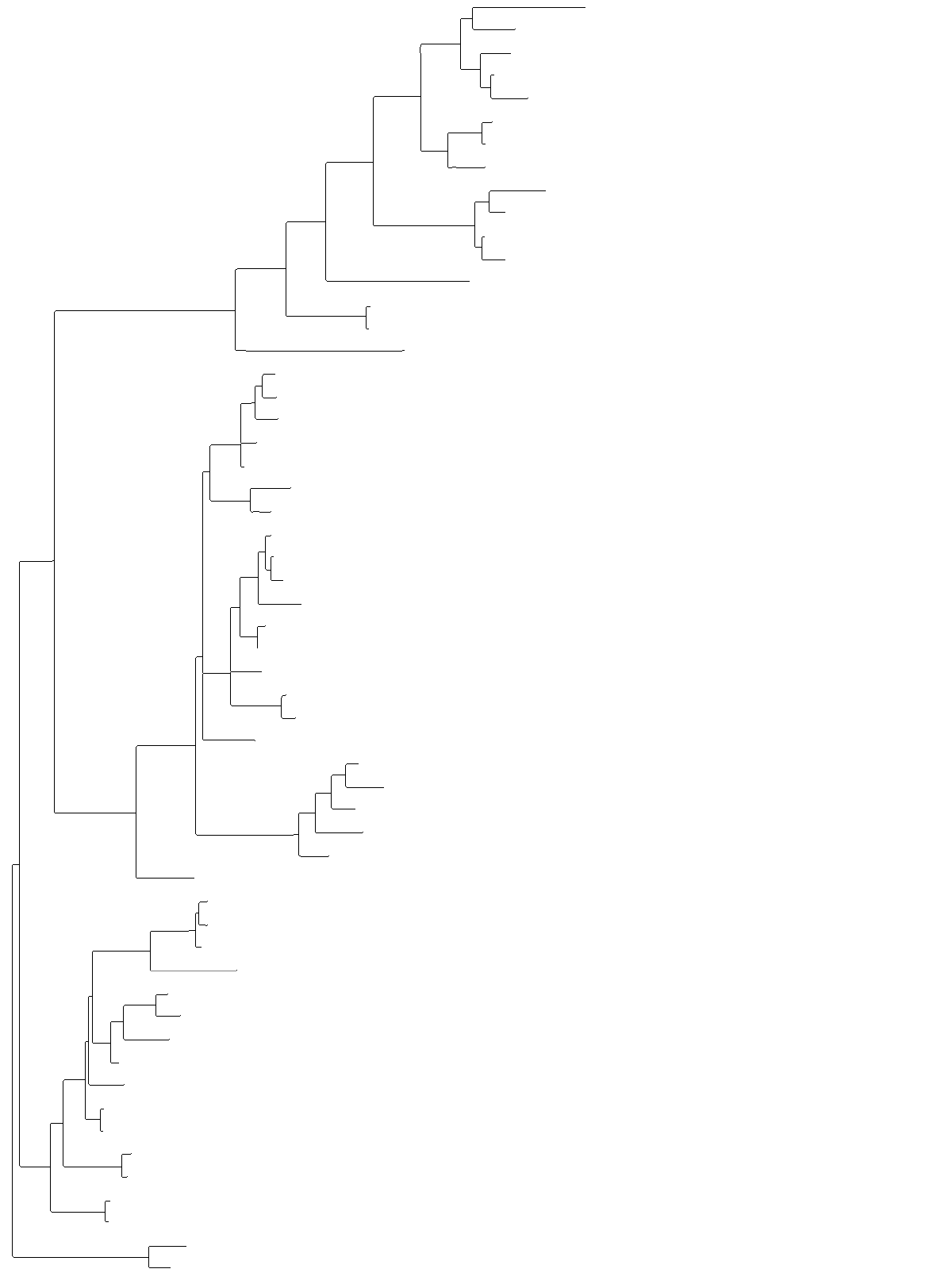

Supplement: Additional file 6 — ZIP files containing several folders, each of which with TreeSnatcher Plus snapshot files, the original image and a text file. [file 1471-2105-13-110-S6.zip › 1471-2148-10-62-5/1471-2148-10-62-5-l_c.PNG]

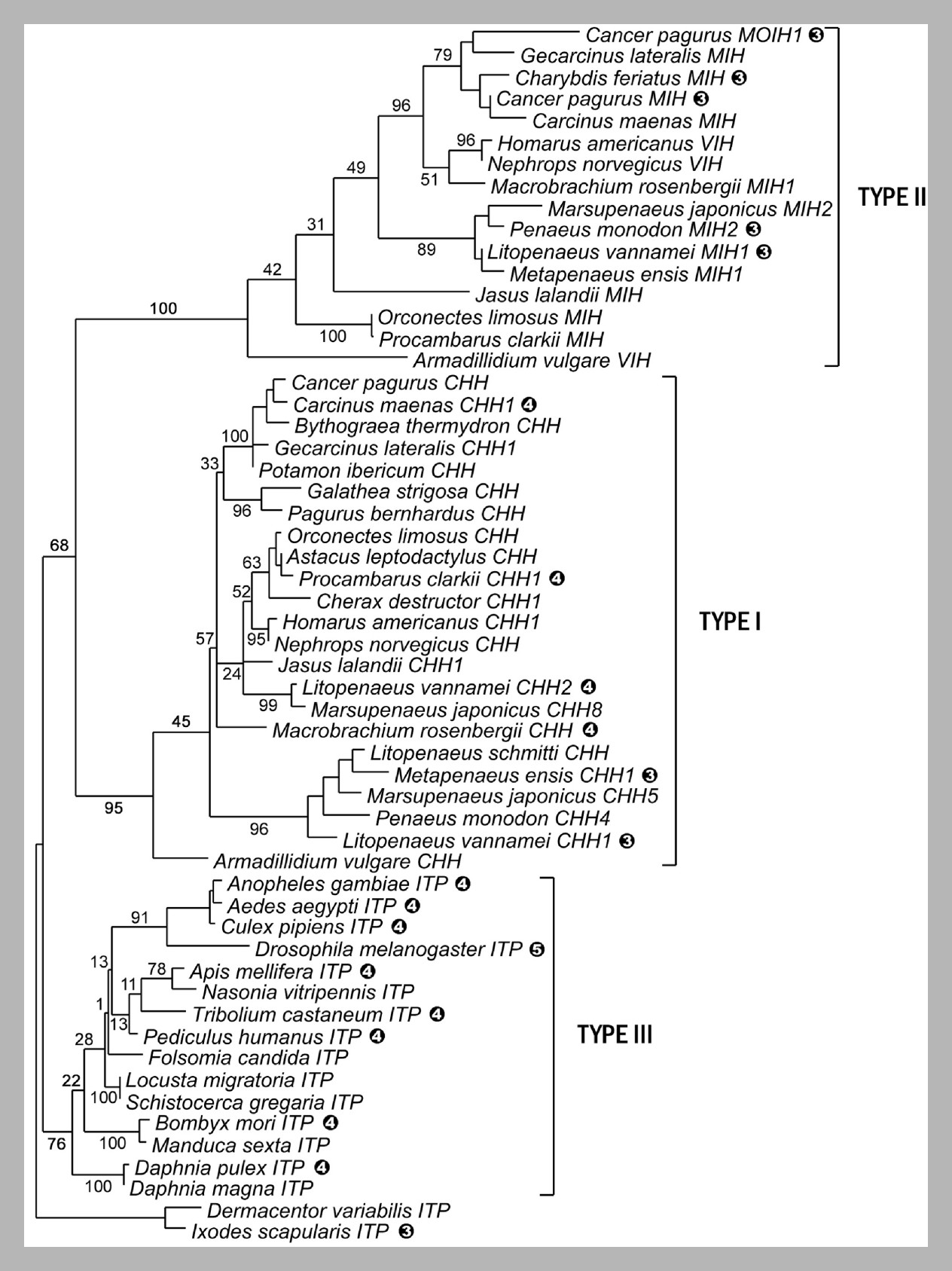

Supplement: Additional file 6 — ZIP files containing several folders, each of which with TreeSnatcher Plus snapshot files, the original image and a text file. [file 1471-2105-13-110-S6.zip › 1471-2148-10-62-5/1471-2148-10-62-5-l_o.PNG]

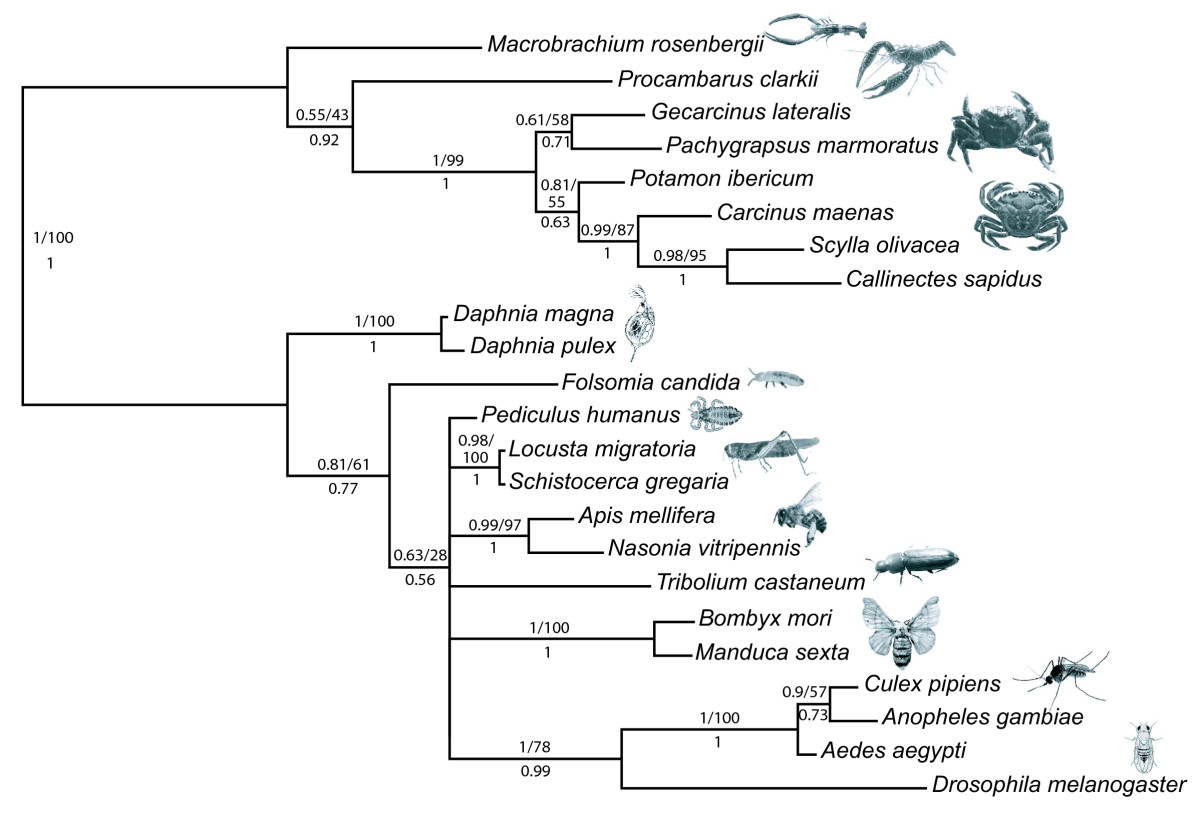

Supplement: Additional file 6 — ZIP files containing several folders, each of which with TreeSnatcher Plus snapshot files, the original image and a text file. [file 1471-2105-13-110-S6.zip › 1471-2148-10-62-6/1471-2148-10-62-6-l.jpg]

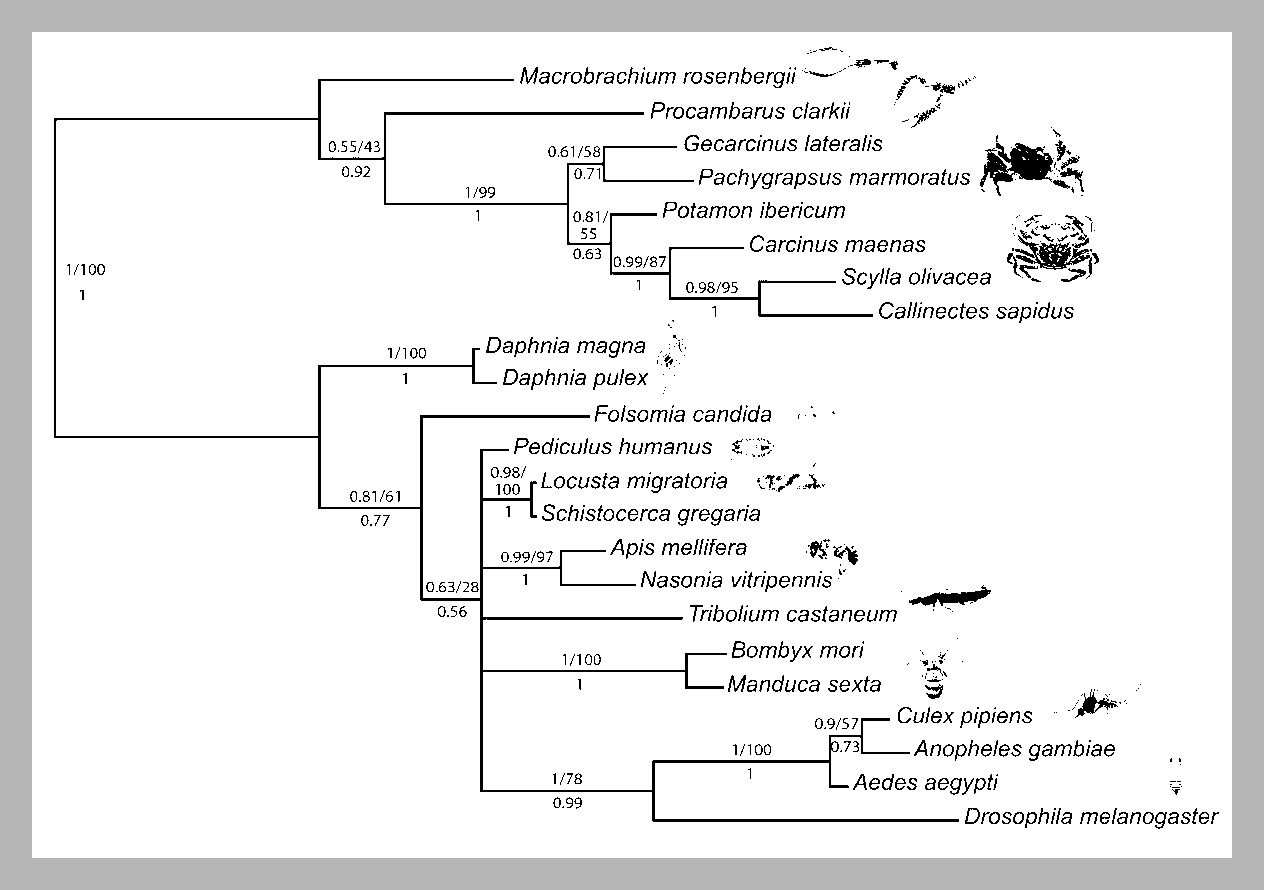

Supplement: Additional file 6 — ZIP files containing several folders, each of which with TreeSnatcher Plus snapshot files, the original image and a text file. [file 1471-2105-13-110-S6.zip › 1471-2148-10-62-6/1471-2148-10-62-6-l_b.PNG]

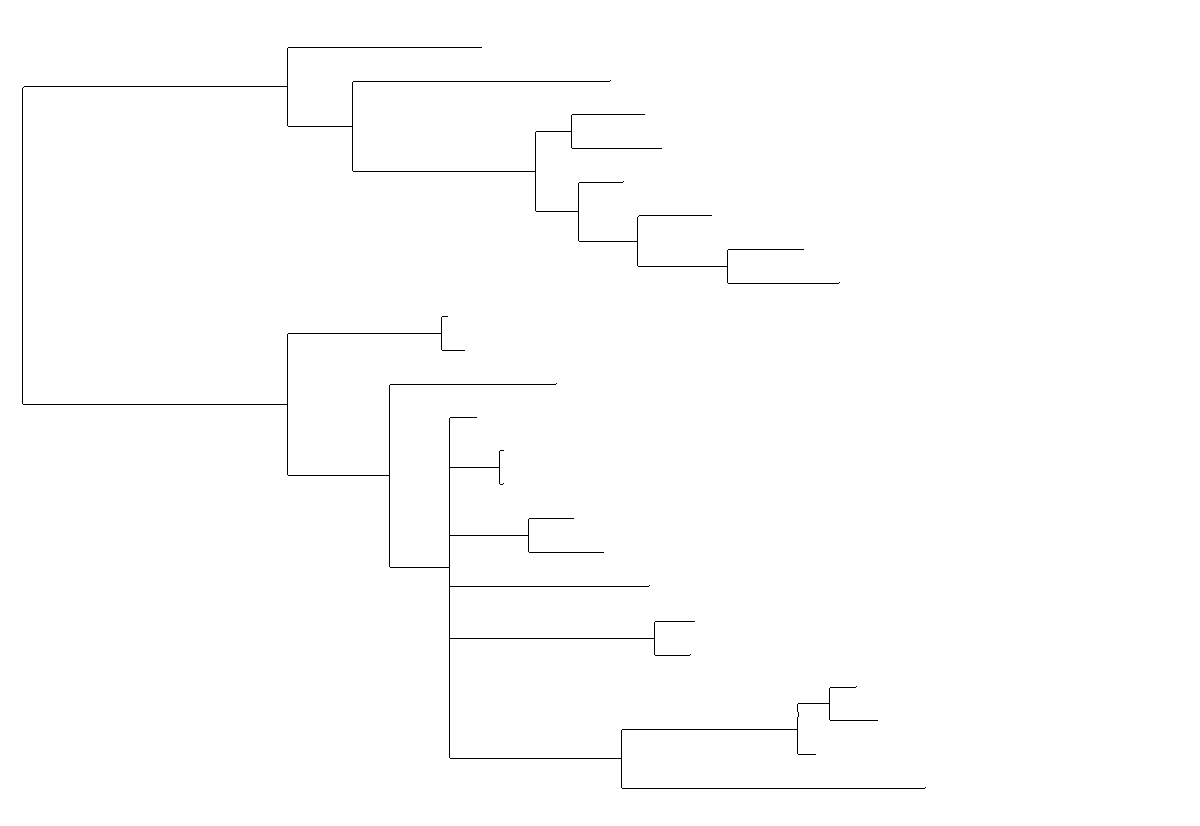

Supplement: Additional file 6 — ZIP files containing several folders, each of which with TreeSnatcher Plus snapshot files, the original image and a text file. [file 1471-2105-13-110-S6.zip › 1471-2148-10-62-6/1471-2148-10-62-6-l_c.PNG]

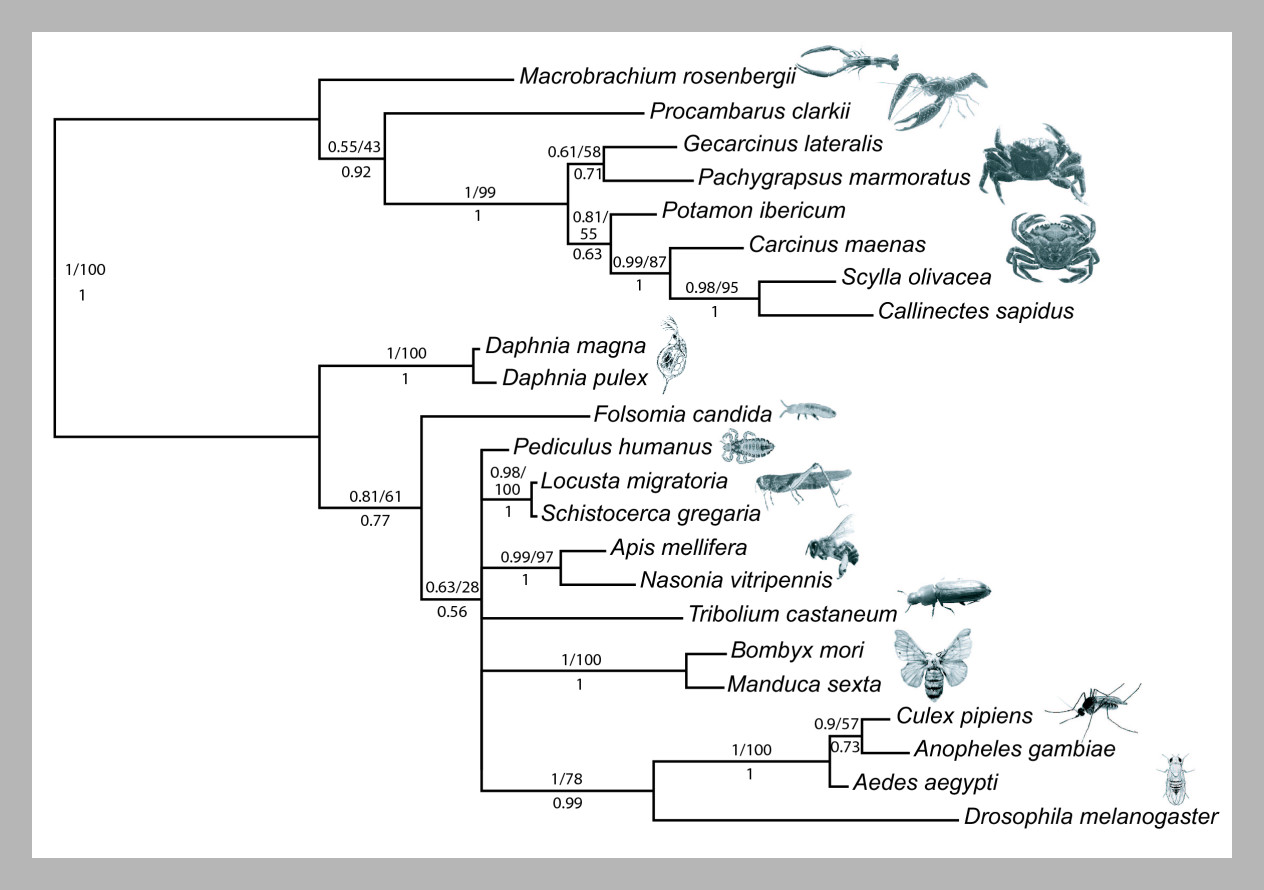

Supplement: Additional file 6 — ZIP files containing several folders, each of which with TreeSnatcher Plus snapshot files, the original image and a text file. [file 1471-2105-13-110-S6.zip › 1471-2148-10-62-6/1471-2148-10-62-6-l_o.PNG]

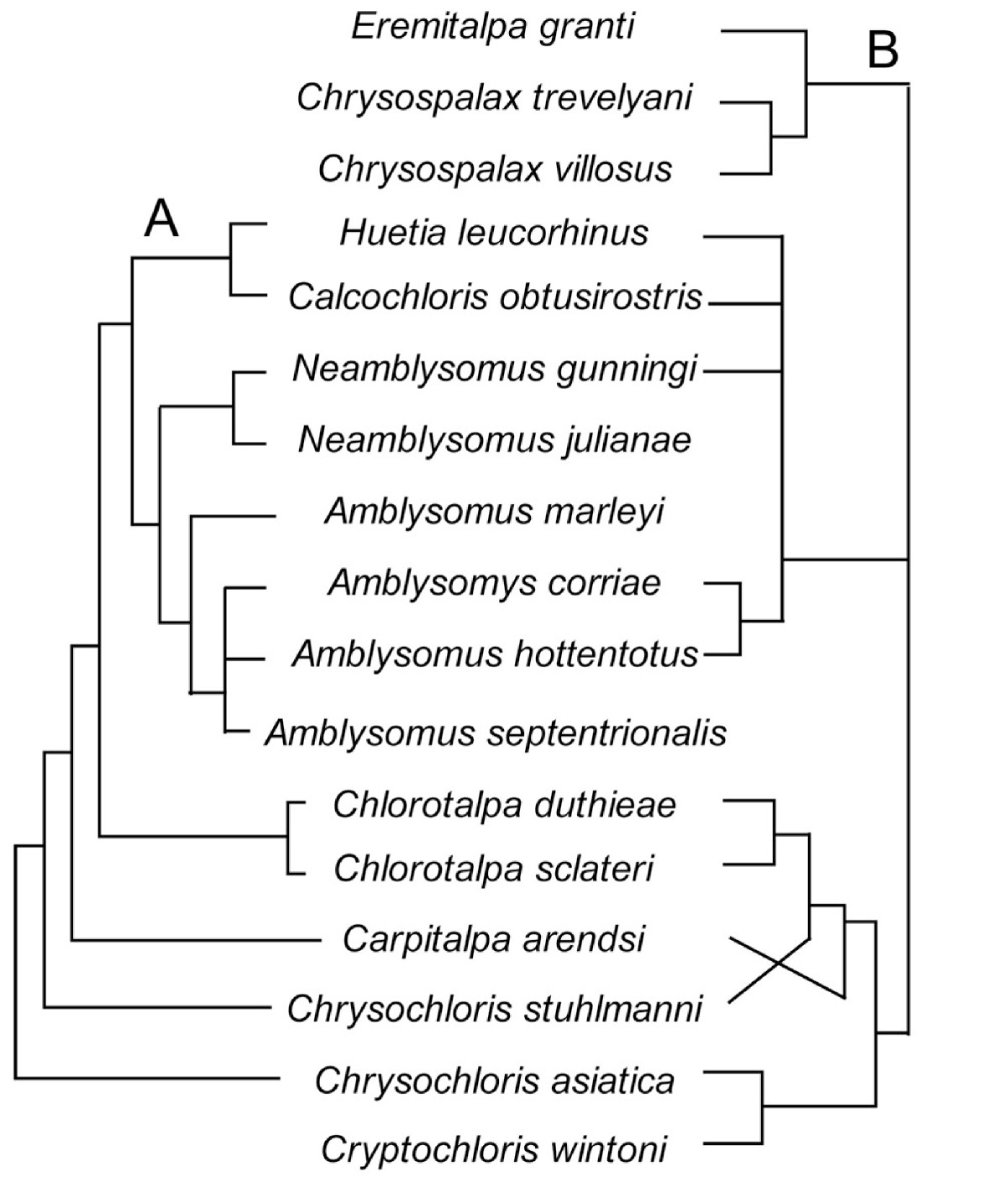

Supplement: Additional file 6 — ZIP files containing several folders, each of which with TreeSnatcher Plus snapshot files, the original image and a text file. [file 1471-2105-13-110-S6.zip › 1471-2148-10-69-1/1471-2148-10-69-1-l.jpg]

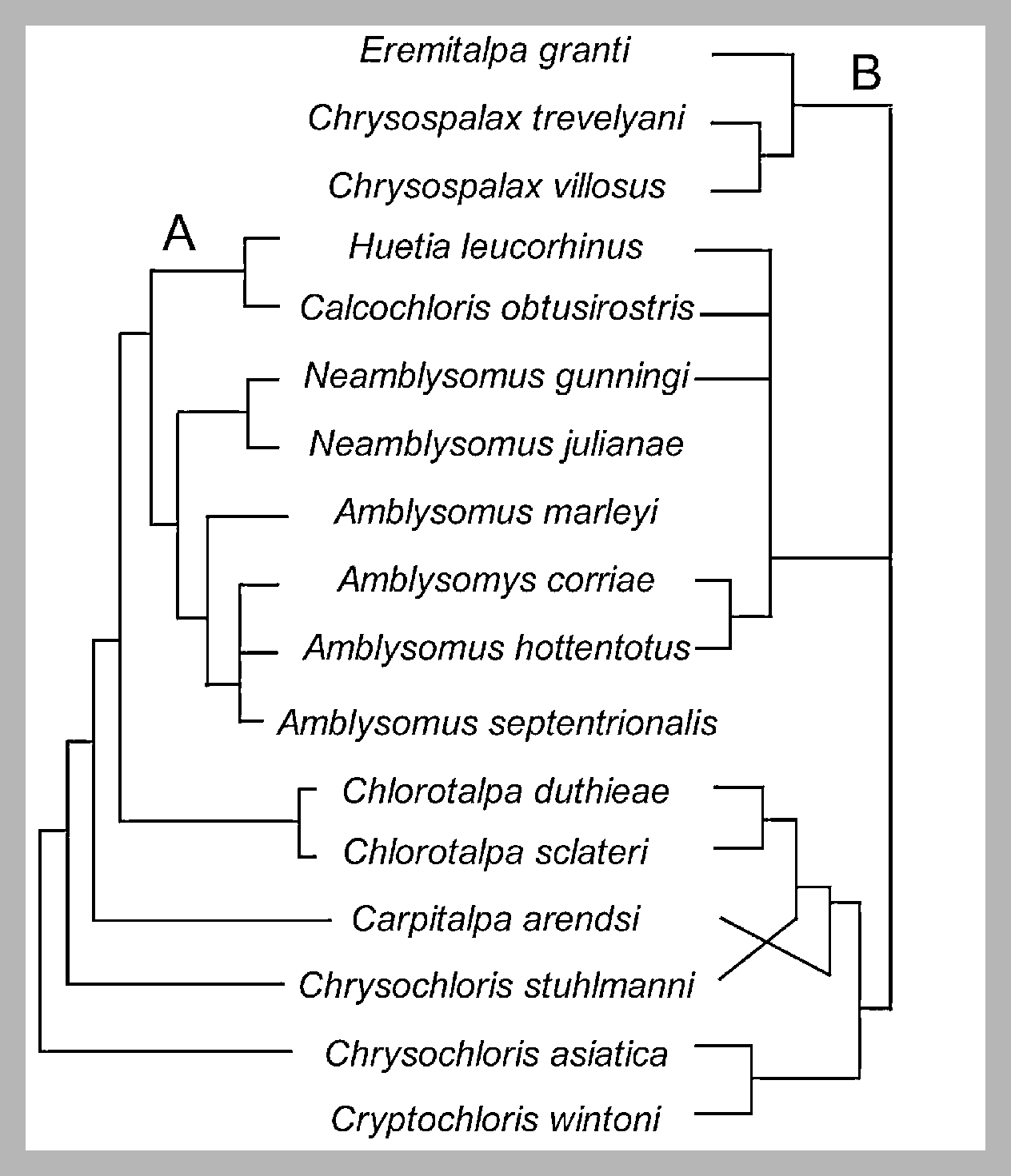

Supplement: Additional file 6 — ZIP files containing several folders, each of which with TreeSnatcher Plus snapshot files, the original image and a text file. [file 1471-2105-13-110-S6.zip › 1471-2148-10-69-1/1471-2148-10-69-1-l_b.PNG]

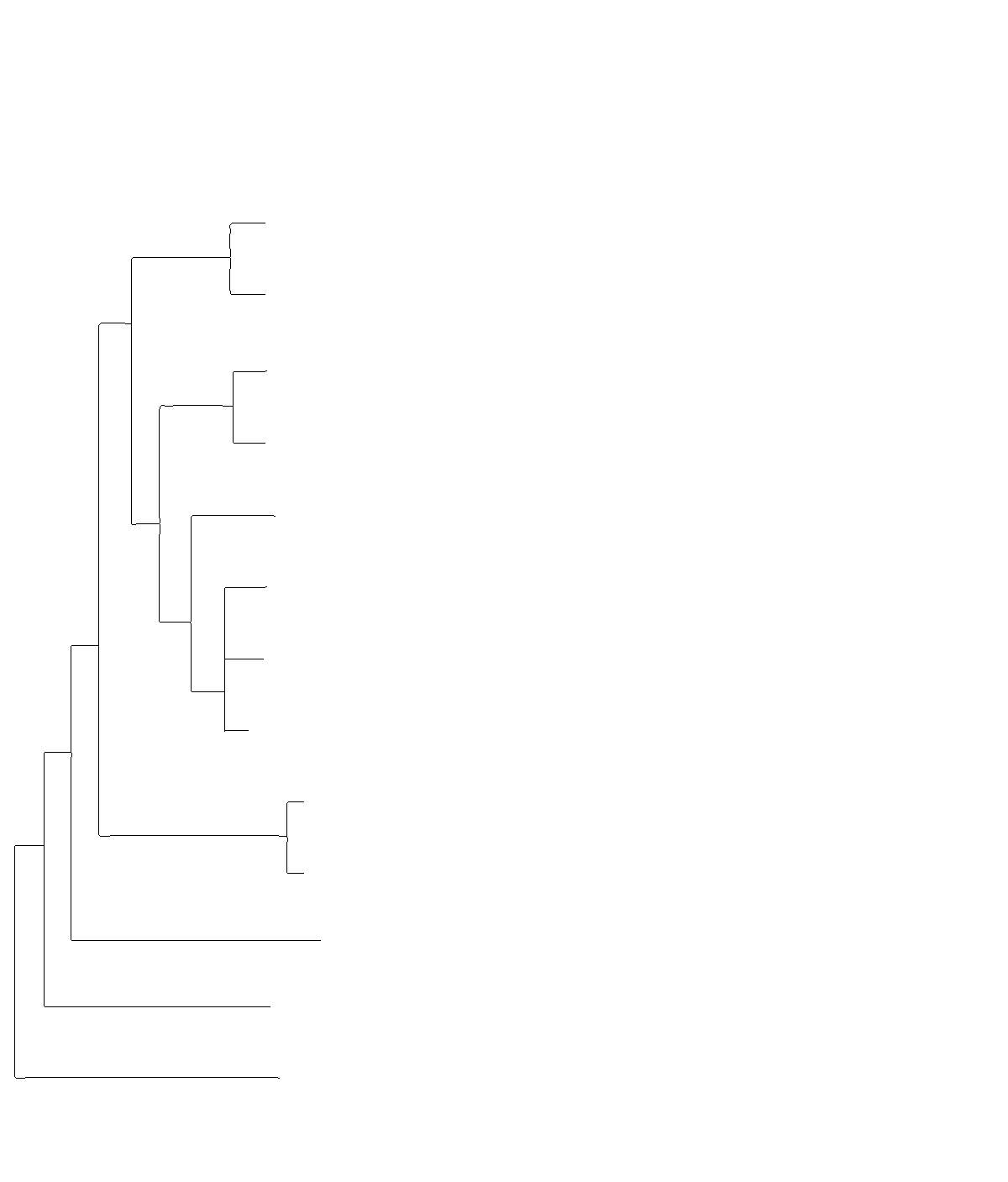

Supplement: Additional file 6 — ZIP files containing several folders, each of which with TreeSnatcher Plus snapshot files, the original image and a text file. [file 1471-2105-13-110-S6.zip › 1471-2148-10-69-1/1471-2148-10-69-1-l_c.PNG]

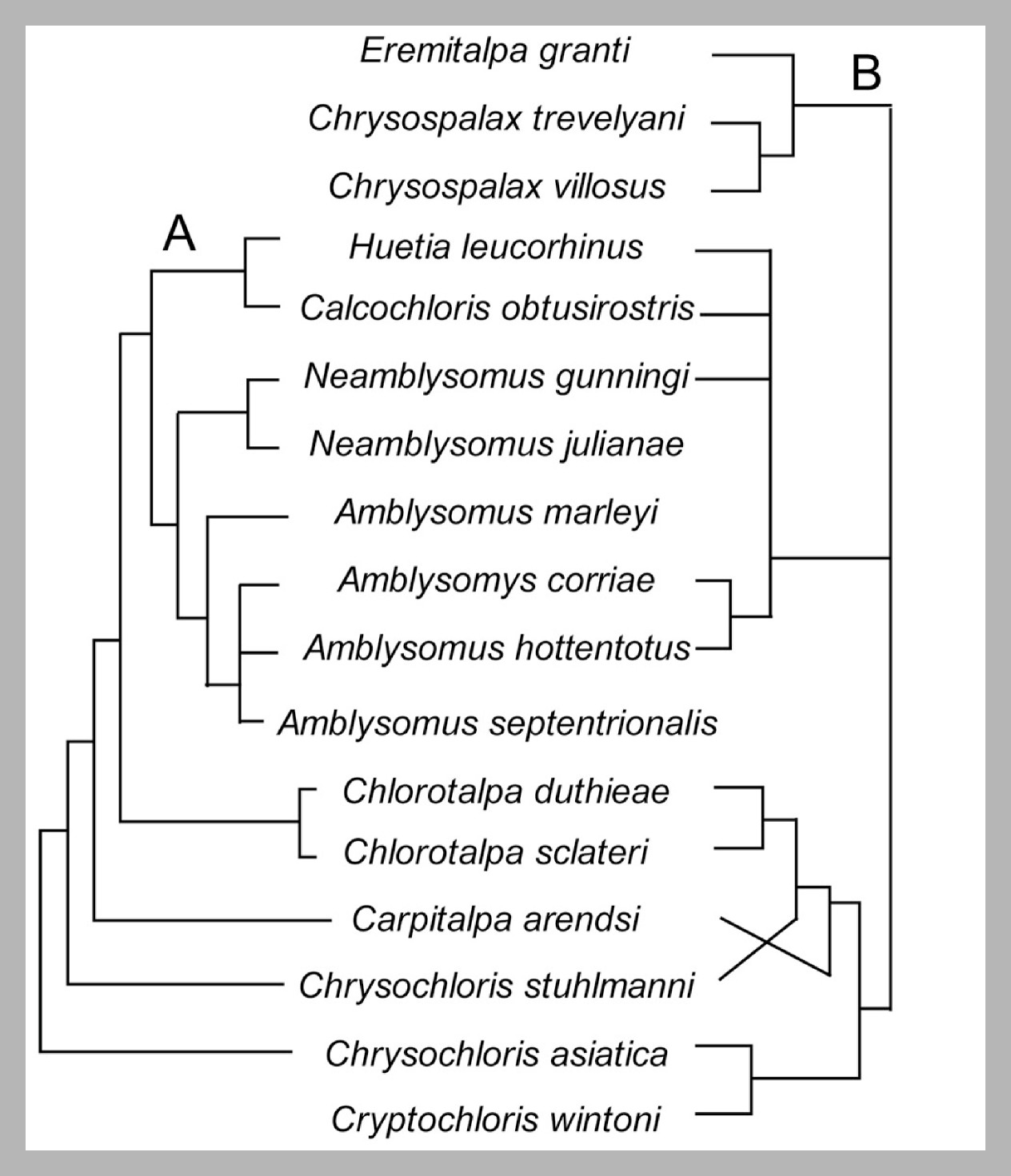

Supplement: Additional file 6 — ZIP files containing several folders, each of which with TreeSnatcher Plus snapshot files, the original image and a text file. [file 1471-2105-13-110-S6.zip › 1471-2148-10-69-1/1471-2148-10-69-1-l_o.PNG]

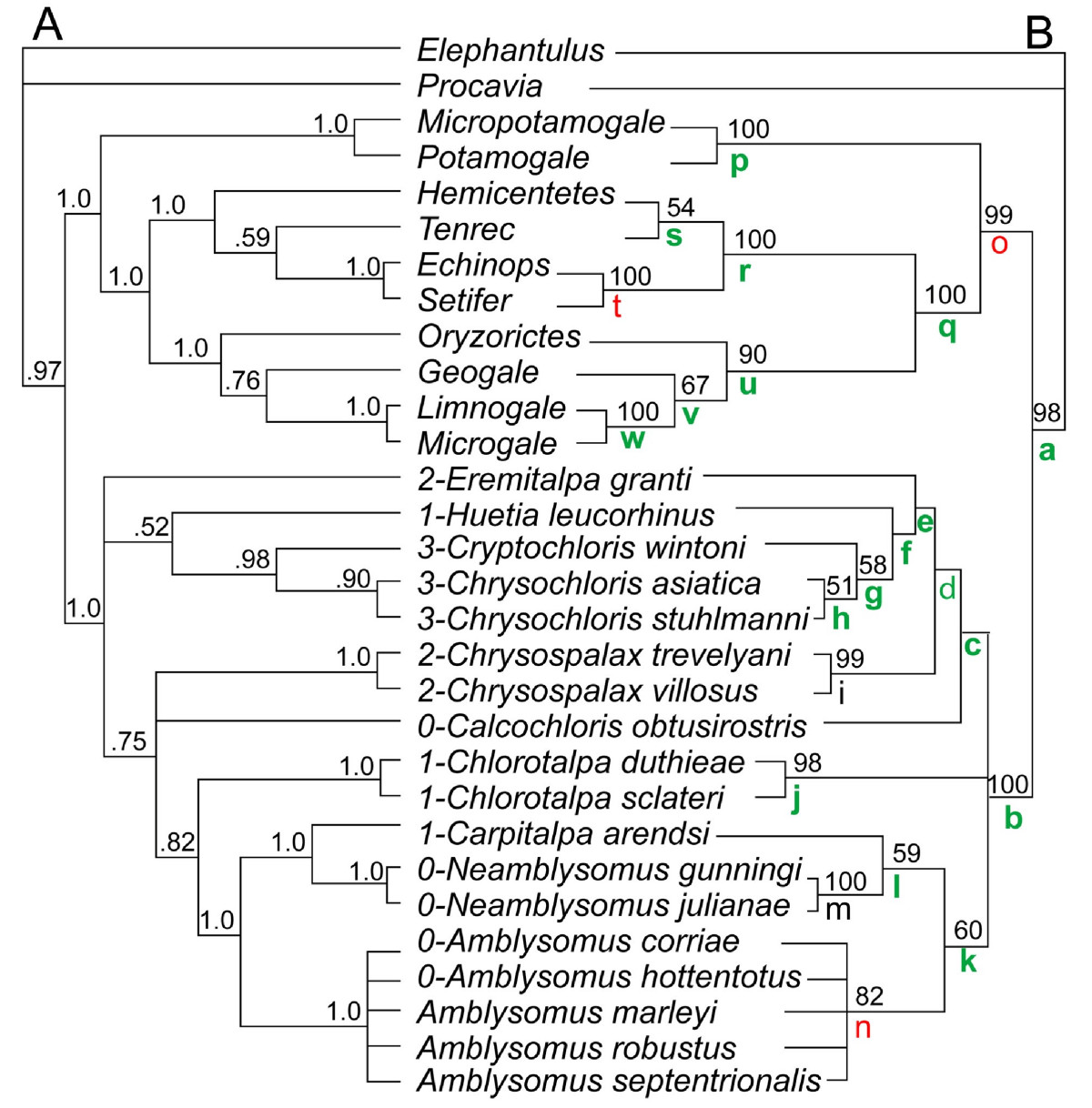

Supplement: Additional file 6 — ZIP files containing several folders, each of which with TreeSnatcher Plus snapshot files, the original image and a text file. [file 1471-2105-13-110-S6.zip › 1471-2148-10-69-2/1471-2148-10-69-2-l.jpg]

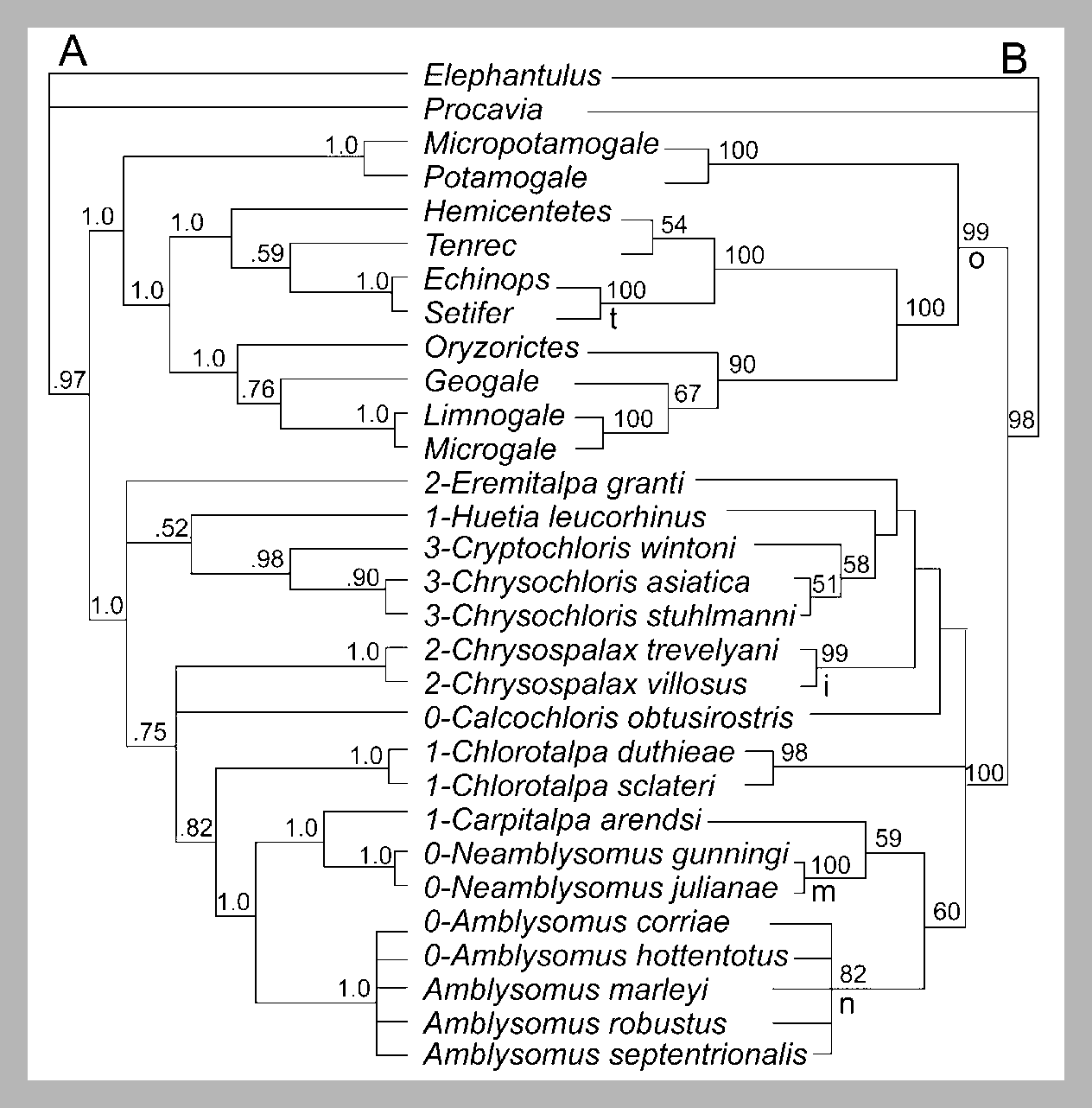

Supplement: Additional file 6 — ZIP files containing several folders, each of which with TreeSnatcher Plus snapshot files, the original image and a text file. [file 1471-2105-13-110-S6.zip › 1471-2148-10-69-2/1471-2148-10-69-2-l_b.PNG]

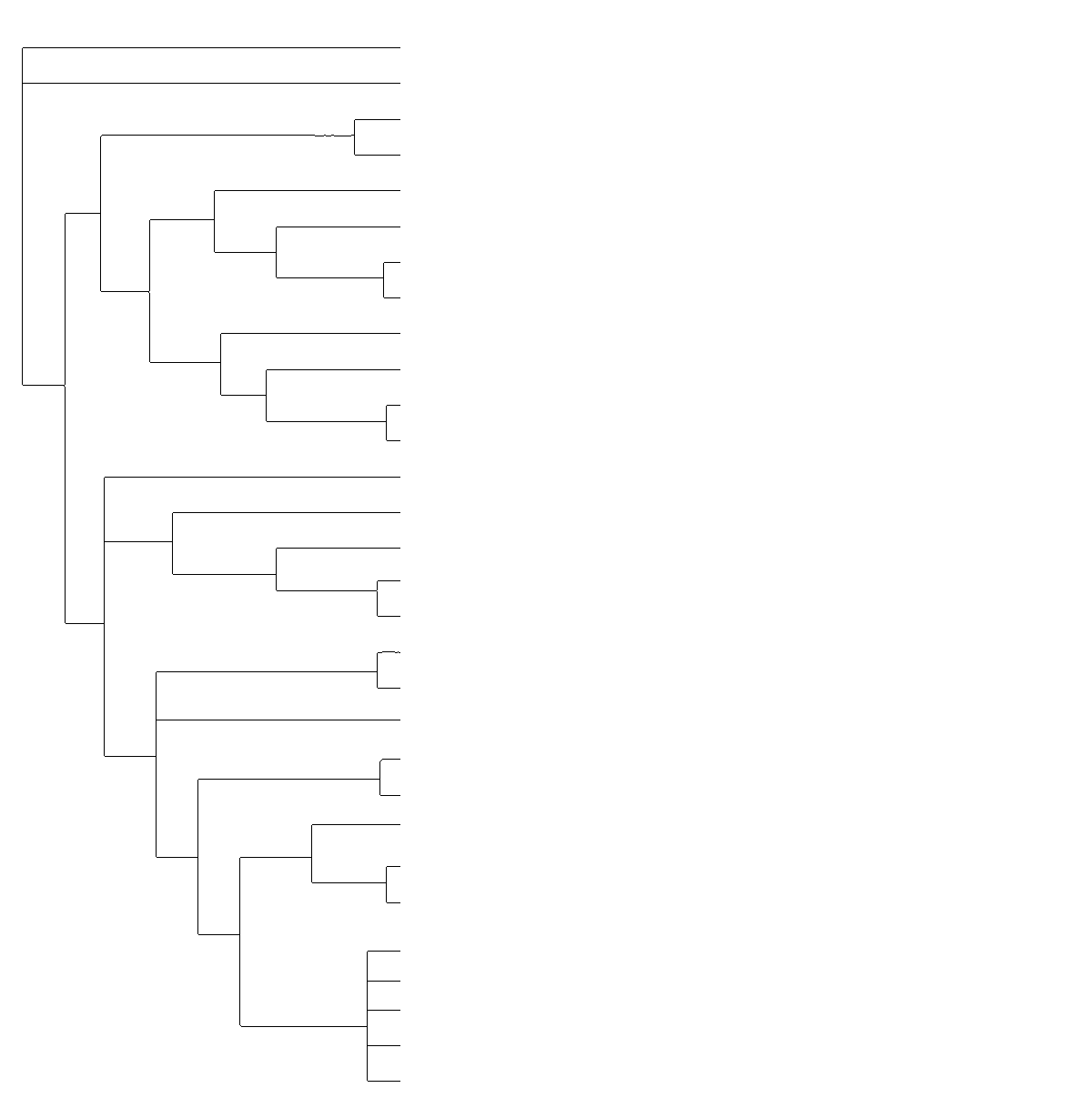

Supplement: Additional file 6 — ZIP files containing several folders, each of which with TreeSnatcher Plus snapshot files, the original image and a text file. [file 1471-2105-13-110-S6.zip › 1471-2148-10-69-2/1471-2148-10-69-2-l_c.PNG]

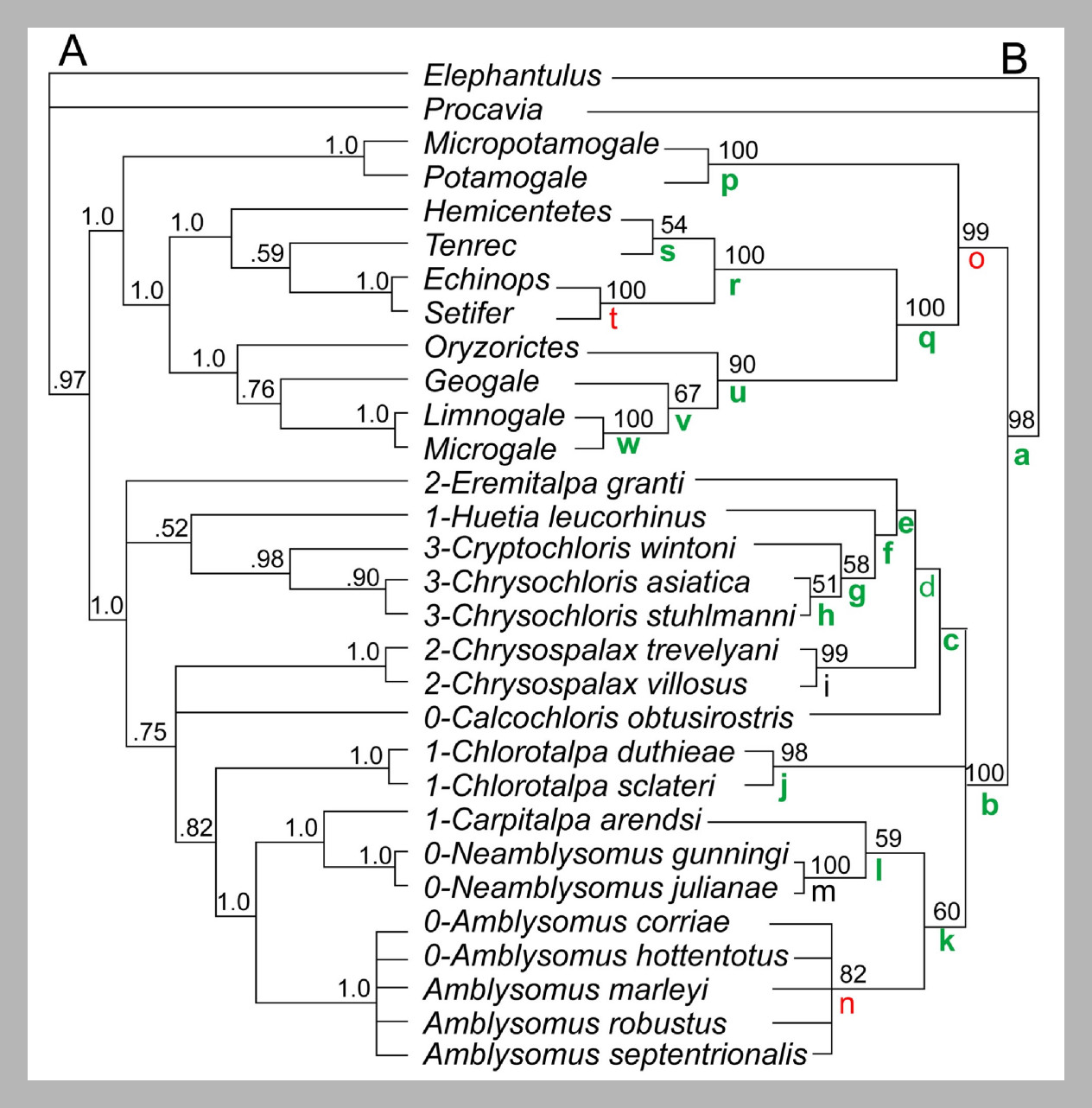

Supplement: Additional file 6 — ZIP files containing several folders, each of which with TreeSnatcher Plus snapshot files, the original image and a text file. [file 1471-2105-13-110-S6.zip › 1471-2148-10-69-2/1471-2148-10-69-2-l_o.PNG]

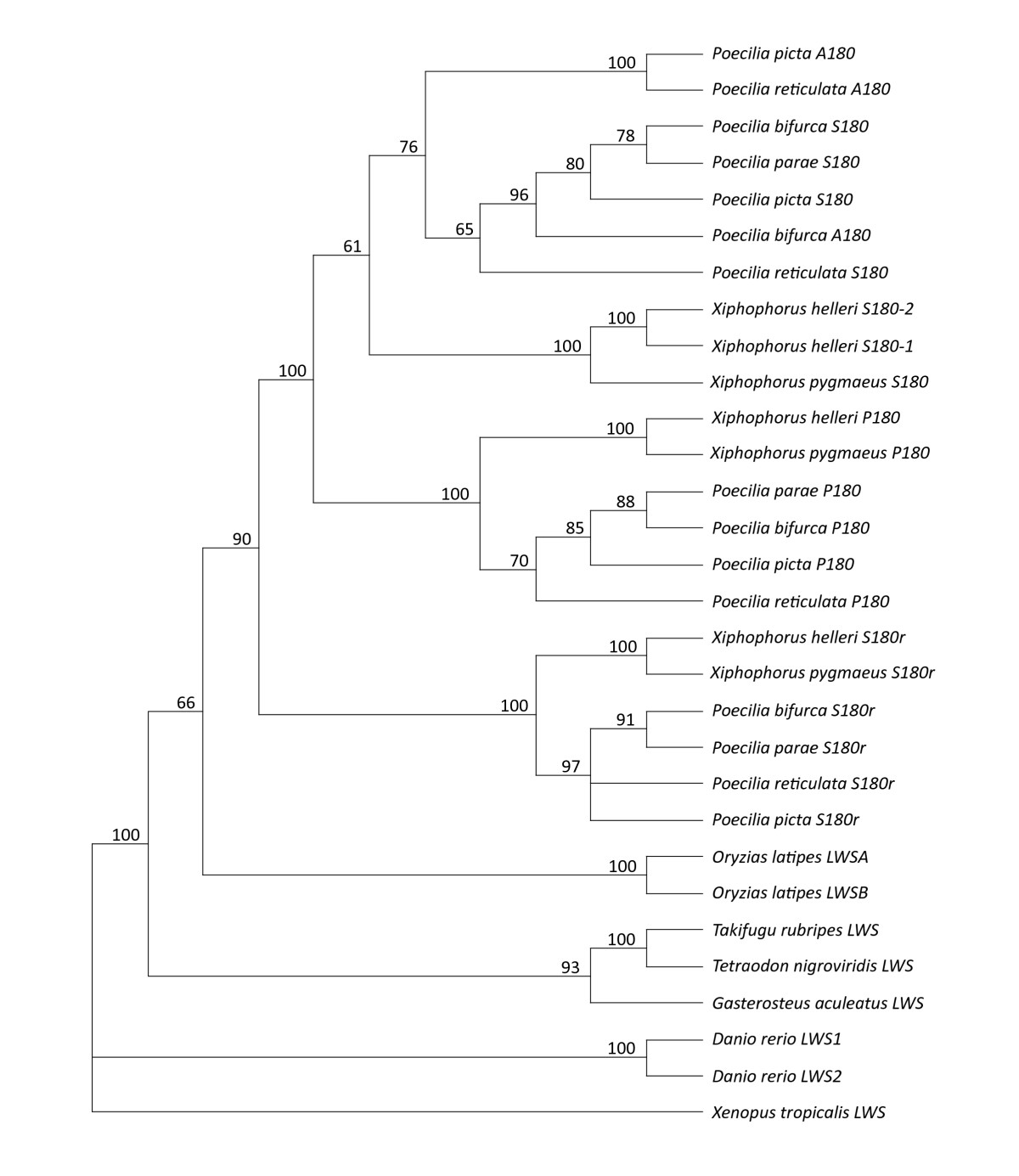

Supplement: Additional file 6 — ZIP files containing several folders, each of which with TreeSnatcher Plus snapshot files, the original image and a text file. [file 1471-2105-13-110-S6.zip › 1471-2148-10-87-3/1471-2148-10-87-3-l.jpg]

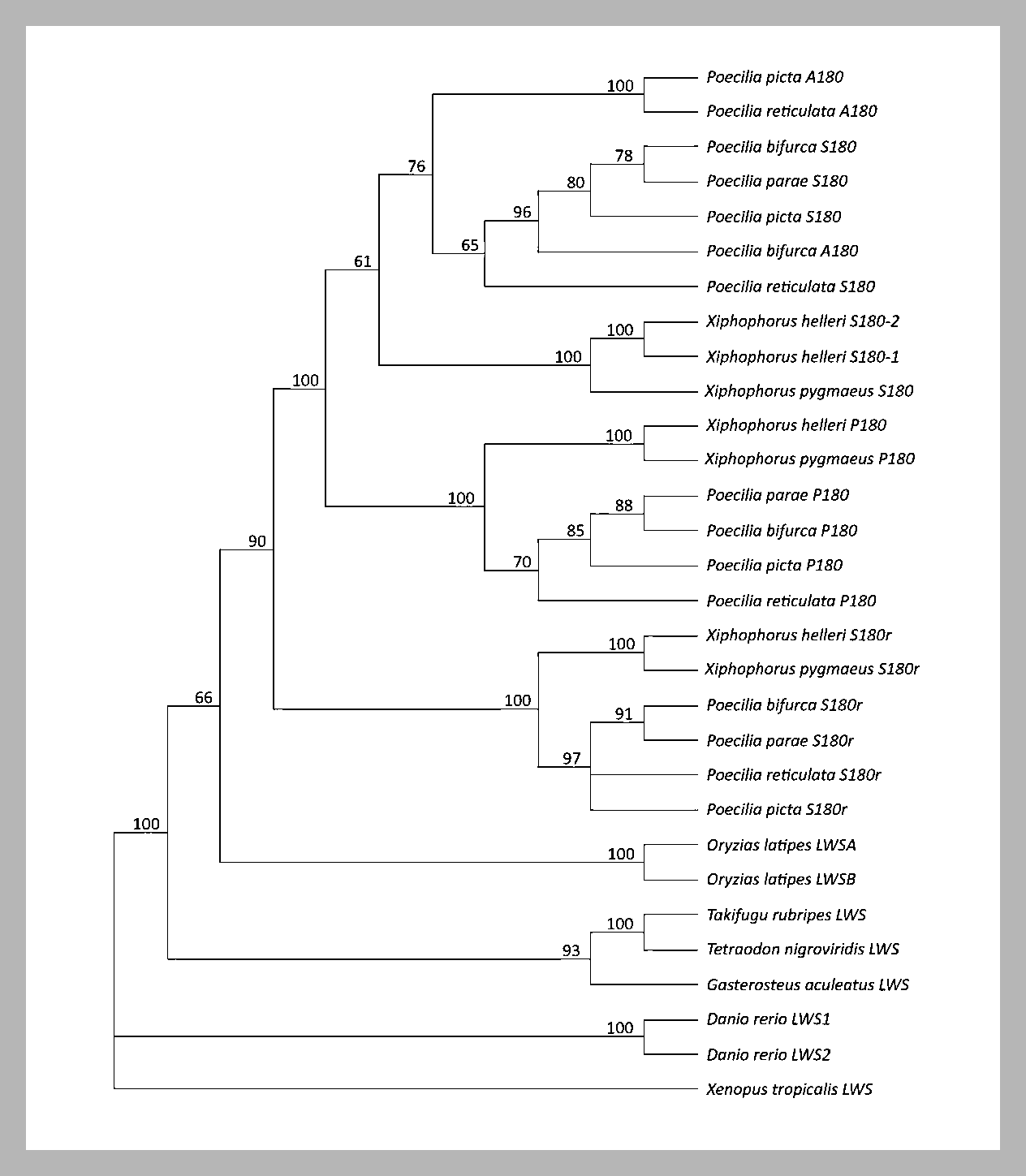

Supplement: Additional file 6 — ZIP files containing several folders, each of which with TreeSnatcher Plus snapshot files, the original image and a text file. [file 1471-2105-13-110-S6.zip › 1471-2148-10-87-3/1471-2148-10-87-3-l_b.PNG]

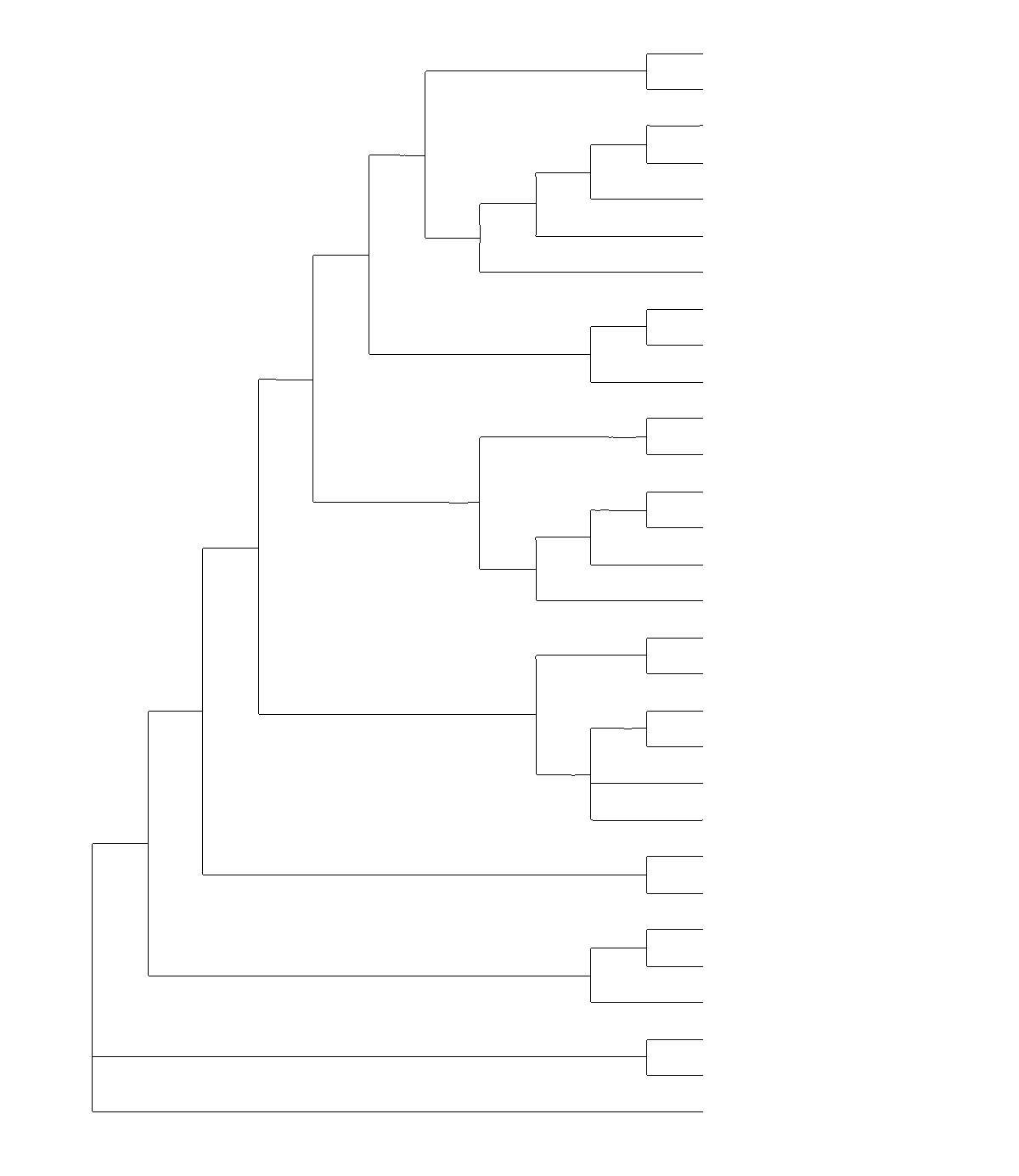

Supplement: Additional file 6 — ZIP files containing several folders, each of which with TreeSnatcher Plus snapshot files, the original image and a text file. [file 1471-2105-13-110-S6.zip › 1471-2148-10-87-3/1471-2148-10-87-3-l_c.PNG]

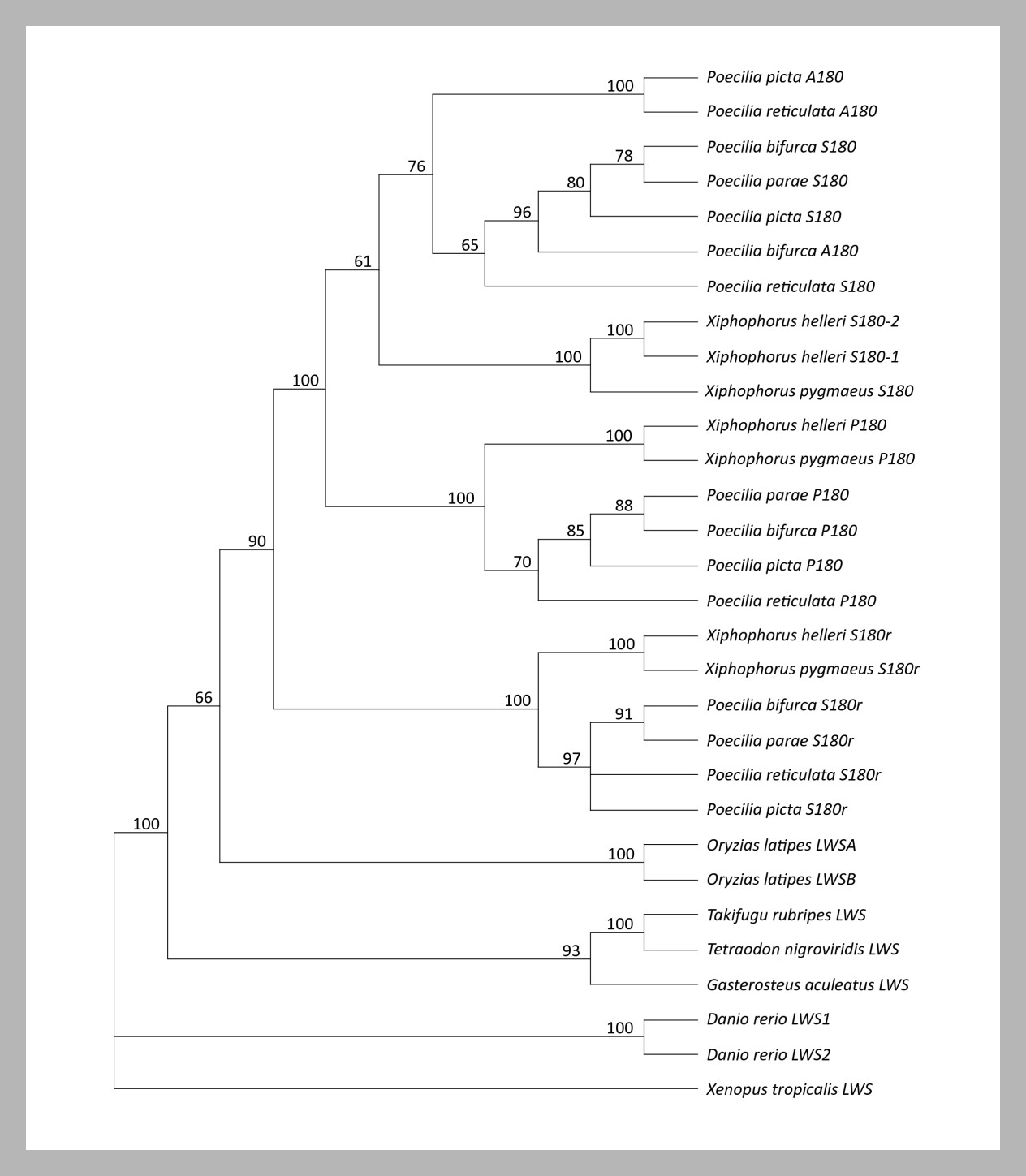

Supplement: Additional file 6 — ZIP files containing several folders, each of which with TreeSnatcher Plus snapshot files, the original image and a text file. [file 1471-2105-13-110-S6.zip › 1471-2148-10-87-3/1471-2148-10-87-3-l_o.PNG]
