# Supplementary figures and images for: TreeSnatcher plus: capturing phylogenetic trees from images (part 5 of 5)
Source: BMC Bioinformatics. 2012 May 24;13:110. doi: 10.1186/1471-2105-13-110 (PMC3411374; doi:10.1186/1471-2105-13-110)

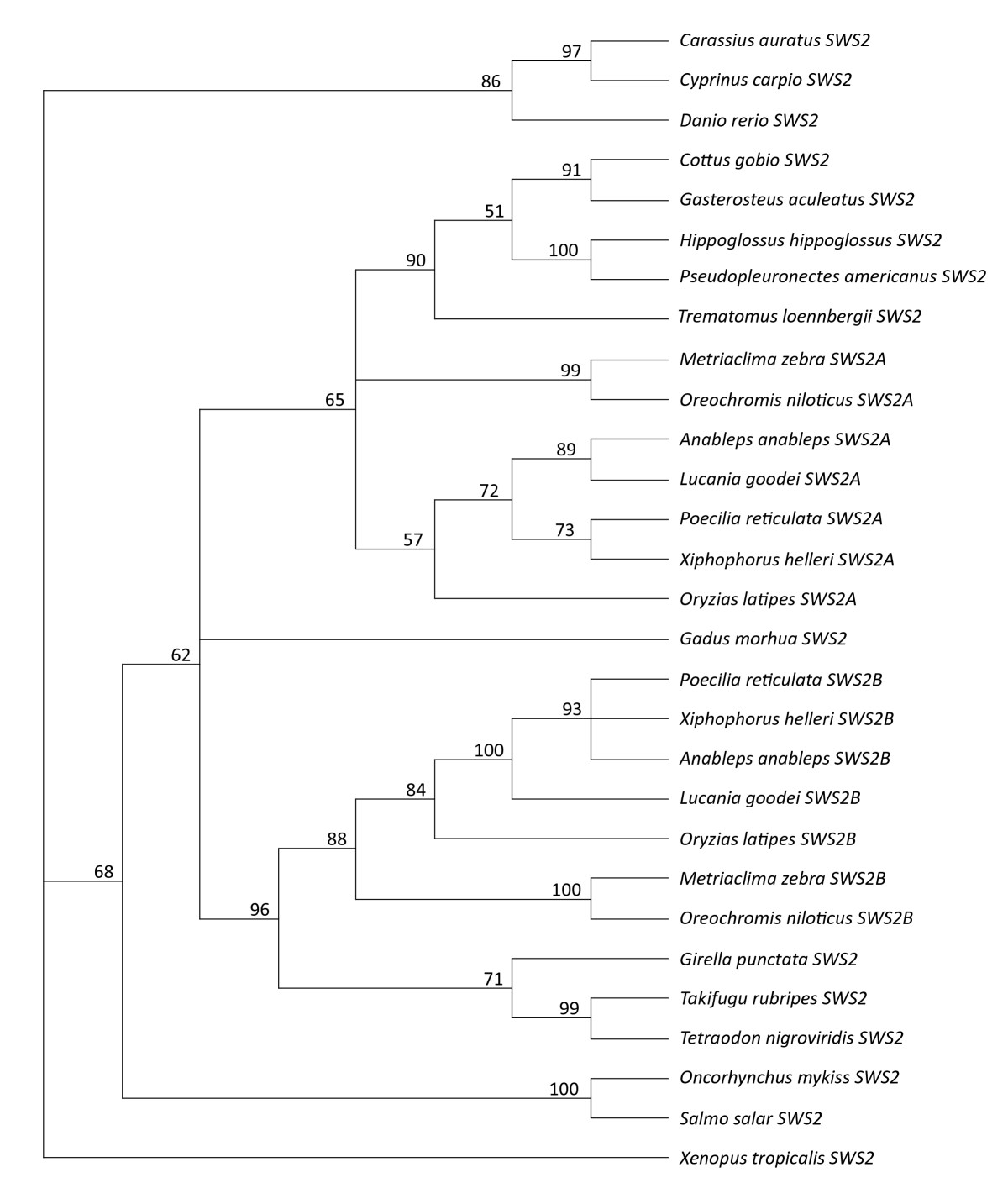

Supplement: Additional file 6 — ZIP files containing several folders, each of which with TreeSnatcher Plus snapshot files, the original image and a text file. [file 1471-2105-13-110-S6.zip › 1471-2148-10-87-4/1471-2148-10-87-4-l.jpg]

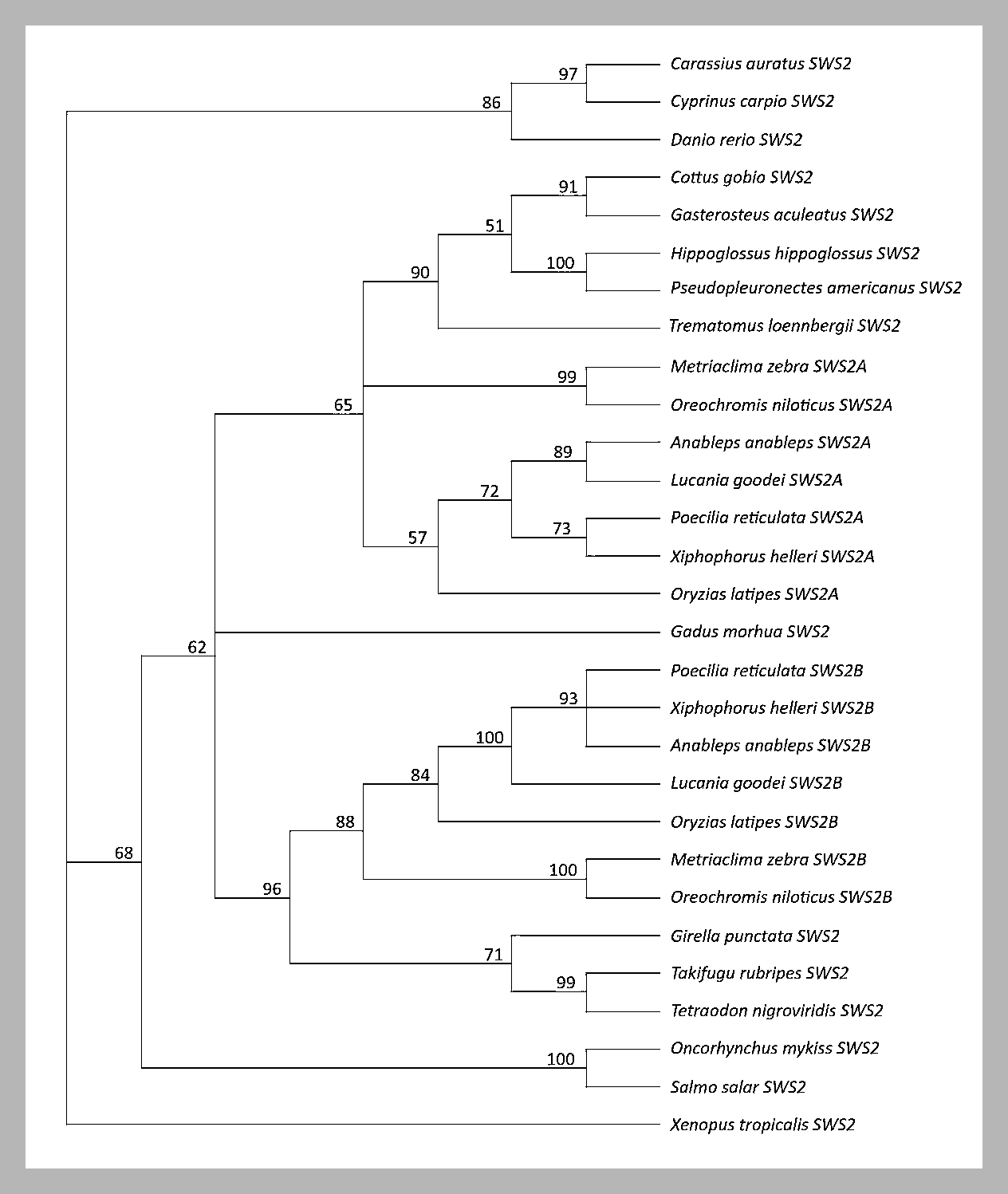

Supplement: Additional file 6 — ZIP files containing several folders, each of which with TreeSnatcher Plus snapshot files, the original image and a text file. [file 1471-2105-13-110-S6.zip › 1471-2148-10-87-4/1471-2148-10-87-4-l_b.PNG]

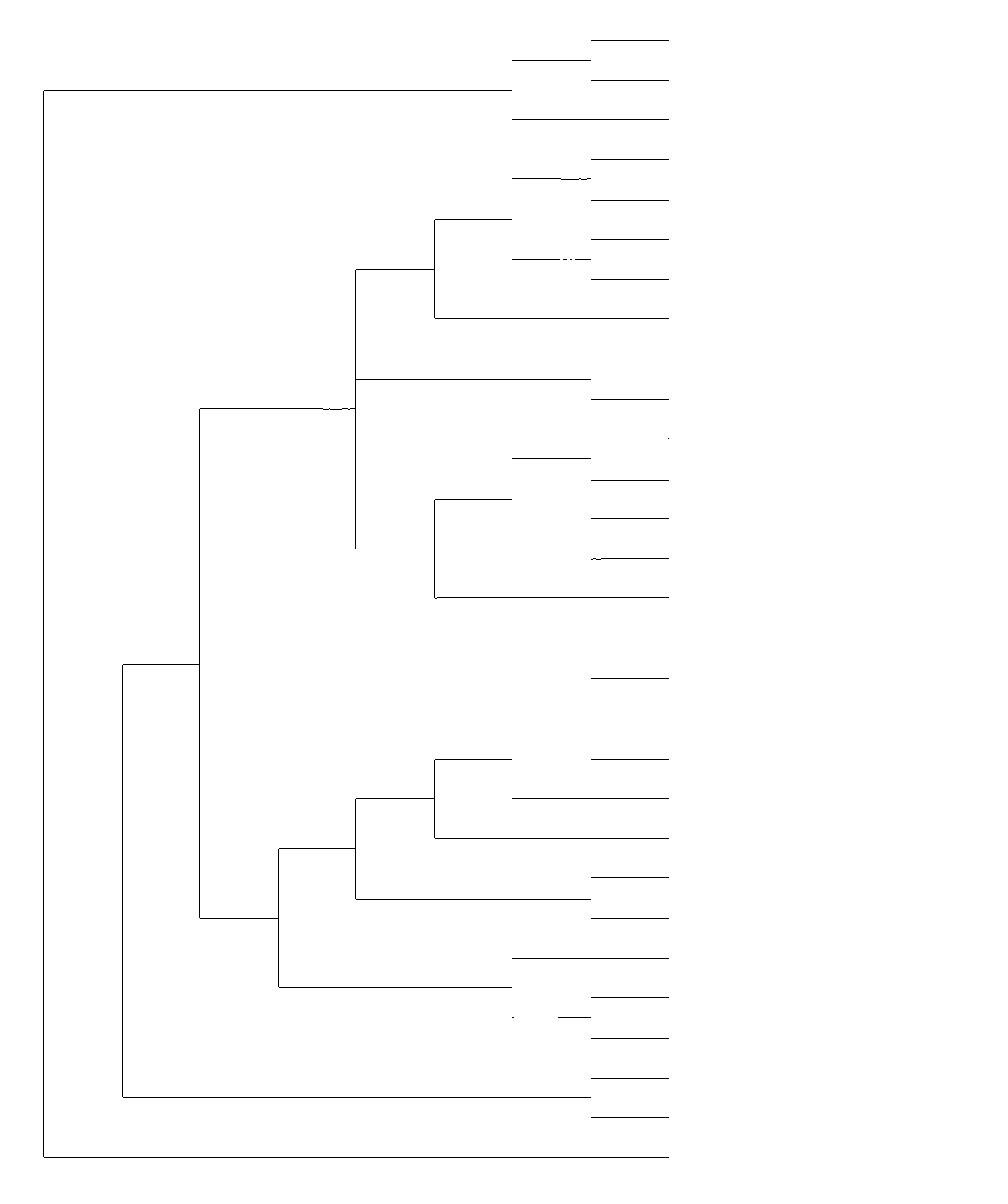

Supplement: Additional file 6 — ZIP files containing several folders, each of which with TreeSnatcher Plus snapshot files, the original image and a text file. [file 1471-2105-13-110-S6.zip › 1471-2148-10-87-4/1471-2148-10-87-4-l_c.PNG]

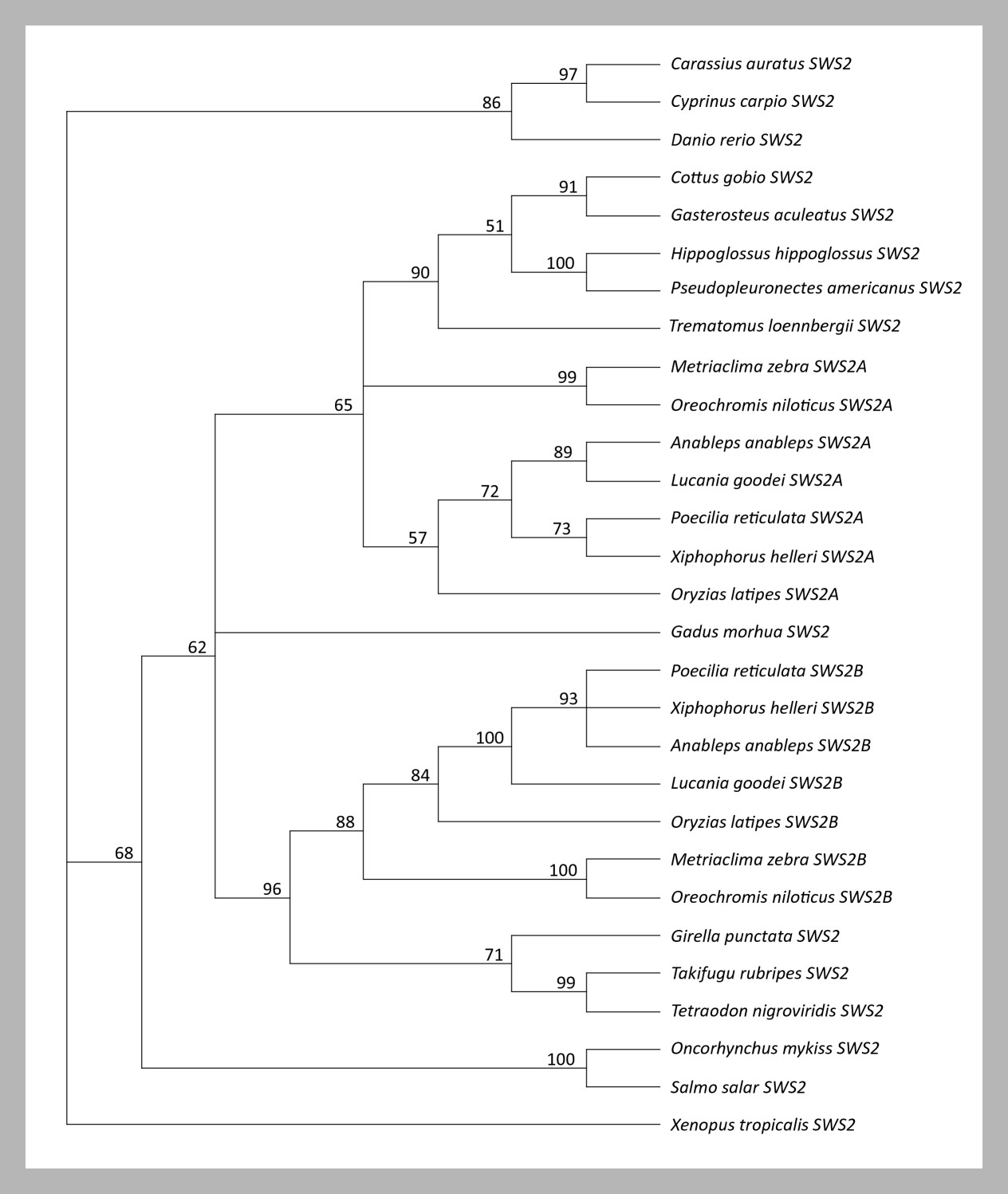

Supplement: Additional file 6 — ZIP files containing several folders, each of which with TreeSnatcher Plus snapshot files, the original image and a text file. [file 1471-2105-13-110-S6.zip › 1471-2148-10-87-4/1471-2148-10-87-4-l_o.PNG]

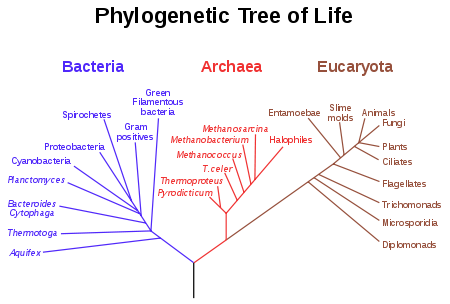

Supplement: Additional file 6 — ZIP files containing several folders, each of which with TreeSnatcher Plus snapshot files, the original image and a text file. [file 1471-2105-13-110-S6.zip › OtherTrees/450px_Phylogenetic_tree/450px-Phylogenetic_tree.svg.png]

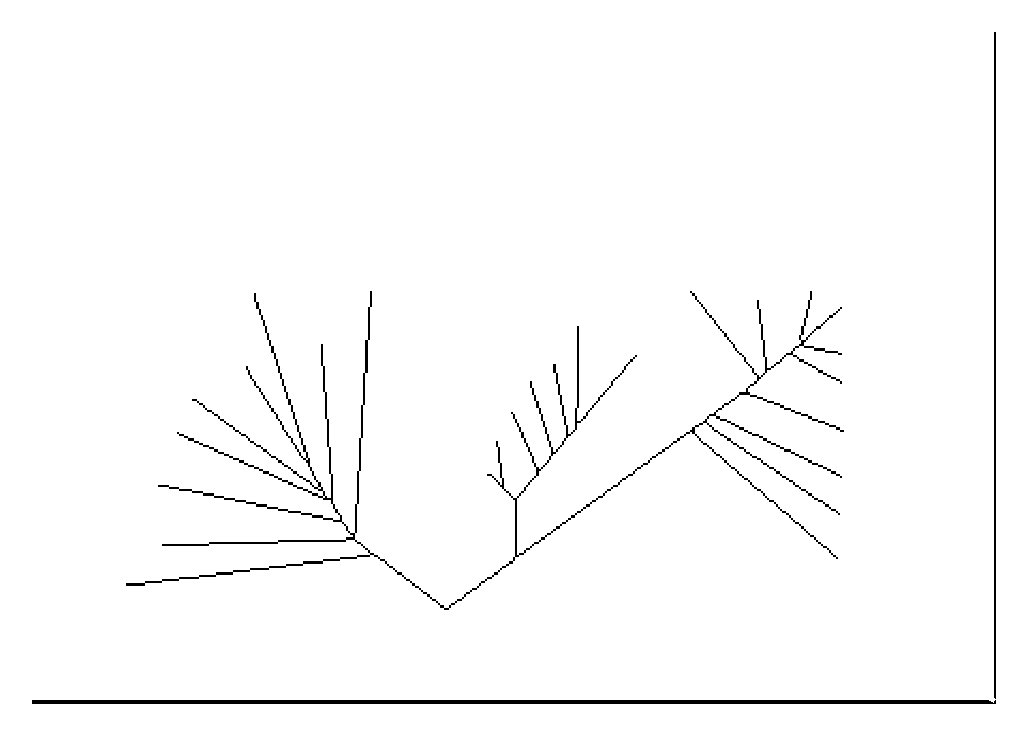

Supplement: Additional file 6 — ZIP files containing several folders, each of which with TreeSnatcher Plus snapshot files, the original image and a text file. [file 1471-2105-13-110-S6.zip › OtherTrees/450px_Phylogenetic_tree/450px-Phylogenetic_tree.svg_b.PNG]

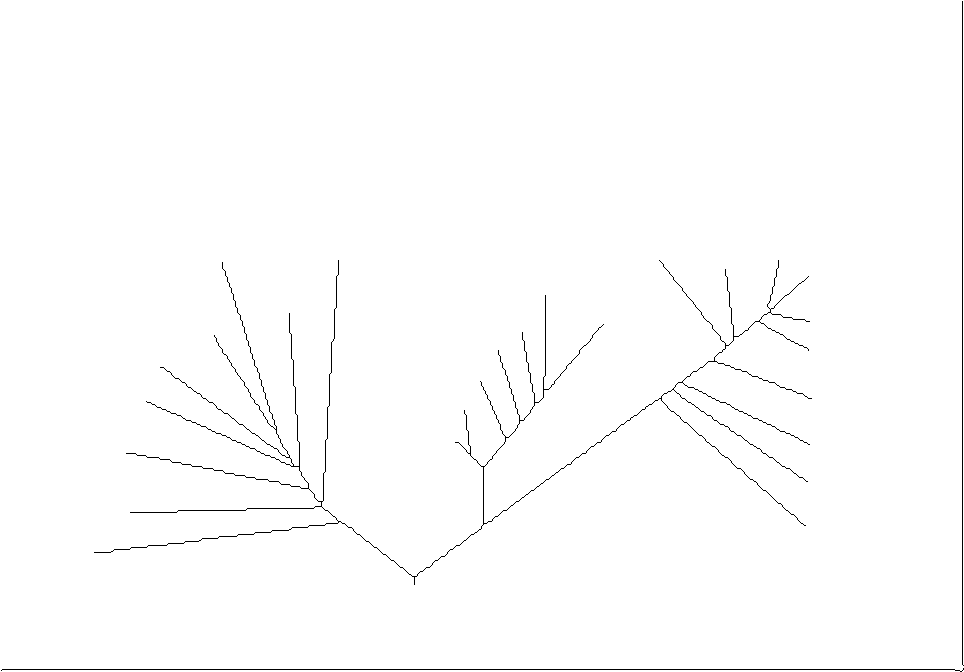

Supplement: Additional file 6 — ZIP files containing several folders, each of which with TreeSnatcher Plus snapshot files, the original image and a text file. [file 1471-2105-13-110-S6.zip › OtherTrees/450px_Phylogenetic_tree/450px-Phylogenetic_tree.svg_c.PNG]

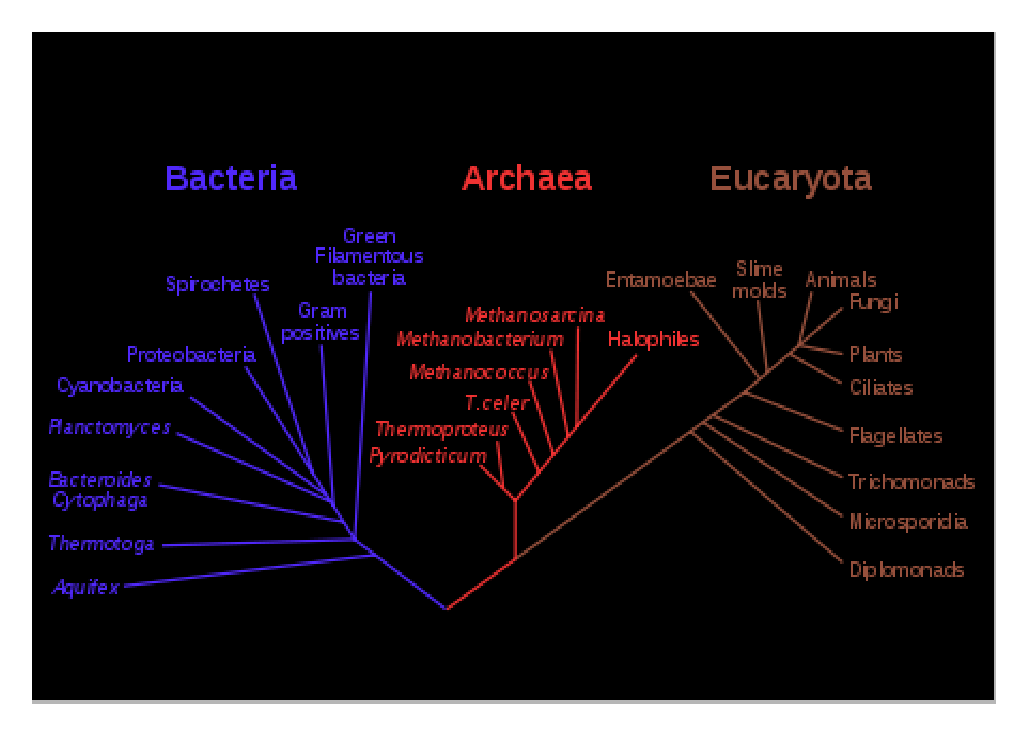

Supplement: Additional file 6 — ZIP files containing several folders, each of which with TreeSnatcher Plus snapshot files, the original image and a text file. [file 1471-2105-13-110-S6.zip › OtherTrees/450px_Phylogenetic_tree/450px-Phylogenetic_tree.svg_o.PNG]

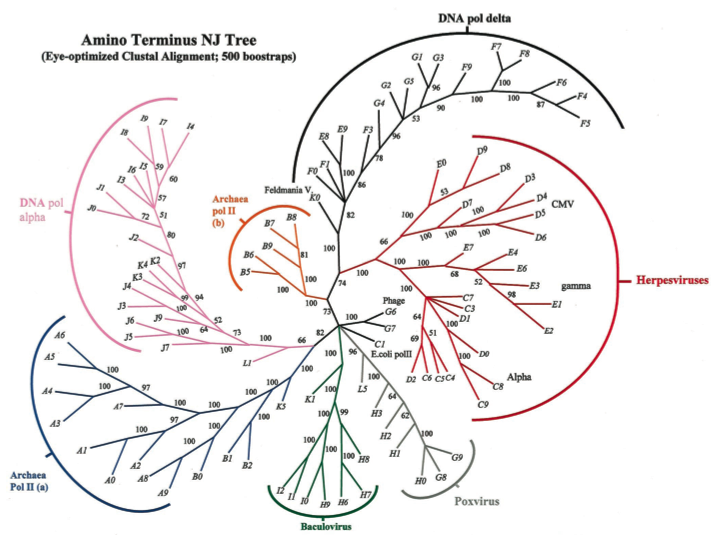

Supplement: Additional file 6 — ZIP files containing several folders, each of which with TreeSnatcher Plus snapshot files, the original image and a text file. [file 1471-2105-13-110-S6.zip › OtherTrees/DNApolamino/DNApolamino.png]

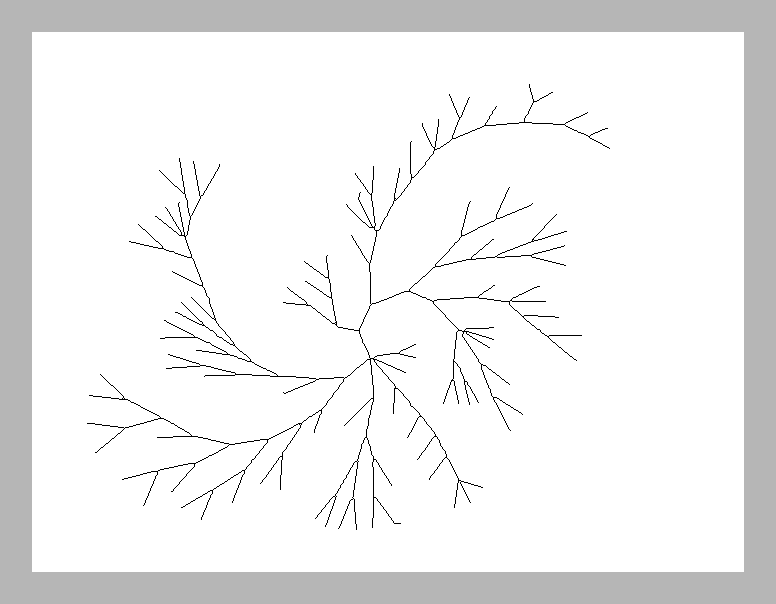

Supplement: Additional file 6 — ZIP files containing several folders, each of which with TreeSnatcher Plus snapshot files, the original image and a text file. [file 1471-2105-13-110-S6.zip › OtherTrees/DNApolamino/DNApolamino_b.PNG]

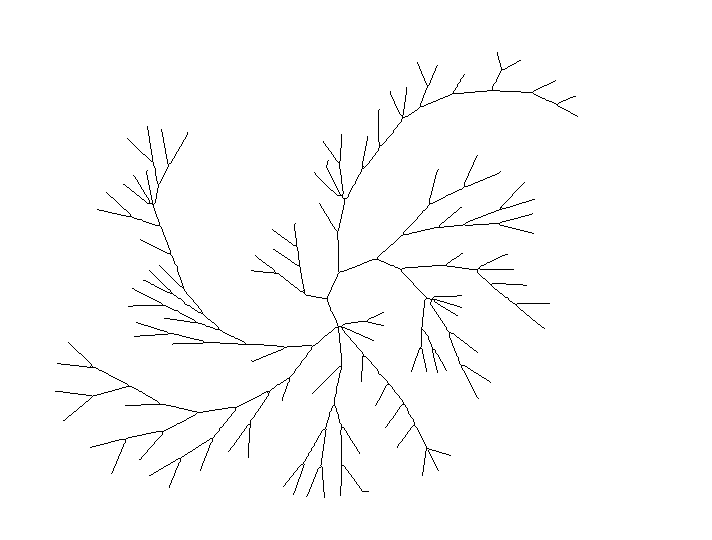

Supplement: Additional file 6 — ZIP files containing several folders, each of which with TreeSnatcher Plus snapshot files, the original image and a text file. [file 1471-2105-13-110-S6.zip › OtherTrees/DNApolamino/DNApolamino_c.PNG]

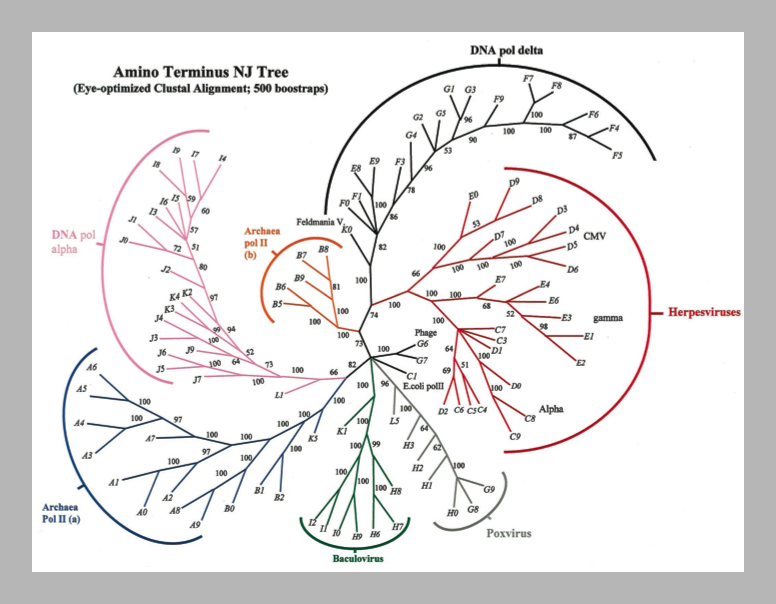

Supplement: Additional file 6 — ZIP files containing several folders, each of which with TreeSnatcher Plus snapshot files, the original image and a text file. [file 1471-2105-13-110-S6.zip › OtherTrees/DNApolamino/DNApolamino_o.PNG]

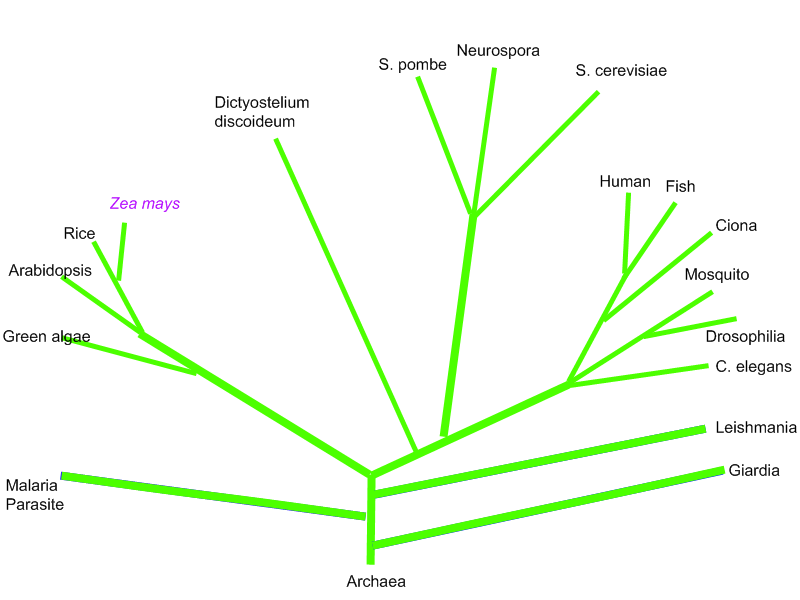

Supplement: Additional file 6 — ZIP files containing several folders, each of which with TreeSnatcher Plus snapshot files, the original image and a text file. [file 1471-2105-13-110-S6.zip › OtherTrees/Phylogenetic Tree 2/Phylogenetic Tree 2.png]

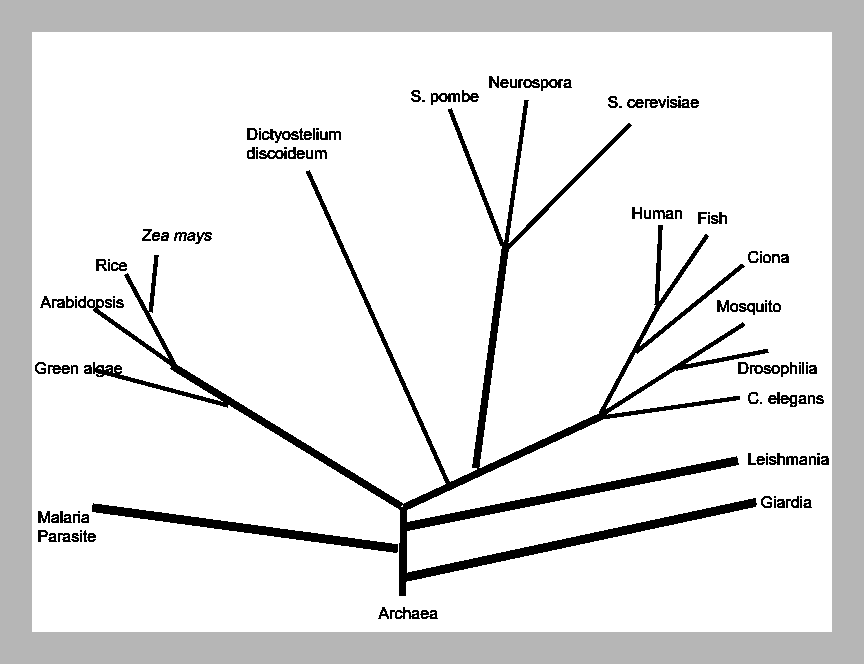

Supplement: Additional file 6 — ZIP files containing several folders, each of which with TreeSnatcher Plus snapshot files, the original image and a text file. [file 1471-2105-13-110-S6.zip › OtherTrees/Phylogenetic Tree 2/Phylogenetic Tree 2_b.PNG]

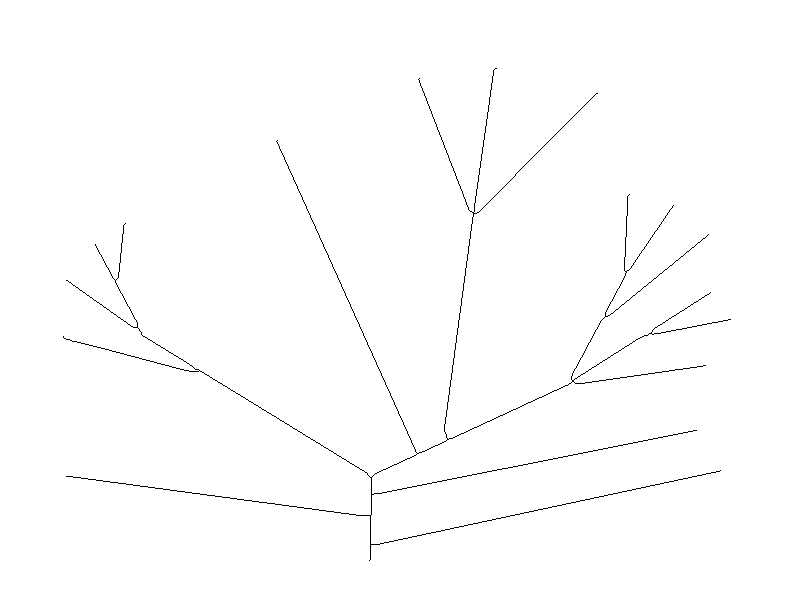

Supplement: Additional file 6 — ZIP files containing several folders, each of which with TreeSnatcher Plus snapshot files, the original image and a text file. [file 1471-2105-13-110-S6.zip › OtherTrees/Phylogenetic Tree 2/Phylogenetic Tree 2_c.PNG]

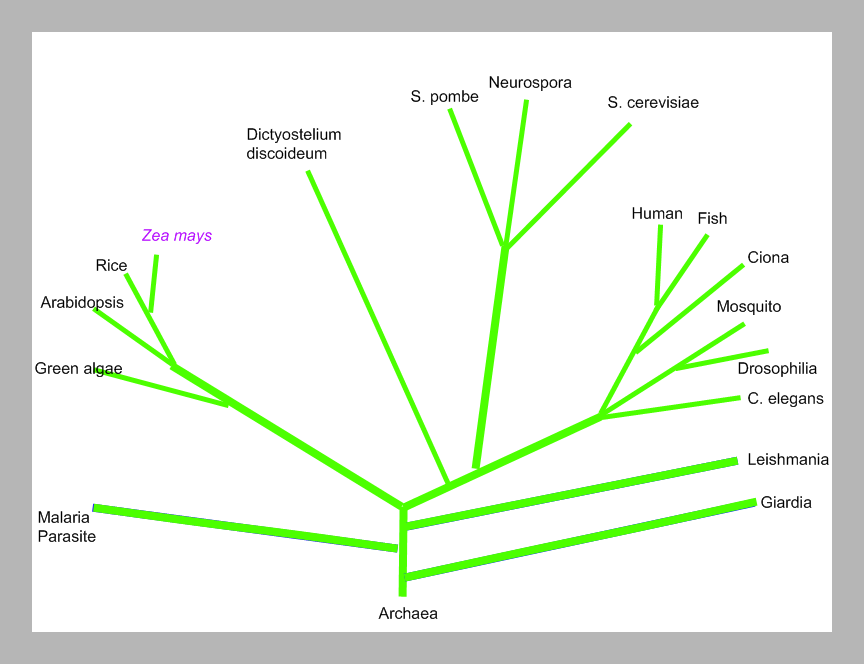

Supplement: Additional file 6 — ZIP files containing several folders, each of which with TreeSnatcher Plus snapshot files, the original image and a text file. [file 1471-2105-13-110-S6.zip › OtherTrees/Phylogenetic Tree 2/Phylogenetic Tree 2_o.PNG]

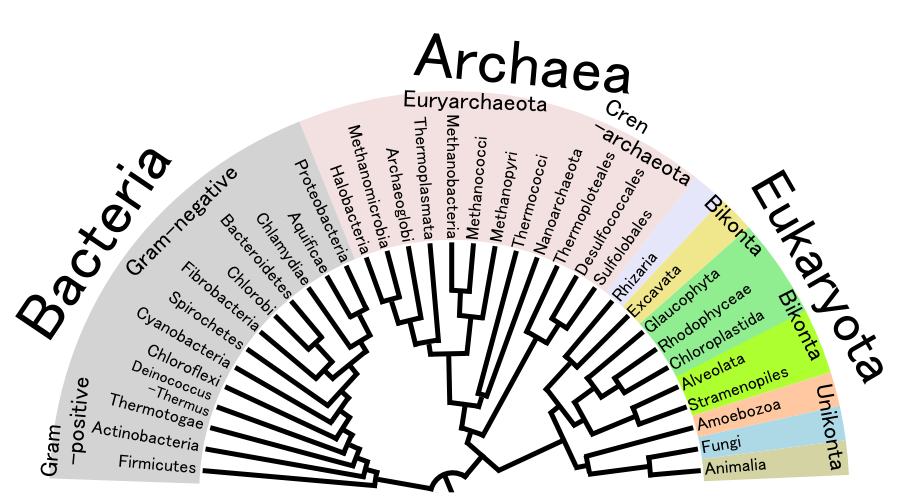

Supplement: Additional file 6 — ZIP files containing several folders, each of which with TreeSnatcher Plus snapshot files, the original image and a text file. [file 1471-2105-13-110-S6.zip › OtherTrees/Phylogenetic_Tree_of_Life/Phylogenetic_Tree_of_Life.png]

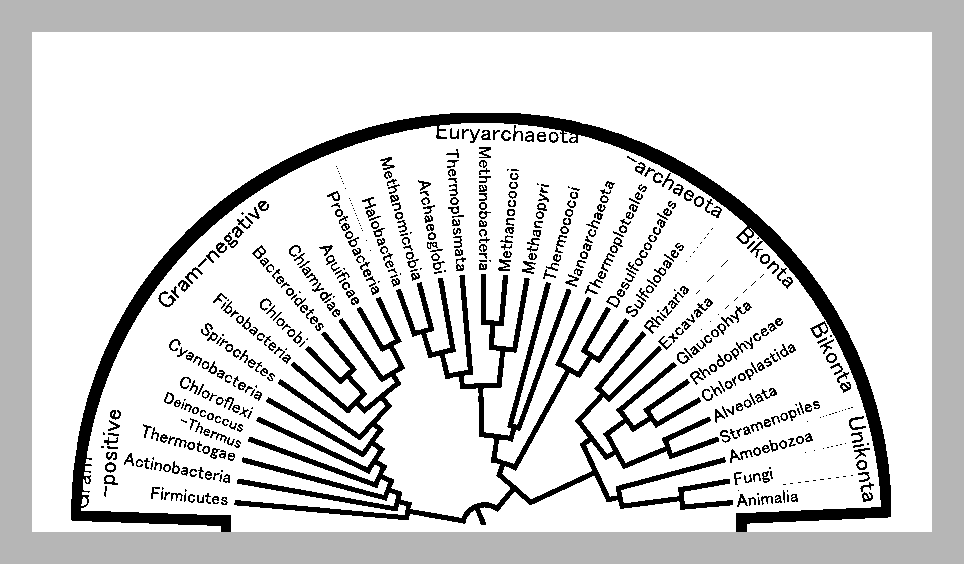

Supplement: Additional file 6 — ZIP files containing several folders, each of which with TreeSnatcher Plus snapshot files, the original image and a text file. [file 1471-2105-13-110-S6.zip › OtherTrees/Phylogenetic_Tree_of_Life/Phylogenetic_Tree_of_Life_b.PNG]

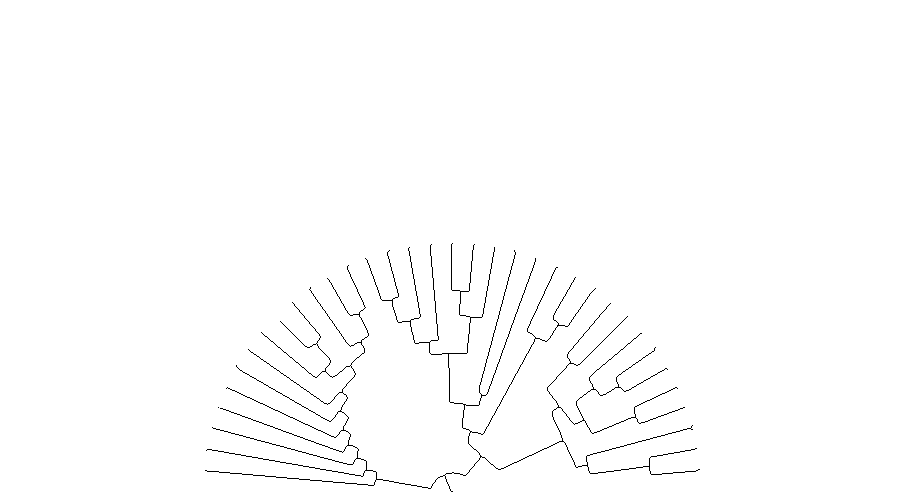

Supplement: Additional file 6 — ZIP files containing several folders, each of which with TreeSnatcher Plus snapshot files, the original image and a text file. [file 1471-2105-13-110-S6.zip › OtherTrees/Phylogenetic_Tree_of_Life/Phylogenetic_Tree_of_Life_c.PNG]

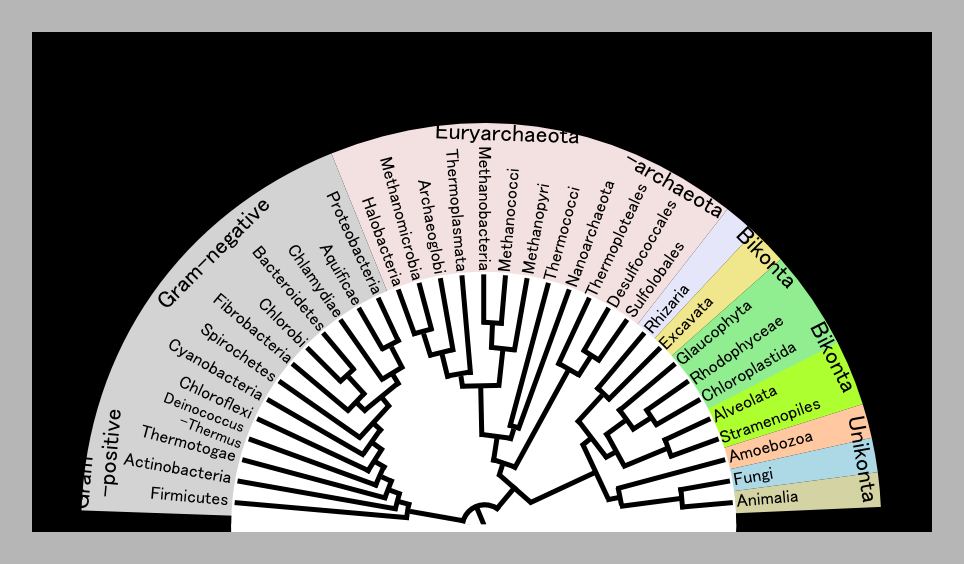

Supplement: Additional file 6 — ZIP files containing several folders, each of which with TreeSnatcher Plus snapshot files, the original image and a text file. [file 1471-2105-13-110-S6.zip › OtherTrees/Phylogenetic_Tree_of_Life/Phylogenetic_Tree_of_Life_o.PNG]

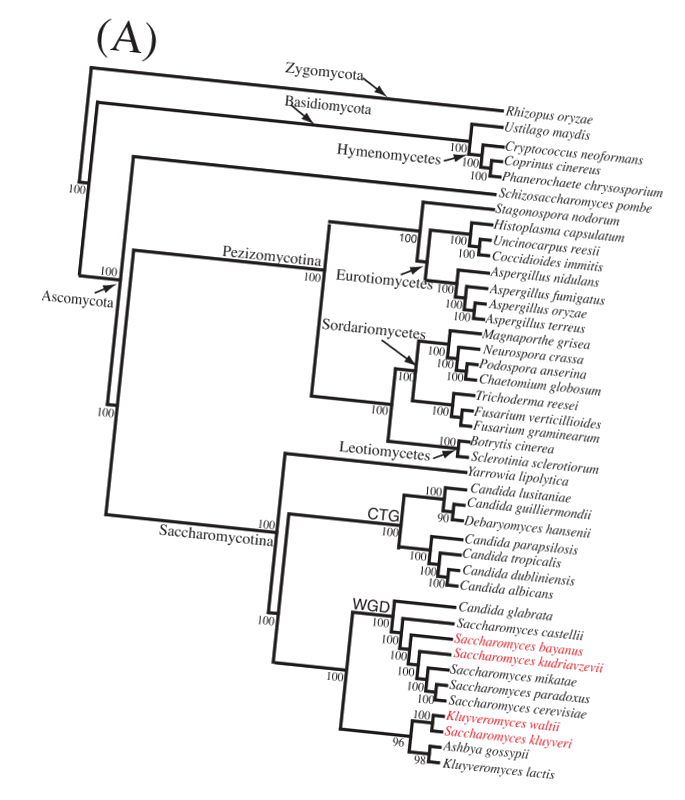

Supplement: Additional file 6 — ZIP files containing several folders, each of which with TreeSnatcher Plus snapshot files, the original image and a text file. [file 1471-2105-13-110-S6.zip › OtherTrees/RotatedTree/1471-2148-6-99-1-l_rotated.png]

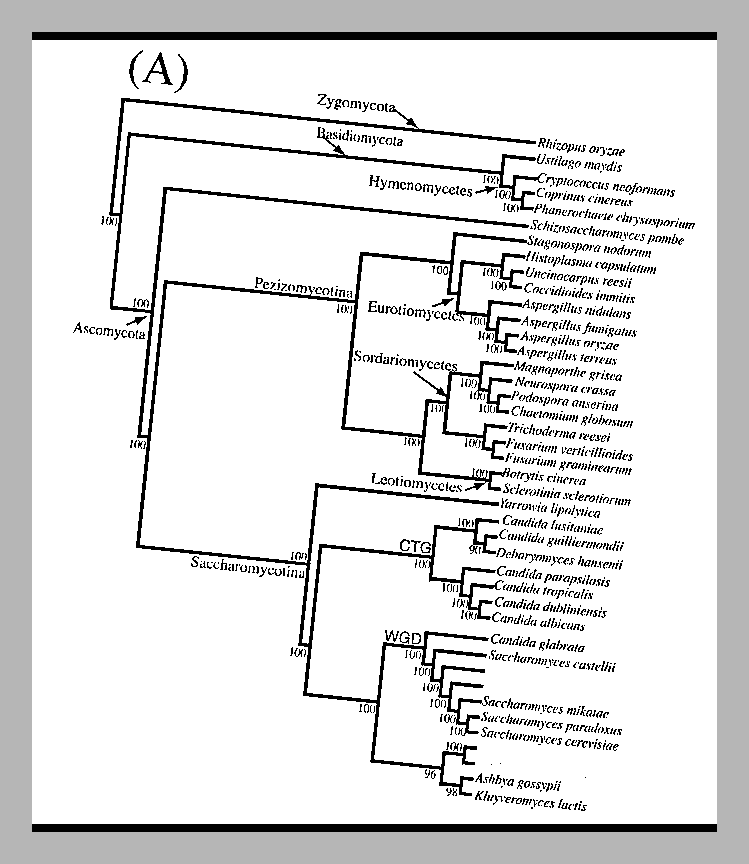

Supplement: Additional file 6 — ZIP files containing several folders, each of which with TreeSnatcher Plus snapshot files, the original image and a text file. [file 1471-2105-13-110-S6.zip › OtherTrees/RotatedTree/1471-2148-6-99-1-l_rotated_b.PNG]

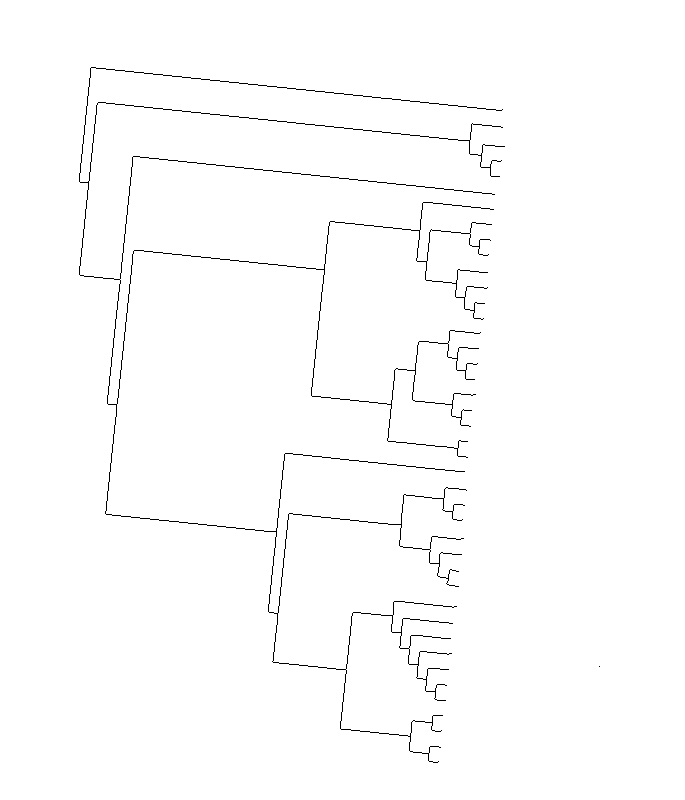

Supplement: Additional file 6 — ZIP files containing several folders, each of which with TreeSnatcher Plus snapshot files, the original image and a text file. [file 1471-2105-13-110-S6.zip › OtherTrees/RotatedTree/1471-2148-6-99-1-l_rotated_c.PNG]

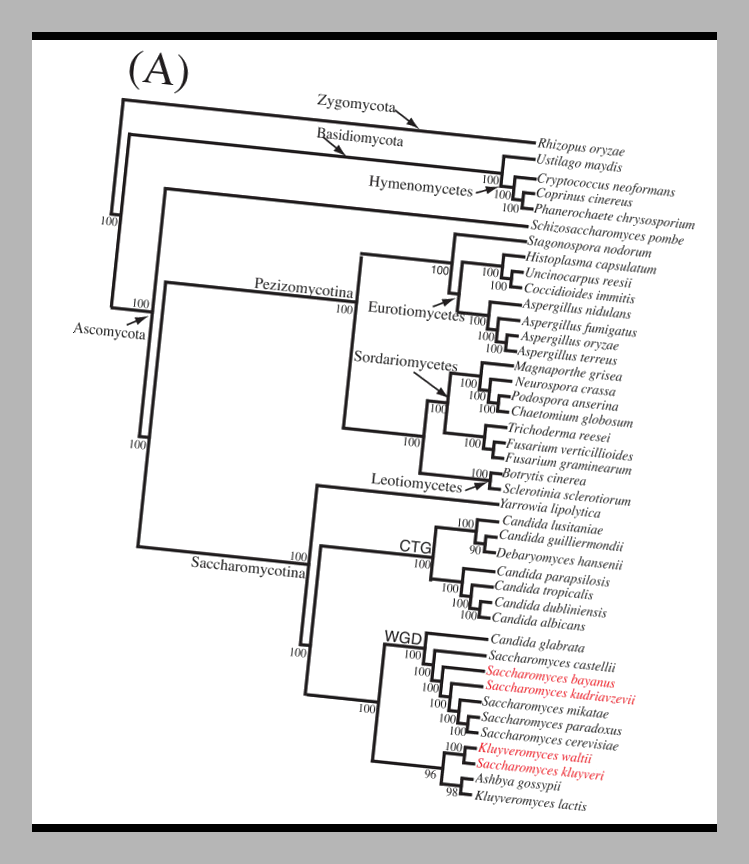

Supplement: Additional file 6 — ZIP files containing several folders, each of which with TreeSnatcher Plus snapshot files, the original image and a text file. [file 1471-2105-13-110-S6.zip › OtherTrees/RotatedTree/1471-2148-6-99-1-l_rotated_o.PNG]

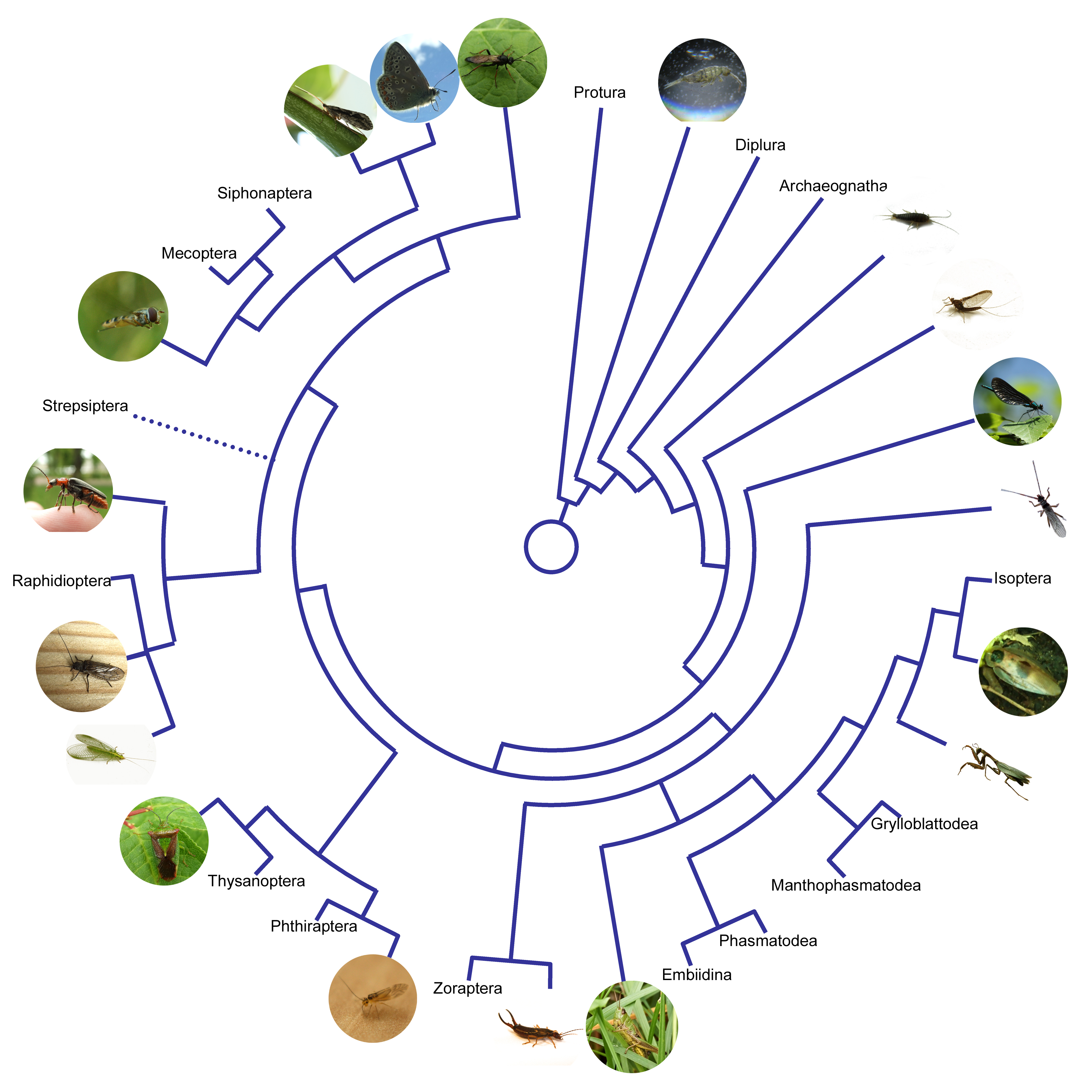

Supplement: Additional file 6 — ZIP files containing several folders, each of which with TreeSnatcher Plus snapshot files, the original image and a text file. [file 1471-2105-13-110-S6.zip › OtherTrees/TreeOfLife/TreeofLife.jpg]

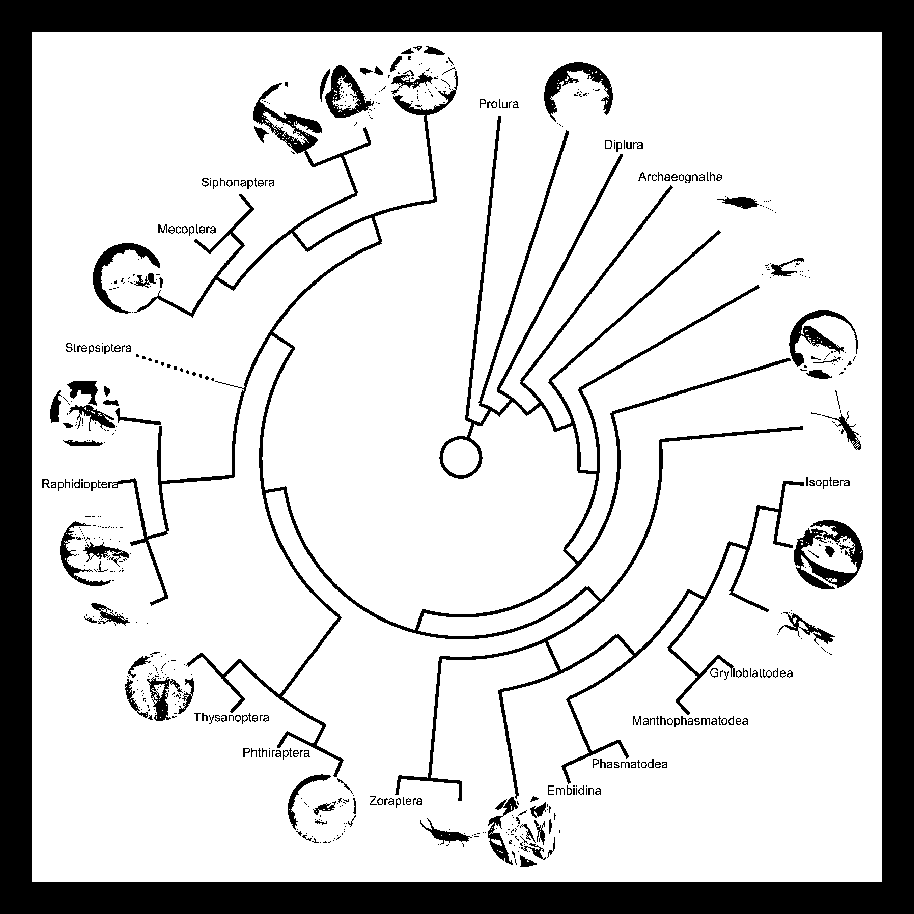

Supplement: Additional file 6 — ZIP files containing several folders, each of which with TreeSnatcher Plus snapshot files, the original image and a text file. [file 1471-2105-13-110-S6.zip › OtherTrees/TreeOfLife/TreeofLife_b.PNG]

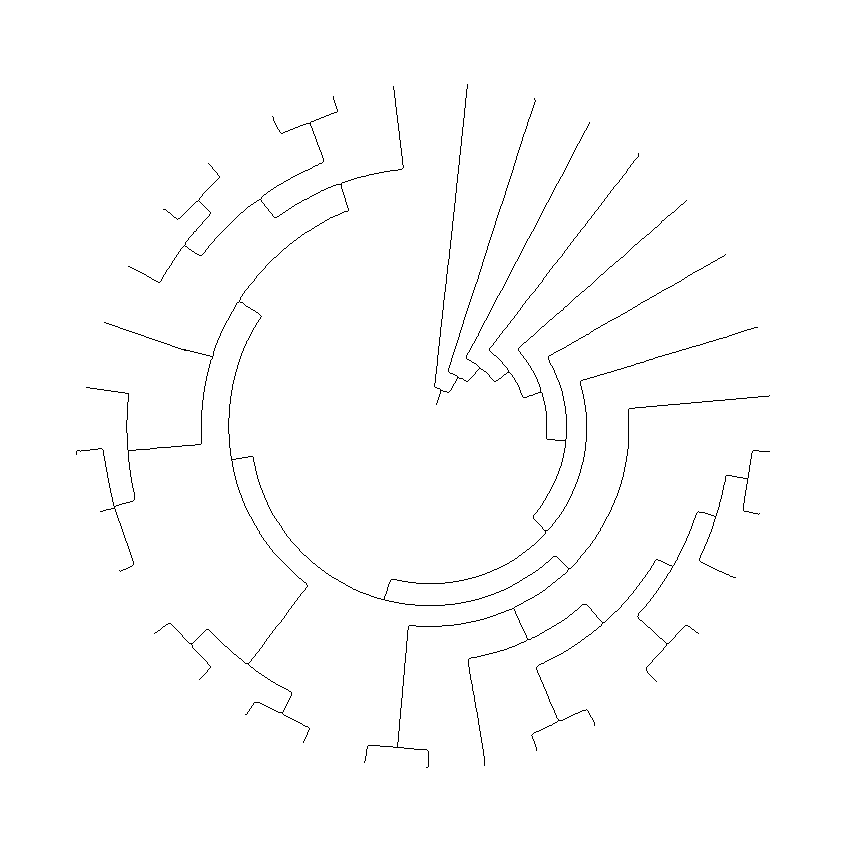

Supplement: Additional file 6 — ZIP files containing several folders, each of which with TreeSnatcher Plus snapshot files, the original image and a text file. [file 1471-2105-13-110-S6.zip › OtherTrees/TreeOfLife/TreeofLife_c.PNG]

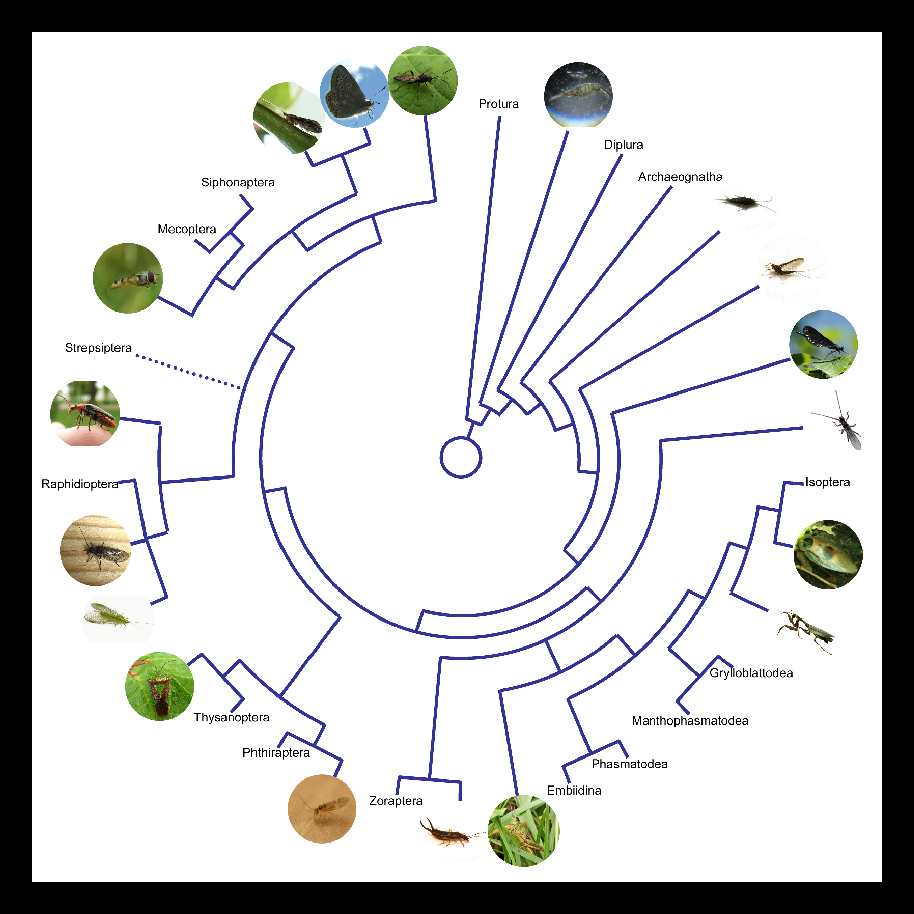

Supplement: Additional file 6 — ZIP files containing several folders, each of which with TreeSnatcher Plus snapshot files, the original image and a text file. [file 1471-2105-13-110-S6.zip › OtherTrees/TreeOfLife/TreeofLife_o.PNG]

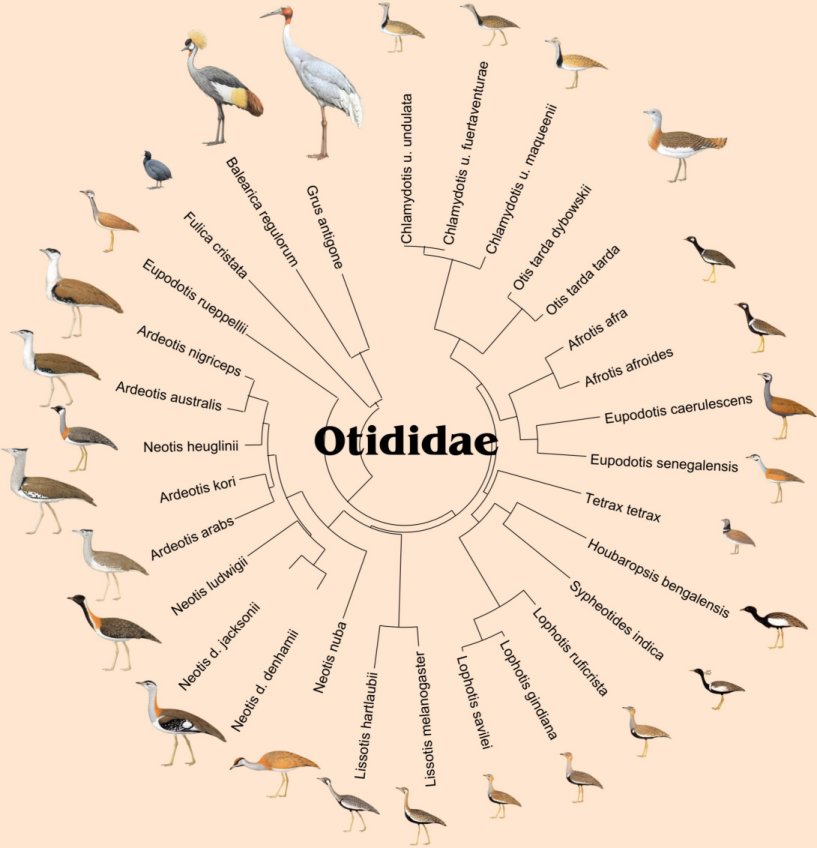

Supplement: Additional file 6 — ZIP files containing several folders, each of which with TreeSnatcher Plus snapshot files, the original image and a text file. [file 1471-2105-13-110-S6.zip › OtherTrees/bustard/bustard.jpg]

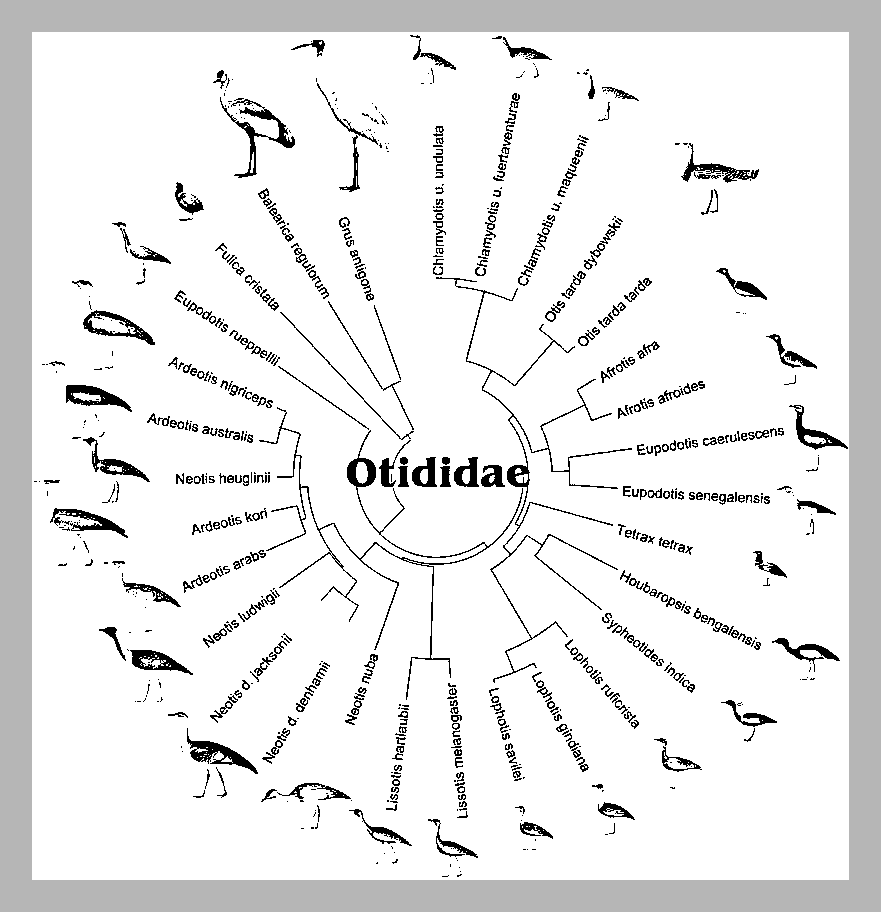

Supplement: Additional file 6 — ZIP files containing several folders, each of which with TreeSnatcher Plus snapshot files, the original image and a text file. [file 1471-2105-13-110-S6.zip › OtherTrees/bustard/bustard_b.PNG]

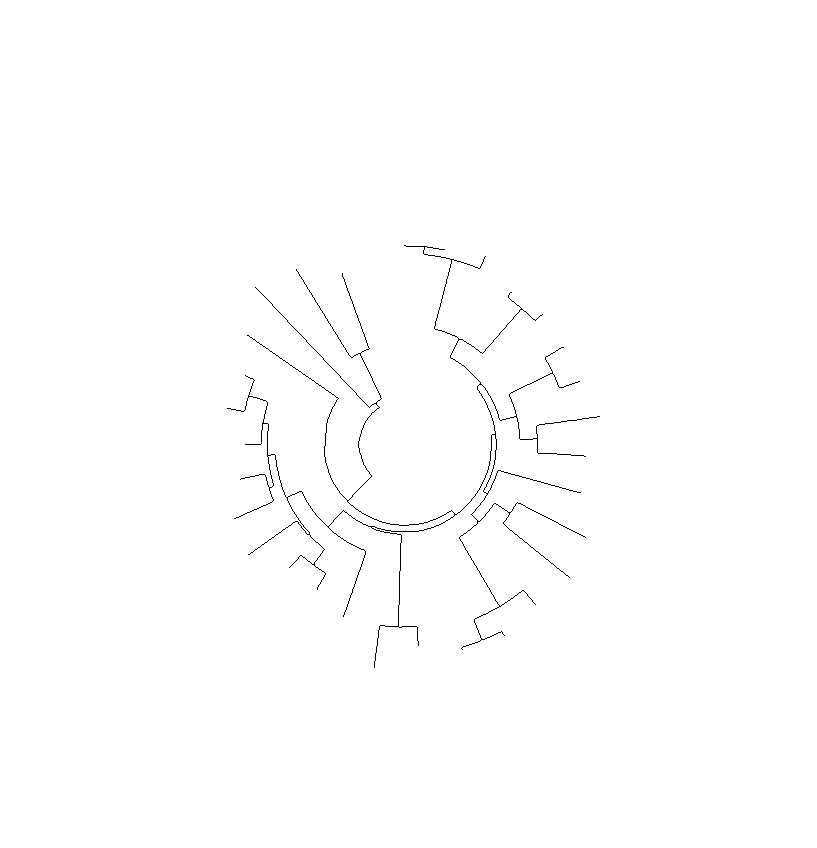

Supplement: Additional file 6 — ZIP files containing several folders, each of which with TreeSnatcher Plus snapshot files, the original image and a text file. [file 1471-2105-13-110-S6.zip › OtherTrees/bustard/bustard_c.PNG]

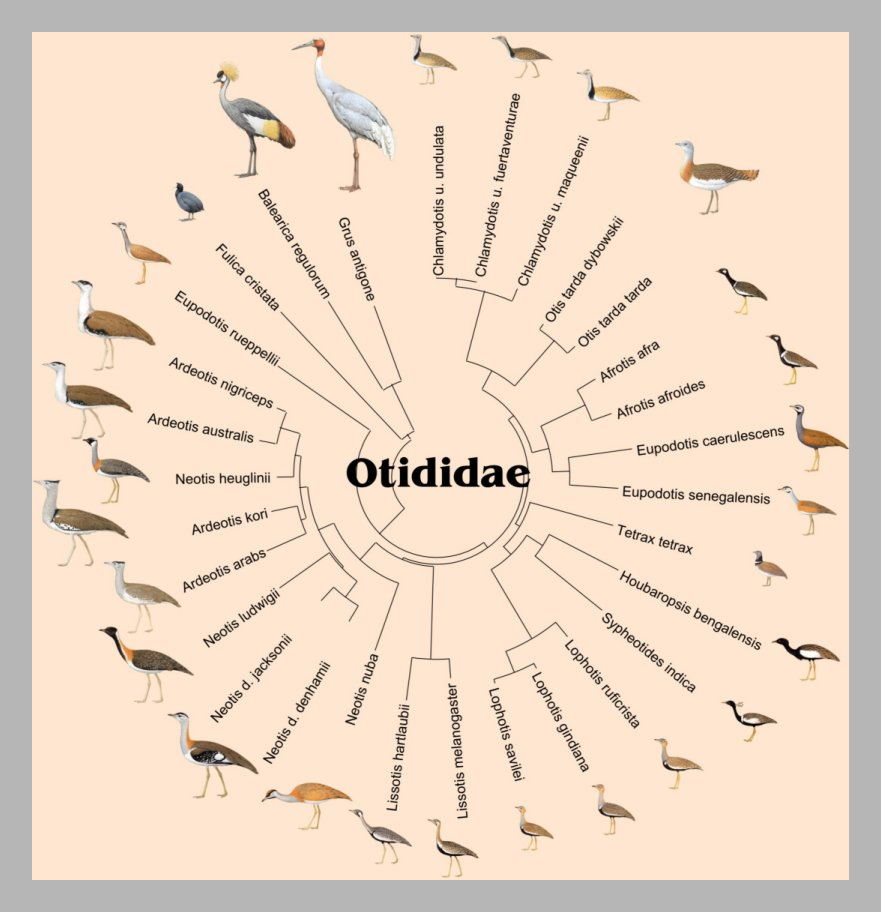

Supplement: Additional file 6 — ZIP files containing several folders, each of which with TreeSnatcher Plus snapshot files, the original image and a text file. [file 1471-2105-13-110-S6.zip › OtherTrees/bustard/bustard_o.PNG]

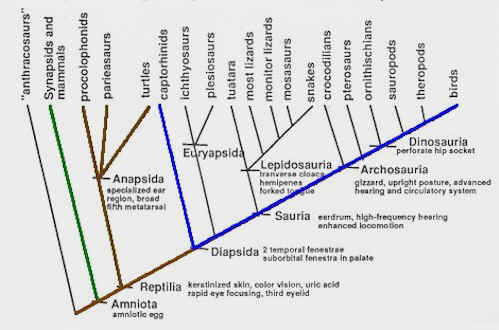

Supplement: Additional file 6 — ZIP files containing several folders, each of which with TreeSnatcher Plus snapshot files, the original image and a text file. [file 1471-2105-13-110-S6.zip › OtherTrees/reptilecladogram14/reptilecladogram14.jpg]

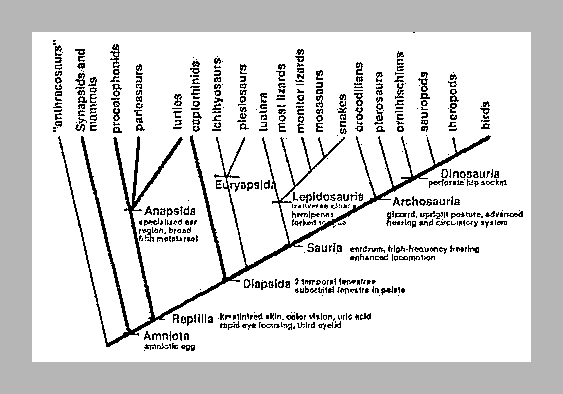

Supplement: Additional file 6 — ZIP files containing several folders, each of which with TreeSnatcher Plus snapshot files, the original image and a text file. [file 1471-2105-13-110-S6.zip › OtherTrees/reptilecladogram14/reptilecladogram14_b.PNG]

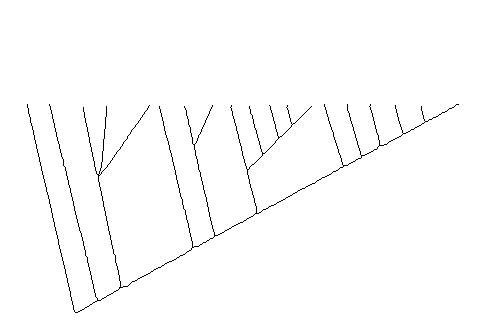

Supplement: Additional file 6 — ZIP files containing several folders, each of which with TreeSnatcher Plus snapshot files, the original image and a text file. [file 1471-2105-13-110-S6.zip › OtherTrees/reptilecladogram14/reptilecladogram14_c.PNG]

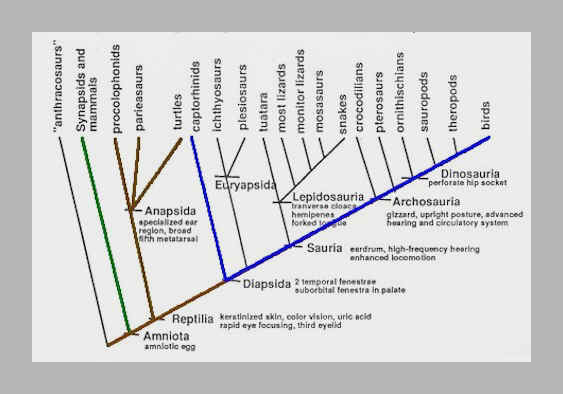

Supplement: Additional file 6 — ZIP files containing several folders, each of which with TreeSnatcher Plus snapshot files, the original image and a text file. [file 1471-2105-13-110-S6.zip › OtherTrees/reptilecladogram14/reptilecladogram14_o.PNG]

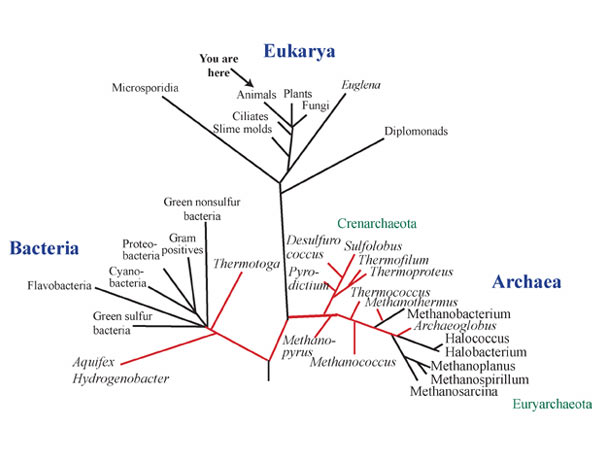

Supplement: Additional file 6 — ZIP files containing several folders, each of which with TreeSnatcher Plus snapshot files, the original image and a text file. [file 1471-2105-13-110-S6.zip › OtherTrees/universal_tree2hotbugs_600/universal_tree2hotbugs_600.jpg]

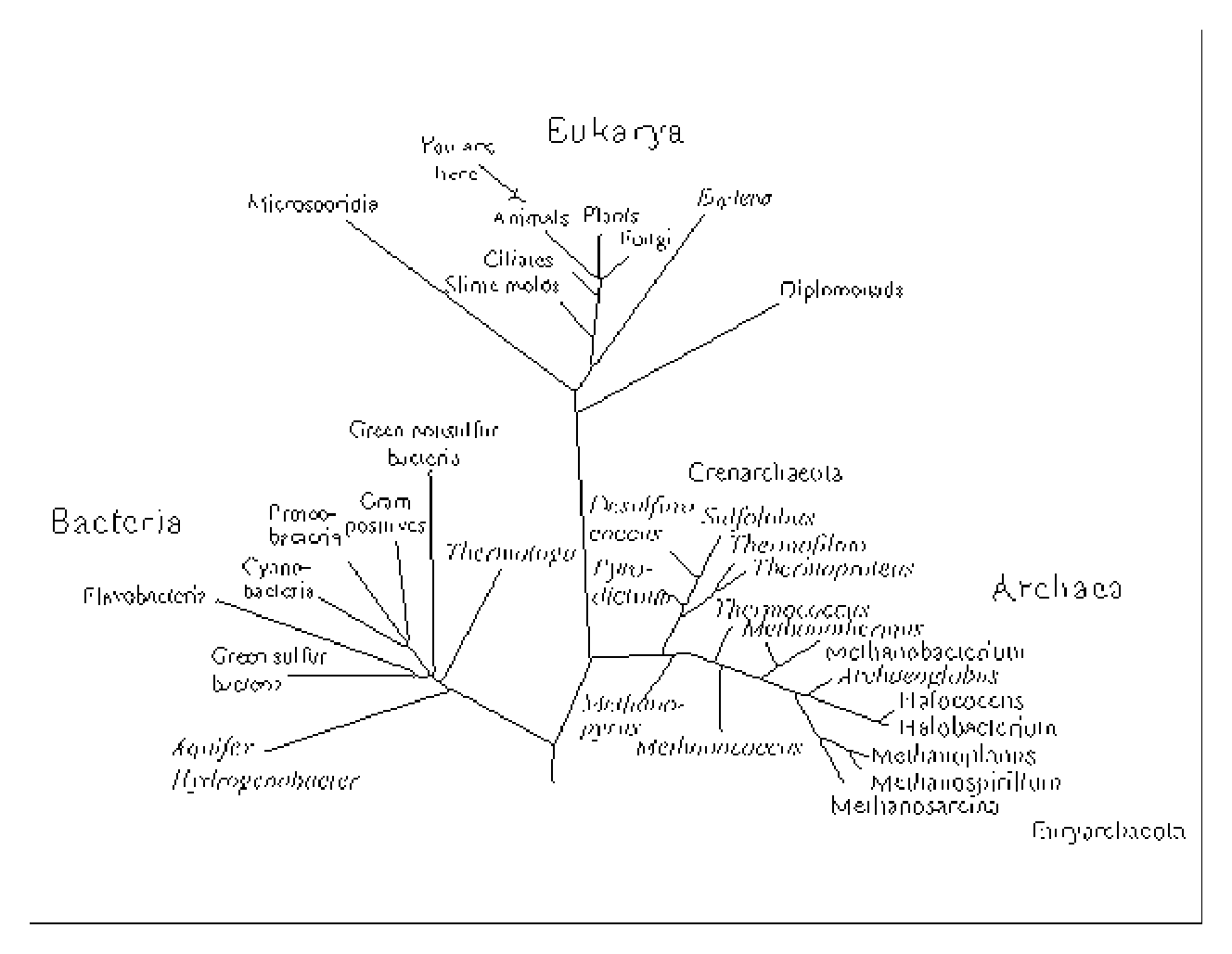

Supplement: Additional file 6 — ZIP files containing several folders, each of which with TreeSnatcher Plus snapshot files, the original image and a text file. [file 1471-2105-13-110-S6.zip › OtherTrees/universal_tree2hotbugs_600/universal_tree2hotbugs_600_b.PNG]

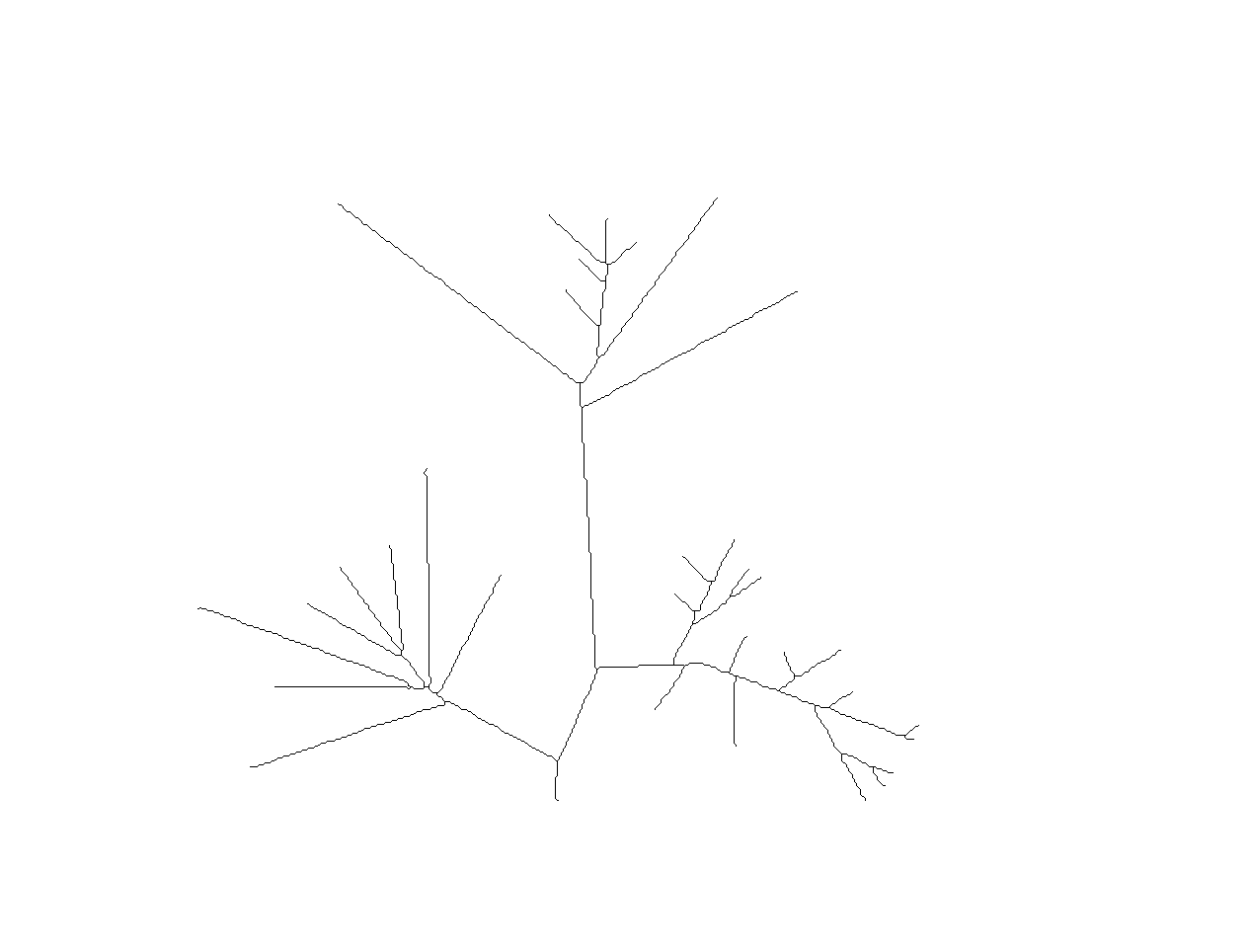

Supplement: Additional file 6 — ZIP files containing several folders, each of which with TreeSnatcher Plus snapshot files, the original image and a text file. [file 1471-2105-13-110-S6.zip › OtherTrees/universal_tree2hotbugs_600/universal_tree2hotbugs_600_c.PNG]

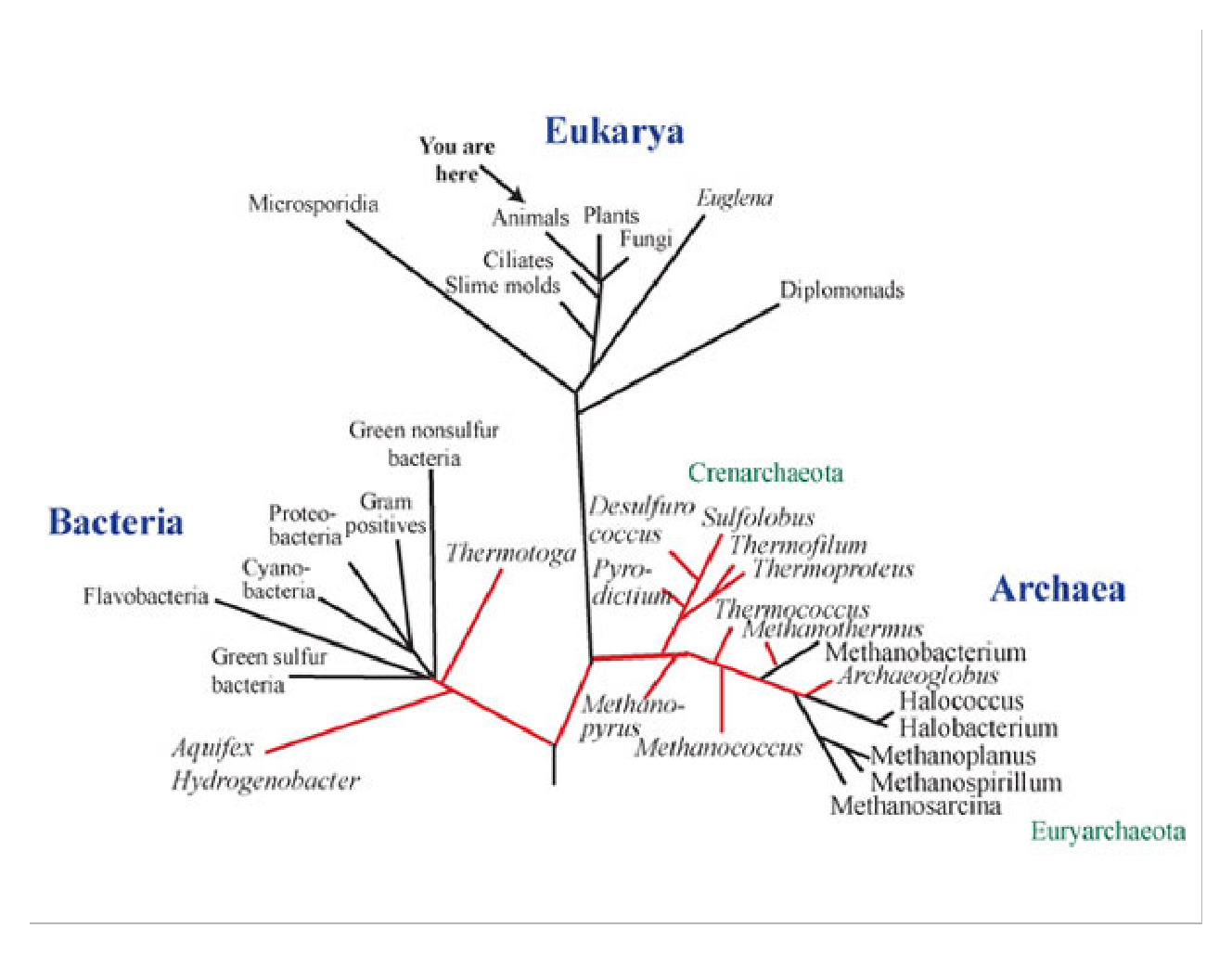

Supplement: Additional file 6 — ZIP files containing several folders, each of which with TreeSnatcher Plus snapshot files, the original image and a text file. [file 1471-2105-13-110-S6.zip › OtherTrees/universal_tree2hotbugs_600/universal_tree2hotbugs_600_o.PNG]

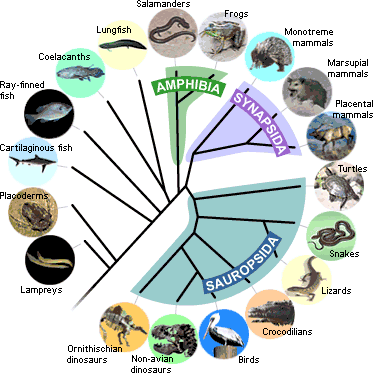

Supplement: Additional file 6 — ZIP files containing several folders, each of which with TreeSnatcher Plus snapshot files, the original image and a text file. [file 1471-2105-13-110-S6.zip › OtherTrees/vert_tree/vert_tree.gif]

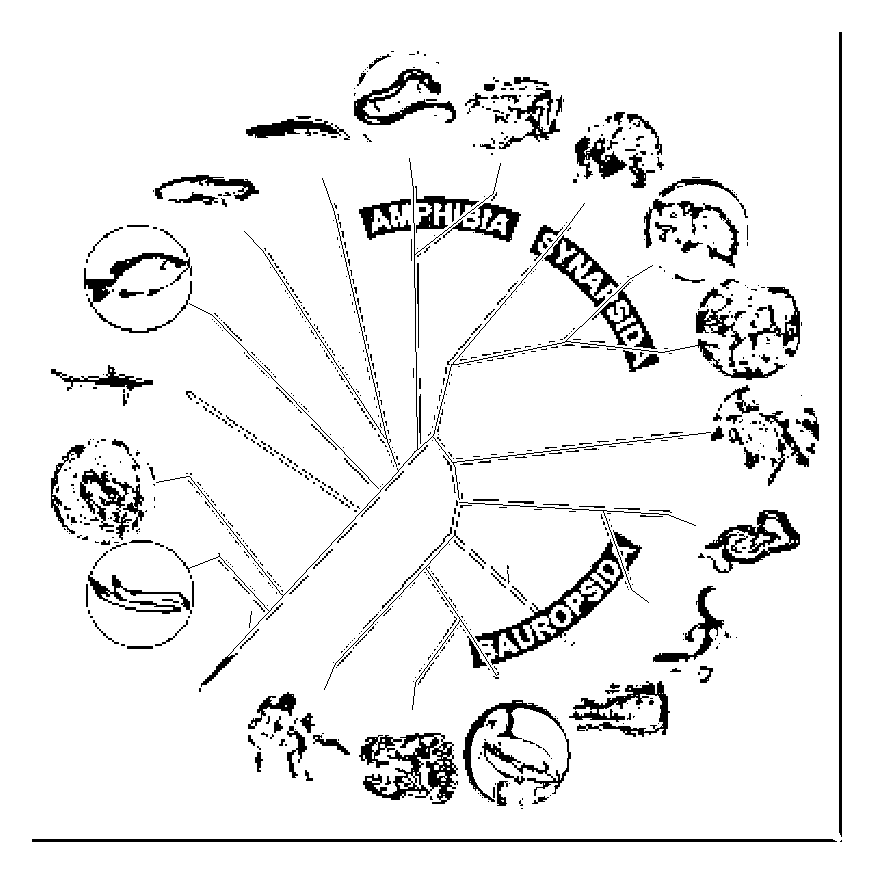

Supplement: Additional file 6 — ZIP files containing several folders, each of which with TreeSnatcher Plus snapshot files, the original image and a text file. [file 1471-2105-13-110-S6.zip › OtherTrees/vert_tree/vert_tree_b.PNG]

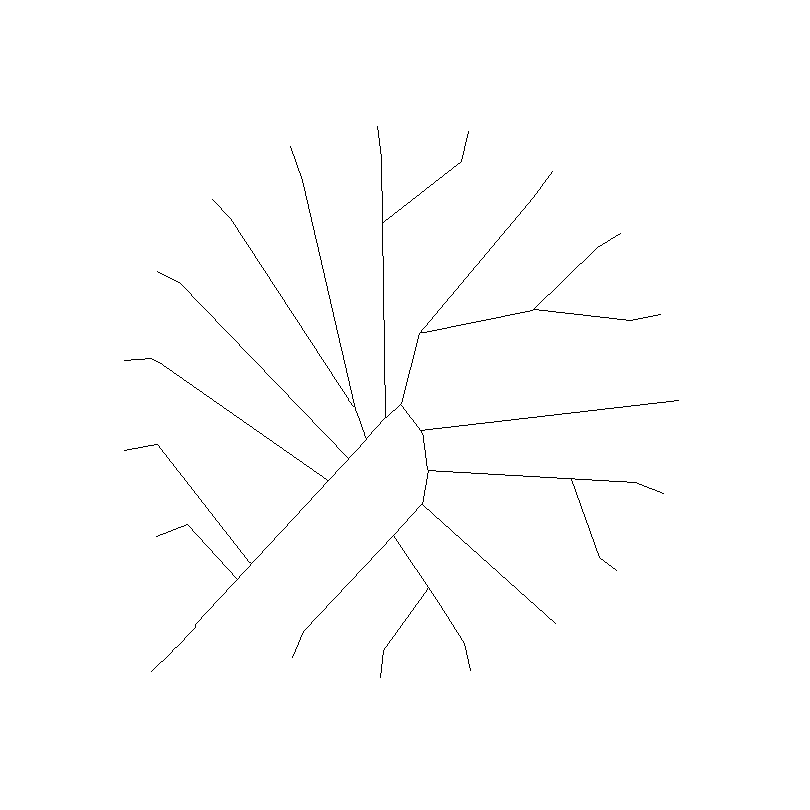

Supplement: Additional file 6 — ZIP files containing several folders, each of which with TreeSnatcher Plus snapshot files, the original image and a text file. [file 1471-2105-13-110-S6.zip › OtherTrees/vert_tree/vert_tree_c.PNG]

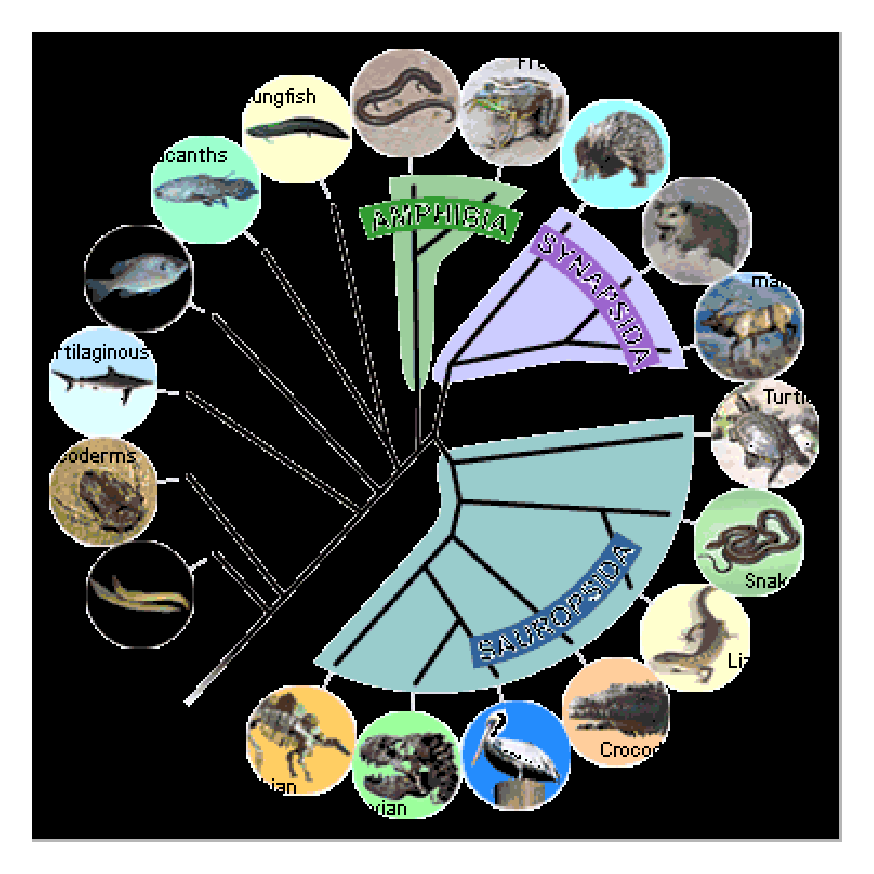

Supplement: Additional file 6 — ZIP files containing several folders, each of which with TreeSnatcher Plus snapshot files, the original image and a text file. [file 1471-2105-13-110-S6.zip › OtherTrees/vert_tree/vert_tree_o.PNG]
